# Identification of Multi-Target Anti-AD Chemical Constituents From Traditional Chinese Medicine Formulae by Integrating Virtual Screening and *In Vitro* Validation

**DOI:** 10.3389/fphar.2021.709607

**Published:** 2021-07-16

**Authors:** Baoyue Zhang, Jun Zhao, Zhe Wang, Pengfei Guo, Ailin Liu, Guanhua Du

**Affiliations:** Institute of Materia Medica, Chinese Academy of Medical Sciences and Peking Union Medical College, Beijing, China

**Keywords:** Alzheimer’s disease, multi-target, traditional Chinese medicine, virtual screening, molecular docking

## Abstract

Alzheimer’s disease (AD) is a neurodegenerative disease that seriously threatens the health of the elderly. At present, no drugs have been proven to cure or delay the progression of the disease. Due to the multifactorial aetiology of this disease, the multi-target-directed ligand (MTDL) approach provides an innovative and promising idea in search for new drugs against AD. In order to find potential multi-target anti-AD drugs from traditional Chinese medicine (TCM) formulae, a compound database derived from anti-AD Chinese herbal formulae was constructed and predicted by the anti-AD multi-target drug prediction platform established in our laboratory. By analyzing the results of virtual screening, 226 chemical constituents with 3 or more potential AD-related targets were collected, from which 16 compounds that were predicted to combat AD through various mechanisms were chosen for biological validation. Several cell models were established to validate the anti-AD effects of these compounds, including KCl, Aβ, okadaic acid (OA), SNP and H_2_O_2_ induced SH-SY5Y cell model and LPS induced BV2 microglia model. The experimental results showed that 12 compounds including Nonivamide, Bavachromene and 3,4-Dimethoxycinnamic acid could protect model cells from AD-related damages and showed potential anti-AD activity. Furthermore, the potential targets of Nonivamide were investigated by molecular docking study and analysis with CDOCKER revealed the possible binding mode of Nonivamide with its predicted targets. In summary, 12 potential multi-target anti-AD compounds have been found from anti-AD TCM formulae by comprehensive application of computational prediction, molecular docking method and biological validation, which laid a theoretical and experimental foundation for in-depth study, also providing important information and new research ideas for the discovery of anti-AD compounds from traditional Chinese medicine.

## Introduction

Alzheimer’s disease (AD) is a neurodegenerative disease with unclear etiology and pathogenesis (Lane et al., 2018), which is usually characterized by progressive loss of short-term memory, damage of language function, accompanied by spatial discrimination impairment, agnosia, and even personality and behavioral changes with the aggravation of disease (Albert et al., 2011). AD, which is more common in people over 65 years old with an increased incidence with age, has become one of the major diseases that seriously threaten the health of the elderly (Pan and Nicolazzo, 2018; Jia et al., 2020). According to the statistics of the World Alzheimer Report 2019, the patients of AD worldwide are nearly 50 million, which is predicted to reach 152 million by 2050. In recent decades, many pharmaceutical platforms have invested hundreds of millions of dollars in the research and development of anti-AD drugs (Cummings et al., 2016; Goldman et al., 2018), but only cholinesterase inhibitors and glutamate receptor blockers can alleviate the symptoms of AD, which still cannot effectively delay the disease progression (Hampel et al., 2018). So far, about 200 clinical trials of anti-AD drugs have been terminated due to ineffective treatment (Cai et al., 2018).

Three main pathological features of AD are senile plaque (SP), neurofibrillary tangles (NFT) and neutropenia (Long and Holtzman, 2019). Although the pathogenesis of AD remains unclear, a large number of clinical and preclinical experiments have shown that the occurrence and development of AD were closely related to Aβ toxic injury, abnormal phosphorylation of tau protein, inflammatory reaction, free radical damage, and so on (Chiti and Dobson, 2017; Li et al., 2018). According to the preliminary studies, the pathogenesis of AD was mainly related to Aβ plaques, but the therapeutic strategies aimed at reducing β-amyloid protein failed one after another (Loureiro et al., 2020; Knopman et al., 2021). Similarly, there was no breakthrough in the study on the phosphorylation of tau protein (Hane et al., 2017; Morris et al., 2018). With the various explorations of AD, some studies have suggested that the pathogenesis of AD was the interrelation of various mechanisms, which lead to a series of complex pathophysiological processes and the irreversible decline in cognitive function with the progression of the disease. Therapy targeting only a single target has little effect on this progressive cognitive loss (Canter et al., 2016), so there is an urgent need to formulate effective therapeutic measures for AD. It is of profound significance to find anti-AD drugs that can not only effectively improve symptoms but also delay the pathological process aiming at the new or multiple targets of AD (Savelieff et al., 2019). Our laboratory established an anti-AD multi-target prediction platform with 52 targets and 208 prediction models by adopting machine learning algorithms in the early stage, which can be applied to predict multi-target anti-AD compounds (Fang et al., 2015).

The application of multi-functional compounds regulating a variety of pathological characteristics has become an effective treatment method (Derrick et al., 2016; Rollo et al., 2016). Traditional Chinese medicine (TCM), the precious wealth of China, has the unique characteristics of multi-components, multi-targets and multi-pathways in the treatment of complex diseases. Extracting active components from TCM and then developing new drugs through structural modification has attracted much attention in recent years (Cheung, 2011; Yang et al., 2017). AD belongs to the category of “forgetfulness” and “dementia” in the field of TCM (Liu et al., 2014). Many classical prescriptions and clinical formulae provided effective methods for the treatment of AD and their effects were mostly confirmed in experimental studies (Hu and Sun, 2017). Emerging evidence has confirmed that the mechanisms of TCM treatment for AD include improving Aβ plaques, abnormal phosphorylation of tau protein, neuroinflammation, and oxidative stress (Tobore, 2019), as well as the improvement of microglia activity (Xia et al., 2017), regulation of heat shock protein pathway, MAPK pathway, mitochondrial membrane potential and release of cytochrome C, and so on (Zong et al., 2016; Dinda et al., 2019). With the development of TCM modernization in recent years, several TCM databases have been established at home and abroad, such as TCMID, CTD, TCMSP, HIT, TCMDB@taiwan (Xie et al., 2015), which are beneficial to the research on AD treatment with TCM.

Based on TCM formulae application database, natural products database and anti-AD multi-target prediction platform established in our laboratory, this study predicted the AD-related target-compound interaction in order to find the potential multi-target constituents from TCM formulae for the prevention and treatment of AD. Furthermore, a variety of AD-related cell models were established to verify the effect of potentially active constituents *in vitro*. This study provides important information for the discovery of new drugs in traditional Chinese medicine formulae for AD treatment.

## Materials and Methods

### Drugs and Reagents

Human neuroblastoma SH-SY5Y cells and mouse microglia BV2 cells were provided by Institute of Basic Medical Sciences (IBMS), Chinese Academy of Medical Sciences and Peking Union Medical College (CAMS and PUMC) (Beijing, China). Dulbecco’s modified eagle’s medium (DMEM) and fetal bovine serum (FBS) were bought from Thermo Fisher Scientific, Inc. (Waltham, MA, United States). 3-(4,5-dimethylthiazolyl-2)-2,5-diphenyltetrazoliumbromide (MTT), KCl, hydrogen peroxide (H_2_O_2_), L-glutamic sodium (MSG), sodium nitroprusside dihydrate (SNP), okadaic acid (OA), amyloid β-protein fragment 1–42 (Aβ), and lipopolysaccharides (LPS) were purchased from Sigma-Aldrich (St. Louis, MO, United States). L-Arctigenin, Dihydrocapsaicin, Rhapontigenin, 6-Shogaol, 6-Gingerol, 10-Gingerol, Isorhapontigenin, Nonivamide and Flavokawain B were bought from Target Molecule Corp. Demethoxycurcumin, Bisdemethoxycurcumin, Carnosol, Cardamonin, Matairesinol, Bavachromene, 3,4-Dimethoxycinnamic acid were purchased from Yuanye Bio-Technology (Shanghai, China). NO assay kit was acquired from Beyotime Institute of Biotechnology (Shanghai, China).

### Collection of TCM Formulae and Compounds

TCM formula is the basic form of TCM used in the prevention and treatment of diseases clinically, which refers to a quantitative mixture of several specific Chinese herbal medicine plants. The TCM formula contain a large number of chemical constituents, which are the material basis of the interaction of multiple targets related to the disease. The collection of traditional Chinese medicine formulae is mainly through the following two ways: 1) searching traditional Chinese medicine formulae with the key words of “dementia”, “forgetfulness” or “stupidity” through the modern prescriptions application database (http://cowork.cintcm.com/engine/login_do.jsp?u=guest&p=guest321&cnid=12895); 2) browsing China National Knowledge Infrastructure (CNKI) and PubMed database to collect traditional Chinese medicine formulae related to the treatment of AD. On this basis, 125 TCM formulae were collected and the frequency of each herb contained in the formulae was counted and a follow-up study was carried out on Chinese herbal medicine with a frequency greater than or equal to 3. The chemical compounds in these herbal plants were collected from the latest edition of China Natural Products Chemical Composition Database (http://pharmdata.ncmi.cn/cnpc/) and Traditional Chinese Medicine Systems Pharmacology (TCMSP) database (http://ibts.hkbu.edu.hk/LSP/tcmsp.php).

### Molecular Fingerprint Calculation

It is necessary to calculate the ECFP_6 molecular fingerprint and MACCS molecular fingerprint for the data preparation of traditional Chinese medicine compound data set before applying the anti-AD target prediction system to predict the potential targets. ECFP_6 molecular fingerprint, which belongs to the molecular characteristics of circular topological fingerprint design, is widely used in molecular similarity search and quantitative structure-activity relationship (QSAR) model, was calculated in Discovery Studio 2018 (SanDiego, CA, United States) software. MACCS molecular fingerprint, which is composed of 166 species substructures, was calculated with PaDEL Descriptor (Yap, 2011) software.

### Target Identification of Constituents From TCM Formulae

We performed target identification *via* our *in-house* prediction platform. The multi-target anti-AD compound-protein interaction (CPI) prediction mt-QSAR model was established earlier in our laboratory. Aiming at 52 AD-related targets, a total of 208 prediction models were established by adopting two machine learning algorithms of naïve Bayesian (NB) and recursive partitioning (RP) based on ECFP_6 and MACCS molecular fingerprints, which meant four binary prediction models of NB (ECFP_6), NB (MACCS), RP (ECFP_6) and RP (MACCS) were established for each target (Fang et al., 2015). Through the previous analysis, two optimal models, NB (ECFP_6) model and RP (MACCS) model were applied for target prediction in the present study, and one chemical constituent was considered to be active to the AD-related targets when it was predicted to be positive by both models.

### Cell Model Verifications

#### Cell Culture

SH-SY5Y cells and BV2 microglia were cultured in DMEM medium mixed with 10% FBS in a humidified incubator supplied with 95% air and 5% CO_2_ at 37°C.

#### Cell Viability Assay

Cells in each group were seeded in 96-well plates and cultured for 24 h, then the culture medium were placed by fresh medium with compounds of different concentrations. After 2 h, the cells were incubated with Aβ, OA, KCl, SNP, H_2_O_2_, LPS, respectively for another 24 h. Subsequently, the culture medium was removed and 100 μl MTT solution was added to each well. After incubation at 37°C for 4 h, 100 µl DMSO was added to each well to solubilize the crystallization and optical density (OD) was measured at 570 nm.

#### Nitric Oxide Assay

NO assay was carried out in opaque 96-well plates. The reaction system is composed of 50 μl cell supernatant or standard solution, 50 μl Griess reagent 1 and 50 μl Griess reagent 2. After mixing, the absorbance was measured at 540 nm. The configuration of standard NaNO_2_: dilute 1M standard to 100, 50, 25, 12.5, 6.25, 3.125, 0 µM with DMEM.

### Molecular Docking

The representative chemical constituent Nonivamide with potential anti-AD activity was adopted for molecular docking with corresponding predicted targets. The crystal structures of these target proteins complexed with ligands were retrieved from Protein Data Bank (PDB) database. The molecular docking program was conducted in the CDOCKER module in Discovery Studio 2018 (San Diego, CA, United States). Firstly, the water was removed and the protein crystal structure was further processed to model missing loop regions, calculate protein ionization and protonate the protein structure. The active pocket was defined by the co-crystallized ligand and the binding site sphere: 3D3L (-3.929336 -2.890014 -6.237888), 4EY7 (-13.984925 -43.974718 27.895130), 6PSJ (-2.849034 -28.613768 23.219457), 1S2Q (55.356471 145.972143 19.261433), 5IKR (55.356471 145.972143 19.261433). After setting the docking parameters of CDocker module, the co-crystallized ligand in the crystal structure were extracted and re-docked to the predefined active pocket. Meanwhile the root-mean-square deviation (RMSD) value between the molecular conformation of the docked ligand and the initial conformation in the crystal structure was calculated. The molecular docking result was considered to be reliable if the value of RMSD was less than 2.5. If there is no co-crystallized ligand, the binding site was defined from PDB site records. On this basis, the potential binding modes of active chemical constituents to predicted targets were predicted and analyzed by molecular docking.

## Results

### Anti-AD TCM Compounds Data Set Construction

Based on the data mining of existing databases and literature investigation, we collected 125 traditional Chinese medicine formulae significantly related to AD, such as BushenYizhi decoction, Tongbi Yinao decoction, Xuefu Zhuyu decoction, etc., from which 102 herbs with a frequency greater than or equal to 3 were selected, such as *Panax Ginseng C. A. Mey.*, *licorice* and so on. Detailed information of the 102 herbs was shown in Supplementary Table S1. The chemical constituents of the above 102 herbs were obtained from databases and the duplicate structures were removed, with 8,426 compounds left. The detailed information of the 8,426 compounds was listed in Supplementary Table S2. The chemical constituents were then screened as follows: firstly, the traditional Chinese medicine constituents were analyzed by Lipinski rules in Discovery Studio 2018 and the compounds would be excluded if they met any of the following conditions: 1) hydrogen-bonded donors were more than 8; 2) hydrogen-bonded receptors were more than 12; 3) molecular weight was more than 600; 4) octanol-water partition coefficient LogP was more than 6. As for most traditional Chinese medicines are administered orally and the blood-brain barrier permeability is an important factor affecting the effects of central nervous system drugs, we further predicted and evaluated the oral bioavailability and blood-brain barrier permeability of constituents in traditional Chinese medicine formulae by using ADMET descriptors in Discovery Studio 2018 and removed the constituents that met any of the following rules: 1) the solubility was less than −8 (very low); 2) the permeability of the blood-brain barrier was equal to 3 (low permeability); 3) inhibitory activity to CYP2D6 enzyme; 4) the absorptive availability was equal to 3 (the absorptivity was very poor). Furthermore, we predicted the toxicity of the chemical constituents by using the ADMET Predictor software (Mehta et al., 2018; Wan et al., 2020) and ruled out the constituents whose TOX_Risk was more than 3.3 or ADMET_Risk was more than 7.5. Finally, an anti-AD TCM compound data set composed of 3,508 constituents was constructed.

### Anti-AD Multi-Target Drug Prediction Platform

**FIGURE 1:**
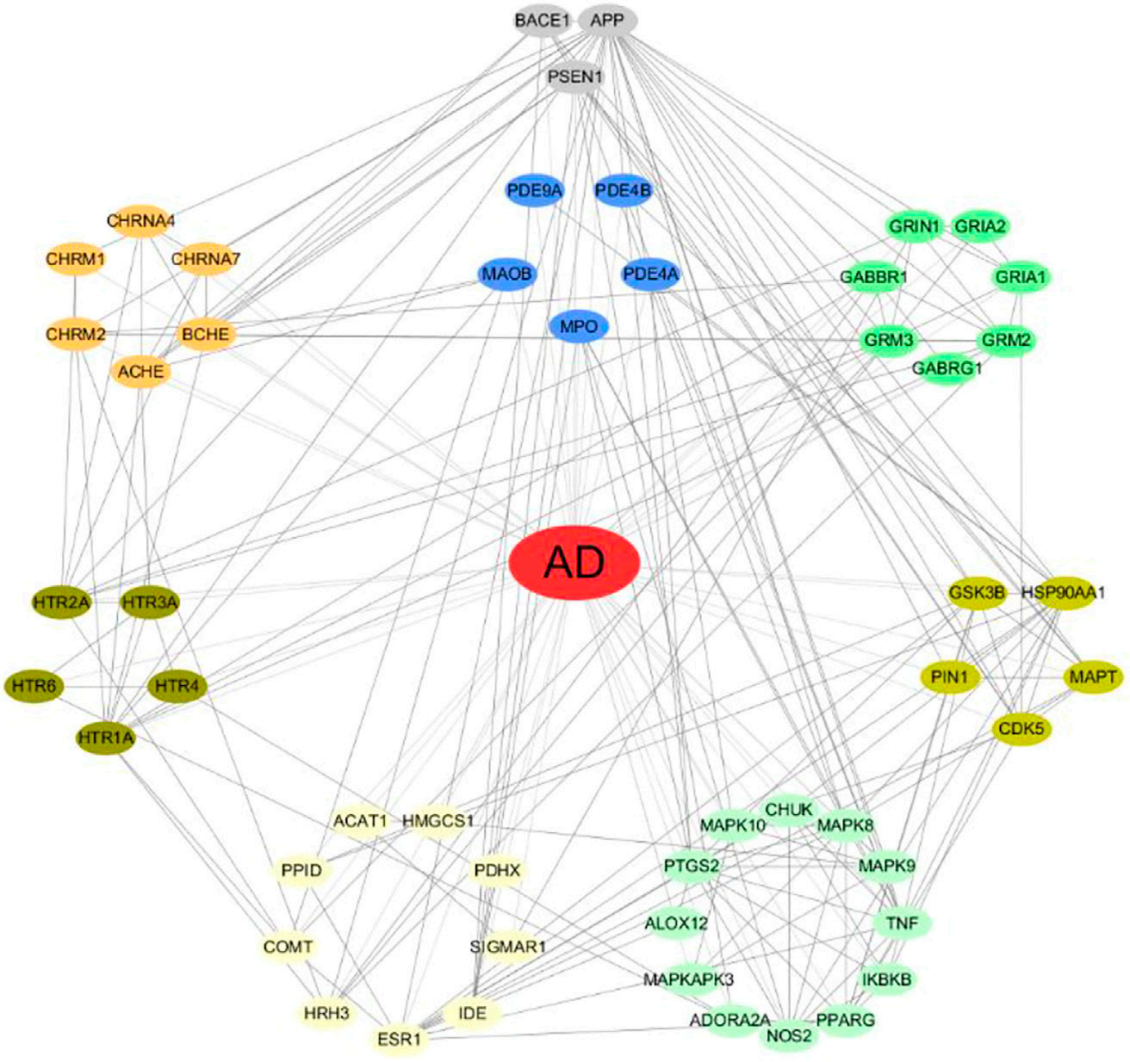
The analysis of protein-protein interaction network of AD-related targets by STRING.

The anti-AD multi-target drug prediction platform established in our laboratory contains 52 protein targets. The relationship among these targets were predicted by STRING database (https://string-db.org/) to construct the protein-protein interaction network, as shown in Figure 1. 52 AD-related targets were counted and classified, as shown in Table 1. The functions related to these targets were involved in various aspects of the occurrence and development of AD including cholinergic system dysfunction, β-amyloid protein (Aβ), tau protein hyperphosphorylation, glutamate/GABA system dysfunction, serotoninergic system dysfunction, oxidative stress, neuroinflammation, mitochondrial dysfunction and so on.

**TABLE 1:** Basic information of AD-related targets.

Target symbol	Target name	Classification of targets
ACHE	Acetylcholinesterase	cholinergic system dysfunction
BCHE	Butyrylcholinesterase	cholinergic system dysfunction
CHRM1	muscarnic m1 receptor	cholinergic system dysfunction
CHRM2	muscarnic m2 receptor	cholinergic system dysfunction
CHRNA4	nicotinic acetylcholine receptor α4	cholinergic system dysfunction
CHRNA7	nicotinic acetylcholine receptor α7	cholinergic system dysfunction
GRIA1	α-amino-3-hydroxy-5-methyl-4-isoxa-zolep-propionate 1 receptor	glutamate/GABA system dysfunction
GRIA2	α-amino-3-hydroxy-5-methyl-4-isoxa-zolep-propionate 2 receptor	glutamate/GABA system dysfunction
GABRG1	gamma-aminobutyric acid A receptor	glutamate/GABA system dysfunction
GABBR1	gamma-aminobutyric acid B receptor	glutamate/GABA system dysfunction
GRM2	metabotropic glutamate receptor 2	glutamate/GABA system dysfunction
GRM3	metabotropic glutamate receptor 3	glutamate/GABA system dysfunction
GRIN1	N-methyl-D-aspartate receptor	glutamate/GABA system dysfunction
APP	beta-amyloid precursor protein	aggregates of amyloid-β peptide
BACE1	β-secreatase	aggregates of amyloid-β peptide
PSEN1	gamma seretase	aggregates of amyloid-β peptide
HSP90AA1	heat shock protein 90	hyper-phosphorylated tau
CDK5	cyclin-dependent kinase 5	hyper-phosphorylated tau
GSK3B	glycogen synthase kinase 3 beta	hyper-phosphorylated tau
MAPT	microtubule-associated protein tau	hyper-phosphorylated tau
PIN1	peptidyl prolyl cis/trans Isomerases	hyper-phosphorylated tau
HTR1A	5 hydroxytryptamine 1A receptor	serotonergic system dysfunction
HTR2A	5 hydroxytryptamine 2A receptor	serotonergic system dysfunction
HTR3A	5 hydroxytryptamine 3A receptor	serotonergic system dysfunction
HTR4	5 hydroxytryptamine 2 receptor	serotonergic system dysfunction
HTR6	5 hydroxytryptamine 6 receptor	serotonergic system dysfunction
MAOB	Monoamine oxidase B	oxidative stress
MPO	Myeloperoxidae	oxidative stress
PDE4A	phosphodiesterase type 4A	oxidative stress
PDE4B	phosphodiesterase type 4B	oxidative stress
PDE9A	phosphodiesterase type 9A	oxidative stress
MAPK8	c-Jun N-terminal kinase-1	neuroinflammation
MAPK9	c-Jun N-terminal kinase-2	neuroinflammation
MAPK10	c-Jun N-terminal kinase-3	neuroinflammation
MAPKAPK3	p38α mitogen-activated protein kinase	neuroinflammation
CHUK	nuclear factor kappa-B kinase alpha	neuroinflammation
IKBKB	nuclear factor kappa-B kinase beta	neuroinflammation
NOS2	inducible nitric oxide synthase	neuroinflammation
PPARG	Peroxisome proliferator-activated receptor gamma	neuroinflammation
TNF	tumor necrosis factor alpha	neuroinflammation
ADORA2A	A2A adenosine receptor	neuroinflammation
ALOX12	12-lipoxygenase	neuroinflammation
PTGS2	cyclooxygenase-2	neuroinflammation
PPID	Cyclophilin D	mitochondrial dysfunction
PDHX	pyruvate dehydrogenase	mitochondrial dysfunction
ACAT1	Cholesterol Acyltransferase	Other
COMT	catechol O-methyltransferase	Other
ESR1	estrogen receptor α	Other
HRH3	histamine H3 receptor	Other
HMGCS1	3-hydroxy-3-methyl glutaryl coenzyme A reductase	Other
IDE	insulin-degrading enzyme	Other
SIGMAR1	sigma-1 receptor	Other

### Good and Bad Fragments of AChE Inhibitors Given by NB Model

**FIGURE 2:**
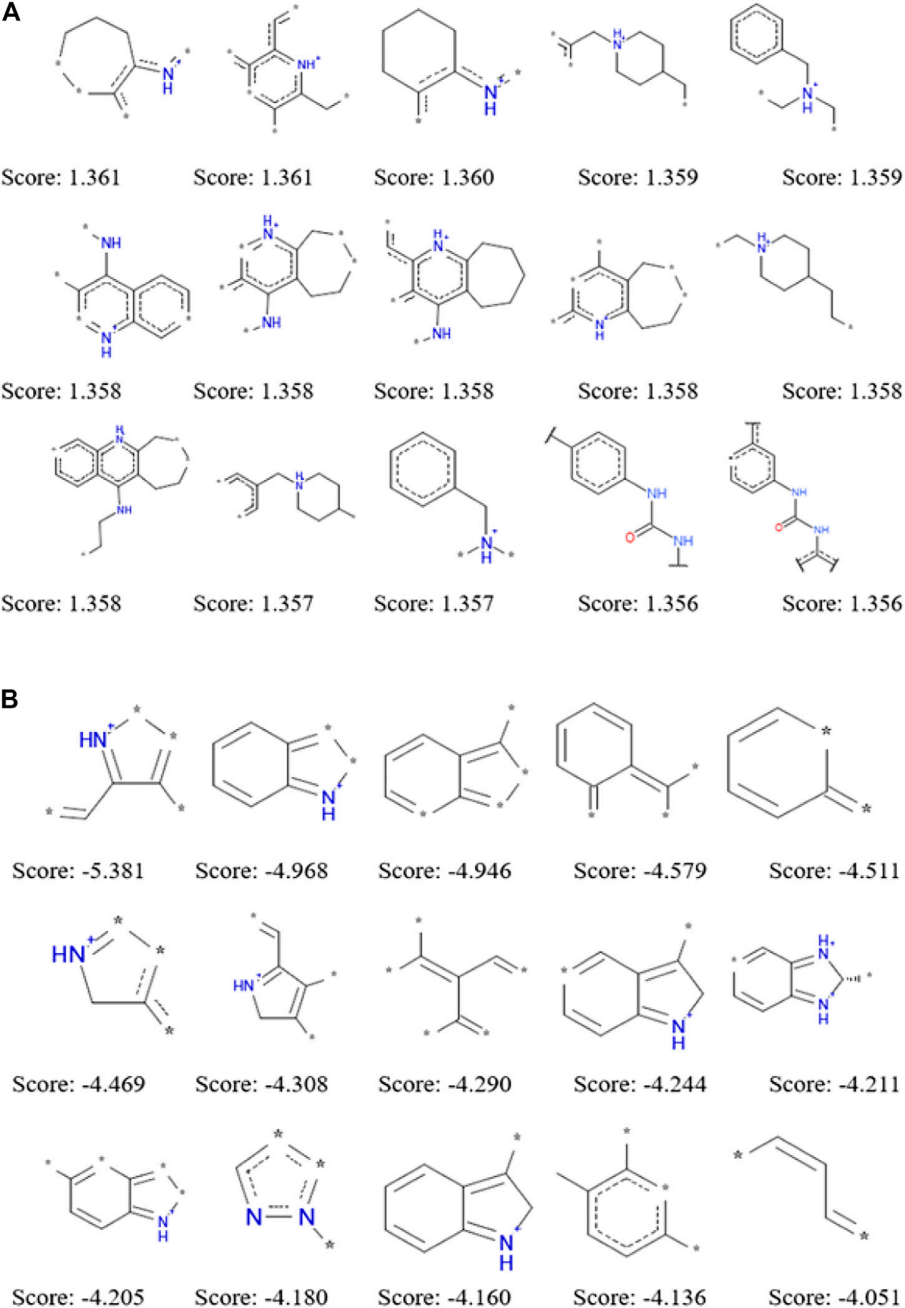
Examples of the top 15 good **(A)** and bad **(B)** fragments for ACHE inhibition as estimated by NB(ECFP_6) model. The Bayesian score (Score) is given for each fragment.

The application of ECFP_6 molecular fingerprint in the process of establishing naïve Bayesian model not only improve the prediction ability, but also analyze the dominant and inferior fragments that had a great influence on compounds to be active against a certain target. During the establishment of naïve Bayesian model for a specific target, each structural fragment was evaluated by Bayesian score. Structural fragments with higher the Bayesian scores make the greater contributions to becoming an active chemical constituent for a specific target. We summarized 15 dominant fragments and 15 inferior fragments to be ACHE inhibitors by the Bayesian score of structural fragments according to the NB (ECFP_6) model (Figure 2). The analysis of these good and bad fragments would help to guide the rational drug design of ACHE inhibitors. Through the analysis of 15 dominant fragments, we found that most of them contained one or more nitrogen atoms. Some of the nitrogen atoms existed in the six-or seven-membered heterocycles, while the other nitrogen atoms are linked with the six-membered ring structure or carbonyl structure. There were nine fragments containing one or more nitrogen atoms among the 15 inferior fragments, however these nitrogen atoms are not directly connected with the six-membered ring or carbonyl structure but existed in the five-membered heterocyclic ring, which suggested that the existence of nitrogen-containing five-membered ring was not conducive to inhibit ACHE. The good and bad fragments of other 51 targets were listed in the supplementary materials.

### Target Prediction for Anti-AD TCM Compounds

**FIGURE 3:**
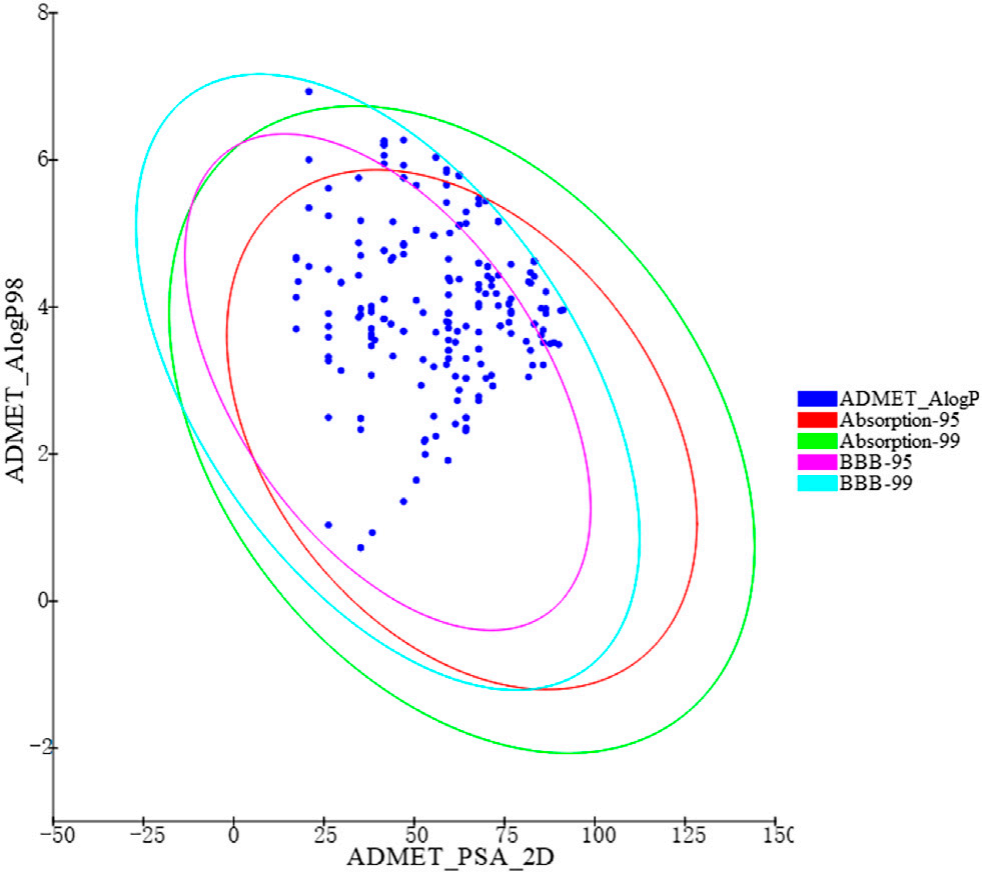
ADMET properties diversity distribution of 226 chemical constituents. ADMET_AlogP98: lipid-water partition coefficient; ADMET_PSA_2D: polar molecular surface area; 2D plots of ADMET_PSA_2D and ADMET_AlogP98 show 2 series of ellipses representing the 95 and 99% confidence regions of the blood-brain barrier permeability (BBB) model, and the human intestinal absorption (HIA) model 95 and 99% CI.

**FIGURE 4:**
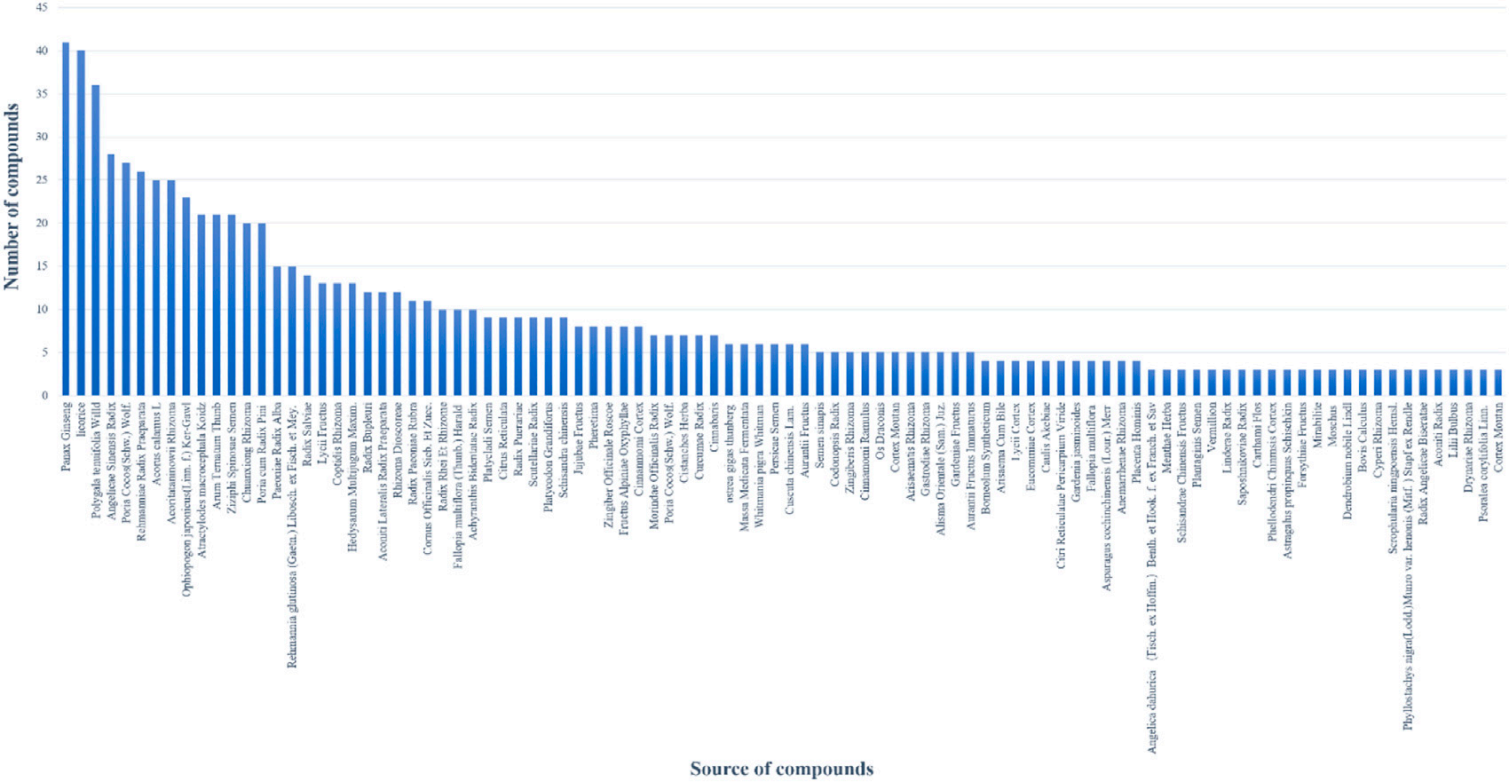
The number of potential active chemical constituents from different Chinese medicinal herbs.

**FIGURE 5:**
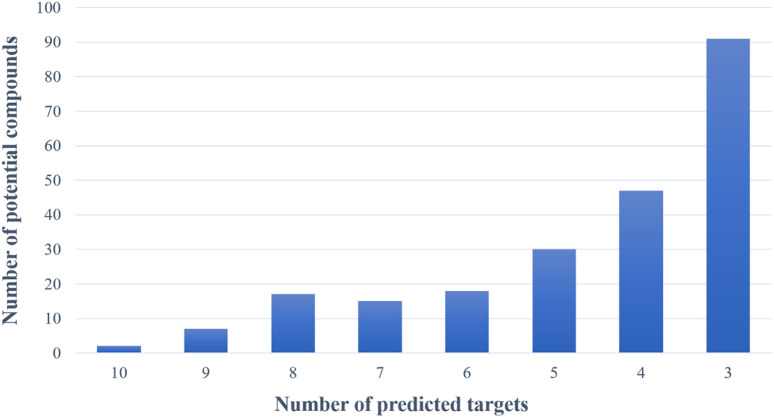
The number of potential active chemical constituents and the number of their predicted targets.

**FIGURE 6:**
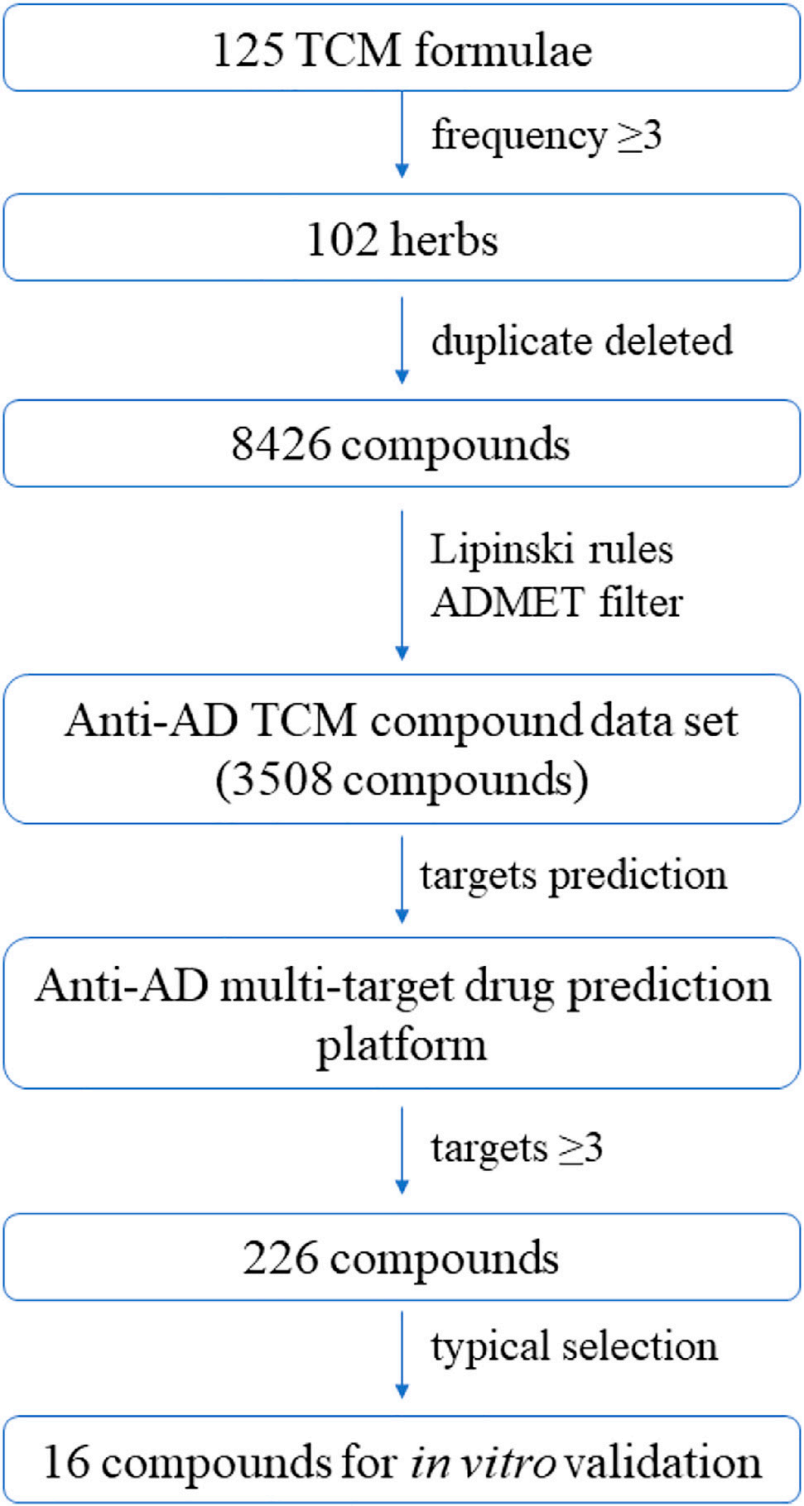
Workflow of the integrated virtual screening and drug screening approaches for anti-AD multi-target compounds from TCM formulae.

**FIGURE 7:**
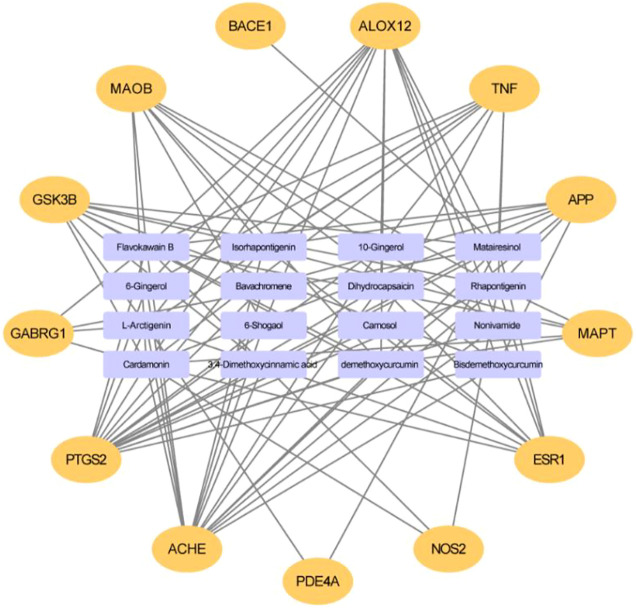
The compound-target network for potential anti-AD chemical constituents. The purple rectangle means chemical constituents, and the orange means predicted target.

Based on the virtual screening platform mentioned above, the data set of anti-AD TCM compounds established in this paper was predicted and screened. A total number of 226 chemical constituents with 3 or more potential active targets were statistically collected and the distribution of their ADMET parameters is shown in Figure 3. The detailed information of the 226 compounds was shown in Supplementary Table S3. These chemical constituents mainly included flavonoids, coumarins, lignans, alkaloids, anthraquinones and steroids. As shown in Figures 4, 5, the sources of the 226 chemical constituents and their predicted targets were counted. These chemical constituents were mainly from *Radix salviae*, *Panacis quinquefolii Radix*, *Zingiberis rhizoma*, *Radix bupleuri*, *licorice* and *Zingiber officinale Roscoe*. All of the chemical constituents were predicted to act on three or more targets in the platform, of which 59 could act on 6 or more AD-related targets. We selected 16 typical chemical constituents that might exert anti-AD effects through a variety of mechanisms and the detailed compound selection process was shown in Figure 6. We constructed a compound-target network of these chemical constituents by using Cytoscape-3.7.1 software (Figure 7). In this paper, several AD-related cell models were established to verify the activity of these 16 chemical constituents *in vitro*. The specific information of 16 chemical constituents is shown in Table 2.

**TABLE 2:** The 16 typical examples of multi-target compounds.

Compound name	Chemical structure	Predicted targets
L-Arctigenin	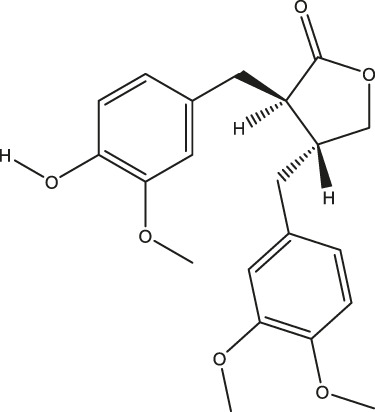	ALOX12, ACHE, PTGS2, ESR1, GABRG1, GSK3B, PDE4A, TNF
Dihydrocapsaicin	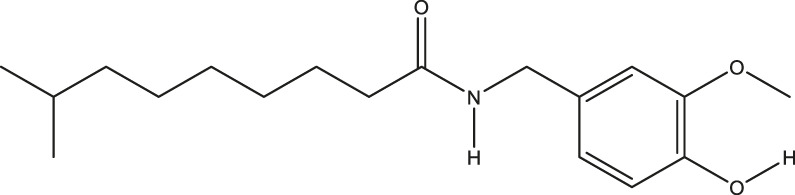	ALOX12, ACHE, PTGS2, ESR1
Rhapontigenin	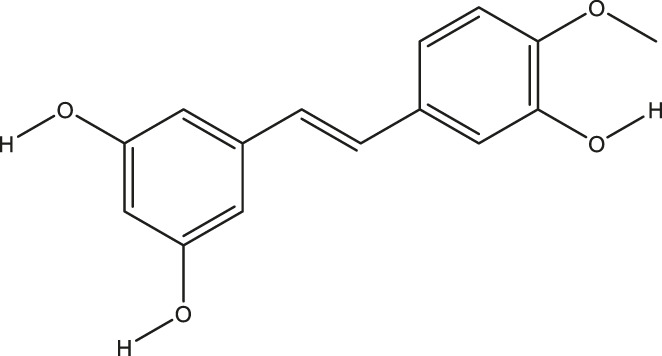	ALOX12, ACHE, APP, PTGS2, ESR1, GSK3B, NOS2, MAOB, MAPT, TNF
6-Shogaol	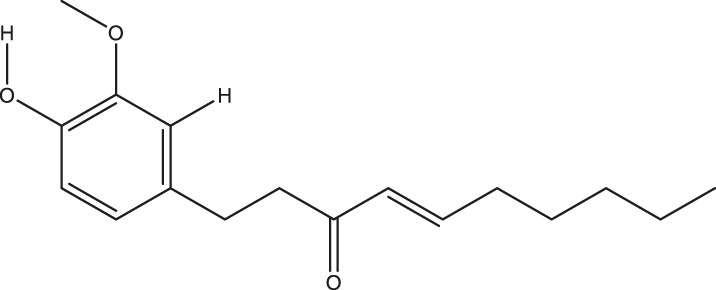	ALOX12, ACHE, PTGS2, MAOB
6-Gingerol	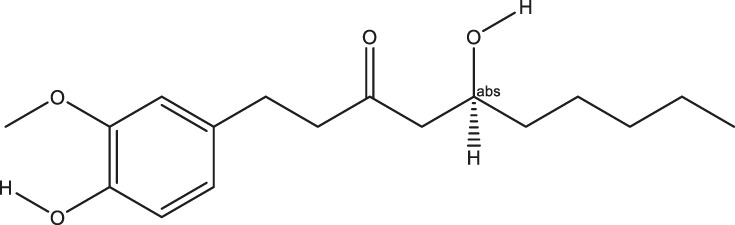	ALOX12, ACHE, PTGS2, TNF
10-Gingerol	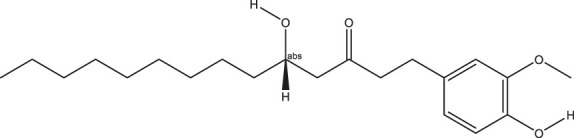	ALOX12, ACHE, PTGS2, TNF
Isorhapontigenin	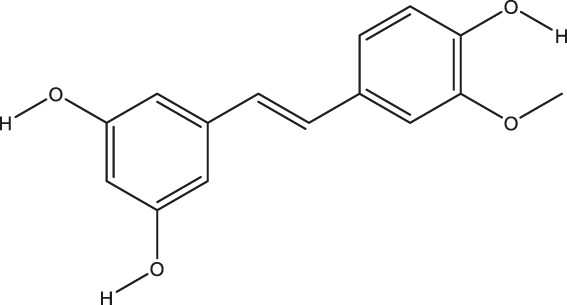	ALOX12, ACHE, APP, PTGS2, ESR1, GSK3B, MAOB, MAPT, TNF
Nonivamide	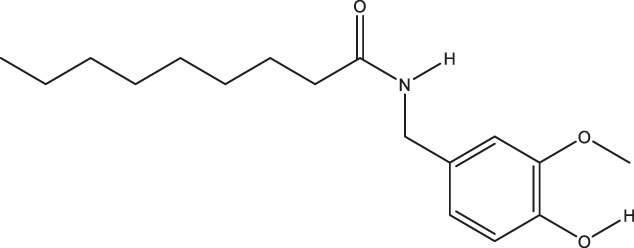	ALOX12, ACHE, PTGS2, ESR1, MAOB
Flavokawain B	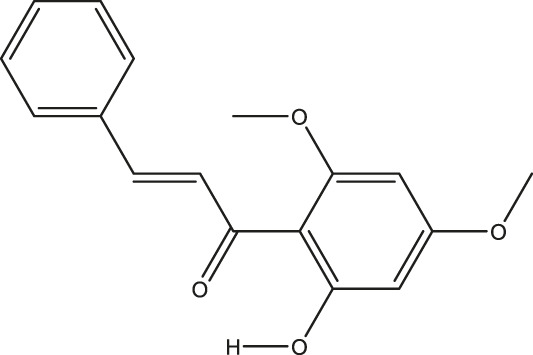	ALOX12, ACHE, APP, PTGS2, ESR1, GABRG1, GSK3B, NOS2, MAOB, MAPT, TNF
Demethoxycurcumin	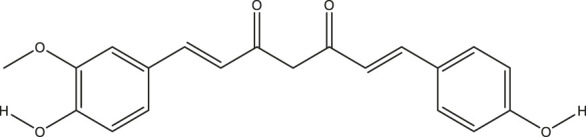	ALOX12, ACHE, APP, PTGS2, GSK3B, MAOB, MAPT
Bisdemethoxycurcumin	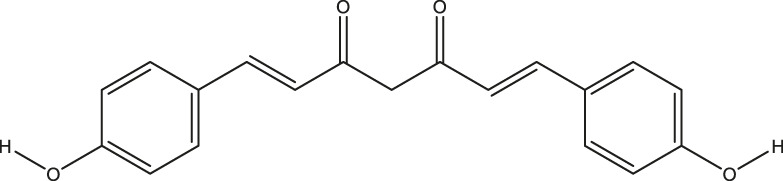	ALOX12, ACHE, APP, PTGS2, GSK3B, MAOB
Carnosol	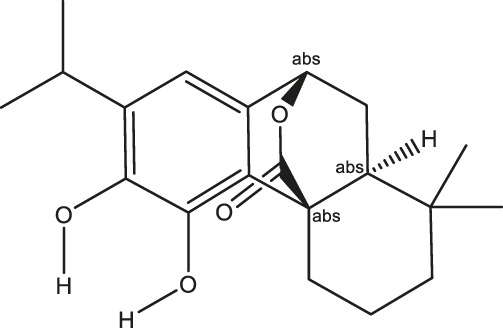	PTGS2, ESR1, TNF
Cardamonin	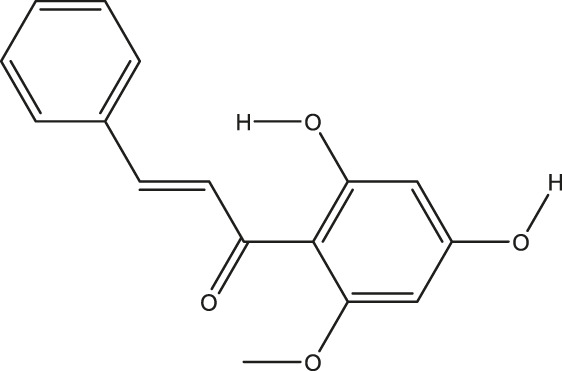	ALOX12, ACHE, APP, PTGS2, ESR1, GABRG1, GSK3B, NOS2, MAOB, MAPT, TNF
Matairesinol	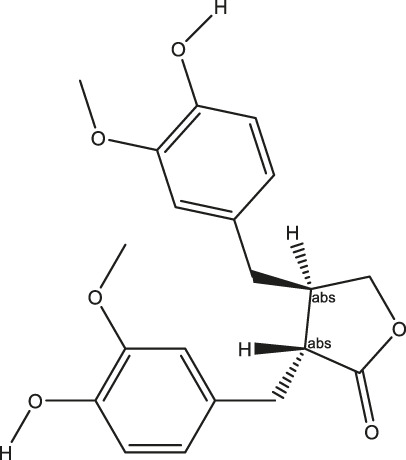	ALOX12, ACHE, BACE1, PTGS2, ESR1, GABRG1, GSK3B, PDE4A, TNF
Bavachromene	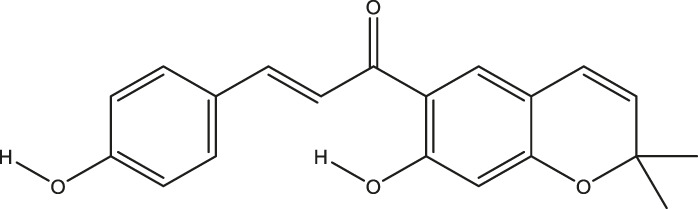	ACHE, APP, PTGS2
3,4-Dimethoxycinnamic acid	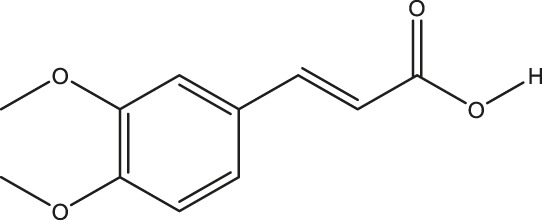	ACHE, APP, PTGS2, GSK3B

### Cell Models Verification

#### KCl-Induced Cytotoxicity of SH-SY5Y Cells

**FIGURE 8:**
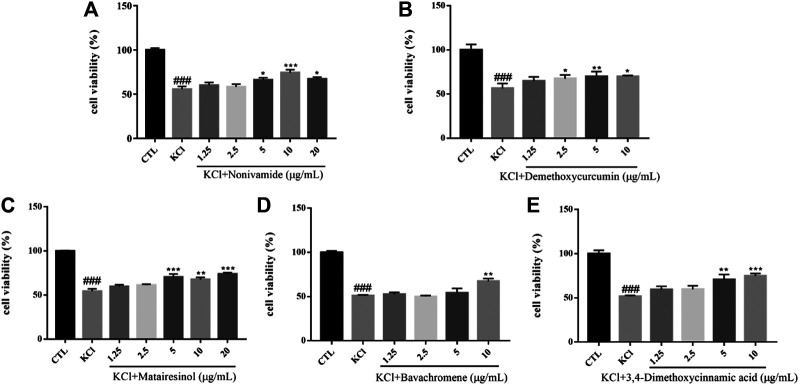
Various compounds suppressed KCl-induced cytotoxicity in SH-SY5Y cells. Cells were pretreated with different concentrations of Nonivamide **(A)**, Demethoxycurcumin **(B)**, Matairesinol **(C)**, Bavachromene **(D)**, 3,4-Dimethoxycinnamic acid **(E)** or 0.1% DMSO for 2 h and then incubated with or without KCl (80 mM) for 24 h and the cell viability was detected by MTT assay. Results are shown as the mean ± SEM and represent three independent experiments. ###*p* < 0.001 compared with control group, *p < 0.05, **p < 0.01, ***p < 0.001 compared with KCl treated group.

High concentration of potassium increases the sensitivity of calcium channels and increases the concentration of intracellular Ca^2+^. The brain damages caused by overload of free calcium are shown in the following aspects: 1) some protease and phospholipase were activated, resulting in the hydrolysis of proteins and phospholipids on the cell membrane and causing the destruction of cell membrane integrity; 2) ATP enzyme was activated, leading to a large amount of ATP consumption and hydrogen ions release, which may contribute to the decrease of intracellular pH and the destruction of lysosomal membrane. Subsequently, lysosomal enzymes hydrolyze proteins, nucleic acids and phospholipids, causing damage to brain cells; 3) a large amount of intracellular calcium accumulates in mitochondria to obstruct the function of oxidative phosphorylation, leading to a lack of intracellular energy production; 4) the disorder of calcium regulation may cause cell death by changing the expression of some cell death-related genes (Abdel-Hamid and Tymianski, 1997). In short, the overload of cytosolic free calcium in brain cells caused by excessive calcium influx could bring about a series of reactions, which eventually leads to delayed neuronal degeneration and necrosis. The viability of SH-SY5Y cells induced by 80 mM KCl for 24 h was significantly lower than that of the control group (*p* < 0.001). Pretreated with Nonivamide, Demethoxycurcumin, Matairesinol, Bavachromene, and 3,4-Dimethoxycinnamic acid, the cell viability was significantly increased and the injury caused by KCl was inhibited to some extent (Figure 8).

#### Aβ-Induced Cytotoxicity of SH-SY5Y Cells

**FIGURE 9:**
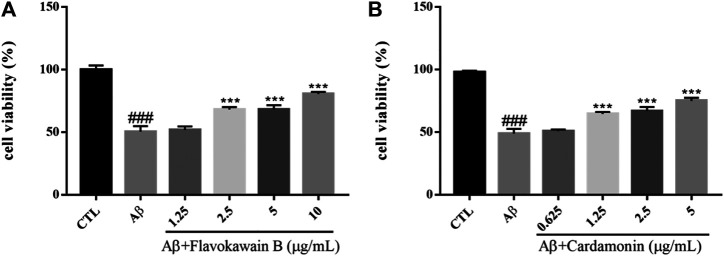
Various constituents suppressed Aβ-induced cytotoxicity in SH-SY5Y cells. Cells were pretreated with different concentrations of Nonivamide **(A)**, 3,4-Dimethoxycinnamic acid **(B)** or 0.1% DMSO for 2 h and then incubated with or without Aβ_1–42_ (10 μM) for 24 h and the cell viability was detected by MTT assay. Results are shown as the mean ± SEM and represent three independent experiments. ###*p* < 0.001 compared with control group, ***p < 0.001 compared with Aβ treated group.

Amyloid cascade hypothesis has been in the dominant position of AD pathogenesis for a long time. Aβ has always been considered to be the main toxic substance causing AD, which eventually leads to cognitive dysfunction by causing a series of malignant events such as neurofibrillary tangles and neuronal loss. Aβ is a polypeptide composed of 39–43 amino acids. A small amount of soluble Aβ has no neurotoxicity and exists normally in human cerebrospinal fluid and plasma. If beta amyloid transforms into insoluble precipitation, some intelligence-related structures in the brain, such as hippocampus and some cortical regions, would be damaged, accompanied by a large number of neuronal fibrous starch degeneration, pathological changes characterized by senile plaque, eventually causing neuronal loss (Yankner et al., 1990). At present, it is considered that the neurotoxic effects of aggregated Aβ mainly include enhancing and amplifying various nociceptive stimuli such as the cell damage effects of excitotoxicity and free radicals, as well as the damage of hypoglycemia to neurons, and direct cytotoxicity and so on. In addition, Aβ can destroy the coupling of poisonous alkaloid receptor with G protein, resulting in the accumulation of phosphatidylinositol and the release of internal calcium (Kelly et al., 1996). Aβ_1–42_ was dissolved in DMEM medium and incubated at 37°C for 48 h before incubation with cells. The survival rate of SH-SY5Y cells injured by 10 μM Aβ_1–42_ for 24 h was significantly lower than that of the control group (*p* < 0.001). However, Flavokawain B and Cardamonin could enhance the cell viability of the model group in a dose-dependent manner and protect SH-SY5Y cells against Aβ (Figure 9).

#### OA-Induced Cytotoxicity of SH-SY5Y Cells

**FIGURE 10:**
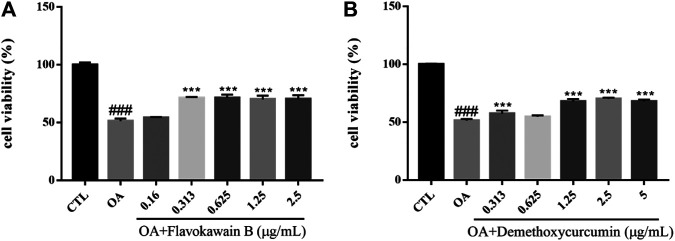
Various constituents suppressed OA-induced cytotoxicity in SH-SY5Y cells. Cells were pre-treated with different concentrations of Flavokawain B **(A)**, Demethoxycurcumin **(B)** or 0.1% DMSO for 2 h and then incubated with or without OA (0.2 μM) for 24 h and the cell viability was detected by MTT assay. Results are shown as the mean ± SEM and represent three independent experiments. ###*p* < 0.001 compared with control group, ***p < 0.001 compared with OA treated group.

Okadaic acid (OA) can induce hyperphosphorylation of tau protein in SH-SY5Y cells. Human neuroblastoma (SH-SY5Y) cells have the ability to differentiate into neuron-like cells, which can express tau protein internally with high concentration of protein kinases and phosphatases that regulate tau protein phosphorylation (Agholme et al., 2010). OA extracted from black sponge is a potent selective inhibitor of protein phosphatase PP1 and PP2A, which can inhibit the activity of phosphatase, hyperphosphorylate tau protein and cause oxidative damage. At the same time, OA promote the deposition of Aβ and cause neuronal degeneration, synaptic loss and memory impairment, leading to AD-like pathological features (Kamat et al., 2014). The viability of SH-SY5Y cells was measured by MTT assay. The survival rate of SH-SY5Y cells injured by 0.2 mM OA for 24 h was significantly lower than that of the control group (*p* < 0.001). After the treatment with chemical constituents with different concentrations, the survival rate of cells pretreated with Flavokawain B, Demethoxycurcumin increased and the injury caused by OA was significantly inhibited (Figure 10).

#### SNP-Induced Cytotoxicity of SH-SY5Y Cells

**FIGURE 11:**
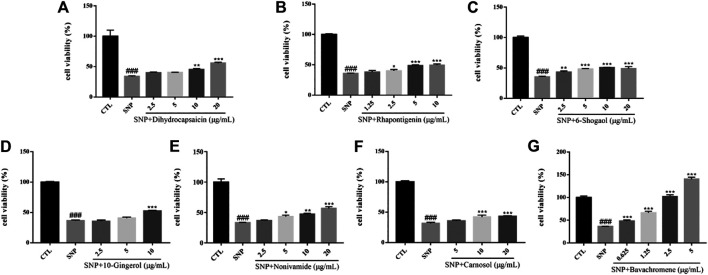
Various constituents suppressed SNP-induced cytotoxicity in SH-SY5Y cells. Cells were pre-treated with different concentrations of Dihydrocapsaicin **(A)**, Rhapontigenin **(B)**, 6-Shogaol **(C)**, 10-Gingerol **(D)**, Nonivamide **(E)**, Carnosol **(F)**, Bavachromene **(G)** or 0.1% DMSO for 2 h and then incubated with or without SNP (600 μM) for 24 h and the cell viability was detected by MTT assay. Results are shown as the mean ± SEM and represent three independent experiments. ###*p* < 0.001 compared with control group, *p < 0.05, **p < 0.01, ***p < 0.001 compared with SNP treated group.

Nitric oxide (NO) is an important signal molecule in neurons, but excess NO has neurotoxicity and participates in cerebral ischemic injury and a series of neurodegenerative diseases. Sodium nitroprusside (SNP) is employed as a NO donor *in vitro* and has been used as a compound to induce neuronal injury model in many experiments. Oxidative damage caused by nitroxide free radicals of NO is considered to be an important way for NO to induce neuronal injury (Calabrese et al., 2007). The survival rate of SH-SY5Y cells injured by 600 µM SNP for 24 h was significantly lower than that of the control group (*p* < 0.001). After pretreatment with chemical constituents of different concentrations, the survival rate of cells treated with Dihydrocapsaicin, Rhapontigenin, 6-Shogaol, 10-Gingerol, Nonivamide, Carnosol, Bavachromene increased and the injury caused by SNP was inhibited (Figure 11).

#### H_2_O_2_-Induced Cytotoxicity of SH-SY5Y Cells

**FIGURE 12:**
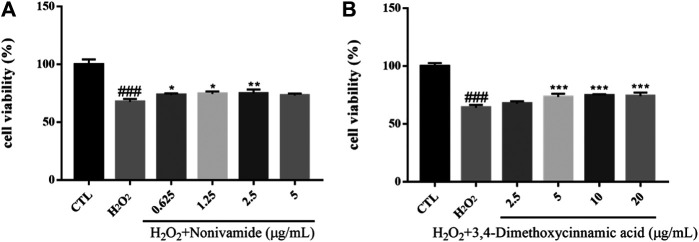
Various constituents suppressed H_2_O_2_-induced cytotoxicity in SH-SY5Y cells. Cells were pre-treated with different concentrations of Nonivamide **(A)**, 3,4-Dimethoxycinnamic acid **(B)** or 0.1% DMSO for 2 h and then incubated with or without H_2_O_2_ (400 μM) for 24 h and the cell viability was detected by MTT assay. Results are shown as the mean ± SEM and represent three independent experiments. ###*p* < 0.001 compared with control group, *p < 0.05, **p < 0.01, ***p < 0.001 compared with H_2_O_2_ treated group.

Hydrogen peroxide (H_2_O_2_), as a generation source of exogenous free radical, can simulate oxidative damage of SH-SY5Y cells to establish an oxidative stress injury model of neurons *in vitro*. Oxidative damage means that there are excessive reactive oxygen free radicals and active nitrogen free radicals when the body is subjected to various harmful stimuli and the effect of oxidation is stronger than that of antioxidation, resulting in an imbalance between the oxidation system and antioxidant system, finally leading to tissue damage. Oxidative damage plays an important role in the pathogenesis of central nervous system diseases, which can cause lipid peroxidation, protein degeneration and DNA damage, decrease mitochondrial membrane potential, destroy cell function and integrity, and eventually result in cell apoptosis or necrosis (Gorman et al., 1996; Hsuuw et al., 2005). The viability of SH-SY5Y cells was measured by MTT assay. The survival rate of SH-SY5Y cells injured by 400 μM H_2_O_2_ for 24 h was significantly lower than that in the control group (*p* < 0.001). After the treatment of chemical constituents with different concentrations, the survival rate of cells treated with Nonivamide (A), 3,4-dimethoxycinnamic acid (B) increased and the injury caused by H_2_O_2_ was inhibited (Figure 12).

#### LPS-Induced Inflammation of BV2 Cells

**FIGURE 13:**
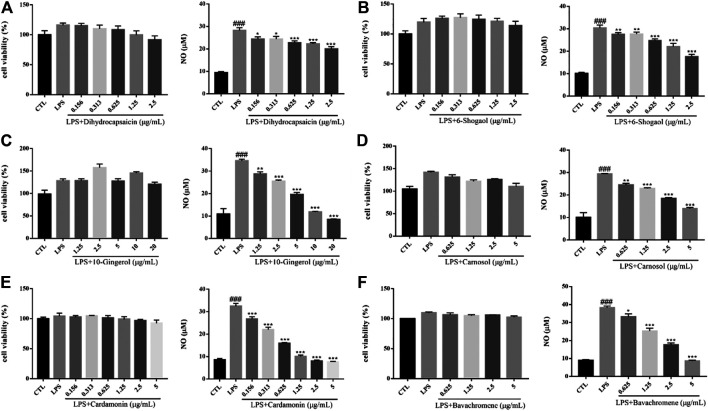
Various constituents suppressed LPS-induced cytotoxicity in BV2 cells. Cells were pre-treated with different concentrations of Dihydrocapsaicin **(A)**, 6-Shogaol **(B)**, 10-Gingerol **(C)**, Carnosol **(D)**, Cardamonin **(E)**, Bavachromene **(F)** or 0.1% DMSO for 2 h and then incubated with or without LPS (500 ng/ml) for 24 h and the cell viability was detected by MTT assay (*n* = 3). NO concentration was detected by Griess reagent kit (*n* = 3). ###*p* < 0.001 compared with control group, *p < 0.05, **p < 0.01, ***p < 0.001 compared with LPS treated group.

**FIGURE 14:**
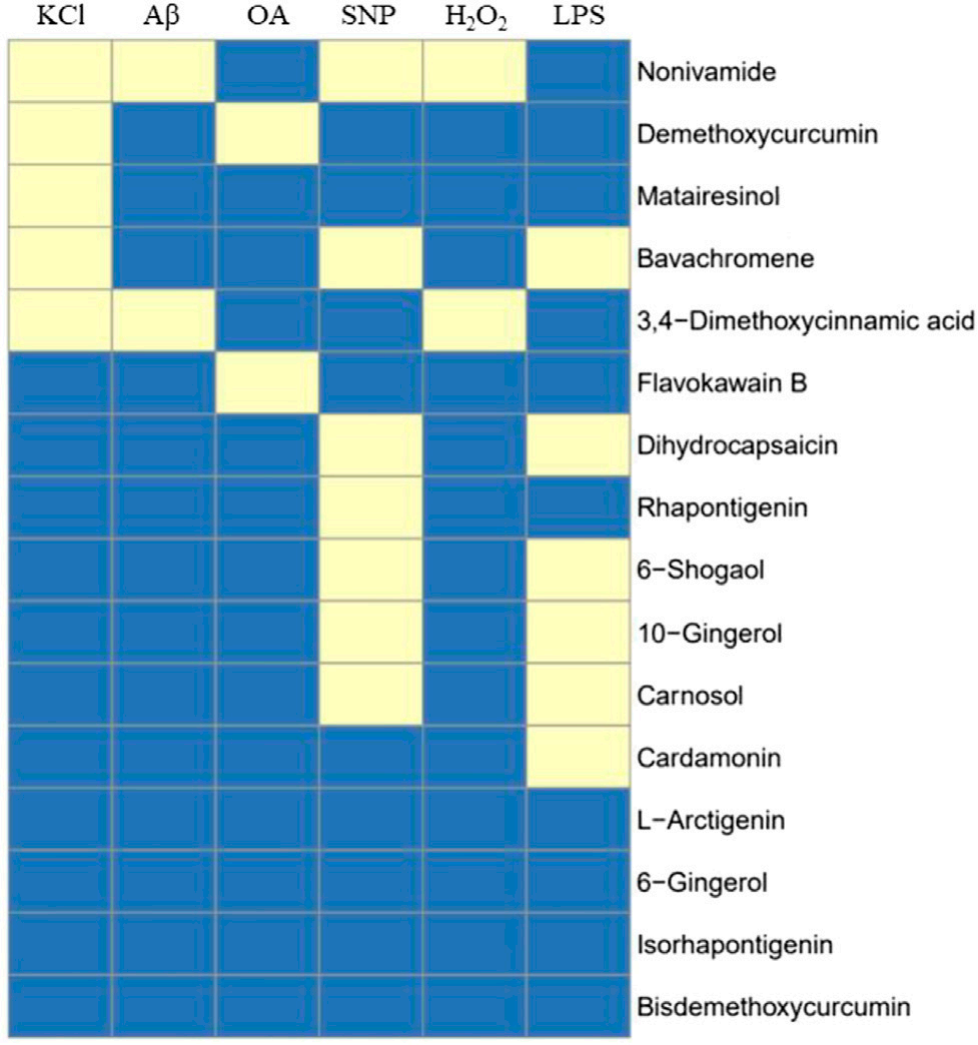
Summary of 16 compound’s activity on 6 cell models. 16 compounds were tested against all cell systems established in this study, including KCl, Aβ, OA, SNP, H_2_O_2_-induced SH-SY5Y cell models and LPS-induced BV2 model, and 12 out of 16 compounds showed activity in one or more cell models, indicating potential anti-AD activity. Yellow means active.

Inflammation plays an important role in the pathogenesis and progression of neurodegenerative diseases. Microglia, as the main immune effector cell in the central nervous system, has the function of regulating inflammation. Activated microglia is the main pathological feature in the brain of neurodegenerative diseases, so the inhibition of neuroinflammation caused by activated microglia play a role in the treatment of neurodegenerative diseases (Annovazzi et al., 2018). Lipopolysaccharide (LPS), the main component of the outer wall layer of Gram-negative bacteria, is a special immune agonist. Administration with LPS *in vitro* or *in vivo* can activate microglia and release a large number of neurotoxic factors, such as NO, reactive oxygen species (ROS), tumor necrosis factor alpha (TNF-α), interleukin1β (IL-1β), IL-6 and excitatory amino acids (Wang et al., 2012). There was no significant change on cell viability of BV2 cells induced by 500 ng/ml LPS for 24 h. Compared with the control group, the cells in the model group released a large amount of NO. Dihydrocapsaicin, 6-Shogaol, 10m-Gingerol, Carnosol, Cardamonin and avachromene had no significant effect on the viability of BV2 cells, but could significantly inhibit the NO release of BV2 cells activated by LPS (Figure 13). The potential anti-AD activities of the 16 compounds are summarized in Figure 14.

### Molecular Docking Suggesting Nonivamide-Target Interaction

**FIGURE 15:**
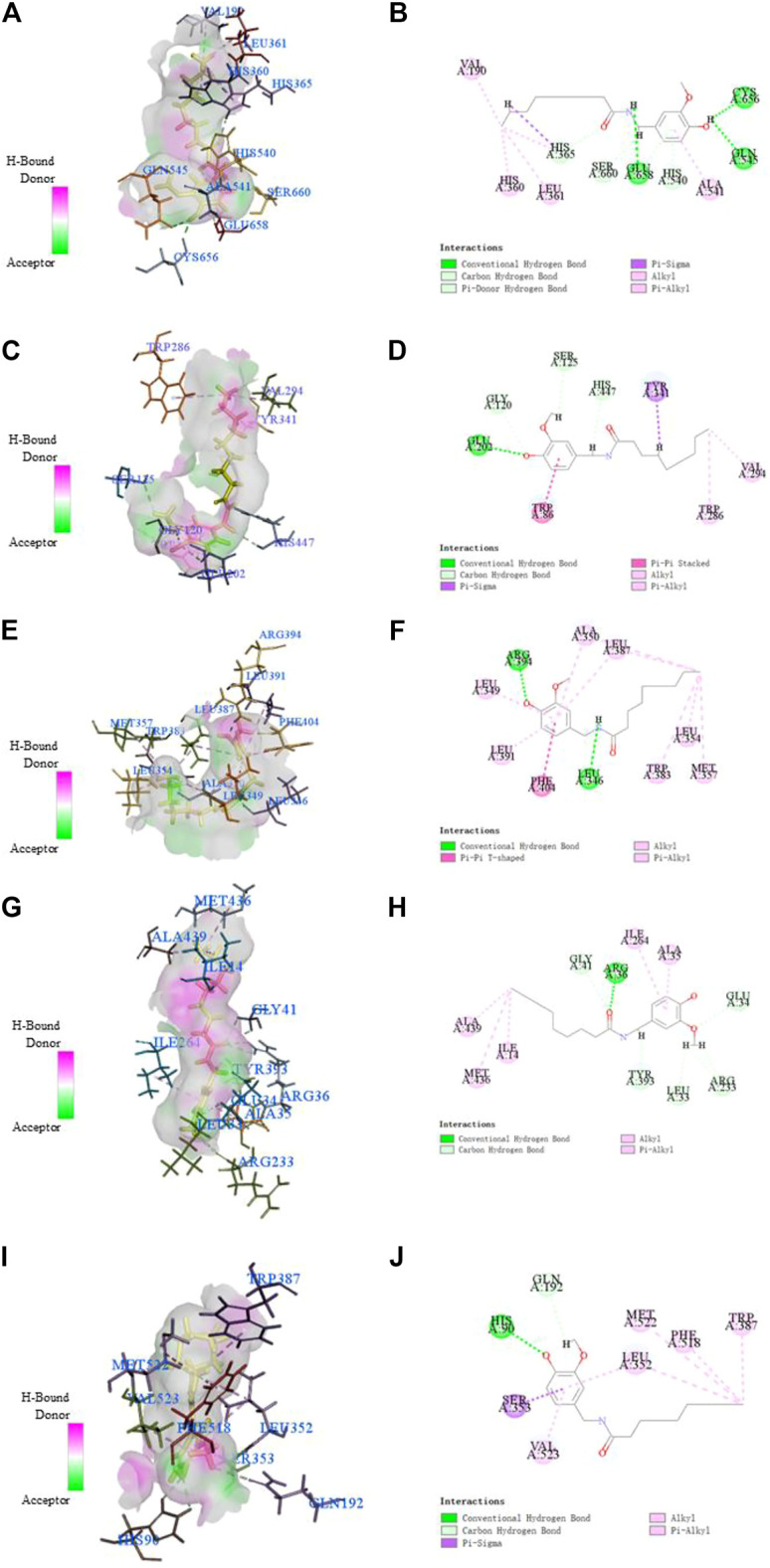
The receptor-ligand interaction of Nonivamide with the active site of ALOX12 **(A, B)**, ACHE **(C, D)**, ESR1 **(E, F)**, MAOB **(G, H)**, PTGS2 **(I, J)**. The ligand was highlighted in yellow in 3D model diagrams.

This study showed that Nonivamide can reduce the damage of SH-SY5Y cells caused by KCl, Aβ, SNP and H_2_O_2_ in a certain concentration range, suggesting that Nonivamide may defeat AD by antagonizing neuro excitotoxicity and oxidative stress. The predicted results indicated that Nonivamide may act on five AD-related targets, including ALOX12, ACHE, ESR1, MAOB, PTGS2. The crystal structures of five target proteins which could be utilized for molecular docking were obtained by searching in PDB database, and then the interaction between Nonivamide and targets was analyzed by establishing molecular docking model. It can be seen from the results in Table 3 that the RMSD values are all less than 1, indicating that the results of molecular docking calculation are accuracy and reliable. As shown in Table 3, the interactions between Nonivamide and these five target proteins were predicted by CDOCKER docking method. The higher the -CDOCKER energy, the stronger the binding ability between the ligand and the target protein. The binding abilities of Nonivamide to ACHE and ESR1 were higher than those of the original ligands in the target proteins and Nonivamide also had good affinity with the other three target proteins. Figure 15 shows that Nonivamide can combine with multiple targets by the ways of hydrogen bond, Pi-bond, ion gravitation and others, which proves the reliability of the prediction results.

**TABLE 3:** The targets for molecular docking and their PDB ID, RMSD value and -CDOCKER energy of Nonivamide and the co-crystallized ligands.

PDB ID	Target	RMSD	-CDOCKER energy of Nonivamide (kcal/mol)	-CDOCKER energy of co-crystallized ligand (kcal/mol)
3D3L	ALOX12	-	46.2326	-
4EY7	ACHE	0.8728	47.2016	22.3205
6PSJ	ESR1	0.789	46.8624	39.6072
1S2Q	MAOB	0.6941	54.5295	106.263
5IKR	PTGS2	0.4059	29.2316	36.6139

## Discussion

The pathogenesis of AD is complex and unclear, but a large number of biological targets with potential therapeutic effects have been identified, such as Aβ, tau protein, cholinergic receptor, glutamate receptor, 5-hydroxytryptamine receptor, dopamine receptor, norepinephrine receptor, acetylcholinesterase, butyrylcholinesterase, α-, β-and γ-acetylcholinesterase, monoamine oxidase A, monoamine oxidase B, etc. (Hampel et al., 2018; Li et al., 2018; Long and Holtzman, 2019). There are many mechanisms considered to be promising for the treatment of AD in the pathological process of AD, of which the most important include excitotoxicity, oxidative stress, neuroinflammation and mitochondrial damage (Murphy and Hartley, 2018). In view of the complexity of AD pathogenesis and the limited understanding, multi-target directional ligands may provide a new way for the discovery of anti-AD drugs. At present, several potential multi-target directional ligands for the treatment of AD have been developed, which provide idea and reference for the research and development of multi-target anti-AD drugs (Xu et al., 2016; Abeijon et al., 2017; Jankowska et al., 2018).

TCM with rich clinical experience and infinite wisdom plays an important role in safeguarding the health of Chinese people (Cheung, 2011). Specifically, TCM provides effective methods for the diagnosis and treatment of chronic and degenerative diseases, including arthritis, diabetes, AD, and so on (Sreenivasmurthy et al., 2017). The purpose of TCM practice is to integrate the combination of herbs under the guidance of a variety of TCM theories according to the specific symptoms of patients. TCM has been widely used in the prevention and treatment of disease for more than 2000 years and there are many Traditional Chinese medicine formulae used to improve cognitive function (Cai et al., 2016). Some chemical components contained in *Radix salviae* (Zhang et al., 2016), *Panacis quinquefolii Radix* (Li et al., 2019), *Zingiberis rhizoma* (Adalier and Parker, 2016), *Radix bupleuri* (Zeng et al., 2019) and *Zingiber officinale Roscoe* (Mohd Sahardi and Makpol, 2019) have been used in the study of AD, which is consistent with the predicted results in this paper. From the prediction results of this paper, most of the chemical components in the anti-AD TCM compounds database could act on the targets in the multi-target anti-AD prediction platform. Sixteen typical chemical components that could act on multiple AD-related pathological processes were selected and several cell models were established to investigate their activity *in vitro*.

It is worth noting that Nonivamide showed good protective activity in four cell models (KCl, Aβ, SNP, H_2_O_2_-induced SH-SY5Y cells injury models). Some studies have shown that Nonivamide exerts anti-inflammatory activity by activating MAPK pathway (Walker et al., 2017) and regulates the process of cellular energy metabolism in a certain concentration range (Hochkogler et al., 2018). Although there are some differences between the activity of Nonivamide in the literature reports and the experimental results in this paper, which needs to be further verified, neuroinflammation and energy metabolism are also two important factors involved in the occurrence and development of AD. Therefore, Nonivamide may be a potential compound of anti-AD treatment and needs to be further studied in the future. Bavachromene and 3,4-Dimethoxycinnamic acid showed good activity in three cell models. At present, there are few reports focused on the activity of Bavachromene, but it showed extraordinary protective effect on SNP-induced SH-SY5Y cells model and its specific protective mechanism needs to be further studied. It was reported that 3,4-Dimethoxycinnamic acid could increase the antioxidant capacity of H9c2 cells, reduce the level of intracellular calcium and effectively prevent cell apoptosis (Sun et al., 2017), which were consistent with the experimental results of this paper. The anti-AD multi-target prediction platform showed that 3,4-Dimethoxycinnamic acid might act on ACHE, APP, PTGS2, GSK3B, which provides a reference for the further study of this chemical constituent. Demethoxycurcumin, Dihydrocapsaicin, 6-Shogaol, 10-Gingerol and Carnosol were effective in two cell models, which provide reference for further research.

In summary, this study predicted the potential targets of chemical constituents from anti-AD traditional Chinese medicine formulae by applying the anti-AD multi-target prediction platform established in our laboratory, from which the representative chemical constituents acting on multiple targets were selected to verify their activity *in vitro*. And the verified activities of potential compounds provide important information for the further study of these chemical constituents. What’s more, the present study also provides reference for the innovative research of other traditional Chinese medicine formulae.

## Data Availability

The raw data supporting the conclusions of this article will be made available by the authors, without undue reservation.
