# Supplementary material for: Identification of Multi-Target Anti-AD Chemical Constituents From Traditional Chinese Medicine Formulae by Integrating Virtual Screening and In Vitro Validation
Source: Front Pharmacol. 2021 Jul 16;12:709607. doi: 10.3389/fphar.2021.709607 (PMC8322649; doi:10.3389/fphar.2021.709607)
Supplement: Supplementary file 2 [file Table1.DOCX]

| Serial number | Chinese name | Chinese pinyin name | Latin name |
| --- | --- | --- | --- |
| 1 | 人参 | Renshen | *Panax Ginseng C. A. Mey.* |
| 2 | 甘草 | Gancao | *licorice* |
| 3 | 远志 | Yuanzhi | *Polygala tenuifolia* |
| 4 | 当归 | Danggui | *Angelicae Sinensis Radix* |
| 5 | 茯苓 | Fuling | *Poria Cocos(Schw.) Wolf.* |
| 6 | 熟地黄 | Shudihuang | *Rehmanniae Radix Praeparata* |
| 7 | 菖蒲 | Changpu | *Rhizoma Acori graminei* |
| 8 | 石菖蒲 | Shichangpu | *Acoritataninowii Rhizoma* |
| 9 | 麦冬 | Maidong | *Ophiopogon japonicus* |
| 10 | 白术 | Baizhu | *Atractylodes Macrocephala Koidz.* |
| 11 | 半夏 | Banxia | *Arum Ternatum Thunb.* |
| 12 | 川芎 | Chuanxiong | *Chuanxiong Rhizoma* |
| 13 | 茯神 | Fushen | *Poria* |
| 14 | 白芍 | Baishao | *Paeoniae Radix Alba* |
| 15 | 生地黄 | Shengdihuang | *Radix Rehmanniae* |
| 16 | 丹参 | Danshen | *Radix Salviae* |
| 17 | 枸杞子 | Gouqizi | *Lycii Fructus* |
| 18 | 黄连 | Huanglian | *Coptidis Rhizoma* |
| 19 | 黄芪 | Huangqi | *Hedysarum Multijugum Maxim.* |
| 20 | 柴胡 | Chaihu | *Radix Bupleuri* |
| 21 | 附子 | Fuzi | *Aconiti Lateralis Radix Praeparata* |
| 22 | 山药 | Shanyao | *Rhizoma Dioscoreae* |
| 23 | 赤芍 | Chishao | *Radix Paeoniae Rubra* |
| 24 | 山茱萸 | Shanzhuyu | *Cornus Officinalis Sieb. Et Zucc.* |
| 25 | 大黄 | Dahuang | *Radix Rhei Et Rhizome* |
| 26 | 何首乌 | Heshouwu | *Polygonum multiflorum* |
| 27 | 牛膝 | Niuxi | *Achyranthis Bidentatae Radix* |
| 28 | 柏子仁 | Baiziren | *Platycladi Semen* |
| 29 | 陈皮 | Chenpi | *Citrus Reticulata* |
| 30 | 葛根 | Gegen | *Radix Puerariae* |
| 31 | 黄芩 | Huangqin | *Scutellariae Radix* |
| 32 | 桔梗 | Jiegeng | *Platycodon Grandiforus* |
| 33 | 南五味子 | Nanwuweizi | *Schisandrae Sphenantherae Fructus* |
| 34 | 大枣 | Dazao | *Jujubae Fructus* |
| 35 | 地龙 | Dilong | *Pheretima* |
| 36 | 生姜 | Shengjiang | *Zingiber Officinale Roscoe* |
| 37 | 益智仁 | Yizhiren | *Alpinia oxyphylla* |
| 38 | 巴戟天 | Bajitian | *Morindae Officinalis Radix* |
| 39 | 白茯苓 | Baifuling | *Poriae Alba* |
| 40 | 肉苁蓉 | Roucongrong | *Cistanches Herba* |
| 41 | 枣仁 | Zaoren | *semen zizphi spinosae* |
| 42 | 酸枣仁 | Suanzaoren | *Ziziphi Spinosae Semen* |
| 43 | 郁金 | Yujin | *Curcumae Radix* |
| 44 | 朱砂 | Zhusha | *Cinnabaris* |
| 45 | 牡蛎 | Muli | *Crassostrea gigas* |
| 46 | 神曲 | Shenqu | *Massa fermentata medicinalis* |
| 47 | 水蛭 | Shuizhi | *Gardenia jasminoides var. grandiflora* |
| 48 | 桃仁 | Taoren | *Persicae Semen* |
| 49 | 菟丝子 | Tusizi | *Cuscutae Semen* |
| 50 | 枳壳 | Zhike | *Aurantii Fructus* |
| 51 | 白芥子 | Baijiezi | *Sinapis alba* |
| 52 | 党参 | Dangshen | *Codonopsis Radix* |
| 53 | 干姜 | Ganjiang | *Zingiberis Rhizoma* |
| 54 | 桂枝 | Guizhi | *Cinnamomi Ramulus* |
| 55 | 龙骨 | Longgu | *Os Draconis* |
| 56 | 牡丹皮 | Mudanpi | *Cortex Moutan* |
| 57 | 天南星 | Tiannanxing | *Arisaematis Rhizoma* |
| 58 | 肉桂 | Rougui | *Cinnanmomi Cortex* |
| 59 | 天麻 | Tianma | *Gastrodia elata* |
| 60 | 泽泻 | Zexie | *Alisma Orientale (Sam.) Juz.* |
| 61 | 栀子 | Zhizi | *Gardeniae Fructus* |
| 62 | 枳实 | Zhishi | *Aurantii Fructus Immaturus* |
| 63 | 冰片 | Bingpian | *Borneolum Syntheticum* |
| 64 | 胆星 | Danxing | *Rhizoma Arisaema cum Bile* |
| 65 | 地骨皮 | Digupi | *Lycii Cortex* |
| 66 | 杜仲 | Duzhong | *Eucommiae Cortex* |
| 67 | 木通 | Mutong | *Caulis Akebiae* |
| 68 | 青皮 | Qingpi | *Citri Reticulatae Pericarpium Viride* |
| 69 | 何首乌 | Heshouwu | *Polygonum multiflorum* |
| 70 | 天冬 | Tiandong | *Asparagi Radix* |
| 71 | 知母 | Zhimu | *Anemarrhenae Rhizoma* |
| 72 | 紫河车 | Ziheche | *Homo sapiens* |
| 73 | 白芷 | Baizhi | *A. Dahurica (Fisch.) Benth. Et Hook* |
| 74 | 薄荷 | Bohe | *Menthae Herba* |
| 75 | 北五味子 | Beiwuweizi | *Schisandrae Chinensis Fructus* |
| 76 | 车前子 | Cheqianzi | *Plantaginis Semen* |
| 77 | 沉香 | Chenxiang | *Linderae Radix* |
| 78 | 防风 | Fangfeng | *Saposhnikoviae Radix* |
| 79 | 红花 | Honghua | *Carthami Flos* |
| 80 | 黄柏 | Huangbo | *Phellodendri Chinrnsis Cortex* |
| 81 | 连翘 | Lianqiao | *Forsythiae Fructus* |
| 82 | 羚羊角 | Lingyangjiao | *Cornu Saigae Tataricae* |
| 83 | 麝香 | Shexiang | *Moschus moschiferus, Moschus berezovskii, Moschus* |
| 84 | 石斛 | Shihu | *Herba Dendrobii* |
| 85 | 牛黄 | Niuhuang | *Bovis Calculus* |
| 86 | 香附 | Xiangfu | *Cyperi Rhizoma* |
| 87 | 玄参 | Xuanshen | *Figwort Root* |
| 88 | 竹沥 | Zhuli | *Bomboo Juice* |
| 89 | 独活 | Duhuo | *Radix Angelicae Biseratae* |
| 90 | 川乌 | Chuanwu | *Aconiti Radix* |
| 91 | 百合 | Baihe | *Lilii Bulbus* |
| 92 | 骨碎补 | Gusuibu | *Drynariae Rhizoma* |
| 93 | 补骨脂 | Buguzhi | *Psoralea corylifolia* |
| 94 | 牡丹皮 | Mudanpi | *Cortex Moutan* |
| 95 | 银杏叶 | Yinxingye | *Ginkgo Folium* |
| 96 | 前胡 | Qianhu | *Peucedani Radix* |
| 97 | 牛蒡子 | Niubangzi | *Fructus Arctii* |
| 98 | 山楂 | Shanzha | *Crataegus pinnatifida* |
| 99 | 吴茱萸 | Wuzhuyu | *Evodiae Fructus* |
| 100 | 莲子心 | Lianzixin | *Nelumbinis Plumula* |
| 101 | 西洋参 | Xiyangshen | *Panacis Quinquefolii Radix* |
| 102 | 苦杏仁 | Kuxingren | *Amygdalus Communis Vas* |
