# Supplementary material for: Identification of Multi-Target Anti-AD Chemical Constituents From Traditional Chinese Medicine Formulae by Integrating Virtual Screening and In Vitro Validation
Source: Front Pharmacol. 2021 Jul 16;12:709607. doi: 10.3389/fphar.2021.709607 (PMC8322649; doi:10.3389/fphar.2021.709607)
Supplement: Supplementary file 3 [file DataSheet1.ZIP › Good and bad fragments of 52 targets/ACAT1.html]

Category NB\_ACCT\_ECFP6: good features from ECFP\_6

|  |  |  |  |  |  |  |  |  |  |  |  |  |  |  |
| --- | --- | --- | --- | --- | --- | --- | --- | --- | --- | --- | --- | --- | --- | --- |
| |  | | --- | |  | | G1: 2008202481  90 out of 90 good  Bayesian Score: 1.170 | | |  | | --- | |  | | G2: 694054216  89 out of 89 good  Bayesian Score: 1.170 | | |  | | --- | |  | | G3: 695384874  89 out of 89 good  Bayesian Score: 1.170 | | |  | | --- | |  | | G4: -4367276  89 out of 89 good  Bayesian Score: 1.170 | | |  | | --- | |  | | G5: 1879463989  89 out of 89 good  Bayesian Score: 1.170 | |
| |  | | --- | |  | | G6: -500963800  89 out of 89 good  Bayesian Score: 1.170 | | |  | | --- | |  | | G7: -667365306  89 out of 89 good  Bayesian Score: 1.170 | | |  | | --- | |  | | G8: 366569504  89 out of 89 good  Bayesian Score: 1.170 | | |  | | --- | |  | | G9: -1470733165  89 out of 89 good  Bayesian Score: 1.170 | | |  | | --- | |  | | G10: 337387206  89 out of 89 good  Bayesian Score: 1.170 | |
| |  | | --- | |  | | G11: -213494185  89 out of 89 good  Bayesian Score: 1.170 | | |  | | --- | |  | | G12: -1153158142  89 out of 89 good  Bayesian Score: 1.170 | | |  | | --- | |  | | G13: 1910526603  89 out of 89 good  Bayesian Score: 1.170 | | |  | | --- | |  | | G14: 1689113257  89 out of 89 good  Bayesian Score: 1.170 | | |  | | --- | |  | | G15: 830475149  89 out of 89 good  Bayesian Score: 1.170 | |
| |  | | --- | |  | | G16: 1446407874  89 out of 89 good  Bayesian Score: 1.170 | | |  | | --- | |  | | G17: 2052971613  89 out of 89 good  Bayesian Score: 1.170 | | |  | | --- | |  | | G18: -1139064737  89 out of 89 good  Bayesian Score: 1.170 | | |  | | --- | |  | | G19: 1531401036  89 out of 89 good  Bayesian Score: 1.170 | | |  | | --- | |  | | G20: -1328953038  89 out of 89 good  Bayesian Score: 1.170 | |

Category NB\_ACCT\_ECFP6: bad features from ECFP\_6

|  |  |  |  |  |  |  |  |  |  |  |  |  |  |  |
| --- | --- | --- | --- | --- | --- | --- | --- | --- | --- | --- | --- | --- | --- | --- |
| |  | | --- | |  | | B1: -1790802833  0 out of 83 good  Bayesian Score: -3.263 | | |  | | --- | |  | | B2: 1035165602  0 out of 59 good  Bayesian Score: -2.937 | | |  | | --- | |  | | B3: -949992060  0 out of 56 good  Bayesian Score: -2.888 | | |  | | --- | |  | | B4: -655344035  0 out of 51 good  Bayesian Score: -2.800 | | |  | | --- | |  | | B5: -1255706725  2 out of 158 good  Bayesian Score: -2.790 | |
| |  | | --- | |  | | B6: 1886768955  0 out of 48 good  Bayesian Score: -2.743 | | |  | | --- | |  | | B7: -1332781180  3 out of 189 good  Bayesian Score: -2.678 | | |  | | --- | |  | | B8: 912478223  0 out of 44 good  Bayesian Score: -2.662 | | |  | | --- | |  | | B9: -949601813  1 out of 87 good  Bayesian Score: -2.615 | | |  | | --- | |  | | B10: 103339584  0 out of 41 good  Bayesian Score: -2.596 | |
| |  | | --- | |  | | B11: 1731843802  0 out of 39 good  Bayesian Score: -2.550 | | |  | | --- | |  | | B12: -845108448  0 out of 37 good  Bayesian Score: -2.501 | | |  | | --- | |  | | B13: -1832102709  0 out of 36 good  Bayesian Score: -2.476 | | |  | | --- | |  | | B14: 1706085096  0 out of 36 good  Bayesian Score: -2.476 | | |  | | --- | |  | | B15: -661766797  0 out of 36 good  Bayesian Score: -2.476 | |
| |  | | --- | |  | | B16: 888054369  0 out of 34 good  Bayesian Score: -2.424 | | |  | | --- | |  | | B17: -1688513024  0 out of 34 good  Bayesian Score: -2.424 | | |  | | --- | |  | | B18: 1334973442  0 out of 34 good  Bayesian Score: -2.424 | | |  | | --- | |  | | B19: -1087070950  1 out of 70 good  Bayesian Score: -2.406 | | |  | | --- | |  | | B20: 865482986  1 out of 70 good  Bayesian Score: -2.406 | |
