# Supplementary material for: Identification of Multi-Target Anti-AD Chemical Constituents From Traditional Chinese Medicine Formulae by Integrating Virtual Screening and In Vitro Validation
Source: Front Pharmacol. 2021 Jul 16;12:709607. doi: 10.3389/fphar.2021.709607 (PMC8322649; doi:10.3389/fphar.2021.709607)
Supplement: Supplementary file 3 [file DataSheet1.ZIP › Good and bad fragments of 52 targets/ACHE.html]

Category Bayesian-AChE: good features from ECFP\_6

|  |  |  |  |  |  |  |  |  |  |  |  |  |  |  |
| --- | --- | --- | --- | --- | --- | --- | --- | --- | --- | --- | --- | --- | --- | --- |
| |  | | --- | |  | | G1: -1056448394  373 out of 373 good  Bayesian Score: 1.361 | | |  | | --- | |  | | G2: -128679049  351 out of 351 good  Bayesian Score: 1.361 | | |  | | --- | |  | | G3: 624344093  341 out of 341 good  Bayesian Score: 1.360 | | |  | | --- | |  | | G4: -1441803809  325 out of 325 good  Bayesian Score: 1.360 | | |  | | --- | |  | | G5: -1686479412  287 out of 287 good  Bayesian Score: 1.359 | |
| |  | | --- | |  | | G6: 485677615  287 out of 287 good  Bayesian Score: 1.359 | | |  | | --- | |  | | G7: -1108879003  278 out of 278 good  Bayesian Score: 1.358 | | |  | | --- | |  | | G8: -1830436798  270 out of 270 good  Bayesian Score: 1.358 | | |  | | --- | |  | | G9: 1973629459  268 out of 268 good  Bayesian Score: 1.358 | | |  | | --- | |  | | G10: 440744344  359 out of 360 good  Bayesian Score: 1.358 | |
| |  | | --- | |  | | G11: -354782378  266 out of 266 good  Bayesian Score: 1.358 | | |  | | --- | |  | | G12: -1731900812  263 out of 263 good  Bayesian Score: 1.358 | | |  | | --- | |  | | G13: 1334101934  244 out of 244 good  Bayesian Score: 1.357 | | |  | | --- | |  | | G14: 805978089  242 out of 242 good  Bayesian Score: 1.357 | | |  | | --- | |  | | G15: 552399862  233 out of 233 good  Bayesian Score: 1.356 | |
| |  | | --- | |  | | G16: 2123449152  295 out of 296 good  Bayesian Score: 1.356 | | |  | | --- | |  | | G17: -390533593  219 out of 219 good  Bayesian Score: 1.356 | | |  | | --- | |  | | G18: -1811250099  292 out of 293 good  Bayesian Score: 1.356 | | |  | | --- | |  | | G19: 1508318431  213 out of 213 good  Bayesian Score: 1.355 | | |  | | --- | |  | | G20: -514501799  427 out of 430 good  Bayesian Score: 1.355 | |

Category Bayesian-AChE: bad features from ECFP\_6

|  |  |  |  |  |  |  |  |  |  |  |  |  |  |  |
| --- | --- | --- | --- | --- | --- | --- | --- | --- | --- | --- | --- | --- | --- | --- |
| |  | | --- | |  | | B1: 908605940  0 out of 850 good  Bayesian Score: -5.381 | | |  | | --- | |  | | B2: 651217135  0 out of 561 good  Bayesian Score: -4.968 | | |  | | --- | |  | | B3: -936852899  0 out of 549 good  Bayesian Score: -4.946 | | |  | | --- | |  | | B4: -756348342  0 out of 379 good  Bayesian Score: -4.579 | | |  | | --- | |  | | B5: 33329059  0 out of 354 good  Bayesian Score: -4.511 | |
| |  | | --- | |  | | B6: -2038514967  0 out of 339 good  Bayesian Score: -4.469 | | |  | | --- | |  | | B7: -2041399277  0 out of 288 good  Bayesian Score: -4.308 | | |  | | --- | |  | | B8: -409182516  1 out of 570 good  Bayesian Score: -4.290 | | |  | | --- | |  | | B9: -1241623705  0 out of 270 good  Bayesian Score: -4.244 | | |  | | --- | |  | | B10: -15368233  0 out of 261 good  Bayesian Score: -4.211 | |
| |  | | --- | |  | | B11: 1796154575  1 out of 523 good  Bayesian Score: -4.205 | | |  | | --- | |  | | B12: 1158715317  0 out of 253 good  Bayesian Score: -4.180 | | |  | | --- | |  | | B13: -1619703999  0 out of 248 good  Bayesian Score: -4.160 | | |  | | --- | |  | | B14: 1014815980  0 out of 242 good  Bayesian Score: -4.136 | | |  | | --- | |  | | B15: -676555381  0 out of 222 good  Bayesian Score: -4.051 | |
| |  | | --- | |  | | B16: -495825537  0 out of 218 good  Bayesian Score: -4.033 | | |  | | --- | |  | | B17: 2082478181  0 out of 212 good  Bayesian Score: -4.006 | | |  | | --- | |  | | B18: 300955665  2 out of 643 good  Bayesian Score: -4.005 | | |  | | --- | |  | | B19: -274598411  0 out of 176 good  Bayesian Score: -3.824 | | |  | | --- | |  | | B20: -393638546  0 out of 170 good  Bayesian Score: -3.790 | |
