# Supplementary material for: Identification of Multi-Target Anti-AD Chemical Constituents From Traditional Chinese Medicine Formulae by Integrating Virtual Screening and In Vitro Validation
Source: Front Pharmacol. 2021 Jul 16;12:709607. doi: 10.3389/fphar.2021.709607 (PMC8322649; doi:10.3389/fphar.2021.709607)
Supplement: Supplementary file 3 [file DataSheet1.ZIP › Good and bad fragments of 52 targets/ADORA2A.html]

Category NB\_aa2ar-ECFP6: good features from ECFP\_6

|  |  |  |  |  |  |  |  |  |  |  |  |  |  |  |
| --- | --- | --- | --- | --- | --- | --- | --- | --- | --- | --- | --- | --- | --- | --- |
| |  | | --- | |  | | G1: -355708035  366 out of 366 good  Bayesian Score: 1.421 | | |  | | --- | |  | | G2: 615197788  461 out of 462 good  Bayesian Score: 1.421 | | |  | | --- | |  | | G3: -1915966606  326 out of 326 good  Bayesian Score: 1.420 | | |  | | --- | |  | | G4: 217896370  308 out of 308 good  Bayesian Score: 1.420 | | |  | | --- | |  | | G5: 21275359  280 out of 280 good  Bayesian Score: 1.419 | |
| |  | | --- | |  | | G6: 1323139714  278 out of 278 good  Bayesian Score: 1.419 | | |  | | --- | |  | | G7: 1946075712  450 out of 452 good  Bayesian Score: 1.418 | | |  | | --- | |  | | G8: 125442029  268 out of 268 good  Bayesian Score: 1.418 | | |  | | --- | |  | | G9: 817760163  267 out of 267 good  Bayesian Score: 1.418 | | |  | | --- | |  | | G10: -1296332732  267 out of 267 good  Bayesian Score: 1.418 | |
| |  | | --- | |  | | G11: 2132359943  260 out of 260 good  Bayesian Score: 1.418 | | |  | | --- | |  | | G12: 1812016488  260 out of 260 good  Bayesian Score: 1.418 | | |  | | --- | |  | | G13: -444179340  247 out of 247 good  Bayesian Score: 1.417 | | |  | | --- | |  | | G14: -1429393516  243 out of 243 good  Bayesian Score: 1.417 | | |  | | --- | |  | | G15: -565701062  394 out of 396 good  Bayesian Score: 1.417 | |
| |  | | --- | |  | | G16: -1219098860  394 out of 396 good  Bayesian Score: 1.417 | | |  | | --- | |  | | G17: -1157241211  240 out of 240 good  Bayesian Score: 1.417 | | |  | | --- | |  | | G18: 2069176821  305 out of 306 good  Bayesian Score: 1.416 | | |  | | --- | |  | | G19: 1236379268  229 out of 229 good  Bayesian Score: 1.416 | | |  | | --- | |  | | G20: 930751877  217 out of 217 good  Bayesian Score: 1.415 | |

Category NB\_aa2ar-ECFP6: bad features from ECFP\_6

|  |  |  |  |  |  |  |  |  |  |  |  |  |  |  |
| --- | --- | --- | --- | --- | --- | --- | --- | --- | --- | --- | --- | --- | --- | --- |
| |  | | --- | |  | | B1: 1976330679  0 out of 1309 good  Bayesian Score: -5.750 | | |  | | --- | |  | | B2: -244159614  0 out of 1190 good  Bayesian Score: -5.655 | | |  | | --- | |  | | B3: 908605940  0 out of 674 good  Bayesian Score: -5.090 | | |  | | --- | |  | | B4: 1133499173  0 out of 546 good  Bayesian Score: -4.880 | | |  | | --- | |  | | B5: 233520344  0 out of 528 good  Bayesian Score: -4.847 | |
| |  | | --- | |  | | B6: 1796154575  0 out of 476 good  Bayesian Score: -4.744 | | |  | | --- | |  | | B7: 1961554343  1 out of 918 good  Bayesian Score: -4.704 | | |  | | --- | |  | | B8: 651217135  0 out of 424 good  Bayesian Score: -4.630 | | |  | | --- | |  | | B9: -936852899  0 out of 418 good  Bayesian Score: -4.616 | | |  | | --- | |  | | B10: -1989458582  0 out of 372 good  Bayesian Score: -4.500 | |
| |  | | --- | |  | | B11: 590183026  0 out of 372 good  Bayesian Score: -4.500 | | |  | | --- | |  | | B12: -762397514  0 out of 344 good  Bayesian Score: -4.423 | | |  | | --- | |  | | B13: -1048916822  0 out of 337 good  Bayesian Score: -4.403 | | |  | | --- | |  | | B14: -1672647522  0 out of 336 good  Bayesian Score: -4.400 | | |  | | --- | |  | | B15: 1433937652  0 out of 330 good  Bayesian Score: -4.382 | |
| |  | | --- | |  | | B16: 1678190906  0 out of 328 good  Bayesian Score: -4.376 | | |  | | --- | |  | | B17: 1383031266  0 out of 327 good  Bayesian Score: -4.373 | | |  | | --- | |  | | B18: 685356098  0 out of 327 good  Bayesian Score: -4.373 | | |  | | --- | |  | | B19: -200820495  0 out of 327 good  Bayesian Score: -4.373 | | |  | | --- | |  | | B20: -59864335  0 out of 327 good  Bayesian Score: -4.373 | |
