# Supplementary material for: Identification of Multi-Target Anti-AD Chemical Constituents From Traditional Chinese Medicine Formulae by Integrating Virtual Screening and In Vitro Validation
Source: Front Pharmacol. 2021 Jul 16;12:709607. doi: 10.3389/fphar.2021.709607 (PMC8322649; doi:10.3389/fphar.2021.709607)
Supplement: Supplementary file 3 [file DataSheet1.ZIP › Good and bad fragments of 52 targets/ALOX12.html]

Category NB\_12-lipoxygenase-ECFP6: good features from ECFP\_6

|  |  |  |  |  |  |  |  |  |  |  |  |  |  |  |
| --- | --- | --- | --- | --- | --- | --- | --- | --- | --- | --- | --- | --- | --- | --- |
| |  | | --- | |  | | G1: -1194983391  44 out of 44 good  Bayesian Score: 1.275 | | |  | | --- | |  | | G2: 1337803373  44 out of 44 good  Bayesian Score: 1.275 | | |  | | --- | |  | | G3: -1238602038  44 out of 44 good  Bayesian Score: 1.275 | | |  | | --- | |  | | G4: 1116322394  44 out of 44 good  Bayesian Score: 1.275 | | |  | | --- | |  | | G5: -857683902  44 out of 44 good  Bayesian Score: 1.275 | |
| |  | | --- | |  | | G6: -1914389527  44 out of 44 good  Bayesian Score: 1.275 | | |  | | --- | |  | | G7: 1375591260  44 out of 44 good  Bayesian Score: 1.275 | | |  | | --- | |  | | G8: -124856687  44 out of 44 good  Bayesian Score: 1.275 | | |  | | --- | |  | | G9: 1157174664  36 out of 36 good  Bayesian Score: 1.263 | | |  | | --- | |  | | G10: -350804603  36 out of 36 good  Bayesian Score: 1.263 | |
| |  | | --- | |  | | G11: 1933422916  36 out of 36 good  Bayesian Score: 1.263 | | |  | | --- | |  | | G12: -1660913849  62 out of 64 good  Bayesian Score: 1.262 | | |  | | --- | |  | | G13: -1625257771  44 out of 45 good  Bayesian Score: 1.255 | | |  | | --- | |  | | G14: 74112679  44 out of 45 good  Bayesian Score: 1.255 | | |  | | --- | |  | | G15: -292555972  44 out of 45 good  Bayesian Score: 1.255 | |
| |  | | --- | |  | | G16: 1566992507  44 out of 45 good  Bayesian Score: 1.255 | | |  | | --- | |  | | G17: -1693360184  27 out of 27 good  Bayesian Score: 1.240 | | |  | | --- | |  | | G18: 203447354  27 out of 27 good  Bayesian Score: 1.240 | | |  | | --- | |  | | G19: -655067379  27 out of 27 good  Bayesian Score: 1.240 | | |  | | --- | |  | | G20: -816659875  27 out of 27 good  Bayesian Score: 1.240 | |

Category NB\_12-lipoxygenase-ECFP6: bad features from ECFP\_6

|  |  |  |  |  |  |  |  |  |  |  |  |  |  |  |
| --- | --- | --- | --- | --- | --- | --- | --- | --- | --- | --- | --- | --- | --- | --- |
| |  | | --- | |  | | B1: -1236483485  0 out of 48 good  Bayesian Score: -2.612 | | |  | | --- | |  | | B2: 672362763  0 out of 41 good  Bayesian Score: -2.467 | | |  | | --- | |  | | B3: -661766797  0 out of 36 good  Bayesian Score: -2.348 | | |  | | --- | |  | | B4: -938530932  0 out of 35 good  Bayesian Score: -2.323 | | |  | | --- | |  | | B5: 663943468  0 out of 34 good  Bayesian Score: -2.297 | |
| |  | | --- | |  | | B6: 781519895  1 out of 71 good  Bayesian Score: -2.286 | | |  | | --- | |  | | B7: -1699286547  0 out of 30 good  Bayesian Score: -2.185 | | |  | | --- | |  | | B8: 865482986  0 out of 29 good  Bayesian Score: -2.155 | | |  | | --- | |  | | B9: 51876938  0 out of 28 good  Bayesian Score: -2.124 | | |  | | --- | |  | | B10: 1572579716  1 out of 59 good  Bayesian Score: -2.111 | |
| |  | | --- | |  | | B11: -801490360  0 out of 27 good  Bayesian Score: -2.092 | | |  | | --- | |  | | B12: -655344035  0 out of 27 good  Bayesian Score: -2.092 | | |  | | --- | |  | | B13: 1430169877  0 out of 27 good  Bayesian Score: -2.092 | | |  | | --- | |  | | B14: 662850656  0 out of 26 good  Bayesian Score: -2.059 | | |  | | --- | |  | | B15: 2085698692  0 out of 23 good  Bayesian Score: -1.953 | |
| |  | | --- | |  | | B16: -1087070950  1 out of 49 good  Bayesian Score: -1.938 | | |  | | --- | |  | | B17: 413587124  0 out of 22 good  Bayesian Score: -1.915 | | |  | | --- | |  | | B18: -845108448  0 out of 22 good  Bayesian Score: -1.915 | | |  | | --- | |  | | B19: -264471301  0 out of 22 good  Bayesian Score: -1.915 | | |  | | --- | |  | | B20: 911256832  0 out of 20 good  Bayesian Score: -1.834 | |
