# Supplementary material for: Identification of Multi-Target Anti-AD Chemical Constituents From Traditional Chinese Medicine Formulae by Integrating Virtual Screening and In Vitro Validation
Source: Front Pharmacol. 2021 Jul 16;12:709607. doi: 10.3389/fphar.2021.709607 (PMC8322649; doi:10.3389/fphar.2021.709607)
Supplement: Supplementary file 3 [file DataSheet1.ZIP › Good and bad fragments of 52 targets/APP.html]

Category Bayesian-AMPA1: good features from ECFP\_6

|  |  |  |  |  |  |  |  |  |  |  |  |  |  |  |
| --- | --- | --- | --- | --- | --- | --- | --- | --- | --- | --- | --- | --- | --- | --- |
| |  | | --- | |  | | G1: 709212425  43 out of 43 good  Bayesian Score: 1.175 | | |  | | --- | |  | | G2: 1063370408  43 out of 43 good  Bayesian Score: 1.175 | | |  | | --- | |  | | G3: -1526166162  40 out of 40 good  Bayesian Score: 1.171 | | |  | | --- | |  | | G4: -2049718428  38 out of 38 good  Bayesian Score: 1.169 | | |  | | --- | |  | | G5: 951853694  38 out of 38 good  Bayesian Score: 1.169 | |
| |  | | --- | |  | | G6: 715183730  37 out of 37 good  Bayesian Score: 1.167 | | |  | | --- | |  | | G7: -1299610481  37 out of 37 good  Bayesian Score: 1.167 | | |  | | --- | |  | | G8: 1940437808  35 out of 35 good  Bayesian Score: 1.164 | | |  | | --- | |  | | G9: -1330434669  47 out of 48 good  Bayesian Score: 1.160 | | |  | | --- | |  | | G10: -711653271  43 out of 44 good  Bayesian Score: 1.154 | |
| |  | | --- | |  | | G11: -2117608450  43 out of 44 good  Bayesian Score: 1.154 | | |  | | --- | |  | | G12: 2145219777  41 out of 42 good  Bayesian Score: 1.150 | | |  | | --- | |  | | G13: -561390568  28 out of 28 good  Bayesian Score: 1.149 | | |  | | --- | |  | | G14: 382805658  28 out of 28 good  Bayesian Score: 1.149 | | |  | | --- | |  | | G15: 1638421040  38 out of 39 good  Bayesian Score: 1.145 | |
| |  | | --- | |  | | G16: -790637051  38 out of 39 good  Bayesian Score: 1.145 | | |  | | --- | |  | | G17: 770157610  47 out of 49 good  Bayesian Score: 1.141 | | |  | | --- | |  | | G18: 510186613  43 out of 45 good  Bayesian Score: 1.133 | | |  | | --- | |  | | G19: -1690551527  32 out of 33 good  Bayesian Score: 1.130 | | |  | | --- | |  | | G20: -2123445564  41 out of 43 good  Bayesian Score: 1.129 | |

Category Bayesian-AMPA1: bad features from ECFP\_6

|  |  |  |  |  |  |  |  |  |  |  |  |  |  |  |
| --- | --- | --- | --- | --- | --- | --- | --- | --- | --- | --- | --- | --- | --- | --- |
| |  | | --- | |  | | B1: 834876373  0 out of 46 good  Bayesian Score: -2.672 | | |  | | --- | |  | | B2: -1087070950  0 out of 40 good  Bayesian Score: -2.542 | | |  | | --- | |  | | B3: -1236483485  0 out of 31 good  Bayesian Score: -2.310 | | |  | | --- | |  | | B4: -1072294614  0 out of 30 good  Bayesian Score: -2.280 | | |  | | --- | |  | | B5: -830332112  0 out of 27 good  Bayesian Score: -2.186 | |
| |  | | --- | |  | | B6: -938530932  0 out of 25 good  Bayesian Score: -2.118 | | |  | | --- | |  | | B7: -845108448  0 out of 25 good  Bayesian Score: -2.118 | | |  | | --- | |  | | B8: 2022454958  0 out of 25 good  Bayesian Score: -2.118 | | |  | | --- | |  | | B9: -757679000  0 out of 22 good  Bayesian Score: -2.007 | | |  | | --- | |  | | B10: -177077903  0 out of 21 good  Bayesian Score: -1.967 | |
| |  | | --- | |  | | B11: -1255706725  0 out of 20 good  Bayesian Score: -1.925 | | |  | | --- | |  | | B12: -1790412586  0 out of 19 good  Bayesian Score: -1.881 | | |  | | --- | |  | | B13: -2067954201  0 out of 18 good  Bayesian Score: -1.836 | | |  | | --- | |  | | B14: 2024749573  0 out of 17 good  Bayesian Score: -1.788 | | |  | | --- | |  | | B15: 412256466  0 out of 17 good  Bayesian Score: -1.788 | |
| |  | | --- | |  | | B16: 1979182050  0 out of 17 good  Bayesian Score: -1.788 | | |  | | --- | |  | | B17: 888054369  0 out of 17 good  Bayesian Score: -1.788 | | |  | | --- | |  | | B18: -949601813  0 out of 17 good  Bayesian Score: -1.788 | | |  | | --- | |  | | B19: -91640731  0 out of 17 good  Bayesian Score: -1.788 | | |  | | --- | |  | | B20: -1059365320  4 out of 94 good  Bayesian Score: -1.741 | |
