# Supplementary material for: Identification of Multi-Target Anti-AD Chemical Constituents From Traditional Chinese Medicine Formulae by Integrating Virtual Screening and In Vitro Validation
Source: Front Pharmacol. 2021 Jul 16;12:709607. doi: 10.3389/fphar.2021.709607 (PMC8322649; doi:10.3389/fphar.2021.709607)
Supplement: Supplementary file 3 [file DataSheet1.ZIP › Good and bad fragments of 52 targets/BACE1.html]

Category Bayesian-BACE1: good features from ECFP\_6

|  |  |  |  |  |  |  |  |  |  |  |  |  |  |  |
| --- | --- | --- | --- | --- | --- | --- | --- | --- | --- | --- | --- | --- | --- | --- |
| |  | | --- | |  | | G1: 3228927  666 out of 666 good  Bayesian Score: 1.295 | | |  | | --- | |  | | G2: 1334840514  818 out of 819 good  Bayesian Score: 1.294 | | |  | | --- | |  | | G3: 766511464  494 out of 494 good  Bayesian Score: 1.293 | | |  | | --- | |  | | G4: -1921294579  433 out of 433 good  Bayesian Score: 1.293 | | |  | | --- | |  | | G5: -1843371804  365 out of 365 good  Bayesian Score: 1.291 | |
| |  | | --- | |  | | G6: -1798867401  325 out of 325 good  Bayesian Score: 1.291 | | |  | | --- | |  | | G7: -1342559370  317 out of 317 good  Bayesian Score: 1.290 | | |  | | --- | |  | | G8: 2119038210  315 out of 315 good  Bayesian Score: 1.290 | | |  | | --- | |  | | G9: 636907239  315 out of 315 good  Bayesian Score: 1.290 | | |  | | --- | |  | | G10: 1900115826  311 out of 311 good  Bayesian Score: 1.290 | |
| |  | | --- | |  | | G11: 1821159565  300 out of 300 good  Bayesian Score: 1.290 | | |  | | --- | |  | | G12: 451953143  278 out of 278 good  Bayesian Score: 1.289 | | |  | | --- | |  | | G13: 571758812  231 out of 231 good  Bayesian Score: 1.287 | | |  | | --- | |  | | G14: 78733088  638 out of 643 good  Bayesian Score: 1.287 | | |  | | --- | |  | | G15: 767790018  220 out of 220 good  Bayesian Score: 1.287 | |
| |  | | --- | |  | | G16: 2080306479  216 out of 216 good  Bayesian Score: 1.286 | | |  | | --- | |  | | G17: 191914930  210 out of 210 good  Bayesian Score: 1.286 | | |  | | --- | |  | | G18: -196183339  188 out of 188 good  Bayesian Score: 1.285 | | |  | | --- | |  | | G19: 2010574440  187 out of 187 good  Bayesian Score: 1.285 | | |  | | --- | |  | | G20: -773754971  325 out of 327 good  Bayesian Score: 1.284 | |

Category Bayesian-BACE1: bad features from ECFP\_6

|  |  |  |  |  |  |  |  |  |  |  |  |  |  |  |
| --- | --- | --- | --- | --- | --- | --- | --- | --- | --- | --- | --- | --- | --- | --- |
| |  | | --- | |  | | B1: -1672647522  0 out of 320 good  Bayesian Score: -4.481 | | |  | | --- | |  | | B2: 908605940  0 out of 305 good  Bayesian Score: -4.434 | | |  | | --- | |  | | B3: 1796154575  0 out of 281 good  Bayesian Score: -4.353 | | |  | | --- | |  | | B4: 1979182050  0 out of 277 good  Bayesian Score: -4.338 | | |  | | --- | |  | | B5: 1986731747  0 out of 259 good  Bayesian Score: -4.272 | |
| |  | | --- | |  | | B6: 300955665  0 out of 244 good  Bayesian Score: -4.213 | | |  | | --- | |  | | B7: -1672512695  0 out of 244 good  Bayesian Score: -4.213 | | |  | | --- | |  | | B8: -91954924  0 out of 222 good  Bayesian Score: -4.120 | | |  | | --- | |  | | B9: -292555972  1 out of 437 good  Bayesian Score: -4.096 | | |  | | --- | |  | | B10: -409182516  0 out of 216 good  Bayesian Score: -4.093 | |
| |  | | --- | |  | | B11: 150794520  0 out of 216 good  Bayesian Score: -4.093 | | |  | | --- | |  | | B12: 2082478181  0 out of 215 good  Bayesian Score: -4.089 | | |  | | --- | |  | | B13: 651217135  0 out of 208 good  Bayesian Score: -4.056 | | |  | | --- | |  | | B14: -936852899  0 out of 208 good  Bayesian Score: -4.056 | | |  | | --- | |  | | B15: -1596132236  0 out of 207 good  Bayesian Score: -4.052 | |
| |  | | --- | |  | | B16: 1635339976  0 out of 206 good  Bayesian Score: -4.047 | | |  | | --- | |  | | B17: 33329059  0 out of 179 good  Bayesian Score: -3.909 | | |  | | --- | |  | | B18: -2038514967  0 out of 175 good  Bayesian Score: -3.887 | | |  | | --- | |  | | B19: 588852368  0 out of 172 good  Bayesian Score: -3.870 | | |  | | --- | |  | | B20: 1965258226  0 out of 171 good  Bayesian Score: -3.864 | |
