# Supplementary material for: Identification of Multi-Target Anti-AD Chemical Constituents From Traditional Chinese Medicine Formulae by Integrating Virtual Screening and In Vitro Validation
Source: Front Pharmacol. 2021 Jul 16;12:709607. doi: 10.3389/fphar.2021.709607 (PMC8322649; doi:10.3389/fphar.2021.709607)
Supplement: Supplementary file 3 [file DataSheet1.ZIP › Good and bad fragments of 52 targets/BCHE.html]

Category Bayesian-buche: good features from ECFP\_6

|  |  |  |  |  |  |  |  |  |  |  |  |  |  |  |
| --- | --- | --- | --- | --- | --- | --- | --- | --- | --- | --- | --- | --- | --- | --- |
| |  | | --- | |  | | G1: 1331561287  217 out of 217 good  Bayesian Score: 1.197 | | |  | | --- | |  | | G2: -514501799  198 out of 198 good  Bayesian Score: 1.196 | | |  | | --- | |  | | G3: -128679049  191 out of 191 good  Bayesian Score: 1.195 | | |  | | --- | |  | | G4: -1056448394  174 out of 174 good  Bayesian Score: 1.194 | | |  | | --- | |  | | G5: 552088469  174 out of 174 good  Bayesian Score: 1.194 | |
| |  | | --- | |  | | G6: 440744344  173 out of 173 good  Bayesian Score: 1.194 | | |  | | --- | |  | | G7: 624344093  170 out of 170 good  Bayesian Score: 1.194 | | |  | | --- | |  | | G8: -1441803809  166 out of 166 good  Bayesian Score: 1.193 | | |  | | --- | |  | | G9: -1442960233  166 out of 166 good  Bayesian Score: 1.193 | | |  | | --- | |  | | G10: -1830436798  165 out of 165 good  Bayesian Score: 1.193 | |
| |  | | --- | |  | | G11: -235775199  164 out of 164 good  Bayesian Score: 1.193 | | |  | | --- | |  | | G12: -354782378  163 out of 163 good  Bayesian Score: 1.193 | | |  | | --- | |  | | G13: 1973629459  162 out of 162 good  Bayesian Score: 1.193 | | |  | | --- | |  | | G14: -1108879003  162 out of 162 good  Bayesian Score: 1.193 | | |  | | --- | |  | | G15: 1334101934  157 out of 157 good  Bayesian Score: 1.192 | |
| |  | | --- | |  | | G16: 805978089  156 out of 156 good  Bayesian Score: 1.192 | | |  | | --- | |  | | G17: -666326105  220 out of 221 good  Bayesian Score: 1.192 | | |  | | --- | |  | | G18: 552399862  145 out of 145 good  Bayesian Score: 1.191 | | |  | | --- | |  | | G19: -212998624  145 out of 145 good  Bayesian Score: 1.191 | | |  | | --- | |  | | G20: 1640603662  138 out of 138 good  Bayesian Score: 1.191 | |

Category Bayesian-buche: bad features from ECFP\_6

|  |  |  |  |  |  |  |  |  |  |  |  |  |  |  |
| --- | --- | --- | --- | --- | --- | --- | --- | --- | --- | --- | --- | --- | --- | --- |
| |  | | --- | |  | | B1: -292555972  0 out of 210 good  Bayesian Score: -4.156 | | |  | | --- | |  | | B2: -1242906247  0 out of 210 good  Bayesian Score: -4.156 | | |  | | --- | |  | | B3: -1813180068  0 out of 191 good  Bayesian Score: -4.062 | | |  | | --- | |  | | B4: -676555381  0 out of 187 good  Bayesian Score: -4.042 | | |  | | --- | |  | | B5: -1507082173  0 out of 186 good  Bayesian Score: -4.036 | |
| |  | | --- | |  | | B6: -1238602038  0 out of 170 good  Bayesian Score: -3.948 | | |  | | --- | |  | | B7: -177264675  0 out of 163 good  Bayesian Score: -3.907 | | |  | | --- | |  | | B8: -797085356  1 out of 309 good  Bayesian Score: -3.844 | | |  | | --- | |  | | B9: 2102150379  1 out of 309 good  Bayesian Score: -3.844 | | |  | | --- | |  | | B10: -2023695201  0 out of 146 good  Bayesian Score: -3.799 | |
| |  | | --- | |  | | B11: 1714259017  0 out of 145 good  Bayesian Score: -3.792 | | |  | | --- | |  | | B12: 1434334340  0 out of 135 good  Bayesian Score: -3.723 | | |  | | --- | |  | | B13: -1596132236  0 out of 134 good  Bayesian Score: -3.715 | | |  | | --- | |  | | B14: -649348348  0 out of 129 good  Bayesian Score: -3.678 | | |  | | --- | |  | | B15: 1658964443  0 out of 125 good  Bayesian Score: -3.647 | |
| |  | | --- | |  | | B16: 1657199878  0 out of 120 good  Bayesian Score: -3.608 | | |  | | --- | |  | | B17: -2040921601  0 out of 120 good  Bayesian Score: -3.608 | | |  | | --- | |  | | B18: -655344035  0 out of 113 good  Bayesian Score: -3.549 | | |  | | --- | |  | | B19: 1167755527  0 out of 112 good  Bayesian Score: -3.541 | | |  | | --- | |  | | B20: 1030853539  0 out of 108 good  Bayesian Score: -3.505 | |
