# Supplementary material for: Identification of Multi-Target Anti-AD Chemical Constituents From Traditional Chinese Medicine Formulae by Integrating Virtual Screening and In Vitro Validation
Source: Front Pharmacol. 2021 Jul 16;12:709607. doi: 10.3389/fphar.2021.709607 (PMC8322649; doi:10.3389/fphar.2021.709607)
Supplement: Supplementary file 3 [file DataSheet1.ZIP › Good and bad fragments of 52 targets/CDK5.html]

Category Bayesian-cdk5: good features from ECFP\_6

|  |  |  |  |  |  |  |  |  |  |  |  |  |  |  |
| --- | --- | --- | --- | --- | --- | --- | --- | --- | --- | --- | --- | --- | --- | --- |
| |  | | --- | |  | | G1: -1443560378  96 out of 96 good  Bayesian Score: 1.338 | | |  | | --- | |  | | G2: -778131027  96 out of 96 good  Bayesian Score: 1.338 | | |  | | --- | |  | | G3: 1705904581  96 out of 96 good  Bayesian Score: 1.338 | | |  | | --- | |  | | G4: 1185390840  96 out of 96 good  Bayesian Score: 1.338 | | |  | | --- | |  | | G5: 354048682  96 out of 96 good  Bayesian Score: 1.338 | |
| |  | | --- | |  | | G6: 1422640156  96 out of 96 good  Bayesian Score: 1.338 | | |  | | --- | |  | | G7: 1608450269  96 out of 96 good  Bayesian Score: 1.338 | | |  | | --- | |  | | G8: -1109401571  96 out of 96 good  Bayesian Score: 1.338 | | |  | | --- | |  | | G9: 163457988  93 out of 93 good  Bayesian Score: 1.337 | | |  | | --- | |  | | G10: -329791374  93 out of 93 good  Bayesian Score: 1.337 | |
| |  | | --- | |  | | G11: 1877121856  92 out of 92 good  Bayesian Score: 1.337 | | |  | | --- | |  | | G12: -311738139  92 out of 92 good  Bayesian Score: 1.337 | | |  | | --- | |  | | G13: 755963777  92 out of 92 good  Bayesian Score: 1.337 | | |  | | --- | |  | | G14: 2021159381  92 out of 92 good  Bayesian Score: 1.337 | | |  | | --- | |  | | G15: 2070263257  89 out of 89 good  Bayesian Score: 1.336 | |
| |  | | --- | |  | | G16: -1224992979  88 out of 88 good  Bayesian Score: 1.335 | | |  | | --- | |  | | G17: -1509297765  88 out of 88 good  Bayesian Score: 1.335 | | |  | | --- | |  | | G18: 79358745  88 out of 88 good  Bayesian Score: 1.335 | | |  | | --- | |  | | G19: -514429668  88 out of 88 good  Bayesian Score: 1.335 | | |  | | --- | |  | | G20: -1449463632  80 out of 80 good  Bayesian Score: 1.332 | |

Category Bayesian-cdk5: bad features from ECFP\_6

|  |  |  |  |  |  |  |  |  |  |  |  |  |  |  |
| --- | --- | --- | --- | --- | --- | --- | --- | --- | --- | --- | --- | --- | --- | --- |
| |  | | --- | |  | | B1: 975766354  0 out of 118 good  Bayesian Score: -3.436 | | |  | | --- | |  | | B2: 1588719643  0 out of 77 good  Bayesian Score: -3.026 | | |  | | --- | |  | | B3: -1686813061  0 out of 63 good  Bayesian Score: -2.836 | | |  | | --- | |  | | B4: 865482986  1 out of 128 good  Bayesian Score: -2.821 | | |  | | --- | |  | | B5: 912478223  0 out of 60 good  Bayesian Score: -2.790 | |
| |  | | --- | |  | | B6: -1049652772  0 out of 56 good  Bayesian Score: -2.726 | | |  | | --- | |  | | B7: -292555972  0 out of 54 good  Bayesian Score: -2.692 | | |  | | --- | |  | | B8: -175882072  1 out of 110 good  Bayesian Score: -2.675 | | |  | | --- | |  | | B9: 1908990050  0 out of 53 good  Bayesian Score: -2.674 | | |  | | --- | |  | | B10: 53207596  0 out of 53 good  Bayesian Score: -2.674 | |
| |  | | --- | |  | | B11: 413587124  0 out of 51 good  Bayesian Score: -2.638 | | |  | | --- | |  | | B12: -1409796893  0 out of 51 good  Bayesian Score: -2.638 | | |  | | --- | |  | | B13: 412256466  1 out of 105 good  Bayesian Score: -2.630 | | |  | | --- | |  | | B14: 2124076609  0 out of 50 good  Bayesian Score: -2.620 | | |  | | --- | |  | | B15: -1693599735  0 out of 50 good  Bayesian Score: -2.620 | |
| |  | | --- | |  | | B16: -327922576  0 out of 49 good  Bayesian Score: -2.601 | | |  | | --- | |  | | B17: 1336540477  0 out of 47 good  Bayesian Score: -2.563 | | |  | | --- | |  | | B18: 657586427  3 out of 199 good  Bayesian Score: -2.559 | | |  | | --- | |  | | B19: 2082478181  0 out of 46 good  Bayesian Score: -2.543 | | |  | | --- | |  | | B20: -1311285389  1 out of 95 good  Bayesian Score: -2.534 | |
