# Supplementary material for: Identification of Multi-Target Anti-AD Chemical Constituents From Traditional Chinese Medicine Formulae by Integrating Virtual Screening and In Vitro Validation
Source: Front Pharmacol. 2021 Jul 16;12:709607. doi: 10.3389/fphar.2021.709607 (PMC8322649; doi:10.3389/fphar.2021.709607)
Supplement: Supplementary file 3 [file DataSheet1.ZIP › Good and bad fragments of 52 targets/CHRM1.html]

Category Bayesian-m1: good features from ECFP\_6

|  |  |  |  |  |  |  |  |  |  |  |  |  |  |  |
| --- | --- | --- | --- | --- | --- | --- | --- | --- | --- | --- | --- | --- | --- | --- |
| |  | | --- | |  | | G1: 1976330679  535 out of 535 good  Bayesian Score: 1.265 | | |  | | --- | |  | | G2: -244159614  452 out of 452 good  Bayesian Score: 1.264 | | |  | | --- | |  | | G3: 1133499173  214 out of 214 good  Bayesian Score: 1.257 | | |  | | --- | |  | | G4: -242828956  138 out of 138 good  Bayesian Score: 1.251 | | |  | | --- | |  | | G5: 1425926360  112 out of 112 good  Bayesian Score: 1.247 | |
| |  | | --- | |  | | G6: 861316614  111 out of 111 good  Bayesian Score: 1.247 | | |  | | --- | |  | | G7: 2069906330  110 out of 110 good  Bayesian Score: 1.247 | | |  | | --- | |  | | G8: -248515768  109 out of 109 good  Bayesian Score: 1.246 | | |  | | --- | |  | | G9: -1926607190  101 out of 101 good  Bayesian Score: 1.245 | | |  | | --- | |  | | G10: -1794005192  97 out of 97 good  Bayesian Score: 1.244 | |
| |  | | --- | |  | | G11: 1960253356  97 out of 97 good  Bayesian Score: 1.244 | | |  | | --- | |  | | G12: 233520344  87 out of 87 good  Bayesian Score: 1.241 | | |  | | --- | |  | | G13: 368983122  73 out of 73 good  Bayesian Score: 1.235 | | |  | | --- | |  | | G14: 2071236988  68 out of 68 good  Bayesian Score: 1.233 | | |  | | --- | |  | | G15: 860114273  64 out of 64 good  Bayesian Score: 1.231 | |
| |  | | --- | |  | | G16: 1151284196  63 out of 63 good  Bayesian Score: 1.230 | | |  | | --- | |  | | G17: -676152452  60 out of 60 good  Bayesian Score: 1.228 | | |  | | --- | |  | | G18: -1072889414  60 out of 60 good  Bayesian Score: 1.228 | | |  | | --- | |  | | G19: -591526139  59 out of 59 good  Bayesian Score: 1.228 | | |  | | --- | |  | | G20: 471451981  58 out of 58 good  Bayesian Score: 1.227 | |

Category Bayesian-m1: bad features from ECFP\_6

|  |  |  |  |  |  |  |  |  |  |  |  |  |  |  |
| --- | --- | --- | --- | --- | --- | --- | --- | --- | --- | --- | --- | --- | --- | --- |
| |  | | --- | |  | | B1: -1832102709  0 out of 140 good  Bayesian Score: -3.697 | | |  | | --- | |  | | B2: -845108448  0 out of 129 good  Bayesian Score: -3.618 | | |  | | --- | |  | | B3: 407900312  0 out of 94 good  Bayesian Score: -3.311 | | |  | | --- | |  | | B4: 1985868180  0 out of 78 good  Bayesian Score: -3.132 | | |  | | --- | |  | | B5: 835630791  1 out of 155 good  Bayesian Score: -3.104 | |
| |  | | --- | |  | | B6: -174914108  0 out of 75 good  Bayesian Score: -3.095 | | |  | | --- | |  | | B7: 544048674  0 out of 72 good  Bayesian Score: -3.056 | | |  | | --- | |  | | B8: -2041399277  0 out of 68 good  Bayesian Score: -3.001 | | |  | | --- | |  | | B9: -175376949  0 out of 68 good  Bayesian Score: -3.001 | | |  | | --- | |  | | B10: 1814278164  0 out of 67 good  Bayesian Score: -2.987 | |
| |  | | --- | |  | | B11: 2025485523  1 out of 121 good  Bayesian Score: -2.862 | | |  | | --- | |  | | B12: -1660913849  0 out of 58 good  Bayesian Score: -2.851 | | |  | | --- | |  | | B13: 912011250  0 out of 58 good  Bayesian Score: -2.851 | | |  | | --- | |  | | B14: 1573945311  0 out of 58 good  Bayesian Score: -2.851 | | |  | | --- | |  | | B15: 1001947487  0 out of 56 good  Bayesian Score: -2.818 | |
| |  | | --- | |  | | B16: 600440273  0 out of 55 good  Bayesian Score: -2.801 | | |  | | --- | |  | | B17: 634582385  0 out of 54 good  Bayesian Score: -2.783 | | |  | | --- | |  | | B18: -955816473  0 out of 51 good  Bayesian Score: -2.730 | | |  | | --- | |  | | B19: 1049075205  0 out of 50 good  Bayesian Score: -2.711 | | |  | | --- | |  | | B20: 2107448306  0 out of 50 good  Bayesian Score: -2.711 | |
