# Supplementary material for: Identification of Multi-Target Anti-AD Chemical Constituents From Traditional Chinese Medicine Formulae by Integrating Virtual Screening and In Vitro Validation
Source: Front Pharmacol. 2021 Jul 16;12:709607. doi: 10.3389/fphar.2021.709607 (PMC8322649; doi:10.3389/fphar.2021.709607)
Supplement: Supplementary file 3 [file DataSheet1.ZIP › Good and bad fragments of 52 targets/CHRM2.html]

Category NB\_M2\_ECFP6: good features from ECFP\_6

|  |  |  |  |  |  |  |  |  |  |  |  |  |  |  |
| --- | --- | --- | --- | --- | --- | --- | --- | --- | --- | --- | --- | --- | --- | --- |
| |  | | --- | |  | | G1: 1640720160  96 out of 96 good  Bayesian Score: 1.189 | | |  | | --- | |  | | G2: 706717463  94 out of 94 good  Bayesian Score: 1.189 | | |  | | --- | |  | | G3: -956348476  94 out of 94 good  Bayesian Score: 1.189 | | |  | | --- | |  | | G4: -1533418246  90 out of 90 good  Bayesian Score: 1.188 | | |  | | --- | |  | | G5: 1274431579  90 out of 90 good  Bayesian Score: 1.188 | |
| |  | | --- | |  | | G6: 850275207  84 out of 84 good  Bayesian Score: 1.186 | | |  | | --- | |  | | G7: -125988021  84 out of 84 good  Bayesian Score: 1.186 | | |  | | --- | |  | | G8: -735303349  84 out of 84 good  Bayesian Score: 1.186 | | |  | | --- | |  | | G9: 741041063  84 out of 84 good  Bayesian Score: 1.186 | | |  | | --- | |  | | G10: 1998938409  84 out of 84 good  Bayesian Score: 1.186 | |
| |  | | --- | |  | | G11: -1555581667  83 out of 83 good  Bayesian Score: 1.185 | | |  | | --- | |  | | G12: -579943384  83 out of 83 good  Bayesian Score: 1.185 | | |  | | --- | |  | | G13: -1526814620  83 out of 83 good  Bayesian Score: 1.185 | | |  | | --- | |  | | G14: 843747072  82 out of 82 good  Bayesian Score: 1.185 | | |  | | --- | |  | | G15: 1979921154  81 out of 81 good  Bayesian Score: 1.185 | |
| |  | | --- | |  | | G16: -1026362178  81 out of 81 good  Bayesian Score: 1.185 | | |  | | --- | |  | | G17: 2018545296  78 out of 78 good  Bayesian Score: 1.184 | | |  | | --- | |  | | G18: -2038354784  77 out of 77 good  Bayesian Score: 1.183 | | |  | | --- | |  | | G19: -1738566027  77 out of 77 good  Bayesian Score: 1.183 | | |  | | --- | |  | | G20: 253645935  76 out of 76 good  Bayesian Score: 1.183 | |

Category NB\_M2\_ECFP6: bad features from ECFP\_6

|  |  |  |  |  |  |  |  |  |  |  |  |  |  |  |
| --- | --- | --- | --- | --- | --- | --- | --- | --- | --- | --- | --- | --- | --- | --- |
| |  | | --- | |  | | B1: 1961554343  0 out of 243 good  Bayesian Score: -4.294 | | |  | | --- | |  | | B2: 834876373  0 out of 176 good  Bayesian Score: -3.976 | | |  | | --- | |  | | B3: 1976330679  0 out of 175 good  Bayesian Score: -3.971 | | |  | | --- | |  | | B4: -244159614  0 out of 139 good  Bayesian Score: -3.745 | | |  | | --- | |  | | B5: -591526139  0 out of 136 good  Bayesian Score: -3.724 | |
| |  | | --- | |  | | B6: 85262808  0 out of 120 good  Bayesian Score: -3.602 | | |  | | --- | |  | | B7: -1416572622  0 out of 116 good  Bayesian Score: -3.569 | | |  | | --- | |  | | B8: 1133499173  0 out of 95 good  Bayesian Score: -3.375 | | |  | | --- | |  | | B9: 1994668215  0 out of 90 good  Bayesian Score: -3.323 | | |  | | --- | |  | | B10: 2116455019  0 out of 84 good  Bayesian Score: -3.257 | |
| |  | | --- | |  | | B11: 1997522062  0 out of 76 good  Bayesian Score: -3.161 | | |  | | --- | |  | | B12: -1508366470  0 out of 72 good  Bayesian Score: -3.109 | | |  | | --- | |  | | B13: 233520344  0 out of 70 good  Bayesian Score: -3.082 | | |  | | --- | |  | | B14: -666326105  0 out of 64 good  Bayesian Score: -2.997 | | |  | | --- | |  | | B15: 1151284196  0 out of 59 good  Bayesian Score: -2.920 | |
| |  | | --- | |  | | B16: 860114273  0 out of 58 good  Bayesian Score: -2.904 | | |  | | --- | |  | | B17: -152683720  0 out of 56 good  Bayesian Score: -2.870 | | |  | | --- | |  | | B18: 469398259  0 out of 56 good  Bayesian Score: -2.870 | | |  | | --- | |  | | B19: 835630791  0 out of 56 good  Bayesian Score: -2.870 | | |  | | --- | |  | | B20: 413587124  0 out of 52 good  Bayesian Score: -2.801 | |
