# Supplementary material for: Identification of Multi-Target Anti-AD Chemical Constituents From Traditional Chinese Medicine Formulae by Integrating Virtual Screening and In Vitro Validation
Source: Front Pharmacol. 2021 Jul 16;12:709607. doi: 10.3389/fphar.2021.709607 (PMC8322649; doi:10.3389/fphar.2021.709607)
Supplement: Supplementary file 3 [file DataSheet1.ZIP › Good and bad fragments of 52 targets/CHRNA4.html]

Category Bayesian-Alpha4: good features from ECFP\_6

|  |  |  |  |  |  |  |  |  |  |  |  |  |  |  |
| --- | --- | --- | --- | --- | --- | --- | --- | --- | --- | --- | --- | --- | --- | --- |
| |  | | --- | |  | | G1: 1951894094  68 out of 69 good  Bayesian Score: 1.262 | | |  | | --- | |  | | G2: 2101140853  44 out of 44 good  Bayesian Score: 1.256 | | |  | | --- | |  | | G3: -1743825633  40 out of 40 good  Bayesian Score: 1.250 | | |  | | --- | |  | | G4: 2107009525  39 out of 39 good  Bayesian Score: 1.248 | | |  | | --- | |  | | G5: -334506388  34 out of 34 good  Bayesian Score: 1.239 | |
| |  | | --- | |  | | G6: -175021654  83 out of 87 good  Bayesian Score: 1.237 | | |  | | --- | |  | | G7: 1681442586  29 out of 29 good  Bayesian Score: 1.227 | | |  | | --- | |  | | G8: 704462208  27 out of 27 good  Bayesian Score: 1.221 | | |  | | --- | |  | | G9: -349755492  25 out of 25 good  Bayesian Score: 1.215 | | |  | | --- | |  | | G10: 863104279  25 out of 25 good  Bayesian Score: 1.215 | |
| |  | | --- | |  | | G11: 173678427  25 out of 25 good  Bayesian Score: 1.215 | | |  | | --- | |  | | G12: -921935251  25 out of 25 good  Bayesian Score: 1.215 | | |  | | --- | |  | | G13: -1334291067  25 out of 25 good  Bayesian Score: 1.215 | | |  | | --- | |  | | G14: 421942661  25 out of 25 good  Bayesian Score: 1.215 | | |  | | --- | |  | | G15: -364029422  25 out of 25 good  Bayesian Score: 1.215 | |
| |  | | --- | |  | | G16: 2138647081  24 out of 24 good  Bayesian Score: 1.211 | | |  | | --- | |  | | G17: -1921235997  24 out of 24 good  Bayesian Score: 1.211 | | |  | | --- | |  | | G18: -1355310510  24 out of 24 good  Bayesian Score: 1.211 | | |  | | --- | |  | | G19: 1372469331  24 out of 24 good  Bayesian Score: 1.211 | | |  | | --- | |  | | G20: -2097986999  24 out of 24 good  Bayesian Score: 1.211 | |

Category Bayesian-Alpha4: bad features from ECFP\_6

|  |  |  |  |  |  |  |  |  |  |  |  |  |  |  |
| --- | --- | --- | --- | --- | --- | --- | --- | --- | --- | --- | --- | --- | --- | --- |
| |  | | --- | |  | | B1: 1961554343  0 out of 165 good  Bayesian Score: -3.814 | | |  | | --- | |  | | B2: 1994668215  0 out of 65 good  Bayesian Score: -2.916 | | |  | | --- | |  | | B3: 2116455019  0 out of 60 good  Bayesian Score: -2.840 | | |  | | --- | |  | | B4: 51876938  0 out of 51 good  Bayesian Score: -2.688 | | |  | | --- | |  | | B5: -591526139  0 out of 47 good  Bayesian Score: -2.612 | |
| |  | | --- | |  | | B6: -1085223908  0 out of 41 good  Bayesian Score: -2.486 | | |  | | --- | |  | | B7: 912478223  0 out of 41 good  Bayesian Score: -2.486 | | |  | | --- | |  | | B8: -1416572622  0 out of 36 good  Bayesian Score: -2.368 | | |  | | --- | |  | | B9: 2116709167  0 out of 34 good  Bayesian Score: -2.316 | | |  | | --- | |  | | B10: 914325265  1 out of 64 good  Bayesian Score: -2.208 | |
| |  | | --- | |  | | B11: -666326105  0 out of 29 good  Bayesian Score: -2.174 | | |  | | --- | |  | | B12: -1699286547  0 out of 29 good  Bayesian Score: -2.174 | | |  | | --- | |  | | B13: -152683720  0 out of 27 good  Bayesian Score: -2.111 | | |  | | --- | |  | | B14: 2102150379  0 out of 26 good  Bayesian Score: -2.078 | | |  | | --- | |  | | B15: -788112909  0 out of 26 good  Bayesian Score: -2.078 | |
| |  | | --- | |  | | B16: -797085356  0 out of 26 good  Bayesian Score: -2.078 | | |  | | --- | |  | | B17: -91640731  0 out of 26 good  Bayesian Score: -2.078 | | |  | | --- | |  | | B18: -1508366470  0 out of 25 good  Bayesian Score: -2.043 | | |  | | --- | |  | | B19: 1333660716  0 out of 25 good  Bayesian Score: -2.043 | | |  | | --- | |  | | B20: -154530762  1 out of 52 good  Bayesian Score: -2.013 | |
