# Supplementary material for: Identification of Multi-Target Anti-AD Chemical Constituents From Traditional Chinese Medicine Formulae by Integrating Virtual Screening and In Vitro Validation
Source: Front Pharmacol. 2021 Jul 16;12:709607. doi: 10.3389/fphar.2021.709607 (PMC8322649; doi:10.3389/fphar.2021.709607)
Supplement: Supplementary file 3 [file DataSheet1.ZIP › Good and bad fragments of 52 targets/CHRNA7.html]

Category Bayesian-Alpha7: good features from ECFP\_6

|  |  |  |  |  |  |  |  |  |  |  |  |  |  |  |
| --- | --- | --- | --- | --- | --- | --- | --- | --- | --- | --- | --- | --- | --- | --- |
| |  | | --- | |  | | G1: 1960253356  132 out of 133 good  Bayesian Score: 1.322 | | |  | | --- | |  | | G2: -705668329  81 out of 81 good  Bayesian Score: 1.317 | | |  | | --- | |  | | G3: 1926900472  80 out of 80 good  Bayesian Score: 1.316 | | |  | | --- | |  | | G4: -144257676  80 out of 80 good  Bayesian Score: 1.316 | | |  | | --- | |  | | G5: 2069906330  132 out of 134 good  Bayesian Score: 1.315 | |
| |  | | --- | |  | | G6: -687573058  77 out of 77 good  Bayesian Score: 1.315 | | |  | | --- | |  | | G7: 427873519  71 out of 71 good  Bayesian Score: 1.312 | | |  | | --- | |  | | G8: 1739010345  71 out of 71 good  Bayesian Score: 1.312 | | |  | | --- | |  | | G9: -1224030765  70 out of 70 good  Bayesian Score: 1.311 | | |  | | --- | |  | | G10: 679151718  60 out of 60 good  Bayesian Score: 1.305 | |
| |  | | --- | |  | | G11: -1859163480  60 out of 60 good  Bayesian Score: 1.305 | | |  | | --- | |  | | G12: 235388692  60 out of 60 good  Bayesian Score: 1.305 | | |  | | --- | |  | | G13: -117006870  60 out of 60 good  Bayesian Score: 1.305 | | |  | | --- | |  | | G14: -359734345  60 out of 60 good  Bayesian Score: 1.305 | | |  | | --- | |  | | G15: 306403611  60 out of 60 good  Bayesian Score: 1.305 | |
| |  | | --- | |  | | G16: 1262362191  55 out of 55 good  Bayesian Score: 1.301 | | |  | | --- | |  | | G17: -1441606516  55 out of 55 good  Bayesian Score: 1.301 | | |  | | --- | |  | | G18: -1439437166  55 out of 55 good  Bayesian Score: 1.301 | | |  | | --- | |  | | G19: -1554150152  126 out of 130 good  Bayesian Score: 1.298 | | |  | | --- | |  | | G20: -256888478  43 out of 43 good  Bayesian Score: 1.288 | |

Category Bayesian-Alpha7: bad features from ECFP\_6

|  |  |  |  |  |  |  |  |  |  |  |  |  |  |  |
| --- | --- | --- | --- | --- | --- | --- | --- | --- | --- | --- | --- | --- | --- | --- |
| |  | | --- | |  | | B1: 1427820655  0 out of 191 good  Bayesian Score: -3.921 | | |  | | --- | |  | | B2: -955816473  0 out of 107 good  Bayesian Score: -3.357 | | |  | | --- | |  | | B3: 2085698692  0 out of 100 good  Bayesian Score: -3.292 | | |  | | --- | |  | | B4: 2082478181  0 out of 95 good  Bayesian Score: -3.243 | | |  | | --- | |  | | B5: -1699286547  0 out of 81 good  Bayesian Score: -3.090 | |
| |  | | --- | |  | | B6: 1986731747  0 out of 79 good  Bayesian Score: -3.066 | | |  | | --- | |  | | B7: -1087070950  1 out of 147 good  Bayesian Score: -2.972 | | |  | | --- | |  | | B8: 2122741631  0 out of 71 good  Bayesian Score: -2.965 | | |  | | --- | |  | | B9: 770725373  0 out of 71 good  Bayesian Score: -2.965 | | |  | | --- | |  | | B10: -661766797  0 out of 68 good  Bayesian Score: -2.924 | |
| |  | | --- | |  | | B11: -1661653144  0 out of 66 good  Bayesian Score: -2.896 | | |  | | --- | |  | | B12: -91954924  0 out of 64 good  Bayesian Score: -2.867 | | |  | | --- | |  | | B13: 768590601  0 out of 63 good  Bayesian Score: -2.852 | | |  | | --- | |  | | B14: -1672647522  0 out of 63 good  Bayesian Score: -2.852 | | |  | | --- | |  | | B15: -845108448  1 out of 125 good  Bayesian Score: -2.815 | |
| |  | | --- | |  | | B16: -1715064478  0 out of 60 good  Bayesian Score: -2.806 | | |  | | --- | |  | | B17: 835630791  0 out of 57 good  Bayesian Score: -2.758 | | |  | | --- | |  | | B18: -1686813061  0 out of 57 good  Bayesian Score: -2.758 | | |  | | --- | |  | | B19: 771018251  0 out of 55 good  Bayesian Score: -2.724 | | |  | | --- | |  | | B20: 103339584  0 out of 53 good  Bayesian Score: -2.690 | |
