# Supplementary material for: Identification of Multi-Target Anti-AD Chemical Constituents From Traditional Chinese Medicine Formulae by Integrating Virtual Screening and In Vitro Validation
Source: Front Pharmacol. 2021 Jul 16;12:709607. doi: 10.3389/fphar.2021.709607 (PMC8322649; doi:10.3389/fphar.2021.709607)
Supplement: Supplementary file 3 [file DataSheet1.ZIP › Good and bad fragments of 52 targets/CHUK.html]

Category NB\_IKK-alpha\_ECFP6: good features from ECFP\_6

|  |  |  |  |  |  |  |  |  |  |  |  |  |  |  |
| --- | --- | --- | --- | --- | --- | --- | --- | --- | --- | --- | --- | --- | --- | --- |
| |  | | --- | |  | | G1: 957530031  48 out of 48 good  Bayesian Score: 1.234 | | |  | | --- | |  | | G2: 1753866522  46 out of 46 good  Bayesian Score: 1.232 | | |  | | --- | |  | | G3: -1560285075  45 out of 45 good  Bayesian Score: 1.231 | | |  | | --- | |  | | G4: -400248150  45 out of 45 good  Bayesian Score: 1.231 | | |  | | --- | |  | | G5: 1313996642  45 out of 45 good  Bayesian Score: 1.231 | |
| |  | | --- | |  | | G6: -1064325344  45 out of 45 good  Bayesian Score: 1.231 | | |  | | --- | |  | | G7: -1917713356  44 out of 44 good  Bayesian Score: 1.230 | | |  | | --- | |  | | G8: 1834679071  44 out of 44 good  Bayesian Score: 1.230 | | |  | | --- | |  | | G9: -1488648724  41 out of 41 good  Bayesian Score: 1.226 | | |  | | --- | |  | | G10: -1673205830  41 out of 41 good  Bayesian Score: 1.226 | |
| |  | | --- | |  | | G11: -974936570  41 out of 41 good  Bayesian Score: 1.226 | | |  | | --- | |  | | G12: -740522194  41 out of 41 good  Bayesian Score: 1.226 | | |  | | --- | |  | | G13: -1554546344  41 out of 41 good  Bayesian Score: 1.226 | | |  | | --- | |  | | G14: -351439243  40 out of 40 good  Bayesian Score: 1.224 | | |  | | --- | |  | | G15: 971221433  40 out of 40 good  Bayesian Score: 1.224 | |
| |  | | --- | |  | | G16: 2030787833  40 out of 40 good  Bayesian Score: 1.224 | | |  | | --- | |  | | G17: -965460789  40 out of 40 good  Bayesian Score: 1.224 | | |  | | --- | |  | | G18: 1139371378  39 out of 39 good  Bayesian Score: 1.223 | | |  | | --- | |  | | G19: -277717775  36 out of 36 good  Bayesian Score: 1.218 | | |  | | --- | |  | | G20: 1280539993  36 out of 36 good  Bayesian Score: 1.218 | |

Category NB\_IKK-alpha\_ECFP6: bad features from ECFP\_6

|  |  |  |  |  |  |  |  |  |  |  |  |  |  |  |
| --- | --- | --- | --- | --- | --- | --- | --- | --- | --- | --- | --- | --- | --- | --- |
| |  | | --- | |  | | B1: 657586427  0 out of 72 good  Bayesian Score: -3.040 | | |  | | --- | |  | | B2: 1961554343  0 out of 70 good  Bayesian Score: -3.013 | | |  | | --- | |  | | B3: 781519895  0 out of 58 good  Bayesian Score: -2.835 | | |  | | --- | |  | | B4: -1236483485  0 out of 54 good  Bayesian Score: -2.768 | | |  | | --- | |  | | B5: -302078100  0 out of 44 good  Bayesian Score: -2.577 | |
| |  | | --- | |  | | B6: 459826767  0 out of 44 good  Bayesian Score: -2.577 | | |  | | --- | |  | | B7: -1087070950  0 out of 44 good  Bayesian Score: -2.577 | | |  | | --- | |  | | B8: 51876938  0 out of 38 good  Bayesian Score: -2.442 | | |  | | --- | |  | | B9: 865482986  0 out of 35 good  Bayesian Score: -2.368 | | |  | | --- | |  | | B10: -1416572622  0 out of 34 good  Bayesian Score: -2.341 | |
| |  | | --- | |  | | B11: -801490360  0 out of 33 good  Bayesian Score: -2.314 | | |  | | --- | |  | | B12: 859433814  0 out of 32 good  Bayesian Score: -2.287 | | |  | | --- | |  | | B13: 412256466  0 out of 32 good  Bayesian Score: -2.287 | | |  | | --- | |  | | B14: 663943468  0 out of 30 good  Bayesian Score: -2.229 | | |  | | --- | |  | | B15: -175882072  0 out of 29 good  Bayesian Score: -2.199 | |
| |  | | --- | |  | | B16: 464808839  0 out of 28 good  Bayesian Score: -2.168 | | |  | | --- | |  | | B17: 2106656448  2 out of 90 good  Bayesian Score: -2.154 | | |  | | --- | |  | | B18: 1335108269  0 out of 26 good  Bayesian Score: -2.102 | | |  | | --- | |  | | B19: 1994668215  0 out of 26 good  Bayesian Score: -2.102 | | |  | | --- | |  | | B20: 912478223  0 out of 26 good  Bayesian Score: -2.102 | |
