# Supplementary material for: Identification of Multi-Target Anti-AD Chemical Constituents From Traditional Chinese Medicine Formulae by Integrating Virtual Screening and In Vitro Validation
Source: Front Pharmacol. 2021 Jul 16;12:709607. doi: 10.3389/fphar.2021.709607 (PMC8322649; doi:10.3389/fphar.2021.709607)
Supplement: Supplementary file 3 [file DataSheet1.ZIP › Good and bad fragments of 52 targets/COMT.html]

Category Bayesian-comt: good features from ECFP\_6

|  |  |  |  |  |  |  |  |  |  |  |  |  |  |  |
| --- | --- | --- | --- | --- | --- | --- | --- | --- | --- | --- | --- | --- | --- | --- |
| |  | | --- | |  | | G1: 1334400011  40 out of 40 good  Bayesian Score: 1.273 | | |  | | --- | |  | | G2: -1660913849  36 out of 36 good  Bayesian Score: 1.266 | | |  | | --- | |  | | G3: -1553987716  35 out of 35 good  Bayesian Score: 1.264 | | |  | | --- | |  | | G4: 2019062761  40 out of 42 good  Bayesian Score: 1.228 | | |  | | --- | |  | | G5: -659271057  30 out of 31 good  Bayesian Score: 1.223 | |
| |  | | --- | |  | | G6: -215026467  30 out of 31 good  Bayesian Score: 1.223 | | |  | | --- | |  | | G7: 2104376220  30 out of 31 good  Bayesian Score: 1.223 | | |  | | --- | |  | | G8: 1043790491  30 out of 31 good  Bayesian Score: 1.223 | | |  | | --- | |  | | G9: -1742225957  20 out of 20 good  Bayesian Score: 1.214 | | |  | | --- | |  | | G10: -1956535100  20 out of 20 good  Bayesian Score: 1.214 | |
| |  | | --- | |  | | G11: 122891423  18 out of 18 good  Bayesian Score: 1.201 | | |  | | --- | |  | | G12: -42449291  18 out of 18 good  Bayesian Score: 1.201 | | |  | | --- | |  | | G13: 1983213501  18 out of 18 good  Bayesian Score: 1.201 | | |  | | --- | |  | | G14: -1553278795  12 out of 12 good  Bayesian Score: 1.143 | | |  | | --- | |  | | G15: -1783972864  10 out of 10 good  Bayesian Score: 1.111 | |
| |  | | --- | |  | | G16: -1736370106  10 out of 10 good  Bayesian Score: 1.111 | | |  | | --- | |  | | G17: 1370545344  10 out of 10 good  Bayesian Score: 1.111 | | |  | | --- | |  | | G18: -414653861  10 out of 10 good  Bayesian Score: 1.111 | | |  | | --- | |  | | G19: 1106989382  9 out of 9 good  Bayesian Score: 1.091 | | |  | | --- | |  | | G20: -336844959  9 out of 9 good  Bayesian Score: 1.091 | |

Category Bayesian-comt: bad features from ECFP\_6

|  |  |  |  |  |  |  |  |  |  |  |  |  |  |  |
| --- | --- | --- | --- | --- | --- | --- | --- | --- | --- | --- | --- | --- | --- | --- |
| |  | | --- | |  | | B1: 655739385  0 out of 47 good  Bayesian Score: -2.589 | | |  | | --- | |  | | B2: -1331450522  0 out of 31 good  Bayesian Score: -2.211 | | |  | | --- | |  | | B3: -1332781180  0 out of 28 good  Bayesian Score: -2.121 | | |  | | --- | |  | | B4: 1572579716  0 out of 27 good  Bayesian Score: -2.089 | | |  | | --- | |  | | B5: -154530762  0 out of 17 good  Bayesian Score: -1.696 | |
| |  | | --- | |  | | B6: -1087070950  0 out of 17 good  Bayesian Score: -1.696 | | |  | | --- | |  | | B7: 865857320  0 out of 15 good  Bayesian Score: -1.595 | | |  | | --- | |  | | B8: 670515721  0 out of 14 good  Bayesian Score: -1.541 | | |  | | --- | |  | | B9: -938530932  0 out of 14 good  Bayesian Score: -1.541 | | |  | | --- | |  | | B10: 220735655  0 out of 13 good  Bayesian Score: -1.483 | |
| |  | | --- | |  | | B11: 834876373  0 out of 13 good  Bayesian Score: -1.483 | | |  | | --- | |  | | B12: 663943468  0 out of 12 good  Bayesian Score: -1.422 | | |  | | --- | |  | | B13: 859433814  0 out of 11 good  Bayesian Score: -1.356 | | |  | | --- | |  | | B14: 683445015  1 out of 24 good  Bayesian Score: -1.293 | | |  | | --- | |  | | B15: -152683720  0 out of 10 good  Bayesian Score: -1.286 | |
| |  | | --- | |  | | B16: 662850656  0 out of 10 good  Bayesian Score: -1.286 | | |  | | --- | |  | | B17: 412256466  0 out of 10 good  Bayesian Score: -1.286 | | |  | | --- | |  | | B18: -167460056  4 out of 62 good  Bayesian Score: -1.238 | | |  | | --- | |  | | B19: -176686665  0 out of 9 good  Bayesian Score: -1.211 | | |  | | --- | |  | | B20: -2068328535  0 out of 9 good  Bayesian Score: -1.211 | |
