# Supplementary material for: Identification of Multi-Target Anti-AD Chemical Constituents From Traditional Chinese Medicine Formulae by Integrating Virtual Screening and In Vitro Validation
Source: Front Pharmacol. 2021 Jul 16;12:709607. doi: 10.3389/fphar.2021.709607 (PMC8322649; doi:10.3389/fphar.2021.709607)
Supplement: Supplementary file 3 [file DataSheet1.ZIP › Good and bad fragments of 52 targets/ESR1.html]

Category BayesianTempModel-moe-ecfp-6: good features from ECFP\_6

|  |  |  |  |  |  |  |  |  |  |  |  |  |  |  |
| --- | --- | --- | --- | --- | --- | --- | --- | --- | --- | --- | --- | --- | --- | --- |
| |  | | --- | |  | | G1: -790637051  809 out of 813 good  Bayesian Score: 1.746 | | |  | | --- | |  | | G2: 477042227  549 out of 551 good  Bayesian Score: 1.744 | | |  | | --- | |  | | G3: -177786161  852 out of 858 good  Bayesian Score: 1.744 | | |  | | --- | |  | | G4: 1419645508  433 out of 437 good  Bayesian Score: 1.737 | | |  | | --- | |  | | G5: 1740779540  433 out of 437 good  Bayesian Score: 1.737 | |
| |  | | --- | |  | | G6: 1473319571  215 out of 215 good  Bayesian Score: 1.735 | | |  | | --- | |  | | G7: -1281742043  204 out of 204 good  Bayesian Score: 1.733 | | |  | | --- | |  | | G8: 691947046  197 out of 197 good  Bayesian Score: 1.733 | | |  | | --- | |  | | G9: 1944720632  188 out of 188 good  Bayesian Score: 1.732 | | |  | | --- | |  | | G10: -493345410  180 out of 180 good  Bayesian Score: 1.730 | |
| |  | | --- | |  | | G11: 1743565986  176 out of 176 good  Bayesian Score: 1.730 | | |  | | --- | |  | | G12: 1672695278  203 out of 204 good  Bayesian Score: 1.729 | | |  | | --- | |  | | G13: 445756558  166 out of 166 good  Bayesian Score: 1.728 | | |  | | --- | |  | | G14: 2131972380  198 out of 199 good  Bayesian Score: 1.728 | | |  | | --- | |  | | G15: 1375517620  163 out of 163 good  Bayesian Score: 1.728 | |
| |  | | --- | |  | | G16: 1600442368  145 out of 145 good  Bayesian Score: 1.724 | | |  | | --- | |  | | G17: 778899417  187 out of 189 good  Bayesian Score: 1.721 | | |  | | --- | |  | | G18: 1238597748  125 out of 125 good  Bayesian Score: 1.719 | | |  | | --- | |  | | G19: 1644948055  120 out of 120 good  Bayesian Score: 1.718 | | |  | | --- | |  | | G20: 2124076609  120 out of 120 good  Bayesian Score: 1.718 | |

Category BayesianTempModel-moe-ecfp-6: bad features from ECFP\_6

|  |  |  |  |  |  |  |  |  |  |  |  |  |  |  |
| --- | --- | --- | --- | --- | --- | --- | --- | --- | --- | --- | --- | --- | --- | --- |
| |  | | --- | |  | | B1: 1961554343  0 out of 1293 good  Bayesian Score: -5.413 | | |  | | --- | |  | | B2: 1976330679  0 out of 796 good  Bayesian Score: -4.930 | | |  | | --- | |  | | B3: -244159614  0 out of 660 good  Bayesian Score: -4.744 | | |  | | --- | |  | | B4: -1416572622  0 out of 528 good  Bayesian Score: -4.523 | | |  | | --- | |  | | B5: -591526139  0 out of 501 good  Bayesian Score: -4.472 | |
| |  | | --- | |  | | B6: -666326105  0 out of 491 good  Bayesian Score: -4.452 | | |  | | --- | |  | | B7: 1151284196  0 out of 463 good  Bayesian Score: -4.394 | | |  | | --- | |  | | B8: 1133499173  0 out of 321 good  Bayesian Score: -4.033 | | |  | | --- | |  | | B9: -661766797  0 out of 312 good  Bayesian Score: -4.005 | | |  | | --- | |  | | B10: 1979182050  0 out of 301 good  Bayesian Score: -3.970 | |
| |  | | --- | |  | | B11: 1331561287  0 out of 284 good  Bayesian Score: -3.913 | | |  | | --- | |  | | B12: 2116455019  0 out of 281 good  Bayesian Score: -3.902 | | |  | | --- | |  | | B13: 1994668215  0 out of 260 good  Bayesian Score: -3.826 | | |  | | --- | |  | | B14: 233520344  0 out of 257 good  Bayesian Score: -3.815 | | |  | | --- | |  | | B15: -1237219435  0 out of 238 good  Bayesian Score: -3.740 | |
| |  | | --- | |  | | B16: 908605940  0 out of 234 good  Bayesian Score: -3.723 | | |  | | --- | |  | | B17: 772040410  0 out of 225 good  Bayesian Score: -3.685 | | |  | | --- | |  | | B18: -2128944818  0 out of 218 good  Bayesian Score: -3.654 | | |  | | --- | |  | | B19: 469398259  0 out of 210 good  Bayesian Score: -3.618 | | |  | | --- | |  | | B20: -655344035  1 out of 388 good  Bayesian Score: -3.526 | |
