# Supplementary material for: Identification of Multi-Target Anti-AD Chemical Constituents From Traditional Chinese Medicine Formulae by Integrating Virtual Screening and In Vitro Validation
Source: Front Pharmacol. 2021 Jul 16;12:709607. doi: 10.3389/fphar.2021.709607 (PMC8322649; doi:10.3389/fphar.2021.709607)
Supplement: Supplementary file 3 [file DataSheet1.ZIP › Good and bad fragments of 52 targets/GABBR1.html]

Category Bayesian-GABA-B: good features from ECFP\_6

|  |  |  |  |  |  |  |  |  |  |  |  |  |  |  |
| --- | --- | --- | --- | --- | --- | --- | --- | --- | --- | --- | --- | --- | --- | --- |
| |  | | --- | |  | | G1: -826638028  11 out of 11 good  Bayesian Score: 1.240 | | |  | | --- | |  | | G2: -1690551527  11 out of 11 good  Bayesian Score: 1.240 | | |  | | --- | |  | | G3: 2100964382  11 out of 11 good  Bayesian Score: 1.240 | | |  | | --- | |  | | G4: -656499981  11 out of 11 good  Bayesian Score: 1.240 | | |  | | --- | |  | | G5: -2130275420  28 out of 34 good  Bayesian Score: 1.211 | |
| |  | | --- | |  | | G6: -1790003781  14 out of 16 good  Bayesian Score: 1.183 | | |  | | --- | |  | | G7: -1311760795  11 out of 12 good  Bayesian Score: 1.177 | | |  | | --- | |  | | G8: -1792133435  11 out of 12 good  Bayesian Score: 1.177 | | |  | | --- | |  | | G9: 192301107  8 out of 8 good  Bayesian Score: 1.168 | | |  | | --- | |  | | G10: 1900450476  7 out of 7 good  Bayesian Score: 1.134 | |
| |  | | --- | |  | | G11: 2102723523  7 out of 7 good  Bayesian Score: 1.134 | | |  | | --- | |  | | G12: -657838016  7 out of 7 good  Bayesian Score: 1.134 | | |  | | --- | |  | | G13: -1651684511  7 out of 7 good  Bayesian Score: 1.134 | | |  | | --- | |  | | G14: 1280143826  9 out of 10 good  Bayesian Score: 1.125 | | |  | | --- | |  | | G15: -1038144875  6 out of 6 good  Bayesian Score: 1.092 | |
| |  | | --- | |  | | G16: -1310859884  6 out of 6 good  Bayesian Score: 1.092 | | |  | | --- | |  | | G17: 1000942765  6 out of 6 good  Bayesian Score: 1.092 | | |  | | --- | |  | | G18: -1293076817  6 out of 6 good  Bayesian Score: 1.092 | | |  | | --- | |  | | G19: 1382777057  6 out of 6 good  Bayesian Score: 1.092 | | |  | | --- | |  | | G20: -1790258816  6 out of 6 good  Bayesian Score: 1.092 | |

Category Bayesian-GABA-B: bad features from ECFP\_6

|  |  |  |  |  |  |  |  |  |  |  |  |  |  |  |
| --- | --- | --- | --- | --- | --- | --- | --- | --- | --- | --- | --- | --- | --- | --- |
| |  | | --- | |  | | B1: 1572579716  0 out of 36 good  Bayesian Score: -2.207 | | |  | | --- | |  | | B2: 655739385  0 out of 28 good  Bayesian Score: -1.987 | | |  | | --- | |  | | B3: -1897341097  0 out of 24 good  Bayesian Score: -1.856 | | |  | | --- | |  | | B4: 2106656448  0 out of 19 good  Bayesian Score: -1.662 | | |  | | --- | |  | | B5: -932108170  0 out of 17 good  Bayesian Score: -1.573 | |
| |  | | --- | |  | | B6: -992506539  1 out of 34 good  Bayesian Score: -1.464 | | |  | | --- | |  | | B7: -154530762  0 out of 12 good  Bayesian Score: -1.308 | | |  | | --- | |  | | B8: -167460056  1 out of 25 good  Bayesian Score: -1.197 | | |  | | --- | |  | | B9: -938530932  0 out of 10 good  Bayesian Score: -1.178 | | |  | | --- | |  | | B10: -1332781180  0 out of 9 good  Bayesian Score: -1.106 | |
| |  | | --- | |  | | B11: -1114776580  0 out of 9 good  Bayesian Score: -1.106 | | |  | | --- | |  | | B12: -709633021  0 out of 9 good  Bayesian Score: -1.106 | | |  | | --- | |  | | B13: 657586427  0 out of 9 good  Bayesian Score: -1.106 | | |  | | --- | |  | | B14: -677309799  0 out of 8 good  Bayesian Score: -1.029 | | |  | | --- | |  | | B15: -1101847286  0 out of 8 good  Bayesian Score: -1.029 | |
| |  | | --- | |  | | B16: 1412053881  0 out of 8 good  Bayesian Score: -1.029 | | |  | | --- | |  | | B17: -845108448  0 out of 8 good  Bayesian Score: -1.029 | | |  | | --- | |  | | B18: -830332112  0 out of 7 good  Bayesian Score: -0.945 | | |  | | --- | |  | | B19: -934039951  0 out of 7 good  Bayesian Score: -0.945 | | |  | | --- | |  | | B20: 1979182050  0 out of 6 good  Bayesian Score: -0.854 | |
