# Supplementary material for: Identification of Multi-Target Anti-AD Chemical Constituents From Traditional Chinese Medicine Formulae by Integrating Virtual Screening and In Vitro Validation
Source: Front Pharmacol. 2021 Jul 16;12:709607. doi: 10.3389/fphar.2021.709607 (PMC8322649; doi:10.3389/fphar.2021.709607)
Supplement: Supplementary file 3 [file DataSheet1.ZIP › Good and bad fragments of 52 targets/GABRG1.html]

Category Bayesian-GABA-A: good features from ECFP\_6

|  |  |  |  |  |  |  |  |  |  |  |  |  |  |  |
| --- | --- | --- | --- | --- | --- | --- | --- | --- | --- | --- | --- | --- | --- | --- |
| |  | | --- | |  | | G1: 1481235578  17 out of 17 good  Bayesian Score: 1.038 | | |  | | --- | |  | | G2: -1794005192  13 out of 13 good  Bayesian Score: 1.008 | | |  | | --- | |  | | G3: 368983122  13 out of 13 good  Bayesian Score: 1.008 | | |  | | --- | |  | | G4: -280629989  12 out of 12 good  Bayesian Score: 0.998 | | |  | | --- | |  | | G5: 1639827160  12 out of 12 good  Bayesian Score: 0.998 | |
| |  | | --- | |  | | G6: 1099224616  17 out of 18 good  Bayesian Score: 0.990 | | |  | | --- | |  | | G7: 1306977740  15 out of 16 good  Bayesian Score: 0.971 | | |  | | --- | |  | | G8: 1133499173  14 out of 15 good  Bayesian Score: 0.960 | | |  | | --- | |  | | G9: 2091385504  9 out of 9 good  Bayesian Score: 0.956 | | |  | | --- | |  | | G10: 1639858918  15 out of 17 good  Bayesian Score: 0.920 | |
| |  | | --- | |  | | G11: -4900690  7 out of 7 good  Bayesian Score: 0.912 | | |  | | --- | |  | | G12: -700211968  7 out of 7 good  Bayesian Score: 0.912 | | |  | | --- | |  | | G13: 681865297  7 out of 7 good  Bayesian Score: 0.912 | | |  | | --- | |  | | G14: -244159614  18 out of 21 good  Bayesian Score: 0.911 | | |  | | --- | |  | | G15: -1659633832  6 out of 6 good  Bayesian Score: 0.882 | |
| |  | | --- | |  | | G16: 1997434820  6 out of 6 good  Bayesian Score: 0.882 | | |  | | --- | |  | | G17: -659376053  6 out of 6 good  Bayesian Score: 0.882 | | |  | | --- | |  | | G18: 1798784255  6 out of 6 good  Bayesian Score: 0.882 | | |  | | --- | |  | | G19: -1071406150  6 out of 6 good  Bayesian Score: 0.882 | | |  | | --- | |  | | G20: 1604133265  6 out of 6 good  Bayesian Score: 0.882 | |

Category Bayesian-GABA-A: bad features from ECFP\_6

|  |  |  |  |  |  |  |  |  |  |  |  |  |  |  |
| --- | --- | --- | --- | --- | --- | --- | --- | --- | --- | --- | --- | --- | --- | --- |
| |  | | --- | |  | | B1: 865482986  0 out of 28 good  Bayesian Score: -2.288 | | |  | | --- | |  | | B2: 2023785560  0 out of 22 good  Bayesian Score: -2.074 | | |  | | --- | |  | | B3: 85262808  0 out of 15 good  Bayesian Score: -1.748 | | |  | | --- | |  | | B4: 1994668215  0 out of 14 good  Bayesian Score: -1.691 | | |  | | --- | |  | | B5: 292958156  0 out of 14 good  Bayesian Score: -1.691 | |
| |  | | --- | |  | | B6: 2116709167  0 out of 12 good  Bayesian Score: -1.567 | | |  | | --- | |  | | B7: -1693599735  0 out of 11 good  Bayesian Score: -1.499 | | |  | | --- | |  | | B8: 2101483135  0 out of 11 good  Bayesian Score: -1.499 | | |  | | --- | |  | | B9: -308870089  0 out of 11 good  Bayesian Score: -1.499 | | |  | | --- | |  | | B10: 1961554343  2 out of 39 good  Bayesian Score: -1.491 | |
| |  | | --- | |  | | B11: -1114776580  4 out of 66 good  Bayesian Score: -1.476 | | |  | | --- | |  | | B12: 1588719643  0 out of 10 good  Bayesian Score: -1.426 | | |  | | --- | |  | | B13: -934039951  0 out of 10 good  Bayesian Score: -1.426 | | |  | | --- | |  | | B14: 1544874086  0 out of 10 good  Bayesian Score: -1.426 | | |  | | --- | |  | | B15: 1412053881  4 out of 59 good  Bayesian Score: -1.369 | |
| |  | | --- | |  | | B16: -1101847286  4 out of 59 good  Bayesian Score: -1.369 | | |  | | --- | |  | | B17: -1925046727  3 out of 46 good  Bayesian Score: -1.357 | | |  | | --- | |  | | B18: 218744008  0 out of 9 good  Bayesian Score: -1.347 | | |  | | --- | |  | | B19: -756348342  0 out of 9 good  Bayesian Score: -1.347 | | |  | | --- | |  | | B20: -206566761  0 out of 9 good  Bayesian Score: -1.347 | |
