# Supplementary material for: Identification of Multi-Target Anti-AD Chemical Constituents From Traditional Chinese Medicine Formulae by Integrating Virtual Screening and In Vitro Validation
Source: Front Pharmacol. 2021 Jul 16;12:709607. doi: 10.3389/fphar.2021.709607 (PMC8322649; doi:10.3389/fphar.2021.709607)
Supplement: Supplementary file 3 [file DataSheet1.ZIP › Good and bad fragments of 52 targets/GRIA2.html]

Category Bayesian-AMPA2: good features from ECFP\_6

|  |  |  |  |  |  |  |  |  |  |  |  |  |  |  |
| --- | --- | --- | --- | --- | --- | --- | --- | --- | --- | --- | --- | --- | --- | --- |
| |  | | --- | |  | | G1: 945431654  38 out of 38 good  Bayesian Score: 1.254 | | |  | | --- | |  | | G2: -1299610481  33 out of 33 good  Bayesian Score: 1.245 | | |  | | --- | |  | | G3: 1553727787  33 out of 33 good  Bayesian Score: 1.245 | | |  | | --- | |  | | G4: -1168972498  33 out of 33 good  Bayesian Score: 1.245 | | |  | | --- | |  | | G5: 2106647334  33 out of 33 good  Bayesian Score: 1.245 | |
| |  | | --- | |  | | G6: 83891051  30 out of 30 good  Bayesian Score: 1.238 | | |  | | --- | |  | | G7: -1330434669  33 out of 34 good  Bayesian Score: 1.218 | | |  | | --- | |  | | G8: -449533156  19 out of 19 good  Bayesian Score: 1.194 | | |  | | --- | |  | | G9: -1137453169  19 out of 19 good  Bayesian Score: 1.194 | | |  | | --- | |  | | G10: -1594160374  19 out of 19 good  Bayesian Score: 1.194 | |
| |  | | --- | |  | | G11: 1730212288  19 out of 19 good  Bayesian Score: 1.194 | | |  | | --- | |  | | G12: 1418699737  18 out of 18 good  Bayesian Score: 1.187 | | |  | | --- | |  | | G13: -770520772  17 out of 17 good  Bayesian Score: 1.180 | | |  | | --- | |  | | G14: 772089587  16 out of 16 good  Bayesian Score: 1.173 | | |  | | --- | |  | | G15: -1285951361  16 out of 16 good  Bayesian Score: 1.173 | |
| |  | | --- | |  | | G16: 1563322986  15 out of 15 good  Bayesian Score: 1.164 | | |  | | --- | |  | | G17: -1665306562  15 out of 15 good  Bayesian Score: 1.164 | | |  | | --- | |  | | G18: -1843377874  15 out of 15 good  Bayesian Score: 1.164 | | |  | | --- | |  | | G19: 449616410  15 out of 15 good  Bayesian Score: 1.164 | | |  | | --- | |  | | G20: -1472972030  15 out of 15 good  Bayesian Score: 1.164 | |

Category Bayesian-AMPA2: bad features from ECFP\_6

|  |  |  |  |  |  |  |  |  |  |  |  |  |  |  |
| --- | --- | --- | --- | --- | --- | --- | --- | --- | --- | --- | --- | --- | --- | --- |
| |  | | --- | |  | | B1: 834876373  0 out of 32 good  Bayesian Score: -2.254 | | |  | | --- | |  | | B2: -176494269  0 out of 24 good  Bayesian Score: -2.001 | | |  | | --- | |  | | B3: -1087070950  1 out of 47 good  Bayesian Score: -1.911 | | |  | | --- | |  | | B4: -1925046727  1 out of 47 good  Bayesian Score: -1.911 | | |  | | --- | |  | | B5: 975766354  0 out of 21 good  Bayesian Score: -1.886 | |
| |  | | --- | |  | | B6: 412256466  0 out of 20 good  Bayesian Score: -1.845 | | |  | | --- | |  | | B7: 769925792  0 out of 19 good  Bayesian Score: -1.802 | | |  | | --- | |  | | B8: 657586427  1 out of 40 good  Bayesian Score: -1.763 | | |  | | --- | |  | | B9: -938530932  0 out of 18 good  Bayesian Score: -1.757 | | |  | | --- | |  | | B10: -176483725  0 out of 18 good  Bayesian Score: -1.757 | |
| |  | | --- | |  | | B11: 1337040050  0 out of 18 good  Bayesian Score: -1.757 | | |  | | --- | |  | | B12: -152683720  0 out of 17 good  Bayesian Score: -1.710 | | |  | | --- | |  | | B13: 2085698692  0 out of 17 good  Bayesian Score: -1.710 | | |  | | --- | |  | | B14: -176686665  0 out of 16 good  Bayesian Score: -1.661 | | |  | | --- | |  | | B15: -91640731  0 out of 16 good  Bayesian Score: -1.661 | |
| |  | | --- | |  | | B16: 2147419938  0 out of 15 good  Bayesian Score: -1.609 | | |  | | --- | |  | | B17: 2023785560  0 out of 15 good  Bayesian Score: -1.609 | | |  | | --- | |  | | B18: -845108448  0 out of 15 good  Bayesian Score: -1.609 | | |  | | --- | |  | | B19: 1961554343  0 out of 15 good  Bayesian Score: -1.609 | | |  | | --- | |  | | B20: -81134287  0 out of 15 good  Bayesian Score: -1.609 | |
