# Supplementary material for: Identification of Multi-Target Anti-AD Chemical Constituents From Traditional Chinese Medicine Formulae by Integrating Virtual Screening and In Vitro Validation
Source: Front Pharmacol. 2021 Jul 16;12:709607. doi: 10.3389/fphar.2021.709607 (PMC8322649; doi:10.3389/fphar.2021.709607)
Supplement: Supplementary file 3 [file DataSheet1.ZIP › Good and bad fragments of 52 targets/GRIN1.html]

Category Bayesian-NMDA-Z: good features from ECFP\_6

|  |  |  |  |  |  |  |  |  |  |  |  |  |  |  |
| --- | --- | --- | --- | --- | --- | --- | --- | --- | --- | --- | --- | --- | --- | --- |
| |  | | --- | |  | | G1: -1342994530  161 out of 161 good  Bayesian Score: 1.312 | | |  | | --- | |  | | G2: 1049060082  166 out of 167 good  Bayesian Score: 1.306 | | |  | | --- | |  | | G3: 356989067  81 out of 81 good  Bayesian Score: 1.296 | | |  | | --- | |  | | G4: 1259567539  80 out of 80 good  Bayesian Score: 1.295 | | |  | | --- | |  | | G5: -1968207  70 out of 70 good  Bayesian Score: 1.290 | |
| |  | | --- | |  | | G6: -1963082943  67 out of 67 good  Bayesian Score: 1.289 | | |  | | --- | |  | | G7: 266322205  65 out of 65 good  Bayesian Score: 1.288 | | |  | | --- | |  | | G8: 576992831  62 out of 62 good  Bayesian Score: 1.286 | | |  | | --- | |  | | G9: 1207061102  62 out of 62 good  Bayesian Score: 1.286 | | |  | | --- | |  | | G10: -3028773  55 out of 55 good  Bayesian Score: 1.280 | |
| |  | | --- | |  | | G11: -2566011  55 out of 55 good  Bayesian Score: 1.280 | | |  | | --- | |  | | G12: 1346243876  51 out of 51 good  Bayesian Score: 1.277 | | |  | | --- | |  | | G13: -1363577299  44 out of 44 good  Bayesian Score: 1.269 | | |  | | --- | |  | | G14: -1025141132  44 out of 44 good  Bayesian Score: 1.269 | | |  | | --- | |  | | G15: -43251252  41 out of 41 good  Bayesian Score: 1.265 | |
| |  | | --- | |  | | G16: -862994807  41 out of 41 good  Bayesian Score: 1.265 | | |  | | --- | |  | | G17: -717206821  41 out of 41 good  Bayesian Score: 1.265 | | |  | | --- | |  | | G18: 579349232  86 out of 89 good  Bayesian Score: 1.265 | | |  | | --- | |  | | G19: 577592657  70 out of 72 good  Bayesian Score: 1.264 | | |  | | --- | |  | | G20: -1455053587  40 out of 40 good  Bayesian Score: 1.263 | |

Category Bayesian-NMDA-Z: bad features from ECFP\_6

|  |  |  |  |  |  |  |  |  |  |  |  |  |  |  |
| --- | --- | --- | --- | --- | --- | --- | --- | --- | --- | --- | --- | --- | --- | --- |
| |  | | --- | |  | | B1: 85262808  0 out of 140 good  Bayesian Score: -3.639 | | |  | | --- | |  | | B2: 912478223  0 out of 78 good  Bayesian Score: -3.075 | | |  | | --- | |  | | B3: 975766354  0 out of 65 good  Bayesian Score: -2.902 | | |  | | --- | |  | | B4: -1298560091  0 out of 48 good  Bayesian Score: -2.618 | | |  | | --- | |  | | B5: 1588719643  0 out of 47 good  Bayesian Score: -2.599 | |
| |  | | --- | |  | | B6: 600440273  0 out of 45 good  Bayesian Score: -2.558 | | |  | | --- | |  | | B7: 1652635785  0 out of 44 good  Bayesian Score: -2.538 | | |  | | --- | |  | | B8: 2004682855  0 out of 43 good  Bayesian Score: -2.517 | | |  | | --- | |  | | B9: 2085698692  1 out of 88 good  Bayesian Score: -2.497 | | |  | | --- | |  | | B10: 1010553103  0 out of 42 good  Bayesian Score: -2.495 | |
| |  | | --- | |  | | B11: 454057662  0 out of 41 good  Bayesian Score: -2.473 | | |  | | --- | |  | | B12: 1337040050  1 out of 84 good  Bayesian Score: -2.453 | | |  | | --- | |  | | B13: -1102925512  0 out of 39 good  Bayesian Score: -2.427 | | |  | | --- | |  | | B14: 865857320  1 out of 80 good  Bayesian Score: -2.406 | | |  | | --- | |  | | B15: -1243508568  0 out of 37 good  Bayesian Score: -2.379 | |
| |  | | --- | |  | | B16: -1941087713  0 out of 37 good  Bayesian Score: -2.379 | | |  | | --- | |  | | B17: 57656204  0 out of 36 good  Bayesian Score: -2.354 | | |  | | --- | |  | | B18: 735928936  0 out of 36 good  Bayesian Score: -2.354 | | |  | | --- | |  | | B19: -953984246  0 out of 33 good  Bayesian Score: -2.276 | | |  | | --- | |  | | B20: 1335702447  0 out of 33 good  Bayesian Score: -2.276 | |
