# Supplementary material for: Identification of Multi-Target Anti-AD Chemical Constituents From Traditional Chinese Medicine Formulae by Integrating Virtual Screening and In Vitro Validation
Source: Front Pharmacol. 2021 Jul 16;12:709607. doi: 10.3389/fphar.2021.709607 (PMC8322649; doi:10.3389/fphar.2021.709607)
Supplement: Supplementary file 3 [file DataSheet1.ZIP › Good and bad fragments of 52 targets/GRM2.html]

Category NB\_mglu2\_ECFP6: good features from ECFP\_6

|  |  |  |  |  |  |  |  |  |  |  |  |  |  |  |
| --- | --- | --- | --- | --- | --- | --- | --- | --- | --- | --- | --- | --- | --- | --- |
| |  | | --- | |  | | G1: -1742760313  93 out of 93 good  Bayesian Score: 1.272 | | |  | | --- | |  | | G2: -624057203  93 out of 93 good  Bayesian Score: 1.272 | | |  | | --- | |  | | G3: 924027888  93 out of 93 good  Bayesian Score: 1.272 | | |  | | --- | |  | | G4: -1768979532  93 out of 93 good  Bayesian Score: 1.272 | | |  | | --- | |  | | G5: 2112331916  93 out of 93 good  Bayesian Score: 1.272 | |
| |  | | --- | |  | | G6: 792786551  93 out of 93 good  Bayesian Score: 1.272 | | |  | | --- | |  | | G7: -1052736359  93 out of 93 good  Bayesian Score: 1.272 | | |  | | --- | |  | | G8: 220999462  93 out of 93 good  Bayesian Score: 1.272 | | |  | | --- | |  | | G9: 517360196  91 out of 91 good  Bayesian Score: 1.272 | | |  | | --- | |  | | G10: 721022671  91 out of 91 good  Bayesian Score: 1.272 | |
| |  | | --- | |  | | G11: -139284136  91 out of 91 good  Bayesian Score: 1.272 | | |  | | --- | |  | | G12: 1429461619  72 out of 72 good  Bayesian Score: 1.264 | | |  | | --- | |  | | G13: -1438282506  56 out of 56 good  Bayesian Score: 1.254 | | |  | | --- | |  | | G14: 695384874  56 out of 56 good  Bayesian Score: 1.254 | | |  | | --- | |  | | G15: 1786994679  56 out of 56 good  Bayesian Score: 1.254 | |
| |  | | --- | |  | | G16: -875413610  55 out of 55 good  Bayesian Score: 1.254 | | |  | | --- | |  | | G17: -1129097787  55 out of 55 good  Bayesian Score: 1.254 | | |  | | --- | |  | | G18: -1485300740  55 out of 55 good  Bayesian Score: 1.254 | | |  | | --- | |  | | G19: 1834087673  54 out of 54 good  Bayesian Score: 1.253 | | |  | | --- | |  | | G20: -1176671353  54 out of 54 good  Bayesian Score: 1.253 | |

Category NB\_mglu2\_ECFP6: bad features from ECFP\_6

|  |  |  |  |  |  |  |  |  |  |  |  |  |  |  |
| --- | --- | --- | --- | --- | --- | --- | --- | --- | --- | --- | --- | --- | --- | --- |
| |  | | --- | |  | | B1: 781519895  0 out of 341 good  Bayesian Score: -4.542 | | |  | | --- | |  | | B2: -655344035  0 out of 227 good  Bayesian Score: -4.141 | | |  | | --- | |  | | B3: 85262808  0 out of 120 good  Bayesian Score: -3.517 | | |  | | --- | |  | | B4: -830332112  0 out of 117 good  Bayesian Score: -3.493 | | |  | | --- | |  | | B5: -1087070950  0 out of 114 good  Bayesian Score: -3.468 | |
| |  | | --- | |  | | B6: -1897341097  2 out of 327 good  Bayesian Score: -3.402 | | |  | | --- | |  | | B7: -661766797  0 out of 99 good  Bayesian Score: -3.331 | | |  | | --- | |  | | B8: 1430169877  0 out of 86 good  Bayesian Score: -3.196 | | |  | | --- | |  | | B9: -1236483485  0 out of 83 good  Bayesian Score: -3.162 | | |  | | --- | |  | | B10: -152683720  0 out of 74 good  Bayesian Score: -3.052 | |
| |  | | --- | |  | | B11: 1961554343  0 out of 70 good  Bayesian Score: -2.999 | | |  | | --- | |  | | B12: 1203316083  0 out of 65 good  Bayesian Score: -2.929 | | |  | | --- | |  | | B13: 497523368  0 out of 62 good  Bayesian Score: -2.884 | | |  | | --- | |  | | B14: -2069292548  0 out of 60 good  Bayesian Score: -2.854 | | |  | | --- | |  | | B15: 914325265  2 out of 186 good  Bayesian Score: -2.847 | |
| |  | | --- | |  | | B16: 1427820655  0 out of 59 good  Bayesian Score: -2.838 | | |  | | --- | |  | | B17: 975766354  0 out of 59 good  Bayesian Score: -2.838 | | |  | | --- | |  | | B18: 51876938  1 out of 120 good  Bayesian Score: -2.824 | | |  | | --- | |  | | B19: -1660340418  0 out of 57 good  Bayesian Score: -2.805 | | |  | | --- | |  | | B20: 1280143826  0 out of 56 good  Bayesian Score: -2.789 | |
