# Supplementary material for: Identification of Multi-Target Anti-AD Chemical Constituents From Traditional Chinese Medicine Formulae by Integrating Virtual Screening and In Vitro Validation
Source: Front Pharmacol. 2021 Jul 16;12:709607. doi: 10.3389/fphar.2021.709607 (PMC8322649; doi:10.3389/fphar.2021.709607)
Supplement: Supplementary file 3 [file DataSheet1.ZIP › Good and bad fragments of 52 targets/GRM3.html]

Category NB\_mglu3\_ECFP6: good features from ECFP\_6

|  |  |  |  |  |  |  |  |  |  |  |  |  |  |  |
| --- | --- | --- | --- | --- | --- | --- | --- | --- | --- | --- | --- | --- | --- | --- |
| |  | | --- | |  | | G1: -81842545  44 out of 45 good  Bayesian Score: 1.400 | | |  | | --- | |  | | G2: 2025485523  46 out of 49 good  Bayesian Score: 1.366 | | |  | | --- | |  | | G3: 264903179  22 out of 22 good  Bayesian Score: 1.355 | | |  | | --- | |  | | G4: -1819194629  22 out of 22 good  Bayesian Score: 1.355 | | |  | | --- | |  | | G5: -938893292  22 out of 22 good  Bayesian Score: 1.355 | |
| |  | | --- | |  | | G6: 1405229902  21 out of 21 good  Bayesian Score: 1.349 | | |  | | --- | |  | | G7: -327548242  21 out of 21 good  Bayesian Score: 1.349 | | |  | | --- | |  | | G8: 23341995  21 out of 21 good  Bayesian Score: 1.349 | | |  | | --- | |  | | G9: -1904117427  21 out of 21 good  Bayesian Score: 1.349 | | |  | | --- | |  | | G10: -815564740  21 out of 21 good  Bayesian Score: 1.349 | |
| |  | | --- | |  | | G11: 323956950  21 out of 21 good  Bayesian Score: 1.349 | | |  | | --- | |  | | G12: -1706238096  21 out of 21 good  Bayesian Score: 1.349 | | |  | | --- | |  | | G13: 610431856  21 out of 21 good  Bayesian Score: 1.349 | | |  | | --- | |  | | G14: 308335392  20 out of 20 good  Bayesian Score: 1.342 | | |  | | --- | |  | | G15: -1510935628  20 out of 20 good  Bayesian Score: 1.342 | |
| |  | | --- | |  | | G16: 1210612712  20 out of 20 good  Bayesian Score: 1.342 | | |  | | --- | |  | | G17: -1129936553  20 out of 20 good  Bayesian Score: 1.342 | | |  | | --- | |  | | G18: -519692610  19 out of 19 good  Bayesian Score: 1.335 | | |  | | --- | |  | | G19: -327186626  22 out of 23 good  Bayesian Score: 1.318 | | |  | | --- | |  | | G20: -932469786  21 out of 22 good  Bayesian Score: 1.310 | |

Category NB\_mglu3\_ECFP6: bad features from ECFP\_6

|  |  |  |  |  |  |  |  |  |  |  |  |  |  |  |
| --- | --- | --- | --- | --- | --- | --- | --- | --- | --- | --- | --- | --- | --- | --- |
| |  | | --- | |  | | B1: 781519895  0 out of 82 good  Bayesian Score: -2.965 | | |  | | --- | |  | | B2: 655739385  0 out of 70 good  Bayesian Score: -2.815 | | |  | | --- | |  | | B3: -655344035  0 out of 49 good  Bayesian Score: -2.484 | | |  | | --- | |  | | B4: 834876373  0 out of 26 good  Bayesian Score: -1.921 | | |  | | --- | |  | | B5: -302078100  0 out of 25 good  Bayesian Score: -1.888 | |
| |  | | --- | |  | | B6: 459826767  0 out of 23 good  Bayesian Score: -1.818 | | |  | | --- | |  | | B7: -1925046727  0 out of 22 good  Bayesian Score: -1.781 | | |  | | --- | |  | | B8: -661766797  0 out of 20 good  Bayesian Score: -1.702 | | |  | | --- | |  | | B9: -1897341097  2 out of 57 good  Bayesian Score: -1.525 | | |  | | --- | |  | | B10: -797085356  0 out of 16 good  Bayesian Score: -1.523 | |
| |  | | --- | |  | | B11: 2102150379  0 out of 16 good  Bayesian Score: -1.523 | | |  | | --- | |  | | B12: -2069292548  0 out of 16 good  Bayesian Score: -1.523 | | |  | | --- | |  | | B13: 51876938  0 out of 15 good  Bayesian Score: -1.473 | | |  | | --- | |  | | B14: -1072294614  0 out of 15 good  Bayesian Score: -1.473 | | |  | | --- | |  | | B15: -177935549  0 out of 13 good  Bayesian Score: -1.365 | |
| |  | | --- | |  | | B16: 1280143826  0 out of 13 good  Bayesian Score: -1.365 | | |  | | --- | |  | | B17: -1236483485  0 out of 13 good  Bayesian Score: -1.365 | | |  | | --- | |  | | B18: -1699286547  0 out of 13 good  Bayesian Score: -1.365 | | |  | | --- | |  | | B19: 2023785560  0 out of 13 good  Bayesian Score: -1.365 | | |  | | --- | |  | | B20: 1430169877  0 out of 13 good  Bayesian Score: -1.365 | |
