# Supplementary material for: Identification of Multi-Target Anti-AD Chemical Constituents From Traditional Chinese Medicine Formulae by Integrating Virtual Screening and In Vitro Validation
Source: Front Pharmacol. 2021 Jul 16;12:709607. doi: 10.3389/fphar.2021.709607 (PMC8322649; doi:10.3389/fphar.2021.709607)
Supplement: Supplementary file 3 [file DataSheet1.ZIP › Good and bad fragments of 52 targets/GSK3B.html]

Category Bayesian-gsk3beta: good features from ECFP\_6

|  |  |  |  |  |  |  |  |  |  |  |  |  |  |  |
| --- | --- | --- | --- | --- | --- | --- | --- | --- | --- | --- | --- | --- | --- | --- |
| |  | | --- | |  | | G1: 346118049  157 out of 157 good  Bayesian Score: 1.347 | | |  | | --- | |  | | G2: 57920516  136 out of 136 good  Bayesian Score: 1.345 | | |  | | --- | |  | | G3: -227756037  125 out of 125 good  Bayesian Score: 1.343 | | |  | | --- | |  | | G4: 1201541949  118 out of 118 good  Bayesian Score: 1.342 | | |  | | --- | |  | | G5: 2070853715  117 out of 117 good  Bayesian Score: 1.341 | |
| |  | | --- | |  | | G6: -343041294  117 out of 117 good  Bayesian Score: 1.341 | | |  | | --- | |  | | G7: -1327967530  98 out of 98 good  Bayesian Score: 1.337 | | |  | | --- | |  | | G8: -2026167296  85 out of 85 good  Bayesian Score: 1.332 | | |  | | --- | |  | | G9: -89202465  84 out of 84 good  Bayesian Score: 1.332 | | |  | | --- | |  | | G10: 108130984  84 out of 84 good  Bayesian Score: 1.332 | |
| |  | | --- | |  | | G11: -542877163  79 out of 79 good  Bayesian Score: 1.330 | | |  | | --- | |  | | G12: 1110900508  79 out of 79 good  Bayesian Score: 1.330 | | |  | | --- | |  | | G13: 22829732  77 out of 77 good  Bayesian Score: 1.329 | | |  | | --- | |  | | G14: 1070163437  76 out of 76 good  Bayesian Score: 1.329 | | |  | | --- | |  | | G15: -1430701714  72 out of 72 good  Bayesian Score: 1.327 | |
| |  | | --- | |  | | G16: 1956515344  68 out of 68 good  Bayesian Score: 1.324 | | |  | | --- | |  | | G17: 322707095  62 out of 62 good  Bayesian Score: 1.320 | | |  | | --- | |  | | G18: -1770997745  62 out of 62 good  Bayesian Score: 1.320 | | |  | | --- | |  | | G19: 222827966  80 out of 81 good  Bayesian Score: 1.319 | | |  | | --- | |  | | G20: 1790105651  161 out of 166 good  Bayesian Score: 1.318 | |

Category Bayesian-gsk3beta: bad features from ECFP\_6

|  |  |  |  |  |  |  |  |  |  |  |  |  |  |  |
| --- | --- | --- | --- | --- | --- | --- | --- | --- | --- | --- | --- | --- | --- | --- |
| |  | | --- | |  | | B1: -2091181441  0 out of 142 good  Bayesian Score: -3.617 | | |  | | --- | |  | | B2: 975766354  0 out of 132 good  Bayesian Score: -3.546 | | |  | | --- | |  | | B3: -1672647522  0 out of 118 good  Bayesian Score: -3.438 | | |  | | --- | |  | | B4: 1657836083  0 out of 106 good  Bayesian Score: -3.334 | | |  | | --- | |  | | B5: 292958156  0 out of 105 good  Bayesian Score: -3.325 | |
| |  | | --- | |  | | B6: 1945129186  1 out of 179 good  Bayesian Score: -3.150 | | |  | | --- | |  | | B7: -649348348  0 out of 87 good  Bayesian Score: -3.144 | | |  | | --- | |  | | B8: -1989458582  0 out of 73 good  Bayesian Score: -2.977 | | |  | | --- | |  | | B9: 1526862590  0 out of 72 good  Bayesian Score: -2.964 | | |  | | --- | |  | | B10: -955816473  0 out of 71 good  Bayesian Score: -2.951 | |
| |  | | --- | |  | | B11: 1526392165  0 out of 68 good  Bayesian Score: -2.910 | | |  | | --- | |  | | B12: -1672512695  0 out of 66 good  Bayesian Score: -2.882 | | |  | | --- | |  | | B13: 2088833028  0 out of 66 good  Bayesian Score: -2.882 | | |  | | --- | |  | | B14: -523264395  0 out of 65 good  Bayesian Score: -2.867 | | |  | | --- | |  | | B15: -778131027  0 out of 65 good  Bayesian Score: -2.867 | |
| |  | | --- | |  | | B16: -676041646  0 out of 63 good  Bayesian Score: -2.838 | | |  | | --- | |  | | B17: -661766797  0 out of 62 good  Bayesian Score: -2.823 | | |  | | --- | |  | | B18: 735928936  0 out of 61 good  Bayesian Score: -2.807 | | |  | | --- | |  | | B19: -1693599735  0 out of 60 good  Bayesian Score: -2.792 | | |  | | --- | |  | | B20: 53207596  1 out of 120 good  Bayesian Score: -2.761 | |
