# Supplementary material for: Identification of Multi-Target Anti-AD Chemical Constituents From Traditional Chinese Medicine Formulae by Integrating Virtual Screening and In Vitro Validation
Source: Front Pharmacol. 2021 Jul 16;12:709607. doi: 10.3389/fphar.2021.709607 (PMC8322649; doi:10.3389/fphar.2021.709607)
Supplement: Supplementary file 3 [file DataSheet1.ZIP › Good and bad fragments of 52 targets/HMGCS1.html]

Category NB\_HMG-CoA\_ECFP6: good features from ECFP\_6

|  |  |  |  |  |  |  |  |  |  |  |  |  |  |  |
| --- | --- | --- | --- | --- | --- | --- | --- | --- | --- | --- | --- | --- | --- | --- |
| |  | | --- | |  | | G1: 1422993963  325 out of 325 good  Bayesian Score: 1.150 | | |  | | --- | |  | | G2: 1000942765  260 out of 260 good  Bayesian Score: 1.149 | | |  | | --- | |  | | G3: -1295207602  260 out of 260 good  Bayesian Score: 1.149 | | |  | | --- | |  | | G4: 737332491  252 out of 252 good  Bayesian Score: 1.148 | | |  | | --- | |  | | G5: -95290900  211 out of 211 good  Bayesian Score: 1.147 | |
| |  | | --- | |  | | G6: 1280143826  300 out of 301 good  Bayesian Score: 1.146 | | |  | | --- | |  | | G7: -1887539559  200 out of 200 good  Bayesian Score: 1.146 | | |  | | --- | |  | | G8: -2058379036  199 out of 199 good  Bayesian Score: 1.146 | | |  | | --- | |  | | G9: 131908232  198 out of 198 good  Bayesian Score: 1.146 | | |  | | --- | |  | | G10: -1032675323  198 out of 198 good  Bayesian Score: 1.146 | |
| |  | | --- | |  | | G11: 1022096216  198 out of 198 good  Bayesian Score: 1.146 | | |  | | --- | |  | | G12: -1312559847  325 out of 327 good  Bayesian Score: 1.144 | | |  | | --- | |  | | G13: 1175458476  163 out of 163 good  Bayesian Score: 1.144 | | |  | | --- | |  | | G14: -163723777  149 out of 149 good  Bayesian Score: 1.142 | | |  | | --- | |  | | G15: -1596025254  149 out of 149 good  Bayesian Score: 1.142 | |
| |  | | --- | |  | | G16: -545716875  121 out of 121 good  Bayesian Score: 1.139 | | |  | | --- | |  | | G17: -1310859884  342 out of 346 good  Bayesian Score: 1.139 | | |  | | --- | |  | | G18: -1147098431  119 out of 119 good  Bayesian Score: 1.139 | | |  | | --- | |  | | G19: -1188536061  119 out of 119 good  Bayesian Score: 1.139 | | |  | | --- | |  | | G20: -1687646422  118 out of 118 good  Bayesian Score: 1.139 | |

Category NB\_HMG-CoA\_ECFP6: bad features from ECFP\_6

|  |  |  |  |  |  |  |  |  |  |  |  |  |  |  |
| --- | --- | --- | --- | --- | --- | --- | --- | --- | --- | --- | --- | --- | --- | --- |
| |  | | --- | |  | | B1: -1087070950  0 out of 233 good  Bayesian Score: -4.308 | | |  | | --- | |  | | B2: -938530932  0 out of 132 good  Bayesian Score: -3.750 | | |  | | --- | |  | | B3: 2104376220  0 out of 125 good  Bayesian Score: -3.697 | | |  | | --- | |  | | B4: -659271057  0 out of 125 good  Bayesian Score: -3.697 | | |  | | --- | |  | | B5: 1043790491  0 out of 125 good  Bayesian Score: -3.697 | |
| |  | | --- | |  | | B6: -154530762  1 out of 235 good  Bayesian Score: -3.623 | | |  | | --- | |  | | B7: -215026467  0 out of 112 good  Bayesian Score: -3.590 | | |  | | --- | |  | | B8: -1508366470  0 out of 102 good  Bayesian Score: -3.499 | | |  | | --- | |  | | B9: 2085698692  0 out of 99 good  Bayesian Score: -3.470 | | |  | | --- | |  | | B10: 866343404  0 out of 93 good  Bayesian Score: -3.409 | |
| |  | | --- | |  | | B11: 464808839  0 out of 86 good  Bayesian Score: -3.334 | | |  | | --- | |  | | B12: -845108448  0 out of 81 good  Bayesian Score: -3.276 | | |  | | --- | |  | | B13: 912478223  1 out of 152 good  Bayesian Score: -3.195 | | |  | | --- | |  | | B14: -152683720  1 out of 147 good  Bayesian Score: -3.162 | | |  | | --- | |  | | B15: 2122741631  0 out of 71 good  Bayesian Score: -3.150 | |
| |  | | --- | |  | | B16: 1203316083  0 out of 69 good  Bayesian Score: -3.122 | | |  | | --- | |  | | B17: -1832102709  0 out of 68 good  Bayesian Score: -3.108 | | |  | | --- | |  | | B18: -570915357  0 out of 64 good  Bayesian Score: -3.051 | | |  | | --- | |  | | B19: 1335108269  0 out of 64 good  Bayesian Score: -3.051 | | |  | | --- | |  | | B20: 1951894094  0 out of 63 good  Bayesian Score: -3.036 | |
