# Supplementary material for: Identification of Multi-Target Anti-AD Chemical Constituents From Traditional Chinese Medicine Formulae by Integrating Virtual Screening and In Vitro Validation
Source: Front Pharmacol. 2021 Jul 16;12:709607. doi: 10.3389/fphar.2021.709607 (PMC8322649; doi:10.3389/fphar.2021.709607)
Supplement: Supplementary file 3 [file DataSheet1.ZIP › Good and bad fragments of 52 targets/HRH3.html]

Category Bayesian-H3: good features from ECFP\_6

|  |  |  |  |  |  |  |  |  |  |  |  |  |  |  |
| --- | --- | --- | --- | --- | --- | --- | --- | --- | --- | --- | --- | --- | --- | --- |
| |  | | --- | |  | | G1: 1976330679  1625 out of 1625 good  Bayesian Score: 1.280 | | |  | | --- | |  | | G2: -244159614  1550 out of 1550 good  Bayesian Score: 1.280 | | |  | | --- | |  | | G3: -1794005192  885 out of 885 good  Bayesian Score: 1.279 | | |  | | --- | |  | | G4: 1133499173  856 out of 856 good  Bayesian Score: 1.279 | | |  | | --- | |  | | G5: -1340901104  598 out of 598 good  Bayesian Score: 1.278 | |
| |  | | --- | |  | | G6: 368983122  540 out of 540 good  Bayesian Score: 1.277 | | |  | | --- | |  | | G7: 408863435  513 out of 513 good  Bayesian Score: 1.277 | | |  | | --- | |  | | G8: 486597464  513 out of 513 good  Bayesian Score: 1.277 | | |  | | --- | |  | | G9: 1424833548  513 out of 513 good  Bayesian Score: 1.277 | | |  | | --- | |  | | G10: -750301151  478 out of 478 good  Bayesian Score: 1.277 | |
| |  | | --- | |  | | G11: 1905301167  478 out of 478 good  Bayesian Score: 1.277 | | |  | | --- | |  | | G12: 1712497868  478 out of 478 good  Bayesian Score: 1.277 | | |  | | --- | |  | | G13: -976851658  457 out of 457 good  Bayesian Score: 1.276 | | |  | | --- | |  | | G14: 1753956000  429 out of 429 good  Bayesian Score: 1.276 | | |  | | --- | |  | | G15: 681865297  417 out of 417 good  Bayesian Score: 1.276 | |
| |  | | --- | |  | | G16: -1242491717  403 out of 403 good  Bayesian Score: 1.276 | | |  | | --- | |  | | G17: -953730098  513 out of 514 good  Bayesian Score: 1.275 | | |  | | --- | |  | | G18: 725072930  367 out of 367 good  Bayesian Score: 1.275 | | |  | | --- | |  | | G19: 100251162  367 out of 367 good  Bayesian Score: 1.275 | | |  | | --- | |  | | G20: 548998319  354 out of 354 good  Bayesian Score: 1.275 | |

Category Bayesian-H3: bad features from ECFP\_6

|  |  |  |  |  |  |  |  |  |  |  |  |  |  |  |
| --- | --- | --- | --- | --- | --- | --- | --- | --- | --- | --- | --- | --- | --- | --- |
| |  | | --- | |  | | B1: 2025485523  0 out of 247 good  Bayesian Score: -4.242 | | |  | | --- | |  | | B2: 1335702447  0 out of 236 good  Bayesian Score: -4.197 | | |  | | --- | |  | | B3: 544048674  0 out of 232 good  Bayesian Score: -4.180 | | |  | | --- | |  | | B4: 1814278164  0 out of 206 good  Bayesian Score: -4.063 | | |  | | --- | |  | | B5: 1182722866  0 out of 188 good  Bayesian Score: -3.973 | |
| |  | | --- | |  | | B6: -2041399277  0 out of 176 good  Bayesian Score: -3.909 | | |  | | --- | |  | | B7: -175376949  0 out of 156 good  Bayesian Score: -3.791 | | |  | | --- | |  | | B8: -955816473  0 out of 142 good  Bayesian Score: -3.699 | | |  | | --- | |  | | B9: -1660913849  0 out of 135 good  Bayesian Score: -3.650 | | |  | | --- | |  | | B10: 1573945311  0 out of 133 good  Bayesian Score: -3.635 | |
| |  | | --- | |  | | B11: -851770808  0 out of 130 good  Bayesian Score: -3.613 | | |  | | --- | |  | | B12: -1236714312  0 out of 129 good  Bayesian Score: -3.605 | | |  | | --- | |  | | B13: -1968207  0 out of 117 good  Bayesian Score: -3.511 | | |  | | --- | |  | | B14: 455570479  0 out of 115 good  Bayesian Score: -3.494 | | |  | | --- | |  | | B15: 1429461619  0 out of 104 good  Bayesian Score: -3.396 | |
| |  | | --- | |  | | B16: 1717462980  1 out of 210 good  Bayesian Score: -3.389 | | |  | | --- | |  | | B17: -1268218162  0 out of 102 good  Bayesian Score: -3.378 | | |  | | --- | |  | | B18: -1566003658  0 out of 100 good  Bayesian Score: -3.359 | | |  | | --- | |  | | B19: -716992514  0 out of 100 good  Bayesian Score: -3.359 | | |  | | --- | |  | | B20: -1832102709  3 out of 408 good  Bayesian Score: -3.352 | |
