# Supplementary material for: Identification of Multi-Target Anti-AD Chemical Constituents From Traditional Chinese Medicine Formulae by Integrating Virtual Screening and In Vitro Validation
Source: Front Pharmacol. 2021 Jul 16;12:709607. doi: 10.3389/fphar.2021.709607 (PMC8322649; doi:10.3389/fphar.2021.709607)
Supplement: Supplementary file 3 [file DataSheet1.ZIP › Good and bad fragments of 52 targets/HSP90AA1.html]

Category NB\_HSP90\_ECFP6: good features from ECFP\_6

|  |  |  |  |  |  |  |  |  |  |  |  |  |  |  |
| --- | --- | --- | --- | --- | --- | --- | --- | --- | --- | --- | --- | --- | --- | --- |
| |  | | --- | |  | | G1: 1934453675  225 out of 226 good  Bayesian Score: 1.114 | | |  | | --- | |  | | G2: 408200288  131 out of 131 good  Bayesian Score: 1.112 | | |  | | --- | |  | | G3: 1795265959  127 out of 127 good  Bayesian Score: 1.111 | | |  | | --- | |  | | G4: -830156479  110 out of 110 good  Bayesian Score: 1.109 | | |  | | --- | |  | | G5: -199218813  110 out of 110 good  Bayesian Score: 1.109 | |
| |  | | --- | |  | | G6: -465639554  105 out of 105 good  Bayesian Score: 1.108 | | |  | | --- | |  | | G7: -715112939  105 out of 105 good  Bayesian Score: 1.108 | | |  | | --- | |  | | G8: -1881636446  102 out of 102 good  Bayesian Score: 1.107 | | |  | | --- | |  | | G9: 72638804  95 out of 95 good  Bayesian Score: 1.106 | | |  | | --- | |  | | G10: -545322638  86 out of 86 good  Bayesian Score: 1.104 | |
| |  | | --- | |  | | G11: -1072397558  86 out of 86 good  Bayesian Score: 1.104 | | |  | | --- | |  | | G12: -965745644  86 out of 86 good  Bayesian Score: 1.104 | | |  | | --- | |  | | G13: -1203125863  86 out of 86 good  Bayesian Score: 1.104 | | |  | | --- | |  | | G14: 1366387863  85 out of 85 good  Bayesian Score: 1.104 | | |  | | --- | |  | | G15: -2082700411  85 out of 85 good  Bayesian Score: 1.104 | |
| |  | | --- | |  | | G16: 1229038054  85 out of 85 good  Bayesian Score: 1.104 | | |  | | --- | |  | | G17: -1371104878  84 out of 84 good  Bayesian Score: 1.103 | | |  | | --- | |  | | G18: -364489768  84 out of 84 good  Bayesian Score: 1.103 | | |  | | --- | |  | | G19: 202031734  84 out of 84 good  Bayesian Score: 1.103 | | |  | | --- | |  | | G20: -923534040  84 out of 84 good  Bayesian Score: 1.103 | |

Category NB\_HSP90\_ECFP6: bad features from ECFP\_6

|  |  |  |  |  |  |  |  |  |  |  |  |  |  |  |
| --- | --- | --- | --- | --- | --- | --- | --- | --- | --- | --- | --- | --- | --- | --- |
| |  | | --- | |  | | B1: 1043790491  0 out of 161 good  Bayesian Score: -3.973 | | |  | | --- | |  | | B2: -659271057  0 out of 160 good  Bayesian Score: -3.967 | | |  | | --- | |  | | B3: 2104376220  0 out of 158 good  Bayesian Score: -3.954 | | |  | | --- | |  | | B4: -215026467  0 out of 140 good  Bayesian Score: -3.836 | | |  | | --- | |  | | B5: 781519895  1 out of 192 good  Bayesian Score: -3.453 | |
| |  | | --- | |  | | B6: 1182722866  0 out of 69 good  Bayesian Score: -3.150 | | |  | | --- | |  | | B7: -176846085  0 out of 59 good  Bayesian Score: -3.001 | | |  | | --- | |  | | B8: 544048674  0 out of 57 good  Bayesian Score: -2.968 | | |  | | --- | |  | | B9: 1814278164  0 out of 56 good  Bayesian Score: -2.951 | | |  | | --- | |  | | B10: -179073144  0 out of 55 good  Bayesian Score: -2.934 | |
| |  | | --- | |  | | B11: 1526862590  0 out of 55 good  Bayesian Score: -2.934 | | |  | | --- | |  | | B12: -1740465052  0 out of 54 good  Bayesian Score: -2.917 | | |  | | --- | |  | | B13: -224638920  0 out of 53 good  Bayesian Score: -2.899 | | |  | | --- | |  | | B14: -175882072  1 out of 109 good  Bayesian Score: -2.899 | | |  | | --- | |  | | B15: -1394206246  0 out of 52 good  Bayesian Score: -2.881 | |
| |  | | --- | |  | | B16: -1508366470  1 out of 104 good  Bayesian Score: -2.853 | | |  | | --- | |  | | B17: 1595541658  0 out of 50 good  Bayesian Score: -2.844 | | |  | | --- | |  | | B18: -1832102709  1 out of 102 good  Bayesian Score: -2.834 | | |  | | --- | |  | | B19: -1956535100  0 out of 49 good  Bayesian Score: -2.825 | | |  | | --- | |  | | B20: -1742225957  0 out of 48 good  Bayesian Score: -2.806 | |
