# Supplementary material for: Identification of Multi-Target Anti-AD Chemical Constituents From Traditional Chinese Medicine Formulae by Integrating Virtual Screening and In Vitro Validation
Source: Front Pharmacol. 2021 Jul 16;12:709607. doi: 10.3389/fphar.2021.709607 (PMC8322649; doi:10.3389/fphar.2021.709607)
Supplement: Supplementary file 3 [file DataSheet1.ZIP › Good and bad fragments of 52 targets/HTR1A.html]

Category Bayesian-5HT1A: good features from ECFP\_6

|  |  |  |  |  |  |  |  |  |  |  |  |  |  |  |
| --- | --- | --- | --- | --- | --- | --- | --- | --- | --- | --- | --- | --- | --- | --- |
| |  | | --- | |  | | G1: 341532899  17 out of 17 good  Bayesian Score: 1.104 | | |  | | --- | |  | | G2: -1794005192  17 out of 17 good  Bayesian Score: 1.104 | | |  | | --- | |  | | G3: 368983122  16 out of 16 good  Bayesian Score: 1.097 | | |  | | --- | |  | | G4: 1425765496  15 out of 15 good  Bayesian Score: 1.090 | | |  | | --- | |  | | G5: 1133499173  19 out of 20 good  Bayesian Score: 1.073 | |
| |  | | --- | |  | | G6: 681865297  13 out of 13 good  Bayesian Score: 1.071 | | |  | | --- | |  | | G7: 1297652335  12 out of 12 good  Bayesian Score: 1.060 | | |  | | --- | |  | | G8: 1951894094  17 out of 18 good  Bayesian Score: 1.057 | | |  | | --- | |  | | G9: 1295541189  11 out of 11 good  Bayesian Score: 1.047 | | |  | | --- | |  | | G10: -1263245884  11 out of 11 good  Bayesian Score: 1.047 | |
| |  | | --- | |  | | G11: -976472993  11 out of 11 good  Bayesian Score: 1.047 | | |  | | --- | |  | | G12: -244159614  25 out of 28 good  Bayesian Score: 1.041 | | |  | | --- | |  | | G13: -1232032529  9 out of 9 good  Bayesian Score: 1.014 | | |  | | --- | |  | | G14: -822911877  9 out of 9 good  Bayesian Score: 1.014 | | |  | | --- | |  | | G15: -888859362  9 out of 9 good  Bayesian Score: 1.014 | |
| |  | | --- | |  | | G16: -1231423474  9 out of 9 good  Bayesian Score: 1.014 | | |  | | --- | |  | | G17: -509950643  9 out of 9 good  Bayesian Score: 1.014 | | |  | | --- | |  | | G18: 1396250933  9 out of 9 good  Bayesian Score: 1.014 | | |  | | --- | |  | | G19: -1568683570  9 out of 9 good  Bayesian Score: 1.014 | | |  | | --- | |  | | G20: 161643267  9 out of 9 good  Bayesian Score: 1.014 | |

Category Bayesian-5HT1A: bad features from ECFP\_6

|  |  |  |  |  |  |  |  |  |  |  |  |  |  |  |
| --- | --- | --- | --- | --- | --- | --- | --- | --- | --- | --- | --- | --- | --- | --- |
| |  | | --- | |  | | B1: -1910270391  0 out of 48 good  Bayesian Score: -2.709 | | |  | | --- | |  | | B2: 864518973  0 out of 34 good  Bayesian Score: -2.392 | | |  | | --- | |  | | B3: 657586427  0 out of 17 good  Bayesian Score: -1.786 | | |  | | --- | |  | | B4: 459826767  0 out of 16 good  Bayesian Score: -1.736 | | |  | | --- | |  | | B5: -302078100  0 out of 16 good  Bayesian Score: -1.736 | |
| |  | | --- | |  | | B6: -176846085  0 out of 15 good  Bayesian Score: -1.683 | | |  | | --- | |  | | B7: -395008465  0 out of 14 good  Bayesian Score: -1.627 | | |  | | --- | |  | | B8: 1961554343  1 out of 31 good  Bayesian Score: -1.615 | | |  | | --- | |  | | B9: 1335691903  0 out of 13 good  Bayesian Score: -1.568 | | |  | | --- | |  | | B10: -1072294614  0 out of 13 good  Bayesian Score: -1.568 | |
| |  | | --- | |  | | B11: 865857320  0 out of 13 good  Bayesian Score: -1.568 | | |  | | --- | |  | | B12: -1236483485  0 out of 12 good  Bayesian Score: -1.505 | | |  | | --- | |  | | B13: 834876373  0 out of 12 good  Bayesian Score: -1.505 | | |  | | --- | |  | | B14: 1994668215  0 out of 11 good  Bayesian Score: -1.438 | | |  | | --- | |  | | B15: 975766354  0 out of 11 good  Bayesian Score: -1.438 | |
| |  | | --- | |  | | B16: 2116455019  0 out of 11 good  Bayesian Score: -1.438 | | |  | | --- | |  | | B17: -817402818  1 out of 24 good  Bayesian Score: -1.387 | | |  | | --- | |  | | B18: 865379614  0 out of 10 good  Bayesian Score: -1.366 | | |  | | --- | |  | | B19: 218744008  0 out of 9 good  Bayesian Score: -1.289 | | |  | | --- | |  | | B20: 663943468  0 out of 9 good  Bayesian Score: -1.289 | |
