# Supplementary material for: Identification of Multi-Target Anti-AD Chemical Constituents From Traditional Chinese Medicine Formulae by Integrating Virtual Screening and In Vitro Validation
Source: Front Pharmacol. 2021 Jul 16;12:709607. doi: 10.3389/fphar.2021.709607 (PMC8322649; doi:10.3389/fphar.2021.709607)
Supplement: Supplementary file 3 [file DataSheet1.ZIP › Good and bad fragments of 52 targets/HTR2A.html]

Category NB\_5HT\_2A\_ECFP6: good features from ECFP\_6

|  |  |  |  |  |  |  |  |  |  |  |  |  |  |  |
| --- | --- | --- | --- | --- | --- | --- | --- | --- | --- | --- | --- | --- | --- | --- |
| |  | | --- | |  | | G1: 1506089003  672 out of 672 good  Bayesian Score: 1.253 | | |  | | --- | |  | | G2: -1681415204  389 out of 389 good  Bayesian Score: 1.250 | | |  | | --- | |  | | G3: 46302398  348 out of 348 good  Bayesian Score: 1.250 | | |  | | --- | |  | | G4: 103053196  336 out of 336 good  Bayesian Score: 1.249 | | |  | | --- | |  | | G5: 1171496488  268 out of 268 good  Bayesian Score: 1.248 | |
| |  | | --- | |  | | G6: -1444246813  350 out of 351 good  Bayesian Score: 1.247 | | |  | | --- | |  | | G7: 1209826035  350 out of 351 good  Bayesian Score: 1.247 | | |  | | --- | |  | | G8: -622423479  350 out of 351 good  Bayesian Score: 1.247 | | |  | | --- | |  | | G9: 2028312840  248 out of 248 good  Bayesian Score: 1.247 | | |  | | --- | |  | | G10: 102005830  227 out of 227 good  Bayesian Score: 1.246 | |
| |  | | --- | |  | | G11: -1153677524  220 out of 220 good  Bayesian Score: 1.246 | | |  | | --- | |  | | G12: -1684509107  206 out of 206 good  Bayesian Score: 1.245 | | |  | | --- | |  | | G13: 203547503  186 out of 186 good  Bayesian Score: 1.243 | | |  | | --- | |  | | G14: 1281235771  172 out of 172 good  Bayesian Score: 1.242 | | |  | | --- | |  | | G15: -809543529  581 out of 587 good  Bayesian Score: 1.242 | |
| |  | | --- | |  | | G16: 1508840879  170 out of 170 good  Bayesian Score: 1.242 | | |  | | --- | |  | | G17: -1542628425  170 out of 170 good  Bayesian Score: 1.242 | | |  | | --- | |  | | G18: -1045601281  361 out of 364 good  Bayesian Score: 1.242 | | |  | | --- | |  | | G19: 102821781  164 out of 164 good  Bayesian Score: 1.242 | | |  | | --- | |  | | G20: 1490305890  161 out of 161 good  Bayesian Score: 1.241 | |

Category NB\_5HT\_2A\_ECFP6: bad features from ECFP\_6

|  |  |  |  |  |  |  |  |  |  |  |  |  |  |  |
| --- | --- | --- | --- | --- | --- | --- | --- | --- | --- | --- | --- | --- | --- | --- |
| |  | | --- | |  | | B1: 1961554343  0 out of 1891 good  Bayesian Score: -6.290 | | |  | | --- | |  | | B2: 1976330679  0 out of 811 good  Bayesian Score: -5.446 | | |  | | --- | |  | | B3: -591526139  0 out of 742 good  Bayesian Score: -5.357 | | |  | | --- | |  | | B4: -244159614  0 out of 659 good  Bayesian Score: -5.239 | | |  | | --- | |  | | B5: 1994668215  0 out of 654 good  Bayesian Score: -5.232 | |
| |  | | --- | |  | | B6: -666326105  0 out of 616 good  Bayesian Score: -5.172 | | |  | | --- | |  | | B7: 2116455019  0 out of 579 good  Bayesian Score: -5.111 | | |  | | --- | |  | | B8: 1151284196  0 out of 397 good  Bayesian Score: -4.736 | | |  | | --- | |  | | B9: 233520344  0 out of 395 good  Bayesian Score: -4.731 | | |  | | --- | |  | | B10: 1331561287  0 out of 356 good  Bayesian Score: -4.628 | |
| |  | | --- | |  | | B11: 2116709167  0 out of 344 good  Bayesian Score: -4.594 | | |  | | --- | |  | | B12: -788112909  0 out of 339 good  Bayesian Score: -4.580 | | |  | | --- | |  | | B13: 860114273  0 out of 258 good  Bayesian Score: -4.310 | | |  | | --- | |  | | B14: -242828956  0 out of 226 good  Bayesian Score: -4.179 | | |  | | --- | |  | | B15: -2127980805  0 out of 210 good  Bayesian Score: -4.107 | |
| |  | | --- | |  | | B16: -2024536851  0 out of 209 good  Bayesian Score: -4.102 | | |  | | --- | |  | | B17: 1133499173  0 out of 207 good  Bayesian Score: -4.093 | | |  | | --- | |  | | B18: 1640603662  0 out of 206 good  Bayesian Score: -4.088 | | |  | | --- | |  | | B19: -342718945  0 out of 204 good  Bayesian Score: -4.078 | | |  | | --- | |  | | B20: -2130275420  0 out of 199 good  Bayesian Score: -4.054 | |
