# Supplementary material for: Identification of Multi-Target Anti-AD Chemical Constituents From Traditional Chinese Medicine Formulae by Integrating Virtual Screening and In Vitro Validation
Source: Front Pharmacol. 2021 Jul 16;12:709607. doi: 10.3389/fphar.2021.709607 (PMC8322649; doi:10.3389/fphar.2021.709607)
Supplement: Supplementary file 3 [file DataSheet1.ZIP › Good and bad fragments of 52 targets/HTR3A.html]

Category Bayesian-5HT3A: good features from ECFP\_6

|  |  |  |  |  |  |  |  |  |  |  |  |  |  |  |
| --- | --- | --- | --- | --- | --- | --- | --- | --- | --- | --- | --- | --- | --- | --- |
| |  | | --- | |  | | G1: 341532899  86 out of 87 good  Bayesian Score: 1.146 | | |  | | --- | |  | | G2: 368983122  55 out of 55 good  Bayesian Score: 1.143 | | |  | | --- | |  | | G3: 1960253356  54 out of 54 good  Bayesian Score: 1.142 | | |  | | --- | |  | | G4: 1425765496  45 out of 45 good  Bayesian Score: 1.134 | | |  | | --- | |  | | G5: -2041916939  44 out of 44 good  Bayesian Score: 1.133 | |
| |  | | --- | |  | | G6: -1342305140  40 out of 40 good  Bayesian Score: 1.129 | | |  | | --- | |  | | G7: 7323192  39 out of 39 good  Bayesian Score: 1.127 | | |  | | --- | |  | | G8: -309990580  39 out of 39 good  Bayesian Score: 1.127 | | |  | | --- | |  | | G9: 759220570  39 out of 39 good  Bayesian Score: 1.127 | | |  | | --- | |  | | G10: -916174044  39 out of 39 good  Bayesian Score: 1.127 | |
| |  | | --- | |  | | G11: 1686169969  39 out of 39 good  Bayesian Score: 1.127 | | |  | | --- | |  | | G12: 681865297  39 out of 39 good  Bayesian Score: 1.127 | | |  | | --- | |  | | G13: 209058652  39 out of 39 good  Bayesian Score: 1.127 | | |  | | --- | |  | | G14: 1878124254  39 out of 39 good  Bayesian Score: 1.127 | | |  | | --- | |  | | G15: 2069906330  56 out of 57 good  Bayesian Score: 1.127 | |
| |  | | --- | |  | | G16: -73930652  38 out of 38 good  Bayesian Score: 1.126 | | |  | | --- | |  | | G17: -1106229527  38 out of 38 good  Bayesian Score: 1.126 | | |  | | --- | |  | | G18: 2043810848  38 out of 38 good  Bayesian Score: 1.126 | | |  | | --- | |  | | G19: -85903119  37 out of 37 good  Bayesian Score: 1.124 | | |  | | --- | |  | | G20: -637151581  37 out of 37 good  Bayesian Score: 1.124 | |

Category Bayesian-5HT3A: bad features from ECFP\_6

|  |  |  |  |  |  |  |  |  |  |  |  |  |  |  |
| --- | --- | --- | --- | --- | --- | --- | --- | --- | --- | --- | --- | --- | --- | --- |
| |  | | --- | |  | | B1: 1994668215  0 out of 86 good  Bayesian Score: -3.309 | | |  | | --- | |  | | B2: 859433814  0 out of 81 good  Bayesian Score: -3.252 | | |  | | --- | |  | | B3: 2116455019  0 out of 67 good  Bayesian Score: -3.070 | | |  | | --- | |  | | B4: 2116709167  0 out of 51 good  Bayesian Score: -2.812 | | |  | | --- | |  | | B5: 413587124  0 out of 51 good  Bayesian Score: -2.812 | |
| |  | | --- | |  | | B6: -395008465  0 out of 47 good  Bayesian Score: -2.735 | | |  | | --- | |  | | B7: -591526139  1 out of 95 good  Bayesian Score: -2.712 | | |  | | --- | |  | | B8: -788112909  0 out of 45 good  Bayesian Score: -2.694 | | |  | | --- | |  | | B9: 2023785560  0 out of 44 good  Bayesian Score: -2.673 | | |  | | --- | |  | | B10: 1996740348  0 out of 41 good  Bayesian Score: -2.608 | |
| |  | | --- | |  | | B11: -1114776580  1 out of 81 good  Bayesian Score: -2.558 | | |  | | --- | |  | | B12: -1101847286  1 out of 73 good  Bayesian Score: -2.459 | | |  | | --- | |  | | B13: 1412053881  1 out of 73 good  Bayesian Score: -2.459 | | |  | | --- | |  | | B14: 590183026  0 out of 34 good  Bayesian Score: -2.436 | | |  | | --- | |  | | B15: 908605940  0 out of 34 good  Bayesian Score: -2.436 | |
| |  | | --- | |  | | B16: 975766354  0 out of 31 good  Bayesian Score: -2.352 | | |  | | --- | |  | | B17: -949992060  0 out of 29 good  Bayesian Score: -2.292 | | |  | | --- | |  | | B18: 1003761175  0 out of 29 good  Bayesian Score: -2.292 | | |  | | --- | |  | | B19: 300955665  0 out of 28 good  Bayesian Score: -2.260 | | |  | | --- | |  | | B20: 866401773  0 out of 27 good  Bayesian Score: -2.228 | |
