# Supplementary material for: Identification of Multi-Target Anti-AD Chemical Constituents From Traditional Chinese Medicine Formulae by Integrating Virtual Screening and In Vitro Validation
Source: Front Pharmacol. 2021 Jul 16;12:709607. doi: 10.3389/fphar.2021.709607 (PMC8322649; doi:10.3389/fphar.2021.709607)
Supplement: Supplementary file 3 [file DataSheet1.ZIP › Good and bad fragments of 52 targets/HTR4.html]

Category Bayesian-5HT4: good features from ECFP\_6

|  |  |  |  |  |  |  |  |  |  |  |  |  |  |  |
| --- | --- | --- | --- | --- | --- | --- | --- | --- | --- | --- | --- | --- | --- | --- |
| |  | | --- | |  | | G1: 2131972380  201 out of 201 good  Bayesian Score: 1.248 | | |  | | --- | |  | | G2: 779992229  166 out of 166 good  Bayesian Score: 1.245 | | |  | | --- | |  | | G3: -1542796220  120 out of 120 good  Bayesian Score: 1.239 | | |  | | --- | |  | | G4: -1172475893  113 out of 113 good  Bayesian Score: 1.238 | | |  | | --- | |  | | G5: -217108455  113 out of 113 good  Bayesian Score: 1.238 | |
| |  | | --- | |  | | G6: 1145675601  112 out of 112 good  Bayesian Score: 1.238 | | |  | | --- | |  | | G7: -1965046861  112 out of 112 good  Bayesian Score: 1.238 | | |  | | --- | |  | | G8: -1152104088  109 out of 109 good  Bayesian Score: 1.237 | | |  | | --- | |  | | G9: -2024533470  106 out of 106 good  Bayesian Score: 1.237 | | |  | | --- | |  | | G10: -467650405  106 out of 106 good  Bayesian Score: 1.237 | |
| |  | | --- | |  | | G11: -922873439  105 out of 105 good  Bayesian Score: 1.237 | | |  | | --- | |  | | G12: -809543529  92 out of 92 good  Bayesian Score: 1.233 | | |  | | --- | |  | | G13: 710197868  80 out of 80 good  Bayesian Score: 1.229 | | |  | | --- | |  | | G14: 1257604650  77 out of 77 good  Bayesian Score: 1.228 | | |  | | --- | |  | | G15: 252169264  70 out of 70 good  Bayesian Score: 1.225 | |
| |  | | --- | |  | | G16: -1756940577  85 out of 86 good  Bayesian Score: 1.220 | | |  | | --- | |  | | G17: 1618209862  60 out of 60 good  Bayesian Score: 1.220 | | |  | | --- | |  | | G18: 1473857266  60 out of 60 good  Bayesian Score: 1.220 | | |  | | --- | |  | | G19: 1296119159  60 out of 60 good  Bayesian Score: 1.220 | | |  | | --- | |  | | G20: 2106008255  60 out of 60 good  Bayesian Score: 1.220 | |

Category Bayesian-5HT4: bad features from ECFP\_6

|  |  |  |  |  |  |  |  |  |  |  |  |  |  |  |
| --- | --- | --- | --- | --- | --- | --- | --- | --- | --- | --- | --- | --- | --- | --- |
| |  | | --- | |  | | B1: 1961554343  0 out of 394 good  Bayesian Score: -4.725 | | |  | | --- | |  | | B2: -591526139  0 out of 141 good  Bayesian Score: -3.713 | | |  | | --- | |  | | B3: 1994668215  0 out of 131 good  Bayesian Score: -3.642 | | |  | | --- | |  | | B4: 2116455019  0 out of 126 good  Bayesian Score: -3.604 | | |  | | --- | |  | | B5: -176846085  0 out of 121 good  Bayesian Score: -3.564 | |
| |  | | --- | |  | | B6: 85262808  0 out of 112 good  Bayesian Score: -3.489 | | |  | | --- | |  | | B7: -666326105  0 out of 111 good  Bayesian Score: -3.481 | | |  | | --- | |  | | B8: -830332112  0 out of 107 good  Bayesian Score: -3.445 | | |  | | --- | |  | | B9: -1416572622  0 out of 104 good  Bayesian Score: -3.418 | | |  | | --- | |  | | B10: -179515162  0 out of 84 good  Bayesian Score: -3.212 | |
| |  | | --- | |  | | B11: -788112909  0 out of 82 good  Bayesian Score: -3.189 | | |  | | --- | |  | | B12: 975766354  0 out of 75 good  Bayesian Score: -3.103 | | |  | | --- | |  | | B13: 1745066357  0 out of 71 good  Bayesian Score: -3.051 | | |  | | --- | |  | | B14: 2116709167  0 out of 71 good  Bayesian Score: -3.051 | | |  | | --- | |  | | B15: -181568884  1 out of 140 good  Bayesian Score: -3.013 | |
| |  | | --- | |  | | B16: 233520344  0 out of 68 good  Bayesian Score: -3.010 | | |  | | --- | |  | | B17: -395008465  0 out of 63 good  Bayesian Score: -2.937 | | |  | | --- | |  | | B18: -175882072  0 out of 61 good  Bayesian Score: -2.907 | | |  | | --- | |  | | B19: 1427820655  0 out of 61 good  Bayesian Score: -2.907 | | |  | | --- | |  | | B20: -210573707  0 out of 60 good  Bayesian Score: -2.891 | |
