# Supplementary material for: Identification of Multi-Target Anti-AD Chemical Constituents From Traditional Chinese Medicine Formulae by Integrating Virtual Screening and In Vitro Validation
Source: Front Pharmacol. 2021 Jul 16;12:709607. doi: 10.3389/fphar.2021.709607 (PMC8322649; doi:10.3389/fphar.2021.709607)
Supplement: Supplementary file 3 [file DataSheet1.ZIP › Good and bad fragments of 52 targets/HTR6.html]

Category Bayesian-5HT6: good features from ECFP\_6

|  |  |  |  |  |  |  |  |  |  |  |  |  |  |  |
| --- | --- | --- | --- | --- | --- | --- | --- | --- | --- | --- | --- | --- | --- | --- |
| |  | | --- | |  | | G1: 861316614  24 out of 24 good  Bayesian Score: 1.197 | | |  | | --- | |  | | G2: -1794005192  19 out of 19 good  Bayesian Score: 1.173 | | |  | | --- | |  | | G3: 368983122  17 out of 17 good  Bayesian Score: 1.160 | | |  | | --- | |  | | G4: -1072889414  15 out of 15 good  Bayesian Score: 1.144 | | |  | | --- | |  | | G5: 577592657  15 out of 15 good  Bayesian Score: 1.144 | |
| |  | | --- | |  | | G6: 1639316612  14 out of 14 good  Bayesian Score: 1.135 | | |  | | --- | |  | | G7: 1099224616  14 out of 14 good  Bayesian Score: 1.135 | | |  | | --- | |  | | G8: 1466334513  14 out of 14 good  Bayesian Score: 1.135 | | |  | | --- | |  | | G9: -1137341530  14 out of 14 good  Bayesian Score: 1.135 | | |  | | --- | |  | | G10: 2082225029  13 out of 13 good  Bayesian Score: 1.124 | |
| |  | | --- | |  | | G11: -1342305140  13 out of 13 good  Bayesian Score: 1.124 | | |  | | --- | |  | | G12: 681865297  12 out of 12 good  Bayesian Score: 1.112 | | |  | | --- | |  | | G13: 341532899  12 out of 12 good  Bayesian Score: 1.112 | | |  | | --- | |  | | G14: -244159614  36 out of 41 good  Bayesian Score: 1.110 | | |  | | --- | |  | | G15: -4900690  11 out of 11 good  Bayesian Score: 1.098 | |
| |  | | --- | |  | | G16: -1792133435  15 out of 16 good  Bayesian Score: 1.092 | | |  | | --- | |  | | G17: 303802483  10 out of 10 good  Bayesian Score: 1.082 | | |  | | --- | |  | | G18: -1788978402  10 out of 10 good  Bayesian Score: 1.082 | | |  | | --- | |  | | G19: 1445685567  14 out of 15 good  Bayesian Score: 1.080 | | |  | | --- | |  | | G20: 1151284196  14 out of 15 good  Bayesian Score: 1.080 | |

Category Bayesian-5HT6: bad features from ECFP\_6

|  |  |  |  |  |  |  |  |  |  |  |  |  |  |  |
| --- | --- | --- | --- | --- | --- | --- | --- | --- | --- | --- | --- | --- | --- | --- |
| |  | | --- | |  | | B1: 1961554343  0 out of 57 good  Bayesian Score: -2.807 | | |  | | --- | |  | | B2: -181568884  0 out of 23 good  Bayesian Score: -1.985 | | |  | | --- | |  | | B3: 1994668215  0 out of 23 good  Bayesian Score: -1.985 | | |  | | --- | |  | | B4: -1236483485  0 out of 20 good  Bayesian Score: -1.866 | | |  | | --- | |  | | B5: -1087070950  0 out of 19 good  Bayesian Score: -1.822 | |
| |  | | --- | |  | | B6: -175882072  0 out of 19 good  Bayesian Score: -1.822 | | |  | | --- | |  | | B7: 865857320  0 out of 17 good  Bayesian Score: -1.730 | | |  | | --- | |  | | B8: 864518973  1 out of 36 good  Bayesian Score: -1.689 | | |  | | --- | |  | | B9: 662850656  0 out of 16 good  Bayesian Score: -1.680 | | |  | | --- | |  | | B10: -264471301  0 out of 15 good  Bayesian Score: -1.628 | |
| |  | | --- | |  | | B11: -1426923364  0 out of 15 good  Bayesian Score: -1.628 | | |  | | --- | |  | | B12: 2116709167  0 out of 14 good  Bayesian Score: -1.573 | | |  | | --- | |  | | B13: -219423964  0 out of 14 good  Bayesian Score: -1.573 | | |  | | --- | |  | | B14: 2116455019  0 out of 14 good  Bayesian Score: -1.573 | | |  | | --- | |  | | B15: 213246618  0 out of 14 good  Bayesian Score: -1.573 | |
| |  | | --- | |  | | B16: 1845080228  0 out of 14 good  Bayesian Score: -1.573 | | |  | | --- | |  | | B17: -302078100  2 out of 49 good  Bayesian Score: -1.567 | | |  | | --- | |  | | B18: 1334250623  0 out of 13 good  Bayesian Score: -1.515 | | |  | | --- | |  | | B19: 218744008  0 out of 13 good  Bayesian Score: -1.515 | | |  | | --- | |  | | B20: -788112909  0 out of 12 good  Bayesian Score: -1.453 | |
