# Supplementary material for: Identification of Multi-Target Anti-AD Chemical Constituents From Traditional Chinese Medicine Formulae by Integrating Virtual Screening and In Vitro Validation
Source: Front Pharmacol. 2021 Jul 16;12:709607. doi: 10.3389/fphar.2021.709607 (PMC8322649; doi:10.3389/fphar.2021.709607)
Supplement: Supplementary file 3 [file DataSheet1.ZIP › Good and bad fragments of 52 targets/IDE.html]

Category NB\_insulin-degrading-ECFP6: good features from ECFP\_6

|  |  |  |  |  |  |  |  |  |  |  |  |  |  |  |
| --- | --- | --- | --- | --- | --- | --- | --- | --- | --- | --- | --- | --- | --- | --- |
| |  | | --- | |  | | G1: 1945129186  24 out of 26 good  Bayesian Score: 1.065 | | |  | | --- | |  | | G2: -175021654  11 out of 11 good  Bayesian Score: 1.044 | | |  | | --- | |  | | G3: 2081597585  8 out of 8 good  Bayesian Score: 0.990 | | |  | | --- | |  | | G4: 1182722866  15 out of 17 good  Bayesian Score: 0.984 | | |  | | --- | |  | | G5: 305957013  11 out of 12 good  Bayesian Score: 0.977 | |
| |  | | --- | |  | | G6: -2010297388  7 out of 7 good  Bayesian Score: 0.964 | | |  | | --- | |  | | G7: -1116476241  7 out of 7 good  Bayesian Score: 0.964 | | |  | | --- | |  | | G8: 1951894094  16 out of 19 good  Bayesian Score: 0.951 | | |  | | --- | |  | | G9: -962771238  9 out of 10 good  Bayesian Score: 0.934 | | |  | | --- | |  | | G10: -552688510  6 out of 6 good  Bayesian Score: 0.931 | |
| |  | | --- | |  | | G11: 1099224616  6 out of 6 good  Bayesian Score: 0.931 | | |  | | --- | |  | | G12: -2137232509  6 out of 6 good  Bayesian Score: 0.931 | | |  | | --- | |  | | G13: 668436193  6 out of 6 good  Bayesian Score: 0.931 | | |  | | --- | |  | | G14: 342267039  6 out of 6 good  Bayesian Score: 0.931 | | |  | | --- | |  | | G15: 1021725999  6 out of 6 good  Bayesian Score: 0.931 | |
| |  | | --- | |  | | G16: -1410276375  6 out of 6 good  Bayesian Score: 0.931 | | |  | | --- | |  | | G17: -176483725  14 out of 17 good  Bayesian Score: 0.920 | | |  | | --- | |  | | G18: 1451403962  8 out of 9 good  Bayesian Score: 0.906 | | |  | | --- | |  | | G19: 1378243846  8 out of 9 good  Bayesian Score: 0.906 | | |  | | --- | |  | | G20: 1313185693  5 out of 5 good  Bayesian Score: 0.890 | |

Category NB\_insulin-degrading-ECFP6: bad features from ECFP\_6

|  |  |  |  |  |  |  |  |  |  |  |  |  |  |  |
| --- | --- | --- | --- | --- | --- | --- | --- | --- | --- | --- | --- | --- | --- | --- |
| |  | | --- | |  | | B1: -655344035  0 out of 33 good  Bayesian Score: -2.367 | | |  | | --- | |  | | B2: 781519895  1 out of 68 good  Bayesian Score: -2.348 | | |  | | --- | |  | | B3: -1910270391  2 out of 65 good  Bayesian Score: -1.899 | | |  | | --- | |  | | B4: -661766797  0 out of 19 good  Bayesian Score: -1.882 | | |  | | --- | |  | | B5: 865857320  0 out of 17 good  Bayesian Score: -1.788 | |
| |  | | --- | |  | | B6: 412256466  0 out of 12 good  Bayesian Score: -1.508 | | |  | | --- | |  | | B7: 2023785560  0 out of 12 good  Bayesian Score: -1.508 | | |  | | --- | |  | | B8: -1085223908  1 out of 27 good  Bayesian Score: -1.494 | | |  | | --- | |  | | B9: 864518973  2 out of 40 good  Bayesian Score: -1.444 | | |  | | --- | |  | | B10: -649348348  0 out of 11 good  Bayesian Score: -1.440 | |
| |  | | --- | |  | | B11: 978469901  0 out of 11 good  Bayesian Score: -1.440 | | |  | | --- | |  | | B12: -176846085  0 out of 11 good  Bayesian Score: -1.440 | | |  | | --- | |  | | B13: 1961554343  0 out of 11 good  Bayesian Score: -1.440 | | |  | | --- | |  | | B14: -1331450522  3 out of 52 good  Bayesian Score: -1.401 | | |  | | --- | |  | | B15: -2097294478  0 out of 10 good  Bayesian Score: -1.369 | |
| |  | | --- | |  | | B16: -264471301  0 out of 10 good  Bayesian Score: -1.369 | | |  | | --- | |  | | B17: -178525456  0 out of 10 good  Bayesian Score: -1.369 | | |  | | --- | |  | | B18: -206566761  0 out of 10 good  Bayesian Score: -1.369 | | |  | | --- | |  | | B19: 1336304100  0 out of 10 good  Bayesian Score: -1.369 | | |  | | --- | |  | | B20: 717474525  0 out of 9 good  Bayesian Score: -1.291 | |
