# Supplementary material for: Identification of Multi-Target Anti-AD Chemical Constituents From Traditional Chinese Medicine Formulae by Integrating Virtual Screening and In Vitro Validation
Source: Front Pharmacol. 2021 Jul 16;12:709607. doi: 10.3389/fphar.2021.709607 (PMC8322649; doi:10.3389/fphar.2021.709607)
Supplement: Supplementary file 3 [file DataSheet1.ZIP › Good and bad fragments of 52 targets/IKBKB.html]

Category NB\_IKK-beta\_ECFP6: good features from ECFP\_6

|  |  |  |  |  |  |  |  |  |  |  |  |  |  |  |
| --- | --- | --- | --- | --- | --- | --- | --- | --- | --- | --- | --- | --- | --- | --- |
| |  | | --- | |  | | G1: -588083609  89 out of 89 good  Bayesian Score: 1.265 | | |  | | --- | |  | | G2: 957530031  73 out of 73 good  Bayesian Score: 1.259 | | |  | | --- | |  | | G3: 222827966  71 out of 71 good  Bayesian Score: 1.258 | | |  | | --- | |  | | G4: -917126572  67 out of 67 good  Bayesian Score: 1.256 | | |  | | --- | |  | | G5: 705711270  67 out of 67 good  Bayesian Score: 1.256 | |
| |  | | --- | |  | | G6: 2089812293  67 out of 67 good  Bayesian Score: 1.256 | | |  | | --- | |  | | G7: 1784625497  67 out of 67 good  Bayesian Score: 1.256 | | |  | | --- | |  | | G8: 269823168  67 out of 67 good  Bayesian Score: 1.256 | | |  | | --- | |  | | G9: 1024846080  67 out of 67 good  Bayesian Score: 1.256 | | |  | | --- | |  | | G10: -222021117  67 out of 67 good  Bayesian Score: 1.256 | |
| |  | | --- | |  | | G11: 267158307  67 out of 67 good  Bayesian Score: 1.256 | | |  | | --- | |  | | G12: 507069893  67 out of 67 good  Bayesian Score: 1.256 | | |  | | --- | |  | | G13: -1499541126  67 out of 67 good  Bayesian Score: 1.256 | | |  | | --- | |  | | G14: 2000600244  67 out of 67 good  Bayesian Score: 1.256 | | |  | | --- | |  | | G15: -1674510805  67 out of 67 good  Bayesian Score: 1.256 | |
| |  | | --- | |  | | G16: 731342042  67 out of 67 good  Bayesian Score: 1.256 | | |  | | --- | |  | | G17: 876780229  67 out of 67 good  Bayesian Score: 1.256 | | |  | | --- | |  | | G18: 128876060  67 out of 67 good  Bayesian Score: 1.256 | | |  | | --- | |  | | G19: -1101783080  67 out of 67 good  Bayesian Score: 1.256 | | |  | | --- | |  | | G20: 1121449130  67 out of 67 good  Bayesian Score: 1.256 | |

Category NB\_IKK-beta\_ECFP6: bad features from ECFP\_6

|  |  |  |  |  |  |  |  |  |  |  |  |  |  |  |
| --- | --- | --- | --- | --- | --- | --- | --- | --- | --- | --- | --- | --- | --- | --- |
| |  | | --- | |  | | B1: 1961554343  0 out of 357 good  Bayesian Score: -4.594 | | |  | | --- | |  | | B2: -1699286547  0 out of 260 good  Bayesian Score: -4.280 | | |  | | --- | |  | | B3: 1976330679  0 out of 224 good  Bayesian Score: -4.134 | | |  | | --- | |  | | B4: -302078100  0 out of 208 good  Bayesian Score: -4.061 | | |  | | --- | |  | | B5: 459826767  0 out of 206 good  Bayesian Score: -4.051 | |
| |  | | --- | |  | | B6: -244159614  0 out of 205 good  Bayesian Score: -4.047 | | |  | | --- | |  | | B7: -1087070950  0 out of 170 good  Bayesian Score: -3.863 | | |  | | --- | |  | | B8: 1335833675  0 out of 157 good  Bayesian Score: -3.785 | | |  | | --- | |  | | B9: -177935549  0 out of 135 good  Bayesian Score: -3.638 | | |  | | --- | |  | | B10: 1133499173  0 out of 131 good  Bayesian Score: -3.609 | |
| |  | | --- | |  | | B11: 53207596  0 out of 114 good  Bayesian Score: -3.474 | | |  | | --- | |  | | B12: 2116455019  0 out of 104 good  Bayesian Score: -3.385 | | |  | | --- | |  | | B13: -669219631  0 out of 103 good  Bayesian Score: -3.375 | | |  | | --- | |  | | B14: 1994668215  0 out of 102 good  Bayesian Score: -3.366 | | |  | | --- | |  | | B15: -206566761  0 out of 100 good  Bayesian Score: -3.347 | |
| |  | | --- | |  | | B16: -661766797  0 out of 100 good  Bayesian Score: -3.347 | | |  | | --- | |  | | B17: -666326105  0 out of 94 good  Bayesian Score: -3.287 | | |  | | --- | |  | | B18: -175882072  1 out of 189 good  Bayesian Score: -3.274 | | |  | | --- | |  | | B19: -749801696  0 out of 92 good  Bayesian Score: -3.267 | | |  | | --- | |  | | B20: -1416572622  0 out of 91 good  Bayesian Score: -3.256 | |
