# Supplementary material for: Identification of Multi-Target Anti-AD Chemical Constituents From Traditional Chinese Medicine Formulae by Integrating Virtual Screening and In Vitro Validation
Source: Front Pharmacol. 2021 Jul 16;12:709607. doi: 10.3389/fphar.2021.709607 (PMC8322649; doi:10.3389/fphar.2021.709607)
Supplement: Supplementary file 3 [file DataSheet1.ZIP › Good and bad fragments of 52 targets/MAOB.html]

Category Bayesian-maoB: good features from ECFP\_6

|  |  |  |  |  |  |  |  |  |  |  |  |  |  |  |
| --- | --- | --- | --- | --- | --- | --- | --- | --- | --- | --- | --- | --- | --- | --- |
| |  | | --- | |  | | G1: -1523675806  175 out of 175 good  Bayesian Score: 1.248 | | |  | | --- | |  | | G2: 863134739  167 out of 168 good  Bayesian Score: 1.241 | | |  | | --- | |  | | G3: -1728184685  115 out of 115 good  Bayesian Score: 1.240 | | |  | | --- | |  | | G4: -1434856875  102 out of 102 good  Bayesian Score: 1.238 | | |  | | --- | |  | | G5: 1219344933  137 out of 138 good  Bayesian Score: 1.237 | |
| |  | | --- | |  | | G6: -779557588  91 out of 91 good  Bayesian Score: 1.235 | | |  | | --- | |  | | G7: 1134440630  86 out of 86 good  Bayesian Score: 1.233 | | |  | | --- | |  | | G8: -605827787  81 out of 81 good  Bayesian Score: 1.231 | | |  | | --- | |  | | G9: 1166083871  79 out of 79 good  Bayesian Score: 1.231 | | |  | | --- | |  | | G10: -1845486197  75 out of 75 good  Bayesian Score: 1.229 | |
| |  | | --- | |  | | G11: 659771533  74 out of 74 good  Bayesian Score: 1.229 | | |  | | --- | |  | | G12: -77266174  70 out of 70 good  Bayesian Score: 1.227 | | |  | | --- | |  | | G13: -1846894271  70 out of 70 good  Bayesian Score: 1.227 | | |  | | --- | |  | | G14: 1980994924  70 out of 70 good  Bayesian Score: 1.227 | | |  | | --- | |  | | G15: 1685917737  69 out of 69 good  Bayesian Score: 1.226 | |
| |  | | --- | |  | | G16: -1562071895  67 out of 67 good  Bayesian Score: 1.225 | | |  | | --- | |  | | G17: -1897063316  92 out of 93 good  Bayesian Score: 1.225 | | |  | | --- | |  | | G18: -1663911879  52 out of 52 good  Bayesian Score: 1.215 | | |  | | --- | |  | | G19: 1990642688  49 out of 49 good  Bayesian Score: 1.212 | | |  | | --- | |  | | G20: 538141125  47 out of 47 good  Bayesian Score: 1.210 | |

Category Bayesian-maoB: bad features from ECFP\_6

|  |  |  |  |  |  |  |  |  |  |  |  |  |  |  |
| --- | --- | --- | --- | --- | --- | --- | --- | --- | --- | --- | --- | --- | --- | --- |
| |  | | --- | |  | | B1: 657586427  0 out of 246 good  Bayesian Score: -4.258 | | |  | | --- | |  | | B2: -801490360  0 out of 143 good  Bayesian Score: -3.725 | | |  | | --- | |  | | B3: 859433814  0 out of 140 good  Bayesian Score: -3.705 | | |  | | --- | |  | | B4: 412256466  0 out of 102 good  Bayesian Score: -3.397 | | |  | | --- | |  | | B5: 85262808  1 out of 140 good  Bayesian Score: -3.012 | |
| |  | | --- | |  | | B6: 581459960  0 out of 61 good  Bayesian Score: -2.905 | | |  | | --- | |  | | B7: 413587124  0 out of 51 good  Bayesian Score: -2.737 | | |  | | --- | |  | | B8: -857882738  0 out of 46 good  Bayesian Score: -2.641 | | |  | | --- | |  | | B9: -1087070950  1 out of 91 good  Bayesian Score: -2.594 | | |  | | --- | |  | | B10: 865482986  2 out of 138 good  Bayesian Score: -2.592 | |
| |  | | --- | |  | | B11: -1939757055  0 out of 41 good  Bayesian Score: -2.534 | | |  | | --- | |  | | B12: -1832102709  0 out of 41 good  Bayesian Score: -2.534 | | |  | | --- | |  | | B13: -147588444  0 out of 39 good  Bayesian Score: -2.488 | | |  | | --- | |  | | B14: 1335340087  0 out of 38 good  Bayesian Score: -2.465 | | |  | | --- | |  | | B15: 1997522062  0 out of 37 good  Bayesian Score: -2.440 | |
| |  | | --- | |  | | B16: 294288814  0 out of 37 good  Bayesian Score: -2.440 | | |  | | --- | |  | | B17: 407900312  0 out of 36 good  Bayesian Score: -2.415 | | |  | | --- | |  | | B18: 292958156  0 out of 36 good  Bayesian Score: -2.415 | | |  | | --- | |  | | B19: -676555381  0 out of 35 good  Bayesian Score: -2.390 | | |  | | --- | |  | | B20: -90310073  0 out of 35 good  Bayesian Score: -2.390 | |
