# Supplementary material for: Identification of Multi-Target Anti-AD Chemical Constituents From Traditional Chinese Medicine Formulae by Integrating Virtual Screening and In Vitro Validation
Source: Front Pharmacol. 2021 Jul 16;12:709607. doi: 10.3389/fphar.2021.709607 (PMC8322649; doi:10.3389/fphar.2021.709607)
Supplement: Supplementary file 3 [file DataSheet1.ZIP › Good and bad fragments of 52 targets/MAPK10.html]

Category NB\_jnk3\_ECFP6: good features from ECFP\_6

|  |  |  |  |  |  |  |  |  |  |  |  |  |  |  |
| --- | --- | --- | --- | --- | --- | --- | --- | --- | --- | --- | --- | --- | --- | --- |
| |  | | --- | |  | | G1: -2080905499  56 out of 56 good  Bayesian Score: 1.160 | | |  | | --- | |  | | G2: -349594035  56 out of 56 good  Bayesian Score: 1.160 | | |  | | --- | |  | | G3: -460071663  56 out of 56 good  Bayesian Score: 1.160 | | |  | | --- | |  | | G4: -1522147051  50 out of 50 good  Bayesian Score: 1.156 | | |  | | --- | |  | | G5: -878292224  47 out of 47 good  Bayesian Score: 1.153 | |
| |  | | --- | |  | | G6: 36753181  47 out of 47 good  Bayesian Score: 1.153 | | |  | | --- | |  | | G7: 2100436185  47 out of 47 good  Bayesian Score: 1.153 | | |  | | --- | |  | | G8: -1539238197  46 out of 46 good  Bayesian Score: 1.152 | | |  | | --- | |  | | G9: 1876705192  46 out of 46 good  Bayesian Score: 1.152 | | |  | | --- | |  | | G10: 222827966  61 out of 62 good  Bayesian Score: 1.148 | |
| |  | | --- | |  | | G11: 1610682404  61 out of 62 good  Bayesian Score: 1.148 | | |  | | --- | |  | | G12: 500629622  41 out of 41 good  Bayesian Score: 1.146 | | |  | | --- | |  | | G13: -1757142404  41 out of 41 good  Bayesian Score: 1.146 | | |  | | --- | |  | | G14: 1942595874  56 out of 57 good  Bayesian Score: 1.144 | | |  | | --- | |  | | G15: -1369305853  38 out of 38 good  Bayesian Score: 1.142 | |
| |  | | --- | |  | | G16: 389909193  53 out of 54 good  Bayesian Score: 1.141 | | |  | | --- | |  | | G17: 1758604519  36 out of 36 good  Bayesian Score: 1.139 | | |  | | --- | |  | | G18: -983424957  35 out of 35 good  Bayesian Score: 1.138 | | |  | | --- | |  | | G19: 1241070950  35 out of 35 good  Bayesian Score: 1.138 | | |  | | --- | |  | | G20: -275948275  35 out of 35 good  Bayesian Score: 1.138 | |

Category NB\_jnk3\_ECFP6: bad features from ECFP\_6

|  |  |  |  |  |  |  |  |  |  |  |  |  |  |  |
| --- | --- | --- | --- | --- | --- | --- | --- | --- | --- | --- | --- | --- | --- | --- |
| |  | | --- | |  | | B1: 912478223  0 out of 135 good  Bayesian Score: -3.729 | | |  | | --- | |  | | B2: 859433814  0 out of 97 good  Bayesian Score: -3.408 | | |  | | --- | |  | | B3: -1508366470  0 out of 90 good  Bayesian Score: -3.336 | | |  | | --- | |  | | B4: -801490360  0 out of 73 good  Bayesian Score: -3.135 | | |  | | --- | |  | | B5: -1832102709  0 out of 66 good  Bayesian Score: -3.039 | |
| |  | | --- | |  | | B6: 2122741631  0 out of 63 good  Bayesian Score: -2.994 | | |  | | --- | |  | | B7: 413587124  0 out of 51 good  Bayesian Score: -2.795 | | |  | | --- | |  | | B8: -86640970  0 out of 47 good  Bayesian Score: -2.718 | | |  | | --- | |  | | B9: -830332112  3 out of 177 good  Bayesian Score: -2.608 | | |  | | --- | |  | | B10: -859213396  0 out of 37 good  Bayesian Score: -2.497 | |
| |  | | --- | |  | | B11: 910652331  0 out of 37 good  Bayesian Score: -2.497 | | |  | | --- | |  | | B12: 544048674  0 out of 34 good  Bayesian Score: -2.419 | | |  | | --- | |  | | B13: -141329148  0 out of 33 good  Bayesian Score: -2.392 | | |  | | --- | |  | | B14: -175882072  1 out of 69 good  Bayesian Score: -2.388 | | |  | | --- | |  | | B15: -1912117433  0 out of 32 good  Bayesian Score: -2.364 | |
| |  | | --- | |  | | B16: 1595541658  0 out of 32 good  Bayesian Score: -2.364 | | |  | | --- | |  | | B17: 1814278164  0 out of 32 good  Bayesian Score: -2.364 | | |  | | --- | |  | | B18: -1394206246  0 out of 32 good  Bayesian Score: -2.364 | | |  | | --- | |  | | B19: 407900312  0 out of 32 good  Bayesian Score: -2.364 | | |  | | --- | |  | | B20: 1126948380  0 out of 31 good  Bayesian Score: -2.336 | |
