# Supplementary material for: Identification of Multi-Target Anti-AD Chemical Constituents From Traditional Chinese Medicine Formulae by Integrating Virtual Screening and In Vitro Validation
Source: Front Pharmacol. 2021 Jul 16;12:709607. doi: 10.3389/fphar.2021.709607 (PMC8322649; doi:10.3389/fphar.2021.709607)
Supplement: Supplementary file 3 [file DataSheet1.ZIP › Good and bad fragments of 52 targets/MAPK8.html]

Category NB\_jnk1\_ECFP6: good features from ECFP\_6

|  |  |  |  |  |  |  |  |  |  |  |  |  |  |  |
| --- | --- | --- | --- | --- | --- | --- | --- | --- | --- | --- | --- | --- | --- | --- |
| |  | | --- | |  | | G1: 2090033707  185 out of 185 good  Bayesian Score: 1.247 | | |  | | --- | |  | | G2: 279501884  165 out of 165 good  Bayesian Score: 1.245 | | |  | | --- | |  | | G3: 1761417782  165 out of 165 good  Bayesian Score: 1.245 | | |  | | --- | |  | | G4: -1930759180  164 out of 164 good  Bayesian Score: 1.245 | | |  | | --- | |  | | G5: 986972475  130 out of 130 good  Bayesian Score: 1.241 | |
| |  | | --- | |  | | G6: -35817915  105 out of 105 good  Bayesian Score: 1.237 | | |  | | --- | |  | | G7: -2080854136  104 out of 104 good  Bayesian Score: 1.236 | | |  | | --- | |  | | G8: -2026167296  103 out of 103 good  Bayesian Score: 1.236 | | |  | | --- | |  | | G9: 1260004158  102 out of 102 good  Bayesian Score: 1.236 | | |  | | --- | |  | | G10: 221937490  102 out of 102 good  Bayesian Score: 1.236 | |
| |  | | --- | |  | | G11: -958660261  181 out of 183 good  Bayesian Score: 1.236 | | |  | | --- | |  | | G12: 760856458  99 out of 99 good  Bayesian Score: 1.235 | | |  | | --- | |  | | G13: -1872981399  99 out of 99 good  Bayesian Score: 1.235 | | |  | | --- | |  | | G14: -217278258  99 out of 99 good  Bayesian Score: 1.235 | | |  | | --- | |  | | G15: -805031269  99 out of 99 good  Bayesian Score: 1.235 | |
| |  | | --- | |  | | G16: -1774317357  99 out of 99 good  Bayesian Score: 1.235 | | |  | | --- | |  | | G17: 1662045702  96 out of 96 good  Bayesian Score: 1.235 | | |  | | --- | |  | | G18: 43417144  95 out of 95 good  Bayesian Score: 1.234 | | |  | | --- | |  | | G19: -1721966501  92 out of 92 good  Bayesian Score: 1.233 | | |  | | --- | |  | | G20: -178055763  92 out of 92 good  Bayesian Score: 1.233 | |

Category NB\_jnk1\_ECFP6: bad features from ECFP\_6

|  |  |  |  |  |  |  |  |  |  |  |  |  |  |  |
| --- | --- | --- | --- | --- | --- | --- | --- | --- | --- | --- | --- | --- | --- | --- |
| |  | | --- | |  | | B1: 1961554343  0 out of 280 good  Bayesian Score: -4.387 | | |  | | --- | |  | | B2: -1699286547  0 out of 273 good  Bayesian Score: -4.362 | | |  | | --- | |  | | B3: 1976330679  0 out of 174 good  Bayesian Score: -3.919 | | |  | | --- | |  | | B4: -176846085  0 out of 173 good  Bayesian Score: -3.913 | | |  | | --- | |  | | B5: -244159614  0 out of 157 good  Bayesian Score: -3.818 | |
| |  | | --- | |  | | B6: 1335833675  0 out of 156 good  Bayesian Score: -3.812 | | |  | | --- | |  | | B7: 912478223  0 out of 155 good  Bayesian Score: -3.806 | | |  | | --- | |  | | B8: 1588719643  0 out of 149 good  Bayesian Score: -3.767 | | |  | | --- | |  | | B9: 53207596  0 out of 149 good  Bayesian Score: -3.767 | | |  | | --- | |  | | B10: 1336304100  0 out of 147 good  Bayesian Score: -3.754 | |
| |  | | --- | |  | | B11: 2085698692  0 out of 145 good  Bayesian Score: -3.741 | | |  | | --- | |  | | B12: -395008465  0 out of 121 good  Bayesian Score: -3.564 | | |  | | --- | |  | | B13: -1693599735  0 out of 114 good  Bayesian Score: -3.506 | | |  | | --- | |  | | B14: -175882072  1 out of 224 good  Bayesian Score: -3.474 | | |  | | --- | |  | | B15: -655344035  0 out of 110 good  Bayesian Score: -3.472 | |
| |  | | --- | |  | | B16: -845108448  0 out of 106 good  Bayesian Score: -3.436 | | |  | | --- | |  | | B17: -1832102709  0 out of 103 good  Bayesian Score: -3.408 | | |  | | --- | |  | | B18: 1298725959  0 out of 99 good  Bayesian Score: -3.370 | | |  | | --- | |  | | B19: -661766797  0 out of 87 good  Bayesian Score: -3.245 | | |  | | --- | |  | | B20: 305957013  0 out of 85 good  Bayesian Score: -3.223 | |
