# Supplementary material for: Identification of Multi-Target Anti-AD Chemical Constituents From Traditional Chinese Medicine Formulae by Integrating Virtual Screening and In Vitro Validation
Source: Front Pharmacol. 2021 Jul 16;12:709607. doi: 10.3389/fphar.2021.709607 (PMC8322649; doi:10.3389/fphar.2021.709607)
Supplement: Supplementary file 3 [file DataSheet1.ZIP › Good and bad fragments of 52 targets/MAPK9.html]

Category NB\_jnk2\_ECFP6: good features from ECFP\_6

|  |  |  |  |  |  |  |  |  |  |  |  |  |  |  |
| --- | --- | --- | --- | --- | --- | --- | --- | --- | --- | --- | --- | --- | --- | --- |
| |  | | --- | |  | | G1: -1033843597  75 out of 75 good  Bayesian Score: 1.258 | | |  | | --- | |  | | G2: 2141444578  53 out of 53 good  Bayesian Score: 1.244 | | |  | | --- | |  | | G3: -1963229762  53 out of 53 good  Bayesian Score: 1.244 | | |  | | --- | |  | | G4: 101151132  53 out of 53 good  Bayesian Score: 1.244 | | |  | | --- | |  | | G5: 389909193  52 out of 52 good  Bayesian Score: 1.243 | |
| |  | | --- | |  | | G6: -1107160118  51 out of 51 good  Bayesian Score: 1.242 | | |  | | --- | |  | | G7: 107498356  50 out of 50 good  Bayesian Score: 1.241 | | |  | | --- | |  | | G8: -745681290  50 out of 50 good  Bayesian Score: 1.241 | | |  | | --- | |  | | G9: 1753564049  50 out of 50 good  Bayesian Score: 1.241 | | |  | | --- | |  | | G10: 1369792062  50 out of 50 good  Bayesian Score: 1.241 | |
| |  | | --- | |  | | G11: -365586470  50 out of 50 good  Bayesian Score: 1.241 | | |  | | --- | |  | | G12: -1278135913  49 out of 49 good  Bayesian Score: 1.240 | | |  | | --- | |  | | G13: -1067417619  46 out of 46 good  Bayesian Score: 1.237 | | |  | | --- | |  | | G14: 183726608  45 out of 45 good  Bayesian Score: 1.236 | | |  | | --- | |  | | G15: -213377878  45 out of 45 good  Bayesian Score: 1.236 | |
| |  | | --- | |  | | G16: -869396132  61 out of 62 good  Bayesian Score: 1.235 | | |  | | --- | |  | | G17: -1665306562  77 out of 79 good  Bayesian Score: 1.234 | | |  | | --- | |  | | G18: 1610682404  59 out of 60 good  Bayesian Score: 1.233 | | |  | | --- | |  | | G19: 1260004158  42 out of 42 good  Bayesian Score: 1.232 | | |  | | --- | |  | | G20: 221937490  42 out of 42 good  Bayesian Score: 1.232 | |

Category NB\_jnk2\_ECFP6: bad features from ECFP\_6

|  |  |  |  |  |  |  |  |  |  |  |  |  |  |  |
| --- | --- | --- | --- | --- | --- | --- | --- | --- | --- | --- | --- | --- | --- | --- |
| |  | | --- | |  | | B1: 1961554343  0 out of 131 good  Bayesian Score: -3.611 | | |  | | --- | |  | | B2: -1087070950  0 out of 119 good  Bayesian Score: -3.517 | | |  | | --- | |  | | B3: 2085698692  0 out of 88 good  Bayesian Score: -3.226 | | |  | | --- | |  | | B4: 912478223  0 out of 77 good  Bayesian Score: -3.098 | | |  | | --- | |  | | B5: 53207596  0 out of 73 good  Bayesian Score: -3.047 | |
| |  | | --- | |  | | B6: -801490360  0 out of 65 good  Bayesian Score: -2.937 | | |  | | --- | |  | | B7: 859433814  0 out of 62 good  Bayesian Score: -2.892 | | |  | | --- | |  | | B8: -1699286547  1 out of 122 good  Bayesian Score: -2.848 | | |  | | --- | |  | | B9: 1427820655  0 out of 57 good  Bayesian Score: -2.813 | | |  | | --- | |  | | B10: 1976330679  0 out of 53 good  Bayesian Score: -2.745 | |
| |  | | --- | |  | | B11: -244159614  0 out of 46 good  Bayesian Score: -2.613 | | |  | | --- | |  | | B12: -655344035  0 out of 44 good  Bayesian Score: -2.572 | | |  | | --- | |  | | B13: -175882072  1 out of 91 good  Bayesian Score: -2.565 | | |  | | --- | |  | | B14: -1740465052  0 out of 43 good  Bayesian Score: -2.551 | | |  | | --- | |  | | B15: 1652635785  0 out of 43 good  Bayesian Score: -2.551 | |
| |  | | --- | |  | | B16: 1334400011  0 out of 43 good  Bayesian Score: -2.551 | | |  | | --- | |  | | B17: -179073144  0 out of 43 good  Bayesian Score: -2.551 | | |  | | --- | |  | | B18: 835229868  0 out of 42 good  Bayesian Score: -2.529 | | |  | | --- | |  | | B19: 172164547  0 out of 40 good  Bayesian Score: -2.484 | | |  | | --- | |  | | B20: -1887927950  0 out of 40 good  Bayesian Score: -2.484 | |
