# Supplementary material for: Identification of Multi-Target Anti-AD Chemical Constituents From Traditional Chinese Medicine Formulae by Integrating Virtual Screening and In Vitro Validation
Source: Front Pharmacol. 2021 Jul 16;12:709607. doi: 10.3389/fphar.2021.709607 (PMC8322649; doi:10.3389/fphar.2021.709607)
Supplement: Supplementary file 3 [file DataSheet1.ZIP › Good and bad fragments of 52 targets/MAPKAPK3.html]

Category NB\_mk14\_ECFP6: good features from ECFP\_6

|  |  |  |  |  |  |  |  |  |  |  |  |  |  |  |
| --- | --- | --- | --- | --- | --- | --- | --- | --- | --- | --- | --- | --- | --- | --- |
| |  | | --- | |  | | G1: -782828288  37 out of 37 good  Bayesian Score: 1.382 | | |  | | --- | |  | | G2: 1145122368  36 out of 36 good  Bayesian Score: 1.380 | | |  | | --- | |  | | G3: -185568434  36 out of 36 good  Bayesian Score: 1.380 | | |  | | --- | |  | | G4: -213377878  35 out of 35 good  Bayesian Score: 1.377 | | |  | | --- | |  | | G5: -1527664662  34 out of 34 good  Bayesian Score: 1.375 | |
| |  | | --- | |  | | G6: -986829520  34 out of 34 good  Bayesian Score: 1.375 | | |  | | --- | |  | | G7: 953896072  32 out of 32 good  Bayesian Score: 1.370 | | |  | | --- | |  | | G8: 467909457  32 out of 32 good  Bayesian Score: 1.370 | | |  | | --- | |  | | G9: -651948845  32 out of 32 good  Bayesian Score: 1.370 | | |  | | --- | |  | | G10: -1224093728  32 out of 32 good  Bayesian Score: 1.370 | |
| |  | | --- | |  | | G11: -1250320199  31 out of 31 good  Bayesian Score: 1.367 | | |  | | --- | |  | | G12: 1833945873  30 out of 30 good  Bayesian Score: 1.364 | | |  | | --- | |  | | G13: 824835316  30 out of 30 good  Bayesian Score: 1.364 | | |  | | --- | |  | | G14: 614774553  30 out of 30 good  Bayesian Score: 1.364 | | |  | | --- | |  | | G15: -296560819  28 out of 28 good  Bayesian Score: 1.357 | |
| |  | | --- | |  | | G16: 1096181188  36 out of 37 good  Bayesian Score: 1.355 | | |  | | --- | |  | | G17: 512782849  27 out of 27 good  Bayesian Score: 1.353 | | |  | | --- | |  | | G18: -996833399  32 out of 33 good  Bayesian Score: 1.342 | | |  | | --- | |  | | G19: 845378926  23 out of 23 good  Bayesian Score: 1.336 | | |  | | --- | |  | | G20: 558201926  37 out of 39 good  Bayesian Score: 1.335 | |

Category NB\_mk14\_ECFP6: bad features from ECFP\_6

|  |  |  |  |  |  |  |  |  |  |  |  |  |  |  |
| --- | --- | --- | --- | --- | --- | --- | --- | --- | --- | --- | --- | --- | --- | --- |
| |  | | --- | |  | | B1: -167460056  0 out of 59 good  Bayesian Score: -2.683 | | |  | | --- | |  | | B2: -1910270391  0 out of 29 good  Bayesian Score: -2.041 | | |  | | --- | |  | | B3: -1331450522  0 out of 28 good  Bayesian Score: -2.010 | | |  | | --- | |  | | B4: 1572579716  0 out of 27 good  Bayesian Score: -1.979 | | |  | | --- | |  | | B5: 657586427  0 out of 27 good  Bayesian Score: -1.979 | |
| |  | | --- | |  | | B6: -1897341097  1 out of 45 good  Bayesian Score: -1.740 | | |  | | --- | |  | | B7: -992506539  2 out of 66 good  Bayesian Score: -1.689 | | |  | | --- | |  | | B8: 1559650422  2 out of 66 good  Bayesian Score: -1.689 | | |  | | --- | |  | | B9: -1925046727  0 out of 18 good  Bayesian Score: -1.640 | | |  | | --- | |  | | B10: -175882072  0 out of 17 good  Bayesian Score: -1.594 | |
| |  | | --- | |  | | B11: -2024255407  0 out of 17 good  Bayesian Score: -1.594 | | |  | | --- | |  | | B12: 864518973  0 out of 17 good  Bayesian Score: -1.594 | | |  | | --- | |  | | B13: 683445015  1 out of 36 good  Bayesian Score: -1.538 | | |  | | --- | |  | | B14: -932108170  0 out of 15 good  Bayesian Score: -1.496 | | |  | | --- | |  | | B15: -1884411803  1 out of 33 good  Bayesian Score: -1.461 | |
| |  | | --- | |  | | B16: 1961554343  0 out of 14 good  Bayesian Score: -1.443 | | |  | | --- | |  | | B17: -1087070950  0 out of 14 good  Bayesian Score: -1.443 | | |  | | --- | |  | | B18: -1085223908  0 out of 14 good  Bayesian Score: -1.443 | | |  | | --- | |  | | B19: 51876938  0 out of 14 good  Bayesian Score: -1.443 | | |  | | --- | |  | | B20: -1699286547  0 out of 13 good  Bayesian Score: -1.387 | |
