# Supplementary material for: Identification of Multi-Target Anti-AD Chemical Constituents From Traditional Chinese Medicine Formulae by Integrating Virtual Screening and In Vitro Validation
Source: Front Pharmacol. 2021 Jul 16;12:709607. doi: 10.3389/fphar.2021.709607 (PMC8322649; doi:10.3389/fphar.2021.709607)
Supplement: Supplementary file 3 [file DataSheet1.ZIP › Good and bad fragments of 52 targets/MAPT.html]

Category NB\_mTAU\_ECFP6: good features from ECFP\_6

|  |  |  |  |  |  |  |  |  |  |  |  |  |  |  |
| --- | --- | --- | --- | --- | --- | --- | --- | --- | --- | --- | --- | --- | --- | --- |
| |  | | --- | |  | | G1: -1916518413  15 out of 15 good  Bayesian Score: 1.185 | | |  | | --- | |  | | G2: 1030926917  14 out of 14 good  Bayesian Score: 1.175 | | |  | | --- | |  | | G3: 827921966  14 out of 14 good  Bayesian Score: 1.175 | | |  | | --- | |  | | G4: -1831055759  15 out of 16 good  Bayesian Score: 1.134 | | |  | | --- | |  | | G5: -176483725  27 out of 31 good  Bayesian Score: 1.131 | |
| |  | | --- | |  | | G6: -1557361740  10 out of 10 good  Bayesian Score: 1.119 | | |  | | --- | |  | | G7: 1412082906  9 out of 9 good  Bayesian Score: 1.098 | | |  | | --- | |  | | G8: 1444648700  9 out of 9 good  Bayesian Score: 1.098 | | |  | | --- | |  | | G9: 1312166648  9 out of 9 good  Bayesian Score: 1.098 | | |  | | --- | |  | | G10: -785259062  9 out of 9 good  Bayesian Score: 1.098 | |
| |  | | --- | |  | | G11: 1163287371  9 out of 9 good  Bayesian Score: 1.098 | | |  | | --- | |  | | G12: 637962201  9 out of 9 good  Bayesian Score: 1.098 | | |  | | --- | |  | | G13: 1925887038  9 out of 9 good  Bayesian Score: 1.098 | | |  | | --- | |  | | G14: 555104647  9 out of 9 good  Bayesian Score: 1.098 | | |  | | --- | |  | | G15: 1051256653  9 out of 9 good  Bayesian Score: 1.098 | |
| |  | | --- | |  | | G16: 1986406965  9 out of 9 good  Bayesian Score: 1.098 | | |  | | --- | |  | | G17: 1715252781  9 out of 9 good  Bayesian Score: 1.098 | | |  | | --- | |  | | G18: -2045921967  9 out of 9 good  Bayesian Score: 1.098 | | |  | | --- | |  | | G19: 181063153  9 out of 9 good  Bayesian Score: 1.098 | | |  | | --- | |  | | G20: -296805570  9 out of 9 good  Bayesian Score: 1.098 | |

Category NB\_mTAU\_ECFP6: bad features from ECFP\_6

|  |  |  |  |  |  |  |  |  |  |  |  |  |  |  |
| --- | --- | --- | --- | --- | --- | --- | --- | --- | --- | --- | --- | --- | --- | --- |
| |  | | --- | |  | | B1: -167460056  0 out of 57 good  Bayesian Score: -2.759 | | |  | | --- | |  | | B2: -1910270391  0 out of 34 good  Bayesian Score: -2.284 | | |  | | --- | |  | | B3: -1331450522  0 out of 31 good  Bayesian Score: -2.202 | | |  | | --- | |  | | B4: 864518973  0 out of 22 good  Bayesian Score: -1.903 | | |  | | --- | |  | | B5: -2024255407  0 out of 20 good  Bayesian Score: -1.822 | |
| |  | | --- | |  | | B6: -801490360  0 out of 18 good  Bayesian Score: -1.735 | | |  | | --- | |  | | B7: 865857320  0 out of 16 good  Bayesian Score: -1.639 | | |  | | --- | |  | | B8: 51876938  0 out of 14 good  Bayesian Score: -1.533 | | |  | | --- | |  | | B9: -176846085  0 out of 13 good  Bayesian Score: -1.475 | | |  | | --- | |  | | B10: -830332112  0 out of 12 good  Bayesian Score: -1.414 | |
| |  | | --- | |  | | B11: -177935549  0 out of 12 good  Bayesian Score: -1.414 | | |  | | --- | |  | | B12: 1088861418  0 out of 12 good  Bayesian Score: -1.414 | | |  | | --- | |  | | B13: -302078100  1 out of 27 good  Bayesian Score: -1.386 | | |  | | --- | |  | | B14: -175882072  0 out of 10 good  Bayesian Score: -1.279 | | |  | | --- | |  | | B15: -179515162  0 out of 10 good  Bayesian Score: -1.279 | |
| |  | | --- | |  | | B16: -1426923364  0 out of 10 good  Bayesian Score: -1.279 | | |  | | --- | |  | | B17: 459826767  1 out of 23 good  Bayesian Score: -1.248 | | |  | | --- | |  | | B18: 412256466  0 out of 9 good  Bayesian Score: -1.204 | | |  | | --- | |  | | B19: 1961554343  0 out of 9 good  Bayesian Score: -1.204 | | |  | | --- | |  | | B20: 781519895  0 out of 9 good  Bayesian Score: -1.204 | |
