# Supplementary material for: Identification of Multi-Target Anti-AD Chemical Constituents From Traditional Chinese Medicine Formulae by Integrating Virtual Screening and In Vitro Validation
Source: Front Pharmacol. 2021 Jul 16;12:709607. doi: 10.3389/fphar.2021.709607 (PMC8322649; doi:10.3389/fphar.2021.709607)
Supplement: Supplementary file 3 [file DataSheet1.ZIP › Good and bad fragments of 52 targets/MPO.html]

Category NB\_myeloperoxidase-ECFP6: good features from ECFP\_6

|  |  |  |  |  |  |  |  |  |  |  |  |  |  |  |
| --- | --- | --- | --- | --- | --- | --- | --- | --- | --- | --- | --- | --- | --- | --- |
| |  | | --- | |  | | G1: -953984246  53 out of 53 good  Bayesian Score: 1.243 | | |  | | --- | |  | | G2: -93267474  47 out of 47 good  Bayesian Score: 1.237 | | |  | | --- | |  | | G3: 1754851917  45 out of 45 good  Bayesian Score: 1.235 | | |  | | --- | |  | | G4: -1560646374  40 out of 40 good  Bayesian Score: 1.229 | | |  | | --- | |  | | G5: -4900690  39 out of 39 good  Bayesian Score: 1.227 | |
| |  | | --- | |  | | G6: 58882645  39 out of 39 good  Bayesian Score: 1.227 | | |  | | --- | |  | | G7: -1659633832  39 out of 39 good  Bayesian Score: 1.227 | | |  | | --- | |  | | G8: -1955758686  39 out of 39 good  Bayesian Score: 1.227 | | |  | | --- | |  | | G9: 2082735577  39 out of 39 good  Bayesian Score: 1.227 | | |  | | --- | |  | | G10: -1613480181  39 out of 39 good  Bayesian Score: 1.227 | |
| |  | | --- | |  | | G11: 434019025  39 out of 39 good  Bayesian Score: 1.227 | | |  | | --- | |  | | G12: -1804650333  39 out of 39 good  Bayesian Score: 1.227 | | |  | | --- | |  | | G13: 73185434  39 out of 39 good  Bayesian Score: 1.227 | | |  | | --- | |  | | G14: -1763137688  39 out of 39 good  Bayesian Score: 1.227 | | |  | | --- | |  | | G15: -697117498  39 out of 39 good  Bayesian Score: 1.227 | |
| |  | | --- | |  | | G16: 348098785  37 out of 37 good  Bayesian Score: 1.224 | | |  | | --- | |  | | G17: -1020814177  37 out of 37 good  Bayesian Score: 1.224 | | |  | | --- | |  | | G18: -1020449580  47 out of 48 good  Bayesian Score: 1.218 | | |  | | --- | |  | | G19: -97599322  41 out of 42 good  Bayesian Score: 1.208 | | |  | | --- | |  | | G20: -176686665  41 out of 42 good  Bayesian Score: 1.208 | |

Category NB\_myeloperoxidase-ECFP6: bad features from ECFP\_6

|  |  |  |  |  |  |  |  |  |  |  |  |  |  |  |
| --- | --- | --- | --- | --- | --- | --- | --- | --- | --- | --- | --- | --- | --- | --- |
| |  | | --- | |  | | B1: 859796174  0 out of 35 good  Bayesian Score: -2.363 | | |  | | --- | |  | | B2: 657586427  0 out of 31 good  Bayesian Score: -2.254 | | |  | | --- | |  | | B3: -167460056  2 out of 99 good  Bayesian Score: -2.242 | | |  | | --- | |  | | B4: 914325265  0 out of 30 good  Bayesian Score: -2.225 | | |  | | --- | |  | | B5: 781519895  0 out of 30 good  Bayesian Score: -2.225 | |
| |  | | --- | |  | | B6: -801490360  0 out of 29 good  Bayesian Score: -2.195 | | |  | | --- | |  | | B7: -1331450522  1 out of 56 good  Bayesian Score: -2.104 | | |  | | --- | |  | | B8: -817402818  0 out of 24 good  Bayesian Score: -2.028 | | |  | | --- | |  | | B9: -176846085  0 out of 21 good  Bayesian Score: -1.913 | | |  | | --- | |  | | B10: -1925046727  0 out of 21 good  Bayesian Score: -1.913 | |
| |  | | --- | |  | | B11: 865482986  0 out of 20 good  Bayesian Score: -1.872 | | |  | | --- | |  | | B12: -1087070950  0 out of 18 good  Bayesian Score: -1.784 | | |  | | --- | |  | | B13: -655344035  0 out of 17 good  Bayesian Score: -1.736 | | |  | | --- | |  | | B14: 865857320  0 out of 17 good  Bayesian Score: -1.736 | | |  | | --- | |  | | B15: 1996740348  0 out of 16 good  Bayesian Score: -1.687 | |
| |  | | --- | |  | | B16: 412256466  0 out of 16 good  Bayesian Score: -1.687 | | |  | | --- | |  | | B17: 683445015  1 out of 35 good  Bayesian Score: -1.670 | | |  | | --- | |  | | B18: -709633021  0 out of 15 good  Bayesian Score: -1.634 | | |  | | --- | |  | | B19: -1416572622  0 out of 15 good  Bayesian Score: -1.634 | | |  | | --- | |  | | B20: 2023785560  0 out of 13 good  Bayesian Score: -1.521 | |
