# Supplementary material for: Identification of Multi-Target Anti-AD Chemical Constituents From Traditional Chinese Medicine Formulae by Integrating Virtual Screening and In Vitro Validation
Source: Front Pharmacol. 2021 Jul 16;12:709607. doi: 10.3389/fphar.2021.709607 (PMC8322649; doi:10.3389/fphar.2021.709607)
Supplement: Supplementary file 3 [file DataSheet1.ZIP › Good and bad fragments of 52 targets/NOS2.html]

Category NB\_inos\_ECFP6: good features from ECFP\_6

|  |  |  |  |  |  |  |  |  |  |  |  |  |  |  |
| --- | --- | --- | --- | --- | --- | --- | --- | --- | --- | --- | --- | --- | --- | --- |
| |  | | --- | |  | | G1: -837836388  114 out of 114 good  Bayesian Score: 1.224 | | |  | | --- | |  | | G2: 822144614  114 out of 114 good  Bayesian Score: 1.224 | | |  | | --- | |  | | G3: 809660515  114 out of 114 good  Bayesian Score: 1.224 | | |  | | --- | |  | | G4: 1276556621  98 out of 98 good  Bayesian Score: 1.221 | | |  | | --- | |  | | G5: 916616722  98 out of 98 good  Bayesian Score: 1.221 | |
| |  | | --- | |  | | G6: -800732900  98 out of 98 good  Bayesian Score: 1.221 | | |  | | --- | |  | | G7: 558728711  92 out of 92 good  Bayesian Score: 1.219 | | |  | | --- | |  | | G8: 1797090558  92 out of 92 good  Bayesian Score: 1.219 | | |  | | --- | |  | | G9: 270026782  92 out of 92 good  Bayesian Score: 1.219 | | |  | | --- | |  | | G10: 584323138  92 out of 92 good  Bayesian Score: 1.219 | |
| |  | | --- | |  | | G11: 1658139883  92 out of 92 good  Bayesian Score: 1.219 | | |  | | --- | |  | | G12: -1699495259  92 out of 92 good  Bayesian Score: 1.219 | | |  | | --- | |  | | G13: -562189999  87 out of 87 good  Bayesian Score: 1.218 | | |  | | --- | |  | | G14: -771398528  84 out of 84 good  Bayesian Score: 1.217 | | |  | | --- | |  | | G15: 1002739372  84 out of 84 good  Bayesian Score: 1.217 | |
| |  | | --- | |  | | G16: -1321572589  83 out of 83 good  Bayesian Score: 1.217 | | |  | | --- | |  | | G17: -552225728  81 out of 81 good  Bayesian Score: 1.216 | | |  | | --- | |  | | G18: 285992842  114 out of 115 good  Bayesian Score: 1.216 | | |  | | --- | |  | | G19: 758792983  114 out of 115 good  Bayesian Score: 1.216 | | |  | | --- | |  | | G20: -1385099951  76 out of 76 good  Bayesian Score: 1.214 | |

Category NB\_inos\_ECFP6: bad features from ECFP\_6

|  |  |  |  |  |  |  |  |  |  |  |  |  |  |  |
| --- | --- | --- | --- | --- | --- | --- | --- | --- | --- | --- | --- | --- | --- | --- |
| |  | | --- | |  | | B1: 1976330679  0 out of 178 good  Bayesian Score: -3.956 | | |  | | --- | |  | | B2: -591526139  0 out of 172 good  Bayesian Score: -3.922 | | |  | | --- | |  | | B3: -244159614  0 out of 162 good  Bayesian Score: -3.863 | | |  | | --- | |  | | B4: 1961554343  0 out of 152 good  Bayesian Score: -3.801 | | |  | | --- | |  | | B5: -2130275420  0 out of 149 good  Bayesian Score: -3.781 | |
| |  | | --- | |  | | B6: -1416572622  2 out of 417 good  Bayesian Score: -3.697 | | |  | | --- | |  | | B7: -2127980805  0 out of 99 good  Bayesian Score: -3.384 | | |  | | --- | |  | | B8: 233520344  0 out of 95 good  Bayesian Score: -3.344 | | |  | | --- | |  | | B9: 2023785560  0 out of 72 good  Bayesian Score: -3.078 | | |  | | --- | |  | | B10: 865857320  1 out of 146 good  Bayesian Score: -3.068 | |
| |  | | --- | |  | | B11: 2022454958  1 out of 144 good  Bayesian Score: -3.055 | | |  | | --- | |  | | B12: 1133499173  0 out of 67 good  Bayesian Score: -3.010 | | |  | | --- | |  | | B13: 1945129186  0 out of 64 good  Bayesian Score: -2.966 | | |  | | --- | |  | | B14: 860114273  0 out of 61 good  Bayesian Score: -2.921 | | |  | | --- | |  | | B15: -232258228  0 out of 61 good  Bayesian Score: -2.921 | |
| |  | | --- | |  | | B16: -224638920  0 out of 60 good  Bayesian Score: -2.905 | | |  | | --- | |  | | B17: -1939757055  0 out of 57 good  Bayesian Score: -2.857 | | |  | | --- | |  | | B18: 2116455019  0 out of 57 good  Bayesian Score: -2.857 | | |  | | --- | |  | | B19: 1430169877  1 out of 115 good  Bayesian Score: -2.836 | | |  | | --- | |  | | B20: -342718945  0 out of 52 good  Bayesian Score: -2.770 | |
