# Supplementary material for: Identification of Multi-Target Anti-AD Chemical Constituents From Traditional Chinese Medicine Formulae by Integrating Virtual Screening and In Vitro Validation
Source: Front Pharmacol. 2021 Jul 16;12:709607. doi: 10.3389/fphar.2021.709607 (PMC8322649; doi:10.3389/fphar.2021.709607)
Supplement: Supplementary file 3 [file DataSheet1.ZIP › Good and bad fragments of 52 targets/PDE4A.html]

Category Bayesian-PDE4A: good features from ECFP\_6

|  |  |  |  |  |  |  |  |  |  |  |  |  |  |  |
| --- | --- | --- | --- | --- | --- | --- | --- | --- | --- | --- | --- | --- | --- | --- |
| |  | | --- | |  | | G1: -1443920737  91 out of 91 good  Bayesian Score: 1.186 | | |  | | --- | |  | | G2: 2036640078  90 out of 90 good  Bayesian Score: 1.186 | | |  | | --- | |  | | G3: 969779495  89 out of 89 good  Bayesian Score: 1.186 | | |  | | --- | |  | | G4: -813441769  88 out of 88 good  Bayesian Score: 1.185 | | |  | | --- | |  | | G5: 795370532  88 out of 88 good  Bayesian Score: 1.185 | |
| |  | | --- | |  | | G6: -909137442  85 out of 85 good  Bayesian Score: 1.184 | | |  | | --- | |  | | G7: 1540484686  84 out of 84 good  Bayesian Score: 1.184 | | |  | | --- | |  | | G8: 190343271  80 out of 80 good  Bayesian Score: 1.183 | | |  | | --- | |  | | G9: 2135649806  79 out of 79 good  Bayesian Score: 1.182 | | |  | | --- | |  | | G10: 85005252  79 out of 79 good  Bayesian Score: 1.182 | |
| |  | | --- | |  | | G11: -411269701  79 out of 79 good  Bayesian Score: 1.182 | | |  | | --- | |  | | G12: -1696447461  79 out of 79 good  Bayesian Score: 1.182 | | |  | | --- | |  | | G13: 1052014839  79 out of 79 good  Bayesian Score: 1.182 | | |  | | --- | |  | | G14: -2121249866  77 out of 77 good  Bayesian Score: 1.182 | | |  | | --- | |  | | G15: 1067500008  77 out of 77 good  Bayesian Score: 1.182 | |
| |  | | --- | |  | | G16: 1598373791  73 out of 73 good  Bayesian Score: 1.180 | | |  | | --- | |  | | G17: 1386779609  70 out of 70 good  Bayesian Score: 1.179 | | |  | | --- | |  | | G18: 1954568086  69 out of 69 good  Bayesian Score: 1.178 | | |  | | --- | |  | | G19: -498089686  69 out of 69 good  Bayesian Score: 1.178 | | |  | | --- | |  | | G20: 638874826  67 out of 67 good  Bayesian Score: 1.177 | |

Category Bayesian-PDE4A: bad features from ECFP\_6

|  |  |  |  |  |  |  |  |  |  |  |  |  |  |  |
| --- | --- | --- | --- | --- | --- | --- | --- | --- | --- | --- | --- | --- | --- | --- |
| |  | | --- | |  | | B1: 865482986  0 out of 83 good  Bayesian Score: -3.247 | | |  | | --- | |  | | B2: -655344035  0 out of 73 good  Bayesian Score: -3.124 | | |  | | --- | |  | | B3: 2085698692  0 out of 63 good  Bayesian Score: -2.984 | | |  | | --- | |  | | B4: -661766797  0 out of 57 good  Bayesian Score: -2.889 | | |  | | --- | |  | | B5: -206566761  0 out of 54 good  Bayesian Score: -2.838 | |
| |  | | --- | |  | | B6: 1334973442  0 out of 50 good  Bayesian Score: -2.765 | | |  | | --- | |  | | B7: -845108448  0 out of 40 good  Bayesian Score: -2.558 | | |  | | --- | |  | | B8: 1335702447  0 out of 38 good  Bayesian Score: -2.511 | | |  | | --- | |  | | B9: 464808839  0 out of 37 good  Bayesian Score: -2.486 | | |  | | --- | |  | | B10: -560785749  0 out of 37 good  Bayesian Score: -2.486 | |
| |  | | --- | |  | | B11: 1182722866  0 out of 37 good  Bayesian Score: -2.486 | | |  | | --- | |  | | B12: -1742225957  0 out of 36 good  Bayesian Score: -2.461 | | |  | | --- | |  | | B13: -1956535100  0 out of 36 good  Bayesian Score: -2.461 | | |  | | --- | |  | | B14: 1427820655  1 out of 74 good  Bayesian Score: -2.444 | | |  | | --- | |  | | B15: -955816473  0 out of 35 good  Bayesian Score: -2.435 | |
| |  | | --- | |  | | B16: 770725373  0 out of 34 good  Bayesian Score: -2.409 | | |  | | --- | |  | | B17: -1071952480  0 out of 34 good  Bayesian Score: -2.409 | | |  | | --- | |  | | B18: -787327968  0 out of 34 good  Bayesian Score: -2.409 | | |  | | --- | |  | | B19: 2086924722  0 out of 33 good  Bayesian Score: -2.382 | | |  | | --- | |  | | B20: 1637630297  0 out of 33 good  Bayesian Score: -2.382 | |
