# Supplementary material for: Identification of Multi-Target Anti-AD Chemical Constituents From Traditional Chinese Medicine Formulae by Integrating Virtual Screening and In Vitro Validation
Source: Front Pharmacol. 2021 Jul 16;12:709607. doi: 10.3389/fphar.2021.709607 (PMC8322649; doi:10.3389/fphar.2021.709607)
Supplement: Supplementary file 3 [file DataSheet1.ZIP › Good and bad fragments of 52 targets/PDE4B.html]

Category Bayesian-PDE4B: good features from ECFP\_6

|  |  |  |  |  |  |  |  |  |  |  |  |  |  |  |
| --- | --- | --- | --- | --- | --- | --- | --- | --- | --- | --- | --- | --- | --- | --- |
| |  | | --- | |  | | G1: 1067500008  74 out of 74 good  Bayesian Score: 1.219 | | |  | | --- | |  | | G2: -2121249866  73 out of 73 good  Bayesian Score: 1.218 | | |  | | --- | |  | | G3: -644250665  72 out of 72 good  Bayesian Score: 1.218 | | |  | | --- | |  | | G4: 1428925092  70 out of 70 good  Bayesian Score: 1.217 | | |  | | --- | |  | | G5: 1386779609  70 out of 70 good  Bayesian Score: 1.217 | |
| |  | | --- | |  | | G6: 1598373791  67 out of 67 good  Bayesian Score: 1.215 | | |  | | --- | |  | | G7: 795370532  93 out of 94 good  Bayesian Score: 1.215 | | |  | | --- | |  | | G8: -498089686  65 out of 65 good  Bayesian Score: 1.214 | | |  | | --- | |  | | G9: 1052014839  64 out of 64 good  Bayesian Score: 1.214 | | |  | | --- | |  | | G10: 85005252  64 out of 64 good  Bayesian Score: 1.214 | |
| |  | | --- | |  | | G11: -411269701  64 out of 64 good  Bayesian Score: 1.214 | | |  | | --- | |  | | G12: -1696447461  63 out of 63 good  Bayesian Score: 1.213 | | |  | | --- | |  | | G13: 1310940530  79 out of 80 good  Bayesian Score: 1.209 | | |  | | --- | |  | | G14: 1156370971  50 out of 50 good  Bayesian Score: 1.204 | | |  | | --- | |  | | G15: 1461686767  50 out of 50 good  Bayesian Score: 1.204 | |
| |  | | --- | |  | | G16: 1966275298  50 out of 50 good  Bayesian Score: 1.204 | | |  | | --- | |  | | G17: -340626066  50 out of 50 good  Bayesian Score: 1.204 | | |  | | --- | |  | | G18: 1954568086  70 out of 71 good  Bayesian Score: 1.203 | | |  | | --- | |  | | G19: 788797351  49 out of 49 good  Bayesian Score: 1.203 | | |  | | --- | |  | | G20: 638874826  69 out of 70 good  Bayesian Score: 1.203 | |

Category Bayesian-PDE4B: bad features from ECFP\_6

|  |  |  |  |  |  |  |  |  |  |  |  |  |  |  |
| --- | --- | --- | --- | --- | --- | --- | --- | --- | --- | --- | --- | --- | --- | --- |
| |  | | --- | |  | | B1: -661766797  0 out of 82 good  Bayesian Score: -3.197 | | |  | | --- | |  | | B2: 770725373  0 out of 51 good  Bayesian Score: -2.747 | | |  | | --- | |  | | B3: -649580166  0 out of 51 good  Bayesian Score: -2.747 | | |  | | --- | |  | | B4: -1887927950  0 out of 51 good  Bayesian Score: -2.747 | | |  | | --- | |  | | B5: 172164547  0 out of 51 good  Bayesian Score: -2.747 | |
| |  | | --- | |  | | B6: 1427820655  1 out of 101 good  Bayesian Score: -2.705 | | |  | | --- | |  | | B7: 1731843802  2 out of 153 good  Bayesian Score: -2.703 | | |  | | --- | |  | | B8: -2137232509  0 out of 46 good  Bayesian Score: -2.651 | | |  | | --- | |  | | B9: 1335702447  0 out of 45 good  Bayesian Score: -2.630 | | |  | | --- | |  | | B10: 454057662  0 out of 45 good  Bayesian Score: -2.630 | |
| |  | | --- | |  | | B11: -1926229349  1 out of 88 good  Bayesian Score: -2.572 | | |  | | --- | |  | | B12: -533780882  1 out of 88 good  Bayesian Score: -2.572 | | |  | | --- | |  | | B13: -669219631  0 out of 42 good  Bayesian Score: -2.566 | | |  | | --- | |  | | B14: 2085698692  1 out of 87 good  Bayesian Score: -2.561 | | |  | | --- | |  | | B15: -1087070950  3 out of 175 good  Bayesian Score: -2.547 | |
| |  | | --- | |  | | B16: 577912634  0 out of 41 good  Bayesian Score: -2.544 | | |  | | --- | |  | | B17: -787327968  0 out of 41 good  Bayesian Score: -2.544 | | |  | | --- | |  | | B18: -1071952480  0 out of 41 good  Bayesian Score: -2.544 | | |  | | --- | |  | | B19: -1939757055  0 out of 40 good  Bayesian Score: -2.521 | | |  | | --- | |  | | B20: 2082478181  0 out of 39 good  Bayesian Score: -2.498 | |
