# Supplementary material for: Identification of Multi-Target Anti-AD Chemical Constituents From Traditional Chinese Medicine Formulae by Integrating Virtual Screening and In Vitro Validation
Source: Front Pharmacol. 2021 Jul 16;12:709607. doi: 10.3389/fphar.2021.709607 (PMC8322649; doi:10.3389/fphar.2021.709607)
Supplement: Supplementary file 3 [file DataSheet1.ZIP › Good and bad fragments of 52 targets/PDE9A.html]

Category NB\_pde9a-ECFP6: good features from ECFP\_6

|  |  |  |  |  |  |  |  |  |  |  |  |  |  |  |
| --- | --- | --- | --- | --- | --- | --- | --- | --- | --- | --- | --- | --- | --- | --- |
| |  | | --- | |  | | G1: 2144876429  100 out of 100 good  Bayesian Score: 1.197 | | |  | | --- | |  | | G2: -1344431299  100 out of 100 good  Bayesian Score: 1.197 | | |  | | --- | |  | | G3: 706717463  100 out of 100 good  Bayesian Score: 1.197 | | |  | | --- | |  | | G4: -442456406  85 out of 85 good  Bayesian Score: 1.193 | | |  | | --- | |  | | G5: 1958683973  85 out of 85 good  Bayesian Score: 1.193 | |
| |  | | --- | |  | | G6: -1084891596  85 out of 85 good  Bayesian Score: 1.193 | | |  | | --- | |  | | G7: -1230044533  85 out of 85 good  Bayesian Score: 1.193 | | |  | | --- | |  | | G8: 344345443  85 out of 85 good  Bayesian Score: 1.193 | | |  | | --- | |  | | G9: 2063258256  85 out of 85 good  Bayesian Score: 1.193 | | |  | | --- | |  | | G10: -1946325183  85 out of 85 good  Bayesian Score: 1.193 | |
| |  | | --- | |  | | G11: -750736791  85 out of 85 good  Bayesian Score: 1.193 | | |  | | --- | |  | | G12: -1620614014  85 out of 85 good  Bayesian Score: 1.193 | | |  | | --- | |  | | G13: -961383061  100 out of 101 good  Bayesian Score: 1.188 | | |  | | --- | |  | | G14: 992477236  61 out of 61 good  Bayesian Score: 1.183 | | |  | | --- | |  | | G15: 941544649  61 out of 61 good  Bayesian Score: 1.183 | |
| |  | | --- | |  | | G16: 517179506  61 out of 61 good  Bayesian Score: 1.183 | | |  | | --- | |  | | G17: 1559666245  61 out of 61 good  Bayesian Score: 1.183 | | |  | | --- | |  | | G18: -287300789  61 out of 61 good  Bayesian Score: 1.183 | | |  | | --- | |  | | G19: 2136048231  61 out of 61 good  Bayesian Score: 1.183 | | |  | | --- | |  | | G20: -1074508478  52 out of 52 good  Bayesian Score: 1.176 | |

Category NB\_pde9a-ECFP6: bad features from ECFP\_6

|  |  |  |  |  |  |  |  |  |  |  |  |  |  |  |
| --- | --- | --- | --- | --- | --- | --- | --- | --- | --- | --- | --- | --- | --- | --- |
| |  | | --- | |  | | B1: 781519895  0 out of 103 good  Bayesian Score: -3.447 | | |  | | --- | |  | | B2: 914325265  0 out of 65 good  Bayesian Score: -3.005 | | |  | | --- | |  | | B3: -1925046727  0 out of 56 good  Bayesian Score: -2.864 | | |  | | --- | |  | | B4: 1430169877  0 out of 55 good  Bayesian Score: -2.847 | | |  | | --- | |  | | B5: 657586427  0 out of 52 good  Bayesian Score: -2.794 | |
| |  | | --- | |  | | B6: -655344035  0 out of 51 good  Bayesian Score: -2.776 | | |  | | --- | |  | | B7: -1087070950  0 out of 45 good  Bayesian Score: -2.659 | | |  | | --- | |  | | B8: -570915357  0 out of 44 good  Bayesian Score: -2.638 | | |  | | --- | |  | | B9: -175146122  0 out of 42 good  Bayesian Score: -2.595 | | |  | | --- | |  | | B10: -556429595  0 out of 38 good  Bayesian Score: -2.502 | |
| |  | | --- | |  | | B11: 1043790491  0 out of 34 good  Bayesian Score: -2.401 | | |  | | --- | |  | | B12: 2104376220  0 out of 34 good  Bayesian Score: -2.401 | | |  | | --- | |  | | B13: -659271057  0 out of 34 good  Bayesian Score: -2.401 | | |  | | --- | |  | | B14: 85262808  0 out of 33 good  Bayesian Score: -2.374 | | |  | | --- | |  | | B15: -215026467  0 out of 33 good  Bayesian Score: -2.374 | |
| |  | | --- | |  | | B16: -1426923364  0 out of 30 good  Bayesian Score: -2.288 | | |  | | --- | |  | | B17: 859433814  0 out of 30 good  Bayesian Score: -2.288 | | |  | | --- | |  | | B18: 1333660716  0 out of 30 good  Bayesian Score: -2.288 | | |  | | --- | |  | | B19: 715524658  0 out of 26 good  Bayesian Score: -2.160 | | |  | | --- | |  | | B20: -176483725  0 out of 26 good  Bayesian Score: -2.160 | |
