# Supplementary material for: Identification of Multi-Target Anti-AD Chemical Constituents From Traditional Chinese Medicine Formulae by Integrating Virtual Screening and In Vitro Validation
Source: Front Pharmacol. 2021 Jul 16;12:709607. doi: 10.3389/fphar.2021.709607 (PMC8322649; doi:10.3389/fphar.2021.709607)
Supplement: Supplementary file 3 [file DataSheet1.ZIP › Good and bad fragments of 52 targets/PDHX.html]

Category NB\_PDK-ECFP6: good features from ECFP\_6

|  |  |  |  |  |  |  |  |  |  |  |  |  |  |  |
| --- | --- | --- | --- | --- | --- | --- | --- | --- | --- | --- | --- | --- | --- | --- |
| |  | | --- | |  | | G1: -902660362  433 out of 433 good  Bayesian Score: 1.271 | | |  | | --- | |  | | G2: 1413241096  433 out of 433 good  Bayesian Score: 1.271 | | |  | | --- | |  | | G3: -1668652729  433 out of 433 good  Bayesian Score: 1.271 | | |  | | --- | |  | | G4: 1109976565  432 out of 432 good  Bayesian Score: 1.271 | | |  | | --- | |  | | G5: -764846601  431 out of 431 good  Bayesian Score: 1.271 | |
| |  | | --- | |  | | G6: -1740442182  227 out of 227 good  Bayesian Score: 1.265 | | |  | | --- | |  | | G7: -1743848436  196 out of 196 good  Bayesian Score: 1.264 | | |  | | --- | |  | | G8: 1093961365  196 out of 196 good  Bayesian Score: 1.264 | | |  | | --- | |  | | G9: -930962236  196 out of 196 good  Bayesian Score: 1.264 | | |  | | --- | |  | | G10: -153760748  189 out of 189 good  Bayesian Score: 1.263 | |
| |  | | --- | |  | | G11: 1452515351  188 out of 188 good  Bayesian Score: 1.263 | | |  | | --- | |  | | G12: 978354880  188 out of 188 good  Bayesian Score: 1.263 | | |  | | --- | |  | | G13: 726446432  188 out of 188 good  Bayesian Score: 1.263 | | |  | | --- | |  | | G14: 567917108  186 out of 186 good  Bayesian Score: 1.263 | | |  | | --- | |  | | G15: -132967869  186 out of 186 good  Bayesian Score: 1.263 | |
| |  | | --- | |  | | G16: -296907946  228 out of 229 good  Bayesian Score: 1.261 | | |  | | --- | |  | | G17: -1369966269  227 out of 228 good  Bayesian Score: 1.261 | | |  | | --- | |  | | G18: -1143727172  145 out of 145 good  Bayesian Score: 1.259 | | |  | | --- | |  | | G19: 804853493  145 out of 145 good  Bayesian Score: 1.259 | | |  | | --- | |  | | G20: -1432726679  137 out of 137 good  Bayesian Score: 1.258 | |

Category NB\_PDK-ECFP6: bad features from ECFP\_6

|  |  |  |  |  |  |  |  |  |  |  |  |  |  |  |
| --- | --- | --- | --- | --- | --- | --- | --- | --- | --- | --- | --- | --- | --- | --- |
| |  | | --- | |  | | B1: -1087070950  0 out of 174 good  Bayesian Score: -3.903 | | |  | | --- | |  | | B2: -830332112  0 out of 147 good  Bayesian Score: -3.738 | | |  | | --- | |  | | B3: 781519895  0 out of 121 good  Bayesian Score: -3.548 | | |  | | --- | |  | | B4: 85262808  0 out of 118 good  Bayesian Score: -3.524 | | |  | | --- | |  | | B5: 1945129186  0 out of 116 good  Bayesian Score: -3.507 | |
| |  | | --- | |  | | B6: 912478223  0 out of 116 good  Bayesian Score: -3.507 | | |  | | --- | |  | | B7: 859433814  0 out of 94 good  Bayesian Score: -3.304 | | |  | | --- | |  | | B8: -659271057  0 out of 92 good  Bayesian Score: -3.283 | | |  | | --- | |  | | B9: 1043790491  0 out of 92 good  Bayesian Score: -3.283 | | |  | | --- | |  | | B10: 2104376220  0 out of 91 good  Bayesian Score: -3.273 | |
| |  | | --- | |  | | B11: -1508366470  0 out of 88 good  Bayesian Score: -3.241 | | |  | | --- | |  | | B12: -797085356  0 out of 83 good  Bayesian Score: -3.184 | | |  | | --- | |  | | B13: 2102150379  0 out of 83 good  Bayesian Score: -3.184 | | |  | | --- | |  | | B14: -215026467  0 out of 79 good  Bayesian Score: -3.137 | | |  | | --- | |  | | B15: 1306977740  0 out of 73 good  Bayesian Score: -3.062 | |
| |  | | --- | |  | | B16: 1427820655  0 out of 71 good  Bayesian Score: -3.035 | | |  | | --- | |  | | B17: 1334415134  0 out of 68 good  Bayesian Score: -2.994 | | |  | | --- | |  | | B18: -845108448  0 out of 68 good  Bayesian Score: -2.994 | | |  | | --- | |  | | B19: -1236953626  0 out of 67 good  Bayesian Score: -2.980 | | |  | | --- | |  | | B20: -178525456  0 out of 66 good  Bayesian Score: -2.966 | |
