# Supplementary material for: Identification of Multi-Target Anti-AD Chemical Constituents From Traditional Chinese Medicine Formulae by Integrating Virtual Screening and In Vitro Validation
Source: Front Pharmacol. 2021 Jul 16;12:709607. doi: 10.3389/fphar.2021.709607 (PMC8322649; doi:10.3389/fphar.2021.709607)
Supplement: Supplementary file 3 [file DataSheet1.ZIP › Good and bad fragments of 52 targets/PIN1.html]

Category NB\_pin1-ECFP6: good features from ECFP\_6

|  |  |  |  |  |  |  |  |  |  |  |  |  |  |  |
| --- | --- | --- | --- | --- | --- | --- | --- | --- | --- | --- | --- | --- | --- | --- |
| |  | | --- | |  | | G1: 770157610  33 out of 33 good  Bayesian Score: 1.113 | | |  | | --- | |  | | G2: 1637591468  27 out of 27 good  Bayesian Score: 1.099 | | |  | | --- | |  | | G3: 1233434266  27 out of 27 good  Bayesian Score: 1.099 | | |  | | --- | |  | | G4: 2006518499  27 out of 27 good  Bayesian Score: 1.099 | | |  | | --- | |  | | G5: -755462605  27 out of 27 good  Bayesian Score: 1.099 | |
| |  | | --- | |  | | G6: -2019199918  27 out of 27 good  Bayesian Score: 1.099 | | |  | | --- | |  | | G7: -1839239287  22 out of 22 good  Bayesian Score: 1.083 | | |  | | --- | |  | | G8: -81428579  32 out of 33 good  Bayesian Score: 1.083 | | |  | | --- | |  | | G9: 1336540477  32 out of 33 good  Bayesian Score: 1.083 | | |  | | --- | |  | | G10: 2025485523  38 out of 40 good  Bayesian Score: 1.073 | |
| |  | | --- | |  | | G11: 710652510  28 out of 29 good  Bayesian Score: 1.070 | | |  | | --- | |  | | G12: 1640927238  19 out of 19 good  Bayesian Score: 1.070 | | |  | | --- | |  | | G13: -1905455774  19 out of 19 good  Bayesian Score: 1.070 | | |  | | --- | |  | | G14: -1302110264  19 out of 19 good  Bayesian Score: 1.070 | | |  | | --- | |  | | G15: 1930526706  18 out of 18 good  Bayesian Score: 1.065 | |
| |  | | --- | |  | | G16: -174623179  18 out of 18 good  Bayesian Score: 1.065 | | |  | | --- | |  | | G17: 1843592437  18 out of 18 good  Bayesian Score: 1.065 | | |  | | --- | |  | | G18: 1099868407  18 out of 18 good  Bayesian Score: 1.065 | | |  | | --- | |  | | G19: 1691569519  18 out of 18 good  Bayesian Score: 1.065 | | |  | | --- | |  | | G20: -1146334930  18 out of 18 good  Bayesian Score: 1.065 | |

Category NB\_pin1-ECFP6: bad features from ECFP\_6

|  |  |  |  |  |  |  |  |  |  |  |  |  |  |  |
| --- | --- | --- | --- | --- | --- | --- | --- | --- | --- | --- | --- | --- | --- | --- |
| |  | | --- | |  | | B1: -661766797  0 out of 39 good  Bayesian Score: -2.567 | | |  | | --- | |  | | B2: -655344035  0 out of 34 good  Bayesian Score: -2.441 | | |  | | --- | |  | | B3: -302078100  0 out of 30 good  Bayesian Score: -2.327 | | |  | | --- | |  | | B4: 459826767  0 out of 28 good  Bayesian Score: -2.265 | | |  | | --- | |  | | B5: 657586427  0 out of 28 good  Bayesian Score: -2.265 | |
| |  | | --- | |  | | B6: -677309799  0 out of 25 good  Bayesian Score: -2.164 | | |  | | --- | |  | | B7: -830332112  0 out of 17 good  Bayesian Score: -1.831 | | |  | | --- | |  | | B8: -1699286547  0 out of 16 good  Bayesian Score: -1.781 | | |  | | --- | |  | | B9: -797085356  0 out of 16 good  Bayesian Score: -1.781 | | |  | | --- | |  | | B10: 2102150379  0 out of 16 good  Bayesian Score: -1.781 | |
| |  | | --- | |  | | B11: 2147419938  0 out of 16 good  Bayesian Score: -1.781 | | |  | | --- | |  | | B12: 863188371  1 out of 35 good  Bayesian Score: -1.774 | | |  | | --- | |  | | B13: 1408898974  0 out of 15 good  Bayesian Score: -1.727 | | |  | | --- | |  | | B14: 1996163143  0 out of 14 good  Bayesian Score: -1.671 | | |  | | --- | |  | | B15: 2085698692  0 out of 14 good  Bayesian Score: -1.671 | |
| |  | | --- | |  | | B16: -845108448  0 out of 14 good  Bayesian Score: -1.671 | | |  | | --- | |  | | B17: -91640731  0 out of 14 good  Bayesian Score: -1.671 | | |  | | --- | |  | | B18: -177935549  0 out of 14 good  Bayesian Score: -1.671 | | |  | | --- | |  | | B19: 859433814  0 out of 14 good  Bayesian Score: -1.671 | | |  | | --- | |  | | B20: -709633021  0 out of 13 good  Bayesian Score: -1.611 | |
