# Supplementary material for: Identification of Multi-Target Anti-AD Chemical Constituents From Traditional Chinese Medicine Formulae by Integrating Virtual Screening and In Vitro Validation
Source: Front Pharmacol. 2021 Jul 16;12:709607. doi: 10.3389/fphar.2021.709607 (PMC8322649; doi:10.3389/fphar.2021.709607)
Supplement: Supplementary file 3 [file DataSheet1.ZIP › Good and bad fragments of 52 targets/PPARG.html]

Category NB\_ppargamma-ECFP6: good features from ECFP\_6

|  |  |  |  |  |  |  |  |  |  |  |  |  |  |  |
| --- | --- | --- | --- | --- | --- | --- | --- | --- | --- | --- | --- | --- | --- | --- |
| |  | | --- | |  | | G1: 1631004977  387 out of 387 good  Bayesian Score: 1.148 | | |  | | --- | |  | | G2: -1577433219  375 out of 375 good  Bayesian Score: 1.148 | | |  | | --- | |  | | G3: 131834252  367 out of 367 good  Bayesian Score: 1.148 | | |  | | --- | |  | | G4: -877343146  356 out of 356 good  Bayesian Score: 1.148 | | |  | | --- | |  | | G5: -802835384  344 out of 344 good  Bayesian Score: 1.147 | |
| |  | | --- | |  | | G6: -898800149  330 out of 330 good  Bayesian Score: 1.147 | | |  | | --- | |  | | G7: 1433040077  327 out of 327 good  Bayesian Score: 1.147 | | |  | | --- | |  | | G8: 1062410947  319 out of 319 good  Bayesian Score: 1.147 | | |  | | --- | |  | | G9: 1609862462  296 out of 296 good  Bayesian Score: 1.146 | | |  | | --- | |  | | G10: 327687054  288 out of 288 good  Bayesian Score: 1.146 | |
| |  | | --- | |  | | G11: -1682850076  283 out of 283 good  Bayesian Score: 1.146 | | |  | | --- | |  | | G12: -1896172266  282 out of 282 good  Bayesian Score: 1.146 | | |  | | --- | |  | | G13: -1925054681  280 out of 280 good  Bayesian Score: 1.146 | | |  | | --- | |  | | G14: -1085710210  252 out of 252 good  Bayesian Score: 1.145 | | |  | | --- | |  | | G15: 740177721  200 out of 200 good  Bayesian Score: 1.143 | |
| |  | | --- | |  | | G16: 375648709  196 out of 196 good  Bayesian Score: 1.143 | | |  | | --- | |  | | G17: -1241724113  194 out of 194 good  Bayesian Score: 1.143 | | |  | | --- | |  | | G18: 376849325  191 out of 191 good  Bayesian Score: 1.142 | | |  | | --- | |  | | G19: -755304676  189 out of 189 good  Bayesian Score: 1.142 | | |  | | --- | |  | | G20: -174137070  172 out of 172 good  Bayesian Score: 1.141 | |

Category NB\_ppargamma-ECFP6: bad features from ECFP\_6

|  |  |  |  |  |  |  |  |  |  |  |  |  |  |  |
| --- | --- | --- | --- | --- | --- | --- | --- | --- | --- | --- | --- | --- | --- | --- |
| |  | | --- | |  | | B1: -1832102709  0 out of 255 good  Bayesian Score: -4.400 | | |  | | --- | |  | | B2: 544048674  0 out of 161 good  Bayesian Score: -3.947 | | |  | | --- | |  | | B3: -1508366470  0 out of 157 good  Bayesian Score: -3.923 | | |  | | --- | |  | | B4: 1814278164  0 out of 153 good  Bayesian Score: -3.897 | | |  | | --- | |  | | B5: -1956535100  0 out of 143 good  Bayesian Score: -3.831 | |
| |  | | --- | |  | | B6: -1742225957  0 out of 143 good  Bayesian Score: -3.831 | | |  | | --- | |  | | B7: 1986731747  0 out of 126 good  Bayesian Score: -3.707 | | |  | | --- | |  | | B8: 600440273  0 out of 109 good  Bayesian Score: -3.566 | | |  | | --- | |  | | B9: -1596132236  0 out of 106 good  Bayesian Score: -3.539 | | |  | | --- | |  | | B10: -215026467  3 out of 405 good  Bayesian Score: -3.472 | |
| |  | | --- | |  | | B11: -1597579789  0 out of 94 good  Bayesian Score: -3.423 | | |  | | --- | |  | | B12: -1665306562  0 out of 93 good  Bayesian Score: -3.412 | | |  | | --- | |  | | B13: -1843377874  0 out of 93 good  Bayesian Score: -3.412 | | |  | | --- | |  | | B14: -851770808  0 out of 92 good  Bayesian Score: -3.402 | | |  | | --- | |  | | B15: 1525531932  0 out of 91 good  Bayesian Score: -3.391 | |
| |  | | --- | |  | | B16: -1236714312  0 out of 89 good  Bayesian Score: -3.370 | | |  | | --- | |  | | B17: -955816473  1 out of 176 good  Bayesian Score: -3.341 | | |  | | --- | |  | | B18: -1507082173  0 out of 85 good  Bayesian Score: -3.326 | | |  | | --- | |  | | B19: -306186198  0 out of 80 good  Bayesian Score: -3.267 | | |  | | --- | |  | | B20: -1715064478  0 out of 80 good  Bayesian Score: -3.267 | |
