# Supplementary material for: Identification of Multi-Target Anti-AD Chemical Constituents From Traditional Chinese Medicine Formulae by Integrating Virtual Screening and In Vitro Validation
Source: Front Pharmacol. 2021 Jul 16;12:709607. doi: 10.3389/fphar.2021.709607 (PMC8322649; doi:10.3389/fphar.2021.709607)
Supplement: Supplementary file 3 [file DataSheet1.ZIP › Good and bad fragments of 52 targets/PPID.html]

Category NB\_Cathepsin d\_ECFP6: good features from ECFP\_6

|  |  |  |  |  |  |  |  |  |  |  |  |  |  |  |
| --- | --- | --- | --- | --- | --- | --- | --- | --- | --- | --- | --- | --- | --- | --- |
| |  | | --- | |  | | G1: 1334840514  728 out of 728 good  Bayesian Score: 1.059 | | |  | | --- | |  | | G2: 3228927  423 out of 423 good  Bayesian Score: 1.057 | | |  | | --- | |  | | G3: 766511464  414 out of 414 good  Bayesian Score: 1.057 | | |  | | --- | |  | | G4: -1919583272  356 out of 356 good  Bayesian Score: 1.057 | | |  | | --- | |  | | G5: 571758812  308 out of 308 good  Bayesian Score: 1.056 | |
| |  | | --- | |  | | G6: 451953143  300 out of 300 good  Bayesian Score: 1.056 | | |  | | --- | |  | | G7: -1342559370  291 out of 291 good  Bayesian Score: 1.055 | | |  | | --- | |  | | G8: -1843371804  219 out of 219 good  Bayesian Score: 1.053 | | |  | | --- | |  | | G9: 1821159565  176 out of 176 good  Bayesian Score: 1.051 | | |  | | --- | |  | | G10: -1919756505  174 out of 174 good  Bayesian Score: 1.051 | |
| |  | | --- | |  | | G11: -918178086  169 out of 169 good  Bayesian Score: 1.051 | | |  | | --- | |  | | G12: 358083482  169 out of 169 good  Bayesian Score: 1.051 | | |  | | --- | |  | | G13: 78733088  701 out of 707 good  Bayesian Score: 1.051 | | |  | | --- | |  | | G14: 185828173  161 out of 161 good  Bayesian Score: 1.050 | | |  | | --- | |  | | G15: -939930615  160 out of 160 good  Bayesian Score: 1.050 | |
| |  | | --- | |  | | G16: 2080306479  158 out of 158 good  Bayesian Score: 1.050 | | |  | | --- | |  | | G17: 150625520  149 out of 149 good  Bayesian Score: 1.049 | | |  | | --- | |  | | G18: 301002615  149 out of 149 good  Bayesian Score: 1.049 | | |  | | --- | |  | | G19: -1205575950  141 out of 141 good  Bayesian Score: 1.049 | | |  | | --- | |  | | G20: 673547498  136 out of 136 good  Bayesian Score: 1.048 | |

Category NB\_Cathepsin d\_ECFP6: bad features from ECFP\_6

|  |  |  |  |  |  |  |  |  |  |  |  |  |  |  |
| --- | --- | --- | --- | --- | --- | --- | --- | --- | --- | --- | --- | --- | --- | --- |
| |  | | --- | |  | | B1: -1087070950  0 out of 595 good  Bayesian Score: -5.332 | | |  | | --- | |  | | B2: -1832102709  0 out of 218 good  Bayesian Score: -4.336 | | |  | | --- | |  | | B3: -813997308  0 out of 174 good  Bayesian Score: -4.114 | | |  | | --- | |  | | B4: -962771238  0 out of 136 good  Bayesian Score: -3.872 | | |  | | --- | |  | | B5: -737616849  0 out of 133 good  Bayesian Score: -3.850 | |
| |  | | --- | |  | | B6: 544048674  0 out of 120 good  Bayesian Score: -3.749 | | |  | | --- | |  | | B7: -1699286547  1 out of 239 good  Bayesian Score: -3.733 | | |  | | --- | |  | | B8: -1517027220  0 out of 112 good  Bayesian Score: -3.682 | | |  | | --- | |  | | B9: 2092245922  0 out of 110 good  Bayesian Score: -3.665 | | |  | | --- | |  | | B10: 1814278164  0 out of 106 good  Bayesian Score: -3.628 | |
| |  | | --- | |  | | B11: 558201926  0 out of 103 good  Bayesian Score: -3.601 | | |  | | --- | |  | | B12: 1974911681  0 out of 101 good  Bayesian Score: -3.581 | | |  | | --- | |  | | B13: -1508366470  0 out of 100 good  Bayesian Score: -3.572 | | |  | | --- | |  | | B14: 1717462980  0 out of 99 good  Bayesian Score: -3.562 | | |  | | --- | |  | | B15: -2137232509  0 out of 96 good  Bayesian Score: -3.532 | |
| |  | | --- | |  | | B16: -175376949  0 out of 94 good  Bayesian Score: -3.512 | | |  | | --- | |  | | B17: -1236714312  0 out of 84 good  Bayesian Score: -3.403 | | |  | | --- | |  | | B18: -438821815  0 out of 70 good  Bayesian Score: -3.227 | | |  | | --- | |  | | B19: -1364059178  0 out of 66 good  Bayesian Score: -3.171 | | |  | | --- | |  | | B20: 455570479  0 out of 65 good  Bayesian Score: -3.156 | |
