# Supplementary material for: Identification of Multi-Target Anti-AD Chemical Constituents From Traditional Chinese Medicine Formulae by Integrating Virtual Screening and In Vitro Validation
Source: Front Pharmacol. 2021 Jul 16;12:709607. doi: 10.3389/fphar.2021.709607 (PMC8322649; doi:10.3389/fphar.2021.709607)
Supplement: Supplementary file 3 [file DataSheet1.ZIP › Good and bad fragments of 52 targets/PSEN1.html]

Category Bayesian-gamma-secretase: good features from ECFP\_6

|  |  |  |  |  |  |  |  |  |  |  |  |  |  |  |
| --- | --- | --- | --- | --- | --- | --- | --- | --- | --- | --- | --- | --- | --- | --- |
| |  | | --- | |  | | G1: -979993137  109 out of 109 good  Bayesian Score: 1.259 | | |  | | --- | |  | | G2: -695637100  69 out of 69 good  Bayesian Score: 1.246 | | |  | | --- | |  | | G3: -1595829635  63 out of 63 good  Bayesian Score: 1.243 | | |  | | --- | |  | | G4: 676683999  63 out of 63 good  Bayesian Score: 1.243 | | |  | | --- | |  | | G5: -523859409  62 out of 62 good  Bayesian Score: 1.242 | |
| |  | | --- | |  | | G6: -1326864213  56 out of 56 good  Bayesian Score: 1.238 | | |  | | --- | |  | | G7: 1099306479  56 out of 56 good  Bayesian Score: 1.238 | | |  | | --- | |  | | G8: 2131696424  55 out of 55 good  Bayesian Score: 1.237 | | |  | | --- | |  | | G9: -1079363164  54 out of 54 good  Bayesian Score: 1.236 | | |  | | --- | |  | | G10: 1798853767  54 out of 54 good  Bayesian Score: 1.236 | |
| |  | | --- | |  | | G11: 89776398  54 out of 54 good  Bayesian Score: 1.236 | | |  | | --- | |  | | G12: -1251617187  54 out of 54 good  Bayesian Score: 1.236 | | |  | | --- | |  | | G13: -1955729072  54 out of 54 good  Bayesian Score: 1.236 | | |  | | --- | |  | | G14: 2089162910  54 out of 54 good  Bayesian Score: 1.236 | | |  | | --- | |  | | G15: -1598085323  54 out of 54 good  Bayesian Score: 1.236 | |
| |  | | --- | |  | | G16: -265719538  54 out of 54 good  Bayesian Score: 1.236 | | |  | | --- | |  | | G17: 945431654  49 out of 49 good  Bayesian Score: 1.232 | | |  | | --- | |  | | G18: -1685106965  46 out of 46 good  Bayesian Score: 1.229 | | |  | | --- | |  | | G19: 1334840514  44 out of 44 good  Bayesian Score: 1.226 | | |  | | --- | |  | | G20: 619424271  43 out of 43 good  Bayesian Score: 1.225 | |

Category Bayesian-gamma-secretase: bad features from ECFP\_6

|  |  |  |  |  |  |  |  |  |  |  |  |  |  |  |
| --- | --- | --- | --- | --- | --- | --- | --- | --- | --- | --- | --- | --- | --- | --- |
| |  | | --- | |  | | B1: 834876373  0 out of 157 good  Bayesian Score: -3.796 | | |  | | --- | |  | | B2: -830332112  0 out of 105 good  Bayesian Score: -3.405 | | |  | | --- | |  | | B3: -302078100  2 out of 234 good  Bayesian Score: -3.089 | | |  | | --- | |  | | B4: -709633021  0 out of 71 good  Bayesian Score: -3.030 | | |  | | --- | |  | | B5: -1087070950  0 out of 71 good  Bayesian Score: -3.030 | |
| |  | | --- | |  | | B6: 459826767  2 out of 219 good  Bayesian Score: -3.024 | | |  | | --- | |  | | B7: 1334250623  0 out of 70 good  Bayesian Score: -3.016 | | |  | | --- | |  | | B8: 781519895  0 out of 62 good  Bayesian Score: -2.901 | | |  | | --- | |  | | B9: 859796174  3 out of 241 good  Bayesian Score: -2.831 | | |  | | --- | |  | | B10: -845108448  0 out of 56 good  Bayesian Score: -2.805 | |
| |  | | --- | |  | | B11: -224638920  0 out of 56 good  Bayesian Score: -2.805 | | |  | | --- | |  | | B12: 1427820655  0 out of 54 good  Bayesian Score: -2.771 | | |  | | --- | |  | | B13: 1410041175  0 out of 50 good  Bayesian Score: -2.699 | | |  | | --- | |  | | B14: 1959277503  0 out of 50 good  Bayesian Score: -2.699 | | |  | | --- | |  | | B15: 2077607946  0 out of 49 good  Bayesian Score: -2.680 | |
| |  | | --- | |  | | B16: -177935549  2 out of 149 good  Bayesian Score: -2.647 | | |  | | --- | |  | | B17: 1845080228  0 out of 45 good  Bayesian Score: -2.601 | | |  | | --- | |  | | B18: 1335108269  0 out of 45 good  Bayesian Score: -2.601 | | |  | | --- | |  | | B19: 1961554343  0 out of 45 good  Bayesian Score: -2.601 | | |  | | --- | |  | | B20: 1639858918  0 out of 44 good  Bayesian Score: -2.580 | |
