# Supplementary material for: Identification of Multi-Target Anti-AD Chemical Constituents From Traditional Chinese Medicine Formulae by Integrating Virtual Screening and In Vitro Validation
Source: Front Pharmacol. 2021 Jul 16;12:709607. doi: 10.3389/fphar.2021.709607 (PMC8322649; doi:10.3389/fphar.2021.709607)
Supplement: Supplementary file 3 [file DataSheet1.ZIP › Good and bad fragments of 52 targets/PTGS2.html]

Category NB\_COX2\_ECFP6: good features from ECFP\_6

|  |  |  |  |  |  |  |  |  |  |  |  |  |  |  |
| --- | --- | --- | --- | --- | --- | --- | --- | --- | --- | --- | --- | --- | --- | --- |
| |  | | --- | |  | | G1: -934226723  380 out of 380 good  Bayesian Score: 1.465 | | |  | | --- | |  | | G2: -2121766239  376 out of 376 good  Bayesian Score: 1.465 | | |  | | --- | |  | | G3: -908165145  354 out of 354 good  Bayesian Score: 1.464 | | |  | | --- | |  | | G4: -1261213389  352 out of 352 good  Bayesian Score: 1.464 | | |  | | --- | |  | | G5: 107868251  352 out of 352 good  Bayesian Score: 1.464 | |
| |  | | --- | |  | | G6: 550321960  168 out of 168 good  Bayesian Score: 1.454 | | |  | | --- | |  | | G7: -2125968768  162 out of 162 good  Bayesian Score: 1.453 | | |  | | --- | |  | | G8: 551008207  147 out of 147 good  Bayesian Score: 1.451 | | |  | | --- | |  | | G9: 159494548  145 out of 145 good  Bayesian Score: 1.451 | | |  | | --- | |  | | G10: 1801843491  140 out of 140 good  Bayesian Score: 1.450 | |
| |  | | --- | |  | | G11: 1802529738  553 out of 565 good  Bayesian Score: 1.446 | | |  | | --- | |  | | G12: 107498356  755 out of 773 good  Bayesian Score: 1.446 | | |  | | --- | |  | | G13: 1753564049  755 out of 773 good  Bayesian Score: 1.446 | | |  | | --- | |  | | G14: 1401597923  117 out of 117 good  Bayesian Score: 1.446 | | |  | | --- | |  | | G15: 1275580254  116 out of 116 good  Bayesian Score: 1.445 | |
| |  | | --- | |  | | G16: 1004577400  113 out of 113 good  Bayesian Score: 1.445 | | |  | | --- | |  | | G17: -593615377  113 out of 113 good  Bayesian Score: 1.445 | | |  | | --- | |  | | G18: 1278384076  112 out of 112 good  Bayesian Score: 1.444 | | |  | | --- | |  | | G19: -420882087  101 out of 101 good  Bayesian Score: 1.441 | | |  | | --- | |  | | G20: -1185319784  101 out of 101 good  Bayesian Score: 1.441 | |

Category NB\_COX2\_ECFP6: bad features from ECFP\_6

|  |  |  |  |  |  |  |  |  |  |  |  |  |  |  |
| --- | --- | --- | --- | --- | --- | --- | --- | --- | --- | --- | --- | --- | --- | --- |
| |  | | --- | |  | | B1: -1813180068  0 out of 325 good  Bayesian Score: -4.323 | | |  | | --- | |  | | B2: -1686813061  0 out of 322 good  Bayesian Score: -4.314 | | |  | | --- | |  | | B3: -1715064478  0 out of 259 good  Bayesian Score: -4.100 | | |  | | --- | |  | | B4: 1526392165  0 out of 254 good  Bayesian Score: -4.081 | | |  | | --- | |  | | B5: 1961021054  0 out of 254 good  Bayesian Score: -4.081 | |
| |  | | --- | |  | | B6: 1986731747  0 out of 253 good  Bayesian Score: -4.077 | | |  | | --- | |  | | B7: 999904819  0 out of 249 good  Bayesian Score: -4.061 | | |  | | --- | |  | | B8: -37698365  0 out of 207 good  Bayesian Score: -3.880 | | |  | | --- | |  | | B9: -1486887252  0 out of 205 good  Bayesian Score: -3.870 | | |  | | --- | |  | | B10: 1657199878  0 out of 192 good  Bayesian Score: -3.806 | |
| |  | | --- | |  | | B11: -1513679478  0 out of 187 good  Bayesian Score: -3.780 | | |  | | --- | |  | | B12: 2077298510  0 out of 184 good  Bayesian Score: -3.765 | | |  | | --- | |  | | B13: 953616030  0 out of 177 good  Bayesian Score: -3.727 | | |  | | --- | |  | | B14: -91954924  0 out of 175 good  Bayesian Score: -3.716 | | |  | | --- | |  | | B15: -1030734259  0 out of 153 good  Bayesian Score: -3.585 | |
| |  | | --- | |  | | B16: 901105631  0 out of 148 good  Bayesian Score: -3.552 | | |  | | --- | |  | | B17: -1504438789  0 out of 144 good  Bayesian Score: -3.526 | | |  | | --- | |  | | B18: 1925815814  0 out of 144 good  Bayesian Score: -3.526 | | |  | | --- | |  | | B19: 780656908  0 out of 143 good  Bayesian Score: -3.519 | | |  | | --- | |  | | B20: 1986657572  0 out of 141 good  Bayesian Score: -3.505 | |
