# Supplementary material for: Identification of Multi-Target Anti-AD Chemical Constituents From Traditional Chinese Medicine Formulae by Integrating Virtual Screening and In Vitro Validation
Source: Front Pharmacol. 2021 Jul 16;12:709607. doi: 10.3389/fphar.2021.709607 (PMC8322649; doi:10.3389/fphar.2021.709607)
Supplement: Supplementary file 3 [file DataSheet1.ZIP › Good and bad fragments of 52 targets/SIGMAR1.html]

Category Bayesian-Sigma-1: good features from ECFP\_6

|  |  |  |  |  |  |  |  |  |  |  |  |  |  |  |
| --- | --- | --- | --- | --- | --- | --- | --- | --- | --- | --- | --- | --- | --- | --- |
| |  | | --- | |  | | G1: 733797433  167 out of 167 good  Bayesian Score: 1.348 | | |  | | --- | |  | | G2: -835787320  156 out of 156 good  Bayesian Score: 1.347 | | |  | | --- | |  | | G3: 1422674680  182 out of 183 good  Bayesian Score: 1.344 | | |  | | --- | |  | | G4: -1602360129  116 out of 116 good  Bayesian Score: 1.340 | | |  | | --- | |  | | G5: -1811250099  115 out of 115 good  Bayesian Score: 1.340 | |
| |  | | --- | |  | | G6: 377876847  114 out of 114 good  Bayesian Score: 1.340 | | |  | | --- | |  | | G7: 992919368  103 out of 103 good  Bayesian Score: 1.337 | | |  | | --- | |  | | G8: -541070345  174 out of 176 good  Bayesian Score: 1.337 | | |  | | --- | |  | | G9: -364688247  97 out of 97 good  Bayesian Score: 1.336 | | |  | | --- | |  | | G10: 485677615  95 out of 95 good  Bayesian Score: 1.335 | |
| |  | | --- | |  | | G11: -1340287910  95 out of 95 good  Bayesian Score: 1.335 | | |  | | --- | |  | | G12: -1568703800  140 out of 142 good  Bayesian Score: 1.331 | | |  | | --- | |  | | G13: -67018160  166 out of 170 good  Bayesian Score: 1.325 | | |  | | --- | |  | | G14: 1746328571  65 out of 65 good  Bayesian Score: 1.322 | | |  | | --- | |  | | G15: -653777476  65 out of 65 good  Bayesian Score: 1.322 | |
| |  | | --- | |  | | G16: 1425031356  65 out of 65 good  Bayesian Score: 1.322 | | |  | | --- | |  | | G17: -2061744983  65 out of 65 good  Bayesian Score: 1.322 | | |  | | --- | |  | | G18: -29580765  64 out of 64 good  Bayesian Score: 1.321 | | |  | | --- | |  | | G19: 681865297  128 out of 131 good  Bayesian Score: 1.320 | | |  | | --- | |  | | G20: 610708696  56 out of 56 good  Bayesian Score: 1.315 | |

Category Bayesian-Sigma-1: bad features from ECFP\_6

|  |  |  |  |  |  |  |  |  |  |  |  |  |  |  |
| --- | --- | --- | --- | --- | --- | --- | --- | --- | --- | --- | --- | --- | --- | --- |
| |  | | --- | |  | | B1: 1961554343  0 out of 656 good  Bayesian Score: -5.127 | | |  | | --- | |  | | B2: 1994668215  0 out of 249 good  Bayesian Score: -4.168 | | |  | | --- | |  | | B3: -666326105  0 out of 186 good  Bayesian Score: -3.881 | | |  | | --- | |  | | B4: 2116455019  0 out of 185 good  Bayesian Score: -3.876 | | |  | | --- | |  | | B5: 2116709167  0 out of 137 good  Bayesian Score: -3.583 | |
| |  | | --- | |  | | B6: -788112909  0 out of 121 good  Bayesian Score: -3.463 | | |  | | --- | |  | | B7: -177077903  0 out of 116 good  Bayesian Score: -3.422 | | |  | | --- | |  | | B8: 1331561287  0 out of 101 good  Bayesian Score: -3.288 | | |  | | --- | |  | | B9: 834876373  2 out of 286 good  Bayesian Score: -3.206 | | |  | | --- | |  | | B10: 1335340087  0 out of 83 good  Bayesian Score: -3.100 | |
| |  | | --- | |  | | B11: 908605940  0 out of 77 good  Bayesian Score: -3.028 | | |  | | --- | |  | | B12: 769925792  0 out of 72 good  Bayesian Score: -2.965 | | |  | | --- | |  | | B13: 888054369  0 out of 72 good  Bayesian Score: -2.965 | | |  | | --- | |  | | B14: 2008088114  0 out of 70 good  Bayesian Score: -2.938 | | |  | | --- | |  | | B15: 226796801  1 out of 138 good  Bayesian Score: -2.897 | |
| |  | | --- | |  | | B16: 300955665  0 out of 67 good  Bayesian Score: -2.896 | | |  | | --- | |  | | B17: 511517215  0 out of 66 good  Bayesian Score: -2.882 | | |  | | --- | |  | | B18: 259395744  0 out of 66 good  Bayesian Score: -2.882 | | |  | | --- | |  | | B19: 1640603662  0 out of 64 good  Bayesian Score: -2.853 | | |  | | --- | |  | | B20: 1997522062  1 out of 130 good  Bayesian Score: -2.839 | |
