# Supplementary material for: Identification of Multi-Target Anti-AD Chemical Constituents From Traditional Chinese Medicine Formulae by Integrating Virtual Screening and In Vitro Validation
Source: Front Pharmacol. 2021 Jul 16;12:709607. doi: 10.3389/fphar.2021.709607 (PMC8322649; doi:10.3389/fphar.2021.709607)
Supplement: Supplementary file 3 [file DataSheet1.ZIP › Good and bad fragments of 52 targets/TNF.html]

Category NB\_TNF-alpha-ECFP6: good features from ECFP\_6

|  |  |  |  |  |  |  |  |  |  |  |  |  |  |  |
| --- | --- | --- | --- | --- | --- | --- | --- | --- | --- | --- | --- | --- | --- | --- |
| |  | | --- | |  | | G1: 1106862477  271 out of 271 good  Bayesian Score: 1.170 | | |  | | --- | |  | | G2: -135622333  271 out of 271 good  Bayesian Score: 1.170 | | |  | | --- | |  | | G3: -404386182  271 out of 271 good  Bayesian Score: 1.170 | | |  | | --- | |  | | G4: -2127032407  271 out of 271 good  Bayesian Score: 1.170 | | |  | | --- | |  | | G5: -1770440234  271 out of 271 good  Bayesian Score: 1.170 | |
| |  | | --- | |  | | G6: 54860204  271 out of 271 good  Bayesian Score: 1.170 | | |  | | --- | |  | | G7: -1315925926  271 out of 271 good  Bayesian Score: 1.170 | | |  | | --- | |  | | G8: 471594083  270 out of 270 good  Bayesian Score: 1.170 | | |  | | --- | |  | | G9: 928810548  270 out of 270 good  Bayesian Score: 1.170 | | |  | | --- | |  | | G10: 1816665433  270 out of 270 good  Bayesian Score: 1.170 | |
| |  | | --- | |  | | G11: 1512662480  270 out of 270 good  Bayesian Score: 1.170 | | |  | | --- | |  | | G12: 993992616  270 out of 270 good  Bayesian Score: 1.170 | | |  | | --- | |  | | G13: -1555945794  269 out of 269 good  Bayesian Score: 1.170 | | |  | | --- | |  | | G14: -1966517806  269 out of 269 good  Bayesian Score: 1.170 | | |  | | --- | |  | | G15: -1525794803  268 out of 268 good  Bayesian Score: 1.170 | |
| |  | | --- | |  | | G16: -887798013  218 out of 218 good  Bayesian Score: 1.168 | | |  | | --- | |  | | G17: 451519819  218 out of 218 good  Bayesian Score: 1.168 | | |  | | --- | |  | | G18: 378973763  217 out of 217 good  Bayesian Score: 1.168 | | |  | | --- | |  | | G19: -2016387687  217 out of 217 good  Bayesian Score: 1.168 | | |  | | --- | |  | | G20: 2071945256  217 out of 217 good  Bayesian Score: 1.168 | |

Category NB\_TNF-alpha-ECFP6: bad features from ECFP\_6

|  |  |  |  |  |  |  |  |  |  |  |  |  |  |  |
| --- | --- | --- | --- | --- | --- | --- | --- | --- | --- | --- | --- | --- | --- | --- |
| |  | | --- | |  | | B1: -1950934120  0 out of 113 good  Bayesian Score: -3.577 | | |  | | --- | |  | | B2: -655344035  0 out of 105 good  Bayesian Score: -3.506 | | |  | | --- | |  | | B3: 1976330679  0 out of 102 good  Bayesian Score: -3.478 | | |  | | --- | |  | | B4: -244159614  0 out of 86 good  Bayesian Score: -3.313 | | |  | | --- | |  | | B5: 768590601  0 out of 84 good  Bayesian Score: -3.290 | |
| |  | | --- | |  | | B6: 200172372  0 out of 71 good  Bayesian Score: -3.129 | | |  | | --- | |  | | B7: 1951769206  0 out of 69 good  Bayesian Score: -3.102 | | |  | | --- | |  | | B8: -661766797  0 out of 66 good  Bayesian Score: -3.059 | | |  | | --- | |  | | B9: 1961554343  0 out of 64 good  Bayesian Score: -3.030 | | |  | | --- | |  | | B10: 1133499173  0 out of 61 good  Bayesian Score: -2.984 | |
| |  | | --- | |  | | B11: -232258228  0 out of 58 good  Bayesian Score: -2.936 | | |  | | --- | |  | | B12: -572965350  0 out of 49 good  Bayesian Score: -2.777 | | |  | | --- | |  | | B13: 294288814  0 out of 48 good  Bayesian Score: -2.758 | | |  | | --- | |  | | B14: 975766354  0 out of 47 good  Bayesian Score: -2.738 | | |  | | --- | |  | | B15: -591526139  0 out of 42 good  Bayesian Score: -2.634 | |
| |  | | --- | |  | | B16: 233520344  0 out of 40 good  Bayesian Score: -2.588 | | |  | | --- | |  | | B17: -2091421151  0 out of 40 good  Bayesian Score: -2.588 | | |  | | --- | |  | | B18: -1868297614  0 out of 39 good  Bayesian Score: -2.565 | | |  | | --- | |  | | B19: -857882738  0 out of 39 good  Bayesian Score: -2.565 | | |  | | --- | |  | | B20: 469398259  0 out of 38 good  Bayesian Score: -2.541 | |
