# Supplementary material for: Identification of Multi-Target Anti-AD Chemical Constituents From Traditional Chinese Medicine Formulae by Integrating Virtual Screening and In Vitro Validation
Source: Front Pharmacol. 2021 Jul 16;12:709607. doi: 10.3389/fphar.2021.709607 (PMC8322649; doi:10.3389/fphar.2021.709607)
Supplement: Supplementary file 4 [file Table2.DOCX]

| No. | Compound structure |
| --- | --- |
| 1 | CC(=O)OC[C@H]1O[C@@H](O[C@H]2OC=C3[C@H]4[C@@H](OC3=O)C=C(COC(=O)C)[C@@H]24)[C@H](OC(=O)C)[C@@H](OC(=O)C)[C@@H]1OC(=O)C |
| 2 | COc1cc(OC)c2C(=O)c3cc(O)ccc3C(=O)c2c1O |
| 3 | COc1cc(OC)c2C(=O)c3cc(O)ccc3C(=O)c2c1[O-] |
| 4 | COc1ccc2C(=O)c3cc(O)ccc3C(=O)c2c1O |
| 5 | COc1ccc2C(=O)c3cc(O)ccc3C(=O)c2c1[O-] |
| 6 | Oc1cccc2C(=O)c3ccccc3C(=O)c12 |
| 7 | [O-]c1cccc2C(=O)c3ccccc3C(=O)c12 |
| 8 | OCc1cc2C(=O)c3ccccc3C(=O)c2cc1O |
| 9 | Cc1ccc2C(=O)c3ccccc3C(=O)c2c1O |
| 10 | Cc1ccc2C(=O)c3ccccc3C(=O)c2c1[O-] |
| 11 | OC[C@H]1O[C@@H](O[C@@H]2OC=C([C@H]3C=C[C@](O)(CO)[C@@H]23)C(=O)[O-])[C@H](O)[C@@H](O)[C@@H]1O |
| 12 | Cc1ccc2C(=O)c3c(O)c(O[C@@H]4O[C@H](CO[C@@H]5OC[C@@H](O)[C@H](O)[C@H]5O)[C@@H](O)[C@H](O)[C@H]4O)ccc3C(=O)c2c1O |
| 13 | Cc1ccc2C(=O)c3c([O-])c(O[C@@H]4O[C@H](CO[C@@H]5OC[C@@H](O)[C@H](O)[C@H]5O)[C@@H](O)[C@H](O)[C@H]4O)ccc3C(=O)c2c1O |
| 14 | Cc1ccc2C(=O)c3c([O-])c(O[C@@H]4O[C@H](CO[C@@H]5OC[C@@H](O)[C@H](O)[C@H]5O)[C@@H](O)[C@H](O)[C@H]4O)ccc3C(=O)c2c1[O-] |
| 15 | CCCCO[C@@]12OC[C@@H](O)[C@H](O)[C@]1(CO)[C@@H]2O |
| 16 | COc1cc(O)c2C(=O)c3c(O)cc(C)cc3C(=O)c2c1 |
| 17 | COc1cc([O-])c2C(=O)c3c(O)cc(C)cc3C(=O)c2c1 |
| 18 | COc1cc([O-])c2C(=O)c3c([O-])cc(C)cc3C(=O)c2c1 |
| 19 | CC[C@H](CC[C@@H](C)[C@H]1CC[C@H]2[C@@H]3CC=C4C[C@@H](O)CC[C@]4(C)[C@H]3CC[C@]12C)C(C)C |
| 20 | C[C@H](CCC=C(C)C)c1ccc(C)cc1 |
| 21 | COc1cc2C=CC(=O)Oc2cc1[O-] |
| 22 | CCCCCCCCCCCCCCCC(=O)[O-] |
| 23 | CCCCCCCC\C=C\C=O |
| 24 | CCCCCCCCC=O |
| 25 | CC(C)c1ccc(C)cc1 |
| 26 | CC1=CC[C@H](CC1)C(C)(C)O |
| 27 | CC(=CCC\C(=C\CO)\C)C |
| 28 | CC1=CC[C@H]2C[C@@H]1C2(C)C |
| 29 | CCCCC\C=C/C\C=C/CCCCCCCC(=O)[O-] |
| 30 | CC(=O)O[C@@H]1C[C@@H]2CC[C@@]1(C)C2(C)C |
| 31 | CC(=CCC[C@@](C)(O)C=C)C |
| 32 | Oc1ccc(\C=C\C(=O)[O-])cc1O |
| 33 | CC1=CC[C@@H](CC1)C(C)(C)O |
| 34 | CC1(C)[C@@H]2CC[C@@]1(C)[C@@H](O)C2 |
| 35 | CCCCCCCC(=O)[O-] |
| 36 | CCCCCCCCCCCC(=O)[O-] |
| 37 | [O-]C(=O)CCC(=O)[O-] |
| 38 | CC[C@H](CC[C@@H](C)[C@H]1CC[C@H]2[C@@H]3CC=C4C[C@H](O)CC[C@]4(C)[C@H]3CC[C@]12C)C(C)C |
| 39 | CC(C)[C@]1(O)CCC(=CC1)C |
| 40 | CCCCCCCCCCCCCCCCCCCCCCCC(=O)[O-] |
| 41 | CCCCCCO |
| 42 | CCCCCc1occc1 |
| 43 | CC1(C)[C@H]2CC[C@]1(C)C(=O)C2 |
| 44 | CCCCCCCC\C=C/CCCCCCCC(=O)[O-] |
| 45 | CCCCOC(=O)c1ccccc1C(=O)OCCCC |
| 46 | CCCCCC(=O)C |
| 47 | CCCCCCC=O |
| 48 | CCCC\C=C\C=O |
| 49 | O=Cc1ccccc1 |
| 50 | CCCCCCCC=O |
| 51 | O=CCc1ccccc1 |
| 52 | CCOC(=O)C |
| 53 | CCCCCCCCCCCCCCCCCC(=O)[O-] |
| 54 | CCCCCCCCCCCCCCCCC |
| 55 | C[C@@H]1CC[C@H]2C(C)(C)[C@H]3C[C@@]12CC[C@@]3(C)O |
| 56 | CCCC(C)(C)c1ccccc1 |
| 57 | CCCCCCCCCCCC |
| 58 | CCCCCCCCCCCCCC |
| 59 | C[C@@H](CCC=C(C)C)[C@H]1CC=C(C)C=C1 |
| 60 | CCCCCCCCCC(=O)C |
| 61 | CCCCCCCCCCCCCCCC(=O)OCC |
| 62 | CCCCCCCCO |
| 63 | OCCc1ccccc1 |
| 64 | OCc1ccccc1 |
| 65 | CCCCCCCCCCCCCC(=O)[O-] |
| 66 | CCCCCCCCCCCCCCCCCC |
| 67 | CCCCCCCCCCCCCCC(=O)[O-] |
| 68 | CCCCC\C=C\C=O |
| 69 | OC(CC(=O)[O-])(CC(=O)[O-])C(=O)[O-] |
| 70 | CCCCCCCCCCCCCCC(=O)OCC |
| 71 | CC(=CCC\C(=C\CC\C(=C\CC\C=C(/C)\CC\C=C(/C)\CCC=C(C)C)\C)\C)C |
| 72 | CCCCCCCCCCCCCCCCCCC |
| 73 | CCCCCCCCCC(=O)[O-] |
| 74 | Cc1cc2C(=O)c3ccccc3C(=O)c2cc1O |
| 75 | CC(=O)OCC1=C[C@@H]2OC(=O)C3=CO[C@@H](O[C@@H]4O[C@H](CO)[C@@H](O)[C@H](O)[C@H]4O)[C@H]1[C@H]23 |
| 76 | OC[C@H]1O[C@@H](O[C@@H]2OC=C([C@H]3[C@H](O)C=C(CO)[C@@H]23)C(=O)[O-])[C@H](O)[C@@H](O)[C@@H]1O |
| 77 | OCC1=C[C@@H](O)[C@H]2[C@@H]1[C@H](O)OC=C2C(=O)[O-] |
| 78 | CCCCCC\C=C/CCCCCCCC(=O)[O-] |
| 79 | c1ccc2[nH]ccc2c1 |
| 80 | CC1=CC(=O)CC(C)(C)C1 |
| 81 | CC(C)CCC[C@H](C)CCC[C@H](C)CCCC(=O)C |
| 82 | CCCCCC(=O)[O-] |
| 83 | C[C@@H](CCC=C(C)C)[C@H]1CCC(=C)C=C1 |
| 84 | CC(=CCCC(=O)C)C |
| 85 | CCCCCCCCCCCCCCC=C |
| 86 | Cc1cc(c(O)c(c1)C(C)(C)C)C(C)(C)C |
| 87 | CC(C)CCCCCOC(=O)c1ccccc1C(=O)OCCCCCC(C)C |
| 88 | CCCCCCCC\C=C/CCCCCCCC(=O)OCC |
| 89 | CC1(C)[C@H]2CC[C@]1(C)[C@H](O)C2 |
| 90 | CCCCCCCCC(=O)[O-] |
| 91 | CC(C)[C@H]1CC=C(C)[C@@H]2CCC(=C[C@H]12)C |
| 92 | c1ccc2c(c1)ccc3ccccc23 |
| 93 | Cc1oc2ccccc2c1 |
| 94 | Oc1cc2OC(=O)C=Cc2cc1O |
| 95 | Oc1cc2C=CC(=O)Oc2cc1[O-] |
| 96 | Cc1ccc2C(=O)c3c(O)c(O)ccc3C(=O)c2c1O |
| 97 | Cc1ccc2C(=O)c3c([O-])c(O)ccc3C(=O)c2c1O |
| 98 | Cc1ccc2C(=O)c3c([O-])c(O)ccc3C(=O)c2c1[O-] |
| 99 | Cc1ccc2C(=O)c3ccccc3C(=O)c2c1 |
| 100 | CCCCCCCCCCCCCC(=O)C |
| 101 | CCC(CC)(C(=O)C)C(=C(C)C)C |
| 102 | CCCCCCCCCCCCCCCCCC(=O)C |
| 103 | CO |
| 104 | CCCCCCC[C@H]1CCC(=O)O1 |
| 105 | CCCCCCCCCCCCCCCC=O |
| 106 | CCCCCCCCCCCCCCCCC(=O)OC |
| 107 | CC[C@@H](C)CCC[C@@H](C)CCC[C@H](C)CCCC(C)C |
| 108 | COc1cc2C=CC(=O)Oc2c(OC)c1[O-] |
| 109 | N(c1ccccc1)c2cccc3ccccc23 |
| 110 | COc1ccc2C(=O)c3ccccc3C(=O)c2c1O |
| 111 | COc1ccc2C(=O)c3ccccc3C(=O)c2c1[O-] |
| 112 | OCc1ccc2C(=O)c3ccccc3C(=O)c2c1O |
| 113 | OCc1ccc2C(=O)c3ccccc3C(=O)c2c1[O-] |
| 114 | Cc1c(O)cc2C(=O)c3ccccc3C(=O)c2c1O |
| 115 | Cc1c(O)cc2C(=O)c3ccccc3C(=O)c2c1[O-] |
| 116 | CC1(C)O[C@@]2(C)CC[C@@H]1C[C@@H]2O |
| 117 | CC(=O)OCC1=C[C@@H]2OC(=O)C3=CO[C@@H](O[C@@H]4O[C@H](CO)[C@@H](O)[C@H](O)[C@H]4O)[C@@H]1[C@H]23 |
| 118 | CC(=O)OCC1=C[C@@H]2OC(=O)C3=CO[C@@H](O)[C@@H]1[C@H]23 |
| 119 | CCCCC[C@H]1CCC(=O)O1 |
| 120 | CC[C@@H]1[C@@H](C)CCC[C@]1([NH3+])CC |
| 121 | Cc1cc2C(=O)c3ccccc3C(=O)c2c(O)c1O |
| 122 | Cc1cc2C(=O)c3ccccc3C(=O)c2c([O-])c1O |
| 123 | COc1c(O)cc2C(=O)c3ccccc3C(=O)c2c1[O-] |
| 124 | COc1cc(O)c2C(=O)c3ccc(C)cc3C(=O)c2c1O |
| 125 | COc1cc(O)c2C(=O)c3ccc(C)cc3C(=O)c2c1[O-] |
| 126 | COCc1ccc2C(=O)c3c(OC)c(O)ccc3C(=O)c2c1OC |
| 127 | COCc1ccc2C(=O)c3c(O)c(OC)c(O)cc3C(=O)c2c1O |
| 128 | COCc1ccc2C(=O)c3c([O-])c(OC)c(O)cc3C(=O)c2c1O |
| 129 | COCc1ccc2C(=O)c3c([O-])c(OC)c(O)cc3C(=O)c2c1[O-] |
| 130 | CC[C@H]1C[C@@H](C)[C@@H](C)[C@@]([NH3+])(CC)C1 |
| 131 | COCc1ccc2C(=O)c3c(OC)c(O)ccc3C(=O)c2c1O |
| 132 | COCc1ccc2C(=O)c3c(OC)c(O)ccc3C(=O)c2c1[O-] |
| 133 | Cc1cc2C(=O)c3ccccc3C(=O)c2c(O)c1C |
| 134 | Cc1cc2C(=O)c3ccccc3C(=O)c2c([O-])c1C |
| 135 | OCc1cc(O)c2C(=O)c3ccccc3C(=O)c2c1 |
| 136 | OCc1cc([O-])c2C(=O)c3ccccc3C(=O)c2c1 |
| 137 | COc1cc(O)c2C(=O)c3ccccc3C(=O)c2c1 |
| 138 | COc1cc([O-])c2C(=O)c3ccccc3C(=O)c2c1 |
| 139 | OCc1ccc2C(=O)c3c(O)cccc3C(=O)c2c1 |
| 140 | OCc1ccc2C(=O)c3c([O-])cccc3C(=O)c2c1 |
| 141 | OCc1ccc2C(=O)c3cccc(O)c3C(=O)c2c1 |
| 142 | OCc1ccc2C(=O)c3cccc([O-])c3C(=O)c2c1 |
| 143 | CC(C)C[C@@]1([NH3+])C[C@@H](C)CC[C@H]1C |
| 144 | CCC[C@@H]1CCCCC[C@H](C)C1 |
| 145 | CCCCCCCCCC[C@@H](C)c1ccccc1 |
| 146 | OC[C@H]1O[C@H](O[C@]2(CO[C@]3(CO[C@]4(CO[C@]5(CO)O[C@H](CO)[C@@H](O)[C@@H]5O)O[C@H](CO)[C@@H](O)[C@@H]4O)O[C@H](CO)[C@@H](O)[C@@H]3O)O[C@H](CO)[C@@H](O)[C@@H]2O)[C@H](O)[C@@H](O)[C@@H]1O |
| 147 | CC(=C)CCC(C)(C)C |
| 148 | COC(=O)c1ccc2C(=O)c3ccccc3C(=O)c2c1 |
| 149 | COCc1ccc2C(=O)c3ccc(O)c(OC)c3C(=O)c2c1OC |
| 150 | COc1c(O)ccc2C(=O)c3ccccc3C(=O)c12 |
| 151 | Oc1ccc2C(=O)c3ccccc3C(=O)c2c1 |
| 152 | COc1c(O)cc2C(=O)c3cc(O)ccc3C(=O)c2c1[O-] |
| 153 | C\C=C\CCCCc1ccc(C)cc1C |
| 154 | COc1cc(O)c2C(=O)[C@@H](O)[C@H](Oc2c1)c3ccc(OC)c(O)c3 |
| 155 | CCC[C@@H](C)CCCC[C@@H](C)CC |
| 156 | COc1c(O)cc2C(=O)c3ccccc3C(=O)c2c1OC |
| 157 | CCCCCCCCCCC[C@@H](O)[C@H](C)CCCCCCCCCCCCCCCCCC(=O)[O-] |
| 158 | Oc1cccc(c1)C#C |
| 159 | CC[C@@H](CC[C@@H](C)[C@H]1CC[C@H]2[C@@H]3CC=C4C[C@@H](O)CC[C@]4(C)[C@@H]3CC[C@]12C)C(C)C |
| 160 | CC(C)CC[C@@H](CC[C@@H](C)[C@H]1CC[C@H]2[C@@H]3CC=C4C[C@@H](O)CC[C@]4(C)[C@@H]3CC[C@]12C)C(C)C |
| 161 | C[C@@H]1CC[C@]2(C[C@@H]3C[C@H]4[C@@H]5CC=C6C[C@@H](O)CC[C@]6(C)[C@@H]5CC[C@]4(C)[C@@H]3[C@@H]2C)OC1 |
| 162 | COc1cc(OC)c2C(=O)c3ccccc3C(=O)c2c1CO |
| 163 | C[C@H](O)[C@H]1CCC(=O)O1 |
| 164 | OC[C@H]1O[C@@H](O[C@@H]2OC=C[C@@H]3[C@@H](O)C=C(O)[C@@H]23)[C@H](O)[C@@H](O)[C@@H]1O |
| 165 | O[C@@H]1OC=C[C@@H]2[C@H](O)C=C(O)[C@@H]12 |
| 166 | COC(=O)C1=CO[C@@H](O[C@@H]2O[C@H](CO)[C@@H](O)[C@H](O)[C@H]2O)[C@H]3[C@@H]1[C@@H]4O[C@@H]4[C@@]3(O)CO |
| 167 | COC(=O)C1=CO[C@@H](O)[C@H]2[C@@H]1[C@@H]3O[C@@H]3[C@@]2(O)CO |
| 168 | CCCCCCCCCCCCCCCCCC(C)C |
| 169 | OC[C@H]1O[C@H](O[C@]2(CO[C@]3(CO[C@]4(CO)O[C@H](CO)[C@@H](O)[C@@H]4O)O[C@H](CO)[C@@H](O)[C@@H]3O)O[C@H](CO)[C@@H](O)[C@@H]2O)[C@H](O)[C@@H](O)[C@@H]1O |
| 170 | CCOc1c(O)ccc2C(=O)c3ccccc3C(=O)c12 |
| 171 | OC[C@H]1Oc2cc(\C=C\C=O)ccc2O[C@H]1c3ccc(O)c(O)c3 |
| 172 | OC\C=C\c1ccc2O[C@@H]([C@@H](CO)Oc2c1)c3ccc(O)c(O)c3 |
| 173 | O=Cc1ccc2C(=O)c3ccccc3C(=O)c2c1 |
| 174 | OC[C@H]1O[C@@H](O[C@@H]2OC=C([C@@H]3[C@@H](O)C=C(CO)[C@H]23)C(=O)[O-])[C@H](O)[C@@H](O)[C@@H]1O |
| 175 | CC(=O)OC[C@H]1O[C@@H](O[C@@H]2OC=C3[C@H]4[C@@H](OC3=O)C=C(COC(=O)C)[C@@H]24)[C@H](OC(=O)C)[C@@H](OC(=O)C)[C@@H]1OC(=O)C |
| 176 | Cc1cccc(CC(C)(C)CO)c1 |
| 177 | O[C@@H]1Cc2ccccc12 |
| 178 | CCCCCCCC\C=C/CCCCCC(=O)[O-] |
| 179 | COC(=O)C1=CO[C@@H](O[C@@H]2O[C@H](CO)[C@@H](O)[C@H](O)[C@H]2O)[C@H]3[C@H](CO[C@@H](O)[C@]4(O)[C@H]5O[C@H]5[C@H]6[C@@H]4[C@H](O[C@@H]7O[C@H](CO)[C@@H](O)[C@H](O)[C@H]7O)OC=C6C(=O)OC)[C@H]8O[C@H]8[C@H]13 |
| 180 | OC[C@H]1O[C@@H](O[C@@H]2OC=C3[C@@H]4[C@@H](OC3=O)C=C(CO)[C@@H]24)[C@H](O)[C@@H](O)[C@@H]1O |
| 181 | OC[C@H]1O[C@@H](O[C@H]2OC=C([C@@H]3[C@@H](O)C=C(CO)[C@H]23)C(=O)[O-])[C@H](O)[C@@H](O)[C@@H]1O |
| 182 | OCC1=C[C@H](O)[C@@H]2[C@H]1[C@@H](O)OC=C2C(=O)[O-] |
| 183 | CCOCC |
| 184 | OC[C@H]1O[C@@H](O)[C@H](O)[C@@H](O)[C@@H]1O[C@@H]2O[C@H](CO[C@H]3OC[C@@H](O)[C@H](O)[C@H]3O)[C@@H](O[C@@H]4O[C@H](CO[C@H]5OC[C@@H](O)[C@H](O)[C@H]5O)[C@@H](O[C@@H]6O[C@H](CO[C@H]7OC[C@@H](O)[C@H](O)[C@H]7O)[C@@H](O)[C@H](O)[C@H]6O)[C@H](O)[C@H]4O)[C@H](O)[C@H]2O |
| 185 | OC[C@H]1Oc2ccc(cc2O[C@@H]1c3ccc(O)c(O)c3)[C@H]4OC[C@H]5[C@@H]4CO[C@@H]5c6ccc(O)c(O)c6 |
| 186 | OC[C@H]1Oc2ccc(cc2O[C@@H]1c3ccc(O)c(O)c3)[C@H]4OC[C@H]5[C@@H]4CO[C@@H]5c6ccc(O)c([O-])c6 |
| 187 | OC[C@H]1Oc2ccc(cc2O[C@@H]1c3ccc([O-])c(O)c3)[C@H]4OC[C@H]5[C@@H]4CO[C@@H]5c6ccc(O)c([O-])c6 |
| 188 | Cc1cccc(C=O)c1 |
| 189 | OC[C@@]1(O)C=C[C@H]2[C@@H]1[C@H](O)OC=C2C(=O)[O-] |
| 190 | OC[C@H]1[C@H]2[C@@H](OC1=O)C=C(CO)[C@H]2CO |
| 191 | OCC1=CC[C@@H]2CCOC(=O)[C@H]12 |
| 192 | COC(=O)C1=CO[C@@H](O[C@@H]2O[C@H](CO)[C@@H](O)[C@H](O)[C@H]2O)[C@H]3[C@@H]1[C@@H]4O[C@@H]4[C@@]35OC(=O)C(=C5)C(=O)c6ccc(O)cc6 |
| 193 | COC(=O)C1=CO[C@@H](O[C@@H]2O[C@H](CO)[C@@H](O)[C@H](O)[C@H]2O)[C@H]3[C@@H]1[C@@H]4O[C@@H]4[C@@]35OC(=O)C(=C5)C(=O)c6ccc([O-])cc6 |
| 194 | OCCOC(=O)c1cc(O)c(CO)cc1C(=O)c2ccccc2O |
| 195 | OCCOC(=O)c1cc([O-])c(CO)cc1C(=O)c2ccccc2O |
| 196 | COc1c(O)c(ccc1[O-])C(=O)c2c([O-])c(C)c(O)cc2C(=O)OCCO |
| 197 | COc1c([O-])ccc(C(=O)c2c([O-])c(C)c([O-])cc2C(=O)OCCO)c1O |
| 198 | COC(=O)C1=CO[C@@H](O[C@@H]2O[C@H](CO)[C@@H](O)[C@H](O)[C@H]2O)[C@@H]3[C@H](CO)[C@H](O)C[C@H]13 |
| 199 | CCCCO[C@]12OC[C@@H](O)[C@H](O)[C@@]1(CO)[C@@H]2O |
| 200 | Oc1cc(O)c2c3ccc([O-])cc3[C@@H]4Oc5ccccc5[C@H]6CC(=O)c1c2[C@@H]46 |
| 201 | Oc1cc([O-])c2c3ccc([O-])cc3[C@@H]4Oc5ccccc5[C@H]6CC(=O)c1c2[C@@H]46 |
| 202 | CC(C)(CO)CO |
| 203 | C[C@@H]1CC[C@@]2(CC[C@]3(C)C(=CC[C@@H]4[C@@]5(C)CC[C@H](O)[C@](C)(CO)[C@H]5CC[C@@]34C)[C@@H]2[C@]1(C)O)C(=O)O |
| 204 | C[C@@H]1CC[C@@]2(CC[C@]3(C)C(=CC[C@@H]4[C@@]5(C)CC[C@H](O)[C@](C)(CO)[C@H]5CC[C@@]34C)[C@@H]2[C@]1(C)O)C(=O)[O-] |
| 205 | COc1c(C)c(O)cc2C(=O)c3ccccc3C(=O)c12 |
| 206 | CCCCCCCCCCCCCCCCCCCC(=O)[O-] |
| 207 | CC(=C)[C@@H]1CCC(=CC1)C |
| 208 | OC[C@H]1O[C@@H](S\C(=N/OS(=O)(=O)[O-])\CCc2ccccc2)[C@H](O)[C@@H](O)[C@@H]1O |
| 209 | C[N+](C)(C)CCOC(=O)c1ccc(O)cc1 |
| 210 | C[N+](C)(C)CCOC(=O)c1ccc([O-])cc1 |
| 211 | Oc1c(O)c(O)c(C(=O)\C=C\c2ccccc2)c(O)c1O |
| 212 | COc1ccc(cc1)C2=C([O-])C(=O)c3c(O)c(O)c(O)c(O)c3O2 |
| 213 | Oc1c(O)c(O)c2C(=O)C(=C(Oc2c1O)c3ccccc3)[O-] |
| 214 | OC[C@H]1O[C@@H](S\C(=N\OS(=O)(=O)[O-])\Cc2ccc(O)cc2)[C@H](O)[C@@H](O)[C@@H]1O |
| 215 | COc1cc(\C=C\C(=O)OCC[N+](C)(C)C)cc(OC)c1O |
| 216 | C[C@]12C[C@@H](O)[C@@H]3C[C@@]1(O[C@@H]4O[C@H](CO)[C@@H](O)[C@@H](O)[C@H]4O)[C@]3(COC(=O)c5ccccc5)C(=O)O2 |
| 217 | C[C@]1(O[C@@H]2O[C@H](CO)[C@@H](O)[C@H](O)[C@H]2O)O[C@@H]3CC(=O)[C@@H]4C[C@@]1(O)[C@]34COC(=O)c5ccccc5 |
| 218 | [O-]C(=O)c1ccccc1 |
| 219 | Oc1cc(cc(O)c1O)C(=O)O[C@@H]2O[C@@H]3COC(=O)c4cc(O)c(O)c(O)c4c5c(O)c(O)c(O)cc5C(=O)O[C@H]3[C@@H]6OC(=O)c7cc(O)c(O)c(O)c7c8c(O)c(O)c(O)cc8C(=O)O[C@@H]26 |
| 220 | Oc1cc2C(=O)OC[C@H]3O[C@@H](OC(=O)c4cc(O)c([O-])c(O)c4)[C@@H]5OC(=O)c6cc(O)c(O)c(O)c6c7c(O)c(O)c(O)cc7C(=O)O[C@H]5[C@@H]3OC(=O)c8cc(O)c(O)c(O)c8c2c(O)c1O |
| 221 | O[C@H]1COC(=O)c2cc(O)c(O)c(O)c2c3c(O)c(O)c(O)cc3C(=O)O[C@H]1[C@H]4OC(=O)c5cc(O)c(O)c(O)c5c6c(O)c(O)c(O)c7[C@@H](O)[C@H]4OC(=O)c67 |
| 222 | O[C@H]1COC(=O)c2cc(O)c(O)c(O)c2c3c(O)c(O)c(O)cc3C(=O)O[C@H]1[C@H]4OC(=O)c5cc(O)c(O)c(O)c5c6c(O)c(O)c([O-])c7[C@@H](O)[C@@H]4OC(=O)c67 |
| 223 | O[C@H]1COC(=O)c2cc(O)c(O)c(O)c2c3c(O)c(O)c(O)cc3C(=O)O[C@H]1[C@H]4OC(=O)c5cc(O)c(O)c(O)c5c6c(O)c(O)c(O)c7[C@H](O)[C@@H]4OC(=O)c67 |
| 224 | O[C@H]1COC(=O)c2cc(O)c(O)c(O)c2c3c(O)c(O)c(O)cc3C(=O)O[C@H]1[C@H]4OC(=O)c5cc(O)c(O)c(O)c5c6c(O)c(O)c([O-])c7[C@H](O)[C@H]4OC(=O)c67 |
| 225 | Oc1cc(cc(O)c1O)C(=O)O[C@@H]2O[C@@H]3COC(=O)c4cc(O)c(O)c(O)c4c5c(O)c(O)c(O)cc5C(=O)O[C@H]3[C@H](OC(=O)c6cc(O)c(O)c(O)c6)[C@H]2OC(=O)c7cc(O)c(O)c(O)c7 |
| 226 | Oc1cc(cc(O)c1O)C(=O)O[C@H]2[C@H](OC(=O)c3cc(O)c([O-])c(O)c3)O[C@@H]4COC(=O)c5cc(O)c(O)c(O)c5c6c(O)c(O)c(O)cc6C(=O)O[C@H]4[C@@H]2OC(=O)c7cc(O)c(O)c(O)c7 |
| 227 | Oc1cc(cc(O)c1O)C(=O)O[C@@H]2O[C@@H]3COC(=O)c4cc(O)c(O)c(O)c4c5c(O)c(O)c(O)cc5C(=O)O[C@H]3[C@H](OC(=O)c6cc(O)c(O)c(O)c6)[C@H]2OC(=O)c7cc(O)c([O-])c(O)c7 |
| 228 | Oc1cc(cc(O)c1O)C(=O)O[C@@H]2[C@@H](OC(=O)c3cc(O)c([O-])c(O)c3)[C@H](OC(=O)c4cc(O)c([O-])c(O)c4)O[C@@H]5COC(=O)c6cc(O)c(O)c(O)c6c7c(O)c(O)c(O)cc7C(=O)O[C@@H]25 |
| 229 | Oc1cc(cc(O)c1O)C(=O)O[C@@H]2O[C@@H]3COC(=O)c4cc(O)c(O)c(O)c4c5c(O)c(O)c(O)cc5C(=O)O[C@H]3[C@H](OC(=O)c6cc(O)c([O-])c(O)c6)[C@H]2OC(=O)c7cc(O)c(O)c(O)c7 |
| 230 | Oc1cc(cc(O)c1O)C(=O)O[C@H]2[C@H](OC(=O)c3cc(O)c([O-])c(O)c3)O[C@@H]4COC(=O)c5cc(O)c(O)c(O)c5c6c(O)c(O)c(O)cc6C(=O)O[C@H]4[C@@H]2OC(=O)c7cc(O)c([O-])c(O)c7 |
| 231 | Oc1cc(cc(O)c1O)C(=O)O[C@@H]2O[C@@H]3COC(=O)c4cc(O)c(O)c(O)c4c5c(O)c(O)c(O)cc5C(=O)O[C@H]3[C@H](OC(=O)c6cc(O)c([O-])c(O)c6)[C@H]2OC(=O)c7cc(O)c([O-])c(O)c7 |
| 232 | Oc1cc2C(=O)OC[C@H]3O[C@@H](OC(=O)c4cc(O)c([O-])c(O)c4)[C@H](OC(=O)c5cc(O)c([O-])c(O)c5)[C@@H](OC(=O)c6cc(O)c([O-])c(O)c6)[C@@H]3OC(=O)c7cc(O)c(O)c(O)c7c2c(O)c1O |
| 233 | Oc1cc(cc(O)c1O)C(=O)[O-] |
| 234 | Oc1cc(cc(O)c1[O-])C(=O)[O-] |
| 235 | OC[C@H]1O[C@@H](OC(=O)c2cc(O)c(O)c(O)c2)[C@H](O)[C@@H](O)[C@@H]1O |
| 236 | OC[C@H]1O[C@@H](OC(=O)c2cc(O)c([O-])c(O)c2)[C@H](O)[C@@H](O)[C@@H]1O |
| 237 | O[C@@H]1C[C@](O)(C[C@@H](OC(=O)c2cc(O)c(O)c(O)c2)[C@@H]1O)C(=O)[O-] |
| 238 | O[C@@H]1C[C@](O)(C[C@@H](OC(=O)c2cc(O)c([O-])c(O)c2)[C@@H]1O)C(=O)[O-] |
| 239 | O[C@@H]1C[C@](O)(C[C@@H](O)[C@@H]1OC(=O)c2cc(O)c(O)c(O)c2)C(=O)[O-] |
| 240 | O[C@@H]1C[C@](O)(C[C@@H](O)[C@@H]1OC(=O)c2cc(O)c([O-])c(O)c2)C(=O)[O-] |
| 241 | OC[C@H]1O[C@@H](O)[C@@H]2OC(=O)c3cc(O)c(O)c(O)c3c4c(O)c(O)c(O)cc4C(=O)O[C@H]2[C@@H]1O |
| 242 | CC(C)CCCCCCCCCCCC(=O)[O-] |
| 243 | COC(=O)c1cc(O)c(O)c([O-])c1[C@@H]2[C@@H]3OC(=O)c4c2c(O)c(O)c(O)c4c5c(O)c(O)c(O)cc5C(=O)O[C@H]3[C@H]6OC(=O)c7cc(O)c(O)c(O)c7c8c(O)c(O)c(O)cc8C(=O)OC[C@H]6O |
| 244 | COC(=O)c1cc(O)c(O)c([O-])c1[C@@H]2[C@H]3OC(=O)c4c2c([O-])c(O)c(O)c4c5c(O)c(O)c(O)cc5C(=O)O[C@H]3[C@H]6OC(=O)c7cc(O)c(O)c(O)c7c8c(O)c(O)c(O)cc8C(=O)OC[C@@H]6O |
| 245 | COC(=O)c1cc(O)c([O-])c([O-])c1[C@@H]2[C@H]3OC(=O)c4c2c([O-])c(O)c(O)c4c5c(O)c(O)c(O)cc5C(=O)O[C@H]3[C@H]6OC(=O)c7cc(O)c(O)c(O)c7c8c(O)c(O)c(O)cc8C(=O)OC[C@@H]6O |
| 246 | O[C@H]1COC(=O)c2cc(O)c(O)c(O)c2c3c(O)c(O)c(O)cc3C(=O)O[C@@H]1[C@@H]4OC(=O)c5cc(O)c(O)c(O)c5c6c(O)c(O)c(O)c7[C@@H]([C@H]4OC(=O)c67)c8c(O)c(O)c(O)cc8C(=O)O[C@@H]9[C@H](OC(=O)c%10cc(O)c(O)c(O)c%10)O[C@@H](COC(=O)c%11cc(O)c(O)c(O)c%11)[C@@H](OC(=O)c%12cc(O)c(O)c(O)c%12)[C@@H]9OC(=O)c%13cc(O)c(O)c(O)c%13 |
| 247 | O[C@H]1COC(=O)c2cc([O-])c(O)c(O)c2c3c(O)c(O)c(O)cc3C(=O)O[C@@H]1[C@@H]4OC(=O)c5cc(O)c(O)c(O)c5c6c(O)c(O)c(O)c7[C@@H]([C@H]4OC(=O)c67)c8c(O)c(O)c(O)cc8C(=O)O[C@@H]9[C@H](OC(=O)c%10cc(O)c(O)c(O)c%10)O[C@@H](COC(=O)c%11cc(O)c(O)c(O)c%11)[C@@H](OC(=O)c%12cc(O)c(O)c(O)c%12)[C@@H]9OC(=O)c%13cc(O)c(O)c(O)c%13 |
| 248 | O[C@H]1COC(=O)c2cc([O-])c(O)c(O)c2c3c(O)c(O)c(O)cc3C(=O)O[C@@H]1[C@@H]4OC(=O)c5cc([O-])c(O)c(O)c5c6c(O)c(O)c(O)c7[C@@H]([C@@H]4OC(=O)c67)c8c(O)c(O)c(O)cc8C(=O)O[C@@H]9[C@H](OC(=O)c%10cc(O)c(O)c(O)c%10)O[C@@H](COC(=O)c%11cc(O)c(O)c(O)c%11)[C@@H](OC(=O)c%12cc(O)c(O)c(O)c%12)[C@@H]9OC(=O)c%13cc(O)c(O)c(O)c%13 |
| 249 | O[C@@H]1COC(=O)c2cc([O-])c(O)c(O)c2c3c(O)c(O)c([O-])cc3C(=O)O[C@@H]1[C@@H]4OC(=O)c5cc(O)c(O)c(O)c5c6c(O)c(O)c(O)c7[C@@H]([C@H]4OC(=O)c67)c8c(O)c(O)c(O)cc8C(=O)O[C@@H]9[C@H](OC(=O)c%10cc(O)c(O)c(O)c%10)O[C@@H](COC(=O)c%11cc(O)c(O)c(O)c%11)[C@@H](OC(=O)c%12cc(O)c(O)c(O)c%12)[C@@H]9OC(=O)c%13cc(O)c(O)c(O)c%13 |
| 250 | O[C@@H]1COC(=O)c2cc([O-])c(O)c(O)c2c3c(O)c(O)c([O-])cc3C(=O)O[C@@H]1[C@@H]4OC(=O)c5cc([O-])c(O)c(O)c5c6c(O)c(O)c(O)c7[C@@H]([C@@H]4OC(=O)c67)c8c(O)c(O)c(O)cc8C(=O)O[C@@H]9[C@H](OC(=O)c%10cc(O)c(O)c(O)c%10)O[C@@H](COC(=O)c%11cc(O)c(O)c(O)c%11)[C@@H](OC(=O)c%12cc(O)c(O)c(O)c%12)[C@@H]9OC(=O)c%13cc(O)c(O)c(O)c%13 |
| 251 | O[C@@H]1COC(=O)c2cc([O-])c(O)c(O)c2c3c(O)c(O)c([O-])cc3C(=O)O[C@@H]1[C@@H]4OC(=O)c5cc([O-])c(O)c(O)c5c6c(O)c(O)c(O)c7[C@@H]([C@@H]4OC(=O)c67)c8c(O)c(O)c(O)cc8C(=O)O[C@@H]9[C@H](OC(=O)c%10cc(O)c(O)c(O)c%10)O[C@@H](COC(=O)c%11cc(O)c(O)c(O)c%11)[C@@H](OC(=O)c%12cc(O)c([O-])c(O)c%12)[C@@H]9OC(=O)c%13cc(O)c(O)c(O)c%13 |
| 252 | O[C@@H]1COC(=O)c2cc([O-])c(O)c(O)c2c3c(O)c(O)c([O-])cc3C(=O)O[C@@H]1[C@@H]4OC(=O)c5cc([O-])c(O)c(O)c5c6c(O)c(O)c(O)c7[C@@H]([C@@H]4OC(=O)c67)c8c(O)c(O)c(O)cc8C(=O)O[C@@H]9[C@H](OC(=O)c%10cc(O)c([O-])c(O)c%10)O[C@@H](COC(=O)c%11cc(O)c(O)c(O)c%11)[C@@H](OC(=O)c%12cc(O)c(O)c(O)c%12)[C@@H]9OC(=O)c%13cc(O)c(O)c(O)c%13 |
| 253 | O[C@H]1COC(=O)c2cc([O-])c(O)c(O)c2c3c(O)c(O)c([O-])cc3C(=O)O[C@@H]1[C@@H]4OC(=O)c5cc([O-])c(O)c(O)c5c6c(O)c(O)c(O)c7[C@@H]([C@@H]4OC(=O)c67)c8c(O)c(O)c(O)cc8C(=O)O[C@@H]9[C@H](OC(=O)c%10cc(O)c([O-])c(O)c%10)O[C@@H](COC(=O)c%11cc(O)c(O)c(O)c%11)[C@@H](OC(=O)c%12cc(O)c([O-])c(O)c%12)[C@@H]9OC(=O)c%13cc(O)c(O)c(O)c%13 |
| 254 | O[C@@H]1COC(=O)c2cc([O-])c(O)c(O)c2c3c(O)c(O)c([O-])cc3C(=O)O[C@@H]1[C@@H]4OC(=O)c5cc([O-])c(O)c(O)c5c6c(O)c(O)c(O)c7[C@@H]([C@@H]4OC(=O)c67)c8c(O)c(O)c(O)cc8C(=O)O[C@@H]9[C@H](OC(=O)c%10cc(O)c(O)c(O)c%10)O[C@@H](COC(=O)c%11cc(O)c(O)c(O)c%11)[C@@H](OC(=O)c%12cc(O)c(O)c(O)c%12)[C@@H]9OC(=O)c%13cc(O)c([O-])c(O)c%13 |
| 255 | O[C@H]1COC(=O)c2cc([O-])c(O)c(O)c2c3c(O)c(O)c([O-])cc3C(=O)O[C@@H]1[C@@H]4OC(=O)c5cc([O-])c(O)c(O)c5c6c(O)c(O)c(O)c7[C@@H]([C@@H]4OC(=O)c67)c8c(O)c(O)c(O)cc8C(=O)O[C@@H]9[C@H](OC(=O)c%10cc(O)c(O)c(O)c%10)O[C@@H](COC(=O)c%11cc(O)c(O)c(O)c%11)[C@@H](OC(=O)c%12cc(O)c([O-])c(O)c%12)[C@@H]9OC(=O)c%13cc(O)c([O-])c(O)c%13 |
| 256 | O[C@H]1COC(=O)c2cc([O-])c(O)c(O)c2c3c(O)c(O)c([O-])cc3C(=O)O[C@@H]1[C@@H]4OC(=O)c5cc([O-])c(O)c(O)c5c6c(O)c(O)c(O)c7[C@@H]([C@@H]4OC(=O)c67)c8c(O)c(O)c(O)cc8C(=O)O[C@@H]9[C@H](OC(=O)c%10cc(O)c([O-])c(O)c%10)O[C@@H](COC(=O)c%11cc(O)c(O)c(O)c%11)[C@@H](OC(=O)c%12cc(O)c(O)c(O)c%12)[C@@H]9OC(=O)c%13cc(O)c([O-])c(O)c%13 |
| 257 | O[C@@H]1COC(=O)c2cc([O-])c(O)c(O)c2c3c(O)c(O)c([O-])cc3C(=O)O[C@@H]1[C@@H]4OC(=O)c5cc([O-])c(O)c(O)c5c6c(O)c(O)c(O)c7[C@@H]([C@@H]4OC(=O)c67)c8c(O)c(O)c(O)cc8C(=O)O[C@@H]9[C@H](OC(=O)c%10cc(O)c([O-])c(O)c%10)O[C@@H](COC(=O)c%11cc(O)c(O)c(O)c%11)[C@@H](OC(=O)c%12cc(O)c([O-])c(O)c%12)[C@@H]9OC(=O)c%13cc(O)c([O-])c(O)c%13 |
| 258 | O[C@@H]1COC(=O)c2cc([O-])c(O)c(O)c2c3c(O)c(O)c([O-])cc3C(=O)O[C@@H]1[C@@H]4OC(=O)c5cc([O-])c(O)c(O)c5c6c(O)c(O)c(O)c7[C@@H]([C@@H]4OC(=O)c67)c8c(O)c(O)c(O)cc8C(=O)O[C@@H]9[C@H](OC(=O)c%10cc(O)c([O-])c(O)c%10)O[C@@H](COC(=O)c%11cc(O)c([O-])c(O)c%11)[C@@H](OC(=O)c%12cc(O)c([O-])c(O)c%12)[C@@H]9OC(=O)c%13cc(O)c([O-])c(O)c%13 |
| 259 | O[C@H]1COC(=O)c2cc([O-])c(O)c(O)c2c3c(O)c(O)c([O-])cc3C(=O)O[C@@H]1[C@@H]4OC(=O)c5cc([O-])c(O)c(O)c5c6c(O)c(O)c([O-])c7[C@@H]([C@@H]4OC(=O)c67)c8c(O)c(O)c(O)cc8C(=O)O[C@@H]9[C@H](OC(=O)c%10cc(O)c([O-])c(O)c%10)O[C@@H](COC(=O)c%11cc(O)c([O-])c(O)c%11)[C@@H](OC(=O)c%12cc(O)c([O-])c(O)c%12)[C@@H]9OC(=O)c%13cc(O)c([O-])c(O)c%13 |
| 260 | O[C@@H]1COC(=O)c2cc(O)c(O)c(O)c2c3c(O)c(O)c(O)cc3C(=O)O[C@@H]1[C@@H]4OC(=O)c5cc(O)c(O)c(O)c5c6c(O)c(O)c(O)c7[C@@H]([C@@H]4OC(=O)c67)c8c(O)c(O)c(O)cc8C(=O)O[C@H]9[C@H](OC(=O)c%10cc(O)c(O)c(O)c%10)[C@H](COC(=O)c%11cc(O)c(O)c(O)c%11)O[C@@H](OC(=O)c%12cc(O)c(O)c(O)c%12)[C@H]9OC(=O)c%13cc(O)c(O)c(O)c%13 |
| 261 | O[C@H]1COC(=O)c2cc([O-])c(O)c(O)c2c3c(O)c(O)c(O)cc3C(=O)O[C@@H]1[C@@H]4OC(=O)c5cc(O)c(O)c(O)c5c6c(O)c(O)c(O)c7[C@@H]([C@@H]4OC(=O)c67)c8c(O)c(O)c(O)cc8C(=O)O[C@H]9[C@H](OC(=O)c%10cc(O)c(O)c(O)c%10)[C@H](COC(=O)c%11cc(O)c(O)c(O)c%11)O[C@@H](OC(=O)c%12cc(O)c(O)c(O)c%12)[C@H]9OC(=O)c%13cc(O)c(O)c(O)c%13 |
| 262 | O[C@@H]1COC(=O)c2cc([O-])c(O)c(O)c2c3c(O)c(O)c([O-])cc3C(=O)O[C@@H]1[C@@H]4OC(=O)c5cc(O)c(O)c(O)c5c6c(O)c(O)c(O)c7[C@@H]([C@@H]4OC(=O)c67)c8c(O)c(O)c(O)cc8C(=O)O[C@H]9[C@H](OC(=O)c%10cc(O)c(O)c(O)c%10)[C@H](COC(=O)c%11cc(O)c(O)c(O)c%11)O[C@@H](OC(=O)c%12cc(O)c(O)c(O)c%12)[C@H]9OC(=O)c%13cc(O)c(O)c(O)c%13 |
| 263 | O[C@H]1COC(=O)c2cc([O-])c(O)c(O)c2c3c(O)c(O)c(O)cc3C(=O)O[C@@H]1[C@@H]4OC(=O)c5cc([O-])c(O)c(O)c5c6c(O)c(O)c(O)c7[C@@H]([C@@H]4OC(=O)c67)c8c(O)c(O)c(O)cc8C(=O)O[C@H]9[C@H](OC(=O)c%10cc(O)c(O)c(O)c%10)[C@H](COC(=O)c%11cc(O)c(O)c(O)c%11)O[C@@H](OC(=O)c%12cc(O)c(O)c(O)c%12)[C@H]9OC(=O)c%13cc(O)c(O)c(O)c%13 |
| 264 | O[C@H]1COC(=O)c2cc([O-])c(O)c(O)c2c3c(O)c(O)c([O-])cc3C(=O)O[C@@H]1[C@@H]4OC(=O)c5cc([O-])c(O)c(O)c5c6c(O)c(O)c(O)c7[C@@H]([C@@H]4OC(=O)c67)c8c(O)c(O)c(O)cc8C(=O)O[C@H]9[C@H](OC(=O)c%10cc(O)c(O)c(O)c%10)[C@H](COC(=O)c%11cc(O)c(O)c(O)c%11)O[C@@H](OC(=O)c%12cc(O)c(O)c(O)c%12)[C@H]9OC(=O)c%13cc(O)c(O)c(O)c%13 |
| 265 | O[C@H]1COC(=O)c2cc([O-])c(O)c(O)c2c3c(O)c(O)c([O-])cc3C(=O)O[C@@H]1[C@@H]4OC(=O)c5cc([O-])c(O)c(O)c5c6c(O)c(O)c(O)c7[C@@H]([C@@H]4OC(=O)c67)c8c(O)c(O)c(O)cc8C(=O)O[C@H]9[C@H](OC(=O)c%10cc(O)c(O)c(O)c%10)[C@H](COC(=O)c%11cc(O)c(O)c(O)c%11)O[C@@H](OC(=O)c%12cc(O)c([O-])c(O)c%12)[C@H]9OC(=O)c%13cc(O)c(O)c(O)c%13 |
| 266 | O[C@H]1COC(=O)c2cc([O-])c(O)c(O)c2c3c(O)c(O)c([O-])cc3C(=O)O[C@@H]1[C@@H]4OC(=O)c5cc([O-])c(O)c(O)c5c6c(O)c(O)c(O)c7[C@@H]([C@@H]4OC(=O)c67)c8c(O)c(O)c(O)cc8C(=O)O[C@H]9[C@H](OC(=O)c%10cc(O)c([O-])c(O)c%10)[C@H](COC(=O)c%11cc(O)c(O)c(O)c%11)O[C@@H](OC(=O)c%12cc(O)c(O)c(O)c%12)[C@H]9OC(=O)c%13cc(O)c(O)c(O)c%13 |
| 267 | O[C@H]1COC(=O)c2cc([O-])c(O)c(O)c2c3c(O)c(O)c([O-])cc3C(=O)O[C@@H]1[C@@H]4OC(=O)c5cc([O-])c(O)c(O)c5c6c(O)c(O)c(O)c7[C@@H]([C@@H]4OC(=O)c67)c8c(O)c(O)c(O)cc8C(=O)O[C@H]9[C@H](OC(=O)c%10cc(O)c([O-])c(O)c%10)[C@H](COC(=O)c%11cc(O)c(O)c(O)c%11)O[C@@H](OC(=O)c%12cc(O)c([O-])c(O)c%12)[C@H]9OC(=O)c%13cc(O)c(O)c(O)c%13 |
| 268 | O[C@H]1COC(=O)c2cc([O-])c(O)c(O)c2c3c(O)c(O)c([O-])cc3C(=O)O[C@@H]1[C@@H]4OC(=O)c5cc([O-])c(O)c(O)c5c6c(O)c(O)c(O)c7[C@@H]([C@@H]4OC(=O)c67)c8c(O)c(O)c(O)cc8C(=O)O[C@H]9[C@H](OC(=O)c%10cc(O)c(O)c(O)c%10)[C@H](COC(=O)c%11cc(O)c(O)c(O)c%11)O[C@@H](OC(=O)c%12cc(O)c(O)c(O)c%12)[C@H]9OC(=O)c%13cc(O)c([O-])c(O)c%13 |
| 269 | O[C@H]1COC(=O)c2cc([O-])c(O)c(O)c2c3c(O)c(O)c([O-])cc3C(=O)O[C@@H]1[C@@H]4OC(=O)c5cc([O-])c(O)c(O)c5c6c(O)c(O)c(O)c7[C@@H]([C@@H]4OC(=O)c67)c8c(O)c(O)c(O)cc8C(=O)O[C@H]9[C@H](OC(=O)c%10cc(O)c(O)c(O)c%10)[C@H](COC(=O)c%11cc(O)c(O)c(O)c%11)O[C@@H](OC(=O)c%12cc(O)c([O-])c(O)c%12)[C@H]9OC(=O)c%13cc(O)c([O-])c(O)c%13 |
| 270 | O[C@H]1COC(=O)c2cc([O-])c(O)c(O)c2c3c(O)c(O)c([O-])cc3C(=O)O[C@@H]1[C@@H]4OC(=O)c5cc([O-])c(O)c(O)c5c6c(O)c(O)c(O)c7[C@@H]([C@@H]4OC(=O)c67)c8c(O)c(O)c(O)cc8C(=O)O[C@H]9[C@H](OC(=O)c%10cc(O)c([O-])c(O)c%10)[C@H](COC(=O)c%11cc(O)c(O)c(O)c%11)O[C@@H](OC(=O)c%12cc(O)c(O)c(O)c%12)[C@H]9OC(=O)c%13cc(O)c([O-])c(O)c%13 |
| 271 | O[C@H]1COC(=O)c2cc([O-])c(O)c(O)c2c3c(O)c(O)c([O-])cc3C(=O)O[C@@H]1[C@@H]4OC(=O)c5cc([O-])c(O)c(O)c5c6c(O)c(O)c(O)c7[C@@H]([C@@H]4OC(=O)c67)c8c(O)c(O)c(O)cc8C(=O)O[C@H]9[C@H](OC(=O)c%10cc(O)c([O-])c(O)c%10)[C@H](COC(=O)c%11cc(O)c(O)c(O)c%11)O[C@@H](OC(=O)c%12cc(O)c([O-])c(O)c%12)[C@H]9OC(=O)c%13cc(O)c([O-])c(O)c%13 |
| 272 | O[C@@H]1COC(=O)c2cc([O-])c(O)c(O)c2c3c(O)c(O)c([O-])cc3C(=O)O[C@@H]1[C@@H]4OC(=O)c5cc([O-])c(O)c(O)c5c6c(O)c(O)c(O)c7[C@@H]([C@@H]4OC(=O)c67)c8c(O)c(O)c(O)cc8C(=O)O[C@H]9[C@H](OC(=O)c%10cc(O)c([O-])c(O)c%10)[C@H](COC(=O)c%11cc(O)c([O-])c(O)c%11)O[C@@H](OC(=O)c%12cc(O)c([O-])c(O)c%12)[C@H]9OC(=O)c%13cc(O)c([O-])c(O)c%13 |
| 273 | O[C@H]1COC(=O)c2cc([O-])c(O)c(O)c2c3c(O)c(O)c([O-])cc3C(=O)O[C@@H]1[C@@H]4OC(=O)c5cc([O-])c(O)c(O)c5c6c(O)c(O)c([O-])c7[C@@H]([C@@H]4OC(=O)c67)c8c(O)c(O)c(O)cc8C(=O)O[C@H]9[C@H](OC(=O)c%10cc(O)c([O-])c(O)c%10)[C@H](COC(=O)c%11cc(O)c([O-])c(O)c%11)O[C@@H](OC(=O)c%12cc(O)c([O-])c(O)c%12)[C@H]9OC(=O)c%13cc(O)c([O-])c(O)c%13 |
| 274 | O[C@@H]1COC(=O)c2cc(O)c(O)c(O)c2c3c(O)c(O)c(O)cc3C(=O)O[C@@H]1[C@@H]4OC(=O)c5cc(O)c(O)c(O)c5c6c(O)c(O)c(O)c7[C@@H]([C@@H]4OC(=O)c67)c8c(O)c(O)c(O)cc8C(=O)O[C@@H]9[C@H](COC(=O)c%10cc(O)c(O)c(O)c%10)O[C@@H](OC(=O)c%11cc(O)c(O)c(O)c%11)[C@@H](OC(=O)c%12cc(O)c(O)c(O)c%12)[C@H]9OC(=O)c%13cc(O)c(O)c(O)c%13 |
| 275 | O[C@H]1COC(=O)c2cc([O-])c(O)c(O)c2c3c(O)c(O)c(O)cc3C(=O)O[C@@H]1[C@@H]4OC(=O)c5cc(O)c(O)c(O)c5c6c(O)c(O)c(O)c7[C@@H]([C@H]4OC(=O)c67)c8c(O)c(O)c(O)cc8C(=O)O[C@@H]9[C@H](COC(=O)c%10cc(O)c(O)c(O)c%10)O[C@@H](OC(=O)c%11cc(O)c(O)c(O)c%11)[C@@H](OC(=O)c%12cc(O)c(O)c(O)c%12)[C@H]9OC(=O)c%13cc(O)c(O)c(O)c%13 |
| 276 | O[C@H]1COC(=O)c2cc([O-])c(O)c(O)c2c3c(O)c(O)c([O-])cc3C(=O)O[C@@H]1[C@@H]4OC(=O)c5cc(O)c(O)c(O)c5c6c(O)c(O)c(O)c7[C@@H]([C@@H]4OC(=O)c67)c8c(O)c(O)c(O)cc8C(=O)O[C@@H]9[C@H](COC(=O)c%10cc(O)c(O)c(O)c%10)O[C@@H](OC(=O)c%11cc(O)c(O)c(O)c%11)[C@@H](OC(=O)c%12cc(O)c(O)c(O)c%12)[C@H]9OC(=O)c%13cc(O)c(O)c(O)c%13 |
| 277 | O[C@H]1COC(=O)c2cc([O-])c(O)c(O)c2c3c(O)c(O)c(O)cc3C(=O)O[C@@H]1[C@@H]4OC(=O)c5cc([O-])c(O)c(O)c5c6c(O)c(O)c(O)c7[C@@H]([C@@H]4OC(=O)c67)c8c(O)c(O)c(O)cc8C(=O)O[C@@H]9[C@H](COC(=O)c%10cc(O)c(O)c(O)c%10)O[C@@H](OC(=O)c%11cc(O)c(O)c(O)c%11)[C@@H](OC(=O)c%12cc(O)c(O)c(O)c%12)[C@H]9OC(=O)c%13cc(O)c(O)c(O)c%13 |
| 278 | O[C@@H]1COC(=O)c2cc([O-])c(O)c(O)c2c3c(O)c(O)c([O-])cc3C(=O)O[C@@H]1[C@@H]4OC(=O)c5cc([O-])c(O)c(O)c5c6c(O)c(O)c(O)c7[C@@H]([C@@H]4OC(=O)c67)c8c(O)c(O)c(O)cc8C(=O)O[C@@H]9[C@H](COC(=O)c%10cc(O)c(O)c(O)c%10)O[C@@H](OC(=O)c%11cc(O)c(O)c(O)c%11)[C@@H](OC(=O)c%12cc(O)c(O)c(O)c%12)[C@H]9OC(=O)c%13cc(O)c(O)c(O)c%13 |
| 279 | O[C@@H]1COC(=O)c2cc([O-])c(O)c(O)c2c3c(O)c(O)c([O-])cc3C(=O)O[C@@H]1[C@@H]4OC(=O)c5cc([O-])c(O)c(O)c5c6c(O)c(O)c(O)c7[C@@H]([C@@H]4OC(=O)c67)c8c(O)c(O)c(O)cc8C(=O)O[C@@H]9[C@H](COC(=O)c%10cc(O)c(O)c(O)c%10)O[C@@H](OC(=O)c%11cc(O)c(O)c(O)c%11)[C@@H](OC(=O)c%12cc(O)c([O-])c(O)c%12)[C@H]9OC(=O)c%13cc(O)c(O)c(O)c%13 |
| 280 | O[C@@H]1COC(=O)c2cc([O-])c(O)c(O)c2c3c(O)c(O)c([O-])cc3C(=O)O[C@@H]1[C@@H]4OC(=O)c5cc([O-])c(O)c(O)c5c6c(O)c(O)c(O)c7[C@@H]([C@@H]4OC(=O)c67)c8c(O)c(O)c(O)cc8C(=O)O[C@@H]9[C@H](COC(=O)c%10cc(O)c(O)c(O)c%10)O[C@@H](OC(=O)c%11cc(O)c([O-])c(O)c%11)[C@@H](OC(=O)c%12cc(O)c(O)c(O)c%12)[C@H]9OC(=O)c%13cc(O)c(O)c(O)c%13 |
| 281 | O[C@@H]1COC(=O)c2cc([O-])c(O)c(O)c2c3c(O)c(O)c([O-])cc3C(=O)O[C@@H]1[C@@H]4OC(=O)c5cc([O-])c(O)c(O)c5c6c(O)c(O)c(O)c7[C@@H]([C@@H]4OC(=O)c67)c8c(O)c(O)c(O)cc8C(=O)O[C@@H]9[C@H](COC(=O)c%10cc(O)c(O)c(O)c%10)O[C@@H](OC(=O)c%11cc(O)c([O-])c(O)c%11)[C@@H](OC(=O)c%12cc(O)c([O-])c(O)c%12)[C@H]9OC(=O)c%13cc(O)c(O)c(O)c%13 |
| 282 | O[C@@H]1COC(=O)c2cc([O-])c(O)c(O)c2c3c(O)c(O)c([O-])cc3C(=O)O[C@@H]1[C@@H]4OC(=O)c5cc([O-])c(O)c(O)c5c6c(O)c(O)c(O)c7[C@@H]([C@@H]4OC(=O)c67)c8c(O)c(O)c(O)cc8C(=O)O[C@@H]9[C@H](COC(=O)c%10cc(O)c(O)c(O)c%10)O[C@@H](OC(=O)c%11cc(O)c(O)c(O)c%11)[C@@H](OC(=O)c%12cc(O)c(O)c(O)c%12)[C@H]9OC(=O)c%13cc(O)c([O-])c(O)c%13 |
| 283 | O[C@@H]1COC(=O)c2cc([O-])c(O)c(O)c2c3c(O)c(O)c([O-])cc3C(=O)O[C@@H]1[C@@H]4OC(=O)c5cc([O-])c(O)c(O)c5c6c(O)c(O)c(O)c7[C@@H]([C@@H]4OC(=O)c67)c8c(O)c(O)c(O)cc8C(=O)O[C@@H]9[C@H](COC(=O)c%10cc(O)c(O)c(O)c%10)O[C@@H](OC(=O)c%11cc(O)c(O)c(O)c%11)[C@@H](OC(=O)c%12cc(O)c([O-])c(O)c%12)[C@H]9OC(=O)c%13cc(O)c([O-])c(O)c%13 |
| 284 | O[C@@H]1COC(=O)c2cc([O-])c(O)c(O)c2c3c(O)c(O)c([O-])cc3C(=O)O[C@@H]1[C@@H]4OC(=O)c5cc([O-])c(O)c(O)c5c6c(O)c(O)c(O)c7[C@@H]([C@@H]4OC(=O)c67)c8c(O)c(O)c(O)cc8C(=O)O[C@@H]9[C@H](COC(=O)c%10cc(O)c(O)c(O)c%10)O[C@@H](OC(=O)c%11cc(O)c([O-])c(O)c%11)[C@@H](OC(=O)c%12cc(O)c(O)c(O)c%12)[C@H]9OC(=O)c%13cc(O)c([O-])c(O)c%13 |
| 285 | O[C@H]1COC(=O)c2cc([O-])c(O)c(O)c2c3c(O)c(O)c([O-])cc3C(=O)O[C@@H]1[C@@H]4OC(=O)c5cc([O-])c(O)c(O)c5c6c(O)c(O)c(O)c7[C@@H]([C@@H]4OC(=O)c67)c8c(O)c(O)c(O)cc8C(=O)O[C@@H]9[C@H](COC(=O)c%10cc(O)c(O)c(O)c%10)O[C@@H](OC(=O)c%11cc(O)c([O-])c(O)c%11)[C@@H](OC(=O)c%12cc(O)c([O-])c(O)c%12)[C@H]9OC(=O)c%13cc(O)c([O-])c(O)c%13 |
| 286 | O[C@@H]1COC(=O)c2cc([O-])c(O)c(O)c2c3c(O)c(O)c([O-])cc3C(=O)O[C@@H]1[C@@H]4OC(=O)c5cc([O-])c(O)c(O)c5c6c(O)c(O)c(O)c7[C@@H]([C@@H]4OC(=O)c67)c8c(O)c(O)c(O)cc8C(=O)O[C@@H]9[C@H](COC(=O)c%10cc(O)c([O-])c(O)c%10)O[C@@H](OC(=O)c%11cc(O)c([O-])c(O)c%11)[C@@H](OC(=O)c%12cc(O)c([O-])c(O)c%12)[C@H]9OC(=O)c%13cc(O)c([O-])c(O)c%13 |
| 287 | O[C@@H]1COC(=O)c2cc([O-])c(O)c(O)c2c3c(O)c(O)c([O-])cc3C(=O)O[C@@H]1[C@@H]4OC(=O)c5cc([O-])c(O)c(O)c5c6c(O)c(O)c([O-])c7[C@@H]([C@@H]4OC(=O)c67)c8c(O)c(O)c(O)cc8C(=O)O[C@@H]9[C@H](COC(=O)c%10cc(O)c([O-])c(O)c%10)O[C@@H](OC(=O)c%11cc(O)c([O-])c(O)c%11)[C@@H](OC(=O)c%12cc(O)c([O-])c(O)c%12)[C@H]9OC(=O)c%13cc(O)c([O-])c(O)c%13 |
| 288 | O[C@@H]1COC(=O)c2cc(O)c(O)c(O)c2c3c(O)c(O)c(O)cc3C(=O)O[C@@H]1[C@@H]4OC(=O)c5cc(O)c(O)c(O)c5c6c(O)c(O)c(O)c7[C@@H]([C@@H]4OC(=O)c67)c8c([O-])c(O)c(O)cc8C(=O)OC[C@@H]9O[C@@H](OC(=O)c%10cc(O)c(O)c(O)c%10)[C@@H](OC(=O)c%11cc(O)c(O)c(O)c%11)[C@@H](OC(=O)c%12cc(O)c(O)c(O)c%12)[C@@H]9OC(=O)c%13cc(O)c(O)c(O)c%13 |
| 289 | O[C@H]1COC(=O)c2cc([O-])c(O)c(O)c2c3c(O)c(O)c(O)cc3C(=O)O[C@@H]1[C@@H]4OC(=O)c5cc(O)c(O)c(O)c5c6c(O)c(O)c(O)c7[C@@H]([C@@H]4OC(=O)c67)c8c([O-])c(O)c(O)cc8C(=O)OC[C@@H]9O[C@@H](OC(=O)c%10cc(O)c(O)c(O)c%10)[C@@H](OC(=O)c%11cc(O)c(O)c(O)c%11)[C@@H](OC(=O)c%12cc(O)c(O)c(O)c%12)[C@@H]9OC(=O)c%13cc(O)c(O)c(O)c%13 |
| 290 | O[C@H]1COC(=O)c2cc([O-])c(O)c(O)c2c3c(O)c(O)c([O-])cc3C(=O)O[C@@H]1[C@@H]4OC(=O)c5cc(O)c(O)c(O)c5c6c(O)c(O)c(O)c7[C@@H]([C@@H]4OC(=O)c67)c8c([O-])c(O)c(O)cc8C(=O)OC[C@@H]9O[C@@H](OC(=O)c%10cc(O)c(O)c(O)c%10)[C@@H](OC(=O)c%11cc(O)c(O)c(O)c%11)[C@@H](OC(=O)c%12cc(O)c(O)c(O)c%12)[C@@H]9OC(=O)c%13cc(O)c(O)c(O)c%13 |
| 291 | O[C@H]1COC(=O)c2cc([O-])c(O)c(O)c2c3c(O)c(O)c(O)cc3C(=O)O[C@@H]1[C@@H]4OC(=O)c5cc([O-])c(O)c(O)c5c6c(O)c(O)c(O)c7[C@@H]([C@@H]4OC(=O)c67)c8c([O-])c(O)c(O)cc8C(=O)OC[C@@H]9O[C@@H](OC(=O)c%10cc(O)c(O)c(O)c%10)[C@@H](OC(=O)c%11cc(O)c(O)c(O)c%11)[C@@H](OC(=O)c%12cc(O)c(O)c(O)c%12)[C@@H]9OC(=O)c%13cc(O)c(O)c(O)c%13 |
| 292 | O[C@@H]1COC(=O)c2cc([O-])c(O)c(O)c2c3c(O)c(O)c([O-])cc3C(=O)O[C@@H]1[C@@H]4OC(=O)c5cc([O-])c(O)c(O)c5c6c(O)c(O)c(O)c7[C@@H]([C@@H]4OC(=O)c67)c8c([O-])c(O)c(O)cc8C(=O)OC[C@@H]9O[C@@H](OC(=O)c%10cc(O)c(O)c(O)c%10)[C@@H](OC(=O)c%11cc(O)c(O)c(O)c%11)[C@@H](OC(=O)c%12cc(O)c(O)c(O)c%12)[C@@H]9OC(=O)c%13cc(O)c(O)c(O)c%13 |
| 293 | O[C@H]1COC(=O)c2cc([O-])c(O)c(O)c2c3c(O)c(O)c([O-])cc3C(=O)O[C@@H]1[C@@H]4OC(=O)c5cc([O-])c(O)c(O)c5c6c(O)c(O)c(O)c7[C@@H]([C@@H]4OC(=O)c67)c8c([O-])c(O)c(O)cc8C(=O)OC[C@@H]9O[C@@H](OC(=O)c%10cc(O)c([O-])c(O)c%10)[C@@H](OC(=O)c%11cc(O)c(O)c(O)c%11)[C@@H](OC(=O)c%12cc(O)c(O)c(O)c%12)[C@@H]9OC(=O)c%13cc(O)c(O)c(O)c%13 |
| 294 | O[C@@H]1COC(=O)c2cc([O-])c(O)c(O)c2c3c(O)c(O)c([O-])cc3C(=O)O[C@@H]1[C@@H]4OC(=O)c5cc([O-])c(O)c(O)c5c6c(O)c(O)c(O)c7[C@@H]([C@@H]4OC(=O)c67)c8c([O-])c(O)c(O)cc8C(=O)OC[C@@H]9O[C@@H](OC(=O)c%10cc(O)c(O)c(O)c%10)[C@@H](OC(=O)c%11cc(O)c([O-])c(O)c%11)[C@@H](OC(=O)c%12cc(O)c(O)c(O)c%12)[C@@H]9OC(=O)c%13cc(O)c(O)c(O)c%13 |
| 295 | O[C@H]1COC(=O)c2cc([O-])c(O)c(O)c2c3c(O)c(O)c([O-])cc3C(=O)O[C@@H]1[C@@H]4OC(=O)c5cc([O-])c(O)c(O)c5c6c(O)c(O)c(O)c7[C@@H]([C@@H]4OC(=O)c67)c8c([O-])c(O)c(O)cc8C(=O)OC[C@@H]9O[C@@H](OC(=O)c%10cc(O)c([O-])c(O)c%10)[C@@H](OC(=O)c%11cc(O)c([O-])c(O)c%11)[C@@H](OC(=O)c%12cc(O)c(O)c(O)c%12)[C@@H]9OC(=O)c%13cc(O)c(O)c(O)c%13 |
| 296 | O[C@@H]1COC(=O)c2cc([O-])c(O)c(O)c2c3c(O)c(O)c([O-])cc3C(=O)O[C@@H]1[C@@H]4OC(=O)c5cc([O-])c(O)c(O)c5c6c(O)c(O)c(O)c7[C@@H]([C@@H]4OC(=O)c67)c8c([O-])c(O)c(O)cc8C(=O)OC[C@@H]9O[C@@H](OC(=O)c%10cc(O)c(O)c(O)c%10)[C@@H](OC(=O)c%11cc(O)c(O)c(O)c%11)[C@@H](OC(=O)c%12cc(O)c(O)c(O)c%12)[C@@H]9OC(=O)c%13cc(O)c([O-])c(O)c%13 |
| 297 | O[C@@H]1COC(=O)c2cc([O-])c(O)c(O)c2c3c(O)c(O)c([O-])cc3C(=O)O[C@@H]1[C@@H]4OC(=O)c5cc([O-])c(O)c(O)c5c6c(O)c(O)c(O)c7[C@@H]([C@@H]4OC(=O)c67)c8c([O-])c(O)c(O)cc8C(=O)OC[C@@H]9O[C@@H](OC(=O)c%10cc(O)c([O-])c(O)c%10)[C@@H](OC(=O)c%11cc(O)c(O)c(O)c%11)[C@@H](OC(=O)c%12cc(O)c(O)c(O)c%12)[C@@H]9OC(=O)c%13cc(O)c([O-])c(O)c%13 |
| 298 | O[C@H]1COC(=O)c2cc([O-])c(O)c(O)c2c3c(O)c(O)c([O-])cc3C(=O)O[C@@H]1[C@@H]4OC(=O)c5cc([O-])c(O)c(O)c5c6c(O)c(O)c(O)c7[C@@H]([C@@H]4OC(=O)c67)c8c([O-])c(O)c(O)cc8C(=O)OC[C@@H]9O[C@@H](OC(=O)c%10cc(O)c(O)c(O)c%10)[C@@H](OC(=O)c%11cc(O)c([O-])c(O)c%11)[C@@H](OC(=O)c%12cc(O)c(O)c(O)c%12)[C@@H]9OC(=O)c%13cc(O)c([O-])c(O)c%13 |
| 299 | O[C@H]1COC(=O)c2cc([O-])c(O)c(O)c2c3c(O)c(O)c([O-])cc3C(=O)O[C@@H]1[C@@H]4OC(=O)c5cc([O-])c(O)c(O)c5c6c(O)c(O)c(O)c7[C@@H]([C@@H]4OC(=O)c67)c8c([O-])c(O)c(O)cc8C(=O)OC[C@@H]9O[C@@H](OC(=O)c%10cc(O)c([O-])c(O)c%10)[C@@H](OC(=O)c%11cc(O)c([O-])c(O)c%11)[C@@H](OC(=O)c%12cc(O)c(O)c(O)c%12)[C@@H]9OC(=O)c%13cc(O)c([O-])c(O)c%13 |
| 300 | O[C@@H]1COC(=O)c2cc([O-])c(O)c(O)c2c3c(O)c(O)c([O-])cc3C(=O)O[C@@H]1[C@@H]4OC(=O)c5cc([O-])c(O)c(O)c5c6c(O)c(O)c(O)c7[C@@H]([C@@H]4OC(=O)c67)c8c([O-])c(O)c(O)cc8C(=O)OC[C@@H]9O[C@@H](OC(=O)c%10cc(O)c(O)c(O)c%10)[C@@H](OC(=O)c%11cc(O)c(O)c(O)c%11)[C@@H](OC(=O)c%12cc(O)c([O-])c(O)c%12)[C@@H]9OC(=O)c%13cc(O)c(O)c(O)c%13 |
| 301 | O[C@H]1COC(=O)c2cc([O-])c(O)c(O)c2c3c(O)c(O)c([O-])cc3C(=O)O[C@@H]1[C@@H]4OC(=O)c5cc([O-])c(O)c(O)c5c6c(O)c(O)c(O)c7[C@@H]([C@@H]4OC(=O)c67)c8c([O-])c(O)c(O)cc8C(=O)OC[C@@H]9O[C@@H](OC(=O)c%10cc(O)c([O-])c(O)c%10)[C@@H](OC(=O)c%11cc(O)c(O)c(O)c%11)[C@@H](OC(=O)c%12cc(O)c([O-])c(O)c%12)[C@@H]9OC(=O)c%13cc(O)c(O)c(O)c%13 |
| 302 | O[C@H]1COC(=O)c2cc([O-])c(O)c(O)c2c3c(O)c(O)c([O-])cc3C(=O)O[C@@H]1[C@@H]4OC(=O)c5cc([O-])c(O)c(O)c5c6c(O)c(O)c(O)c7[C@@H]([C@@H]4OC(=O)c67)c8c([O-])c(O)c(O)cc8C(=O)OC[C@@H]9O[C@@H](OC(=O)c%10cc(O)c(O)c(O)c%10)[C@@H](OC(=O)c%11cc(O)c([O-])c(O)c%11)[C@@H](OC(=O)c%12cc(O)c([O-])c(O)c%12)[C@@H]9OC(=O)c%13cc(O)c(O)c(O)c%13 |
| 303 | O[C@@H]1COC(=O)c2cc([O-])c(O)c(O)c2c3c(O)c(O)c([O-])cc3C(=O)O[C@@H]1[C@@H]4OC(=O)c5cc([O-])c(O)c(O)c5c6c(O)c(O)c(O)c7[C@@H]([C@@H]4OC(=O)c67)c8c([O-])c(O)c(O)cc8C(=O)OC[C@@H]9O[C@@H](OC(=O)c%10cc(O)c([O-])c(O)c%10)[C@@H](OC(=O)c%11cc(O)c([O-])c(O)c%11)[C@@H](OC(=O)c%12cc(O)c([O-])c(O)c%12)[C@@H]9OC(=O)c%13cc(O)c(O)c(O)c%13 |
| 304 | O[C@H]1COC(=O)c2cc([O-])c(O)c(O)c2c3c(O)c(O)c([O-])cc3C(=O)O[C@@H]1[C@@H]4OC(=O)c5cc([O-])c(O)c(O)c5c6c(O)c(O)c(O)c7[C@@H]([C@@H]4OC(=O)c67)c8c([O-])c(O)c(O)cc8C(=O)OC[C@@H]9O[C@@H](OC(=O)c%10cc(O)c(O)c(O)c%10)[C@@H](OC(=O)c%11cc(O)c(O)c(O)c%11)[C@@H](OC(=O)c%12cc(O)c([O-])c(O)c%12)[C@@H]9OC(=O)c%13cc(O)c([O-])c(O)c%13 |
| 305 | O[C@@H]1COC(=O)c2cc([O-])c(O)c(O)c2c3c(O)c(O)c([O-])cc3C(=O)O[C@@H]1[C@@H]4OC(=O)c5cc([O-])c(O)c(O)c5c6c(O)c(O)c(O)c7[C@@H]([C@@H]4OC(=O)c67)c8c([O-])c(O)c(O)cc8C(=O)OC[C@@H]9O[C@@H](OC(=O)c%10cc(O)c([O-])c(O)c%10)[C@@H](OC(=O)c%11cc(O)c(O)c(O)c%11)[C@@H](OC(=O)c%12cc(O)c([O-])c(O)c%12)[C@@H]9OC(=O)c%13cc(O)c([O-])c(O)c%13 |
| 306 | O[C@@H]1COC(=O)c2cc([O-])c(O)c(O)c2c3c(O)c(O)c([O-])cc3C(=O)O[C@@H]1[C@@H]4OC(=O)c5cc([O-])c(O)c(O)c5c6c(O)c(O)c(O)c7[C@@H]([C@@H]4OC(=O)c67)c8c([O-])c(O)c(O)cc8C(=O)OC[C@@H]9O[C@@H](OC(=O)c%10cc(O)c(O)c(O)c%10)[C@@H](OC(=O)c%11cc(O)c([O-])c(O)c%11)[C@@H](OC(=O)c%12cc(O)c([O-])c(O)c%12)[C@@H]9OC(=O)c%13cc(O)c([O-])c(O)c%13 |
| 307 | O[C@H]1COC(=O)c2cc([O-])c(O)c(O)c2c3c(O)c(O)c([O-])cc3C(=O)O[C@@H]1[C@@H]4OC(=O)c5cc([O-])c(O)c(O)c5c6c(O)c(O)c(O)c7[C@@H]([C@@H]4OC(=O)c67)c8c([O-])c(O)c(O)cc8C(=O)OC[C@@H]9O[C@@H](OC(=O)c%10cc(O)c([O-])c(O)c%10)[C@@H](OC(=O)c%11cc(O)c([O-])c(O)c%11)[C@@H](OC(=O)c%12cc(O)c([O-])c(O)c%12)[C@@H]9OC(=O)c%13cc(O)c([O-])c(O)c%13 |
| 308 | O[C@H]1COC(=O)c2cc([O-])c(O)c(O)c2c3c(O)c(O)c([O-])cc3C(=O)O[C@@H]1[C@@H]4OC(=O)c5cc([O-])c(O)c(O)c5c6c(O)c(O)c([O-])c7[C@@H]([C@@H]4OC(=O)c67)c8c([O-])c(O)c(O)cc8C(=O)OC[C@@H]9O[C@@H](OC(=O)c%10cc(O)c([O-])c(O)c%10)[C@@H](OC(=O)c%11cc(O)c([O-])c(O)c%11)[C@@H](OC(=O)c%12cc(O)c([O-])c(O)c%12)[C@@H]9OC(=O)c%13cc(O)c([O-])c(O)c%13 |
| 309 | C[C@]12CC(=O)[C@@H]3C[C@@]1(O)O[C@H](O2)[C@H]3COC(=O)c4ccccc4 |
| 310 | C[C@@H]1[C@H]2CC(=O)[C@@](C)(O)C[C@H]2OC1=O |
| 311 | C[C@]1(O)C[C@H]2OC(=O)C(=C)[C@H]2CC1=O |
| 312 | C[C@]1(O)C[C@H]2OC(=O)[C@H](COC(=O)c3ccccc3)[C@H]2CC1=O |
| 313 | COc1ccc(C(=O)C)c(O)c1 |
| 314 | CC1(C)[C@@H](O)CC[C@@]2(C)[C@H]1CC[C@]3(C)[C@@H]2C=CC4=C([O-])C(=O)C(=O)[C@@]34C |
| 315 | O[C@@H]1O[C@@H]2COC(=O)c3cc(O)c(O)c(O)c3c4c(O)c(O)c(O)cc4C(=O)O[C@H]2[C@@H]5OC(=O)c6cc(O)c(O)c(O)c6c7c(O)c(O)c(O)cc7C(=O)O[C@@H]15 |
| 316 | Oc1cc(cc(O)c1O)C(=O)OC[C@H]2O[C@@H](OC(=O)c3cc(O)c(O)c(O)c3)[C@H](OC(=O)c4cc(O)c(O)c(O)c4)[C@@H](OC(=O)c5cc(O)c(O)c(O)c5)[C@@H]2OC(=O)c6cc(O)c(O)c(O)c6 |
| 317 | Oc1cc(cc(O)c1O)C(=O)OC[C@H]2O[C@@H](OC(=O)c3cc(O)c(O)c(O)c3)[C@H](OC(=O)c4cc(O)c(O)c(O)c4)[C@@H](OC(=O)c5cc(O)c([O-])c(O)c5)[C@@H]2OC(=O)c6cc(O)c(O)c(O)c6 |
| 318 | Oc1cc(cc(O)c1O)C(=O)OC[C@H]2O[C@@H](OC(=O)c3cc(O)c(O)c(O)c3)[C@H](OC(=O)c4cc(O)c([O-])c(O)c4)[C@@H](OC(=O)c5cc(O)c(O)c(O)c5)[C@@H]2OC(=O)c6cc(O)c(O)c(O)c6 |
| 319 | Oc1cc(cc(O)c1O)C(=O)OC[C@H]2O[C@@H](OC(=O)c3cc(O)c(O)c(O)c3)[C@H](OC(=O)c4cc(O)c([O-])c(O)c4)[C@@H](OC(=O)c5cc(O)c([O-])c(O)c5)[C@@H]2OC(=O)c6cc(O)c(O)c(O)c6 |
| 320 | Oc1cc(cc(O)c1O)C(=O)OC[C@H]2O[C@@H](OC(=O)c3cc(O)c([O-])c(O)c3)[C@H](OC(=O)c4cc(O)c(O)c(O)c4)[C@@H](OC(=O)c5cc(O)c(O)c(O)c5)[C@@H]2OC(=O)c6cc(O)c(O)c(O)c6 |
| 321 | Oc1cc(cc(O)c1O)C(=O)OC[C@H]2O[C@@H](OC(=O)c3cc(O)c([O-])c(O)c3)[C@H](OC(=O)c4cc(O)c(O)c(O)c4)[C@@H](OC(=O)c5cc(O)c([O-])c(O)c5)[C@@H]2OC(=O)c6cc(O)c(O)c(O)c6 |
| 322 | Oc1cc(cc(O)c1O)C(=O)OC[C@H]2O[C@@H](OC(=O)c3cc(O)c([O-])c(O)c3)[C@H](OC(=O)c4cc(O)c([O-])c(O)c4)[C@@H](OC(=O)c5cc(O)c(O)c(O)c5)[C@@H]2OC(=O)c6cc(O)c(O)c(O)c6 |
| 323 | Oc1cc(cc(O)c1O)C(=O)OC[C@H]2O[C@@H](OC(=O)c3cc(O)c([O-])c(O)c3)[C@H](OC(=O)c4cc(O)c([O-])c(O)c4)[C@@H](OC(=O)c5cc(O)c([O-])c(O)c5)[C@@H]2OC(=O)c6cc(O)c(O)c(O)c6 |
| 324 | Oc1cc(cc(O)c1O)C(=O)OC[C@H]2O[C@@H](OC(=O)c3cc(O)c(O)c(O)c3)[C@H](OC(=O)c4cc(O)c(O)c(O)c4)[C@@H](OC(=O)c5cc(O)c(O)c(O)c5)[C@@H]2OC(=O)c6cc(O)c([O-])c(O)c6 |
| 325 | Oc1cc(cc(O)c1O)C(=O)OC[C@H]2O[C@@H](OC(=O)c3cc(O)c(O)c(O)c3)[C@H](OC(=O)c4cc(O)c(O)c(O)c4)[C@@H](OC(=O)c5cc(O)c([O-])c(O)c5)[C@@H]2OC(=O)c6cc(O)c([O-])c(O)c6 |
| 326 | Oc1cc(cc(O)c1O)C(=O)OC[C@H]2O[C@@H](OC(=O)c3cc(O)c(O)c(O)c3)[C@H](OC(=O)c4cc(O)c([O-])c(O)c4)[C@@H](OC(=O)c5cc(O)c(O)c(O)c5)[C@@H]2OC(=O)c6cc(O)c([O-])c(O)c6 |
| 327 | Oc1cc(cc(O)c1O)C(=O)OC[C@H]2O[C@@H](OC(=O)c3cc(O)c(O)c(O)c3)[C@H](OC(=O)c4cc(O)c([O-])c(O)c4)[C@@H](OC(=O)c5cc(O)c([O-])c(O)c5)[C@@H]2OC(=O)c6cc(O)c([O-])c(O)c6 |
| 328 | Oc1cc(cc(O)c1O)C(=O)OC[C@H]2O[C@@H](OC(=O)c3cc(O)c([O-])c(O)c3)[C@H](OC(=O)c4cc(O)c(O)c(O)c4)[C@@H](OC(=O)c5cc(O)c(O)c(O)c5)[C@@H]2OC(=O)c6cc(O)c([O-])c(O)c6 |
| 329 | Oc1cc(cc(O)c1O)C(=O)OC[C@H]2O[C@@H](OC(=O)c3cc(O)c([O-])c(O)c3)[C@H](OC(=O)c4cc(O)c(O)c(O)c4)[C@@H](OC(=O)c5cc(O)c([O-])c(O)c5)[C@@H]2OC(=O)c6cc(O)c([O-])c(O)c6 |
| 330 | Oc1cc(cc(O)c1O)C(=O)OC[C@H]2O[C@@H](OC(=O)c3cc(O)c([O-])c(O)c3)[C@H](OC(=O)c4cc(O)c([O-])c(O)c4)[C@@H](OC(=O)c5cc(O)c(O)c(O)c5)[C@@H]2OC(=O)c6cc(O)c([O-])c(O)c6 |
| 331 | Oc1cc(cc(O)c1O)C(=O)OC[C@H]2O[C@@H](OC(=O)c3cc(O)c([O-])c(O)c3)[C@H](OC(=O)c4cc(O)c([O-])c(O)c4)[C@@H](OC(=O)c5cc(O)c([O-])c(O)c5)[C@@H]2OC(=O)c6cc(O)c([O-])c(O)c6 |
| 332 | Oc1cc(cc(O)c1[O-])C(=O)OC[C@H]2O[C@@H](OC(=O)c3cc(O)c([O-])c(O)c3)[C@H](OC(=O)c4cc(O)c([O-])c(O)c4)[C@@H](OC(=O)c5cc(O)c([O-])c(O)c5)[C@@H]2OC(=O)c6cc(O)c([O-])c(O)c6 |
| 333 | COc1cc(ccc1O)c2[o+]c3cc(O)cc(O[C@@H]4O[C@H](CO)[C@@H](O)[C@H](O)[C@H]4O)c3cc2O[C@@H]5O[C@H](CO)[C@@H](O)[C@H](O)[C@H]5O |
| 334 | COc1cc(ccc1[O-])c2[o+]c3cc(O)cc(O[C@@H]4O[C@H](CO)[C@@H](O)[C@H](O)[C@H]4O)c3cc2O[C@@H]5O[C@H](CO)[C@@H](O)[C@H](O)[C@H]5O |
| 335 | Oc1ccccc1 |
| 336 | CC(=C[C@@H]1[C@@H](C(=O)O[C@H]2CC(=O)C(=C2C)C\C=C/C=C)C1(C)C)C |
| 337 | COC(=O)\C(=C\[C@@H]1[C@@H](C(=O)O[C@H]2CC(=O)C(=C2C)C\C=C/C=C)C1(C)C)\C |
| 338 | O[C@@H]1[C@@H](O)[C@@H]2OC(=O)c3cc(O)c(O)c(O)c3c4c(O)c(O)c(O)cc4C(=O)OC[C@H]2O[C@H]1OC(=O)c5cc(O)c(O)c(O)c5 |
| 339 | O[C@@H]1[C@@H](O)[C@@H]2OC(=O)c3cc(O)c(O)c(O)c3c4c(O)c(O)c(O)cc4C(=O)OC[C@H]2O[C@H]1OC(=O)c5cc(O)c([O-])c(O)c5 |
| 340 | O[C@@H]1O[C@@H]2COC(=O)c3cc(O)c(O)c(O)c3c4c(O)c(O)c(O)cc4C(=O)O[C@H]2[C@H](OC(=O)c5cc(O)c(O)c(O)c5)[C@H]1OC(=O)c6cc(O)c(O)c(O)c6 |
| 341 | O[C@@H]1O[C@@H]2COC(=O)c3cc(O)c(O)c(O)c3c4c(O)c(O)c(O)cc4C(=O)O[C@H]2[C@H](OC(=O)c5cc(O)c([O-])c(O)c5)[C@H]1OC(=O)c6cc(O)c(O)c(O)c6 |
| 342 | O[C@@H]1O[C@@H]2COC(=O)c3cc(O)c(O)c(O)c3c4c(O)c(O)c(O)cc4C(=O)O[C@H]2[C@H](OC(=O)c5cc(O)c(O)c(O)c5)[C@H]1OC(=O)c6cc(O)c([O-])c(O)c6 |
| 343 | O[C@@H]1O[C@@H]2COC(=O)c3cc(O)c(O)c(O)c3c4c(O)c(O)c(O)cc4C(=O)O[C@H]2[C@H](OC(=O)c5cc(O)c([O-])c(O)c5)[C@H]1OC(=O)c6cc(O)c([O-])c(O)c6 |
| 344 | O[C@@H]1[C@@H](COC(=O)c2cc(O)c(O)c(O)c2)O[C@@H](OC(=O)c3cc(O)c(O)c(O)c3)[C@H](OC(=O)c4cc(O)c(O)c(O)c4)[C@H]1OC(=O)c5cc(O)c(O)c(O)c5 |
| 345 | O[C@@H]1[C@@H](COC(=O)c2cc(O)c(O)c(O)c2)O[C@@H](OC(=O)c3cc(O)c(O)c(O)c3)[C@H](OC(=O)c4cc(O)c([O-])c(O)c4)[C@H]1OC(=O)c5cc(O)c(O)c(O)c5 |
| 346 | O[C@@H]1[C@@H](COC(=O)c2cc(O)c(O)c(O)c2)O[C@@H](OC(=O)c3cc(O)c(O)c(O)c3)[C@H](OC(=O)c4cc(O)c(O)c(O)c4)[C@H]1OC(=O)c5cc(O)c([O-])c(O)c5 |
| 347 | O[C@@H]1[C@@H](COC(=O)c2cc(O)c(O)c(O)c2)O[C@@H](OC(=O)c3cc(O)c(O)c(O)c3)[C@H](OC(=O)c4cc(O)c([O-])c(O)c4)[C@H]1OC(=O)c5cc(O)c([O-])c(O)c5 |
| 348 | O[C@@H]1[C@@H](COC(=O)c2cc(O)c(O)c(O)c2)O[C@@H](OC(=O)c3cc(O)c([O-])c(O)c3)[C@H](OC(=O)c4cc(O)c(O)c(O)c4)[C@H]1OC(=O)c5cc(O)c(O)c(O)c5 |
| 349 | O[C@@H]1[C@@H](COC(=O)c2cc(O)c(O)c(O)c2)O[C@@H](OC(=O)c3cc(O)c([O-])c(O)c3)[C@H](OC(=O)c4cc(O)c([O-])c(O)c4)[C@H]1OC(=O)c5cc(O)c(O)c(O)c5 |
| 350 | O[C@@H]1[C@@H](COC(=O)c2cc(O)c(O)c(O)c2)O[C@@H](OC(=O)c3cc(O)c([O-])c(O)c3)[C@H](OC(=O)c4cc(O)c(O)c(O)c4)[C@H]1OC(=O)c5cc(O)c([O-])c(O)c5 |
| 351 | O[C@@H]1[C@@H](COC(=O)c2cc(O)c(O)c(O)c2)O[C@@H](OC(=O)c3cc(O)c([O-])c(O)c3)[C@H](OC(=O)c4cc(O)c([O-])c(O)c4)[C@H]1OC(=O)c5cc(O)c([O-])c(O)c5 |
| 352 | O[C@@H]1[C@@H](COC(=O)c2cc(O)c([O-])c(O)c2)O[C@@H](OC(=O)c3cc(O)c([O-])c(O)c3)[C@H](OC(=O)c4cc(O)c([O-])c(O)c4)[C@H]1OC(=O)c5cc(O)c([O-])c(O)c5 |
| 353 | OC[C@H]1O[C@@H](OC(=O)c2cc(O)c(O)c(O)c2)[C@H](OC(=O)c3cc(O)c(O)c(O)c3)[C@@H](OC(=O)c4cc(O)c(O)c(O)c4)[C@@H]1O |
| 354 | OC[C@H]1O[C@@H](OC(=O)c2cc(O)c(O)c(O)c2)[C@H](OC(=O)c3cc(O)c(O)c(O)c3)[C@@H](OC(=O)c4cc(O)c([O-])c(O)c4)[C@@H]1O |
| 355 | OC[C@H]1O[C@@H](OC(=O)c2cc(O)c([O-])c(O)c2)[C@H](OC(=O)c3cc(O)c(O)c(O)c3)[C@@H](OC(=O)c4cc(O)c(O)c(O)c4)[C@@H]1O |
| 356 | OC[C@H]1O[C@@H](OC(=O)c2cc(O)c([O-])c(O)c2)[C@H](OC(=O)c3cc(O)c(O)c(O)c3)[C@@H](OC(=O)c4cc(O)c([O-])c(O)c4)[C@@H]1O |
| 357 | OC[C@H]1O[C@@H](OC(=O)c2cc(O)c(O)c(O)c2)[C@H](OC(=O)c3cc(O)c([O-])c(O)c3)[C@@H](OC(=O)c4cc(O)c(O)c(O)c4)[C@@H]1O |
| 358 | OC[C@H]1O[C@@H](OC(=O)c2cc(O)c(O)c(O)c2)[C@H](OC(=O)c3cc(O)c([O-])c(O)c3)[C@@H](OC(=O)c4cc(O)c([O-])c(O)c4)[C@@H]1O |
| 359 | OC[C@H]1O[C@@H](OC(=O)c2cc(O)c([O-])c(O)c2)[C@H](OC(=O)c3cc(O)c([O-])c(O)c3)[C@@H](OC(=O)c4cc(O)c(O)c(O)c4)[C@@H]1O |
| 360 | OC[C@H]1O[C@@H](OC(=O)c2cc(O)c([O-])c(O)c2)[C@H](OC(=O)c3cc(O)c([O-])c(O)c3)[C@@H](OC(=O)c4cc(O)c([O-])c(O)c4)[C@@H]1O |
| 361 | O[C@H]1[C@H](O)[C@@H](OC(=O)c2cc(O)c(O)c(O)c2)[C@H](OC(=O)c3cc(O)c(O)c(O)c3)O[C@@H]1COC(=O)c4cc(O)c(O)c(O)c4 |
| 362 | O[C@H]1[C@H](O)[C@@H](OC(=O)c2cc(O)c(O)c(O)c2)[C@H](OC(=O)c3cc(O)c([O-])c(O)c3)O[C@@H]1COC(=O)c4cc(O)c(O)c(O)c4 |
| 363 | O[C@H]1[C@H](O)[C@@H](OC(=O)c2cc(O)c([O-])c(O)c2)[C@H](OC(=O)c3cc(O)c(O)c(O)c3)O[C@@H]1COC(=O)c4cc(O)c(O)c(O)c4 |
| 364 | O[C@H]1[C@H](O)[C@@H](OC(=O)c2cc(O)c([O-])c(O)c2)[C@H](OC(=O)c3cc(O)c([O-])c(O)c3)O[C@@H]1COC(=O)c4cc(O)c(O)c(O)c4 |
| 365 | O[C@H]1[C@H](O)[C@@H](OC(=O)c2cc(O)c([O-])c(O)c2)[C@H](OC(=O)c3cc(O)c([O-])c(O)c3)O[C@@H]1COC(=O)c4cc(O)c([O-])c(O)c4 |
| 366 | O[C@@H]1[C@@H](COC(=O)c2cc(O)c(O)c(O)c2)O[C@@H](OC(=O)c3cc(O)c(O)c(O)c3)[C@H](O)[C@H]1OC(=O)c4cc(O)c(O)c(O)c4 |
| 367 | O[C@@H]1[C@@H](COC(=O)c2cc(O)c(O)c(O)c2)O[C@@H](OC(=O)c3cc(O)c(O)c(O)c3)[C@H](O)[C@H]1OC(=O)c4cc(O)c([O-])c(O)c4 |
| 368 | O[C@@H]1[C@@H](COC(=O)c2cc(O)c(O)c(O)c2)O[C@@H](OC(=O)c3cc(O)c([O-])c(O)c3)[C@H](O)[C@H]1OC(=O)c4cc(O)c(O)c(O)c4 |
| 369 | O[C@@H]1[C@@H](COC(=O)c2cc(O)c(O)c(O)c2)O[C@@H](OC(=O)c3cc(O)c([O-])c(O)c3)[C@H](O)[C@H]1OC(=O)c4cc(O)c([O-])c(O)c4 |
| 370 | O[C@@H]1[C@@H](COC(=O)c2cc(O)c([O-])c(O)c2)O[C@@H](OC(=O)c3cc(O)c([O-])c(O)c3)[C@H](O)[C@H]1OC(=O)c4cc(O)c([O-])c(O)c4 |
| 371 | CC(=C)[C@@H]1CC[C@@]2(CC[C@]3(C)[C@H](CC[C@@H]4[C@@]5(C)CC[C@H](O)C(C)(C)[C@@H]5CC[C@@]34C)[C@@H]12)C(=O)O |
| 372 | CC(=C)[C@@H]1CC[C@@]2(CC[C@]3(C)[C@H](CC[C@@H]4[C@@]5(C)CC[C@H](O)C(C)(C)[C@@H]5CC[C@@]34C)[C@@H]12)C(=O)[O-] |
| 373 | CC1(C)CC[C@@]2(CC[C@]3(C)C(=CC[C@@H]4[C@@]5(C)CC[C@H](O)C(C)(C)[C@@H]5CC[C@@]34C)[C@@H]2C1)C(=O)O |
| 374 | CC1(C)CC[C@@]2(CC[C@]3(C)C(=CC[C@@H]4[C@@]5(C)CC[C@H](O)C(C)(C)[C@@H]5CC[C@@]34C)[C@@H]2C1)C(=O)[O-] |
| 375 | CC[C@H](CC[C@@H](C)[C@H]1CC[C@H]2[C@@H]3CC=C4C[C@H](CC[C@]4(C)[C@H]3CC[C@]12C)O[C@@H]5O[C@H](CO)[C@@H](O)[C@H](O)[C@H]5O)C(C)C |
| 376 | Oc1ccc(cc1)C2=C([O-])C(=O)c3c(O)cc([O-])cc3O2 |
| 377 | Oc1cc([O-])cc2OC(=C([O-])C(=O)c12)c3ccc([O-])cc3 |
| 378 | O[C@H]1Cc2c(O)cc(O)cc2O[C@@H]1c3ccc(O)c(O)c3 |
| 379 | O[C@H]1Cc2c(O)cc([O-])cc2O[C@@H]1c3ccc(O)c(O)c3 |
| 380 | CC1(C)CC[C@@]2(CC[C@]3(C)C(=CC[C@@H]4[C@@]5(C)CC[C@H](O)[C@@](C)(CO)[C@@H]5CC[C@@]34C)[C@@H]2C1)C(=O)O |
| 381 | CC1(C)CC[C@@]2(CC[C@]3(C)C(=CC[C@@H]4[C@@]5(C)CC[C@H](O)[C@@](C)(CO)[C@@H]5CC[C@@]34C)[C@@H]2C1)C(=O)[O-] |
| 382 | OC[C@H]1O[C@@H](OC2=C(Oc3cc(O)cc(O)c3C2=O)c4ccc(O)cc4)[C@H](O)[C@@H](O)[C@@H]1O |
| 383 | OC[C@H]1O[C@@H](OC2=C(Oc3cc(O)cc(O)c3C2=O)c4ccc([O-])cc4)[C@H](O)[C@@H](O)[C@@H]1O |
| 384 | CCCCCCCCCCCCC |
| 385 | C[C@@H]1CC[C@H]2C(C)(C)[C@H]3C[C@@]12CC=C3C |
| 386 | OC[C@H]1O[C@H](O[C@]2(CO)O[C@H](CO)[C@@H](O)[C@@H]2O)[C@H](O)[C@@H](O)[C@@H]1O |
| 387 | CCCCCCCCCCCCCCCCCCCC |
| 388 | CCCCCCCCCCCCCCCCCCCCC |
| 389 | CC(C)CCC[C@@H](C)CCC[C@@H](C)CCCC(=O)C |
| 390 | CCCCCCCCCCCCO |
| 391 | CCCCCCCC\C=C/CCCCCCCCO |
| 392 | CC1(C)[C@@H]2CCC(=O)[C@H]1C2 |
| 393 | CCCCCCCCCCCCCCCCCCCCCCCCCCCC |
| 394 | CCCCCCCCCCCC=O |
| 395 | Oc1ccccc1C(=O)[O-] |
| 396 | CC1(C)CCCC[C@@H]1O |
| 397 | CCCCC\C=C\C\C=C\CCCCCCCC(=O)OC |
| 398 | CCCCCCCC\C=C\CCCCCCCC |
| 399 | C=C1c2ccccc2c3ccccc13 |
| 400 | CCCOC(=O)c1ccccc1C(=O)OCCC |
| 401 | C[C@@H](O)[C@@H](C)O |
| 402 | CCCCCCCCCCCCCCCCCCCCCCCCCCCCCCCC |
| 403 | CC(C)(C)c1cccc(c1O)C(C)(C)C |
| 404 | CC1(C)[C@@H]2CC=C(CO)[C@H]1C2 |
| 405 | CC1(C)[C@@H]2CC[C@@H](CO)[C@H]1C2 |
| 406 | CC(=O)OC(=O)C |
| 407 | CC(=C)[C@@H]1CC[C@@]2(CC[C@]3(C)[C@H](CC[C@@H]4[C@@]5(C)CC[C@H](O)[C@@](C)(CO)[C@@H]5CC[C@@]34C)[C@@H]12)C(=O)O |
| 408 | CC(=C)[C@@H]1CC[C@@]2(CC[C@]3(C)[C@H](CC[C@@H]4[C@@]5(C)CC[C@H](O)[C@@](C)(CO)[C@@H]5CC[C@@]34C)[C@@H]12)C(=O)[O-] |
| 409 | CC1(C)CC[C@@]2(CC[C@]3(C)C(=C2C1)C=C[C@@H]4[C@@]5(C)CC[C@H](O)[C@@](C)(CO)[C@@H]5CC[C@@]34C)C(=O)[O-] |
| 410 | CC1(C)CC[C@@]2(CC[C@]3(C)C(=CC(=O)[C@@H]4[C@@]5(C)CC[C@H](O)C(C)(C)[C@H]5CC[C@@]34C)[C@H]2C1)C(=O)O |
| 411 | CC1(C)CC[C@@]2(CC[C@]3(C)C(=CC(=O)[C@@H]4[C@@]5(C)CC[C@H](O)C(C)(C)[C@H]5CC[C@@]34C)[C@H]2C1)C(=O)[O-] |
| 412 | Cc1c(O)cccc1CC=C |
| 413 | CC1(C)CC[C@@]2(CC[C@]3(C)C(=C2C1)C=C[C@@H]4[C@@]5(C)CC[C@H](O)C(C)(C)[C@@H]5CC[C@@]34C)C(=O)[O-] |
| 414 | COC(=O)c1cc(O)c(O)c([O-])c1 |
| 415 | CCOC(=O)c1cc(O)c(O)c([O-])c1 |
| 416 | CC1(C)[C@@H]2CC\C(=C\O[C@@H]3O[C@H](CO[C@@H]4OC[C@H](O)[C@H](O)[C@H]4O)[C@@H](O)[C@H](O)[C@H]3O)\[C@H]1C2 |
| 417 | CC1(C)[C@@H]2CC\C(=C\O)\[C@H]1C2 |
| 418 | CC1=CC[C@]23CC[C@]4(C)[C@H]([C@H]2C1)[C@]5(O[C@H]5[C@@H]6[C@@]7(C)CC[C@H](O)[C@@](C)(CO)[C@H]7CC[C@@]46C)OC3=O |
| 419 | C[C@]1(O[C@@H]2O[C@H](CO)[C@@H](O)[C@H](O)[C@H]2O)O[C@@H]3CC(=O)[C@@H]4C[C@@]1(O)[C@]34COC(=O)c5ccccc5 |
| 420 | C[C@]1(O)O[C@@H]2CC(=O)[C@@H]3C[C@@]1(O)[C@]23COC(=O)c4ccccc4 |
| 421 | CCCOC(=O)[C@@H](C)O |
| 422 | Oc1cc(cc(O)c1O)C(=O)Oc2cc(cc(O)c2O)C(=O)OC[C@@H]3O[C@@H](OC(=O)c4cc(O)c(O)c(OC(=O)c5cc(O)c(O)c(O)c5)c4)[C@H](OC(=O)c6cc(O)c(O)c(OC(=O)c7cc(O)c(O)c(O)c7)c6)[C@@H](OC(=O)c8cc(O)c(O)c(OC(=O)c9cc(O)c(O)c(O)c9)c8)[C@@H]3OC(=O)c%10cc(O)c(O)c(OC(=O)c%11cc(O)c(O)c(O)c%11)c%10 |
| 423 | Oc1cc(cc(O)c1O)C(=O)Oc2cc(cc(O)c2O)C(=O)OC[C@@H]3O[C@@H](OC(=O)c4cc(O)c([O-])c(OC(=O)c5cc(O)c(O)c(O)c5)c4)[C@H](OC(=O)c6cc(O)c(O)c(OC(=O)c7cc(O)c(O)c(O)c7)c6)[C@@H](OC(=O)c8cc(O)c(O)c(OC(=O)c9cc(O)c(O)c(O)c9)c8)[C@@H]3OC(=O)c%10cc(O)c(O)c(OC(=O)c%11cc(O)c(O)c(O)c%11)c%10 |
| 424 | Oc1cc(cc(O)c1O)C(=O)Oc2cc(cc(O)c2O)C(=O)OC[C@@H]3O[C@@H](OC(=O)c4cc(O)c(O)c(OC(=O)c5cc(O)c(O)c(O)c5)c4)[C@H](OC(=O)c6cc(O)c([O-])c(OC(=O)c7cc(O)c(O)c(O)c7)c6)[C@@H](OC(=O)c8cc(O)c(O)c(OC(=O)c9cc(O)c(O)c(O)c9)c8)[C@@H]3OC(=O)c%10cc(O)c(O)c(OC(=O)c%11cc(O)c(O)c(O)c%11)c%10 |
| 425 | Oc1cc(cc(O)c1O)C(=O)Oc2cc(cc(O)c2O)C(=O)OC[C@@H]3O[C@@H](OC(=O)c4cc(O)c([O-])c(OC(=O)c5cc(O)c(O)c(O)c5)c4)[C@H](OC(=O)c6cc(O)c([O-])c(OC(=O)c7cc(O)c(O)c(O)c7)c6)[C@@H](OC(=O)c8cc(O)c(O)c(OC(=O)c9cc(O)c(O)c(O)c9)c8)[C@@H]3OC(=O)c%10cc(O)c(O)c(OC(=O)c%11cc(O)c(O)c(O)c%11)c%10 |
| 426 | Oc1cc(cc(O)c1O)C(=O)Oc2cc(cc(O)c2O)C(=O)OC[C@@H]3O[C@@H](OC(=O)c4cc(O)c(O)c(OC(=O)c5cc(O)c(O)c(O)c5)c4)[C@H](OC(=O)c6cc(O)c(O)c(OC(=O)c7cc(O)c(O)c(O)c7)c6)[C@@H](OC(=O)c8cc(O)c(O)c(OC(=O)c9cc(O)c(O)c(O)c9)c8)[C@@H]3OC(=O)c%10cc(O)c([O-])c(OC(=O)c%11cc(O)c(O)c(O)c%11)c%10 |
| 427 | Oc1cc(cc(O)c1O)C(=O)Oc2cc(cc(O)c2O)C(=O)OC[C@@H]3O[C@@H](OC(=O)c4cc(O)c([O-])c(OC(=O)c5cc(O)c(O)c(O)c5)c4)[C@H](OC(=O)c6cc(O)c(O)c(OC(=O)c7cc(O)c(O)c(O)c7)c6)[C@@H](OC(=O)c8cc(O)c(O)c(OC(=O)c9cc(O)c(O)c(O)c9)c8)[C@@H]3OC(=O)c%10cc(O)c([O-])c(OC(=O)c%11cc(O)c(O)c(O)c%11)c%10 |
| 428 | Oc1cc(cc(O)c1O)C(=O)Oc2cc(cc(O)c2O)C(=O)OC[C@@H]3O[C@@H](OC(=O)c4cc(O)c(O)c(OC(=O)c5cc(O)c(O)c(O)c5)c4)[C@H](OC(=O)c6cc(O)c([O-])c(OC(=O)c7cc(O)c(O)c(O)c7)c6)[C@@H](OC(=O)c8cc(O)c(O)c(OC(=O)c9cc(O)c(O)c(O)c9)c8)[C@@H]3OC(=O)c%10cc(O)c([O-])c(OC(=O)c%11cc(O)c(O)c(O)c%11)c%10 |
| 429 | Oc1cc(cc(O)c1O)C(=O)Oc2cc(cc(O)c2O)C(=O)OC[C@@H]3O[C@@H](OC(=O)c4cc(O)c([O-])c(OC(=O)c5cc(O)c(O)c(O)c5)c4)[C@H](OC(=O)c6cc(O)c([O-])c(OC(=O)c7cc(O)c(O)c(O)c7)c6)[C@@H](OC(=O)c8cc(O)c(O)c(OC(=O)c9cc(O)c(O)c(O)c9)c8)[C@@H]3OC(=O)c%10cc(O)c([O-])c(OC(=O)c%11cc(O)c(O)c(O)c%11)c%10 |
| 430 | Oc1cc(cc(O)c1O)C(=O)Oc2cc(cc(O)c2O)C(=O)OC[C@@H]3O[C@@H](OC(=O)c4cc(O)c(O)c(OC(=O)c5cc(O)c(O)c(O)c5)c4)[C@H](OC(=O)c6cc(O)c(O)c(OC(=O)c7cc(O)c(O)c(O)c7)c6)[C@@H](OC(=O)c8cc(O)c([O-])c(OC(=O)c9cc(O)c(O)c(O)c9)c8)[C@@H]3OC(=O)c%10cc(O)c(O)c(OC(=O)c%11cc(O)c(O)c(O)c%11)c%10 |
| 431 | Oc1cc(cc(O)c1O)C(=O)Oc2cc(cc(O)c2O)C(=O)OC[C@@H]3O[C@@H](OC(=O)c4cc(O)c([O-])c(OC(=O)c5cc(O)c(O)c(O)c5)c4)[C@H](OC(=O)c6cc(O)c(O)c(OC(=O)c7cc(O)c(O)c(O)c7)c6)[C@@H](OC(=O)c8cc(O)c([O-])c(OC(=O)c9cc(O)c(O)c(O)c9)c8)[C@@H]3OC(=O)c%10cc(O)c(O)c(OC(=O)c%11cc(O)c(O)c(O)c%11)c%10 |
| 432 | Oc1cc(cc(O)c1O)C(=O)Oc2cc(cc(O)c2O)C(=O)OC[C@@H]3O[C@@H](OC(=O)c4cc(O)c(O)c(OC(=O)c5cc(O)c(O)c(O)c5)c4)[C@H](OC(=O)c6cc(O)c([O-])c(OC(=O)c7cc(O)c(O)c(O)c7)c6)[C@@H](OC(=O)c8cc(O)c([O-])c(OC(=O)c9cc(O)c(O)c(O)c9)c8)[C@@H]3OC(=O)c%10cc(O)c(O)c(OC(=O)c%11cc(O)c(O)c(O)c%11)c%10 |
| 433 | Oc1cc(cc(O)c1O)C(=O)Oc2cc(cc(O)c2O)C(=O)OC[C@@H]3O[C@@H](OC(=O)c4cc(O)c([O-])c(OC(=O)c5cc(O)c(O)c(O)c5)c4)[C@H](OC(=O)c6cc(O)c([O-])c(OC(=O)c7cc(O)c(O)c(O)c7)c6)[C@@H](OC(=O)c8cc(O)c([O-])c(OC(=O)c9cc(O)c(O)c(O)c9)c8)[C@@H]3OC(=O)c%10cc(O)c(O)c(OC(=O)c%11cc(O)c(O)c(O)c%11)c%10 |
| 434 | Oc1cc(cc(O)c1O)C(=O)Oc2cc(cc(O)c2O)C(=O)OC[C@@H]3O[C@@H](OC(=O)c4cc(O)c(O)c(OC(=O)c5cc(O)c(O)c(O)c5)c4)[C@H](OC(=O)c6cc(O)c(O)c(OC(=O)c7cc(O)c(O)c(O)c7)c6)[C@@H](OC(=O)c8cc(O)c([O-])c(OC(=O)c9cc(O)c(O)c(O)c9)c8)[C@@H]3OC(=O)c%10cc(O)c([O-])c(OC(=O)c%11cc(O)c(O)c(O)c%11)c%10 |
| 435 | Oc1cc(cc(O)c1O)C(=O)Oc2cc(cc(O)c2O)C(=O)OC[C@@H]3O[C@@H](OC(=O)c4cc(O)c([O-])c(OC(=O)c5cc(O)c(O)c(O)c5)c4)[C@H](OC(=O)c6cc(O)c(O)c(OC(=O)c7cc(O)c(O)c(O)c7)c6)[C@@H](OC(=O)c8cc(O)c([O-])c(OC(=O)c9cc(O)c(O)c(O)c9)c8)[C@@H]3OC(=O)c%10cc(O)c([O-])c(OC(=O)c%11cc(O)c(O)c(O)c%11)c%10 |
| 436 | Oc1cc(cc(O)c1O)C(=O)Oc2cc(cc(O)c2O)C(=O)OC[C@@H]3O[C@@H](OC(=O)c4cc(O)c(O)c(OC(=O)c5cc(O)c(O)c(O)c5)c4)[C@H](OC(=O)c6cc(O)c([O-])c(OC(=O)c7cc(O)c(O)c(O)c7)c6)[C@@H](OC(=O)c8cc(O)c([O-])c(OC(=O)c9cc(O)c(O)c(O)c9)c8)[C@@H]3OC(=O)c%10cc(O)c([O-])c(OC(=O)c%11cc(O)c(O)c(O)c%11)c%10 |
| 437 | Oc1cc(cc(O)c1O)C(=O)Oc2cc(cc(O)c2O)C(=O)OC[C@@H]3O[C@@H](OC(=O)c4cc(O)c([O-])c(OC(=O)c5cc(O)c(O)c(O)c5)c4)[C@H](OC(=O)c6cc(O)c([O-])c(OC(=O)c7cc(O)c(O)c(O)c7)c6)[C@@H](OC(=O)c8cc(O)c([O-])c(OC(=O)c9cc(O)c(O)c(O)c9)c8)[C@@H]3OC(=O)c%10cc(O)c([O-])c(OC(=O)c%11cc(O)c(O)c(O)c%11)c%10 |
| 438 | Oc1cc(cc(O)c1O)C(=O)Oc2cc(cc(O)c2O)C(=O)OC[C@@H]3O[C@@H](OC(=O)c4cc(O)c([O-])c(OC(=O)c5cc(O)c(O)c(O)c5)c4)[C@H](OC(=O)c6cc(O)c([O-])c(OC(=O)c7cc(O)c(O)c(O)c7)c6)[C@@H](OC(=O)c8cc(O)c([O-])c(OC(=O)c9cc(O)c([O-])c(O)c9)c8)[C@@H]3OC(=O)c%10cc(O)c([O-])c(OC(=O)c%11cc(O)c(O)c(O)c%11)c%10 |
| 439 | Oc1cc(cc(O)c1O)C(=O)Oc2cc(cc(O)c2O)C(=O)OC[C@@H]3O[C@@H](OC(=O)c4cc(O)c([O-])c(OC(=O)c5cc(O)c(O)c(O)c5)c4)[C@H](OC(=O)c6cc(O)c([O-])c(OC(=O)c7cc(O)c(O)c(O)c7)c6)[C@@H](OC(=O)c8cc(O)c([O-])c(OC(=O)c9cc(O)c(O)c(O)c9)c8)[C@@H]3OC(=O)c%10cc(O)c([O-])c(OC(=O)c%11cc(O)c([O-])c(O)c%11)c%10 |
| 440 | Oc1cc(cc(O)c1O)C(=O)Oc2cc(cc(O)c2O)C(=O)OC[C@@H]3O[C@@H](OC(=O)c4cc(O)c([O-])c(OC(=O)c5cc(O)c(O)c(O)c5)c4)[C@H](OC(=O)c6cc(O)c([O-])c(OC(=O)c7cc(O)c(O)c(O)c7)c6)[C@@H](OC(=O)c8cc(O)c([O-])c(OC(=O)c9cc(O)c([O-])c(O)c9)c8)[C@@H]3OC(=O)c%10cc(O)c([O-])c(OC(=O)c%11cc(O)c([O-])c(O)c%11)c%10 |
| 441 | Oc1cc(cc(O)c1O)C(=O)Oc2cc(cc(O)c2O)C(=O)OC[C@@H]3O[C@@H](OC(=O)c4cc(O)c([O-])c(OC(=O)c5cc(O)c(O)c(O)c5)c4)[C@H](OC(=O)c6cc(O)c([O-])c(OC(=O)c7cc(O)c([O-])c(O)c7)c6)[C@@H](OC(=O)c8cc(O)c([O-])c(OC(=O)c9cc(O)c(O)c(O)c9)c8)[C@@H]3OC(=O)c%10cc(O)c([O-])c(OC(=O)c%11cc(O)c(O)c(O)c%11)c%10 |
| 442 | Oc1cc(cc(O)c1O)C(=O)Oc2cc(cc(O)c2O)C(=O)OC[C@@H]3O[C@@H](OC(=O)c4cc(O)c([O-])c(OC(=O)c5cc(O)c(O)c(O)c5)c4)[C@H](OC(=O)c6cc(O)c([O-])c(OC(=O)c7cc(O)c([O-])c(O)c7)c6)[C@@H](OC(=O)c8cc(O)c([O-])c(OC(=O)c9cc(O)c([O-])c(O)c9)c8)[C@@H]3OC(=O)c%10cc(O)c([O-])c(OC(=O)c%11cc(O)c(O)c(O)c%11)c%10 |
| 443 | Oc1cc(cc(O)c1O)C(=O)Oc2cc(cc(O)c2O)C(=O)OC[C@@H]3O[C@@H](OC(=O)c4cc(O)c([O-])c(OC(=O)c5cc(O)c(O)c(O)c5)c4)[C@H](OC(=O)c6cc(O)c([O-])c(OC(=O)c7cc(O)c([O-])c(O)c7)c6)[C@@H](OC(=O)c8cc(O)c([O-])c(OC(=O)c9cc(O)c(O)c(O)c9)c8)[C@@H]3OC(=O)c%10cc(O)c([O-])c(OC(=O)c%11cc(O)c([O-])c(O)c%11)c%10 |
| 444 | Oc1cc(cc(O)c1O)C(=O)Oc2cc(cc(O)c2O)C(=O)OC[C@@H]3O[C@@H](OC(=O)c4cc(O)c([O-])c(OC(=O)c5cc(O)c(O)c(O)c5)c4)[C@H](OC(=O)c6cc(O)c([O-])c(OC(=O)c7cc(O)c([O-])c(O)c7)c6)[C@@H](OC(=O)c8cc(O)c([O-])c(OC(=O)c9cc(O)c([O-])c(O)c9)c8)[C@@H]3OC(=O)c%10cc(O)c([O-])c(OC(=O)c%11cc(O)c([O-])c(O)c%11)c%10 |
| 445 | Oc1cc(cc(O)c1O)C(=O)Oc2cc(cc(O)c2O)C(=O)OC[C@@H]3O[C@@H](OC(=O)c4cc(O)c([O-])c(OC(=O)c5cc(O)c([O-])c(O)c5)c4)[C@H](OC(=O)c6cc(O)c([O-])c(OC(=O)c7cc(O)c(O)c(O)c7)c6)[C@@H](OC(=O)c8cc(O)c([O-])c(OC(=O)c9cc(O)c(O)c(O)c9)c8)[C@@H]3OC(=O)c%10cc(O)c([O-])c(OC(=O)c%11cc(O)c(O)c(O)c%11)c%10 |
| 446 | Oc1cc(cc(O)c1O)C(=O)Oc2cc(cc(O)c2O)C(=O)OC[C@@H]3O[C@@H](OC(=O)c4cc(O)c([O-])c(OC(=O)c5cc(O)c([O-])c(O)c5)c4)[C@H](OC(=O)c6cc(O)c([O-])c(OC(=O)c7cc(O)c(O)c(O)c7)c6)[C@@H](OC(=O)c8cc(O)c([O-])c(OC(=O)c9cc(O)c([O-])c(O)c9)c8)[C@@H]3OC(=O)c%10cc(O)c([O-])c(OC(=O)c%11cc(O)c(O)c(O)c%11)c%10 |
| 447 | Oc1cc(cc(O)c1O)C(=O)Oc2cc(cc(O)c2O)C(=O)OC[C@@H]3O[C@@H](OC(=O)c4cc(O)c([O-])c(OC(=O)c5cc(O)c([O-])c(O)c5)c4)[C@H](OC(=O)c6cc(O)c([O-])c(OC(=O)c7cc(O)c(O)c(O)c7)c6)[C@@H](OC(=O)c8cc(O)c([O-])c(OC(=O)c9cc(O)c(O)c(O)c9)c8)[C@@H]3OC(=O)c%10cc(O)c([O-])c(OC(=O)c%11cc(O)c([O-])c(O)c%11)c%10 |
| 448 | Oc1cc(cc(O)c1O)C(=O)Oc2cc(cc(O)c2O)C(=O)OC[C@@H]3O[C@@H](OC(=O)c4cc(O)c([O-])c(OC(=O)c5cc(O)c([O-])c(O)c5)c4)[C@H](OC(=O)c6cc(O)c([O-])c(OC(=O)c7cc(O)c(O)c(O)c7)c6)[C@@H](OC(=O)c8cc(O)c([O-])c(OC(=O)c9cc(O)c([O-])c(O)c9)c8)[C@@H]3OC(=O)c%10cc(O)c([O-])c(OC(=O)c%11cc(O)c([O-])c(O)c%11)c%10 |
| 449 | Oc1cc(cc(O)c1O)C(=O)Oc2cc(cc(O)c2O)C(=O)OC[C@@H]3O[C@@H](OC(=O)c4cc(O)c([O-])c(OC(=O)c5cc(O)c([O-])c(O)c5)c4)[C@H](OC(=O)c6cc(O)c([O-])c(OC(=O)c7cc(O)c([O-])c(O)c7)c6)[C@@H](OC(=O)c8cc(O)c([O-])c(OC(=O)c9cc(O)c(O)c(O)c9)c8)[C@@H]3OC(=O)c%10cc(O)c([O-])c(OC(=O)c%11cc(O)c(O)c(O)c%11)c%10 |
| 450 | Oc1cc(cc(O)c1O)C(=O)Oc2cc(cc(O)c2O)C(=O)OC[C@@H]3O[C@@H](OC(=O)c4cc(O)c([O-])c(OC(=O)c5cc(O)c([O-])c(O)c5)c4)[C@H](OC(=O)c6cc(O)c([O-])c(OC(=O)c7cc(O)c([O-])c(O)c7)c6)[C@@H](OC(=O)c8cc(O)c([O-])c(OC(=O)c9cc(O)c([O-])c(O)c9)c8)[C@@H]3OC(=O)c%10cc(O)c([O-])c(OC(=O)c%11cc(O)c(O)c(O)c%11)c%10 |
| 451 | Oc1cc(cc(O)c1O)C(=O)Oc2cc(cc(O)c2O)C(=O)OC[C@@H]3O[C@@H](OC(=O)c4cc(O)c([O-])c(OC(=O)c5cc(O)c([O-])c(O)c5)c4)[C@H](OC(=O)c6cc(O)c([O-])c(OC(=O)c7cc(O)c([O-])c(O)c7)c6)[C@@H](OC(=O)c8cc(O)c([O-])c(OC(=O)c9cc(O)c(O)c(O)c9)c8)[C@@H]3OC(=O)c%10cc(O)c([O-])c(OC(=O)c%11cc(O)c([O-])c(O)c%11)c%10 |
| 452 | Oc1cc(cc(O)c1O)C(=O)Oc2cc(cc(O)c2O)C(=O)OC[C@@H]3O[C@@H](OC(=O)c4cc(O)c([O-])c(OC(=O)c5cc(O)c([O-])c(O)c5)c4)[C@H](OC(=O)c6cc(O)c([O-])c(OC(=O)c7cc(O)c([O-])c(O)c7)c6)[C@@H](OC(=O)c8cc(O)c([O-])c(OC(=O)c9cc(O)c([O-])c(O)c9)c8)[C@@H]3OC(=O)c%10cc(O)c([O-])c(OC(=O)c%11cc(O)c([O-])c(O)c%11)c%10 |
| 453 | Oc1cc(cc(O)c1O)C(=O)Oc2cc(cc(O)c2[O-])C(=O)O[C@@H]3O[C@@H](COC(=O)c4cc(O)c(O)c(OC(=O)c5cc(O)c([O-])c(O)c5)c4)[C@@H](OC(=O)c6cc(O)c([O-])c(OC(=O)c7cc(O)c(O)c(O)c7)c6)[C@H](OC(=O)c8cc(O)c([O-])c(OC(=O)c9cc(O)c(O)c(O)c9)c8)[C@H]3OC(=O)c%10cc(O)c([O-])c(OC(=O)c%11cc(O)c(O)c(O)c%11)c%10 |
| 454 | Oc1cc(cc(O)c1O)C(=O)Oc2cc(cc(O)c2[O-])C(=O)O[C@@H]3O[C@@H](COC(=O)c4cc(O)c(O)c(OC(=O)c5cc(O)c([O-])c(O)c5)c4)[C@@H](OC(=O)c6cc(O)c([O-])c(OC(=O)c7cc(O)c(O)c(O)c7)c6)[C@H](OC(=O)c8cc(O)c([O-])c(OC(=O)c9cc(O)c([O-])c(O)c9)c8)[C@H]3OC(=O)c%10cc(O)c([O-])c(OC(=O)c%11cc(O)c(O)c(O)c%11)c%10 |
| 455 | Oc1cc(cc(O)c1O)C(=O)Oc2cc(cc(O)c2[O-])C(=O)O[C@@H]3O[C@@H](COC(=O)c4cc(O)c(O)c(OC(=O)c5cc(O)c([O-])c(O)c5)c4)[C@@H](OC(=O)c6cc(O)c([O-])c(OC(=O)c7cc(O)c([O-])c(O)c7)c6)[C@H](OC(=O)c8cc(O)c([O-])c(OC(=O)c9cc(O)c(O)c(O)c9)c8)[C@H]3OC(=O)c%10cc(O)c([O-])c(OC(=O)c%11cc(O)c(O)c(O)c%11)c%10 |
| 456 | Oc1cc(cc(O)c1O)C(=O)Oc2cc(cc(O)c2[O-])C(=O)O[C@@H]3O[C@@H](COC(=O)c4cc(O)c(O)c(OC(=O)c5cc(O)c([O-])c(O)c5)c4)[C@@H](OC(=O)c6cc(O)c([O-])c(OC(=O)c7cc(O)c([O-])c(O)c7)c6)[C@H](OC(=O)c8cc(O)c([O-])c(OC(=O)c9cc(O)c([O-])c(O)c9)c8)[C@H]3OC(=O)c%10cc(O)c([O-])c(OC(=O)c%11cc(O)c(O)c(O)c%11)c%10 |
| 457 | Oc1cc(cc(O)c1O)C(=O)Oc2cc(cc(O)c2[O-])C(=O)O[C@@H]3O[C@@H](COC(=O)c4cc(O)c(O)c(OC(=O)c5cc(O)c([O-])c(O)c5)c4)[C@@H](OC(=O)c6cc(O)c([O-])c(OC(=O)c7cc(O)c(O)c(O)c7)c6)[C@H](OC(=O)c8cc(O)c([O-])c(OC(=O)c9cc(O)c(O)c(O)c9)c8)[C@H]3OC(=O)c%10cc(O)c([O-])c(OC(=O)c%11cc(O)c([O-])c(O)c%11)c%10 |
| 458 | Oc1cc(cc(O)c1O)C(=O)Oc2cc(cc(O)c2[O-])C(=O)O[C@@H]3O[C@@H](COC(=O)c4cc(O)c(O)c(OC(=O)c5cc(O)c([O-])c(O)c5)c4)[C@@H](OC(=O)c6cc(O)c([O-])c(OC(=O)c7cc(O)c(O)c(O)c7)c6)[C@H](OC(=O)c8cc(O)c([O-])c(OC(=O)c9cc(O)c([O-])c(O)c9)c8)[C@H]3OC(=O)c%10cc(O)c([O-])c(OC(=O)c%11cc(O)c([O-])c(O)c%11)c%10 |
| 459 | Oc1cc(cc(O)c1O)C(=O)Oc2cc(cc(O)c2[O-])C(=O)O[C@@H]3O[C@@H](COC(=O)c4cc(O)c(O)c(OC(=O)c5cc(O)c([O-])c(O)c5)c4)[C@@H](OC(=O)c6cc(O)c([O-])c(OC(=O)c7cc(O)c([O-])c(O)c7)c6)[C@H](OC(=O)c8cc(O)c([O-])c(OC(=O)c9cc(O)c(O)c(O)c9)c8)[C@H]3OC(=O)c%10cc(O)c([O-])c(OC(=O)c%11cc(O)c([O-])c(O)c%11)c%10 |
| 460 | Oc1cc(cc(O)c1O)C(=O)Oc2cc(cc(O)c2[O-])C(=O)O[C@@H]3O[C@@H](COC(=O)c4cc(O)c(O)c(OC(=O)c5cc(O)c([O-])c(O)c5)c4)[C@@H](OC(=O)c6cc(O)c([O-])c(OC(=O)c7cc(O)c([O-])c(O)c7)c6)[C@H](OC(=O)c8cc(O)c([O-])c(OC(=O)c9cc(O)c([O-])c(O)c9)c8)[C@H]3OC(=O)c%10cc(O)c([O-])c(OC(=O)c%11cc(O)c([O-])c(O)c%11)c%10 |
| 461 | Oc1cc(cc(O)c1O)C(=O)Oc2cc(cc(O)c2[O-])C(=O)O[C@@H]3[C@H](COC(=O)c4cc(O)c(O)c(OC(=O)c5cc(O)c([O-])c(O)c5)c4)O[C@@H](OC(=O)c6cc(O)c([O-])c(OC(=O)c7cc(O)c([O-])c(O)c7)c6)[C@H](OC(=O)c8cc(O)c([O-])c(OC(=O)c9cc(O)c(O)c(O)c9)c8)[C@H]3OC(=O)c%10cc(O)c([O-])c(OC(=O)c%11cc(O)c(O)c(O)c%11)c%10 |
| 462 | Oc1cc(cc(O)c1O)C(=O)Oc2cc(cc(O)c2[O-])C(=O)O[C@@H]3[C@H](COC(=O)c4cc(O)c(O)c(OC(=O)c5cc(O)c([O-])c(O)c5)c4)O[C@@H](OC(=O)c6cc(O)c([O-])c(OC(=O)c7cc(O)c([O-])c(O)c7)c6)[C@H](OC(=O)c8cc(O)c([O-])c(OC(=O)c9cc(O)c(O)c(O)c9)c8)[C@H]3OC(=O)c%10cc(O)c([O-])c(OC(=O)c%11cc(O)c([O-])c(O)c%11)c%10 |
| 463 | Oc1cc(cc(O)c1O)C(=O)Oc2cc(cc(O)c2[O-])C(=O)O[C@H]3[C@H](OC(=O)c4cc(O)c([O-])c(OC(=O)c5cc(O)c([O-])c(O)c5)c4)O[C@@H](COC(=O)c6cc(O)c(O)c(OC(=O)c7cc(O)c([O-])c(O)c7)c6)[C@@H](OC(=O)c8cc(O)c([O-])c(OC(=O)c9cc(O)c([O-])c(O)c9)c8)[C@@H]3OC(=O)c%10cc(O)c([O-])c(OC(=O)c%11cc(O)c(O)c(O)c%11)c%10 |
| 464 | Oc1cc(cc(O)c1O)C(=O)Oc2cc(cc(O)c2[O-])C(=O)O[C@H]3[C@H](OC(=O)c4cc(O)c([O-])c(OC(=O)c5cc(O)c([O-])c(O)c5)c4)O[C@@H](COC(=O)c6cc(O)c(O)c(OC(=O)c7cc(O)c([O-])c(O)c7)c6)[C@@H](OC(=O)c8cc(O)c([O-])c(OC(=O)c9cc(O)c([O-])c(O)c9)c8)[C@@H]3OC(=O)c%10cc(O)c([O-])c(OC(=O)c%11cc(O)c([O-])c(O)c%11)c%10 |
| 465 | Oc1cc(cc(O)c1O)C(=O)Oc2cc(cc(O)c2[O-])C(=O)O[C@@H]3[C@H](COC(=O)c4cc(O)c(O)c(OC(=O)c5cc(O)c([O-])c(O)c5)c4)O[C@@H](OC(=O)c6cc(O)c([O-])c(OC(=O)c7cc(O)c([O-])c(O)c7)c6)[C@H](OC(=O)c8cc(O)c([O-])c(OC(=O)c9cc(O)c([O-])c(O)c9)c8)[C@H]3OC(=O)c%10cc(O)c([O-])c(OC(=O)c%11cc(O)c(O)c(O)c%11)c%10 |
| 466 | Oc1cc(cc(O)c1O)C(=O)Oc2cc(cc(O)c2[O-])C(=O)O[C@@H]3[C@H](COC(=O)c4cc(O)c(O)c(OC(=O)c5cc(O)c([O-])c(O)c5)c4)O[C@@H](OC(=O)c6cc(O)c([O-])c(OC(=O)c7cc(O)c([O-])c(O)c7)c6)[C@H](OC(=O)c8cc(O)c([O-])c(OC(=O)c9cc(O)c([O-])c(O)c9)c8)[C@H]3OC(=O)c%10cc(O)c([O-])c(OC(=O)c%11cc(O)c([O-])c(O)c%11)c%10 |
| 467 | Oc1cc(cc(O)c1O)C(=O)Oc2cc(cc(O)c2[O-])C(=O)O[C@H]3[C@H](OC(=O)c4cc(O)c([O-])c(OC(=O)c5cc(O)c([O-])c(O)c5)c4)[C@H](COC(=O)c6cc(O)c(O)c(OC(=O)c7cc(O)c([O-])c(O)c7)c6)O[C@@H](OC(=O)c8cc(O)c([O-])c(OC(=O)c9cc(O)c([O-])c(O)c9)c8)[C@@H]3OC(=O)c%10cc(O)c([O-])c(OC(=O)c%11cc(O)c([O-])c(O)c%11)c%10 |
| 468 | Oc1cc(cc(OC(=O)c2cc(O)c([O-])c(O)c2)c1O)C(=O)OC[C@@H]3O[C@@H](OC(=O)c4cc(O)c([O-])c(OC(=O)c5cc(O)c([O-])c(O)c5)c4)[C@H](OC(=O)c6cc(O)c([O-])c(OC(=O)c7cc(O)c([O-])c(O)c7)c6)[C@@H](OC(=O)c8cc(O)c([O-])c(OC(=O)c9cc(O)c([O-])c(O)c9)c8)[C@@H]3OC(=O)c%10cc(O)c([O-])c(OC(=O)c%11cc(O)c([O-])c(O)c%11)c%10 |
| 469 | Oc1cc(cc(O)c1[O-])C(=O)Oc2cc(cc(O)c2[O-])C(=O)OC[C@@H]3O[C@@H](OC(=O)c4cc(O)c([O-])c(OC(=O)c5cc(O)c([O-])c(O)c5)c4)[C@H](OC(=O)c6cc(O)c([O-])c(OC(=O)c7cc(O)c([O-])c(O)c7)c6)[C@@H](OC(=O)c8cc(O)c([O-])c(OC(=O)c9cc(O)c([O-])c(O)c9)c8)[C@@H]3OC(=O)c%10cc(O)c([O-])c(OC(=O)c%11cc(O)c([O-])c(O)c%11)c%10 |
| 470 | Oc1cc(cc(O)c1[O-])C(=O)Oc2cc(cc(O)c2[O-])C(=O)O[C@@H]3O[C@@H](COC(=O)c4cc(O)c([O-])c(OC(=O)c5cc(O)c([O-])c([O-])c5)c4)[C@@H](OC(=O)c6cc(O)c([O-])c(OC(=O)c7cc(O)c([O-])c(O)c7)c6)[C@H](OC(=O)c8cc(O)c([O-])c(OC(=O)c9cc(O)c([O-])c(O)c9)c8)[C@H]3OC(=O)c%10cc(O)c([O-])c(OC(=O)c%11cc(O)c([O-])c(O)c%11)c%10 |
| 471 | Oc1cc(cc(O)c1[O-])C(=O)Oc2cc(cc(O)c2[O-])C(=O)OC[C@@H]3O[C@@H](OC(=O)c4cc(O)c([O-])c(OC(=O)c5cc(O)c([O-])c([O-])c5)c4)[C@H](OC(=O)c6cc(O)c([O-])c(OC(=O)c7cc(O)c([O-])c(O)c7)c6)[C@@H](OC(=O)c8cc(O)c([O-])c(OC(=O)c9cc(O)c([O-])c(O)c9)c8)[C@@H]3OC(=O)c%10cc(O)c([O-])c(OC(=O)c%11cc(O)c([O-])c(O)c%11)c%10 |
| 472 | Oc1cc(cc(O)c1[O-])C(=O)Oc2cc(cc(O)c2[O-])C(=O)O[C@@H]3[C@H](COC(=O)c4cc(O)c([O-])c(OC(=O)c5cc(O)c([O-])c([O-])c5)c4)O[C@@H](OC(=O)c6cc(O)c([O-])c(OC(=O)c7cc(O)c([O-])c([O-])c7)c6)[C@H](OC(=O)c8cc(O)c([O-])c(OC(=O)c9cc(O)c([O-])c(O)c9)c8)[C@H]3OC(=O)c%10cc(O)c([O-])c(OC(=O)c%11cc(O)c([O-])c(O)c%11)c%10 |
| 473 | Oc1cc(cc(O)c1[O-])C(=O)Oc2cc(cc(O)c2[O-])C(=O)OC[C@@H]3O[C@@H](OC(=O)c4cc(O)c([O-])c(OC(=O)c5cc(O)c([O-])c(O)c5)c4)[C@H](OC(=O)c6cc(O)c([O-])c(OC(=O)c7cc(O)c([O-])c([O-])c7)c6)[C@@H](OC(=O)c8cc(O)c([O-])c(OC(=O)c9cc(O)c([O-])c(O)c9)c8)[C@@H]3OC(=O)c%10cc(O)c([O-])c(OC(=O)c%11cc(O)c([O-])c(O)c%11)c%10 |
| 474 | Oc1cc(cc(O)c1[O-])C(=O)Oc2cc(cc(O)c2[O-])C(=O)O[C@@H]3O[C@@H](COC(=O)c4cc(O)c([O-])c(OC(=O)c5cc(O)c([O-])c([O-])c5)c4)[C@@H](OC(=O)c6cc(O)c([O-])c(OC(=O)c7cc(O)c([O-])c(O)c7)c6)[C@H](OC(=O)c8cc(O)c([O-])c(OC(=O)c9cc(O)c([O-])c(O)c9)c8)[C@H]3OC(=O)c%10cc(O)c([O-])c(OC(=O)c%11cc(O)c([O-])c([O-])c%11)c%10 |
| 475 | Oc1cc(cc(O)c1[O-])C(=O)Oc2cc(cc(O)c2[O-])C(=O)OC[C@@H]3O[C@@H](OC(=O)c4cc(O)c([O-])c(OC(=O)c5cc(O)c([O-])c([O-])c5)c4)[C@H](OC(=O)c6cc(O)c([O-])c(OC(=O)c7cc(O)c([O-])c([O-])c7)c6)[C@@H](OC(=O)c8cc(O)c([O-])c(OC(=O)c9cc(O)c([O-])c(O)c9)c8)[C@@H]3OC(=O)c%10cc(O)c([O-])c(OC(=O)c%11cc(O)c([O-])c(O)c%11)c%10 |
| 476 | Oc1cc(cc(O)c1[O-])C(=O)Oc2cc(cc(O)c2[O-])C(=O)O[C@@H]3[C@H](COC(=O)c4cc(O)c([O-])c(OC(=O)c5cc(O)c([O-])c([O-])c5)c4)O[C@@H](OC(=O)c6cc(O)c([O-])c(OC(=O)c7cc(O)c([O-])c([O-])c7)c6)[C@H](OC(=O)c8cc(O)c([O-])c(OC(=O)c9cc(O)c([O-])c([O-])c9)c8)[C@H]3OC(=O)c%10cc(O)c([O-])c(OC(=O)c%11cc(O)c([O-])c(O)c%11)c%10 |
| 477 | Oc1cc(cc(O)c1[O-])C(=O)Oc2cc(cc(O)c2[O-])C(=O)OC[C@@H]3O[C@@H](OC(=O)c4cc(O)c([O-])c(OC(=O)c5cc(O)c([O-])c(O)c5)c4)[C@H](OC(=O)c6cc(O)c([O-])c(OC(=O)c7cc(O)c([O-])c(O)c7)c6)[C@@H](OC(=O)c8cc(O)c([O-])c(OC(=O)c9cc(O)c([O-])c(O)c9)c8)[C@@H]3OC(=O)c%10cc(O)c([O-])c(OC(=O)c%11cc(O)c([O-])c([O-])c%11)c%10 |
| 478 | Oc1cc(cc(O)c1[O-])C(=O)Oc2cc(cc(O)c2[O-])C(=O)O[C@@H]3O[C@@H](COC(=O)c4cc(O)c([O-])c(OC(=O)c5cc(O)c([O-])c([O-])c5)c4)[C@@H](OC(=O)c6cc(O)c([O-])c(OC(=O)c7cc(O)c([O-])c([O-])c7)c6)[C@H](OC(=O)c8cc(O)c([O-])c(OC(=O)c9cc(O)c([O-])c(O)c9)c8)[C@H]3OC(=O)c%10cc(O)c([O-])c(OC(=O)c%11cc(O)c([O-])c(O)c%11)c%10 |
| 479 | Oc1cc(cc(O)c1[O-])C(=O)Oc2cc(cc(O)c2[O-])C(=O)OC[C@@H]3O[C@@H](OC(=O)c4cc(O)c([O-])c(OC(=O)c5cc(O)c([O-])c([O-])c5)c4)[C@H](OC(=O)c6cc(O)c([O-])c(OC(=O)c7cc(O)c([O-])c(O)c7)c6)[C@@H](OC(=O)c8cc(O)c([O-])c(OC(=O)c9cc(O)c([O-])c(O)c9)c8)[C@@H]3OC(=O)c%10cc(O)c([O-])c(OC(=O)c%11cc(O)c([O-])c([O-])c%11)c%10 |
| 480 | Oc1cc(cc(O)c1[O-])C(=O)Oc2cc(cc(O)c2[O-])C(=O)O[C@H]3[C@H](OC(=O)c4cc(O)c([O-])c(OC(=O)c5cc(O)c([O-])c([O-])c5)c4)O[C@@H](COC(=O)c6cc(O)c([O-])c(OC(=O)c7cc(O)c([O-])c([O-])c7)c6)[C@@H](OC(=O)c8cc(O)c([O-])c(OC(=O)c9cc(O)c([O-])c([O-])c9)c8)[C@@H]3OC(=O)c%10cc(O)c([O-])c(OC(=O)c%11cc(O)c([O-])c(O)c%11)c%10 |
| 481 | Oc1cc(cc(O)c1[O-])C(=O)Oc2cc(cc(O)c2[O-])C(=O)OC[C@@H]3O[C@@H](OC(=O)c4cc(O)c([O-])c(OC(=O)c5cc(O)c([O-])c(O)c5)c4)[C@H](OC(=O)c6cc(O)c([O-])c(OC(=O)c7cc(O)c([O-])c([O-])c7)c6)[C@@H](OC(=O)c8cc(O)c([O-])c(OC(=O)c9cc(O)c([O-])c(O)c9)c8)[C@@H]3OC(=O)c%10cc(O)c([O-])c(OC(=O)c%11cc(O)c([O-])c([O-])c%11)c%10 |
| 482 | Oc1cc(cc(O)c1[O-])C(=O)Oc2cc(cc(O)c2[O-])C(=O)O[C@@H]3O[C@@H](COC(=O)c4cc(O)c([O-])c(OC(=O)c5cc(O)c([O-])c([O-])c5)c4)[C@@H](OC(=O)c6cc(O)c([O-])c(OC(=O)c7cc(O)c([O-])c([O-])c7)c6)[C@H](OC(=O)c8cc(O)c([O-])c(OC(=O)c9cc(O)c([O-])c(O)c9)c8)[C@H]3OC(=O)c%10cc(O)c([O-])c(OC(=O)c%11cc(O)c([O-])c([O-])c%11)c%10 |
| 483 | Oc1cc(cc(O)c1[O-])C(=O)Oc2cc(cc(O)c2[O-])C(=O)OC[C@@H]3O[C@@H](OC(=O)c4cc(O)c([O-])c(OC(=O)c5cc(O)c([O-])c([O-])c5)c4)[C@H](OC(=O)c6cc(O)c([O-])c(OC(=O)c7cc(O)c([O-])c([O-])c7)c6)[C@@H](OC(=O)c8cc(O)c([O-])c(OC(=O)c9cc(O)c([O-])c(O)c9)c8)[C@@H]3OC(=O)c%10cc(O)c([O-])c(OC(=O)c%11cc(O)c([O-])c([O-])c%11)c%10 |
| 484 | Oc1cc(cc(O)c1[O-])C(=O)Oc2cc(cc(O)c2[O-])C(=O)O[C@H]3[C@H](OC(=O)c4cc(O)c([O-])c(OC(=O)c5cc(O)c([O-])c([O-])c5)c4)[C@H](COC(=O)c6cc(O)c([O-])c(OC(=O)c7cc(O)c([O-])c([O-])c7)c6)O[C@@H](OC(=O)c8cc(O)c([O-])c(OC(=O)c9cc(O)c([O-])c([O-])c9)c8)[C@@H]3OC(=O)c%10cc(O)c([O-])c(OC(=O)c%11cc(O)c([O-])c([O-])c%11)c%10 |
| 485 | Oc1cc(cc(O)c1[O-])C(=O)Oc2cc(cc(O)c2[O-])C(=O)OC[C@@H]3O[C@@H](OC(=O)c4cc(O)c([O-])c(OC(=O)c5cc(O)c([O-])c(O)c5)c4)[C@H](OC(=O)c6cc(O)c([O-])c(OC(=O)c7cc(O)c([O-])c(O)c7)c6)[C@@H](OC(=O)c8cc(O)c([O-])c(OC(=O)c9cc(O)c([O-])c([O-])c9)c8)[C@@H]3OC(=O)c%10cc(O)c([O-])c(OC(=O)c%11cc(O)c([O-])c(O)c%11)c%10 |
| 486 | Oc1cc(cc(O)c1[O-])C(=O)Oc2cc(cc(O)c2[O-])C(=O)O[C@@H]3O[C@@H](COC(=O)c4cc(O)c([O-])c(OC(=O)c5cc(O)c([O-])c([O-])c5)c4)[C@@H](OC(=O)c6cc(O)c([O-])c(OC(=O)c7cc(O)c([O-])c(O)c7)c6)[C@H](OC(=O)c8cc(O)c([O-])c(OC(=O)c9cc(O)c([O-])c([O-])c9)c8)[C@H]3OC(=O)c%10cc(O)c([O-])c(OC(=O)c%11cc(O)c([O-])c(O)c%11)c%10 |
| 487 | Oc1cc(cc(O)c1[O-])C(=O)Oc2cc(cc(O)c2[O-])C(=O)OC[C@@H]3O[C@@H](OC(=O)c4cc(O)c([O-])c(OC(=O)c5cc(O)c([O-])c([O-])c5)c4)[C@H](OC(=O)c6cc(O)c([O-])c(OC(=O)c7cc(O)c([O-])c(O)c7)c6)[C@@H](OC(=O)c8cc(O)c([O-])c(OC(=O)c9cc(O)c([O-])c([O-])c9)c8)[C@@H]3OC(=O)c%10cc(O)c([O-])c(OC(=O)c%11cc(O)c([O-])c(O)c%11)c%10 |
| 488 | Oc1cc(cc(O)c1[O-])C(=O)Oc2cc(cc(O)c2[O-])C(=O)O[C@@H]3[C@H](COC(=O)c4cc(O)c([O-])c(OC(=O)c5cc(O)c([O-])c([O-])c5)c4)O[C@@H](OC(=O)c6cc(O)c([O-])c(OC(=O)c7cc(O)c([O-])c([O-])c7)c6)[C@H](OC(=O)c8cc(O)c([O-])c(OC(=O)c9cc(O)c([O-])c(O)c9)c8)[C@H]3OC(=O)c%10cc(O)c([O-])c(OC(=O)c%11cc(O)c([O-])c([O-])c%11)c%10 |
| 489 | Oc1cc(cc(O)c1[O-])C(=O)Oc2cc(cc(O)c2[O-])C(=O)OC[C@@H]3O[C@@H](OC(=O)c4cc(O)c([O-])c(OC(=O)c5cc(O)c([O-])c(O)c5)c4)[C@H](OC(=O)c6cc(O)c([O-])c(OC(=O)c7cc(O)c([O-])c([O-])c7)c6)[C@@H](OC(=O)c8cc(O)c([O-])c(OC(=O)c9cc(O)c([O-])c([O-])c9)c8)[C@@H]3OC(=O)c%10cc(O)c([O-])c(OC(=O)c%11cc(O)c([O-])c(O)c%11)c%10 |
| 490 | Oc1cc(cc(O)c1[O-])C(=O)Oc2cc(cc(O)c2[O-])C(=O)O[C@@H]3O[C@@H](COC(=O)c4cc(O)c([O-])c(OC(=O)c5cc(O)c([O-])c([O-])c5)c4)[C@@H](OC(=O)c6cc(O)c([O-])c(OC(=O)c7cc(O)c([O-])c(O)c7)c6)[C@H](OC(=O)c8cc(O)c([O-])c(OC(=O)c9cc(O)c([O-])c([O-])c9)c8)[C@H]3OC(=O)c%10cc(O)c([O-])c(OC(=O)c%11cc(O)c([O-])c([O-])c%11)c%10 |
| 491 | Oc1cc(cc(O)c1[O-])C(=O)Oc2cc(cc(O)c2[O-])C(=O)OC[C@@H]3O[C@@H](OC(=O)c4cc(O)c([O-])c(OC(=O)c5cc(O)c([O-])c([O-])c5)c4)[C@H](OC(=O)c6cc(O)c([O-])c(OC(=O)c7cc(O)c([O-])c([O-])c7)c6)[C@@H](OC(=O)c8cc(O)c([O-])c(OC(=O)c9cc(O)c([O-])c([O-])c9)c8)[C@@H]3OC(=O)c%10cc(O)c([O-])c(OC(=O)c%11cc(O)c([O-])c(O)c%11)c%10 |
| 492 | Oc1cc(cc(O)c1[O-])C(=O)Oc2cc(cc(O)c2[O-])C(=O)O[C@@H]3[C@H](COC(=O)c4cc(O)c([O-])c(OC(=O)c5cc(O)c([O-])c([O-])c5)c4)O[C@@H](OC(=O)c6cc(O)c([O-])c(OC(=O)c7cc(O)c([O-])c([O-])c7)c6)[C@H](OC(=O)c8cc(O)c([O-])c(OC(=O)c9cc(O)c([O-])c([O-])c9)c8)[C@H]3OC(=O)c%10cc(O)c([O-])c(OC(=O)c%11cc(O)c([O-])c([O-])c%11)c%10 |
| 493 | Oc1cc(cc(O)c1[O-])C(=O)Oc2cc(cc(O)c2[O-])C(=O)OC[C@@H]3O[C@@H](OC(=O)c4cc(O)c([O-])c(OC(=O)c5cc(O)c([O-])c(O)c5)c4)[C@H](OC(=O)c6cc(O)c([O-])c(OC(=O)c7cc(O)c([O-])c(O)c7)c6)[C@@H](OC(=O)c8cc(O)c([O-])c(OC(=O)c9cc(O)c([O-])c([O-])c9)c8)[C@@H]3OC(=O)c%10cc(O)c([O-])c(OC(=O)c%11cc(O)c([O-])c([O-])c%11)c%10 |
| 494 | Oc1cc(cc(O)c1[O-])C(=O)Oc2cc(cc(O)c2[O-])C(=O)O[C@@H]3O[C@@H](COC(=O)c4cc(O)c([O-])c(OC(=O)c5cc(O)c([O-])c([O-])c5)c4)[C@@H](OC(=O)c6cc(O)c([O-])c(OC(=O)c7cc(O)c([O-])c([O-])c7)c6)[C@H](OC(=O)c8cc(O)c([O-])c(OC(=O)c9cc(O)c([O-])c([O-])c9)c8)[C@H]3OC(=O)c%10cc(O)c([O-])c(OC(=O)c%11cc(O)c([O-])c(O)c%11)c%10 |
| 495 | Oc1cc(cc(O)c1[O-])C(=O)Oc2cc(cc(O)c2[O-])C(=O)OC[C@@H]3O[C@@H](OC(=O)c4cc(O)c([O-])c(OC(=O)c5cc(O)c([O-])c([O-])c5)c4)[C@H](OC(=O)c6cc(O)c([O-])c(OC(=O)c7cc(O)c([O-])c(O)c7)c6)[C@@H](OC(=O)c8cc(O)c([O-])c(OC(=O)c9cc(O)c([O-])c([O-])c9)c8)[C@@H]3OC(=O)c%10cc(O)c([O-])c(OC(=O)c%11cc(O)c([O-])c([O-])c%11)c%10 |
| 496 | Oc1cc(cc(O)c1[O-])C(=O)Oc2cc(cc(O)c2[O-])C(=O)O[C@H]3[C@H](OC(=O)c4cc(O)c([O-])c(OC(=O)c5cc(O)c([O-])c([O-])c5)c4)O[C@@H](COC(=O)c6cc(O)c([O-])c(OC(=O)c7cc(O)c([O-])c([O-])c7)c6)[C@@H](OC(=O)c8cc(O)c([O-])c(OC(=O)c9cc(O)c([O-])c([O-])c9)c8)[C@@H]3OC(=O)c%10cc(O)c([O-])c(OC(=O)c%11cc(O)c([O-])c([O-])c%11)c%10 |
| 497 | Oc1cc(cc(O)c1[O-])C(=O)Oc2cc(cc(O)c2[O-])C(=O)OC[C@@H]3O[C@@H](OC(=O)c4cc(O)c([O-])c(OC(=O)c5cc(O)c([O-])c(O)c5)c4)[C@H](OC(=O)c6cc(O)c([O-])c(OC(=O)c7cc(O)c([O-])c([O-])c7)c6)[C@@H](OC(=O)c8cc(O)c([O-])c(OC(=O)c9cc(O)c([O-])c([O-])c9)c8)[C@@H]3OC(=O)c%10cc(O)c([O-])c(OC(=O)c%11cc(O)c([O-])c([O-])c%11)c%10 |
| 498 | Oc1cc(cc(O)c1[O-])C(=O)Oc2cc(cc(O)c2[O-])C(=O)O[C@@H]3O[C@@H](COC(=O)c4cc(O)c([O-])c(OC(=O)c5cc(O)c([O-])c([O-])c5)c4)[C@@H](OC(=O)c6cc(O)c([O-])c(OC(=O)c7cc(O)c([O-])c([O-])c7)c6)[C@H](OC(=O)c8cc(O)c([O-])c(OC(=O)c9cc(O)c([O-])c([O-])c9)c8)[C@H]3OC(=O)c%10cc(O)c([O-])c(OC(=O)c%11cc(O)c([O-])c([O-])c%11)c%10 |
| 499 | Oc1cc(cc(O)c1[O-])C(=O)Oc2cc(cc(O)c2[O-])C(=O)OC[C@@H]3O[C@@H](OC(=O)c4cc(O)c([O-])c(OC(=O)c5cc(O)c([O-])c([O-])c5)c4)[C@H](OC(=O)c6cc(O)c([O-])c(OC(=O)c7cc(O)c([O-])c([O-])c7)c6)[C@@H](OC(=O)c8cc(O)c([O-])c(OC(=O)c9cc(O)c([O-])c([O-])c9)c8)[C@@H]3OC(=O)c%10cc(O)c([O-])c(OC(=O)c%11cc(O)c([O-])c([O-])c%11)c%10 |
| 500 | Oc1cc(cc([O-])c1[O-])C(=O)Oc2cc(cc(O)c2[O-])C(=O)OC[C@@H]3O[C@@H](OC(=O)c4cc(O)c([O-])c(OC(=O)c5cc(O)c([O-])c([O-])c5)c4)[C@H](OC(=O)c6cc(O)c([O-])c(OC(=O)c7cc(O)c([O-])c([O-])c7)c6)[C@@H](OC(=O)c8cc(O)c([O-])c(OC(=O)c9cc(O)c([O-])c([O-])c9)c8)[C@@H]3OC(=O)c%10cc(O)c([O-])c(OC(=O)c%11cc(O)c([O-])c([O-])c%11)c%10 |
| 501 | OC[C@H]1O[C@H](OC2=C(Oc3cc(O[C@@H]4O[C@H](CO)[C@@H](O)[C@H](O)[C@H]4O)cc(O)c3C2=O)c5ccc(O)cc5)[C@H](O)[C@@H](O)[C@@H]1O |
| 502 | OC[C@H]1O[C@H](OC2=C(Oc3cc(O[C@@H]4O[C@H](CO)[C@@H](O)[C@H](O)[C@H]4O)cc(O)c3C2=O)c5ccc([O-])cc5)[C@H](O)[C@@H](O)[C@@H]1O |
| 503 | C[C@H]1[C@H]2C[C@H](O)[C@@](C)(O)C[C@H]2OC1=O |
| 504 | C[C@@]12CC(=O)[C@H]3C[C@]1(O)O[C@@H](O2)[C@@H]3COC(=O)c4ccccc4 |
| 505 | CCCCCCCC[C@H](CO)CCCCCC |
| 506 | C[C@]1(O)C[C@H]2OC(=O)[C@H](COC(=O)c3ccccc3)[C@H]2CC1=O |
| 507 | C[C@@]12C[C@@]3(O)O[C@@H](O1)[C@]4(COC(=O)c5ccccc5)[C@H]3C[C@]24O[C@@H]6O[C@H](CO)[C@@H](O)[C@H](O)[C@H]6O |
| 508 | C[C@@]12C[C@@]3(O)O[C@@H](O1)[C@]4(COC(=O)c5ccccc5)[C@H]3C[C@]24O |
| 509 | O[C@H]([C@H](O)C(=O)c1cc(O)c(O)c(O)c1)[C@@H](O)[C@@](O)(C(=O)c2cc(O)c(O)c(O)c2)[C@@](O)(C(=O)C(=O)c3cc(O)c(O)c(O)c3)C(=O)c4cc(O)c(O)c(O)c4 |
| 510 | C[C@@]12C[C@@H](O)[C@H]3C[C@]1(O[C@@H]4O[C@H](CO)[C@@H](O)[C@H](O)[C@H]4O)[C@@]3(COC(=O)c5ccccc5)C(=O)O2 |
| 511 | C[C@@]12C[C@@H](O)[C@H]3C[C@]1(O)[C@@]3(COC(=O)c4ccccc4)C(=O)O2 |
| 512 | CC[C@H](CC[C@@H](C)[C@H]1CC[C@H]2[C@@H]3CC=C4C[C@@H](CC[C@]4(C)[C@H]3CC[C@]12C)O[C@@H]5O[C@H](CO)[C@@H](O)[C@H](O)[C@H]5O)C(C)C |
| 513 | [O-]C(=O)CCCCl |
| 514 | C[C@@]12C[C@@]3(O)O[C@@H](O1)[C@]4(COC(=O)c5ccccc5)[C@H]3C[C@]24O[C@H]6O[C@H](COC(=O)c7cc(O)c(O)c(O)c7)[C@@H](O)[C@H](O)[C@H]6O |
| 515 | C[C@@]12C[C@@]3(O)O[C@@H](O1)[C@]4(COC(=O)c5ccccc5)[C@H]3C[C@]24O[C@H]6O[C@H](COC(=O)c7cc(O)c([O-])c(O)c7)[C@@H](O)[C@H](O)[C@H]6O |
| 516 | C[C@@]12C[C@@]3(O)O[C@@H](O1)[C@]4(COC(=O)c5ccc(O)cc5)[C@H]3C[C@]24O[C@H]6O[C@H](CO)[C@@H](O)[C@H](O)[C@H]6O |
| 517 | C[C@@]12C[C@@]3(O)O[C@@H](O1)[C@]4(COC(=O)c5ccc([O-])cc5)[C@H]3C[C@]24O[C@H]6O[C@H](CO)[C@@H](O)[C@H](O)[C@H]6O |
| 518 | CC\C=C/CCCCO |
| 519 | CCC(=O)CC=C |
| 520 | CC(=O)[O-] |
| 521 | C[C@@]12C[C@@]3(O)O[C@@H](O1)[C@]4(COC(=O)c5ccc(O)cc5)[C@H]3C[C@]24O[C@@H]6O[C@H](CO)[C@@H](O)[C@H](O)[C@H]6O |
| 522 | C[C@@]12C[C@@]3(O)O[C@@H](O1)[C@]4(COC(=O)c5ccc([O-])cc5)[C@H]3C[C@]24O[C@@H]6O[C@H](CO)[C@@H](O)[C@H](O)[C@H]6O |
| 523 | CCCCCCCCCC(C)CCCCCCCCC |
| 524 | CCOC(=O)C(C)C |
| 525 | Oc1cccc(O)c1O |
| 526 | CC1=C2C[C@H]3C(=C)CCC[C@]3(C)C=C2OC1=O |
| 527 | CC1=C2C[C@H]3C(=C)CCC[C@]3(C)C[C@]2(O)OC1=O |
| 528 | OCC[C@@H](O)\C=C\C=C\C#CC#C\C=C\CO |
| 529 | Cc1coc2C[C@@]3(C)CCCC(=C)[C@@H]3Cc12 |
| 530 | CCO[C@]12C[C@@]3(C)CCCC(=C)[C@]3(C)CC1=C(C)C(=O)O2 |
| 531 | CC(=C1C[C@H]2C(=C)CCC[C@]2(C)CC1=O)C |
| 532 | CC(=C1CC[C@@]2(C)CCC[C@@](C)(O)[C@@H]2C1)C |
| 533 | CC[C@@H](C)C(=O)O[C@@H](CCOC(=O)C)\C=C\C=C\C#CC#C\C=C\CO |
| 534 | CC[C@@H](C)C(=O)O[C@@H](CCOC(=O)C)\C=C\C=C/C#CC#C\C=C\CO |
| 535 | CC[C@@H](C)C(=O)OCC[C@@H](O)\C=C\C=C\C#CC#C\C=C\CO |
| 536 | CC[C@@H](C)C(=O)OCC[C@@H](O)\C=C\C=C/C#CC#C\C=C\CO |
| 537 | CC1(C)[C@@H]2CC[C@@H](C2)C1=C |
| 538 | CC(=CC(=O)O[C@H](CCO)\C=C\C=C\C#CC#C\C=C/CO)C |
| 539 | CC(=CC(=O)O[C@@H](CCOC(=O)C)\C=C\C=C\C#CC#C\C=C/CO)C |
| 540 | CC(=CC(=O)O[C@H](CCOC(=O)C)\C=C\C=C/C#CC#C\C=C\CO)C |
| 541 | C\C\1=C/CC(C)(C)\C=C\C\C(=C\CC1)\C |
| 542 | CC1=CC[C@@H]2[C@@H]3[C@H]1[C@@]2(C)CCCC3(C)C |
| 543 | CC[C@H](\C=C\[C@@H](C)[C@H]1CC[C@H]2[C@@H]3CC[C@H]4C[C@@H](O)CC[C@]4(C)[C@H]3CC[C@]12C)C(C)C |
| 544 | C[C@@H]1CC[C@]2(C)CC[C@]3(C)C(=CC[C@@H]4[C@@]5(C)CC[C@H](O)C(C)(C)[C@H]5CC[C@@]34C)[C@@H]2[C@H]1C |
| 545 | C\C\1=C/CC(C)(C)\C=C\CC(=C)CCC1 |
| 546 | CC(=C)[C@@H](O)c1ccccc1 |
| 547 | C[C@H](CCC=C(C)C)[C@@H]1CCC(=C)C=C1 |
| 548 | CC(C)(O)[C@@H]1CC[C@@]2(C)CCCC(=C)[C@@H]2C1 |
| 549 | CCC[C@@H](CC[C@@H](C)[C@H]1CC[C@H]2[C@@H]3CC=C4C[C@@H](O)CC[C@]4(C)[C@H]3CC[C@]12C)C(C)C |
| 550 | CC(=C)[C@@H]1C[C@@H](CC[C@@]1(C)C=C)C(C)(C)O |
| 551 | CC(=C)[C@@H]1CC[C@@]2(C)CCCC(=C)[C@@H]2C1 |
| 552 | C\C\1=C/CCC(=C)[C@H]2CC(C)(C)[C@@H]2CC1 |
| 553 | CC(=C1CC[C@](C)(C=C)[C@H](C1)C(=C)C)C |
| 554 | c1ccc2nc3ccccc3cc2c1 |
| 555 | C[C@H]1CC[C@H](C[C@H]1O)C(=C)C |
| 556 | [NH3+][C@@H](Cc1ccccc1)C(=O)[O-] |
| 557 | C[C@H]([NH3+])C(=O)[O-] |
| 558 | CC1=C2C[C@H]3C(=C)CCC[C@]3(C)C[C@@H]2OC1=O |
| 559 | Cc1coc2C[C@]3(C)CCCC(=C)[C@@H]3Cc12 |
| 560 | CC(=C1CC[C@@]2(C)CCC[C@@](C)(O)[C@H]2C1)C |
| 561 | C\C\1=C/Cc2c(C)coc2C\C(=C/CC1)\C |
| 562 | CC(=O)O[C@H]1CC[C@@]2(C)Cc3occ(C)c3C[C@H]2C1=C |
| 563 | [NH3+]CC(=O)[O-] |
| 564 | OC[C@@H](O)[C@@H](O)[C@H](O)[C@H](O)C=O |
| 565 | [NH3+][C@@H](CCC(=O)[O-])C(=O)[O-] |
| 566 | OC[C@@H](O)[C@@H](O)[C@H](O)C(=O)CO |
| 567 | NC(=[NH2+])NCCC[C@H]([NH3+])C(=O)[O-] |
| 568 | [NH3+]CCCC[C@H]([NH3+])C(=O)[O-] |
| 569 | [NH3+][C@@H](Cc1ccc(O)cc1)C(=O)[O-] |
| 570 | CC(C)COC(=O)c1ccccc1C(=O)OCC(C)C |
| 571 | C[C@H]1CCC=C(C)[C@]12CC[C@H](C2)C(C)(C)O |
| 572 | OC[C@H]1O[C@H]([C@H](O)[C@@H]1O)N2C=CC(=O)NC2=O |
| 573 | CC(=C1C[C@@H]2C(=C)CCC[C@]2(C)CC1=O)C |
| 574 | [O-]C(=O)[C@@H]1CCC[NH2+]1 |
| 575 | CC1=C2C[C@@H]3C(=C)CCC[C@]3(C)C[C@]2(OC1=O)[C@@]45C[C@]6(C)CCCC(=C)[C@H]6CC4=C(C)C(=O)O5 |
| 576 | CC1=C2C[C@H]3C(=C)CCC[C@@]3(C)C[C@]2(OC1=O)[C@@]45C[C@@]6(C)CCCC(=C)[C@@H]6CC4=C(C)C(=O)O5 |
| 577 | [NH3+][C@H](CO)C(=O)[O-] |
| 578 | [NH3+][C@@H](CC(=O)[O-])C(=O)[O-] |
| 579 | C[C@H]1CC[C@@H]2[C@H]1[C@H]3[C@@H](CCC2=C)C3(C)C |
| 580 | CC(C)[C@H]([NH3+])C(=O)[O-] |
| 581 | CC[C@H](C)[C@H]([NH3+])C(=O)[O-] |
| 582 | CCOC(=O)CC(=O)C(C)(C)C |
| 583 | [NH3+][C@@H](Cc1c[nH]cn1)C(=O)[O-] |
| 584 | CCO[C@]12C[C@@]3(C)CCCC(=C)[C@@H]3CC1=C(C)C(=O)O2 |
| 585 | CC1(C)[C@@H]2CC[C@@]1(C)[C@H](O)C2 |
| 586 | C\C\1=C\CCC(=C)[C@H]2CC(C)(C)[C@H]2CC1 |
| 587 | CCCCC\C=C\C\C=C\CCCCCCCC=O |
| 588 | CC1(C)O[C@H]1COc2c3OC(=O)C=Cc3cc4ccoc24 |
| 589 | CC1(C)[C@@H]2CC[C@@]1(C)C(=O)C2 |
| 590 | CC(=CCCC(=C)C=C)C |
| 591 | CC(C)C1=CCC(=CC1)C |
| 592 | COc1ccc(CC=C)cc1OC |
| 593 | C[C@@H]1CC[C@@H]2[C@@H]1[C@H]3[C@@H](CCC2=C)C3(C)C |
| 594 | CC(=C)[C@H]1CCC(=CC1)C |
| 595 | CC(=CCC\C(=C\CC\C(=C\CO)\C)\C)C |
| 596 | COc1cc(CC=C)ccc1O |
| 597 | CC(C)c1ccc(C)c(O)c1 |
| 598 | COc1cc(\C=C\C(=O)[O-])ccc1O |
| 599 | O=C1Oc2ccccc2C=C1 |
| 600 | CC[C@H](\C=C\[C@@H](C)[C@H]1CC[C@H]2[C@@H]3CC=C4C[C@@H](O)CC[C@]4(C)[C@H]3CC[C@]12C)C(C)C |
| 601 | CC1(C)C[C@H]2[C@H]1CC[C@@]3(C)O[C@@H]3CCC2=C |
| 602 | COc1ccc(\C=C\C)cc1 |
| 603 | CC(=CCC\C(=C\CCC(=C)C=C)\C)C |
| 604 | CC1=CC[C@@H]2C[C@H]1C2(C)C |
| 605 | COc1ccc2C=CC(=O)Oc2c1CC=C(C)C |
| 606 | CC(C)[C@@H]1CCC(=C2CCC(=C[C@@H]12)C)C |
| 607 | CC(C)[C@H]1CC=C(C)C=C1 |
| 608 | CCCCC[C@H](O)C=C |
| 609 | CCCCCC\C=C\C=O |
| 610 | COC(=O)c1ccccc1O |
| 611 | CCCCC\C=C\C\C=C\CCCCCCCC(=O)[O-] |
| 612 | CCCCCCCCCCCCCCC |
| 613 | CCCCCCCCCCCCCCCC |
| 614 | CCCCOC(=O)C |
| 615 | CCCCCCCCCCCCCCCC(=O)OC |
| 616 | CC(=C)[C@@H]1CC[C@@](C)(C=C)[C@@H](C1)C(=C)C |
| 617 | CC(=C1C\C=C(/C)\CC\C=C(/C)\CC1=O)C |
| 618 | CC(C)[C@@]1(O)CCC(=CC1)C |
| 619 | CC(=CCC\C(=C\C\C=C(/C)\C=C)\C)C |
| 620 | C[C@@H]1CCC=C2CC[C@@H]3[C@@H](C3(C)C)[C@@]12C |
| 621 | CC(C)[C@@H]1CC[C@@](C)(O)[C@@H]2CCC(=C[C@@H]12)C |
| 622 | CC(C)CCC[C@@H](C)[C@H]1CC[C@H]2[C@@H]3CC=C4C[C@@H](O)CC[C@]4(C)[C@H]3CC[C@]12C |
| 623 | CC(=CC\C=C(\C)/[C@H]1CCC(=CC1)C)C |
| 624 | CC(=CC\C=C(\C)/C=C)C |
| 625 | CC1(C)[C@H]2CC=C(CO)[C@@H]1C2 |
| 626 | CC(C)[C@@H]1CC=C(C)[C@H]2CCC(=C[C@@H]12)C |
| 627 | C[C@@H]1CC[C@H]2[C@@H]1[C@H]3[C@@H](CCC2=C)C3(C)C |
| 628 | CC(=CCC[C@@]1(C)[C@@H]2CC=C(C)[C@H]1C2)C |
| 629 | CC(C)[C@H]1CCC(=CC1)C=O |
| 630 | CCCCCCCCCCCCCCO |
| 631 | CCCCCCCCCCCO |
| 632 | CCCCCCCC\C=C\CCCCCCCC(=O)[O-] |
| 633 | C[C@@H]1CC[C@@H]2[C@@H]1[C@H]3[C@@H](CC[C@@]2(C)O)C3(C)C |
| 634 | CCCCCCCCCCCCCCCCCCCCCCCCCC |
| 635 | CCCCCCCCCCCCCCCCCCCC(=O)OCC |
| 636 | CCCCCCCCCCCCCCCCC(=O)OCC |
| 637 | CCCCC\C=C/C\C=C/CCCCCCCC(=O)OCC |
| 638 | CCCCCCCCCCCCCCCCCC(=O)OCC |
| 639 | CCCCCCCCCCCCCCCCC(=O)[O-] |
| 640 | C\C\1=C\CCC(=C)[C@H]2CC(C)(C)[C@@H]2CC1 |
| 641 | CCCCCCCCCCCCCCCCCC=C |
| 642 | CC(C)C1=C[C@H](C(=C)C)[C@](C)(CC1)C=C |
| 643 | CCCCCCCCCCCCC=C |
| 644 | C\C=C(/C)\C(=O)[O-] |
| 645 | CCCCC\C=C/C\C=C/CCCCCCCC(=O)OC |
| 646 | CCCCCCCCCCCCCCCCCCCCCCCC |
| 647 | CCCC[C@@H](CC)COC(=O)c1ccccc1C(=O)OC[C@H](CC)CCCC |
| 648 | COc1cc(C=C)ccc1O |
| 649 | CC(=CCc1c2OC(=O)C=Cc2c(O)c3ccoc13)C |
| 650 | CCCCCCC\C=C/[C@H](O)C#CC#C[C@H](O)C=C |
| 651 | CC(=CCOc1c2OC(=O)C=Cc2cc3ccoc13)C |
| 652 | CC(=CCOc1c2C=CC(=O)Oc2cc3occc13)C |
| 653 | COc1c2C=CC(=O)Oc2cc3occc13 |
| 654 | CCCCCCC\C=C/CC#CC#C[C@H](O)C=C |
| 655 | COc1c2OC(=O)C=Cc2cc3ccoc13 |
| 656 | Oc1c2OC(=O)C=Cc2cc3ccoc13 |
| 657 | COc1c2OC(=O)C=Cc2c(OCC=C(C)C)c3ccoc13 |
| 658 | Cc1ccc(cc1)[C@]2(C)CCCC2(C)C |
| 659 | CC1=CC[C@]2(C)CCCC(C)(C)[C@@]23C[C@@H]13 |
| 660 | CCC\C=C\1/OC(=O)c2ccccc12 |
| 661 | CC1(C)[C@@H]2CCC(=C)[C@@H]3CC[C@](C)(O)[C@H]3[C@H]12 |
| 662 | CCCCCCCCCCCCCCCCCCCCCCCCCCC |
| 663 | CCCCCCCCCCCCCCCCCCCCCCCCC |
| 664 | CC1(C)[C@H]2CC[C@H](C2)C1=C |
| 665 | CC(=CCC\C(=C/CC[C@](C)(O)C=C)\C)C |
| 666 | COc1c2C=CC(=O)Oc2c(O)c3occc13 |
| 667 | [O-]c1ccc2C=CC(=O)Oc2c1 |
| 668 | COc1c2C=CC(=O)Oc2c(OCC=C(C)C)c3occc13 |
| 669 | CC(C)CC(=O)[O-] |
| 670 | OC1CCCCCCCCCCC1 |
| 671 | CC\C=C(/C)\C=C(/C)\C=O |
| 672 | CC(=CCCC(=C)[C@H]1CCC(=CC1)C)C |
| 673 | CCCCCCCCCCCCCCCCCCOCCO |
| 674 | CCCCCC\C=C\C\C=C\CCCCCCC(=O)OC |
| 675 | CCCCCCCCCCCCCC[C@H]1CO1 |
| 676 | CCCCCC#CCC#CCC#CCCCC(=O)OC |
| 677 | CCCCCCCO |
| 678 | CCCCCCCCCCO |
| 679 | CC(C)[C@@H]1CC[C@](C)(O)[C@@H]2CCC(=C[C@@H]12)C |
| 680 | CC(C)[C@@H]1CC[C@](C)(O)[C@H]2CCC(=C[C@@H]12)C |
| 681 | C[C@@H]1CCCC2=CC(=O)[C@@H]3[C@@H](C3(C)C)[C@@]12C |
| 682 | COc1c2OC(=O)C=Cc2c(OC)c3ccoc13 |
| 683 | CC(=CCc1c2C=CC(=O)Oc2c(O)c3occc13)C |
| 684 | O=C1Oc2c(C=C1)ccc3occc23 |
| 685 | COc1ccc2C=CC(=O)Oc2c1C[C@H](O)C(=C)C |
| 686 | CCCCC\C=C/C\C=C/CCCCCCCC(=O)OC(CO)CO |
| 687 | Cc1oc(C=O)cc1 |
| 688 | CCCCCCCCCCCCOC(=O)C |
| 689 | CCCCCCCCCCCCCO |
| 690 | CC(=O)OCC1=CC[C@@H]2C[C@H]1C2(C)C |
| 691 | CC(C)C1=CCC(=C)CC1 |
| 692 | CCCCCCCCCCCCCCCCCCO |
| 693 | CCCCCCCCCCCCCCCCO |
| 694 | CC(C)(O)[C@H]1Cc2cc3C=CC(=O)Oc3cc2O1 |
| 695 | CCCCCCCCCCCCCCCO |
| 696 | CCCC1CCCC1 |
| 697 | CCCCCCCCCCCCCCCCCO |
| 698 | CCCC[C@@H](CC)COC(=O)c1ccccc1C(=O)[O-] |
| 699 | CCCCCCCCCCCCCCCCCCC=C |
| 700 | CCCC[C@@H](C)CCC[C@H](C)CCCC(C)C |
| 701 | CCCCCCCCCCCCCCC=O |
| 702 | OCCOC(=O)C=C |
| 703 | CCCCCCCCCCCCCCCCOC(=O)CC(C)C |
| 704 | Oc1c2C=CC(=O)Oc2cc3occc13 |
| 705 | COc1c2C=CC(=O)Oc2c(OC[C@@H](O)C(C)(C)O)c3occc13 |
| 706 | C1CCCCCCCCCCC1 |
| 707 | CCCCCCCCCC(=O)OCC |
| 708 | COc1c2C=CC(=O)Oc2c(OC[C@H](O)C(=C)C)c3occc13 |
| 709 | CC1(C)O[C@H]1COC2=CCOc3cc4OC(=O)Cc4cc23 |
| 710 | CC1(C)O[C@@H]1COC2=CC(=O)Oc3cc4occc4cc23 |
| 711 | CC(=C)[C@H](O)COc1c2C=CC(=O)Oc2cc3occc13 |
| 712 | CC1(C)O[C@@H]1COc2c3OC(=O)C=Cc3cc4ccoc24 |
| 713 | CC(C)[C@@]12C[C@@H](O)C(=C)[C@@H]1C2 |
| 714 | COc1cc2OC(=O)C=Cc2cc1CC=C(C)C |
| 715 | CCCCCCCCCCCCCCCCCCCCCCCCO |
| 716 | CCCCCCCCCCCCC=O |
| 717 | CCCCCCCCCCC(=O)OCC |
| 718 | CCOC(=O)\C=C\C |
| 719 | COc1c2C=CC(=O)Oc2c(OC[C@H]3OC3(C)C)c4occc14 |
| 720 | CC(=CCOc1c2C=CC(=O)Oc2c(O)c3occc13)C |
| 721 | CCCCCCCC\C=C/CCCCCCCC(=O)OC[C@@H](C)O |
| 722 | C[C@H]1OC(=O)Cn2c(C=O)ccc2[C@H](O)[C@H]1C |
| 723 | CCCCCCCCCCCCCCCCCCOP(=O)([O-])OCCCCCCCCCCCCCCCCCC |
| 724 | c1cc2ccc3cccc4ccc(c1)c2c34 |
| 725 | CC(C)(O)[C@@H](O)COc1c2C=CC(=O)Oc2cc3occc13 |
| 726 | CC1(C)O[C@@H]1COc2c3C=CC(=O)Oc3c(CO[C@H]4OC4(C)C)c5occc25 |
| 727 | CCCCC[C@H](CC)OC(=O)C |
| 728 | CC(=CC\C=C(/C)\CCO)C |
| 729 | CCCCCCCCCCCCCCCC(=O)OCCCCCCCCCCCC |
| 730 | CCCCCCCCCCCCC\C=C\C(=O)[O-] |
| 731 | CCCCCCCCCCCCCC=O |
| 732 | COc1c(O)cccc1CC=C |
| 733 | CC1=C2[C@H]3[C@@H](CC[C@]2(C)CCC1)C3(C)C |
| 734 | OC[C@@]1(O)CO[C@@H](OC[C@H]2O[C@@H](OCCc3ccc(O)cc3)[C@H](O)[C@@H](O)[C@@H]2O)[C@@H]1O |
| 735 | CCCCC\C=C\C\C=C\CCCCCCCC(=O)OCC |
| 736 | CCCCCCCCCCCCCCCCCCCCCOC=O |
| 737 | C1CCCCCCCCCCCCC1 |
| 738 | CC(C)[C@@H]1CC[C@@H](C)C[C@H]1O |
| 739 | CCCCC\C=C\C\C=C\CCCCCCCCCC(=O)OC |
| 740 | C[C@]12CC[C@H](C1)C(C)(C)[C@@H]2O |
| 741 | CCc1cnc(C)cn1 |
| 742 | Cc1cnc(C)cn1 |
| 743 | CCCCCCOC=O |
| 744 | Cc1cnc(C)c(C)n1 |
| 745 | CCCCCCCCCCC=C |
| 746 | CC1=C[C@@H]2[C@H](CC1)C2(C)C |
| 747 | C=CCCCCCCCCCC=C |
| 748 | CCCCCCCCCCCCCCCCOC(=O)C |
| 749 | CCCCCCCCCCCCCC[C@@H](C)O |
| 750 | CC(C)[C@@H]1CC[C@@H](C)[C@]2(O)CCC(=C[C@@H]12)C |
| 751 | CCCCCCCCCCCCCCOC(=O)C |
| 752 | CC(=CCCC=C(C)C)C |
| 753 | CCCCCCCC\C=C\CCCCCCCCCCCCCC(=O)OC |
| 754 | CCCCCCCCCC\C=C\CCCCCCCC |
| 755 | C[N@H+]1[C@H]2C[C@H]3C[C@@H]1[C@H](O3)[C@H]2O |
| 756 | CN1[C@H]2C[C@H]3C[C@@H]1[C@H](O3)[C@H]2O |
| 757 | CCOC(=O)CCC(=O)OCC |
| 758 | CCCC\C=C/CCCCCCCCCCCC=O |
| 759 | C[C@H]1CCC=C2CC[C@H](C[C@]12C)C(=C)C |
| 760 | C[C@@H]1CC[C@H]2C(C)(C)[C@H]3C[C@@]12CCC3=C |
| 761 | CC1=C2C[C@@H](CC[C@@]2(C)CCC1)C(C)(C)O |
| 762 | C1CCCCCCCCCCCCCCC1 |
| 763 | CC(=O)OC[C@@H](COC(=O)\C=C/c1ccc(O)cc1)O[C@@H]2O[C@H](CO)[C@@H](O)[C@H](O)[C@H]2O |
| 764 | CC(=O)OC[C@H](O)COC(=O)\C=C\c1ccc(O)cc1 |
| 765 | OC[C@@H](COC(=O)\C=C/c1ccc(O)cc1)O[C@@H]2O[C@H](CO)[C@@H](O)[C@H](O)[C@H]2O |
| 766 | CC(=O)OC[C@@H](COC(=O)\C=C/c1ccc(O)c(O)c1)O[C@@H]2O[C@H](CO)[C@@H](O)[C@H](O)[C@H]2O |
| 767 | CC(=O)OC[C@H](O)COC(=O)\C=C\c1ccc(O)c(O)c1 |
| 768 | COc1cc(\C=C\C(=O)OC[C@@H](O)CO[C@@H]2O[C@H](CO)[C@@H](O)[C@H](O)[C@H]2O)ccc1O |
| 769 | COc1cc(\C=C\C(=O)OC[C@@H](O)CO)ccc1O |
| 770 | CCCCCCCCCCCCCCCCCCCCCCCCCCCCCCCCCCO |
| 771 | CC(C)[C@@H]1CC[C@@](C)(O)C=C1 |
| 772 | CC\C=C\CCOC(=O)C |
| 773 | CCCCCCCCCC=O |
| 774 | CC1(C)[C@H]2CCC(=C)[C@@H]1C2 |
| 775 | CC(=CCC\C(=C\COC(=O)C)\C)C |
| 776 | CC(=C1CCC(=CC1)C)C |
| 777 | Cc1cc(O)c2C(=O)c3c([O-])cc(O)cc3C(=O)c2c1 |
| 778 | Cc1cc([O-])c2C(=O)c3c([O-])cc(O)cc3C(=O)c2c1 |
| 779 | CC(=CCC\C(=C/COC(=O)C)\C)C |
| 780 | COc1cc(C)ccc1C(C)C |
| 781 | CC[C@@H](\C=C\[C@H](C)[C@H]1CC[C@@H]2[C@@H]3CC=C4C[C@@H](CC[C@]4(C)[C@H]3CC[C@]12C)O[C@@H]5O[C@H](CO)[C@@H](O)[C@H](O)[C@H]5O)C(C)C |
| 782 | CC(=C)C1CCC(C)(O)CC1 |
| 783 | CC(=C)CC\C=C(/C)\C=C |
| 784 | OCC(O)CO |
| 785 | Oc1ccc(C=O)cc1 |
| 786 | [O-]c1ccc(C=O)cc1 |
| 787 | CCCCO[C@]1(CO)O[C@H](CO)[C@@H](O)[C@@H]1O |
| 788 | C[C@@]1(CC[C@H]2C(=CC[C@@H]3[C@]2(C)CCC[C@@]3(C)C(=O)O)C1)C=C |
| 789 | C[C@@]1(CC[C@H]2C(=CC[C@@H]3[C@]2(C)CCC[C@@]3(C)C(=O)[O-])C1)C=C |
| 790 | CC1(C)[C@H]2C[C@H]3[C@@H]1[C@@]3(C)C2 |
| 791 | Cc1cc(O)c2C(=O)c3c(O)cc(O)cc3Cc2c1 |
| 792 | Cc1cc(O)c2C(=O)c3c([O-])cc(O)cc3Cc2c1 |
| 793 | Cc1cc([O-])c2C(=O)c3c([O-])cc(O)cc3Cc2c1 |
| 794 | C[C@@H]1COC=C1 |
| 795 | CC(=O)OC(C)(C)[C@H]1CCC(=CC1)C |
| 796 | C[C@H](CCC=C(C)C)CC=O |
| 797 | C[C@@H]1CC[C@@]2(OC1)O[C@H]3C[C@@H]4[C@@H]5CC=C6C[C@@H](O)CC[C@]6(C)[C@H]5CC[C@]4(C)[C@H]3[C@@H]2C |
| 798 | CCCCCCCCCCCCCCCCCCCCO |
| 799 | COC(=O)c1ccccc1 |
| 800 | CC[C@H](\C=C\[C@@H](C)[C@H]1CC[C@H]2[C@@H]3CC=C4C[C@H](CC[C@]4(C)[C@H]3CC[C@]12C)O[C@@H]5O[C@H](CO)[C@@H](O)[C@H](O)[C@H]5O)C(C)C |
| 801 | CCCCCCCCCCCCCCCCCCCCC(=O)[O-] |
| 802 | C[C@@H]1CC[C@@]2([NH2+]C1)O[C@H]3C[C@@H]4[C@@H]5CC=C6C[C@@H](O)CC[C@]6(C)[C@H]5CC[C@]4(C)[C@H]3[C@@H]2C |
| 803 | COc1cc(\C=C/C(=O)OC[C@H](O)CO)ccc1O |
| 804 | OC[C@H](O)COC(=O)\C=C\c1ccc(O)cc1 |
| 805 | C[C@H](CCC(=O)[C@@H](C)[C@H]1C(=O)C[C@@H]2[C@@H]3CC[C@@H]4C[C@H](CC[C@]4(C)[C@H]3CC[C@]12C)O[C@@H]5O[C@H](CO)[C@@H](O)[C@H](O)[C@H]5O[C@@H]6O[C@@H](C)[C@H](O)[C@@H](O)[C@H]6O)CO[C@@H]7O[C@H](CO)[C@@H](O)[C@H](O)[C@H]7O |
| 806 | C[C@@H](CO)CCC(=O)[C@@H](C)[C@H]1C(=O)C[C@@H]2[C@@H]3CC[C@@H]4C[C@@H](O)CC[C@]4(C)[C@H]3CC[C@]12C |
| 807 | CC(=CCC[C@@](C)(OC(=O)c1ccccc1N)C=C)C |
| 808 | C[C@@H](Cc1ccccc1)C=O |
| 809 | COc1cc(\C=C\C(=O)O[C@H]2C[C@@H](O[C@@]3(CO)O[C@H](CO)[C@@H](O)[C@H]3OC(=O)\C=C/c4ccc(O)c(OC)c4)[C@H](O)[C@H](O)[C@@H]2O)ccc1O |
| 810 | CC(=C)[C@@H]1CC[C@@]2(CC[C@]3(C)C(=CC[C@@H]4[C@]5(C)CC[C@@H](O[C@@H]6O[C@H](CO)[C@@H](O)[C@H](O)[C@H]6O)C(C)(C)[C@H]5CC[C@@]34C)[C@@H]12)C(=O)O[C@@H]7O[C@H](CO[C@@H]8O[C@H](CO)[C@@H](O)[C@H](O)[C@H]8O)[C@@H](O)[C@H](O)[C@H]7O |
| 811 | CC(=C)[C@@H]1CC[C@@]2(CC[C@]3(C)C(=CC[C@@H]4[C@]5(C)CC[C@@H](O)C(C)(C)[C@H]5CC[C@@]34C)[C@@H]12)C(=O)O |
| 812 | CC(=C)[C@@H]1CC[C@@]2(CC[C@]3(C)C(=CC[C@@H]4[C@]5(C)CC[C@@H](O)C(C)(C)[C@H]5CC[C@@]34C)[C@@H]12)C(=O)[O-] |
| 813 | CC(C)[C@]12C[C@H]1[C@H](C)C=C2 |
| 814 | COC1=CC=C2C(=CC1=O)[C@H](CCc3cc(O[C@@H]4O[C@H](CO)[C@@H](O)[C@H](O)[C@H]4O)c(OC)c(OC)c23)NC(=O)C |
| 815 | COC1=CC=C2C(=CC1=O)[C@H](CCc3cc(O)c(OC)c(OC)c23)NC(=O)C |
| 816 | C1CC\C=C/2\C[C@@H]2CC1 |
| 817 | CC[C@H]1C[C@H]1C |
| 818 | CC(=CCC\C(=C/COC=O)\C)C |
| 819 | C[C@@H]1CC[C@@]2(OC1)O[C@H]3C[C@@H]4[C@@H]5CC=C6C[C@H](CC[C@]6(C)[C@H]5CC[C@]4(C)[C@H]3[C@@H]2C)O[C@@H]7O[C@H](CO)[C@@H](O)[C@H](O[C@@H]8OC[C@@H](O)[C@H](O)[C@H]8O)[C@H]7O[C@@H]9O[C@@H](C)[C@H](O)[C@@H](O)[C@H]9O |
| 820 | OC[C@@H](O)CO[C@@H]1O[C@H](CO)[C@@H](O)[C@H](O)[C@H]1O |
| 821 | C[C@@H](CCC(=O)[C@@H](C)[C@H]1C(=O)C[C@@H]2[C@@H]3CC=C4C[C@H](CC[C@]4(C)[C@H]3CC[C@]12C)O[C@@H]5O[C@H](CO)[C@@H](O)[C@H](O)[C@H]5O[C@H]6O[C@@H](C)[C@H](O)[C@@H](O)[C@H]6O)CO[C@@H]7O[C@@H](C)[C@H](O)[C@@H](O)[C@H]7O |
| 822 | C[C@H](CO)CCC(=O)[C@@H](C)[C@H]1C(=O)C[C@@H]2[C@@H]3CC=C4C[C@@H](O)CC[C@]4(C)[C@H]3CC[C@]12C |
| 823 | C[C@@H]1O[C@@H](O[C@@H]2[C@@H](O)[C@H](O[C@@H]3O[C@H](CO)[C@@H](O)[C@@H](O)[C@H]3O)[C@@H](CO)O[C@H]2O[C@H]4CC[C@]5(C)[C@H]6CC[C@@]7(C)[C@H](C[C@@H]8O[C@]9(CC[C@@H](COC(=O)C[C@](C)(O)CC(=O)[O-])CO9)[C@@H](C)[C@H]78)[C@@H]6CC=C5C4)[C@H](O)[C@H](O)[C@H]1O |
| 824 | C[C@H]1[C@H]2[C@H](C[C@@H]3[C@@H]4CC=C5C[C@@H](O)CC[C@]5(C)[C@H]4CC[C@]23C)O[C@]16CC[C@@H](COC(=O)C[C@](C)(O)CC(=O)[O-])CO6 |
| 825 | C[C@@H]1CC[C@@]2([NH2+]C1)O[C@H]3C[C@@H]4[C@@H]5CC=C6C[C@H](CC[C@]6(C)[C@H]5CC[C@]4(C)[C@H]3[C@@H]2C)O[C@@H]7O[C@H](CO)[C@@H](O)[C@H](O)[C@H]7O[C@@H]8O[C@@H](C)[C@H](O)[C@@H](O)[C@H]8O |
| 826 | C[C@@H]1CC[C@@]2([NH2+]C1)O[C@H]3C[C@@H]4[C@@H]5CC=C6C[C@H](CC[C@]6(C)[C@H]5CC[C@]4(C)[C@H]3[C@@H]2C)O[C@@H]7O[C@H](CO)[C@@H](O[C@@H]8O[C@H](CO)[C@@H](O)[C@H](O)[C@H]8O)[C@H](O)[C@H]7O[C@@H]9O[C@@H](C)[C@H](O)[C@@H](O)[C@H]9O |
| 827 | C[C@@H](CCC(=O)[C@@H](C)[C@@H]1C(=O)C[C@@H]2[C@@H]3CC[C@@H]4C[C@H](CC[C@]4(C)[C@H]3CC[C@]12C)O[C@@H]5O[C@H](CO)[C@@H](O)[C@H](O)[C@H]5O[C@@H]6O[C@@H](C)[C@H](O)[C@@H](O)[C@H]6O)CO[C@@H]7O[C@@H](C)[C@H](O)[C@@H](O)[C@H]7O |
| 828 | C[C@H](CO)CCC(=O)[C@@H](C)[C@@H]1C(=O)C[C@@H]2[C@@H]3CC[C@@H]4C[C@@H](O)CC[C@]4(C)[C@H]3CC[C@]12C |
| 829 | C[C@@H]1O[C@H](O[C@@H]2[C@@H](O)[C@H](O)[C@@H](CO)O[C@H]2O[C@H]3CC[C@]4(C)[C@H]5CC[C@@]6(C)[C@H](C[C@@H]7O[C@]8(CC[C@@](C)(CO[C@@H]9O[C@H](CO)[C@@H](O)[C@H](O)[C@H]9O)O8)[C@@H](C)[C@H]67)[C@@H]5CC=C4C3)[C@H](O)[C@H](O)[C@H]1O |
| 830 | C[C@H]1[C@H]2[C@H](C[C@@H]3[C@@H]4CC=C5C[C@@H](O)CC[C@]5(C)[C@H]4CC[C@]23C)O[C@]16CC[C@@](C)(CO)O6 |
| 831 | C[C@@H]1O[C@H](O[C@@H]2[C@@H](O)[C@H](O[C@@H]3O[C@H](CO)[C@@H](O)[C@@H](O)[C@H]3O)[C@@H](CO)O[C@H]2O[C@H]4CC[C@]5(C)[C@H]6CC[C@@]7(C)[C@H](C[C@@H]8O[C@]9(CC[C@@](C)(CO[C@@H]%10O[C@H](CO)[C@@H](O)[C@H](O)[C@H]%10O)O9)[C@@H](C)[C@H]78)[C@@H]6CC=C5C4)[C@H](O)[C@H](O)[C@H]1O |
| 832 | C[C@H]1[C@H]2[C@H](C[C@@H]3[C@@H]4CC=C5C[C@@H](O)CC[C@]5(C)[C@H]4CC[C@]23C)O[C@]16CC[C@@](C)(CO[C@@H]7O[C@H](CO)[C@@H](O)[C@H](O)[C@H]7O)O6 |
| 833 | C[C@@H]1O[C@@H](O[C@@H]2[C@@H](O)[C@H](O)[C@@H](CO)O[C@H]2O[C@H]3CC[C@]4(C)[C@H]5CC[C@@]6(C)[C@H](C[C@@H]7O[C@]8(CC[C@@H](COC(=O)C[C@@](C)(O)CC(=O)[O-])CO8)[C@@H](C)[C@H]67)[C@@H]5CC=C4C3)[C@H](O)[C@H](O)[C@H]1O |
| 834 | C[C@H]1[C@H]2[C@H](C[C@@H]3[C@@H]4CC=C5C[C@@H](O)CC[C@]5(C)[C@H]4CC[C@]23C)O[C@]16CC[C@@H](COC(=O)C[C@@](C)(O)CC(=O)[O-])CO6 |
| 835 | C[C@@H]1O[C@@H](O[C@@H]2[C@@H](O)[C@H](O)[C@@H](CO)O[C@H]2O[C@H]3CC[C@]4(C)[C@H]5CC[C@@]6(C)[C@H](C[C@@H]7O[C@]8(CC[C@@H](CO)CO8)[C@@H](C)[C@H]67)[C@@H]5CC=C4C3)[C@H](O)[C@H](O)[C@H]1O |
| 836 | C[C@H]1[C@H]2[C@H](C[C@@H]3[C@@H]4CC=C5C[C@@H](O)CC[C@]5(C)[C@H]4CC[C@]23C)O[C@]16CC[C@@H](CO)CO6 |
| 837 | OC[C@H]1O[C@@H](OC[C@H](O)COC(=O)\C=C\c2ccc(O)cc2)[C@H](O)[C@@H](O)[C@H]1O |
| 838 | CC(=O)\C=C\[C@H]1C(=C)CC[C@@H]2[C@]1(C)CCC[C@]2(C)C(=O)O |
| 839 | CC(=O)\C=C\[C@H]1C(=C)CC[C@@H]2[C@]1(C)CCC[C@]2(C)C(=O)[O-] |
| 840 | CC(=O)CC[C@H]1C(=C)CC[C@@H]2[C@]1(C)CCC[C@]2(C)C(=O)O |
| 841 | CC(=O)CC[C@H]1C(=C)CC[C@@H]2[C@]1(C)CCC[C@]2(C)C(=O)[O-] |
| 842 | C[C@@]1(CC[C@@H]2[C@](O)(CC[C@@H]3C(C)(C)[C@H](O)CC[C@@]23C)C1)C=C |
| 843 | CO[C@H]1OC(=O)C(=C1)CC[C@@H]2C(=C)CC[C@@H]3[C@]2(C)CCC[C@]3(C)C(=O)O |
| 844 | CO[C@H]1OC(=O)C(=C1)CC[C@@H]2C(=C)CC[C@@H]3[C@]2(C)CCC[C@]3(C)C(=O)[O-] |
| 845 | COC(=O)[C@@]1(C)CCC[C@]2(C)[C@@H](CCC3=CCOC3=O)C(=C)CC[C@@H]12 |
| 846 | C[C@]12CCC[C@@](C)([C@@H]1CCC(=C)[C@@H]2CCC(=O)[O-])C(=O)O |
| 847 | C[C@]12CCC[C@@](C)([C@@H]1CCC(=C)[C@@H]2CCC(=O)[O-])C(=O)[O-] |
| 848 | CCCCCCCCCCCCCC(=O)OC |
| 849 | CCCCC\C=C/C\C=C/C\C=C/C\C=C/CCCC(=O)[O-] |
| 850 | CCCCCCCCCCCCCCCCCC(=O)OC |
| 851 | CCCCCCCC\C=C/CCCCCCCC(=O)OC |
| 852 | CCCCC\C=C\C\C=C\CCCCCCCCCC(=O)[O-] |
| 853 | CCCCCCCC\C=C/CCCCCCCCCC(=O)OC |
| 854 | CCCCCCCCCCCCCCCCCCCC(=O)OC |
| 855 | CC\C=C/C\C=C/C\C=C/CCCCCCCCCC(=O)[O-] |
| 856 | CCCCCCCC(=O)OC |
| 857 | CCCCCCCCC(=O)OC |
| 858 | COC(=O)CCCCCCCCC=C |
| 859 | CCCCCCCCCCCCCCCCCCC(=O)OC |
| 860 | CCCCCCC(=O)OC |
| 861 | CCCCCCCCCCCCCC\C=C/CCCC(=O)[O-] |
| 862 | CC\C=C/CC\C=C\C=O |
| 863 | CCCCCCCCCCCCCCCCCCCCCC(=O)[O-] |
| 864 | CC\C=C/C\C=C/C\C=C/CCCCCCCC(=O)[O-] |
| 865 | Nc1ncnc2c1ncn2[C@@H]3O[C@H](CO)[C@@H](O)[C@H]3O |
| 866 | C[C@H]([NH3+])CC(=O)[O-] |
| 867 | Oc1ccc(COCc2ccc(O)cc2)cc1 |
| 868 | C[N+](C)(C)CCO |
| 869 | CCC[C@H]1CCCC[NH2+]1 |
| 870 | C[C@@H]1NC(=O)[C@H]2CCCN2C1=O |
| 871 | CC(C)[C@@H]1NC(=O)[C@H](C)NC1=O |
| 872 | CC(C)C[C@H]1NC(=O)[C@@H](NC1=O)C(C)C |
| 873 | C[C@@H]1NC(=O)[C@@H](Cc2ccccc2)NC1=O |
| 874 | Oc1ccc(cc1O)C(=O)[O-] |
| 875 | Oc1cc(ccc1[O-])C(=O)[O-] |
| 876 | C[NH2+][C@@H](C)[C@H](O)c1ccccc1 |
| 877 | OC[C@H](O)[C@H](O)CO |
| 878 | CCCCCCCCCCCCCC[C@@H](O)C(=O)N[C@@H](CO[C@@H]1O[C@H](CO)[C@@H](O)[C@H](O)[C@H]1O)[C@H](O)\C=C\CCCCC\C=C\CCCCCC |
| 879 | NC1=Nc2c(ncn2[C@@H]3O[C@H](CO)[C@@H](O)[C@H]3O)C(=O)N1 |
| 880 | CCCCCCCCC(O)CCCCCCCC |
| 881 | [NH3+][C@H](Cc1c[nH]cn1)C(=O)[O-] |
| 882 | Oc1ccc(O)c(CC(=O)[O-])c1 |
| 883 | Cc1ncccc1O |
| 884 | Cc1ncccc1[O-] |
| 885 | O=C1NC=[NH+]c2nc[nH]c12 |
| 886 | O=C1NC=Nc2nc[nH]c12 |
| 887 | CC(C)[C@@H]1NC(=O)[C@H](NC1=O)C(C)(C)C |
| 888 | CSCC[C@@H]([NH3+])C(=O)[O-] |
| 889 | COC(=O)C(=C)Cl |
| 890 | CCCCCCCCCCCCCCCCC[C@H](C)CC |
| 891 | Cc1cnc(cn1)[C@@H](O)[C@H](O)[C@H](O)CO |
| 892 | Cc1nccnc1C[C@H](O)[C@H](O)CO |
| 893 | CC1=CNC(=O)NC1=O |
| 894 | CCCCCCC=C |
| 895 | Nc1ncnc2c1ncn2Cc3ccc(O)cn3 |
| 896 | Nc1ncnc2c1ncn2Cc3ccc([O-])cn3 |
| 897 | CCOC(=O)\C=C\c1nccc2c3ccc(C=O)cc3[nH]c12 |
| 898 | OCCCCCO |
| 899 | CCC\C=C\NO |
| 900 | [NH3+][C@@H](Cc1ccccc1)C(=O)[O-] |
| 901 | OC[C@@H]1NC(=O)[C@@H](Cc2ccccc2)NC1=O |
| 902 | O=C1[C@@H]2CCCN2C(=O)[C@@H]3CCCN13 |
| 903 | CC(C)[C@H]1NC(=O)[C@]2(C)CCCN2C1=O |
| 904 | Oc1ccc(C=O)cc1O |
| 905 | Oc1cc(C=O)ccc1[O-] |
| 906 | [NH3+][C@@H](CO)C(=O)[O-] |
| 907 | C[C@@H](O)[C@H]([NH3+])C(=O)[O-] |
| 908 | C[n+]1cccc(c1)C(=O)[O-] |
| 909 | O=C1NC=CC(=O)N1 |
| 910 | CC(C)[C@@H]1NC(=O)[C@@H](NC1=O)C(C)C |
| 911 | Oc1cc2OC(=CC(=O)c2c(O)c1O)c3ccccc3 |
| 912 | COc1cc2CCN3Cc4c5OCOc5ccc4[C@H](C)[C@@H]3c2cc1OC |
| 913 | CCCCC\C=C\C(=O)CCc1ccc(O)c(OC)c1 |
| 914 | CCCCCCCCCCCCCCCC(=O)OCC(COC(=O)CCCCCCCCCCCCCCC)OC(=O)CCCCCCCCCCCCCCC |
| 915 | O=C1N=CNc2[nH+]c[nH]c12 |
| 916 | CCCCCC\C=C\CCCCCCCC(=O)OC |
| 917 | CCCCCCCCCCCCCCCC(=O)N |
| 918 | Nc1ncnc2[nH]cnc12 |
| 919 | NC1=Nc2[nH+]c[nH]c2C(=O)N1 |
| 920 | NC1=Nc2nc[nH]c2C(=O)N1 |
| 921 | CC[C@H](CC[C@@H](C)[C@H]1CC[C@H]2[C@@H]3CCC4=CC(=O)CC[C@]4(C)[C@H]3CC[C@]12C)C(C)C |
| 922 | Cc1cc(O)c2C(=O)c3c(O)cccc3C(=O)c2c1 |
| 923 | Cc1cc(O)c2C(=O)c3c([O-])cccc3C(=O)c2c1 |
| 924 | Cc1cc([O-])c2C(=O)c3c([O-])cccc3C(=O)c2c1 |
| 925 | O=Cc1occc1 |
| 926 | [O-]C(=O)CCCCCCCC=O |
| 927 | CCCCCCCCCCCCCCCC(=O)OC[C@H](O)CO |
| 928 | CC(=CCC\C(=C/C=O)\C)C |
| 929 | COc1cc(ccc1O)C(=O)[O-] |
| 930 | COc1cc(ccc1[O-])C(=O)[O-] |
| 931 | Oc1ccccc1O |
| 932 | CCCCCCCCCCCCCCCC(=O)OC[C@@H](O)CO |
| 933 | C[C@@H](CCC=C(C)C)CC=O |
| 934 | OCc1oc(C=O)cc1 |
| 935 | OC[C@H]1O[C@H]([C@H](O)[C@@H]1O)n2cnc3c(O)nc(O)nc23 |
| 936 | CCCCCCCCCCCCCC[C@@H](O)C(=O)N[C@@H](CO)[C@H](O)\C=C\CC\C=C/CCCCCCCCC |
| 937 | CCCCCCCCCCCCCC[C@@H](O)C(=O)N[C@@H](CO[C@H]1O[C@H](CO)[C@@H](O)[C@H](O)[C@H]1O)[C@H](O)\C=C\CC\C=C/CCCCCCCCC |
| 938 | CCCC\C=N\O |
| 939 | CCCCCCCCCCCCC\C=C/C(=O)[O-] |
| 940 | CCCCCCCCC[C@@H](O)CC(=O)CCc1ccc(O)c(OC)c1 |
| 941 | C[C@H](CCC=C(C)C)[C@H]1CC[C@@]2(C)[C@@H]3CC[C@H]4C(C)(C)[C@@H](O)CC[C@@]45C[C@@]35CC[C@]12C |
| 942 | CC(C)[C@@H]1NC(=O)[C@H](Cc2ccc(O)cc2)NC1=O |
| 943 | Oc1ccc(C[C@@H]2NC(=O)[C@H](Cc3ccccc3)NC2=O)cc1 |
| 944 | CC(C)C[C@@H]1NC(=O)[C@H](Cc2ccc(O)cc2)NC1=O |
| 945 | CCCCOC=C |
| 946 | Cc1cncc(n1)[C@@H](O)[C@@H](O)C(O)O |
| 947 | CC(=O)N[C@H](CCC(=O)[O-])C(=O)[O-] |
| 948 | C[C@@H](O)[C@H](O)[C@@H](O)[C@@H](O)C=O |
| 949 | OC[C@H]1O[C@@H]([C@H](O)[C@@H]1O)n2cnc3C(=O)NC=[NH+]c23 |
| 950 | CCCCCCCCCCCCCCCCC(=O)OC[C@H](O)CO |
| 951 | [NH3+][C@@H]1NC=[NH+]c2c1[nH+]cn2[C@@H]3O[C@H](O)[C@@H](O)[C@H]3O |
| 952 | [NH3+][C@@H]1NC=Nc2c1[nH+]cn2[C@@H]3O[C@H](O)[C@@H](O)[C@H]3O |
| 953 | N[C@@H]1NC=Nc2c1[nH+]cn2[C@@H]3O[C@H](O)[C@@H](O)[C@H]3O |
| 954 | N[C@@H]1NC=Nc2c1ncn2[C@@H]3O[C@H](O)[C@@H](O)[C@H]3O |
| 955 | CCCCCCCCC\C=C\CCCCCCC(=O)[O-] |
| 956 | CC(C)[C@H](C)\C=C\[C@H](C)[C@@H]1CC[C@H]2[C@@]1(C)CC[C@@H]3[C@]4(C)CC[C@H](O)C[C@@]45OO[C@@]23C=C5 |
| 957 | CC(=O)Nc1cc(C)on1 |
| 958 | CCCCCCCCCCCCCCCCC[C@@H](C)CC |
| 959 | CC[C@@H]([NH3+])C(=O)[O-] |
| 960 | Cc1cnccn1 |
| 961 | OC[C@H]1O[C@H](C[C@@H]1O)N2C=CC(=O)NC2=O |
| 962 | OC[C@H]1O[C@H](C[C@@H]1O)N2C=CC(=O)[N-]C2=O |
| 963 | CCCCCC[C@H]1O[C@H]1\C=C\[C@@H](O)\C=C\CCCCCC(=O)[O-] |
| 964 | CCCCCC\C=C\C\C=C\CCCCCCCCC(=O)[O-] |
| 965 | Oc1cc(cc(O)c1O)C(=O)OC[C@H]2O[C@@H](OC(=O)c3cc(O)c(O)c(O)c3)[C@@H](OC(=O)c4cc(O)c(O)c(OC(=O)c5cc(O)c(O)c([O-])c5)c4)[C@@H](OC(=O)c6cc(O)c(O)c(O)c6)[C@@H]2OC(=O)c7cc(O)c(O)c(O)c7 |
| 966 | Oc1cc(cc(O)c1O)C(=O)OC[C@H]2O[C@@H](OC(=O)c3cc(O)c([O-])c(O)c3)[C@@H](OC(=O)c4cc(O)c(O)c(OC(=O)c5cc(O)c(O)c([O-])c5)c4)[C@@H](OC(=O)c6cc(O)c(O)c(O)c6)[C@@H]2OC(=O)c7cc(O)c(O)c(O)c7 |
| 967 | Oc1cc(cc(O)c1O)C(=O)OC[C@H]2O[C@@H](OC(=O)c3cc(O)c(O)c(O)c3)[C@@H](OC(=O)c4cc(O)c(O)c(OC(=O)c5cc(O)c(O)c([O-])c5)c4)[C@@H](OC(=O)c6cc(O)c([O-])c(O)c6)[C@@H]2OC(=O)c7cc(O)c(O)c(O)c7 |
| 968 | Oc1cc(cc(O)c1O)C(=O)OC[C@H]2O[C@@H](OC(=O)c3cc(O)c([O-])c(O)c3)[C@@H](OC(=O)c4cc(O)c(O)c(OC(=O)c5cc(O)c(O)c([O-])c5)c4)[C@@H](OC(=O)c6cc(O)c([O-])c(O)c6)[C@@H]2OC(=O)c7cc(O)c(O)c(O)c7 |
| 969 | Oc1cc(cc(O)c1O)C(=O)OC[C@H]2O[C@@H](OC(=O)c3cc(O)c(O)c(O)c3)[C@@H](OC(=O)c4cc(O)c(O)c(OC(=O)c5cc(O)c(O)c([O-])c5)c4)[C@@H](OC(=O)c6cc(O)c(O)c(O)c6)[C@@H]2OC(=O)c7cc(O)c([O-])c(O)c7 |
| 970 | Oc1cc(cc(O)c1O)C(=O)OC[C@H]2O[C@@H](OC(=O)c3cc(O)c([O-])c(O)c3)[C@@H](OC(=O)c4cc(O)c(O)c(OC(=O)c5cc(O)c(O)c([O-])c5)c4)[C@@H](OC(=O)c6cc(O)c(O)c(O)c6)[C@@H]2OC(=O)c7cc(O)c([O-])c(O)c7 |
| 971 | Oc1cc(cc(O)c1O)C(=O)OC[C@H]2O[C@@H](OC(=O)c3cc(O)c(O)c(O)c3)[C@@H](OC(=O)c4cc(O)c(O)c(OC(=O)c5cc(O)c(O)c([O-])c5)c4)[C@@H](OC(=O)c6cc(O)c([O-])c(O)c6)[C@@H]2OC(=O)c7cc(O)c([O-])c(O)c7 |
| 972 | Oc1cc(cc(O)c1O)C(=O)OC[C@H]2O[C@@H](OC(=O)c3cc(O)c([O-])c(O)c3)[C@@H](OC(=O)c4cc(O)c(O)c(OC(=O)c5cc(O)c(O)c([O-])c5)c4)[C@@H](OC(=O)c6cc(O)c([O-])c(O)c6)[C@@H]2OC(=O)c7cc(O)c([O-])c(O)c7 |
| 973 | Oc1cc(cc(O)c1O)C(=O)OC[C@H]2O[C@@H](OC(=O)c3cc(O)c([O-])c(O)c3)[C@@H](OC(=O)c4cc(O)c([O-])c(OC(=O)c5cc(O)c(O)c([O-])c5)c4)[C@@H](OC(=O)c6cc(O)c([O-])c(O)c6)[C@@H]2OC(=O)c7cc(O)c([O-])c(O)c7 |
| 974 | Oc1cc(cc([O-])c1O)C(=O)Oc2cc(cc(O)c2[O-])C(=O)O[C@@H]3[C@H](OC(=O)c4cc(O)c([O-])c(O)c4)O[C@H](COC(=O)c5cc(O)c([O-])c(O)c5)[C@@H](OC(=O)c6cc(O)c([O-])c(O)c6)[C@@H]3OC(=O)c7cc(O)c([O-])c(O)c7 |
| 975 | C[NH2+][C@H](C)[C@H](O)c1ccccc1 |
| 976 | COc1cc2C[C@H](CO)[C@@H](CO)[C@@H](c3ccc(O)c(OC)c3)c2cc1O |
| 977 | COc1cc2C[C@H](CO)[C@@H](CO[C@@H]3O[C@H](CO)[C@H](O)[C@@H](O)[C@@H]3O)[C@@H](c4ccc(O)c(OC)c4)c2cc1O |
| 978 | c1ccc2c(c1)[nH]c3c[nH+]ccc23 |
| 979 | c1ccc2c(c1)[nH]c3cnccc23 |
| 980 | CC1=CN([C@H]2C[C@H](O)[C@@H](CO)O2)C(=O)NC1=O |
| 981 | Oc1ccc(O)cc1 |
| 982 | CC(C)C[C@H]([NH3+])C(=O)[O-] |
| 983 | C[C@@H](CO)C1=C([O-])C(=O)c2c3CCCC(C)(C)c3ccc2C1=O |
| 984 | COc1ccc(cc1)C(=O)[O-] |
| 985 | CCCCCCCC\C=C/CCCCCCCCCC(=O)[O-] |
| 986 | CCCCCCCCCCCCCC[C@@H](O)C(=O)N[C@@H](CO)[C@H](O)\C=C\CC\C=C\CCCCCCCCC |
| 987 | CCCCCCCCCCCCCC[C@@H](O)C(=O)N[C@@H](CO[C@H]1O[C@H](CO)[C@@H](O)[C@H](O)[C@H]1O)[C@H](O)\C=C\CC\C=C\CCCCCCCCC |
| 988 | C[C@@H]([NH3+])C(=O)[O-] |
| 989 | NC(=O)OCCCc1ccccc1 |
| 990 | OC[C@@H]1O[C@H]([C@H](O)[C@@H]1O)N2C=CC(=O)NC2=O |
| 991 | CC(C)[C@H](C)CC[C@@H](C)[C@H]1CC[C@H]2[C@@H]3CC[C@@H]4CC(=CC[C@]4(C)[C@H]3CC[C@]12C)O |
| 992 | [NH3+][C@@H](CSSC[C@@H]([NH3+])C(=O)[O-])C(=O)[O-] |
| 993 | N[C@H](CSSC[C@H]([NH3+])C(=O)[O-])C(=O)[O-] |
| 994 | N[C@@H](CSSC[C@@H]([NH3+])C(=O)[O-])C(=O)[O-] |
| 995 | N[C@@H](CSSC[C@@H](N)C(=O)[O-])C(=O)[O-] |
| 996 | Oc1ccc(\C=C/C(=O)[O-])cc1 |
| 997 | COc1cc(\C=C/C(=O)[O-])ccc1O |
| 998 | [NH3+]CCCC(=O)[O-] |
| 999 | O[C@@H](C=O)[C@@H](O)[C@H](O)[C@H](O)C(=O)[O-] |
| 1000 | O[C@H]1[C@H](Oc2cc3OC(=CC(=O)c3c(O)c2O)c4ccccc4)O[C@@H]([C@@H](O)[C@@H]1O)C(=O)[O-] |
| 1001 | CC(=O)OC(C)(C)[C@@H]1CC=C(C)C(=O)C1 |
| 1002 | Oc1ccc(C[C@H](OC(=O)[C@@H]2[C@H](C(=Cc3ccc(O)c(O)c23)C(=O)O[C@@H](Cc4ccc(O)c(O)c4)C(=O)[O-])c5ccc(O)c(O)c5)C(=O)[O-])cc1O |
| 1003 | Oc1ccc(C[C@H](OC(=O)[C@H]2[C@@H](C(=Cc3ccc(O)c(O)c23)C(=O)O[C@@H](Cc4ccc(O)c(O)c4)C(=O)[O-])c5ccc(O)c([O-])c5)C(=O)[O-])cc1O |
| 1004 | COC(=O)[C@H](Cc1ccc(O)c(O)c1)OC(=O)[C@@H]2[C@H](C(=Cc3ccc(O)c(O)c23)C(=O)O[C@@H](Cc4ccc(O)c(O)c4)C(=O)OC)c5ccc(O)c(O)c5 |
| 1005 | COC(=O)[C@H](Cc1ccc(O)c(O)c1)OC(=O)[C@@H]2[C@H](C(=Cc3ccc(O)c(O)c23)C(=O)O[C@@H](Cc4ccc(O)c(O)c4)C(=O)OC)c5ccc(O)c([O-])c5 |
| 1006 | CC1(C)[C@H]2CC[C@H](C2)C1=C |
| 1007 | COc1cc2OC(=CC(=O)c2c(O)c1O)c3ccc(OC)c(OC)c3 |
| 1008 | Oc1cc(O)c2C(=O)C[C@H](Oc2c1)c3ccc(O)c(O)c3 |
| 1009 | COc1ccc(cc1O)[C@@H]2CC(=O)c3c(O)cc(O[C@@H]4O[C@H](CO[C@@H]5O[C@@H](C)[C@H](O)[C@@H](O)[C@H]5O)[C@@H](O)[C@H](O)[C@H]4O)cc3O2 |
| 1010 | CCCC[C@H](C)O |
| 1011 | CC(C)[C@@H]1CC[C@@H](C)CC1=O |
| 1012 | CC(C)[C@@H]1CC[C@@H](C)C[C@@H]1OC(=O)C |
| 1013 | O[C@H]1[C@H](O)[C@@H](COC(=O)\C=C\c2ccc(O)cc2)O[C@@H](Oc3cc4c(O[C@@H]5O[C@H](COC(=O)CC(=O)[O-])[C@@H](OC(=O)CC(=O)[O-])[C@H](O)[C@H]5O)cc(O)cc4[o+]c3c6ccc(O)cc6)[C@@H]1O |
| 1014 | O[C@H]1[C@H](O)[C@@H](COC(=O)\C=C\c2ccc(O)cc2)O[C@@H](Oc3cc4c(O[C@@H]5O[C@H](COC(=O)CC(=O)[O-])[C@@H](OC(=O)CC(=O)[O-])[C@H](O)[C@H]5O)cc(O)cc4[o+]c3c6ccc([O-])cc6)[C@@H]1O |
| 1015 | CC(C)[C@@H]1CCC(=C[C@H]1O)C |
| 1016 | CC(C)[C@H]1CC[C@]2(C)O[C@@H]2C1=O |
| 1017 | C[C@@H]1CCC(=C(C)C)C(=O)C1 |
| 1018 | CC(=C1CC[C@]2(C)O[C@@H]2C1=O)C |
| 1019 | COc1cc(ccc1O)C2=CC(=O)c3c(O)c(O)c(OC)cc3O2 |
| 1020 | COc1cc(ccc1[O-])C2=CC(=O)c3c(O)c(O)c(OC)cc3O2 |
| 1021 | COc1c(O)c2C(=O)C=C(Oc2c(OC)c1OC)c3ccc(O)cc3 |
| 1022 | COc1c(O)c2C(=O)C=C(Oc2c(OC)c1OC)c3ccc([O-])cc3 |
| 1023 | OC[C@H]1O[C@@H](Oc2cc(O)c3C(=O)C=C(Oc3c2)c4ccc(O)c(O)c4)[C@H](O)[C@@H](O)[C@@H]1O |
| 1024 | CC12CCC(CC1)C(C)(C)O2 |
| 1025 | C\C\1=C/CCC(=C)[C@H]2CC(C)(C)[C@H]2CC1 |
| 1026 | CC(=CC\C=C(/C)\C=C)C |
| 1027 | CC(C)[C@H]1CCC(=CC1=O)C |
| 1028 | CC(C)[C@H]1CCC(=C)C=C1 |
| 1029 | CC(C)[C@@H]1CC[C@@H](C)[C@@]23CCC(=C)[C@@H]2[C@@H]13 |
| 1030 | CC(C)[C@@]12CCC(=C)[C@@H]1C2 |
| 1031 | CC(=C)[C@@H]1CC=C(C)C(=O)C1 |
| 1032 | OCc1cc(O)c2C(=O)c3c(O)cccc3C(=O)c2c1 |
| 1033 | OCc1cc(O)c2C(=O)c3c([O-])cccc3C(=O)c2c1 |
| 1034 | OCc1cc([O-])c2C(=O)c3c([O-])cccc3C(=O)c2c1 |
| 1035 | CC(=C)[C@@H]1CC=C(C)[C@@H](O)C1 |
| 1036 | C[C@@H]1CC[C@@]2(CC[C@]3(C)C(=CC[C@@H]4[C@@]5(C)CC[C@H](O)C(C)(C)[C@@H]5CC[C@@]34C)[C@@H]2[C@H]1C)C(=O)O |
| 1037 | C[C@@H]1CC[C@@]2(CC[C@]3(C)C(=CC[C@@H]4[C@@]5(C)CC[C@H](O)C(C)(C)[C@@H]5CC[C@@]34C)[C@@H]2[C@H]1C)C(=O)[O-] |
| 1038 | CC(C)[C@@H]1CC[C@@]2(C)[C@@H]3CCC(=C)[C@@H]3[C@@H]12 |
| 1039 | CC(C)[C@H]1CC[C@@H](C)c2ccc(C)cc12 |
| 1040 | C=Cc1ccccc1 |
| 1041 | CC(C)[C@H]1CC[C@@H](C)CC1=O |
| 1042 | Oc1ccc(\C=C\C(=O)[O-])cc1 |
| 1043 | c1ccc2cccc2cc1 |
| 1044 | CC1(C)[C@@H]2CCC(=C)[C@H]1C2 |
| 1045 | CC(C)C1=CC=C(C)CC1 |
| 1046 | CC(C)[C@@]12CC=C(C)[C@@H]1C2 |
| 1047 | CC(=CCC\C(=C/CCC(=C)C=C)\C)C |
| 1048 | C[C@@H]1CC[C@@H]2[C@H](C3=C(C)CC[C@H]13)C2(C)C |
| 1049 | CC(C)[C@]12CC=C(C)[C@H]1C2 |
| 1050 | CC(=C1CC[C@@](C)(C=C)[C@@H](C1)C(=C)C)C |
| 1051 | CCCCCCCCCCOC(=O)C |
| 1052 | CC(C)[C@@]12C[C@@H]1[C@@H](C)C(=O)C2 |
| 1053 | CC(C)CCC[C@@H](C)CCC[C@@H](C)CCC\C(=C\CO)\C |
| 1054 | CC(=C1CCC(=CC1=O)C)C |
| 1055 | COc1ccc(cc1)C2=CC(=O)c3c(O)cc([O-])cc3O2 |
| 1056 | COc1ccc(cc1)C2=CC(=O)c3c(O)cc(O[C@@H]4O[C@H](CO[C@@H]5O[C@@H](C)[C@H](O)[C@@H](O)[C@H]5O)[C@@H](O)[C@H](O)[C@H]4O)cc3O2 |
| 1057 | CC(=C)[C@@H]1CC=C(C)[C@H](O)C1 |
| 1058 | CC1(C)C[C@H]2[C@H]1CC[C@@]3(C)O[C@@H]3CCC2=C |
| 1059 | C1CCC2CCCCC2C1 |
| 1060 | CC(C)c1ccc(C)cc1O |
| 1061 | CC(C)[C@@H]1CC[C@@H](C)[C@@]23CC=C(C)[C@@H]2[C@@H]13 |
| 1062 | C\C=C(/C)\C=C\C=C(C)C |
| 1063 | CC(=C)C1=CC=C(C)CC1 |
| 1064 | CCCCC\C=C\C=C\C=C |
| 1065 | CC\C=C/CCO |
| 1066 | CC(C)[C@H]1CC[C@@H](C)C[C@@H]1O |
| 1067 | CC(C)[C@@H]1CC[C@@H](C)C[C@@H]1O |
| 1068 | CC(C)[C@@H]1CC[C@H](C)CC1=O |
| 1069 | CCCCCCCCCCC |
| 1070 | CC(C)[C@H]1CC[C@@H](C)C[C@H]1OC(=O)C |
| 1071 | CC(C)CC=O |
| 1072 | CC(=C)[C@@H]1CC[C@@]2(C)O[C@H]2C1 |
| 1073 | CCC\C=C\C=O |
| 1074 | CCCC[C@@H](C)O |
| 1075 | C[C@@H]1CC[C@H](C[C@H]1O)C(=C)C |
| 1076 | COc1ccc(cc1O)C2=CC(=O)c3c(O)cc([O-])cc3O2 |
| 1077 | CC(C)[C@@H]1CC\C(=C\CCC(=C)\C=C\1)\C |
| 1078 | CCCCCCCCCCCCCCCCCCCCCCCCCCCCCCC |
| 1079 | CC(C)C=O |
| 1080 | CC(=C)\C=C\C=C(/C)\C=C |
| 1081 | CC(C)[C@H]1CCC(=C)[C@H]2CCC(=C[C@H]12)C |
| 1082 | CC(C)[C@@H]1CC=C(C)[C@@H]2CCC(=C[C@@H]12)C |
| 1083 | Oc1ccc(cc1)[C@@H]2CC(=O)c3c(O)cc(O)cc3O2 |
| 1084 | C[C@@H]1CC[C@H]([C@H](O)C1)C(=C)C |
| 1085 | CC(C)[C@H]1CCC(=C)[C@@H]2CCC(=C[C@H]12)C |
| 1086 | COC(=O)c1ccccc1C(=O)OC |
| 1087 | COc1ccc(cc1O)C2=CC(=O)c3c(O)cc(O[C@@H]4O[C@H](CO[C@@H]5O[C@@H](C)[C@H](O)[C@@H](O)[C@H]5O)[C@@H](O)[C@H](O)[C@H]4O)cc3O2 |
| 1088 | Cc1ccc2ccccc2c1 |
| 1089 | COc1cc(O)c2C(=O)C=C(Oc2c1)c3ccc(O)cc3 |
| 1090 | COc1cc(O)c2C(=O)C=C(Oc2c1)c3ccc([O-])cc3 |
| 1091 | CC(=CC[C@@H](CO)C(=C)C)C |
| 1092 | C[C@@H]1CC[C@H](C=C1)C(=C)C |
| 1093 | CC(C)[C@H]1CC=C(C)[C@H]2CCC(=C[C@@H]12)C |
| 1094 | CC(=CCC[C@]1(C)[C@H]2CC[C@H](C2)C1=C)C |
| 1095 | CCCCC[C@H](O)CC |
| 1096 | CC[C@H](C)C=O |
| 1097 | CCC(O)CC |
| 1098 | CSC |
| 1099 | OC[C@H]1O[C@@H](Oc2cc(O)c3C(=O)C[C@H](Oc3c2)c4ccc(O)cc4)[C@H](O)[C@@H](O)[C@@H]1O |
| 1100 | Cc1ccc(C)c2ccoc12 |
| 1101 | CCCCCCOC(=O)C |
| 1102 | CC(C)(O)[C@H]1CC[C@](C)(O1)C=C |
| 1103 | CCCC(=O)OCC\C=C/CC |
| 1104 | CC\C=C/CCOC(=O)C |
| 1105 | CC(C)[C@@H]1CC[C@H](C)c2ccc(C)cc12 |
| 1106 | CC(=CCC[C@]1(C)[C@H]2CC=C(C)[C@@H]1C2)C |
| 1107 | CC\C=C\CCO |
| 1108 | Oc1ccc(C[C@@H](OC(=O)[C@@H]2[C@H](C(=Cc3ccc([O-])c(O)c23)C(=O)O[C@H](Cc4ccc(O)c(O)c4)C(=O)[O-])c5ccc(O)c(O)c5)C(=O)[O-])cc1O |
| 1109 | Oc1ccc(C[C@@H](OC(=O)[C@@H]2[C@H](C(=Cc3ccc([O-])c(O)c23)C(=O)O[C@H](Cc4ccc(O)c(O)c4)C(=O)[O-])c5ccc([O-])c(O)c5)C(=O)[O-])cc1O |
| 1110 | COC(=O)[C@@H](Cc1ccc(O)c(O)c1)OC(=O)[C@H]2[C@H](C(=Cc3ccc([O-])c(O)c23)C(=O)O[C@H](Cc4ccc(O)c(O)c4)C(=O)OC)c5ccc(O)c(O)c5 |
| 1111 | COC(=O)[C@@H](Cc1ccc(O)c(O)c1)OC(=O)[C@H]2[C@H](C(=Cc3ccc([O-])c(O)c23)C(=O)O[C@H](Cc4ccc(O)c(O)c4)C(=O)OC)c5ccc([O-])c(O)c5 |
| 1112 | Oc1ccc(C[C@@H](OC(=O)C2=Cc3ccc([O-])c(O)c3[C@@H]([C@@H]2c4ccc(O)c(O)c4)C(=O)[O-])C(=O)[O-])cc1O |
| 1113 | Oc1ccc(C[C@@H](OC(=O)C2=Cc3ccc([O-])c(O)c3[C@@H]([C@@H]2c4ccc([O-])c(O)c4)C(=O)[O-])C(=O)[O-])cc1O |
| 1114 | CC(C)(C)c1ccc(O)cc1 |
| 1115 | Oc1ccc(cc1O)[C@@H]2[C@H](C(=O)[O-])c3c(O)c(O)ccc3C=C2C(=O)[O-] |
| 1116 | Oc1ccc2C=C([C@@H]([C@H](C(=O)[O-])c2c1O)c3ccc([O-])c(O)c3)C(=O)[O-] |
| 1117 | CC(=O)OC(C)(C)[C@H]1CC=C(C)C(=O)C1 |
| 1118 | Oc1ccc(C[C@@H](OC(=O)C2=Cc3cc(O)c(O)cc3[C@@H]([C@H]2C(=O)[O-])c4ccc(O)c(O)c4)C(=O)[O-])cc1O |
| 1119 | Oc1ccc(C[C@@H](OC(=O)C2=Cc3cc(O)c(O)cc3[C@@H]([C@H]2C(=O)[O-])c4ccc(O)c([O-])c4)C(=O)[O-])cc1O |
| 1120 | Oc1ccc(C[C@@H](OC(=O)C2=Cc3cc(O)c([O-])cc3[C@@H]([C@H]2C(=O)[O-])c4ccc(O)c([O-])c4)C(=O)[O-])cc1O |
| 1121 | C[C@@H]1CC[C@@H](CC1=O)C(=C)C |
| 1122 | COc1ccc(cc1)C2=CC(=O)c3c(O)cc(O[C@@H]4O[C@H](CO)[C@@H](O)[C@H](O)[C@H]4O[C@@H]5O[C@@H](C)[C@H](O)[C@@H](O)[C@H]5O)cc3O2 |
| 1123 | OC(=O)C1=Cc2cc(O)c(O)cc2[C@@H]([C@H]1C(=O)[O-])c3ccc(O)c(O)c3 |
| 1124 | OC(=O)C1=Cc2cc(O)c(O)cc2[C@@H]([C@H]1C(=O)[O-])c3ccc(O)c([O-])c3 |
| 1125 | Oc1cc2C=C([C@@H]([C@@H](c3ccc(O)c([O-])c3)c2cc1O)C(=O)[O-])C(=O)[O-] |
| 1126 | Oc1ccc(cc1[O-])[C@@H]2[C@@H](C(=O)[O-])C(=Cc3cc(O)c([O-])cc23)C(=O)[O-] |
| 1127 | CC(=CC\C=C(/C=C)\C=O)C |
| 1128 | Oc1ccc(C[C@H](OC(=O)[C@H]2[C@@H](C(=Cc3ccc([O-])c(O)c23)C(=O)O[C@@H](Cc4ccc(O)c(O)c4)C(=O)[O-])c5ccc(O)c(O)c5)C(=O)[O-])cc1O |
| 1129 | Oc1ccc(C[C@H](OC(=O)[C@H]2[C@@H](C(=Cc3ccc([O-])c(O)c23)C(=O)O[C@@H](Cc4ccc(O)c(O)c4)C(=O)[O-])c5ccc(O)c([O-])c5)C(=O)[O-])cc1O |
| 1130 | C[C@@H]1O[C@@H](OC[C@H]2O[C@@H](Oc3cc(O)c4C(=O)C[C@H](Oc4c3)c5ccc(O)c(O)c5)[C@H](O)[C@@H](O)[C@@H]2O)[C@H](O)[C@H](O)[C@H]1O |
| 1131 | C[C@@H]1O[C@@H](OC[C@H]2O[C@@H](Oc3cc(O)c4C(=O)C=C(Oc4c3)c5ccc(O)cc5)[C@H](O)[C@@H](O)[C@@H]2O)[C@H](O)[C@H](O)[C@H]1O |
| 1132 | C[C@@H]1O[C@@H](OC[C@H]2O[C@@H](Oc3cc(O)c4C(=O)C=C(Oc4c3)c5ccc([O-])cc5)[C@H](O)[C@@H](O)[C@@H]2O)[C@H](O)[C@H](O)[C@H]1O |
| 1133 | COC(=O)[C@H](Cc1ccc(O)c(O)c1)OC(=O)C2=Cc3ccc([O-])c(O)c3[C@@H]([C@@H]2c4ccc(O)c(O)c4)C(=O)O[C@H](Cc5ccc(O)c(O)c5)C(=O)[O-] |
| 1134 | COC(=O)[C@H](Cc1ccc(O)c(O)c1)OC(=O)C2=Cc3ccc([O-])c(O)c3[C@@H]([C@@H]2c4ccc(O)c([O-])c4)C(=O)O[C@H](Cc5ccc(O)c(O)c5)C(=O)[O-] |
| 1135 | Oc1ccc(C[C@H](OC(=O)[C@@H]2[C@@H](C(=Cc3ccc([O-])c(O)c23)C(=O)[O-])c4ccc(O)c(O)c4)C(=O)[O-])cc1O |
| 1136 | Oc1ccc(C[C@H](OC(=O)[C@@H]2[C@@H](C(=Cc3ccc([O-])c(O)c23)C(=O)[O-])c4ccc(O)c([O-])c4)C(=O)[O-])cc1O |
| 1137 | C[C@H](CC=O)c1ccccc1 |
| 1138 | CCCCCC(=O)CC |
| 1139 | CC(C)[C@@H]1CCC(=CC1=O)C |
| 1140 | C[C@@H]1CC[C@H](CC1=O)C(=C)C |
| 1141 | C[C@@H]1CC[C@@H](C(=C)C)C(=O)C1 |
| 1142 | CC1(C)[C@@H]2CC[C@H]1C(=C)C2 |
| 1143 | C\C\1=C/CC(C)(C)\C=C\C\C(=C/CC1)\C |
| 1144 | CC[C@H](O)C=C |
| 1145 | CC(C)[C@@H]1CC[C@@H](C)[C@H]2CCC(=C)C=C12 |
| 1146 | CC[C@@H](C)C(=O)OC |
| 1147 | Oc1cc(O)c2C(=O)C=C(Oc2c1)c3ccc(O)c(O)c3 |
| 1148 | Oc1ccc(cc1)C2=CC(=O)c3c(O)cc(O)cc3O2 |
| 1149 | Oc1cc(O)c2C(=O)C=C(Oc2c1)c3ccc([O-])cc3 |
| 1150 | CC(=C)[C@@H]1CC[C@@]2(CC[C@]3(C)[C@H](CC[C@@H]4[C@@]5(C)C[C@@H](O)[C@H](O)C(C)(C)[C@@H]5CC[C@@]34C)[C@@H]12)C(=O)O |
| 1151 | CC(=C)[C@@H]1CC[C@@]2(CC[C@]3(C)[C@H](CC[C@@H]4[C@@]5(C)C[C@@H](O)[C@H](O)C(C)(C)[C@@H]5CC[C@@]34C)[C@@H]12)C(=O)[O-] |
| 1152 | C[C@@H]1CC[C@@]2(CC[C@]3(C)C(=CC[C@@H]4[C@@]5(C)C[C@@H](O)[C@H](O)[C@@](C)(CO)[C@@H]5CC[C@@]34C)[C@@H]2[C@H]1C)C(=O)O |
| 1153 | C[C@@H]1CC[C@@]2(CC[C@]3(C)C(=CC[C@@H]4[C@@]5(C)C[C@@H](O)[C@H](O)[C@@](C)(CO)[C@@H]5CC[C@@]34C)[C@@H]2[C@H]1C)C(=O)[O-] |
| 1154 | CC1(C)[C@@H]2CC[C@@]1(C)[C@@H](O)C2 |
| 1155 | CC(=CCC[C@](C)(O)[C@H]1CC[C@]2(C)[C@@H]1CC[C@@H]3[C@@]4(C)CCC(=O)C(C)(C)[C@@H]4CC[C@@]23C)C |
| 1156 | CC(=CCC[C@@](O)(CO)[C@H]1CC[C@]2(C)[C@@H]1CC[C@@H]3[C@@]4(C)CCC(=O)C(C)(C)[C@@H]4CC[C@@]23C)C |
| 1157 | CC1(C)CC[C@]2(CO)CC[C@]3(C)C(=CC[C@@H]4[C@@]5(C)CC[C@H](O)C(C)(C)[C@@H]5CC[C@@]34C)[C@@H]2C1 |
| 1158 | CC1(C)[C@H]2CC[C@]1(C)[C@@H](O)C2 |
| 1159 | CC(C)[C@@H]1CC=C(C)C=C1 |
| 1160 | CC1=CC[C@@H]2[C@H](C1)C2(C)C |
| 1161 | CC(=CCC[C@](C)(O)C=C)C |
| 1162 | C\C\1=C\CC(C)(C)\C=C/C\C(=C/CC1)\C |
| 1163 | C[C@@H]1CC[C@@]2(CC[C@]3(C)C(=CC[C@H]4[C@@]3(C)CC[C@@]5(O)[C@](C)(CO)C[C@H](O)C[C@]45C)[C@@H]2[C@H]1C)C(=O)O |
| 1164 | C[C@@H]1CC[C@@]2(CC[C@]3(C)C(=CC[C@H]4[C@@]3(C)CC[C@@]5(O)[C@](C)(CO)C[C@H](O)C[C@]45C)[C@@H]2[C@H]1C)C(=O)[O-] |
| 1165 | CCOC(=O)c1sc(NC(=O)\C=C/c2ccc(OC)c(OC)c2)c(C(=O)OCC)c1C |
| 1166 | CC1(C)CCC[C@@]2(C)[C@H]3CCC[C@@H](CC2=C)[C@H]13 |
| 1167 | CC(=CCC[C@@](C)(O)[C@H]1CC[C@]2(C)[C@H]1CC[C@@H]3[C@]4(C)CCC(=O)C(C)(C)[C@@H]4CC[C@@]23C)C |
| 1168 | C\C=C\c1ccc2OCOc2c1 |
| 1169 | C[C@@H]1CC=C(C)[C@H](C)C1 |
| 1170 | CC[C@H](CC[C@@H](C)[C@H]1CC[C@@H]2[C@@H]3CC=C4C[C@@H](CC[C@@]4(C)[C@H]3CC[C@]12C)O[C@@H]5O[C@H](CO)[C@@H](O)[C@H](O)[C@H]5O)C(C)C |
| 1171 | CC(=CCc1cc2c(OC(=O)c3c2oc4cc(O)ccc34)cc1O)C |
| 1172 | CC(=CCc1c(O)ccc([C@H]2COc3cc(O)ccc3C2=O)c1O)C |
| 1173 | CC(=CCc1c(O)ccc(C(=O)\C=C\c2ccc(O)cc2)c1O)C |
| 1174 | CC1(C)Oc2cc([O-])c(cc2C=C1)C(=O)\C=C\c3ccc(O)cc3 |
| 1175 | COc1cc2O[C@H](CC(=O)c2cc1CC=C(C)C)c3ccc(O)cc3 |
| 1176 | CC(=CCc1cc(C(=O)\C=C\c2ccc(O)cc2)c(O)cc1O)C |
| 1177 | CC(=CCC[C@@](C)(C=C)\C=C\c1ccc(O)cc1)C |
| 1178 | CC1(C)Oc2ccc3C(=O)C=C(Oc3c2C=C1)c4ccc(O)c(O)c4 |
| 1179 | O=C1Oc2cc3occc3cc2C=C1 |
| 1180 | CC(C)(O)[C@@H]1Cc2c(O)c(ccc2O1)C(=O)\C=C\c3ccc(O)cc3 |
| 1181 | CCOC(=O)CCc1ccc2occc2c1O |
| 1182 | CC1(C)O[C@@H]1Cc2cc3c(OC(=O)c4c3oc5cc(O)ccc45)cc2O |
| 1183 | CC1(C)O[C@@H]1Cc2cc3c(OC(=O)c4c3oc5cc(O)ccc45)cc2[O-] |
| 1184 | CC(=CCC[C@](C)(C=C)\C=C\c1ccc(O)cc1)C |
| 1185 | CC(=CCc1cc2C(=O)C[C@@H](Oc2cc1O)c3ccc(O)cc3)C |
| 1186 | CC1(C)Oc2c(C[C@@H]1O)c(O)ccc2C(=O)\C=C\c3ccc(O)cc3 |
| 1187 | CC1(C)Oc2cc3oc4c5ccc(O)cc5OC(=O)c4c3cc2C[C@@H]1O |
| 1188 | CC1(C)Oc2cc3oc4c5ccc([O-])cc5OC(=O)c4c3cc2C[C@@H]1O |
| 1189 | CC(C)(O)[C@@H]1Cc2cc3c4C(=O)Oc5cc(O)ccc5c4oc3cc2O1 |
| 1190 | CC(C)(O)[C@@H]1Cc2cc3c4C(=O)Oc5cc([O-])ccc5c4oc3cc2O1 |
| 1191 | CC1(C)Oc2cc3OC(=O)c4c(oc5cc(O)ccc45)c3cc2[C@H](O)[C@H]1O |
| 1192 | CC1(C)Oc2ccc(cc2C=C1)C3=COc4cc(O)ccc4C3=O |
| 1193 | CC1(C)Oc2ccc(cc2C=C1)C3=COc4cc([O-])ccc4C3=O |
| 1194 | Oc1ccc2C(=O)C(=COc2c1)c3cccc(O)c3C=O |
| 1195 | Oc1cccc(C2=COc3cc([O-])ccc3C2=O)c1C=O |
| 1196 | CC(=CCc1c(O)cc(O)c2C(=O)C[C@@H](Oc12)c3ccc(O)cc3)C |
| 1197 | COc1cc(O)c(C=O)cc1C(=O)\C=C\c2ccc(O)cc2 |
| 1198 | COc1cc([O-])c(C=O)cc1C(=O)\C=C\c2ccc(O)cc2 |
| 1199 | CC1(C)CCc2cc3c(OC(=O)c4c3oc5c(O)cccc45)cc2O1 |
| 1200 | CC1(C)CCc2cc3c(OC(=O)c4c3oc5c([O-])cccc45)cc2O1 |
| 1201 | COc1cc(O)c(cc1C=O)C(=O)\C=C\c2ccc(O)cc2 |
| 1202 | COc1cc([O-])c(cc1C=O)C(=O)\C=C\c2ccc(O)cc2 |
| 1203 | CC1(C)Oc2ccc(cc2C[C@H]1O)C3=COc4cc(O)ccc4C3=O |
| 1204 | CC1(C)Oc2ccc(cc2C[C@H]1O)C3=COc4cc([O-])ccc4C3=O |
| 1205 | CCCCCCCCCCCCCCCCCCCCCCCCCCCCCC |
| 1206 | CC[C@@H](\C=C\[C@H](C)[C@H]1CC[C@@H]2[C@@H]3CC=C4C[C@H](O)CC[C@]4(C)[C@H]3CC[C@]12C)C(C)C |
| 1207 | C[C@H]1O[C@@H](O[C@H]2CC[C@@]3(C)[C@@H](CC[C@]4(C)[C@@H]3C=C[C@]56OC[C@@]7(CCC(C)(C)C[C@@H]57)[C@H](O)C[C@@]46C)[C@]2(C)COC(=O)C)[C@H](O)[C@@H](O[C@H]8O[C@H](CO)[C@@H](O)[C@H](O)[C@H]8O[C@H]9O[C@H](CO)[C@@H](O)[C@H](O)[C@H]9O)[C@H]1O |
| 1208 | CCC[C@H](CC\C=C\C=C\C#CC#C\C=C/CO)OC(=O)C |
| 1209 | C[C@H]1O[C@@H](O[C@H]2CC[C@@]3(C)[C@@H](CC[C@]4(C)[C@@H]3C=C[C@]56OC[C@@]7(CCC(C)(C)C[C@@H]57)[C@@H](O)C[C@@]46C)[C@]2(C)CO)[C@H](O)[C@@H](O[C@@H]8O[C@H](CO)[C@@H](O)[C@H](OC(=O)C)[C@H]8O)[C@H]1O |
| 1210 | CO[C@@H]1C=C2[C@H]3CC(C)(C)CC[C@]3(CO)[C@@H](O)C[C@@]2(C)[C@]4(C)CC[C@H]5[C@](C)(CO)[C@H](CC[C@]5(C)[C@@H]14)O[C@@H]6O[C@H](C)[C@H](O)[C@H](O[C@@H]7O[C@H](OC(=O)C)[C@@H](O)[C@H](O)[C@H]7O)[C@H]6O |
| 1211 | C[C@H]1O[C@@H](O[C@H]2CC[C@@]3(C)[C@@H](CC[C@]4(C)[C@@H]3C=C[C@]56OC[C@@]7(CCC(C)(C)C[C@@H]57)[C@H](O)C[C@@]46C)[C@]2(C)CO)[C@H](O)[C@@H](O[C@@H]8O[C@H](CO)[C@@H](O)[C@H](O)[C@H]8OC(=O)C)[C@H]1O |
| 1212 | C[C@H]1O[C@@H](O[C@H]2CC[C@@]3(C)[C@@H](CC[C@]4(C)[C@@H]3C=C[C@]56OC[C@@]7(CCC(C)(C)C[C@@H]57)[C@H](O)C[C@@]46C)[C@]2(C)CO)[C@H](O)[C@@H](O[C@@H]8O[C@H](CO)[C@@H](O)[C@H](OC(=O)C)[C@H]8O)[C@H]1O |
| 1213 | C[C@H]1O[C@@H](O[C@H]2CC[C@@]3(C)[C@@H](CC[C@]4(C)[C@@H]3C=C[C@]56OC[C@@]7(CCC(C)(C)C[C@@H]57)[C@H](O)C[C@@]46C)[C@]2(C)CO)[C@H](O)[C@@H](O[C@@H]8O[C@H](COC(=O)C)[C@@H](O)[C@H](O)[C@H]8O)[C@H]1O |
| 1214 | OC[C@@H](O)C(O)[C@@H](O)CO |
| 1215 | C[C@@H]1CC[C@@H]2[C@@H]1[C@@H]3[C@H](CCC2=C)C3(C)C |
| 1216 | CCCC(=O)CC\C=C\C=C\C#CC#C\C=C/CO |
| 1217 | CCC[C@H](O)CC\C=C\C=C\C#CC#C\C=C/CO |
| 1218 | CCCCCC\C=C\C=C\C#CC#C\C=C/CO |
| 1219 | CC(=C)[C@H]1CC=C(C)C(=O)C1 |
| 1220 | COc1cc2cc3C(=O)OCc3c(c4ccc5OCOc5c4)c2c(OC)c1OC |
| 1221 | COc1ccc(cc1OC)c2c3C(=O)OCc3c(O)c4cc5OCOc5cc24 |
| 1222 | CC(C)[C@@H]1CC[C@]2(C)[C@H]3CC=C(C)[C@@H]2[C@@H]13 |
| 1223 | CC(C)[C@@H]1CC[C@@H](C)[C@@]23CC=C(C)[C@@H]2[C@@H]13 |
| 1224 | C[C@H]1O[C@@H](O[C@H]2CC[C@@]3(C)[C@@H](CC[C@]4(C)[C@@H]3C=CC5=C6CC(C)(C)CC[C@]6(CO)[C@H](O)C[C@@]45C)[C@]2(C)CO)[C@H](O)[C@@H](O[C@@H]7O[C@H](COC(=O)C)[C@@H](O)[C@H](OC(=O)C)[C@H]7O)[C@H]1O |
| 1225 | COC(=O)CCNC(=O)c1cc2c3ccccc3[nH]c2c(n1)C(=O)C |
| 1226 | COC(=O)\C=C/NC(=O)c1cc2c3ccccc3[nH]c2c(n1)C(=O)C |
| 1227 | C[C@H](O)c1nc(cc2c3ccccc3[nH]c12)C(=O)[O-] |
| 1228 | OC[C@@H](O)c1nc(cc2c3ccccc3[nH]c12)C(=O)[O-] |
| 1229 | COC(=O)c1cc2c3ccccc3[nH]c2c(n1)[C@H](O)CO |
| 1230 | CCCCOC(=O)c1cc2c3ccccc3[nH]c2c(n1)[C@H](O)CO |
| 1231 | COc1cc(CCC(=O)[O-])cc(c1O)c2cc(CCC(=O)[O-])cc(OC)c2O[C@@H]3O[C@H](CO)[C@@H](O)[C@H](O)[C@H]3O |
| 1232 | CCCCOC(=O)CCc1cc(OC)c(O[C@@H]2O[C@H](CO)[C@@H](O)[C@H](O)[C@H]2O)c(c1)c3cc(CCC(=O)[O-])cc(OC)c3O |
| 1233 | CCCCOC(=O)CCc1cc(OC)c(O)c(c1)c2cc(CCC(=O)[O-])cc(OC)c2O[C@@H]3O[C@H](CO)[C@@H](O)[C@H](O)[C@H]3O |
| 1234 | CCCCOC(=O)CCc1cc(OC)c(O)c(c1)c2cc(CCC(=O)OCCCC)cc(OC)c2O[C@@H]3O[C@H](CO)[C@@H](O)[C@H](O)[C@H]3O |
| 1235 | CCCCOC(=O)CCc1ccc(O[C@@H]2O[C@H](CO)[C@@H](O)[C@H](O)[C@H]2O)c(OC)c1 |
| 1236 | COc1cc(C[C@@H]2[C@@H](Cc3ccc4OCOc4c3)COC2=O)cc(OC)c1OC |
| 1237 | COc1ccc(\C=C\COC(=O)\C(=C/C)\COC(=O)\C(=C\C)\C)cc1OC |
| 1238 | CCCCCCC(=O)[O-] |
| 1239 | COc1cc(O)c2C(=O)C=C(C)Oc2c1 |
| 1240 | CC(=CCC\C(=C/C\C=C(\C)/C=C)\C)C |
| 1241 | CC1(C)C[C@@H]2C[C@H]1CC2=C |
| 1242 | OC[C@H](O[C@@H]1O[C@H](CO)[C@@H](O)[C@H](O)[C@H]1O)c2nc(cc3c4ccccc4[nH]c23)C(=O)[O-] |
| 1243 | C[C@H]1O[C@@H](O[C@H]2CC[C@@]3(C)[C@@H](CC[C@]4(C)[C@@H]3C=CC5=C6CC(C)(C)CC[C@]6(CO)[C@H](O)C[C@@]45C)[C@]2(C)CO)[C@H](O)[C@@H](O[C@@H]7O[C@H](CO)[C@@H](O)[C@H](O[C@@H]8O[C@H](CO)[C@@H](O)[C@H](O)[C@H]8O)[C@H]7O)[C@H]1O |
| 1244 | COc1ccccc1O |
| 1245 | CC1(C)CC[C@@]2(CC[C@]3(C)C(=CC[C@@H]4[C@@]5(C)CC[C@H](O)[C@@](C)(C=O)[C@@H]5CC[C@@]34C)[C@@H]2C1)C(=O)O |
| 1246 | CC1(C)CC[C@@]2(CC[C@]3(C)C(=CC[C@@H]4[C@@]5(C)CC[C@H](O)[C@@](C)(C=O)[C@@H]5CC[C@@]34C)[C@@H]2C1)C(=O)[O-] |
| 1247 | C[C@H]1O[C@@H](O[C@H]2[C@H](C)O[C@@H](OC(=O)[C@]34CCC(C)(C)C[C@H]3C5=CC[C@@H]6[C@@]7(C)CC[C@H](O[C@@H]8O[C@@H]([C@@H](O[C@@H]9O[C@H](CO)[C@@H](O[C@@H]%10O[C@H](CO)[C@H](O)[C@H](O)[C@H]%10O)[C@H](O)[C@H]9O)[C@H](O[C@@H]%11OC[C@@H](O)[C@H](O)[C@@H]%11O)[C@H]8O)C(=O)[O-])[C@@](C)(C=O)[C@@H]7CC[C@@]6(C)[C@]5(C)CC4)[C@H](O[C@@H]%12OC[C@@H](O)[C@H](O[C@@H]%13OC[C@@H](O)[C@H](O)[C@H]%13O)[C@H]%12O)[C@@H]2O)[C@H](O)[C@@H](O[C@@H]%14OC[C@@H](O)[C@H](O)[C@H]%14O)[C@H]1O |
| 1248 | CCC[C@H]1CCC(=O)O1 |
| 1249 | CCCC\C=C\C(=O)[O-] |
| 1250 | CC(C)CCC[C@H](C)CCC[C@H](C)CCCC(=O)C |
| 1251 | COc1c(O)cc(O)c2C(=O)C=C(Oc12)c3ccc(O)cc3 |
| 1252 | COc1c(O)cc(O)c2C(=O)C=C(Oc12)c3ccc([O-])cc3 |
| 1253 | C\C=C/1\C[C@@H](C)[C@](O)(CO)C(=O)OCC2=CC[N@@+]3([O-])CC[C@@H](OC1=O)[C@@H]23 |
| 1254 | COc1cc(\C=C/2\[C@@H](Cc3ccc4OCOc4c3)COC2=O)cc(OC)c1OC |
| 1255 | COc1ccc(\C=C/2\[C@@H](Cc3ccc4OCOc4c3)COC2=O)cc1OC |
| 1256 | OC[C@H]1O[C@@H](OC2=C(Oc3cc(O)cc(O)c3C2=O)c4ccc(O)c(O)c4)[C@H](O)[C@@H](O)[C@@H]1O |
| 1257 | C[C@@H]1O[C@@H](OC2=C(Oc3cc(O[C@@H]4O[C@@H](C)[C@H](O)[C@@H](O)[C@H]4O)cc(O)c3C2=O)c5ccc(O)cc5)[C@H](O)[C@H](O)[C@H]1O |
| 1258 | C[C@@H]1O[C@@H](OC2=C(Oc3cc(O[C@@H]4O[C@@H](C)[C@H](O)[C@@H](O)[C@H]4O)cc(O)c3C2=O)c5ccc([O-])cc5)[C@H](O)[C@H](O)[C@H]1O |
| 1259 | COc1ccc(\C=C\2/[C@@H](Cc3ccc4OCOc4c3)COC2=O)cc1OC |
| 1260 | C[C@H]1CC[C@H]2[C@H]1[C@H]3[C@@H](CC[C@@]2(C)O)C3(C)C |
| 1261 | CC1(C)CCC[C@@]2(C)[C@@H]3CC[C@@H]([C@H]13)C2=C |
| 1262 | CC1=CC[C@H]2[C@H]3[C@@H]1[C@]2(C)CCCC3(C)C |
| 1263 | CC1(C)CC[C@]2(CO)[C@@H](O)C[C@]3(C)C(=CC[C@@H]4[C@@]5(C)CC[C@H](O)C(C)(C)[C@@H]5CC[C@@]34C)[C@@H]2C1 |
| 1264 | CO[C@@H]1C=C2[C@@H]3CC(C)(C)CC[C@]3(CO)[C@@H](O)C[C@@]2(C)[C@]4(C)CC[C@H]5C(C)(C)[C@H](CC[C@]5(C)[C@@H]14)O[C@@H]6O[C@H](CO[C@@H]7O[C@H](CO)[C@@H](O)[C@H](O)[C@H]7O)[C@@H](O[C@@H]8O[C@@H](C)[C@H](O)[C@@H](O)[C@H]8O)[C@H](O)[C@H]6O |
| 1265 | C[C@H]1CCCC1=O |
| 1266 | CC1(C)[C@@H]2CC[C@@H](CO)[C@H]1C2 |
| 1267 | COc1cc(\C=C\2/[C@@H](Cc3ccc4OCOc4c3)COC2=O)cc(OC)c1OC |
| 1268 | CCCCCC\C=C\C(=O)[O-] |
| 1269 | C[C@@H]1CC(=O)C=C2CC[C@H](C[C@@]12C)C(=C)C |
| 1270 | CCCC[C@H]1CCC(=O)O1 |
| 1271 | CCCCC\C=C\C(=O)[O-] |
| 1272 | COc1c(O)cc2OC(=CC(=O)c2c1O)c3ccccc3 |
| 1273 | C[C@H]1CC[C@]23[C@H](C)CC[C@H](C[C@@H]12)C3(C)C |
| 1274 | C[C@H]1O[C@@H](O[C@H]2CC[C@@]3(C)[C@H](CC[C@]4(C)[C@@H]3C=CC5=C6C[C@@](C)(CO)CC[C@]6(CO)[C@H](O)C[C@@]45C)[C@]2(C)CO)[C@H](O)[C@@H](O[C@@H]7O[C@H](CO)[C@@H](O)[C@H](O)[C@H]7O[C@@H]8O[C@H](CO)[C@@H](O)[C@H](O)[C@H]8O)[C@H]1O |
| 1275 | CCCCC(=O)[O-] |
| 1276 | CCCCCCCCCCC\C=C\CCCCC(=O)[O-] |
| 1277 | CCCCCCCCCCC\C=C/CCCCC(=O)[O-] |
| 1278 | CC(=O)[C@H]1CC[C@H]2[C@@H]3CC=C4C[C@@H](O)CC[C@]4(C)[C@H]3CC[C@]12C |
| 1279 | CC1(C)C[C@H]2C3=C[C@@H](O)[C@@H]4[C@@]5(C)CCC(=O)C(C)(C)[C@@H]5CC[C@@]4(C)[C@]3(C)CC[C@]2(CC1=O)C(=O)O |
| 1280 | CC1(C)C[C@H]2C3=C[C@@H](O)[C@@H]4[C@@]5(C)CCC(=O)C(C)(C)[C@@H]5CC[C@@]4(C)[C@]3(C)CC[C@]2(CC1=O)C(=O)[O-] |
| 1281 | C[C@H]1O[C@@H](O[C@H]2CC[C@@]3(C)[C@@H](CC[C@]4(C)[C@@H]3C=C[C@]56OC[C@@]7(CCC(C)(C)C[C@H]57)[C@@H](O)C[C@@]46C)[C@]2(C)CO)[C@H](O)[C@@H](O)[C@H]1O |
| 1282 | C[C@H]1O[C@@H](O[C@H]2CC[C@@]3(C)[C@@H](CC[C@]4(C)[C@@H]3C=C[C@]56OC[C@@]7(CCC(C)(C)C[C@H]57)[C@H](O)C[C@@]46C)[C@]2(C)CO)[C@H](O)[C@@H](O)[C@H]1O |
| 1283 | C[C@@H]1CC[C@]23CO[C@@]4(C=C[C@@H]5[C@@]6(C)CC[C@H](O[C@@H]7O[C@H](C)[C@H](O)[C@H](O)[C@H]7O[C@@H]8O[C@H](CO)[C@@H](O)[C@H](O)[C@H]8O[C@@H]9OC[C@@H](O)[C@H](O)[C@H]9O)[C@@](C)(CO)[C@@H]6CC[C@@]5(C)[C@]4(C)C[C@H]2O)[C@@H]3[C@H]1C |
| 1284 | C[C@@H]1CC[C@]2(CO)[C@H](O)C[C@]3(C)C(=C[C@@H](O)[C@H]4[C@@]5(C)CC[C@H](O[C@@H]6O[C@H](C)[C@H](O)[C@H](O)[C@H]6O[C@@H]7O[C@H](CO)[C@@H](O)[C@H](O)[C@H]7O[C@@H]8OC[C@@H](O)[C@H](O)[C@H]8O)C(C)(C)[C@H]5CC[C@@]34C)[C@H]2[C@H]1C |
| 1285 | C[C@@H]1CC[C@]2(CO)[C@H](O)C[C@]3(C)C(=C[C@@H](O)[C@@H]4[C@@]5(C)CC[C@H](O[C@@H]6O[C@H](C)[C@H](O)[C@H](O)[C@H]6O[C@@H]7O[C@H](CO)[C@@H](O)[C@H](O)[C@H]7O[C@@H]8O[C@@H](C)[C@H](O)[C@@H](O)[C@H]8O)C(C)(C)[C@@H]5CC[C@@]34C)[C@H]2[C@H]1C |
| 1286 | C[C@@H]1CC[C@]2(CO)[C@@H]3O[C@@H]3[C@]4(C)C(=C[C@@H](O)[C@@H]5[C@@]6(C)CC[C@H](O[C@@H]7O[C@H](C)[C@H](O)[C@H](O)[C@H]7O[C@@H]8O[C@H](CO)[C@@H](O)[C@H](O)[C@H]8O[C@@H]9OC[C@@H](O)[C@H](O)[C@H]9O)C(C)(C)[C@@H]6CC[C@@]45C)[C@H]2[C@H]1C |
| 1287 | C[C@H]1O[C@@H](O[C@H]2CC[C@@]3(C)[C@@H](CC[C@]4(C)[C@@H]3C=C[C@]56OC[C@@]7(C[C@H](O)[C@@H](C)[C@H](C)[C@@H]57)[C@H](O)C[C@@]46C)C2(C)C)[C@H](O[C@@H]8O[C@H](CO)[C@@H](O)[C@H](O)[C@H]8O[C@@H]9OC[C@@H](O)[C@H](O)[C@H]9O)[C@@H](O)[C@H]1O |
| 1288 | C[C@@H]1O[C@@H](O[C@@H]2[C@@H](O)[C@H](O)[C@@H](CO)O[C@H]2O[C@@H]3[C@@H](O)[C@@H](O)[C@@H](C)O[C@H]3O[C@H]4CC[C@@]5(C)[C@@H](CC[C@]6(C)[C@@H]5C=C[C@]78OC[C@@]9(C[C@H](O)[C@@H](C)[C@H](C)[C@@H]79)[C@H](O)C[C@@]68C)C4(C)C)[C@H](O)[C@H](O)[C@H]1O |
| 1289 | C[C@@H]1CC[C@]23CO[C@@]4(C=C[C@@H]5[C@@]6(C)CC[C@H](O[C@@H]7O[C@H](CO)[C@@H](O)[C@H](O)[C@H]7O[C@@H]8O[C@H](CO)[C@@H](O)[C@H](O)[C@H]8O[C@@H]9OC[C@@H](O)[C@H](O)[C@H]9O)[C@@](C)(CO)[C@H]6CC[C@@]5(C)[C@]4(C)C[C@H]2O)[C@@H]3[C@H]1C |
| 1290 | C[C@@H]1CC[C@]23CO[C@@]4(C=C[C@@H]5[C@@]6(C)CC[C@H](O[C@@H]7O[C@H](CO)[C@@H](O)[C@H](O)[C@H]7O[C@@H]8O[C@H](CO)[C@@H](O)[C@H](O)[C@H]8O[C@@H]9OC[C@@H](O)[C@H](O)[C@H]9O)C(C)(C)[C@H]6CC[C@@]5(C)[C@]4(C)C[C@H]2O)[C@@H]3[C@H]1C |
| 1291 | C[C@@H]1CC[C@]23CO[C@@]4(C=C[C@@H]5[C@@]6(C)CC[C@H](O[C@@H]7O[C@H](C)[C@H](O)[C@H](O)[C@H]7O[C@@H]8O[C@H](CO)[C@@H](O)[C@H](O)[C@H]8O[C@@H]9OC[C@@H](O)[C@H](O)[C@H]9O)C(C)(C)[C@H]6CC[C@@]5(C)[C@]4(C)C[C@H]2O)[C@@H]3[C@H]1C |
| 1292 | C[C@@H]1CC[C@]23CO[C@@]4(C=C[C@@H]5[C@@]6(C)CC[C@H](O[C@@H]7O[C@H](C)[C@H](O)[C@H](O)[C@H]7O[C@@H]8O[C@H](CO)[C@@H](O)[C@H](O)[C@H]8O[C@@H]9O[C@@H](C)[C@H](O)[C@@H](O)[C@H]9O)C(C)(C)[C@H]6CC[C@@]5(C)[C@]4(C)C[C@H]2O)[C@@H]3[C@H]1C |
| 1293 | C[C@@H]1O[C@@H](O[C@@H]2[C@@H](O)[C@H](O)[C@@H](CO)O[C@H]2O[C@@H]3[C@@H](O)[C@@H](O)[C@@H](C)O[C@H]3O[C@H]4CC[C@@]5(C)[C@H](CC[C@]6(C)[C@@H]5C=C[C@]78OC[C@@]9(CCC(C)(C)C[C@H]79)[C@H](O)C[C@@]68C)C4(C)C)[C@H](O)[C@H](O)[C@H]1O |
| 1294 | C[C@H]1O[C@@H](O[C@H]2CC[C@@]3(C)[C@@H](CC[C@]4(C)[C@@H]3C=C[C@]56OC[C@@]7(CCC(C)(C)C[C@@H]57)[C@H](O)C[C@@]46C)C2(C)C)[C@H](O[C@@H]8O[C@H](CO)[C@@H](O)[C@H](O)[C@H]8O[C@@H]9OC[C@@H](O)[C@H](O)[C@H]9O)[C@@H](O)[C@H]1O |
| 1295 | Oc1cc(O)c2C(=O)C=C(Oc2c1)C(=O)[O-] |
| 1296 | COc1cc(O)c2C(=O)C=C(CO[C@@H]3O[C@H](CO)[C@@H](O)[C@H](O)[C@H]3O)Oc2c1 |
| 1297 | CC1(C)CC[C@]23CO[C@@]4(C=C[C@@H]5[C@@]6(C)CC[C@H](O)C(C)(C)[C@@H]6CC[C@@]5(C)[C@]4(C)C[C@@H]2O)[C@@H]3C1 |
| 1298 | CC1(C)CC[C@]23CO[C@@]4(C=C[C@@H]5[C@@]6(C)CC[C@H](O)[C@@](C)(CO)[C@@H]6CC[C@@]5(C)[C@]4(C)C[C@@H]2O)[C@@H]3C1 |
| 1299 | CC1(C)CC[C@]23CO[C@@]4(C=C[C@@H]5[C@@]6(C)CC[C@H](O)[C@@](C)(CO)[C@@H]6CC[C@@]5(C)[C@]4(C)C[C@H]2O)[C@@H]3C1 |
| 1300 | C[C@]1(CO)CC[C@]2(CO)[C@@H](O)C[C@]3(C)C(=C2C1)C=C[C@@H]4[C@@]5(C)CC[C@H](O)[C@@](C)(CO)[C@@H]5CC[C@@]34C |
| 1301 | COc1ccc(cc1)C2=COc3cc(O[C@@H]4O[C@H](CO[C@@H]5O[C@H](CO)[C@@H](O)[C@H](O)[C@H]5O)[C@@H](O)[C@H](O)[C@H]4O)ccc3C2=O |
| 1302 | COc1cc(C[C@H](CO)c2cc(\C=C\CO)cc(OC)c2O[C@@H]3O[C@H](CO)[C@@H](O)[C@H](O)[C@H]3O)ccc1O |
| 1303 | COc1cc(ccc1O[C@@H]2O[C@H](CO)[C@@H](O)[C@H](O)[C@H]2O)[C@H]3Oc4c(OC)cc(\C=C\CO)cc4[C@@H]3CO |
| 1304 | CO[C@@H]1C=C2[C@H]3CC(C)(C)CC[C@]3(CO)[C@@H](O)C[C@@]2(C)[C@]4(C)CC[C@H]5[C@](C)(CO)[C@H](CC[C@]5(C)[C@@H]14)O[C@@H]6O[C@H](C)[C@H](O)[C@H](O[C@@H]7O[C@H](CO)[C@@H](O)[C@H](O)[C@H]7O[C@@H]8OC[C@@H](O)[C@H](O)[C@H]8O)[C@H]6O |
| 1305 | CO[C@@H]1C=C2[C@H]3CC(C)(C)CC[C@]3(CO)[C@@H](O)C[C@@]2(C)[C@]4(C)CC[C@H]5[C@](C)(CO)[C@H](CC[C@]5(C)[C@@H]14)O[C@@H]6O[C@H](CO[C@@H]7O[C@H](CO)[C@@H](O)[C@H](O)[C@H]7O)[C@@H](O[C@@H]8O[C@@H](C)[C@H](O)[C@@H](O)[C@H]8O)[C@H](O)[C@H]6O |
| 1306 | C[C@H]1O[C@@H](O[C@H]2CC[C@@]3(C)[C@@H](CC[C@]4(C)[C@@H]3C=C[C@]56OC[C@@]7(CCC(C)(C)C[C@@H]57)[C@@H](O)C[C@@]46C)[C@]2(C)CO)[C@H](O)[C@@H](O[C@@H]8O[C@H](CO)[C@@H](O)[C@H](O)[C@H]8O)[C@H]1O |
| 1307 | C[C@H]1O[C@@H](O[C@H]2CC[C@@]3(C)[C@@H](CC[C@]4(C)[C@@H]3C=CC5=C6CC(C)(C)CC[C@]6(CO)[C@H](O)C[C@@]45C)[C@]2(C)CO)[C@H](O)[C@@H](O[C@@H]7O[C@H](CO)[C@@H](O)[C@H](O)[C@H]7O)[C@H]1O |
| 1308 | CO[C@@H]1C=C2[C@@H]3CC(C)(C)CC[C@]3(CO)[C@@H](O)C[C@@]2(C)[C@]4(C)CC[C@@H]5[C@](C)(CO)[C@H](CC[C@]5(C)[C@@H]14)O[C@@H]6O[C@H](C)[C@H](O)[C@H](O[C@@H]7O[C@H](CO)[C@@H](O)[C@H](O)[C@H]7O)[C@H]6O |
| 1309 | CO[C@@H]1C=C2[C@@H]3CC(C)(C)CC[C@]3(CO)[C@H](O)C[C@@]2(C)[C@]4(C)CC[C@@H]5[C@](C)(CO)[C@H](CC[C@]5(C)[C@@H]14)O[C@@H]6O[C@H](C)[C@H](O)[C@H](O[C@@H]7O[C@H](CO)[C@@H](O)[C@H](O)[C@H]7O)[C@H]6O |
| 1310 | C[C@@H]1O[C@@H](O[C@H]2[C@H](O)[C@@H](O)[C@H](OC[C@H]3O[C@@H](O[C@H]4CC[C@@]5(C)[C@@H](CC[C@]6(C)[C@@H]5C=CC7=C8CC(C)(C)CC[C@]8(CO)[C@H](O)C[C@@]67C)C4(C)C)[C@H](O)[C@@H](O)[C@@H]3O)O[C@@H]2CO)[C@H](O)[C@H](O)[C@H]1O |
| 1311 | C[C@@H]1O[C@@H](O[C@H]2[C@H](O)[C@@H](O)[C@H](O[C@H]3CC[C@@]4(C)[C@@H](CC[C@]5(C)[C@@H]4C=C[C@]67OC[C@@]8(CCC(C)(C)C[C@@H]68)[C@@H](O)C[C@@]57C)C3(C)C)O[C@@H]2CO[C@@H]9O[C@H](CO)[C@@H](O)[C@H](O)[C@H]9O)[C@H](O)[C@H](O)[C@H]1O |
| 1312 | C[C@H]1O[C@@H](O[C@H]2CC[C@@]3(C)[C@@H](CC[C@]4(C)[C@@H]3C=C[C@]56OC[C@@]7(CCC(C)(C)C[C@@H]57)[C@H](O)C[C@@]46C)[C@]2(C)CO)[C@H](O)[C@@H](O[C@@H]8O[C@H](CO)[C@@H](O)[C@H](O)[C@H]8O)[C@H]1O |
| 1313 | C[C@H]1O[C@@H](O[C@H]2CC[C@@]3(C)[C@@H](CC[C@]4(C)[C@@H]3C=C[C@]56OC[C@@]7(CCC(C)(C)C[C@@H]57)[C@@H](O)C[C@@]46C)C2(C)C)[C@H](O)[C@@H](O[C@@H]8O[C@H](CO)[C@@H](O)[C@H](O)[C@H]8O)[C@H]1O |
| 1314 | C[C@H]1O[C@@H](O[C@H]2CC[C@@]3(C)[C@@H](CC[C@]4(C)[C@@H]3C=CC5=C6CC(C)(C)CC[C@]6(CO)[C@@H](O)C[C@@]45C)[C@]2(C)CO)[C@H](O)[C@@H](O[C@@H]7O[C@H](CO)[C@@H](O)[C@H](O)[C@H]7C)[C@H]1O |
| 1315 | C[C@H]1O[C@@H](O[C@H]2CC[C@@]3(C)[C@@H](CC[C@]4(C)[C@@H]3C=CC5=C6C[C@@](C)(CO)CC[C@]6(CO)[C@H](O)C[C@@]45C)[C@]2(C)CO)[C@H](O)[C@@H](O[C@@H]7O[C@H](CO)[C@@H](O)[C@H](O)[C@H]7O)[C@H]1O |
| 1316 | C[C@H]1O[C@@H](O[C@H]2CC[C@@]3(C)[C@@H](CC[C@]4(C)[C@@H]3C=CC5=C6CC(C)(C)CC[C@]6(CO)CC[C@@]45C)[C@]2(C)CO)[C@H](O)[C@@H](O[C@@H]7O[C@H](CO)[C@@H](O)[C@H](O)[C@H]7O)[C@H]1O |
| 1317 | C[C@@H]1O[C@@H](O[C@H]2[C@H](O)[C@@H](O)[C@H](O[C@H]3CC[C@@]4(C)[C@@H](CC[C@]5(C)[C@@H]4C=CC6=C7CC(C)(C)CC[C@]7(CO)[C@@H](O)C[C@@]56C)[C@]3(C)CO)O[C@@H]2CO[C@@H]8O[C@H](CO)[C@@H](O)[C@H](O)[C@H]8O)[C@H](O)[C@H](O)[C@H]1O |
| 1318 | CC1(C)CC[C@]2(CO)[C@@H](O)C[C@]3(C)C(=C2C1)C=C[C@@H]4[C@@]5(C)CC[C@H](O[C@@H]6O[C@H](CO[C@@H]7O[C@H](CO)[C@@H](O)[C@H](O)[C@H]7O[C@@H]8O[C@H](CO)[C@@H](O)[C@H](O)[C@H]8O)[C@@H](O)[C@H](O)[C@H]6O[C@@H]9O[C@H](CO)[C@@H](O)[C@H](O)[C@H]9O)[C@@](C)(CO)[C@@H]5CC[C@@]34C |
| 1319 | CC1(C)CC[C@]2(CO)[C@@H](O)C[C@]3(C)C(=C2C1)C=C[C@@H]4[C@@]5(C)CC[C@H](O[C@@H]6O[C@H](CO[C@@H]7O[C@H](CO)[C@@H](O)[C@H](O)[C@H]7O)[C@@H](O)[C@H](O)[C@H]6O[C@@H]8O[C@H](CO)[C@@H](O)[C@H](O)[C@H]8O)[C@@](C)(CO)[C@@H]5CC[C@@]34C |
| 1320 | C[C@@H]1O[C@@H](O[C@H]2[C@H](O)[C@@H](O)[C@H](O[C@H]3CC[C@@]4(C)[C@@H](CC[C@]5(C)[C@@H]4C=CC6=C7C[C@@](C)(CO)CC[C@]7(CO)[C@@H](O)C[C@@]56C)[C@@]3(C)CO)O[C@@H]2CO[C@@H]8O[C@H](CO)[C@@H](O)[C@H](O)[C@H]8O)[C@H](O)[C@H](O)[C@H]1O |
| 1321 | C[C@@H]1O[C@@H](O[C@H]2[C@H](O)[C@@H](O)[C@H](O[C@H]3CC[C@@]4(C)[C@H](CC[C@]5(C)[C@@H]4C=CC6=C7C[C@@](C)(CO)CC[C@]7(CO)[C@H](O)C[C@@]56C)[C@]3(C)CO)O[C@@H]2CO[C@@H]8O[C@H](CO)[C@@H](O)[C@H](O)[C@H]8O)[C@H](O)[C@H](O)[C@H]1O |
| 1322 | C[C@@H]1O[C@@H](O[C@H]2[C@H](O)[C@@H](O)[C@H](O[C@H]3CC[C@@]4(C)[C@@H](CC[C@]5(C)[C@@H]4C=CC6=C7C[C@](C)(CO)CC[C@]7(CO)[C@H](O)C[C@@]56C)[C@]3(C)CO)O[C@@H]2CO[C@@H]8O[C@H](CO)[C@@H](O)[C@H](O)[C@H]8O)[C@H](O)[C@H](O)[C@H]1O |
| 1323 | C[C@H]1O[C@@H](O[C@H]2CC[C@@]3(C)[C@@H](CC[C@]4(C)[C@@H]3C=CC5=C6C[C@](C)(CO)CC[C@]6(CO)[C@H](O)C[C@@]45C)[C@]2(C)CO)[C@H](O)[C@@H](O[C@@H]7O[C@H](CO)[C@@H](O)[C@H](O)[C@H]7O[C@@H]8O[C@H](CO)[C@@H](O)[C@H](O)[C@H]8O)[C@H]1O |
| 1324 | C[C@@H]1O[C@@H](O[C@H]2[C@H](O)[C@@H](O)[C@H](O[C@H]3CC[C@@]4(C)[C@@H](CC[C@]5(C)[C@@H]4C=CC6=C7CC(C)(C)CC[C@]7(CO)[C@H](O)C[C@@]56C)[C@]3(C)CO)O[C@@H]2CO[C@@H]8O[C@H](CO)[C@@H](O)[C@H](O)[C@H]8O)[C@H](O)[C@H](O)[C@H]1O |
| 1325 | CC1(C)CC[C@@]2(CC[C@]3(C)C(=CC[C@@H]4[C@@]5(C)CC[C@H](O[C@@H]6O[C@@H]([C@@H](O)[C@H](O[C@@H]7O[C@@H](CO)[C@H](O)[C@H]7O)[C@H]6O)C(=O)[O-])C(C)(C)[C@@H]5CC[C@@]34C)[C@H]2C1)C(=O)O[C@@H]8O[C@H](CO)[C@@H](O)[C@H](O)[C@H]8O |
| 1326 | CO[C@@H]1C=C2[C@@H]3CC(C)(C)CC[C@]3(CO)[C@@H](O)C[C@@]2(C)[C@]4(C)CC[C@H]5C(C)(C)[C@H](CC[C@]5(C)[C@@H]14)O[C@@H]6O[C@H](C)[C@H](O)[C@H](O)[C@H]6O[C@@H]7O[C@H](CO)[C@@H](O)[C@H](O)[C@H]7O |
| 1327 | C[C@H]1O[C@@H](O[C@H]2CC[C@@]3(C)[C@@H](CC[C@]4(C)[C@@H]3C=CC5=C6C[C@](C)(CC[C@]6(CO)[C@H](O)C[C@@]45C)C(=O)OC[C@H]7O[C@@H](OC[C@H](O)[C@H](O)[C@H](O)CO)[C@H](O)[C@@H](O)[C@@H]7O)[C@]2(C)CO)[C@H](O)[C@@H](O[C@@H]8O[C@H](CO)[C@@H](O)[C@H](O)[C@H]8O[C@@H]9O[C@H](CO)[C@@H](O)[C@H](O)[C@H]9O)[C@H]1O |
| 1328 | C[C@H]1O[C@@H](O[C@H]2CC[C@@]3(C)[C@@H](CC[C@]4(C)[C@@H]3C=CC5=C6C[C@](C)(CC[C@]6(CO)[C@H](O)C[C@@]45C)C(=O)OC[C@H]7O[C@@H](OC[C@H](O)[C@H](O)[C@H](O)CO)[C@H](O)[C@@H](O)[C@@H]7O)[C@]2(C)CO)[C@H](O)[C@@H](O[C@@H]8O[C@H](CO)[C@@H](O)[C@H](O)[C@H]8O)[C@H]1O |
| 1329 | C[C@H]1O[C@@H](O[C@H]2CC[C@@]3(C)[C@H](CC[C@]4(C)[C@@H]3C=CC5=C6C[C@](C)(CC[C@]6(CO)[C@H](O)C[C@@]45C)C(=O)OC[C@H](O)[C@H](O)[C@H](O)CO)[C@]2(C)CO)[C@H](O)[C@@H](O[C@@H]7O[C@H](CO)[C@@H](O)[C@H](O)[C@H]7O)[C@H]1O |
| 1330 | C[C@H]1O[C@@H](O[C@H]2CC[C@@]3(C)[C@@H](CC[C@]4(C)[C@@H]3C=CC5=C6C[C@](C)(CC[C@]6(CO)[C@@H](O)C[C@@]45C)C(=O)OC[C@H]7O[C@@H](OC[C@H](O)[C@H](O)[C@H](O)CO)[C@H](O)[C@@H](O)[C@@H]7O)[C@]2(C)CO)[C@H](O)[C@@H](O[C@@H]8O[C@H](CO)[C@@H](O)[C@H](O)[C@H]8O[C@@H]9O[C@H](CO)[C@@H](O)[C@H](O)[C@H]9O)[C@H]1O |
| 1331 | C[C@H]1O[C@@H](O[C@H]2CC[C@@]3(C)[C@H](CC[C@]4(C)[C@@H]3C=CC5=C6C[C@](C)(CC[C@]6(CO)[C@H](O)C[C@@]45C)C(=O)[O-])[C@]2(C)CO)[C@H](O)[C@@H](O[C@@H]7O[C@H](CO)[C@@H](O)[C@H](O)[C@H]7O)[C@H]1O |
| 1332 | C[C@H]1O[C@@H](O[C@H]2CC[C@@]3(C)[C@H](CC[C@]4(C)[C@@H]3C=C[C@]56OC[C@@]7(CC[C@@](C)(CO)C[C@H]57)[C@@H](O)C[C@@]46C)[C@]2(C)CO)[C@H](O[C@@H]8O[C@H](CO)[C@@H](O)[C@H](O)[C@H]8O)[C@@H](O[C@@H]9O[C@H](CO)[C@@H](OS(=O)(=O)[O-])[C@H](O)[C@H]9O)[C@H]1O |
| 1333 | C[C@H]1O[C@@H](O[C@H]2CC[C@@]3(C)[C@H](CC[C@]4(C)[C@@H]3C=C[C@]56OC[C@@]7(CC[C@@](C)(C[C@H]57)C(=O)[O-])[C@@H](O)C[C@@]46C)[C@]2(C)CO)[C@H](O[C@@H]8O[C@H](CO)[C@@H](O)[C@H](O)[C@H]8O)[C@@H](O[C@@H]9O[C@H](CO)[C@@H](OS(=O)(=O)[O-])[C@H](O)[C@H]9O)[C@H]1O |
| 1334 | C[C@H]1O[C@@H](O[C@H]2CC[C@@]3(C)[C@H](CC[C@]4(C)[C@@H]3C=C[C@]56OC[C@@]7(CC[C@@](CO)(C[C@H]57)C(=O)[O-])[C@@H](O)C[C@@]46C)[C@]2(C)CO)[C@H](O[C@@H]8O[C@H](CO)[C@@H](O)[C@H](O)[C@H]8O)[C@@H](O[C@@H]9O[C@H](CO)[C@@H](OS(=O)(=O)[O-])[C@H](O)[C@H]9O)[C@H]1O |
| 1335 | C[C@H]1O[C@@H](O[C@H]2CC[C@@]3(C)[C@H](CC[C@]4(C)[C@@H]3C=C[C@]56OC[C@@]7(CC[C@](C)(CO)C[C@H]57)[C@@H](O)C[C@@]46C)[C@]2(C)CO)[C@H](O[C@@H]8O[C@H](CO)[C@@H](O)[C@H](O)[C@H]8O)[C@@H](O[C@@H]9O[C@H](CO)[C@@H](OS(=O)(=O)[O-])[C@H](O)[C@H]9O)[C@H]1O |
| 1336 | C[C@H]1O[C@@H](O[C@H]2CC[C@@]3(C)[C@H](CC[C@]4(C)[C@@H]3C=CC5=C6C[C@](C)(CO)CC[C@@]6(CO)[C@H](O)C[C@@]45C)[C@]2(C)CO)[C@H](O[C@@H]7O[C@H](CO)[C@@H](O)[C@H](O)[C@H]7O)[C@@H](O[C@@H]8O[C@H](CO)[C@@H](OS(=O)(=O)[O-])[C@H](O)[C@H]8O)[C@H]1O |
| 1337 | COc1cc(O)c2C(=O)C=C(CO)Oc2c1 |
| 1338 | COc1cc2OC(=O)C=Cc2cc1OC |
| 1339 | C[C@@H]1O[C@@H](O[C@H]2CC[C@@]3(C)[C@@H](CC[C@]4(C)[C@@H]3C=CC5=C6C[C@](C)(CC[C@]6(CO)[C@H](O)C[C@@]45C)C(=O)OC[C@H](O)[C@H](O)[C@H](O)CO[C@@H]7O[C@H](CO)[C@@H](O)[C@H](O)[C@H]7O)[C@]2(C)CO)[C@H](O)[C@H](O[C@@H]8O[C@H](CO)[C@@H](O)[C@H](O)[C@H]8O)[C@H]1O |
| 1340 | C[C@@H]1O[C@@H](O[C@H]2CC[C@@]3(C)[C@@H](CC[C@]4(C)[C@@H]3C=CC5=C6C[C@](C)(CC[C@]6(CO)[C@H](O)C[C@@]45C)C(=O)OC[C@H](O)[C@H](O)[C@H](O)CO)[C@]2(C)CO)[C@H](O)[C@H](O[C@@H]7O[C@H](CO)[C@@H](O)[C@H](O)[C@H]7O)[C@H]1O |
| 1341 | C[C@@H]1O[C@@H](O[C@H]2CC[C@@]3(C)[C@@H](CC[C@]4(C)[C@@H]3C=CC5=C6C[C@](C)(CC[C@]6(CO)[C@H](O)C[C@@]45C)C(=O)OC[C@H](O)[C@H](O)[C@H](O)CO)[C@]2(C)CO)[C@H](O)[C@H](O)[C@H]1O |
| 1342 | CC[C@@H](\C=C\[C@@H](C)[C@H]1CC[C@H]2C3=CC[C@H]4C[C@@H](O)CC[C@]4(C)[C@H]3CC[C@]12C)C(C)C |
| 1343 | CC[C@H](\C=C\[C@@H](C)[C@H]1CC[C@H]2C3=CC[C@H]4C[C@H](CC[C@]4(C)[C@H]3CC[C@]12C)O[C@@H]5O[C@H](CO)[C@@H](O)[C@H](O)[C@H]5O)C(C)C |
| 1344 | CC(C)[C@@H]1NC(=O)[C@H](C)NC(=O)[C@@H](C)NC(=O)CNC(=O)CNC(=O)[C@H](Cc2ccc(O)cc2)NC1=O |
| 1345 | CC[C@H](\C=C\[C@@H](C)[C@H]1CC[C@H]2[C@@H]3CC=C4C[C@@H](O)CC[C@]4(C)[C@H]3CC[C@]12C)C(C)C |
| 1346 | CC[C@H](\C=C\[C@@H](C)[C@H]1CC[C@H]2[C@@H]3CC[C@H]4C[C@@H](O)CC[C@]4(C)[C@H]3CC[C@]12C)C(C)C |
| 1347 | CC(=C)C1CCC(C)(O)CC1 |
| 1348 | CC(=C)C1CCC(C)(O)CC1 |
| 1349 | C[C@H]1C[C@@H](O[C@H]2CC[C@@]3(C)[C@@H](CC[C@]4(C)[C@@H]3C=CC5=C6C[C@](C)(CO)CC[C@]6(CO)[C@H](O)C[C@@]45C)[C@]2(C)CO)[C@H](O)[C@@H](O[C@@H]7O[C@H](CO)[C@@H](O)[C@H](O)[C@H]7O)[C@H]1O |
| 1350 | C[C@@H]1O[C@@H](O[C@H]2[C@H](O)[C@@H](O)[C@H](O[C@H]3CC[C@@]4(C)[C@@H](CC[C@]5(C)[C@@H]4C=CC6=C7C[C@](C)(CO)CC[C@]7(CO)[C@H](O)C[C@@]56C)C3(C)C)O[C@@H]2CO[C@@H]8O[C@H](CO)[C@@H](O)[C@H](O)[C@H]8O)[C@H](O)[C@H](O)[C@H]1O |
| 1351 | C[C@H]1O[C@@H](O[C@H]2CC[C@@]3(C)[C@H](CC[C@]4(C)[C@@H]3C=CC5=C6C[C@](C)(CC[C@]6(CO)[C@H](O)C[C@@]45C)C(=O)[O-])[C@]2(C)CO)[C@H](O)[C@@H](O[C@@H]7O[C@H](CO)[C@@H](O)[C@H](O)[C@H]7O[C@@H]8O[C@H](CO)[C@@H](O)[C@H](O)[C@H]8O)[C@H]1O |
| 1352 | C[C@H]1O[C@@H](O[C@H]2CC[C@@]3(C)[C@@H](CC[C@]4(C)[C@@H]3[C@H](O)C=C5[C@@H]6CC(C)(C)CC[C@]6(CO)[C@@H](O)C[C@@]45C)[C@]2(C)CO)[C@H](O[C@@H]7O[C@H](CO)[C@@H](O)[C@H](O)[C@H]7O)[C@@H](O[C@@H]8O[C@H](CO)[C@@H](O)[C@H](O)[C@H]8O)[C@H]1O |
| 1353 | COc1ccc2Oc3cc(OC)c(OC)c(OC)c3C(=O)c2c1 |
| 1354 | C[C@H]1O[C@@H](O[C@H]2CC[C@@]3(C)[C@@H](CC[C@]4(C)[C@@H]3C=C[C@]56OC[C@@]7(CCC(C)(C)C[C@@H]57)[C@@H](O)C[C@@]46C)[C@]2(C)CO)[C@H](O)[C@@H](O[C@@H]8O[C@H](CO)[C@@H](O)[C@H](O)[C@H]8O[C@@H]9O[C@H](CO)[C@@H](O)[C@H](O)[C@H]9O)[C@H]1O |
| 1355 | CO[C@@H]1C=C2[C@@H]3CC(C)(C)CC[C@]3(CO)[C@H](O)C[C@@]2(C)[C@]4(C)CC[C@H]5[C@](C)(CO)[C@H](CC[C@]5(C)[C@@H]14)O[C@@H]6O[C@H](C)[C@H](O)[C@H](O[C@@H]7O[C@H](CO)[C@@H](O)[C@H](O)[C@H]7O)[C@H]6O[C@@H]8O[C@H](CO)[C@@H](O)[C@H](O)[C@H]8O |
| 1356 | C[C@@H]1O[C@@H](O[C@H]2[C@H](O)[C@@H](O)[C@H](O[C@H]3CC[C@@]4(C)[C@@H](CC[C@]5(C)[C@@H]4C=CC6=C7CC(C)(C)CC[C@]7(CO)[C@H](O)C[C@@]56C)C3(C)C)O[C@@H]2CO[C@@H]8O[C@H](CO)[C@@H](O)[C@H](O)[C@H]8O)[C@H](O)[C@H](O)[C@H]1O |
| 1357 | C[C@@H]1O[C@@H](O[C@H]2[C@H](O)[C@@H](O)[C@H](O[C@H]3CC[C@@]4(C)[C@@H](CC[C@]5(C)[C@@H]4C=CC6=C7CC(C)(C)CC[C@]7(CO[C@@H]8O[C@H](CO)[C@@H](O)[C@H](O)[C@H]8O)[C@H](O)C[C@@]56C)C3(C)C)O[C@@H]2CO[C@@H]9O[C@H](CO)[C@@H](O)[C@H](O)[C@H]9O)[C@H](O)[C@H](O)[C@H]1O |
| 1358 | COc1cc(cc(OC)c1OC)\C(=C/C)\OC(=O)\C(=C/C)\COC(=O)\C(=C\C)\C |
| 1359 | COc1cc(C=O)ccc1OC(=O)C |
| 1360 | COc1c(O)cc(O)c2C(=O)C=C(Oc12)c3ccccc3 |
| 1361 | C[C@H]1O[C@@H](O[C@H]2CC[C@@]3(C)[C@@H](CC[C@]4(C)[C@@H]3C=CC5=C6CC(C)(C)CC[C@]6(CO)[C@H](O)C[C@@]45C)[C@]2(C)CO)[C@H](O)[C@@H](O[C@@H]7O[C@H](CO)[C@@H](O)[C@H](O)[C@H]7O[C@@H]8OC[C@@H](O)[C@H](O)[C@H]8O)[C@H]1O |
| 1362 | CCCCCC[C@H]1CCC(=O)O1 |
| 1363 | CCC[C@H]1CCC(=O)O1 |
| 1364 | C[C@@H]1CC[C@@]23[C@H]1C[C@@H](CCC2=C)C3(C)C |
| 1365 | COc1ccc(C=O)c(O)c1 |
| 1366 | OCc1occc1 |
| 1367 | COc1cc(ccc1O)C2=C(O[C@@H]3O[C@H](CO)[C@@H](O)[C@H](O)[C@H]3O)C(=O)c4c(O)cc(O)cc4O2 |
| 1368 | COc1cc(ccc1[O-])C2=C(O[C@@H]3O[C@H](CO)[C@@H](O)[C@H](O)[C@H]3O)C(=O)c4c(O)cc(O)cc4O2 |
| 1369 | c1cc[nH]c1 |
| 1370 | CCCCCC(=O)c1occc1 |
| 1371 | CC(=O)c1occc1 |
| 1372 | CCCCCC(=O)C=C |
| 1373 | CCC\C=C\C=C\C=O |
| 1374 | CC(=CCC\C(=C/CCC(=O)C)\C)C |
| 1375 | c1ccncc1 |
| 1376 | OC[C@H]1O[C@H]([C@H](O)[C@@H](O)[C@@H]1O)c2c(O)ccc3C(=O)C(=COc23)c4ccc(O)cc4 |
| 1377 | OC[C@H]1O[C@H]([C@H](O)[C@@H](O)[C@@H]1O)c2c(O)ccc3C(=O)C(=COc23)c4ccc([O-])cc4 |
| 1378 | OC[C@H]1O[C@H]([C@H](O)[C@@H](O)[C@@H]1O)c2c([O-])ccc3C(=O)C(=COc23)c4ccc([O-])cc4 |
| 1379 | COc1c(O[C@@H]2O[C@@H]([C@@H](O)[C@H](O)[C@H]2O)C(=O)[O-])cc(O)c3C(=O)C=C(Oc13)c4ccccc4 |
| 1380 | O[C@H]1OC[C@H](Cc2ccc3OCOc3c2)[C@H]1Cc4ccc5OCOc5c4 |
| 1381 | Oc1ccc(cc1O)C2=C([O-])C(=O)c3c(O)cc([O-])cc3O2 |
| 1382 | Oc1cc(ccc1[O-])C2=C([O-])C(=O)c3c(O)cc([O-])cc3O2 |
| 1383 | CCCCCCC\C=C\C=O |
| 1384 | C[C@H]1CC[C@H](CC2=C1CC[C@@H]2C)C(=C)C |
| 1385 | CC(C)(O)C1CCC(=C)CC1 |
| 1386 | CC(=CCC\C(=C/CC\C(=C/CO)\C)\C)C |
| 1387 | CCCCCCCCCCC(=O)[O-] |
| 1388 | COc1cc(ccc1O)C2=C([O-])C(=O)c3c(O)cc([O-])cc3O2 |
| 1389 | COc1cc(ccc1[O-])C2=C([O-])C(=O)c3c(O)cc([O-])cc3O2 |
| 1390 | OC[C@@H](O)[C@@H](O)[C@H](O)C=O |
| 1391 | C[C@@H]1O[C@@H](OC[C@H]2O[C@@H](OC3=C(Oc4cc(O)cc(O)c4C3=O)c5ccc(O)c(O)c5)[C@H](O)[C@@H](O)[C@@H]2O)[C@H](O)[C@H](O)[C@H]1O |
| 1392 | OC[C@@H](O)[C@H](O)[C@@H](O)C=O |
| 1393 | CCCCC[C@@H](O)C=C |
| 1394 | COc1cc(cc(O)c1O)c2[o+]c3cc(O)cc(O)c3cc2O |
| 1395 | CC(=C)c1ccc(C)cc1 |
| 1396 | CC1(C)[C@@H]2CC=C(C=O)[C@H]1C2 |
| 1397 | COc1cc(C=O)ccc1O |
| 1398 | COc1cc(C=O)ccc1[O-] |
| 1399 | CCCCCC=O |
| 1400 | CC\C=C\C=C\C=O |
| 1401 | CCCC\C=C\C(=O)C |
| 1402 | CCCC\C=C\C=C\C=O |
| 1403 | CCCCC\C=C\C=C\C=O |
| 1404 | CC(=CCC\C(=C\CCC(=O)C)\C)C |
| 1405 | CC[C@@H](C)CCCC(C)C |
| 1406 | O=C1CCCCC1 |
| 1407 | CC(=CCC\C(=C\CC\C(=C\CCC(=O)C)\C)\C)C |
| 1408 | CCCCCCCC(=O)C |
| 1409 | C[C@@H]1CC[C@@H]2[C@H](C3=C1CC[C@H]3C)C2(C)C |
| 1410 | CC(C)c1ccc(C=O)cc1 |
| 1411 | CC(C)[C@@]12C[C@@H]1[C@@H](C)C=C2 |
| 1412 | CC1=CC[C@@H](CC=O)C1(C)C |
| 1413 | CC1=CC(=O)[C@@H]2C[C@H]1C2(C)C |
| 1414 | CC(=CCC\C(=C\CC[C@](C)(O)C=C)\C)C |
| 1415 | CC(=C)[C@H]1CC[C@](C)(C=C)[C@H](C1)C(=C)C |
| 1416 | CCCCCC\C=C\CCCCCCCC(=O)[O-] |
| 1417 | CCCCCCCCCCCC(=O)OC |
| 1418 | CCCC\C=C/C=O |
| 1419 | CC(C)CCC[C@@H](C)CCC[C@H](C)CCCC(=O)C |
| 1420 | CC(=O)c1ccccc1 |
| 1421 | CC(C)[C@@H]1CC[C@@]2(C)[C@@H]3CC=C(C)[C@H]2[C@H]13 |
| 1422 | C[C@@H]1CCC=C2CC[C@H](C[C@@]12C)C(=C)C |
| 1423 | CCCCC\C=C\C\C=C\CCCCCCCCOC(=O)C |
| 1424 | OC[C@H]1O[C@H](O)[C@H](O)[C@@H](O)[C@@H]1O |
| 1425 | Oc1ccc(\C=C\C(=O)c2ccc(O)cc2O)cc1 |
| 1426 | CCCCCCCCCCCCCCC(=O)OC |
| 1427 | CCOC(=O)c1ccccc1C(=O)OCC |
| 1428 | CCCCC(=O)c1ccccc1 |
| 1429 | CCC\C=C/1\OC(=O)c2ccccc12 |
| 1430 | C[C@@H]1CC[C@@H](C(=C)C)[C@@]12CCC(=CC2)C |
| 1431 | Cc1ccc(cc1)C(C)(C)O |
| 1432 | CC(=O)[C@H]1CC[C@H]2[C@@H]3CC=C4C[C@@H](O)CC[C@]4(C)[C@H]3CC[C@]12C |
| 1433 | CCCCCc1ccccc1 |
| 1434 | CCC\C=C/1\OC(=O)C2=C1CCC=C2 |
| 1435 | C[C@@H]1CC[C@@H]2[C@H]([C@H]3[C@@H]1CCC3=C)C2(C)C |
| 1436 | CC1(C)[C@@H]2CCC(=C)[C@H]3CC[C@@](C)(O)[C@H]3[C@H]12 |
| 1437 | CCCCC=O |
| 1438 | COc1cc(CNC(=O)CCCC\C=C\C(C)C)ccc1O |
| 1439 | CCCCCCCCCCCCCCCCCCCCCCCCCCCCCO |
| 1440 | COc1cc(ccc1O)C(=O)C |
| 1441 | COc1cc(ccc1[O-])C(=O)C |
| 1442 | Cc1ccc(O)cc1 |
| 1443 | COc1c(O)c2OC(=CC(=O)c2c(O)c1OC)c3ccccc3 |
| 1444 | CCCCCO |
| 1445 | C\C=C\C=C\C=O |
| 1446 | CC1(C)CC[C@]2(CO)CC=C3[C@]4(C)CC[C@@H]5C(C)(C)[C@@H](O)CC[C@]5(C)[C@H]4CC[C@@]3(C)[C@H]2C1 |
| 1447 | CCCCCCC\C=C/C=O |
| 1448 | c1ccc2ccccc2c1 |
| 1449 | CCCCCCCCCO |
| 1450 | CC(=O)c1cccc(C)c1 |
| 1451 | CC(C)[C@@H]1CC=C(C)c2ccc(C)cc12 |
| 1452 | C[C@@H]1CC[C@@H](C(=C)C)[C@]12CCC(=CC2)C |
| 1453 | Cc1occc1 |
| 1454 | COc1cc(ccc1O)C2=C(O[C@@H]3O[C@H](CO[C@@H]4O[C@@H](C)[C@H](O)[C@@H](O)[C@H]4O)[C@@H](O)[C@H](O)[C@H]3O)C(=O)c5c(O)cc(O)cc5O2 |
| 1455 | COc1cc(ccc1[O-])C2=C(O[C@@H]3O[C@H](CO[C@@H]4O[C@@H](C)[C@H](O)[C@@H](O)[C@H]4O)[C@@H](O)[C@H](O)[C@H]3O)C(=O)c5c(O)cc(O)cc5O2 |
| 1456 | C[C@@H]1CC[C@]23[C@@H](C)CC[C@H](C[C@@H]12)C3(C)C |
| 1457 | C[C@@H]1CCC2=C(CC[C@@H](C[C@H]12)C(=C)C)C=O |
| 1458 | O=Cc1cocc1 |
| 1459 | CCCCCCCCCCCCCCCCCC(=O)OCC(COC(=O)CCCCCCCCCCCCCCCCC)OC(=O)CCCCCCCCCCCCCCCCC |
| 1460 | OC[C@H]1O[C@@H](OC2=C(Oc3cc(O)cc(O)c3C2=O)c4ccc(O)c(O)c4)[C@H](O)[C@@H](O)[C@H]1O |
| 1461 | Cc1cccc2ccccc12 |
| 1462 | CO[C@H]1C=C2[C@H]3CCCC[C@@]3(CO)[C@@H](O)C[C@]2(C)[C@]4(C)CC[C@@H]5C(C)(C)[C@@H](CC[C@]5(C)[C@H]14)O[C@@H]6O[C@H](CO[C@@H]7O[C@H](CO)[C@@H](O)[C@@H](O)[C@H]7O)[C@@H](O[C@@H]8O[C@H](C)[C@@H](O)[C@@H](O)[C@H]8O)[C@H](O)[C@H]6O |
| 1463 | CO[C@H]1C=C2[C@H]3CCCC[C@@]3(CO)[C@@H](O)C[C@]2(C)[C@]4(C)CC[C@@H]5C(C)(C)[C@H](O)CC[C@]5(C)[C@H]14 |
| 1464 | CCCCCCC\C=C\C=C\C=O |
| 1465 | CCCC\C=C\C=C\CO |
| 1466 | C[C@@H]1CCCC1=O |
| 1467 | CCCCCC(C)C |
| 1468 | CCCCCCCCCCCCCCC(C)C |
| 1469 | C[C@H]1O[C@@H](O[C@H]2CC[C@@]3(C)[C@H](CC[C@]4(C)[C@H]3C=C[C@]56OC[C@@]7(CCC(C)(C)C[C@H]57)[C@H](O)C[C@@]46C)[C@]2(C)CO)[C@H](O)[C@@H](O[C@@H]8O[C@H](CO)[C@@H](O)[C@H](OC(=O)C)[C@H]8O)[C@H]1O |
| 1470 | CC1(C)CC[C@]23CO[C@@]4(C=C[C@H]5[C@@]6(C)CC[C@H](O)[C@@](C)(CO)[C@H]6CC[C@@]5(C)[C@]4(C)C[C@H]2O)[C@H]3C1 |
| 1471 | C[C@H]1O[C@@H](O[C@H]2CC[C@@]3(C)[C@@H](CC[C@]4(C)[C@H]3C=CC5=C6CC(C)(C)CC[C@@]6(CO)[C@H](O)C[C@@]45C)[C@]2(C)CO)[C@H](O)[C@@H](O[C@@H]7O[C@H](COC(=O)C)[C@@H](O)[C@H](OC(=O)C)[C@H]7O)[C@H]1O |
| 1472 | CC1(C)CC[C@@]2(CO)[C@H](O)C[C@]3(C)C(=C2C1)C=C[C@H]4[C@@]5(C)CC[C@H](O)[C@@](C)(CO)[C@@H]5CC[C@@]34C |
| 1473 | C[C@H]1O[C@@H](O[C@@H]2CC[C@@]3(C)[C@H](CC[C@]4(C)[C@H]3CC=C5[C@H]6CC(C)(C)CC[C@]6(CO)[C@@H](O)C[C@@]45C)[C@]2(C)CO)[C@H](O)[C@@H](O[C@@H]7O[C@H](CO)[C@@H](O)[C@H](OC(=O)C)[C@H]7O)[C@@H]1O |
| 1474 | COC1=C(Oc2cc(OC)c(OC)c(OC)c2C1=O)c3cc(OC)c(OC)c(OC)c3 |
| 1475 | CC[C@@H](C)CC(C)(C)CC |
| 1476 | COC1=C(Oc2cc(O)cc(O)c2C1=O)c3ccc(OC)c(O)c3 |
| 1477 | CC[C@@](C)(O)CCCC(C)C |
| 1478 | C[C@@H]1CCCCCCCCCCC(=O)C1 |
| 1479 | CC\C=C(/CC)\C(=C)C |
| 1480 | CCCCC(=O)CC |
| 1481 | CCCCCCCCC[C@H](C)CC |
| 1482 | CCCCC[C@H](C)CCC[C@H](C)CCC |
| 1483 | CCC[C@@H](C)[C@@H](O)CC |
| 1484 | CCCC[C@H]1CCC(=O)O1 |
| 1485 | COc1cc(C2=CC(=O)c3c(O)c(OC)c(OC)cc3O2)c(O)cc1O |
| 1486 | CCCCC[C@](C)(CC)CCCC |
| 1487 | CCCCC[C@H](C)CCCC |
| 1488 | CCC\C=C/1\OC(=O)C2=C1CC[C@@H]3[C@H](CCC)[C@]4(OC(=O)C5=C4CCC=C5)[C@H]23 |
| 1489 | COc1cc2C=CC(=O)Oc2c(OC)c1OC |
| 1490 | COc1cc(ccc1O)C(=O)O[C@@H]2C[C@](C)(O)[C@H]3[C@H](O[C@H]4O[C@H](CO)[C@H](O)[C@@H](O)[C@H]4O)OC=C[C@@H]23 |
| 1491 | COc1cc(ccc1[O-])C(=O)O[C@@H]2C[C@](C)(O)[C@H]3[C@H](O[C@H]4O[C@H](CO)[C@H](O)[C@@H](O)[C@H]4O)OC=C[C@@H]23 |
| 1492 | CCC[C@H](O)CCC=C |
| 1493 | COc1ccc2C=CC(=O)Oc2c1 |
| 1494 | CCCCCCC(C)CCCCCC |
| 1495 | [O-]C(=O)CCCCCCC=C |
| 1496 | Nc1ncnc2c1ncn2[C@@H]3O[C@@H](CO)[C@H](O)[C@H]3O |
| 1497 | OC[C@@H](O)C(O)[C@@H](O)CO |
| 1498 | COc1cc2OC(C)(C)C=Cc2cc1C(=O)C |
| 1499 | CC1(C)CCC[C@]23CO[C@](O)([C@@H](O)[C@H]12)[C@@]45[C@@H](O)[C@@H](CC[C@@H]34)C(=C)C5=O |
| 1500 | CC1(C)CC[C@]2(CO)[C@@H](O)C[C@]3(C)C(=CC[C@@H]4[C@@]5(C)CC[C@H](O[C@@H]6O[C@@H]([C@@H](O)[C@H](O)[C@H]6O)C(=O)[O-])C(C)(C)[C@H]5CC[C@@]34C)[C@H]2C1 |
| 1501 | CC1(C)CC[C@]2(CO)[C@@H](O)C[C@]3(C)C(=CC[C@@H]4[C@@]5(C)CC[C@H](O)C(C)(C)[C@@H]5CC[C@@]34C)[C@H]2C1 |
| 1502 | CCCCCC(=O)OC |
| 1503 | O[C@H]1CC[N@H+]2C[C@@H]3C[C@@H](CN4[C@H]3CCCC4=O)[C@H]2C1 |
| 1504 | [O-]C(=O)Cc1ccccc1 |
| 1505 | Oc1ccc(cc1)C2=COc3c(O)c(O)ccc3C2=O |
| 1506 | Oc1ccc2C(=O)C(=COc2c1O)c3ccc([O-])cc3 |
| 1507 | CC(=C)[C@@H]1CC[C@@]2(CC[C@]3(C)[C@H](CC[C@@H]4[C@@]5(C)CCC(=O)[C@@](C)(CO)[C@@H]5CC[C@@]34C)[C@@H]12)C(=O)O |
| 1508 | CC(=C)[C@@H]1CC[C@@]2(CC[C@]3(C)[C@H](CC[C@@H]4[C@@]5(C)CCC(=O)[C@@](C)(CO)[C@@H]5CC[C@@]34C)[C@@H]12)C(=O)[O-] |
| 1509 | C[C@H]1O[C@@H](O[C@H]2CC[C@]3(C)[C@@H](CC[C@]4(C)[C@@H]3C=CC5=C6CC(C)(C)CC[C@]6(CO)[C@H](O)C[C@@]45C)[C@]2(C)CO)[C@H](O)[C@@H](O[C@@H]7O[C@H](CO)[C@@H](O)[C@H](O)[C@H]7O)[C@H]1O |
| 1510 | C[C@H]1O[C@@H](O[C@@H]2CC[C@@]3(C)[C@@H](CC[C@]4(C)[C@@H]3C=C[C@]56OC[C@@]7(CCC(C)(C)C[C@@H]57)[C@H](O)C[C@@]46C)[C@]2(C)CO)[C@H](O)[C@@H](O[C@@H]8O[C@H](CO)[C@@H](O)[C@H](O)[C@H]8O)[C@H]1O |
| 1511 | C[C@H]1O[C@@H](O[C@H]2CC[C@]3(C)[C@@H](CC[C@]4(C)[C@@H]3C=CC5=C6CC(C)(C)CC[C@]6(CO)[C@@H](O)C[C@@]45C)[C@]2(C)CO)[C@H](O)[C@@H](O[C@@H]7O[C@H](CO)[C@@H](O)[C@H](O)[C@H]7O[C@H]8OC[C@@H](O)[C@H](O)[C@H]8O)[C@H]1O |
| 1512 | C[C@H]1O[C@@H](O[C@H]2CC[C@]3(C)[C@@H](CC[C@]4(C)[C@@H]3C=CC5=C6C[C@](C)(CC[C@]6(CO)[C@H](O)C[C@@]45C)C(=O)OC[C@H]7O[C@@H](OC[C@H](O)[C@@H](O)[C@H](O)CO)[C@H](O)[C@@H](O)[C@@H]7O)[C@]2(C)CO)[C@H](O)[C@@H](O[C@@H]8O[C@H](CO)[C@@H](O)[C@H](O)[C@H]8O)[C@H]1O |
| 1513 | C[C@@H]1O[C@@H](O[C@H]2[C@H](O)[C@@H](O)[C@H](O[C@H]3CC[C@@]4(C)[C@H](CC[C@]5(C)[C@@H]4CC=C6[C@@H]7CC(C)(C)CC[C@]7(CO)[C@@H](O)C[C@@]56C)C3(C)C)O[C@@H]2CO[C@@H]8O[C@H](CO)[C@@H](O)[C@H](O)[C@H]8O)[C@H](O)[C@H](O)[C@H]1O |
| 1514 | CC1(C)CC[C@]2(CO)[C@@H](O)C[C@]3(C)C(=CC[C@@H]4[C@@]5(C)CC[C@H](O)C(C)(C)[C@H]5CC[C@@]34C)[C@@H]2C1 |
| 1515 | CO[C@H]1C=C2[C@H]3CC(C)(C)CC[C@@]3(CO)[C@H](O)C[C@]2(C)[C@]4(C)CC[C@@H]5C(C)(C)[C@@H](CC[C@]5(C)[C@H]14)O[C@]6(CO)O[C@H](CO)[C@@H](O[C@@H]7O[C@H](CO)[C@@H](O)[C@@H](O)[C@H]7O)[C@H]6O |
| 1516 | CO[C@H]1C=C2[C@H]3CC(C)(C)CC[C@@]3(CO)[C@H](O)C[C@]2(C)[C@]4(C)CC[C@@H]5C(C)(C)[C@H](O)CC[C@]5(C)[C@H]14 |
| 1517 | COc1ccc(c(O)c1)c2oc3cc(OC)c(O)cc3c2 |
| 1518 | COc1ccc(c(O)c1)c2oc3cc(OC)c([O-])cc3c2 |
| 1519 | COc1cc(ccc1O)C2=CC(=O)c3c(O)c(O)c(OC)c(OC)c3O2 |
| 1520 | COc1cc(ccc1[O-])C2=CC(=O)c3c(O)c(O)c(OC)c(OC)c3O2 |
| 1521 | CCCCCCCCCCCCC(=O)[O-] |
| 1522 | C\C(=C\CCC(O)(O)O)\C=C\C=C(\C)/C=C/C1=C(C)CCCC1(C)C |
| 1523 | CCCCCCCC\C=C/C=O |
| 1524 | C\C=C(/C)\C(=O)O[C@H]1[C@@H](OC(=O)\C(=C\C)\C)C(C)(C)Oc2ccc3C=CC(=O)Oc3c12 |
| 1525 | CCCC\C=C\CO |
| 1526 | c1ccc(cc1)c2ccccc2 |
| 1527 | CC(C)[C@@H]1CC=C(C)C(=O)C1 |
| 1528 | CC1(C)C[C@H]2[C@@H]1CC[C@]3(C)O[C@@H]3CCC2=C |
| 1529 | C[C@@H]1CC[C@@]23C[C@@H]1C(C)(C)[C@H]2C=C[C@]3(C)O |
| 1530 | C[C@@H]1O[C@@H](O[C@H]2[C@H](O)[C@@H](O)[C@H](O[C@H]3CC[C@@]4(C)[C@H](CC[C@]5(C)[C@@H]4C=C[C@]67OC[C@@]8(CCC(C)(C)C[C@H]68)[C@@H](O)C[C@@]57C)[C@]3(C)CO)O[C@@H]2CO[C@@H]9O[C@H](CO)[C@@H](O)[C@H](O)[C@H]9O)[C@@H](O)[C@H](O)[C@H]1O |
| 1531 | CC1(C)CC[C@]23CO[C@@]4(C=C[C@@H]5[C@@]6(C)CC[C@H](O)[C@@](C)(CO)[C@H]6CC[C@@]5(C)[C@]4(C)C[C@@H]2O)[C@H]3C1 |
| 1532 | C[C@@H](CCOC(=O)C)CCC=C(C)C |
| 1533 | O=C=[NH+]C1CCCCC1 |
| 1534 | Cc1cccc(C=C)c1C |
| 1535 | CCOC(=O)\C=C(/C)\CCC=C(C)C |
| 1536 | CCOC(=O)c1ccc(O)c([O-])c1 |
| 1537 | COc1cc2C=CC(=O)Oc2c(O)c1O |
| 1538 | COc1cc2C=CC(=O)Oc2c(O)c1[O-] |
| 1539 | OC[C@H]1O[C@@H](O)[C@H](O)[C@@H](O)[C@H]1O |
| 1540 | C[C@@H]1[C@@H](O)C[C@H]2C[C@@H]1C2(C)C |
| 1541 | C[C@H]1O[C@H](OC2=C(Oc3cc(O[C@@H]4O[C@H](C)[C@@H](O)[C@@H](O)[C@H]4O)cc(O)c3C2=O)c5ccc(O)cc5)[C@H](O)[C@H](O)[C@@H]1O |
| 1542 | C[C@H]1O[C@H](OC2=C(Oc3cc(O[C@@H]4O[C@H](C)[C@@H](O)[C@@H](O)[C@H]4O)cc(O)c3C2=O)c5ccc([O-])cc5)[C@H](O)[C@H](O)[C@@H]1O |
| 1543 | C[C@@H]1O[C@H](OC2=C(Oc3cc(O[C@@H]4O[C@@H](C)[C@H](O)[C@@H](O)[C@H]4O)cc(O)c3C2=O)c5ccc(O)cc5)[C@H](O)[C@H](O)[C@H]1O |
| 1544 | C[C@@H]1O[C@H](OC2=C(Oc3cc(O[C@@H]4O[C@@H](C)[C@H](O)[C@@H](O)[C@H]4O)cc(O)c3C2=O)c5ccc([O-])cc5)[C@H](O)[C@H](O)[C@H]1O |
| 1545 | C[C@@H]1O[C@H](Oc2cc(O)c3C(=O)C(=C(Oc3c2)c4ccc(O)cc4)[O-])[C@@H](O)[C@H](O)[C@H]1O |
| 1546 | C[C@@H]1O[C@H](Oc2cc(O)c3C(=O)C(=C(Oc3c2)c4ccc([O-])cc4)[O-])[C@@H](O)[C@H](O)[C@H]1O |
| 1547 | C[C@H]1O[C@@H](Oc2cc(O)c3C(=O)C(=C(Oc3c2)c4ccc(O)cc4)[O-])[C@H](O)[C@@H](O)[C@@H]1O |
| 1548 | C[C@H]1O[C@@H](Oc2cc(O)c3C(=O)C(=C(Oc3c2)c4ccc([O-])cc4)[O-])[C@H](O)[C@@H](O)[C@@H]1O |
| 1549 | C[C@H]1CC[C@H]2[C@@H]1[C@H]3[C@@H](CC[C@]2(C)O)C3(C)C |
| 1550 | COc1cc(OC)c2C=CC(=O)Oc2c1 |
| 1551 | CC(C)[C@H]1CC[C@@](C)(O)C=C1 |
| 1552 | CCCCC[C@H]1CC=CC(=O)O1 |
| 1553 | CCCCCCCCCCCCC\C=C\C=C\C(=O)OC |
| 1554 | CCCCCCCCCCCCCCC\C=C\C(=O)OC |
| 1555 | CCCCCC(=O)\C=C\C |
| 1556 | CCCCC\C=C\C(=O)C |
| 1557 | CCCCCCC(=O)C |
| 1558 | CC(C)c1ccc(cc1)C(=O)[O-] |
| 1559 | C[C@H](O)[C@H](O)[C@@H](O)[C@@H](O)C=O |
| 1560 | OC[C@@H](O)[C@@H](O)[C@@H](O)C=O |
| 1561 | C[C@H]1O[C@@H](O[C@H]2CC[C@@]3(C)[C@@H](CC[C@]4(C)[C@H]3C=CC5=C6CC(C)(C)CC[C@]6(CO)[C@@H](O)C[C@@]45C)[C@]2(C)CO)[C@H](O)[C@@H](O[C@@H]7O[C@H](CO)[C@@H](O)[C@H](O)[C@H]7O)[C@H]1O |
| 1562 | CC1(C)CC[C@]2(CO)[C@@H](O)C[C@]3(C)C(=C2C1)C=C[C@H]4[C@@]5(C)CC[C@H](O)[C@@](C)(CO)[C@@H]5CC[C@@]34C |
| 1563 | C[C@H]1O[C@@H](O[C@H]2CC[C@@]3(C)[C@@H](CC[C@]4(C)[C@H]3C=CC5=C6CC(C)(C)CC[C@]6(CO)[C@H](O)C[C@]45C)[C@]2(C)CO)[C@H](O)[C@@H](O[C@@H]7O[C@H](CO)[C@@H](O)[C@H](O)[C@@H]7O)[C@H]1O |
| 1564 | CC1(C)CC[C@]2(CO)[C@H](O)C[C@@]3(C)C(=C2C1)C=C[C@H]4[C@@]5(C)CC[C@H](O)[C@@](C)(CO)[C@@H]5CC[C@@]34C |
| 1565 | CO[C@@H]1C=C2[C@H]3CC(C)(C)CC[C@]3(CO)[C@@H](O)C[C@@]2(C)[C@]4(C)CC[C@@H]5[C@](C)(CO)[C@H](CC[C@]5(C)[C@@H]14)O[C@@H]6O[C@H](C)[C@H](O)[C@H](O[C@@H]7O[C@H](CO)[C@@H](O)[C@H](O)[C@H]7O)[C@H]6O |
| 1566 | CO[C@@H]1C=C2[C@H]3CC(C)(C)CC[C@]3(CO)[C@@H](O)C[C@@]2(C)[C@]4(C)CC[C@@H]5[C@](C)(CO)[C@@H](O)CC[C@]5(C)[C@@H]14 |
| 1567 | CO[C@@H]1C=C2[C@H]3CC(C)(C)CC[C@]3(CO)[C@H](O)C[C@@]2(C)[C@]4(C)CC[C@@H]5[C@](C)(CO)[C@H](CC[C@]5(C)[C@@H]14)O[C@@H]6O[C@H](C)[C@H](O)[C@H](O[C@@H]7O[C@H](CO)[C@@H](O)[C@H](O)[C@H]7O)[C@H]6O |
| 1568 | CO[C@@H]1C=C2[C@H]3CC(C)(C)CC[C@]3(CO)[C@H](O)C[C@@]2(C)[C@]4(C)CC[C@@H]5[C@](C)(CO)[C@@H](O)CC[C@]5(C)[C@@H]14 |
| 1569 | C[C@@H]1O[C@H](O[C@H]2[C@H](O)[C@@H](O)[C@H](O[C@H]3CC[C@@]4(C)[C@@H](CC[C@]5(C)[C@H]4C=C[C@]67OC[C@@]8(CCC(C)(C)C[C@H]68)[C@@H](O)C[C@@]57C)[C@]3(C)CO)O[C@@H]2CO[C@@H]9O[C@H](CO)[C@@H](O)[C@H](O)[C@H]9O)[C@H](O)[C@@H](O)[C@H]1O |
| 1570 | CC1(C)CC[C@]23CO[C@@]4(C=C[C@H]5[C@@]6(C)CC[C@H](O)[C@@](C)(CO)[C@@H]6CC[C@@]5(C)[C@]4(C)C[C@@H]2O)[C@H]3C1 |
| 1571 | C[C@H]1O[C@@H](O[C@H]2CC[C@@]3(C)[C@H](CC[C@]4(C)[C@@H]3C=C[C@]56OC[C@@]7(CCC(C)(C)C[C@H]57)[C@@H](O)C[C@@]46C)C2(C)C)[C@H](O)[C@@H](O[C@@H]8O[C@H](CO)[C@@H](O)[C@H](O)[C@H]8O)[C@H]1O |
| 1572 | CC1(C)CC[C@]23CO[C@@]4(C=C[C@@H]5[C@@]6(C)CC[C@H](O)C(C)(C)[C@@H]6CC[C@@]5(C)[C@]4(C)C[C@@H]2O)[C@H]3C1 |
| 1573 | CCC\C=C/1\OC(=O)C2=C1CC[C@H](O)[C@@H]2O |
| 1574 | CC1(C)[C@@H]2CCC(=C)[C@H]3CC[C@](C)(O)[C@H]3[C@H]12 |
| 1575 | CC(C)[C@@H]1CC[C@](C)(O)[C@@H]2CCC(=C[C@H]12)C |
| 1576 | CC(C)[C@H]1CC[C@@](C)(O)[C@@H]2CCC(=C[C@H]12)C |
| 1577 | CC(=C)[C@H]1CC=C(C)[C@H](O)C1 |
| 1578 | [NH3+][C@H](Cc1c[nH]c2ccccc12)C(=O)[O-] |
| 1579 | COc1ccc(CO)cc1OC |
| 1580 | CC1=CCC[C@]2(C)CC[C@H](C[C@@H]12)C(C)(C)O |
| 1581 | CC1(C)[C@H]2CC[C@@H]1C(=C)C2 |
| 1582 | CC1=C[C@H]2[C@H](CC1)C(=C)CCCC2(C)C |
| 1583 | CC1=C(C)[C@H]2CC[C@@H]1C2 |
| 1584 | CC(=C)[C@@H]1CC[C@@]2(C)CCC=C(C)[C@@H]2C1 |
| 1585 | CC[C@@H](\C=C\[C@@H](C)[C@H]1CC[C@H]2C3=CC[C@@H]4C[C@@H](O)CC[C@]4(C)[C@H]3CC[C@]12C)C(C)C |
| 1586 | CC[C@H](\C=C\[C@@H](C)[C@H]1CC[C@H]2C3=CC[C@@H]4C[C@H](CC[C@]4(C)[C@H]3CC[C@]12C)O[C@@H]5O[C@H](CO)[C@@H](O)[C@H](O)[C@H]5O)C(C)C |
| 1587 | C[C@@H]1CC[C@@H]2C(C)(C)[C@H]3C[C@@]12CCC3=C |
| 1588 | C[C@@]12C[C@@H](C=C1)C(C)(C)C2 |
| 1589 | CC(C)[C@@H]1CCC(=C)[C@@H]2CC[C@@H]([C@H]12)C(=O)C |
| 1590 | CC(C)[C@@H]1CCC(=C)[C@H]2CCC(=C[C@H]12)C |
| 1591 | COc1cc(OC)c(\C=C\C)cc1OC |
| 1592 | COc1cc(OC)c(\C=C/C)cc1OC |
| 1593 | COc1cc(OC)c(cc1OC)[C@H]2O[C@@H]2C |
| 1594 | CCCCCC\C=C/CCCCCCCCCC1=CC(=O)C=C(OC)C1=O |
| 1595 | CCC(=O)c1cc(OC)c(OC)cc1OC |
| 1596 | CCCCCC\C=C\CCCCCCCCCC1=CC(=O)C=C(OC)C1=O |
| 1597 | OC[C@H]1O[C@@H](Oc2cc3c(O[C@@H]4O[C@H](CO)[C@@H](O)[C@H](O)[C@H]4O)cc(O)cc3[o+]c2c5ccc(O)cc5)[C@H](O)[C@@H](O)[C@@H]1O |
| 1598 | OC[C@H]1O[C@@H](Oc2cc3c(O[C@@H]4O[C@H](CO)[C@@H](O)[C@H](O)[C@H]4O)cc(O)cc3[o+]c2c5ccc([O-])cc5)[C@H](O)[C@@H](O)[C@@H]1O |
| 1599 | C[C@@H]1O[C@@H](O[C@H]2[C@H](Oc3cc(O)c4C(=O)C=C(Oc4c3)c5ccc(O)cc5)O[C@H](CO)[C@@H](O)[C@@H]2O)[C@H](O)[C@H](O)[C@H]1O |
| 1600 | C[C@@H]1O[C@@H](O[C@H]2[C@H](Oc3cc(O)c4C(=O)C=C(Oc4c3)c5ccc([O-])cc5)O[C@H](CO)[C@@H](O)[C@@H]2O)[C@H](O)[C@H](O)[C@H]1O |
| 1601 | CC(=CCC\C(=C\CC[C@@](C)(O)C=C)\C)C |
| 1602 | CC(=CCC[C@@](C)(O)[C@@H]1CCC(=CC1)C)C |
| 1603 | C[C@H]1CC[C@H](CC2=C1CC[C@@H]2C)C(C)(C)O |
| 1604 | COc1cc(\C=C\C)cc(OC)c1OC |
| 1605 | COc1cc(CC=C)cc(OC)c1OC |
| 1606 | COc1ccc(cc1OC)[C@H]2O[C@H]([C@@H](C)[C@@H]2C)c3ccc(OC)c(OC)c3 |
| 1607 | CC(=C)[C@@H]1CC[C@]2(C)CC[C@]3(C)[C@H](CC[C@@H]4[C@@]5(C)CC[C@H](O)C(C)(C)[C@@H]5CC[C@@]34C)[C@@H]12 |
| 1608 | CC1(C)C[C@H]2CC[C@]1(C)OC2 |
| 1609 | CC(=O)\C=C\[C@H]1C(=CCCC1(C)C)C |
| 1610 | C[C@H]1CCC=C2CC[C@H]3[C@H](C3(C)C)[C@]12C |
| 1611 | CC1=C[C@@H]2[C@H](CC1)[C@](C)(O)CCC2(C)C |
| 1612 | COc1ccc(\C=C\C(=O)[O-])cc1 |
| 1613 | CC(C)(O)[C@@H]1Cc2cc3C=CC(=O)Oc3cc2O1 |
| 1614 | CC(C)[C@@H]1CC\C(=C\CC\C(=C/CC[C@](C)(O)\C=C\1)\C)\C |
| 1615 | COc1ccc(CC=C)cc1 |
| 1616 | C[C@@H]1O[C@@H](OC[C@H]2O[C@@H](OC3=C(Oc4cc(O)cc(O)c4C3=O)c5ccc(O)cc5)[C@H](O)[C@@H](O)[C@@H]2O)[C@H](O)[C@H](O)[C@H]1O |
| 1617 | C[C@@H]1O[C@@H](OC[C@H]2O[C@@H](OC3=C(Oc4cc(O)cc(O)c4C3=O)c5ccc([O-])cc5)[C@H](O)[C@@H](O)[C@@H]2O)[C@H](O)[C@H](O)[C@H]1O |
| 1618 | COc1cc(O)ccc1C(=O)\C=C\c2ccc(O)cc2 |
| 1619 | COc1cc([O-])ccc1C(=O)\C=C\c2ccc(O)cc2 |
| 1620 | O=C(OCc1ccccc1)c2ccccc2 |
| 1621 | COC1=CC(=O)C(=CC1=O)OC |
| 1622 | COc1ccc(\C=C\C)cc1OC |
| 1623 | CC(C)[C@@H]1CC=C(C)[C@@H]2CC=C(C)C[C@@H]12 |
| 1624 | C[C@@H]1CCC2=C(C)CC[C@@H]3[C@H]([C@H]12)C3(C)C |
| 1625 | COC(=O)\C=C\C1=C(C)[C@@H](O)CCC1(C)C |
| 1626 | CC1(C)[C@H]2CC[C@]1(CS(=O)(=O)[O-])C(=O)C2 |
| 1627 | CC(=CCc1c(O)cc(O)c2C(=O)C(=C(Oc12)c3ccc(O)cc3)[O-])C |
| 1628 | CC(=CCc1c(O)cc(O)c2C(=O)C(=C(Oc12)c3ccc([O-])cc3)[O-])C |
| 1629 | Nc1c2ccccc2[nH+]c3ccccc13 |
| 1630 | Nc1c2ccccc2nc3ccccc13 |
| 1631 | COc1cc(OC)c(OC)cc1CC(=O)C |
| 1632 | C[C@@H]1CCCC2=CC[C@@H]3[C@@H](C3(C)C)[C@@]12C |
| 1633 | COC1=C[C@@]2(CC=C)[C@H]([C@H]3C(=C[C@]2(OC)C(=O)C3(OC)OC)CC=C)C(OC)(OC)C1=O |
| 1634 | COc1cc(OC)c(OC)cc1CC=C |
| 1635 | ClCc1ccccc1 |
| 1636 | COc1cc(OC)c(cc1OC)[C@H]2[C@H](C)[C@@H](C)[C@@H]2c3cc(OC)c(OC)cc3OC |
| 1637 | BrCc1ccccc1 |
| 1638 | CC(C)C1=CC[C@](C)(O)[C@@H]2CCC(=C)C[C@]12O |
| 1639 | COc1cc(OC)c(C=O)cc1OC |
| 1640 | COc1ccc(\C=C/C)cc1OC |
| 1641 | CC(C)[C@@H]1CCC(=C2CCC(=C[C@H]12)C)C |
| 1642 | C[C@@H]1CCC=C2CC[C@H](C[C@]12C)C(=C)C |
| 1643 | COc1cc(OC)c(cc1OC)C(=O)[O-] |
| 1644 | CC(C)C1=CC[C@@]2(C)CC[C@H](C)C[C@@H]2C1=O |
| 1645 | CC(C)[C@@H]1CC[C@@](C)(O)[C@H]2CCC(=C)C[C@@]12O |
| 1646 | CC(C)[C@@H]1CC[C@@](C)(C=C)C(=C(C)C)C1=O |
| 1647 | Cc1ccc(\C=C\C(=O)[O-])c(C)c1 |
| 1648 | CC(C)[C@@H]1C=C[C@@H](C)[C@@H]2CCC(=C)[C@H](OO)[C@@H]12 |
| 1649 | CC[NH+](CC)Cc1ccccc1 |
| 1650 | C[C@@H]1O[C@@H](Oc2cc(O)c3C(=O)C(=C(Oc3c2O)c4ccc(O)cc4)[O-])[C@H](O)[C@H](O)[C@H]1O |
| 1651 | C[C@@H]1O[C@@H](Oc2cc(O)c3C(=O)C(=C(Oc3c2O)c4ccc([O-])cc4)[O-])[C@H](O)[C@H](O)[C@H]1O |
| 1652 | CC(C)[C@@H]1CC[C@@](C)(C=C)[C@@H](C(=C)C)C1=O |
| 1653 | CC1(C)[C@@H]2CCC(=C)[C@@H]3CC[C@@](C)(O)[C@H]3[C@H]12 |
| 1654 | CC(=CCC[C@@](C)(O)[C@H]1CCC(=CC1)C)C |
| 1655 | C[C@@H]1CC[C@@H]2[C@H](C3=C(C)CC[C@@H]13)C2(C)C |
| 1656 | CC1=CCC[C@@]2(C)CC[C@@H]3C(C)(C)C[C@]123 |
| 1657 | COc1ccc(cc1OC)[C@@H]2OC[C@@H]3[C@H]2CO[C@H]3c4ccc(OC)c(OC)c4 |
| 1658 | COc1cc(OC)c(cc1OC)[C@@H]2[C@H](C)[C@H](C)[C@@H]2c3cc(OC)c(OC)cc3OC |
| 1659 | COc1ccccc1 |
| 1660 | OC[C@H]1O[C@H]([C@H](O)[C@@H](O)[C@@H]1O)c2c(O)c([C@@H]3OC[C@H](O)[C@H](O)[C@H]3O)c(O)c4C(=O)C=C(Oc24)c5ccc(O)cc5 |
| 1661 | OC[C@H]1O[C@H]([C@H](O)[C@@H](O)[C@@H]1O)c2c(O)c([C@@H]3OC[C@H](O)[C@H](O)[C@H]3O)c(O)c4C(=O)C=C(Oc24)c5ccc([O-])cc5 |
| 1662 | OC[C@@H](O)[C@@H](O)[C@H](O)[C@H](O)CO |
| 1663 | CC[C@@H](CC[C@@H](C)[C@H]1CC[C@H]2[C@@H]3CC[C@@H]4C[C@@H](O)CC[C@]4(C)[C@H]3CC[C@]12C)C(C)C |
| 1664 | CC[C@H](CC[C@@H](C)[C@H]1CC[C@@H]2[C@@H]3CC[C@H]4C[C@H](O)CC[C@]4(C)[C@H]3CC[C@]12C)C(C)C |
| 1665 | O[C@H]1[C@H](O)[C@@H](COC(=O)\C=C\c2ccc(O)c(O)c2)O[C@@H](OCCc3ccc(O)c(O)c3)[C@@H]1O |
| 1666 | C[C@H]1O[C@H](O[C@H]2[C@H](O)[C@@H](COC(=O)\C=C\c3ccc(O)c(O)c3)O[C@@H](OCCc4ccc(O)c(O)c4)[C@@H]2O)[C@H](O)[C@H](O)[C@@H]1O |
| 1667 | O[C@@H]1[C@H](O)[C@@H](COC(=O)\C=C\c2ccc(O)c(O)c2)O[C@@H](OCCc3ccc(O)c(O)c3)[C@@H]1O |
| 1668 | C[C@H]1O[C@H](O[C@H]2[C@H](O)[C@@H](COC(=O)\C=C\c3ccc(O)c(O)c3)O[C@@H](OCCc4ccc(O)c(O)c4)[C@@H]2O)[C@@H](O)[C@@H](O)[C@@H]1O |
| 1669 | CC1(C)CC[C@@]2(CC[C@]3(C)C(=CC[C@@H]4[C@@]5(C)CC[C@@H](O)C(C)(C)[C@@H]5CC[C@@]34C)[C@@H]2C1)C(=O)O |
| 1670 | CC1(C)CC[C@@]2(CC[C@]3(C)C(=CC[C@@H]4[C@@]5(C)CC[C@@H](O)C(C)(C)[C@@H]5CC[C@@]34C)[C@@H]2C1)C(=O)[O-] |
| 1671 | OC[C@H]1O[C@@H](O[C@@H]2OC=C([C@H]3CC=C(CO)[C@@H]23)C(=O)[O-])[C@H](O)[C@@H](O)[C@@H]1O |
| 1672 | OCC1=CC[C@H]2[C@@H]1[C@H](O)OC=C2C(=O)[O-] |
| 1673 | COc1c(O)cc2OC(=CC(=O)c2c1O)c3ccc(O)cc3 |
| 1674 | COc1c(O)cc2OC(=CC(=O)c2c1O)c3ccc([O-])cc3 |
| 1675 | OC[C@H]1O[C@@H](O[C@@H]2OC=C[C@H]3[C@H](O)[C@@H]4O[C@]4(CO)[C@@H]23)[C@H](O)[C@@H](O)[C@@H]1O |
| 1676 | OC[C@H]1O[C@@H](O[C@@H]2OC=C[C@H]3[C@H](O)C=C(CO)[C@@H]23)[C@H](O)[C@@H](O)[C@@H]1O |
| 1677 | COC(=O)C1=CO[C@@H](O[C@@H]2O[C@H](CO)[C@@H](O)[C@H](O)[C@H]2O)[C@H]3[C@@H]1CC=C3CO |
| 1678 | CC[C@@H](CC[C@@H](C)[C@@H]1CC[C@H]2[C@H]3CC=C4C[C@H](CC[C@]4(C)[C@@H]3CC[C@@]12C)O[C@H]5O[C@@H](CO)[C@H](O)[C@@H](O)[C@@H]5O)C(C)C |
| 1679 | CC[C@@H](CC[C@@H](C)[C@@H]1CC[C@H]2[C@H]3CC=C4C[C@@H](O)CC[C@]4(C)[C@@H]3CC[C@@]12C)C(C)C |
| 1680 | OCC1=C[C@@H](O)[C@@H]2C=CO[C@@H](O)[C@H]12 |
| 1681 | OC[C@H]1O[C@@H](OCCc2ccc(O)c(O)c2)[C@H](O)[C@@H](O)[C@@H]1OC(=O)\C=C\c3ccc(O)c(O)c3 |
| 1682 | OC[C@H]1O[C@@H](Oc2cc3OC(=CC(=O)c3c(O)c2O)c4ccc(O)cc4)[C@H](O)[C@@H](O)[C@@H]1O |
| 1683 | OC[C@H]1O[C@@H](Oc2cc3OC(=CC(=O)c3c(O)c2O)c4ccc([O-])cc4)[C@H](O)[C@@H](O)[C@@H]1O |
| 1684 | OC[C@@H]1O[C@H](O[C@@H]2[C@@H](O)[C@H](OCCc3ccc(O)c(O)c3)O[C@H](CO)[C@H]2OC(=O)\C=C\c4ccc(O)c(O)c4)[C@@H](O)[C@H](O)[C@H]1O |
| 1685 | COC(=O)C1=CO[C@H](O[C@H]2O[C@@H](CO)[C@H](O)[C@@H](O)[C@@H]2O)C3=C(C)[C@H](O)C[C@@H]13 |
| 1686 | COC(=O)C1=CO[C@H](O)C2=C(C)[C@H](O)C[C@@H]12 |
| 1687 | COc1ccc(CCO[C@@H]2O[C@H](CO)[C@@H](OC(=O)\C=C\c3ccc(O)c(OC)c3)[C@H](O[C@@H]4O[C@@H](C)[C@H](O)[C@@H](O)[C@H]4O)[C@H]2O)cc1O |
| 1688 | COc1c(O)c2C(=O)C=C(Oc2cc1O[C@@H]3O[C@H](CO)[C@@H](O)[C@H](O)[C@H]3O)c4ccc(O)cc4 |
| 1689 | COc1c(O)c2C(=O)C=C(Oc2cc1O[C@@H]3O[C@H](CO)[C@@H](O)[C@H](O)[C@H]3O)c4ccc([O-])cc4 |
| 1690 | OC[C@@H]1O[C@@H](O[C@H]2[C@H](O)[C@H](OC[C@H](O)c3ccc(O)c(O)c3)O[C@H](CO)[C@H]2OC(=O)\C=C\c4ccc(O)c(O)c4)[C@@H](O)[C@@H](O)[C@H]1O |
| 1691 | OC[C@H]1O[C@@H](OC[C@H](O)c2ccc(O)c(O)c2)[C@@H](O)[C@H](O)[C@@H]1OC(=O)\C=C\c3ccc(O)c(O)c3 |
| 1692 | OC[C@H]1O[C@@H](Oc2cc(cc(O)c2O)[C@@H]3CC(=O)c4c(O)cc(O)cc4O3)[C@H](O)[C@@H](O)[C@@H]1O |
| 1693 | OC[C@H]1O[C@@H](Oc2cc(cc([O-])c2O)[C@@H]3CC(=O)c4c(O)cc(O)cc4O3)[C@H](O)[C@@H](O)[C@@H]1O |
| 1694 | Oc1cc(O)c2C(=O)C[C@H](Oc2c1)c3cc(O)c(O)c(O)c3 |
| 1695 | Oc1cc(O)c2C(=O)C[C@H](Oc2c1)c3cc(O)c(O)c([O-])c3 |
| 1696 | OC[C@@H]1O[C@@H](O[C@H]2[C@@H](O)[C@@H](COC(=O)\C=C\c3ccc(O)c(O)c3)O[C@H](OCCc4ccc(O)c(O)c4)[C@@H]2O)[C@@H](O)[C@H](O)[C@H]1O |
| 1697 | O[C@H]1[C@@H](O)[C@@H](COC(=O)\C=C/c2ccc(O)c(O)c2)O[C@H](OCCc3ccc(O)c(O)c3)[C@@H]1O |
| 1698 | O[C@@H]1[C@@H](COC(=O)\C=C\c2ccc(O)c(O)c2)O[C@H]3OC[C@@H](O[C@@H]3[C@H]1O)c4ccc(O)c(O)c4 |
| 1699 | OC[C@H]1O[C@@H](Oc2c([O-])cc(O)c3C(=O)C=C(Oc23)c4ccc(O)c(O)c4)[C@H](O)[C@@H](O)[C@@H]1O |
| 1700 | Oc1ccc(cc1O)C2=CC(=O)c3c(O)cc([O-])c(O)c3O2 |
| 1701 | CC1(C)CC[C@@]2(CC[C@]3(C)C(=CC[C@H]4[C@@]3(C)CC[C@H]5C(C)(C)[C@]6(O)CC[C@]45CO6)[C@@H]2C1)C(=O)O |
| 1702 | CC1(C)CC[C@@]2(CC[C@]3(C)C(=CC[C@H]4[C@@]3(C)CC[C@H]5C(C)(C)[C@]6(O)CC[C@]45CO6)[C@@H]2C1)C(=O)[O-] |
| 1703 | OC[C@@]12O[C@@H]1[C@@H](O)[C@@H]3C=CO[C@@H](O)[C@H]23 |
| 1704 | C[C@@H]1O[C@H](OC[C@H]2C[C@@H](OCCc3ccc(O)c(O)c3)[C@H](O)[C@@H](O[C@H]4O[C@@H](CO)[C@H](O)[C@@H](O)[C@@H]4O)[C@@H]2OC(=O)\C=C\c5ccc(O)c(O)c5)[C@@H](O)[C@H](O)[C@H]1O |
| 1705 | OC[C@H]1C[C@@H](OCCc2ccc(O)c(O)c2)[C@H](O)[C@@H](O)[C@@H]1OC(=O)\C=C\c3ccc(O)c(O)c3 |
| 1706 | COc1ccc(\C=C\C(=O)O[C@H]2[C@H](CO)O[C@H](OCCc3ccc(O)c(OC)c3)[C@H](O)[C@H]2O)cc1O |
| 1707 | CC(C)C(C)(C)CC[C@H](C)[C@H]1CC[C@@H]2[C@@H]3CC[C@H]4C[C@@H](O)CC[C@]4(C)[C@H]3CC[C@]12C |
| 1708 | OC[C@H]1O[C@@H](O[C@@H]2OCC[C@H]3[C@H](O)C=C(CO)[C@@H]23)[C@H](O)[C@@H](O)[C@@H]1O |
| 1709 | OCC1=C[C@@H](O)[C@@H]2CCO[C@@H](O)[C@H]12 |
| 1710 | C[C@@H]1O[C@H](O[C@H]2[C@@H](OC(=O)\C=C\c3ccc(O)c(O)c3)[C@H](CO)O[C@@H]4OC[C@H](O[C@H]24)c5ccc(O)c(O)c5)[C@@H](O)[C@H](O)[C@H]1O |
| 1711 | OC[C@@H]1O[C@@H]2OC[C@H](O[C@@H]2[C@@H](O)[C@@H]1OC(=O)\C=C/c3ccc(O)c(O)c3)c4ccc(O)c(O)c4 |
| 1712 | COC(=O)C1=CO[C@H](O)[C@@H]2[C@H]1CC=C2CO |
| 1713 | C[C@@H]1O[C@@H](O[C@H]2[C@H](OC(=O)\C=C\c3ccc(O)c(O)c3)[C@@H](CO)O[C@H]4OC[C@H](O[C@H]24)c5ccc(O)c(O)c5)[C@H](O)[C@H](O)[C@H]1O |
| 1714 | C[C@@H]1O[C@@H](O[C@H]2[C@H](OC(=O)\C=C\c3ccc(O)c(O)c3)[C@@H](CO)O[C@H]4OC[C@H](O[C@H]24)c5ccc([O-])c(O)c5)[C@H](O)[C@H](O)[C@H]1O |
| 1715 | OC[C@H]1O[C@H]2OC[C@H](O[C@@H]2[C@@H](O)[C@@H]1OC(=O)\C=C/c3ccc(O)c(O)c3)c4ccc(O)c(O)c4 |
| 1716 | OC[C@H]1O[C@H]2OC[C@H](O[C@@H]2[C@@H](O)[C@@H]1OC(=O)\C=C/c3ccc(O)c(O)c3)c4ccc([O-])c(O)c4 |
| 1717 | OC1=C(O)C(=C2[C@H](C1)OC(=CC2=O)c3ccc(O)cc3)[O-] |
| 1718 | OC1=C(O)C(=C2[C@H](C1)OC(=CC2=O)c3ccc([O-])cc3)[O-] |
| 1719 | CC(=CCC[C@H]([C@H]1CNC(=[NH+]1)N)C(=O)[O-])C |
| 1720 | CC(=CCC[C@H]([C@H]1CNC(=N1)N)C(=O)[O-])C |
| 1721 | CC(=O)O[C@@H]1CC2=C([C@H](OC(=O)C)[C@H]1OC(=O)C)C(=O)C=C(CCc3ccccc3OC(=O)C)O2 |
| 1722 | CC1=CCC[C@@]2(C)CC[C@@H]3C[C@]12OC3(C)C |
| 1723 | CC1(C)O[C@]23C[C@H]1CC[C@]2(C)CCCC3=C |
| 1724 | CC(C)[C@H]1CC(=O)c2coc3[C@@H](O)[C@@H](C)C[C@H]1c23 |
| 1725 | C[C@@H]1CCC=C(C)[C@]12CC[C@@H](C2)C(C)(C)O |
| 1726 | O[C@@H]1[C@H](O)[C@@H](O)C2=C(OC(=CC2=O)CCc3ccccc3)[C@H]1O |
| 1727 | O[C@H]1[C@@H](O)[C@H](Oc2cc3OC(=CC(=O)c3cc2O[C@@H]4[C@@H](O)[C@@H](O)[C@H](O)C5=C4C(=O)C=C(CCc6ccccc6)O5)CCc7ccccc7)C8=C(OC(=CC8=O)CCc9ccccc9)[C@@H]1O |
| 1728 | O[C@@H]1[C@@H](O)[C@H](Oc2cc3OC(=CC(=O)c3cc2O[C@@H]4[C@@H](O)[C@@H](O)[C@H](O)C5=C4C(=O)C=C(CCc6ccccc6)O5)CCc7ccccc7)C8=C(OC(=CC8=O)CCc9ccccc9)[C@H]1O |
| 1729 | O[C@H]1[C@@H](O)[C@H](Oc2cc3OC(=CC(=O)c3cc2O[C@@H]4[C@@H](O)[C@H](O)[C@@H](O)C5=C4C(=O)C=C(CCc6ccccc6)O5)CCc7ccccc7)C8=C(OC(=CC8=O)CCc9ccccc9)[C@@H]1O |
| 1730 | O[C@@H]1[C@H](O)[C@@H](Oc2ccc(O[C@H]3[C@@H](O)[C@H](O)C4=C([C@@H]3O)C(=O)C=C(CCc5ccccc5)O4)c6C(=O)C=C(CCc7ccccc7)Oc26)C8=C(OC(=CC8=O)CCc9ccccc9)[C@H]1O |
| 1731 | O[C@H]1[C@H](O)C2=C([C@@H]3Oc4cc5C(=O)C=C(CCc6ccccc6)Oc5cc4O[C@@H]13)C(=O)C=C(CCc7ccccc7)O2 |
| 1732 | C[C@@H]1CCC=C(C=O)[C@@]12CC[C@H](C2)C(C)(C)O |
| 1733 | CC1(C)O[C@]23C[C@H]1CC[C@]2(C)CCC[C@H]3CO |
| 1734 | CC(=O)CCc1ccccc1 |
| 1735 | COc1cc(cc(OC)c1O)[C@H]2Oc3c(OC)cc4C=CC(=O)Oc4c3O[C@@H]2CO |
| 1736 | COc1cc(cc(OC)c1[O-])[C@H]2Oc3c(OC)cc4C=CC(=O)Oc4c3O[C@@H]2CO |
| 1737 | C[C@@H]1CC=CC2=CC[C@H](C[C@@]12C)C(C)(C)O |
| 1738 | C[C@@H]1CCC[C@@]2(C)CC[C@@H]3C[C@]12OC3(C)C |
| 1739 | C[C@H]1CCCC2=CC(=O)C(=C(C)C)C[C@]12C |
| 1740 | CC1(C)O[C@]23C[C@H]1CC[C@]2(C)CC[C@H](O)[C@@]3(C)O |
| 1741 | Oc1cc(O)c2OC(=CC(=O)c2c1)CCc3ccccc3 |
| 1742 | Oc1ccc(c2C(=O)C=C(CCc3ccccc3)Oc12)c4c(O)ccc5OC(=CC(=O)c45)CCc6ccccc6 |
| 1743 | Oc1ccc2OC(=CC(=O)c2c1c3ccc([O-])c4OC(=CC(=O)c34)CCc5ccccc5)CCc6ccccc6 |
| 1744 | CC1=C2C[C@@H](CC[C@]2(C)CCC1)C(C)(C)O |
| 1745 | C[C@H]1CC[C@@]23O[C@]2(C)CC[C@H](C[C@H]13)C(=C)C |
| 1746 | C[C@H]1CC[C@]23O[C@]12C[C@@H](CCC3=O)C(=C)C |
| 1747 | CC(C)[C@H]1CC(=O)c2coc3C(=O)[C@@H](C)C[C@H]1c23 |
| 1748 | C[C@H]1CCC2=C(CC[C@H](C[C@@H]12)C(=C)C)C=O |
| 1749 | C[C@H]1CCC2=C(CC[C@H](C[C@@H]12)C(=C)C)C(=O)[O-] |
| 1750 | C[C@H]1CCC2=C(CO)CC[C@H](C[C@@H]12)C(=C)C |
| 1751 | C[C@H]1C[C@H]2OC(=O)C3=C2[C@H]1C[C@@H](CC3)C(=C)C |
| 1752 | C[C@H]1CCC2=C(C)C(=O)C[C@H](C[C@@H]12)C(=C)C |
| 1753 | [O-]C(=O)CCc1ccccc1 |
| 1754 | C[C@@]1(O)CCC[C@@]2(C)CC[C@@H]3C[C@]12OC3(C)C |
| 1755 | Oc1ccc2OC(=CC(=O)c2c1)CCc3ccccc3O |
| 1756 | Oc1ccc(CCC2=CC(=O)c3cc(O)ccc3O2)cc1 |
| 1757 | COc1cc2OC(=CC(=O)c2cc1O)CCc3ccccc3 |
| 1758 | Oc1ccc2C(=O)C=C(CCc3ccccc3)Oc2c1 |
| 1759 | [O-]c1ccc2C(=O)C=C(CCc3ccccc3)Oc2c1 |
| 1760 | O[C@H]1[C@H](O)[C@@H](O)C2=C(OC(=CC2=O)CCc3ccccc3)[C@@H]1O |
| 1761 | C[C@@H]1CCCC2=CC[C@H](C[C@@]12C)C(C)(C)O |
| 1762 | C[C@H]1CC[C@H]2C(C)(C)[C@@](C)(O)[C@@H]3CC[C@]12C3 |
| 1763 | C[C@H]1CC[C@H]2C(C)(C)[C@H](CO)[C@@H]3CC[C@]12C3 |
| 1764 | C[C@H]1CC=CC2=CC(=O)C(=C(C)C)C[C@]12C |
| 1765 | C[C@H]1CCC=C2CC[C@@H](C[C@]12C)C(C)(C)O |
| 1766 | O=C1c2ccccc2c3c4OCOc4cc5ccnc1c35 |
| 1767 | COc1ccc(CCC(=O)C)cc1 |
| 1768 | COc1ccc(CCC(=O)[O-])cc1 |
| 1769 | COc1ccc2OC(=CC(=O)c2c1)CCc3ccc(O)c(OC)c3 |
| 1770 | COc1ccc(CCC2=CC(=O)c3ccccc3O2)cc1 |
| 1771 | COc1ccc2OC(=CC(=O)c2c1)CCc3ccccc3 |
| 1772 | COC(=O)C1=C2CC[C@H](C)[C@@H]2C[C@@H](CC1)C(=C)C |
| 1773 | CCC(=O)C1=C2C[C@H](C[C@@H](O)[C@]2(C)CCC1)C(=C)C |
| 1774 | COC(=O)C1=CCC[C@]2(C)CC[C@H](C[C@@H]12)C(=C)C |
| 1775 | COC(=O)C1=C2C[C@@H](CC[C@@]2(C)CCC1)C(=C)C |
| 1776 | C[C@H]1CCCC2=CC(=O)[C@H](C[C@]12C)C(=C)C |
| 1777 | CC1(C)O[C@]23C[C@H]1CC[C@]2(C)CCCC3=O |
| 1778 | O=C1C=C(CCc2ccccc2)Oc3ccccc13 |
| 1779 | O[C@H]1[C@H](O)[C@H](O)C2=C(OC(=CC2=O)CCc3ccccc3)[C@@H]1O |
| 1780 | O[C@H]1[C@@H](O)[C@H](Oc2ccc3OC(=CC(=O)c3c2)CCc4ccccc4)C5=C([C@@H]1O)C(=O)C=C(CCc6ccccc6)O5 |
| 1781 | O[C@H]1[C@H](O)[C@@H](Oc2ccc3OC(=CC(=O)c3c2)CCc4ccccc4)C5=C(OC(=CC5=O)CCc6ccccc6)[C@@H]1O |
| 1782 | COc1cc2OC(=CC(=O)c2cc1O[C@H]3[C@H](O)[C@H](O)[C@@H](O)C4=C3OC(=CC4=O)CCc5ccccc5)CCc6ccccc6 |
| 1783 | O[C@@H]1[C@H](O)[C@@H](Oc2cc3C(=O)C=C(CCc4ccccc4)Oc3cc2O)C5=C(OC(=CC5=O)CCc6ccccc6)[C@H]1O |
| 1784 | O[C@@H]1[C@H](O)[C@@H](Oc2cc3C(=O)C=C(CCc4ccccc4)Oc3cc2[O-])C5=C(OC(=CC5=O)CCc6ccccc6)[C@H]1O |
| 1785 | CC(=C)[C@@H]1CC[C@@]2(C)CCC=C(C=O)[C@@H]2C1 |
| 1786 | CC(=C)[C@@H]1CC[C@@]2(C)CCCC(=C2C1)C=O |
| 1787 | CC(=C)[C@@H]1C[C@H](O)[C@@]2(C)CCC=C(C)[C@H]2C1 |
| 1788 | CC(=C)[C@H]1C[C@@H]2C(=CCC[C@]2(C)C(=O)C1)C |
| 1789 | CC(C)[C@@H]1CC[C@@]2(C)CCC[C@@H](C)[C@H]2C1 |
| 1790 | COc1ccc(CCC2=CC(=O)C3=C(O2)[C@@H](OC(=O)C)[C@H](OC(=O)C)[C@H](OC(=O)C)[C@H]3OC(=O)C)cc1 |
| 1791 | O[C@@H]1[C@H](O)[C@@H](O)C2=C(OC(=CC2=O)CCc3ccccc3O)[C@H]1O |
| 1792 | O[C@H]1[C@H](O)[C@@H](O)C2=C(OC(=CC2=O)CCc3ccccc3O)[C@@H]1O |
| 1793 | COc1ccc(CCC2=CC(=O)C3=C(O2)[C@H](O)[C@@H](O)[C@H](O)[C@H]3O)cc1 |
| 1794 | CO[C@H]1[C@H](O)[C@H](O)[C@@H](O)C2=C1OC(=CC2=O)CCc3ccccc3 |
| 1795 | COc1cccc(OC)c1O |
| 1796 | C[C@H]1CCC=C(C)[C@]12CC[C@@H](C2)C(C)(C)O |
| 1797 | C\C=C/c1ccc2OCOc2c1 |
| 1798 | CCCCCCCCC\C=C\C(=O)[O-] |
| 1799 | Cc1coc2cc(C)c3ccc(C)c3cc12 |
| 1800 | CC(=O)O[C@H]1C[C@@H]2CC[C@@]1(C)C2(C)C |
| 1801 | C[C@@H]1CC[C@H]2[C@@H]1[C@H]3[C@@H](CC[C@@]2(C)O)C3(C)C |
| 1802 | CC(C)[C@@H]1CCC(=C)C=C1 |
| 1803 | CC(C)(C)c1ccc(O)c(c1)C(C)(C)C |
| 1804 | C[C@H]1CCCC2=CC[C@@H]3[C@@H](C3(C)C)[C@@]12C |
| 1805 | C[C@H]1CC(=O)C=C2CC[C@@H](C[C@]12C)C(=C)C |
| 1806 | C[C@H]1CCC2=C(C)CC[C@H](C[C@@H]12)C(=C)C |
| 1807 | C[C@H]1CCC2=C1C[C@H]3CC[C@]2(C)C3(C)C |
| 1808 | CC1(C)CCC[C@]2(C)[C@H]3CC[C@H]([C@H]13)C2=C |
| 1809 | Oc1ccc(O)c2C(=O)C=C(CCc3ccccc3)Oc12 |
| 1810 | C[C@@H]1CC[C@H]2[C@@H]1[C@H]3[C@@H](CC[C@]2(C)O)C3(C)C |
| 1811 | CCCCCCCCCCC\C=C\C(=O)[O-] |
| 1812 | CC(C)c1ccc(C)c2ccc(C)c2c1 |
| 1813 | C[C@@H]1CCC=C(C)[C@@]12CC[C@H](C2)C(C)(C)O |
| 1814 | N(c1ccccc1)c2ccccc2 |
| 1815 | C\C(=C/CC[C@@]1(C)[C@H]2CC[C@H](C2)C1=C)\CO |
| 1816 | CC1(C)O[C@]23C[C@H]1CC[C@]2(C)CCC=C3CO |
| 1817 | CC(C)(C)C1=CC(C)(O)C=C(C1=O)C(C)(C)C |
| 1818 | COc1ccc(C[C@@H]2N(C)CCc3cc(OC)c(O)cc23)cc1O |
| 1819 | COc1ccc(CCC2=CC(=O)c3c(O)ccc(O)c3O2)cc1 |
| 1820 | COc1cc2OC(=CC(=O)c2cc1OC)CCc3ccccc3 |
| 1821 | COc1ccc(CCC2=CC(=O)c3cc(OC)c(OC)cc3O2)cc1 |
| 1822 | Oc1ccc2OC(=CC(=O)c2c1)CCc3ccccc3 |
| 1823 | COc1ccc(CCC2=CC(=O)c3cc(O)ccc3O2)cc1 |
| 1824 | COc1cccc(CCC2=CC(=O)c3cc(OC)ccc3O2)c1 |
| 1825 | CC(C)(C)C1=CC2(CCC(=O)O2)C=C(C1=O)C(C)(C)C |
| 1826 | O[C@H]1[C@@H](O)[C@@H](O)C2=C(OC(=CC2=O)CCc3ccccc3)[C@@H]1O |
| 1827 | C[C@@H]1CCC=C(C(=O)[O-])[C@@]12CC[C@H](C2)C(C)(C)O |
| 1828 | CCCCOCCOCCO |
| 1829 | C[C@H]1CCC[C@@]2(C)CC[C@@H]3C[C@@]12OC3(C)C |
| 1830 | CC(C)[C@@H]1CC(=O)c2coc3C(=O)[C@H](C)C[C@@H]1c23 |
| 1831 | O[C@H]1[C@@H](O)[C@H](O)C2=C(OC(=CC2=O)CCc3ccccc3)[C@@H]1O |
| 1832 | C[C@H]1CC[C@@H]2[C@H]1[C@H]3[C@@H](C[C@H]4O[C@@]24C)C3(C)C |
| 1833 | C[C@@H]1CCC=C2CC[C@H](C[C@@]12C)C(C)(C)O |
| 1834 | CC(=C)[C@@H]1CCc2cc(O)cc(C=O)c2C1 |
| 1835 | CC(C)(C)c1cc(CCC(=O)[O-])cc(c1O)C(C)(C)C |
| 1836 | [O-]C(=O)C#Cc1ccccc1 |
| 1837 | C[C@@H]1CC[C@H]2C[C@@]3(OC2(C)C)[C@H](CC[C@@H]13)C=O |
| 1838 | C[C@@H]1C[C@@H]2C[C@]3(OC2(C)C)[C@H](CO)CC[C@@H]13 |
| 1839 | CC1(C)O[C@@]23C[C@@H]1CC[C@@]2(C)CC[C@H](O)[C@@]3(C)O |
| 1840 | C[C@@H]1CC[C@@H](\C=C(/C)\C(=O)[O-])C2=C(C)CC[C@H]12 |
| 1841 | CC1=C[C@H](C=O)C(=C[C@H]2CC(C)(C)C[C@H]12)C=O |
| 1842 | C[C@H]1CCC2=C(CC[C@@H](C[C@H]12)C(=C)C)C=O |
| 1843 | CC(C)(C)c1cc(O)cc(c1)C(C)(C)C |
| 1844 | CC1(C)C=CC=C2C(C)(C)[C@@H]3C[C@]12C=C3 |
| 1845 | C[C@@]1(O)CCC[C@]2(C)CC[C@H]3C[C@@]12OC3(C)C |
| 1846 | CCc1ccc(C)c2ccc(C)c2c1 |
| 1847 | C\C\1=C/CCC2=C[C@H](OC2=O)c3c(C)coc3C1 |
| 1848 | CCC[C@H]1O[C@@H]1CCCCC(C)C |
| 1849 | COc1cc2c(C[C@@H]3[NH2+]CCc4cc(O)c(OC)c2c34)cc1O |
| 1850 | COc1cc2c(C[C@@H]3[NH2+]CCc4cc(O)c(OC)c2c34)cc1[O-] |
| 1851 | Cc1coc2C[C@@]3(C)[C@@H]4C[C@@H]4C(=C)[C@@H]3[C@@H](O)c12 |
| 1852 | CCCCCCC\C=C\C(=O)C=C |
| 1853 | CCCCCCC\C=C\CC=C |
| 1854 | Cc1cc(O)cc(C)c1C |
| 1855 | CCCCCCC\C=C\CCC(=O)[O-] |
| 1856 | C\C\1=C/CC[C@]23O[C@H]2[C@@H](OC3=O)c4c(C)coc4C1 |
| 1857 | CCCCCCC[C@@H]1O[C@@H]1CCC |
| 1858 | C\C\1=C\CC[C@@H]2C[C@@H](OC2=O)c3c(C)coc13 |
| 1859 | O[C@H](Cc1ccc(O)cc1)C(=O)c2c(O)cc(O)cc2O |
| 1860 | O[C@H](Cc1ccc(O)cc1)C(=O)c2c(O)cc(O)cc2[O-] |
| 1861 | COc1cc2c(C[C@@H]3[N@@H+](C)CCc4cc(O)c(OC)c2c34)cc1O |
| 1862 | COc1cc2c(C[C@@H]3N(C)CCc4cc(O)c(OC)c2c34)cc1O |
| 1863 | COc1cc2c(C[C@@H]3N(C)CCc4cc(O)c(OC)c2c34)cc1[O-] |
| 1864 | COc1ccc(cc1O)[C@@H]2CC(=O)c3c(O)cc(O[C@@H]4O[C@H](CO[C@@H]5O[C@H](C)[C@H](O)[C@@H](O)[C@H]5O)[C@@H](O)[C@H](O)[C@H]4O)cc3O2 |
| 1865 | C[NH2+]CCc1ccc(O)cc1 |
| 1866 | C[NH2+]CCc1ccc([O-])cc1 |
| 1867 | C[C@@H]1O[C@@H](O[C@@H]2[C@@H](O)[C@H](O)[C@@H](CO)O[C@H]2Oc3cc(O)c4C(=O)C[C@H](Oc4c3)c5ccc(O)cc5)[C@H](O)[C@H](O)[C@H]1O |
| 1868 | COc1ccc(cc1OC)C2=C(O[C@@H]3O[C@H](CO)[C@@H](O)[C@H](O)[C@H]3O)C(=O)c4c(OC)c(OC)c(OC)c(OC)c4O2 |
| 1869 | C[NH2+]C[C@H](O)c1ccc(O)cc1 |
| 1870 | CNC[C@H](O)c1ccc(O)cc1 |
| 1871 | CNC[C@H](O)c1ccc([O-])cc1 |
| 1872 | CC1=C[C@H]2[C@@H](CC1)C2(C)C |
| 1873 | COc1cc(O)cc(c1)[C@@H]2CC(=O)c3c(O)cc(O[C@@H]4O[C@H](CO)[C@@H](O)[C@H](O)[C@H]4O[C@@H]5O[C@@H](C)[C@H](O)[C@@H](O)[C@H]5O)cc3O2 |
| 1874 | COc1ccc(cc1O)C(=O)[O-] |
| 1875 | CC[C@@H](C)CCC[C@@H](C)CCCC(C)C |
| 1876 | CCCC(=O)OCC[C@H](C)CCC=C(C)C |
| 1877 | CCCCCCCCCC#C |
| 1878 | CC1(C)O[C@](C)(CC[C@@H]1O)C=C |
| 1879 | CCCCCCCCC\C=C\C=O |
| 1880 | CNc1ccccc1C(=O)OC |
| 1881 | COc1ccc(cc1O)[C@H]2CC(=O)c3c(O)cc(O)cc3O2 |
| 1882 | CCCCCCCCCCC=O |
| 1883 | COC1=C(Oc2c(OC)c(OC)c(OC)c(OC)c2C1=O)c3ccc(OC)c(OC)c3 |
| 1884 | C[C@@H]1O[C@@H](O[C@@H]2[C@@H](O)[C@H](O)[C@@H](CO)O[C@H]2Oc3cc(O)c4C(=O)C[C@H](Oc4c3)c5ccc(O)cc5)[C@H](O)[C@H](O)[C@H]1O |
| 1885 | CC(=C)[C@H]1CC\C(=C/CC\C(=C/C1)\C)\C |
| 1886 | COc1ccc(cc1)C2=CC(=O)c3c(OC)c(OC)c(OC)c(OC)c3O2 |
| 1887 | COc1ccc(cc1OC)[C@H]2CC(=O)c3c(OC)c(OC)c(OC)c(OC)c3O2 |
| 1888 | C\C(=C/C\C=C(/C)\C=C)\CC\C=C(/C)\C=O |
| 1889 | CC#CC[C@H]1CCCCC1=O |
| 1890 | CC(=C)CCC(C)(C)C=C |
| 1891 | CC(C)CCCC[C@@H](C)CCCC(C)C |
| 1892 | CC(=CCC\C=C(/C)\CO)C |
| 1893 | CCC[C@H](C)OCC |
| 1894 | CCCCCCC#C[C@H](C)O |
| 1895 | CC(=C)CCO |
| 1896 | CC(=O)c1ccc(cc1)C(=O)[O-] |
| 1897 | OCCCCCC=C |
| 1898 | C\C=C(\C)/CCC=C(C)C |
| 1899 | CCOC(=O)OCC |
| 1900 | COc1ccc(cc1OC)C2=CC(=O)c3c(OC)c(OC)c(OC)c(OC)c3O2 |
| 1901 | CCCCCCCCCCCOC(=O)C |
| 1902 | C[C@@]12CC(=O)[C@@H]3C[C@@](CC(=O)Oc4ccccc4)([C@@H]3O1)[C@@]25O[C@@H]6O[C@H](CO)[C@@H](O)[C@H](O)[C@H]6O5 |
| 1903 | CC[C@H](CC[C@@H](C)[C@H]1CC[C@H]2[C@@H]3CC=C4C[C@H](CC[C@]4(C)[C@H]3CC[C@]12C)O[C@H]5O[C@H](CO)[C@@H](O)[C@H](O)[C@H]5O)C(C)C |
| 1904 | CC1(C)CC[C@]2(C)CC[C@]3(C)C(=CC[C@@H]4[C@@]5(C)CC[C@H](O)C(C)(C)[C@@H]5CC[C@@]34C)[C@@H]2C1 |
| 1905 | C[C@H]1[C@@H](O)CC[C@@H]2[C@]1(C)CC[C@H]3[C@@]2(C)CC[C@@]4(C)[C@@H]5CC(C)(C)CC[C@]5(C)CC[C@]34C |
| 1906 | C[C@H]1C(=O)CC[C@@H]2[C@]1(C)CC[C@H]3[C@@]2(C)CC[C@@]4(C)[C@@H]5CC(C)(C)CC[C@]5(C)CC[C@]34C |
| 1907 | OC[C@@H]1O[C@@H](Oc2ccc(O)cc2)[C@@H](O)[C@H](O)[C@@H]1O |
| 1908 | CCCCCCCCCCCCCCCCCCCCCCC |
| 1909 | Oc1cc2C(=O)Oc3c(O)c(O)cc4C(=O)Oc(c1O)c2c34 |
| 1910 | Oc1cc2C(=O)Oc3c(O)c([O-])cc4C(=O)Oc(c1O)c2c34 |
| 1911 | Oc1c([O-])cc2C(=O)Oc3c(O)c([O-])cc4C(=O)Oc1c2c34 |
| 1912 | CCCCCCCCCCCCCCCCCCCCCC |
| 1913 | CCCCCCCC\C=C/CCCCCCCC(=O)N |
| 1914 | COc1ccc(C(=O)C)c(O[C@@H]2O[C@H](CO[C@@H]3OC[C@H](O)[C@H](O)[C@H]3O)[C@@H](O)[C@H](O)[C@H]2O)c1 |
| 1915 | CC[C@H](\C=C\[C@@H](C)[C@H]1CC[C@H]2C3=CC[C@H]4C[C@@H](O)CC[C@]4(C)[C@H]3CC[C@]12C)C(C)C |
| 1916 | CC(C)[C@H](C)CC[C@@H](C)[C@H]1CC[C@H]2[C@@H]3CC=C4C[C@@H](O)CC[C@]4(C)[C@H]3CC[C@]12C |
| 1917 | C[C@@]12C[C@@]3(O)O[C@@H](O1)[C@]4(COC(=O)c5ccc(O)cc5)[C@H]3C[C@]24O |
| 1918 | C[C@@]12C[C@@]3(O)O[C@@H](O1)[C@]4(COC(=O)c5ccc([O-])cc5)[C@H]3C[C@]24O |
| 1919 | CCCCCCCCCCCCCCCCCCCCCCC(=O)OC |
| 1920 | Cc1cc(C)c(C)cc1C |
| 1921 | CC(=O)c1ccccc1O |
| 1922 | Oc1cc(C(=O)[O-])c(c(O)c1O)c2c(O)c(O)c(O)cc2C(=O)[O-] |
| 1923 | COc1cc(ccc1O)c2[o+]c3cc(O)cc(O)c3cc2O |
| 1924 | COc1cc(ccc1[O-])c2[o+]c3cc(O)cc(O)c3cc2O |
| 1925 | O[C@H]1O[C@@H]2COC(=O)c3cc(O)c(O)c(O)c3c4c(O)c(O)c(O)cc4C(=O)O[C@@H]2[C@@H]5OC(=O)c6cc(O)c(O)c(O)c6c7c(O)c(O)c(O)cc7C(=O)O[C@@H]15 |
| 1926 | CC1(C)O[C@@]2(C)CC[C@@H]1C[C@@H]2O[C@H]3O[C@H](CO)[C@@H](O)[C@@H](O)[C@@H]3O |
| 1927 | C[C@]12CC(=O)[C@H]3C[C@]1(O[C@@H]4O[C@H](CO)[C@@H](O)[C@H](O)[C@H]4O)[C@@]3(COC(=O)c5ccccc5)CO2 |
| 1928 | C[C@@]12CC(=O)[C@H]3C[C@]1(O)[C@@]3(COC(=O)c4ccccc4)CO2 |
| 1929 | CO[C@@H]1O[C@]2(C)CC(=O)[C@H]3C[C@]2(O[C@@H]4O[C@H](CO)[C@@H](O)[C@H](O)[C@H]4O)[C@@]13COC(=O)c5ccccc5 |
| 1930 | CO[C@@H]1O[C@]2(C)CC(=O)[C@H]3C[C@]2(O)[C@@]13COC(=O)c4ccccc4 |
| 1931 | CC[C@H](CC[C@@H](C)[C@H]1CC[C@H]2C3=CC[C@H]4C[C@@H](O)CC[C@]4(C)[C@@H]3CC[C@]12C)C(C)C |
| 1932 | COc1ccc(\C=C\C)cc1O[C@@H]2O[C@H](CO[C@@H]3OC[C@H](O)[C@H](O)[C@H]3O)[C@@H](O)[C@H](O)[C@H]2O |
| 1933 | COc1ccc(\C=C/C)cc1O |
| 1934 | C[C@@]12C[C@@]3(O)O[C@@H](O1)[C@]4(COC(=O)c5ccccc5)[C@H]3C[C@]24O[C@@H]6O[C@@H](CO)[C@@H](O)[C@@H](O)[C@@H]6O |
| 1935 | C[C@@]12C[C@@]3(O)O[C@@H](O1)[C@]4(COC(=O)c5ccccc5)[C@H]3C[C@]24O[C@@H]6O[C@@H](COC(=O)c7ccccc7)[C@@H](O)[C@@H](O)[C@@H]6O |
| 1936 | C[C@]12C[C@H](O)[C@@H]3C[C@@]1(O[C@@H]4O[C@@H](CO)[C@@H](O)[C@@H](O)[C@@H]4O)[C@]3(COC(=O)c5ccccc5)C(=O)O2 |
| 1937 | C[C@]12C[C@@H](O)[C@@H]3C[C@@]1(O)[C@]3(COC(=O)c4ccccc4)C(=O)O2 |
| 1938 | C[C@@]12C[C@@]3(O)O[C@@H](O1)[C@]4(COC(=O)c5ccc(O)cc5)[C@H]3C[C@]24O[C@@H]6O[C@@H](CO)[C@@H](O)[C@@H](O)[C@@H]6O |
| 1939 | C[C@@]12C[C@@]3(O)O[C@@H](O1)[C@]4(COC(=O)c5ccc([O-])cc5)[C@H]3C[C@]24O[C@@H]6O[C@@H](CO)[C@@H](O)[C@@H](O)[C@@H]6O |
| 1940 | CCO[C@@]12C[C@H]3O[C@@H](O1)[C@@]4(COC(=O)c5ccccc5)[C@@H]2C[C@@]34O |
| 1941 | C[C@]12C[C@@H](OC(=O)c3cc(O)c(O)c(O)c3)[C@@H]4C[C@@]1(O[C@@H]5O[C@H](CO)[C@@H](O)[C@H](O)[C@H]5O)[C@]4(COC(=O)c6ccccc6)C(=O)O2 |
| 1942 | C[C@]12C[C@@H](OC(=O)c3cc(O)c([O-])c(O)c3)[C@@H]4C[C@@]1(O[C@@H]5O[C@H](CO)[C@@H](O)[C@H](O)[C@H]5O)[C@]4(COC(=O)c6ccccc6)C(=O)O2 |
| 1943 | C[C@]12C[C@@H](OC(=O)c3cc(O)c(O)c(O)c3)[C@@H]4C[C@@]1(O)[C@]4(COC(=O)c5ccccc5)C(=O)O2 |
| 1944 | C[C@]12C[C@@H](OC(=O)c3cc(O)c([O-])c(O)c3)[C@@H]4C[C@@]1(O)[C@]4(COC(=O)c5ccccc5)C(=O)O2 |
| 1945 | CO[C@@H]1O[C@@]2(C)CC(=O)[C@@H]3C[C@@]2(O)[C@]13CO |
| 1946 | C[C@]12CC(=O)[C@@H]3C[C@@]1(O)O[C@H](O2)[C@H]3COC(=O)c4ccccc4 |
| 1947 | CCO[C@H]1O[C@@H]2CC[C@@H](C(=O)C2)[C@]1(COC(=O)c3ccccc3)O[C@@H]4O[C@H](CO)[C@@H](O)[C@H](O)[C@H]4O |
| 1948 | CCO[C@H]1O[C@@H]2CC[C@@H](C(=O)C2)[C@@]1(O)COC(=O)c3ccccc3 |
| 1949 | C[C@@]12C[C@@H](O)[C@@]3(O)C[C@@H]1[C@@]3(COC(=O)c4ccccc4)C(=O)O2 |
| 1950 | CC(C)(OC(=O)c1ccccc1)C2CCC(=C)CC2 |
| 1951 | COc1cc(ccc1O)[C@H](CO)C(=O)c2ccc(O)c(OC)c2 |
| 1952 | COc1cc(ccc1O)[C@H](CO)C(=O)c2ccc([O-])c(OC)c2 |
| 1953 | C[C@]12C[C@]3(O)O[C@H](O1)[C@@]4(COC(=O)c5ccccc5)[C@@H]3C[C@@]24O[C@@H]6O[C@H](COC(=O)c7cc(O)c(O)c(O)c7)[C@@H](O)[C@H](O)[C@H]6O |
| 1954 | C[C@]12C[C@]3(O)O[C@H](O1)[C@@]4(COC(=O)c5ccccc5)[C@@H]3C[C@@]24O[C@@H]6O[C@H](COC(=O)c7cc(O)c([O-])c(O)c7)[C@@H](O)[C@H](O)[C@H]6O |
| 1955 | C[C@]12C[C@]3(O)O[C@H](O1)[C@@]4(COC(=O)c5ccccc5)[C@@H]3C[C@@]24O |
| 1956 | C[C@]12C[C@]3(O)O[C@H](O1)[C@@]4(COC(=O)c5ccccc5)[C@@H]3C[C@@]24O[C@H]6O[C@@H](COC(=O)c7ccccc7)[C@H](O)[C@@H](O)[C@@H]6O |
| 1957 | COCC1=CO[C@@]2(C[C@H]1C(=O)C=C2C)O[C@@H]3O[C@@H](CO)[C@H](O)[C@@H](O)[C@H]3O |
| 1958 | COCC1=CO[C@]2(O)C[C@H]1C(=O)C=C2C |
| 1959 | CC1=CC(=O)[C@@H]2C[C@@]1(O[C@@H]3O[C@@H](CO)[C@H](O)[C@@H](O)[C@H]3O)OC=C2CO |
| 1960 | CC1=CC(=O)[C@@H]2C[C@@]1(O)OC=C2CO |
| 1961 | CO[C@@H]1O[C@@]2(C[C@@H](C(=O)C=C2C)C1=C)O[C@@H]3O[C@H](CO)[C@@H](O)[C@H](O)[C@H]3O |
| 1962 | CO[C@@H]1O[C@]2(O)C[C@@H](C(=O)C=C2C)C1=C |
| 1963 | Oc1ccccc1C=O |
| 1964 | OCc1ccccc1O |
| 1965 | COC[C@@]12CC[C@H](OC)[C@@]34[C@@H]5C[C@]6(O)[C@@H](OC)[C@H](O)[C@@](OC(=O)C)([C@H]5[C@@H]6OC(=O)c7ccccc7)[C@@H]([C@H](OC)[C@H]13)[C@H]4[N@@H+](C)C2 |
| 1966 | CC(=O)[C@H]1CC[C@H]2[C@@H]3CC[C@H]4CCCC[C@]4(C)[C@H]3C[C@@H](O)[C@]12C |
| 1967 | C[C@]12CC[C@H]3[C@@H](CCC4=CC(=O)CC[C@]34C)[C@@H]1CC=C2 |
| 1968 | CC[N@@H+]1C[C@]2(COC)[C@@H](O)C[C@@H](OC)[C@]34[C@@H]5C[C@]6(O)[C@@H](OC)[C@@H](O)[C@](OC(=O)C)([C@H]([C@H](OC)[C@@H]23)[C@H]14)[C@H]5[C@H]6OC(=O)c7ccccc7 |
| 1969 | CCN1C[C@]2(COC)[C@@H](O)C[C@@H](OC)[C@]34[C@@H]5C[C@]6(O)[C@@H](OC)[C@@H](O)[C@](OC(=O)C)([C@H]([C@H](OC)[C@@H]23)[C@H]14)[C@H]5[C@H]6OC(=O)c7ccccc7 |
| 1970 | COC[C@]12C[N@H+](C)[C@@H]3[C@@H]4[C@H](OC)[C@H]1[C@@]3([C@H](C[C@H]2O)OC)[C@@H]5C[C@]6(O)[C@H](OC)[C@H](O)[C@@]4(OC(=O)C)[C@H]5[C@H]6OC(=O)c7ccccc7 |
| 1971 | C1[C@@H]2O[C@@H]2[C@@H]3[C@H]1[C@@H]4C[C@H]3[C@H]5O[C@@H]45 |
| 1972 | COc1ccc2cccc(OC)c2c1 |
| 1973 | COC[C@]12C[N@H+](C)[C@@H]3[C@@H]4[C@@H](OC)[C@H]1[C@@]3([C@H](C[C@H]2O)OC)[C@@H]5C[C@]6(O)[C@H](OC)[C@@H](O)[C@@]4(O)[C@H]5[C@H]6OC(=O)c7ccccc7 |
| 1974 | CC[N@@H+]1C[C@@]2(COC)CC[C@@H](OC)[C@]34[C@H]5C[C@H]6[C@@H](O)[C@H]5[C@@](O)(C[C@H]6OC)[C@H](C[C@@H]23)[C@H]14 |
| 1975 | CCCC[C@@H]1OC(=O)C2=C1CCC=C2 |
| 1976 | CCCC[C@]1(O)OC(=O)C2=C1CC[C@H](O)[C@H]2O |
| 1977 | CCC\C=C\1/OC(=O)c2c(O)cccc12 |
| 1978 | CCCC[C@@H]1OC(=O)c2ccccc12 |
| 1979 | CCC\C=C\1/OCC(=O)c2cccc(O)c12 |
| 1980 | C[C@@H]1CC[C@@]2(CC[C@]3(C)C(=CC[C@@H]4[C@@]5(C)CC[C@H](OC(=O)\C=C\c6ccc(O)cc6)C(C)(C)[C@@H]5CC[C@@]34C)[C@@H]2[C@H]1C)C(=O)O |
| 1981 | C[C@@H]1CC[C@@]2(CC[C@]3(C)C(=CC[C@@H]4[C@@]5(C)CC[C@H](OC(=O)\C=C\c6ccc(O)cc6)C(C)(C)[C@@H]5CC[C@@]34C)[C@@H]2[C@H]1C)C(=O)[O-] |
| 1982 | Cc1nc(C)c(C)nc1C |
| 1983 | CCCC[C@H]1OC(=O)[C@@H]2CCCC=C12 |
| 1984 | CCC\C=C\1/OC(=O)C2=C1CC[C@@H](O)[C@@H]2O |
| 1985 | CCC\C=C\1/OC(=O)C2=C1CC[C@H](O)[C@@H]2O |
| 1986 | CCCC[C@@H]1OC(=O)C2=C1CC[C@H]3[C@H]4CC[C@]5([C@@H]23)\C(=C\CCC)\OC(=O)C5=C4 |
| 1987 | CCCC[C@@H]1OC(=O)c2c(O)ccc(O)c12 |
| 1988 | CCC\C=C\1/OC(=O)C2=C1CC[C@H]3[C@H]4CC[C@]5([C@@H]23)\C(=C\CCC)\OC(=O)C5=C4 |
| 1989 | CCC\C=C\1/OC(=O)C2=C1CC[C@@H]3O[C@@H]23 |
| 1990 | CCOC(=O)CCCCCCCCCCCCCC(C)C |
| 1991 | CCOC(=O)CCCCCCCCCCCCCCC(C)C |
| 1992 | Nc1nc(O)c2nc(CNc3ccc(cc3)C(=O)N[C@@H](CCC(=O)[O-])C(=O)[O-])cnc2n1 |
| 1993 | COc1cc(ccc1O)[C@@H]2C[C@@H]3C(=O)C=C[C@@]34O[C@@]24CO |
| 1994 | CCC\C=C\1/OC(=O)C2=C1CCC=C2 |
| 1995 | CCC\C=C/1\OC(=O)C2=C[C@H]3CC[C@@]12[C@@H](CCC)[C@]34OC(=O)C5=C4CCC=C5 |
| 1996 | COC\C=C\c1ccc(O)c(OC)c1 |
| 1997 | CCCC\C=C\CC\C=C\CCCCCCCC(=O)OC |
| 1998 | COC(=O)Cc1ccccc1 |
| 1999 | CCCC[C@@H]1OC(=O)C2=CCCC[C@@H]12 |
| 2000 | CCCCC(=O)c1ccccc1C(=O)OC |
| 2001 | C\C(=C/CO)\C=C\C=C(/C)\C=C\C1=C(C)CCCC1(C)C |
| 2002 | CCCCC(=O)[C@@H]1CCCC=C1C(=O)[O-] |
| 2003 | CCC\C=C\1/OC(=O)c2ccc(O)cc12 |
| 2004 | CCCC(=O)[C@]1(O)OC(=O)C2=C1CCC=C2 |
| 2005 | CC[C@@H](O)\C=C\1/OC(=O)c2ccccc12 |
| 2006 | CC[C@@H](O)\C=C\1/OC(=O)C2=C1CCC=C2 |
| 2007 | CCCC[C@]1(O)OC(=O)C2=C1CCC=C2 |
| 2008 | CCC\C=C\1/OC(=O)C2=C1CC[C@H](O)[C@H]2O |
| 2009 | CCCC[C@@H]1OC(=O)C2=C1CC[C@@H](O)[C@@H]2O |
| 2010 | CCCC[C@@H]1OC(=O)C2=C1[C@H](O)CC=C2 |
| 2011 | CCC\C=C\1/OC(=O)C2=C1CC[C@H](O)[C@H]2Cl |
| 2012 | CCC\C=C\1/OC(=O)C2=C1CC[C@@H]([C@H]2O)C(=O)CCC |
| 2013 | CCCC[C@@H]1OC(=O)C2=C1CC[C@H](O)[C@H]2O |
| 2014 | CCC\C=C\1/OC(=O)C2=C1CC[C@@H]([C@@H]2O)C(=O)CCC |
| 2015 | CC(=CCC\C(=C\CC\C(=C\CC1=CC(=O)C=C(C)C1=O)\C)\C)C |
| 2016 | C[NH+](C)C |
| 2017 | CC(C)[C@@H]([NH3+])C(=O)OC(=O)[C@H]([NH3+])C(C)C |
| 2018 | CC(C)[C@H](N)C(=O)OC(=O)[C@H]([NH3+])C(C)C |
| 2019 | CC(C)[C@H](N)C(=O)OC(=O)[C@@H](N)C(C)C |
| 2020 | CC(=C)[C@@H]1CC[C@@](C)(C=C)[C@H](C1)C(=C)C |
| 2021 | NC1=Nc2ncc(CNc3ccc(cc3)C(=O)N[C@@H](CCC(=O)[O-])C(=O)[O-])nc2C(=O)N1 |
| 2022 | CC(C)c1ccccc1C |
| 2023 | CC(=CCC\C(=C\1/CCC(=CC1)C)\C)C |
| 2024 | CC(=O)c1cc(C)ccc1O |
| 2025 | CC(C)[C@H]1CC[C@@H](C)C\C=C/[C@H](C)CC1 |
| 2026 | C[C@H]1CC[C@H]2C(C)(C)[C@@H]3C[C@]12CC=C3C |
| 2027 | CC1(C)CCCC1 |
| 2028 | CCC\C=C\1/OC(=O)C2=C1CC[C@H]3[C@@H]4CC[C@@]5([C@@H]23)\C(=C/CCC)\OC(=O)C5=C4 |
| 2029 | CCCC[C@@H]1OC(=O)C2=C[C@@H]3CC[C@]12[C@H]4[C@@H]3CCC5=C4C(=O)O/C/5=C/CCC |
| 2030 | CCC\C=C/1\OC(=O)C2=C1CC[C@H]([C@H]2O)C(=O)CCC |
| 2031 | CC[C@@H](O)\C=C/1\OC(=O)C2=C1CC[C@H](O)[C@H]2O |
| 2032 | CCCC(OCC)OCC |
| 2033 | CC1(C)[C@H]2C[C@@H]1C(=C)C=C2 |
| 2034 | CCC\C=C\1/OC(=O)C2=C1CC[C@@H]3[C@@H](CCC)[C@]4(OC(=O)C5=C4CCC=C5)[C@H]23 |
| 2035 | CC(=C)[C@H]1CC[C@]2(C)CCC=C(C)[C@@]2(C)C1 |
| 2036 | C[C@H]1CCC[C@H]2C=C(C)CC=C12 |
| 2037 | CC1(C)CC[C@@]2(CC[C@]3(C)C(=CC[C@@H]4[C@@]5(C)C[C@H](O)[C@H](O)C(C)(C)[C@@H]5CC[C@@]34C)[C@@H]2C1)C(=O)O |
| 2038 | CC1(C)CC[C@@]2(CC[C@]3(C)C(=CC[C@@H]4[C@@]5(C)C[C@H](O)[C@H](O)C(C)(C)[C@@H]5CC[C@@]34C)[C@@H]2C1)C(=O)[O-] |
| 2039 | CCc1cccc(C)c1 |
| 2040 | CC(=C)[C@H]1CC[C@]2(C)CCCC(=C)[C@H]2C1 |
| 2041 | CC(=C)\C=C\C(C)(C)C=C |
| 2042 | CC(C)[C@H]1CC[C@@H](C)[C@]23CCC(=C)[C@H]2[C@H]13 |
| 2043 | C\C\1=C/CC\C(=C\[C@@H]2[C@H](CC1)C2(C)C)\C |
| 2044 | CC(C)[C@@H]1CCC(=C[C@@H]1O)C |
| 2045 | CC(C)[C@@]12CC[C@@](C)(O)[C@@H]1C2 |
| 2046 | CCCC[C@@H]1OC(=O)[C@@H]2C=CCC[C@H]12 |
| 2047 | C1CCCCC\C=C/CCCC1 |
| 2048 | C[C@H]1CCCC(C)(C)[C@H]1C |
| 2049 | CC(C)CCCCCCC(C)C |
| 2050 | CC(=C)[C@@H]1CC[C@@]2(C)CCCC(=C2C1)C |
| 2051 | CC1=CCCC(C)(C)C1=C |
| 2052 | CC(C)C(=O)c1ccccc1 |
| 2053 | COc1c(O)c2CCCCC(=O)CCc3ccc(O)c(c3)c(c2)c1OC |
| 2054 | COc1c([O-])c2CCCCC(=O)CCc3ccc(O)c(c3)c(c2)c1OC |
| 2055 | CCCC[C@@H]1OC(=O)C2=CCCC[C@H]12 |
| 2056 | CCCCCCCC |
| 2057 | CC(=O)[N-]Nc1ccccc1 |
| 2058 | OCc1oc(cc1)c2[nH+]ccc3c4ccccc4[nH]c23 |
| 2059 | OCc1oc(cc1)c2nccc3c4ccccc4[nH]c23 |
| 2060 | CCCCC(=O)[C@H]1CCCC=C1C(=O)[O-] |
| 2061 | CCC\C=C/1\OC(=O)c2ccc(O)cc12 |
| 2062 | CC[C@@H](O)\C=C\1/OC(=O)c2ccccc12 |
| 2063 | CC[C@@H](O)\C=C/1\OC(=O)C2=C1CCC=C2 |
| 2064 | CCCC[C@H]1OC(=O)C2=C1CC[C@H](O)[C@H]2O |
| 2065 | CCC\C=C/1\OC(=O)C2=C1CC[C@H](O)[C@H]2Cl |
| 2066 | CC[C@H](O)C[C@@H]1CC(=O)C2=C1CC[C@H](O)[C@@H]2O |
| 2067 | CC(=O)c1nccc2c3ccccc3[nH]c12 |
| 2068 | COc1cc(\C=C\C(=O)[O-])cc(OC)c1O |
| 2069 | CCCCCC\C=C\CO |
| 2070 | CCCCC(=O)[C@@]12CC[C@@H](C=C1C(=O)OC)[C@H]3CCC4=C([C@@H]23)C(=O)O/C/4=C\CCC |
| 2071 | C[C@@H]1CC[C@@]2(CC[C@]3(C)C(=CC[C@H]4[C@@]5(C)CC[C@@H](C[C@H]5CC[C@@]34C)OC(=O)\C=C/c6cccc(O)c6)[C@@H]2[C@H]1C)C(=O)O |
| 2072 | C[C@@H]1CC[C@@]2(CC[C@]3(C)C(=CC[C@H]4[C@@]5(C)CC[C@@H](C[C@H]5CC[C@@]34C)OC(=O)\C=C/c6cccc(O)c6)[C@@H]2[C@H]1C)C(=O)[O-] |
| 2073 | CC(C)CCCC[C@@H](C)CO |
| 2074 | CC(C)C1=CC[C@](C)(O)CC1 |
| 2075 | CCC(=O)c1occc1 |
| 2076 | CCC(=O)c1ccccc1 |
| 2077 | CC1=CC[C@@H](C=O)C1(C)C |
| 2078 | CCCCC(C)C |
| 2079 | COC(=O)c1occc1 |
| 2080 | CC(=Cc1ccccc1)C |
| 2081 | CC1=C2CC(C2)C=C1 |
| 2082 | CC(=C)[C@H]1CC=C(C)[C@@H](O)C1 |
| 2083 | Cc1oc2ccccc2n1 |
| 2084 | C[C@H]1CCC(=O)O[C@@H]1C |
| 2085 | CCCC[C@@H]1OC(=O)C2=C1CCCC2 |
| 2086 | CC(C)(C)CC[C@@H]1O[C@@H]1C(C)(C)C |
| 2087 | O[C@H]1CCC=CC1 |
| 2088 | COC(=O)c1cocc1 |
| 2089 | CC(C)[C@H]1CCC(=C[C@@H]1O)C |
| 2090 | CCCC[C@H]1OC(=O)c2c(O)ccc(O)c12 |
| 2091 | Ic1cccc2NCCc12 |
| 2092 | CCCCC(=O)CCC |
| 2093 | CCCC[C@H]1OC(=O)c2cccc(O)c12 |
| 2094 | CC1(C)[C@H]2CCC(=C)[C@H]3CC[C@@](C)(O)[C@H]3[C@@H]12 |
| 2095 | CCCC1=CNC(=S)[N-]C1=O |
| 2096 | CCCC[C@@H]1CC=CCC=C1 |
| 2097 | CC(C)C12CCC(C)(CC1)O2 |
| 2098 | C[C@@H]1CC[C@@H]2[C@@H]1[C@H]3[C@@H](CC[C@@]24CO4)C3(C)C |
| 2099 | COC(=O)C[C@@H](O)C(=O)OC |
| 2100 | CCCC[C@H]1OC(=O)c2ccccc12 |
| 2101 | C[C@@H]1CC[C@]23C[C@H]1C(C)(C)[C@@H]2CCC3=C |
| 2102 | CC(C)[C@H]1CC[C@@]2(C)CC=C(C)CC[C@]12O |
| 2103 | COc1ccc(\C=C\COC(=O)\C=C\c2ccc(O)c(OC)c2)cc1O |
| 2104 | CCCc1ccc(O)c(OC)c1 |
| 2105 | CC(C)[C@@H]1CCC(=C)[C@H]2CCC(=C)C[C@@H]12 |
| 2106 | CCCCCC(=O)c1ccccc1 |
| 2107 | CCCCCCC |
| 2108 | CC(C)[C@H]([NH3+])C(=O)CC(=O)[C@H]([NH3+])C(C)C |
| 2109 | CCC\C=C/1\OC(=O)C2=C1CC[C@H]3[C@@H]4CC[C@@]5([C@@H]23)\C(=C/CCC)\OC(=O)C5=C4 |
| 2110 | CCCCC\C=C/C\C=C/CCCCCCCCO |
| 2111 | CCCCCCCCCCCCCCCCCCCCCCCCCCCCCCCCCCCCCC |
| 2112 | CCCCCc1ccccc1CO |
| 2113 | CC(=O)OC[C@H]1O[C@@H](Oc2cccc3C(=O)c4cc(C)cc(O)c4C(=O)c23)[C@H](O)[C@@H](O)[C@@H]1O |
| 2114 | CC(=O)OC[C@H]1O[C@@H](Oc2cccc3C(=O)c4cc(C)cc([O-])c4C(=O)c23)[C@H](O)[C@@H](O)[C@@H]1O |
| 2115 | CC(=O)c1cc(O)cc2OC(=CC(=O)c12)C |
| 2116 | CC(=O)c1cc([O-])cc2OC(=CC(=O)c12)C |
| 2117 | Oc1ccc2C(=O)c3ccccc3C(=O)c2c1[O-] |
| 2118 | OC[C@H]1O[C@@H](Oc2cccc3C(=O)c4cc(CO)cc(O[C@@H]5O[C@H](CO)[C@@H](O)[C@H](O)[C@H]5O)c4C(=O)c23)[C@H](O)[C@@H](O)[C@@H]1O |
| 2119 | OC[C@H]1O[C@@H](OCc2cc(O)c3C(=O)c4c(O)cccc4C(=O)c3c2)[C@H](O)[C@@H](O)[C@@H]1O |
| 2120 | OC[C@H]1O[C@@H](OCc2cc(O)c3C(=O)c4c([O-])cccc4C(=O)c3c2)[C@H](O)[C@@H](O)[C@@H]1O |
| 2121 | OC[C@H]1O[C@@H](OCc2cc([O-])c3C(=O)c4c([O-])cccc4C(=O)c3c2)[C@H](O)[C@@H](O)[C@@H]1O |
| 2122 | OC[C@H]1O[C@@H](Oc2cccc3C(=O)c4cc(CO)cc(O)c4C(=O)c23)[C@H](O)[C@@H](O)[C@@H]1O |
| 2123 | OC[C@H]1O[C@@H](Oc2cccc3C(=O)c4cc(CO)cc([O-])c4C(=O)c23)[C@H](O)[C@@H](O)[C@@H]1O |
| 2124 | Cc1cc(O[C@@H]2O[C@H](CO)[C@@H](O)[C@H](O)[C@H]2O)c3C(=O)c4c([O-])cc(O)cc4C(=O)c3c1 |
| 2125 | OC[C@H]1O[C@@H](Oc2cc(O)cc(\C=C\c3ccc(O)c(O)c3)c2)[C@H](O)[C@@H](O)[C@@H]1O |
| 2126 | OC[C@H]1O[C@@H](Oc2cc([O-])cc(\C=C\c3ccc(O)c(O)c3)c2)[C@H](O)[C@@H](O)[C@@H]1O |
| 2127 | CC1=CC(=O)c2c(O1)cc(O[C@@H]3O[C@H](CO)[C@@H](O)[C@H](O)[C@H]3O)cc2C(=O)[O-] |
| 2128 | CC1=CC(=O)c2c(O1)cc(O)cc2C(=O)[O-] |
| 2129 | CC1=CC(=O)c2c(O1)cc([O-])cc2C(=O)[O-] |
| 2130 | COc1cc([O-])cc2O[C@@](O)(Cc3ccc(O)cc3)C(=O)c12 |
| 2131 | Cc1cc([O-])c2C(=O)c3c(O[C@@H]4O[C@H](CO)[C@@H](O)[C@H](O)[C@H]4O)cccc3[C@H]([C@@H]5O[C@H](CO)[C@@H](O)[C@H](O)[C@H]5O)c2c1 |
| 2132 | CC(=O)Cc1cc(O)cc2OC(=CC(=O)c12)C |
| 2133 | CC(=O)Cc1cc([O-])cc2OC(=CC(=O)c12)C |
| 2134 | Cc1cc([O-])c2C(=O)c3c([O-])cccc3[C@@](O)([C@H]4O[C@H](CO)[C@@H](O)[C@H](O)[C@H]4O)c2c1 |
| 2135 | OC[C@H]1O[C@@H](Oc2cc(O)cc3O[C@@H]([C@@H](O)Cc23)c4ccc(O)c(O)c4)[C@H](O)[C@@H](O)[C@@H]1O |
| 2136 | OC[C@H]1O[C@@H](Oc2cc([O-])cc3O[C@@H]([C@@H](O)Cc23)c4ccc(O)c(O)c4)[C@H](O)[C@@H](O)[C@@H]1O |
| 2137 | Cc1cc(O[C@@H]2O[C@H](CO)[C@@H](O)[C@H](O)[C@H]2O)c3C(=O)c4c([O-])cccc4[C@@H]([C@H]5O[C@H](CO)[C@@H](O)[C@H](O)[C@H]5O)c3c1 |
| 2138 | Cc1cc(O)c2C(=O)c3c(O)cccc3Cc2c1 |
| 2139 | Cc1cc([O-])c2C(=O)c3c(O)cccc3Cc2c1 |
| 2140 | Cc1cc([O-])c2C(=O)c3c([O-])cccc3Cc2c1 |
| 2141 | Oc1cccc2C(=O)c3cccc(O)c3C(=O)c12 |
| 2142 | Oc1cccc2C(=O)c3cccc([O-])c3C(=O)c12 |
| 2143 | [O-]c1cccc2C(=O)c3cccc([O-])c3C(=O)c12 |
| 2144 | Cc1cc(O)c2C(=O)c3c(O[C@@H]4O[C@H](COC(=O)c5cc(O)c(O)c(O)c5)[C@@H](O)[C@H](O)[C@H]4O)cccc3C(=O)c2c1 |
| 2145 | Cc1cc([O-])c2C(=O)c3c(O[C@@H]4O[C@H](COC(=O)c5cc(O)c(O)c(O)c5)[C@@H](O)[C@H](O)[C@H]4O)cccc3C(=O)c2c1 |
| 2146 | Cc1cc([O-])c2C(=O)c3c(O[C@@H]4O[C@H](COC(=O)c5cc(O)c([O-])c(O)c5)[C@@H](O)[C@H](O)[C@H]4O)cccc3C(=O)c2c1 |
| 2147 | Cc1cc(O)c2C(=O)c3c(O[C@@H]4O[C@H](CO[C@@H]5O[C@H](CO)[C@@H](O)[C@H](O)[C@H]5O)[C@@H](O)[C@H](O)[C@H]4O)cccc3C(=O)c2c1 |
| 2148 | Cc1cc([O-])c2C(=O)c3c(O[C@@H]4O[C@H](CO[C@@H]5O[C@H](CO)[C@@H](O)[C@H](O)[C@H]5O)[C@@H](O)[C@H](O)[C@H]4O)cccc3C(=O)c2c1 |
| 2149 | Cc1cc(O)c2C(=O)c3c(O[C@@H]4O[C@H](CO)[C@@H](O)[C@H](O)[C@H]4O)cccc3C(=O)c2c1 |
| 2150 | Cc1cc([O-])c2C(=O)c3c(O[C@@H]4O[C@H](CO)[C@@H](O)[C@H](O)[C@H]4O)cccc3C(=O)c2c1 |
| 2151 | Cc1cc(O[C@@H]2O[C@H](CO)[C@@H](O)[C@H](O)[C@H]2O)c3C(=O)c4c(O)cccc4C(=O)c3c1 |
| 2152 | Cc1cc(O[C@@H]2O[C@H](CO)[C@@H](O)[C@H](O)[C@H]2O)c3C(=O)c4c([O-])cccc4C(=O)c3c1 |
| 2153 | Cc1cc(O)c2C(=O)c3c(O[C@@H]4O[C@H](COC(=O)CC(=O)[O-])[C@@H](O)[C@H](O)[C@H]4O)cccc3C(=O)c2c1 |
| 2154 | Cc1cc([O-])c2C(=O)c3c(O[C@@H]4O[C@H](COC(=O)CC(=O)[O-])[C@@H](O)[C@H](O)[C@H]4O)cccc3C(=O)c2c1 |
| 2155 | O[C@H]1[C@H](O)[C@@H](COC(=O)CC[N+](=O)[O-])O[C@@H](OC(=O)CC[N+](=O)[O-])[C@@H]1O |
| 2156 | OC[C@H]1O[C@@H](OC(=O)c2cc(O)c(O)c(O)c2)[C@H](OC(=O)\C=C\c3ccccc3)[C@@H](O)[C@@H]1O |
| 2157 | OC[C@H]1O[C@@H](OC(=O)c2cc(O)c([O-])c(O)c2)[C@H](OC(=O)\C=C\c3ccccc3)[C@@H](O)[C@@H]1O |
| 2158 | OC[C@H]1O[C@@H](O)[C@H](OC(=O)\C=C\c2ccccc2)[C@@H](O)[C@@H]1O |
| 2159 | COc1ccc(\C=C\c2cc(O)cc(O[C@@H]3O[C@H](CO)[C@@H](O)[C@H](O)[C@H]3O)c2)cc1 |
| 2160 | COc1ccc(\C=C\c2cc([O-])cc(O[C@@H]3O[C@H](CO)[C@@H](O)[C@H](O)[C@H]3O)c2)cc1 |
| 2161 | O[C@H]1[C@H](O)[C@@H](OC(=O)\C=C\c2ccccc2)[C@H](OC(=O)c3cc(O)c(O)c(O)c3)O[C@@H]1COC(=O)c4cc(O)c(O)c(O)c4 |
| 2162 | O[C@H]1[C@H](O)[C@@H](OC(=O)\C=C\c2ccccc2)[C@H](OC(=O)c3cc(O)c([O-])c(O)c3)O[C@@H]1COC(=O)c4cc(O)c(O)c(O)c4 |
| 2163 | O[C@H]1[C@H](O)[C@@H](OC(=O)\C=C\c2ccccc2)[C@H](OC(=O)c3cc(O)c([O-])c(O)c3)O[C@@H]1COC(=O)c4cc(O)c([O-])c(O)c4 |
| 2164 | O[C@H]1[C@H](O)[C@@H](COC(=O)c2cc(O)c(O)c(O)c2)O[C@@H](OC(=O)c3cc(O)c(O)c(O)c3)[C@@H]1O |
| 2165 | O[C@H]1[C@H](O)[C@@H](COC(=O)c2cc(O)c(O)c(O)c2)O[C@@H](OC(=O)c3cc(O)c([O-])c(O)c3)[C@@H]1O |
| 2166 | O[C@H]1[C@H](O)[C@@H](COC(=O)c2cc(O)c([O-])c(O)c2)O[C@@H](OC(=O)c3cc(O)c([O-])c(O)c3)[C@@H]1O |
| 2167 | OC[C@H]1[C@H]2CC=C(CO)[C@H]2[C@H](O)OC1=O |
| 2168 | CO[C@@H]1OC(=O)[C@@H](CO)[C@H]2CC=C(CO)[C@@H]12 |
| 2169 | CO[C@@H]1OC(=O)[C@H](CO)[C@H]2CC=C(CO)[C@@H]12 |
| 2170 | CC1=CC(=O)c2c(C)cc(O)cc2O1 |
| 2171 | CC1=CC(=O)c2c(C)cc([O-])cc2O1 |
| 2172 | Cc1cc(O)c2C(=O)c3c(O[C@@H]4O[C@H](CO)[C@@H](O)[C@H](O)[C@H]4O)cc(O)cc3C(=O)c2c1 |
| 2173 | Cc1cc([O-])c2C(=O)c3c(O[C@@H]4O[C@H](CO)[C@@H](O)[C@H](O)[C@H]4O)cc(O)cc3C(=O)c2c1 |
| 2174 | Cc1cc([O-])c2C(=O)c3c(O[C@@H]4O[C@H](CO)[C@@H](O)[C@H](O)[C@H]4O)cc([O-])cc3C(=O)c2c1 |
| 2175 | O[C@H]1Cc2c(O)cc(O)cc2O[C@@H]1c3ccc(O)c(O)c3 |
| 2176 | Oc1cc(O)c2C[C@@H](OC(=O)c3cc(O)c(O)c(O)c3)[C@H](Oc2c1)c4ccc(O)c(O)c4 |
| 2177 | Oc1cc(O)c2C[C@@H](OC(=O)c3cc(O)c([O-])c(O)c3)[C@H](Oc2c1)c4ccc(O)c(O)c4 |
| 2178 | O[C@H]1[C@H](O)[C@@H](COC(=O)c2cc(O)c(O)c(O)c2)O[C@@H](Oc3c(O)c(O)ccc3C(=O)[O-])[C@@H]1O |
| 2179 | O[C@H]1[C@H](O)[C@@H](COC(=O)c2cc(O)c([O-])c(O)c2)O[C@@H](Oc3c(O)c(O)ccc3C(=O)[O-])[C@@H]1O |
| 2180 | O[C@H]1[C@H](O)[C@@H](COC(=O)c2cc(O)c([O-])c(O)c2)O[C@@H](Oc3c([O-])c(O)ccc3C(=O)[O-])[C@@H]1O |
| 2181 | O[C@H]1[C@H](O)[C@@H](COC(=O)c2cc(O)c(O)c(O)c2)O[C@@H](Oc3c(O)cc(cc3O)C(=O)[O-])[C@@H]1O |
| 2182 | O[C@H]1[C@H](O)[C@@H](COC(=O)c2cc(O)c(O)c(O)c2)O[C@@H](Oc3c(O)cc(cc3[O-])C(=O)[O-])[C@@H]1O |
| 2183 | O[C@H]1[C@H](O)[C@@H](COC(=O)c2cc(O)c([O-])c(O)c2)O[C@@H](Oc3c(O)cc(cc3[O-])C(=O)[O-])[C@@H]1O |
| 2184 | O[C@@H]1O[C@H](COC(=O)c2cc(O)c(O)c(O)c2)[C@@H](O)[C@H](O)[C@H]1O |
| 2185 | O[C@@H]1O[C@H](COC(=O)c2cc(O)c([O-])c(O)c2)[C@@H](O)[C@H](O)[C@H]1O |
| 2186 | OC[C@@H](O)COC(=O)c1cc(O)c(O)c([O-])c1 |
| 2187 | Oc1ccc(\C=C\c2cc([O-])c3[C@@H]([C@H](Oc3c2)c4ccc(O)cc4)c5cc(O)cc([O-])c5)cc1 |
| 2188 | Oc1ccc(\C=C\c2cc([O-])c3[C@@H]([C@H](Oc3c2)c4ccc([O-])cc4)c5cc(O)cc([O-])c5)cc1 |
| 2189 | Cc1cc([O-])c2C(=O)c3c(O[C@@H]4O[C@H](CO)[C@@H](O)[C@H](O)[C@H]4O)cccc3[C@](O)([C@@H]5O[C@H](CO)[C@@H](O)[C@H](O)[C@H]5O)c2c1 |
| 2190 | Cc1cc([O-])c2C(=O)c3c(O[C@@H]4O[C@H](CO)[C@@H](O)[C@H](O)[C@H]4O)cccc3[C@@](O)([C@H]5O[C@H](CO)[C@@H](O)[C@H](O)[C@H]5O)c2c1 |
| 2191 | C[C@H](O)CC1=CC(=O)c2c(C)cc(O)cc2O1 |
| 2192 | C[C@H](O)CC1=CC(=O)c2c(C)cc([O-])cc2O1 |
| 2193 | CO[C@@H]1OC(=O)[C@@H](CO)[C@H]2CC[C@](C)(O)[C@@H]12 |
| 2194 | CO[C@@H]1OC(=O)[C@H](CO)[C@H]2CC[C@](C)(O)[C@@H]12 |
| 2195 | CO[C@H]1OC(=O)[C@@H](CO)[C@H]2CC[C@](C)(O)[C@@H]12 |
| 2196 | CC(=O)c1c(C)cc2cc(O)cc(O[C@@H]3O[C@H](CO)[C@@H](O)[C@H](O)[C@H]3O)c2c1O |
| 2197 | CC(=O)c1c(C)cc2cc([O-])cc(O[C@@H]3O[C@H](CO)[C@@H](O)[C@H](O)[C@H]3O)c2c1O |
| 2198 | C[C@@H](CCc1ccc(O)cc1)O[C@@H]2O[C@H](COC(=O)C)[C@@H](O)[C@H](O)[C@H]2O |
| 2199 | COc1cc(cc(OC)c1O[C@@H]2O[C@@H](C)[C@H](O)[C@@H](O)[C@H]2O)C(=O)OC[C@H]3O[C@@H](O[C@@H](C)CCc4ccc(O)cc4)[C@H](O)[C@@H](O)[C@@H]3O |
| 2200 | C[C@@H](CCc1ccc(O)cc1)O[C@@H]2O[C@H](COC(=O)c3cc(O)c(O)c(O)c3)[C@@H](O)[C@H](O)[C@H]2O |
| 2201 | C[C@@H](CCc1ccc(O)cc1)O[C@@H]2O[C@H](COC(=O)c3cc(O)c([O-])c(O)c3)[C@@H](O)[C@H](O)[C@H]2O |
| 2202 | C[C@@H](CCc1ccc(O)cc1)OS(=O)(=O)[O-] |
| 2203 | CC(=O)CCc1ccc(O[C@@H]2O[C@H](CO)[C@@H](O)[C@H](O)[C@H]2OC(=O)c3cc(O)c(O)c(O)c3)cc1 |
| 2204 | CC(=O)CCc1ccc(O[C@@H]2O[C@H](CO)[C@@H](O)[C@H](O)[C@H]2OC(=O)c3cc(O)c([O-])c(O)c3)cc1 |
| 2205 | CC(=O)CCc1ccc(O[C@@H]2O[C@H](COC(=O)c3cc(O)c(O)c(O)c3)[C@@H](O)[C@H](O)[C@H]2O)cc1 |
| 2206 | CC(=O)CCc1ccc(O[C@@H]2O[C@H](COC(=O)c3cc(O)c([O-])c(O)c3)[C@@H](O)[C@H](O)[C@H]2O)cc1 |
| 2207 | Oc1ccc(C[C@@]2(O)Oc3cc(O)cc(O)c3C2=O)cc1 |
| 2208 | Oc1ccc(C[C@]2(O)Oc3cc(O)cc([O-])c3C2=O)cc1 |
| 2209 | Oc1ccc(cc1)[C@@H]2Oc3ccc(\C=C\c4cc(O)cc(O)c4)cc3[C@H]2c5cc(O)cc([O-])c5 |
| 2210 | Oc1cc(O)cc(\C=C\c2ccc3O[C@H]([C@H](c4cc(O)cc([O-])c4)c3c2)c5ccc([O-])cc5)c1 |
| 2211 | Oc1cc([O-])cc(\C=C\c2ccc3O[C@H]([C@H](c4cc(O)cc([O-])c4)c3c2)c5ccc([O-])cc5)c1 |
| 2212 | Oc1ccc(cc1)[C@@H]2Oc3ccc(\C=C/c4cc(O)cc(O)c4)cc3[C@@H]2c5cc(O)cc([O-])c5 |
| 2213 | Oc1cc(O)cc(\C=C/c2ccc3O[C@H]([C@@H](c4cc(O)cc([O-])c4)c3c2)c5ccc([O-])cc5)c1 |
| 2214 | Oc1cc([O-])cc(\C=C/c2ccc3O[C@H]([C@@H](c4cc(O)cc([O-])c4)c3c2)c5ccc([O-])cc5)c1 |
| 2215 | C[C@H]1CC(=O)c2c(CC(=O)[O-])cc(O)cc2O1 |
| 2216 | CC1=CC(=O)c2c(CC(=O)[O-])cc(O)cc2O1 |
| 2217 | CC1=CC(=O)c2c(CC(=O)[O-])cc([O-])cc2O1 |
| 2218 | CC(=O)c1c(C)cc2cccc(O[C@@H]3O[C@H](CO)[C@@H](O)[C@H](O)[C@H]3O)c2c1O |
| 2219 | [O-]C(=O)C(=O)[O-] |
| 2220 | Cc1cc([O-])c2C(=O)c3c([O-])cc(O)cc3[C@H]([C@@H]4c5cccc([O-])c5C(=O)c6c(O)cc(CO)cc46)c2c1 |
| 2221 | Cc1cc([O-])c2C(=O)c3c([O-])cc(O)cc3[C@H]([C@@H]4c5cccc([O-])c5C(=O)c6c([O-])cc(CO)cc46)c2c1 |
| 2222 | Cc1cc([O-])c2C(=O)c3c([O-])cccc3[C@H]([C@@H]4c5cccc([O-])c5C(=O)c6c(O)cc(CO)cc46)c2c1 |
| 2223 | Cc1cc([O-])c2C(=O)c3c([O-])cccc3[C@H]([C@@H]4c5cccc([O-])c5C(=O)c6c([O-])cc(CO)cc46)c2c1 |
| 2224 | Cc1cc([O-])c2C(=O)c3c([O-])cccc3[C@H]([C@@H]4c5cc(C)cc([O-])c5C(=O)c6c([O-])cc(O)cc46)c2c1 |
| 2225 | CC(=O)CCc1ccc(O[C@@H]2O[C@H](CO)[C@@H](O)[C@H](O)[C@H]2O)cc1 |
| 2226 | COc1cc(O[C@@H]2O[C@H](CO[C@@H]3O[C@H](CO)[C@@H](O)[C@H](O)[C@H]3O)[C@@H](O)[C@H](O)[C@H]2O)c4C(=O)c5c(O)cc(C)cc5C(=O)c4c1 |
| 2227 | COc1cc(O[C@@H]2O[C@H](CO[C@@H]3O[C@H](CO)[C@@H](O)[C@H](O)[C@H]3O)[C@@H](O)[C@H](O)[C@H]2O)c4C(=O)c5c([O-])cc(C)cc5C(=O)c4c1 |
| 2228 | COc1cc(O)c2C(=O)c3c(O[C@@H]4O[C@H](CO[C@@H]5O[C@H](CO)[C@@H](O)[C@H](O)[C@H]5O)[C@@H](O)[C@H](O)[C@H]4O)cc(C)cc3C(=O)c2c1 |
| 2229 | COc1cc([O-])c2C(=O)c3c(O[C@@H]4O[C@H](CO[C@@H]5O[C@H](CO)[C@@H](O)[C@H](O)[C@H]5O)[C@@H](O)[C@H](O)[C@H]4O)cc(C)cc3C(=O)c2c1 |
| 2230 | COc1cc(O[C@@H]2O[C@H](CO)[C@@H](O)[C@H](O)[C@H]2O)c3C(=O)c4c(O)cc(C)cc4C(=O)c3c1 |
| 2231 | COc1cc(O[C@@H]2O[C@H](CO)[C@@H](O)[C@H](O)[C@H]2O)c3C(=O)c4c([O-])cc(C)cc4C(=O)c3c1 |
| 2232 | Oc1cc(O)cc(\C=C\c2ccc(O)c(O)c2)c1 |
| 2233 | Oc1cc([O-])cc(\C=C\c2ccc(O)c(O)c2)c1 |
| 2234 | O[C@@H]1[C@@H](COC(=O)c2cc(O)c(O)c(O)c2)O[C@@H](Oc3cc(\C=C\c4cc(O)cc(O)c4)ccc3O)[C@H](O)[C@H]1O |
| 2235 | O[C@@H]1[C@@H](COC(=O)c2cc(O)c([O-])c(O)c2)O[C@@H](Oc3cc(\C=C\c4cc(O)cc(O)c4)ccc3O)[C@H](O)[C@H]1O |
| 2236 | O[C@@H]1[C@@H](COC(=O)c2cc(O)c([O-])c(O)c2)O[C@@H](Oc3cc(\C=C\c4cc(O)cc([O-])c4)ccc3O)[C@H](O)[C@H]1O |
| 2237 | O[C@@H]1[C@@H](COC(=O)c2cc(O)c(O)c(O)c2)O[C@@H](Oc3ccc(\C=C\c4cc(O)cc(O)c4)cc3O)[C@H](O)[C@H]1O |
| 2238 | O[C@@H]1[C@@H](COC(=O)c2cc(O)c([O-])c(O)c2)O[C@@H](Oc3ccc(\C=C\c4cc(O)cc(O)c4)cc3O)[C@H](O)[C@H]1O |
| 2239 | O[C@@H]1[C@@H](COC(=O)c2cc(O)c([O-])c(O)c2)O[C@@H](Oc3ccc(\C=C\c4cc(O)cc([O-])c4)cc3O)[C@H](O)[C@H]1O |
| 2240 | OC[C@H]1O[C@@H](Oc2ccc(\C=C\c3cc(O)cc(O)c3)cc2O)[C@H](O)[C@@H](O)[C@@H]1O |
| 2241 | OC[C@H]1O[C@@H](Oc2ccc(\C=C\c3cc(O)cc([O-])c3)cc2O)[C@H](O)[C@@H](O)[C@@H]1O |
| 2242 | O[C@@H]1CO[C@@H](Oc2cc(\C=C\c3cc(O)cc(O)c3)ccc2O)[C@H](O)[C@H]1O |
| 2243 | O[C@@H]1CO[C@@H](Oc2cc(\C=C\c3cc(O)cc([O-])c3)ccc2O)[C@H](O)[C@H]1O |
| 2244 | OC[C@H]1O[C@@H](Oc2cc(O)cc(\C=C\c3ccc(O)cc3)c2)[C@H](O)[C@@H](O)[C@@H]1O |
| 2245 | OC[C@H]1O[C@@H](Oc2cc([O-])cc(\C=C\c3ccc(O)cc3)c2)[C@H](O)[C@@H](O)[C@@H]1O |
| 2246 | O[C@H]1Cc2c(O)cc(O)c([C@@H]3[C@@H](OC(=O)c4cc(O)c(O)c(O)c4)[C@H](Oc5cc(O)cc([O-])c35)c6ccc(O)c(O)c6)c2O[C@@H]1c7ccc(O)c(O)c7 |
| 2247 | O[C@H]1Cc2c(O)cc([O-])c([C@@H]3[C@@H](OC(=O)c4cc(O)c(O)c(O)c4)[C@H](Oc5cc(O)cc([O-])c35)c6ccc(O)c(O)c6)c2O[C@@H]1c7ccc(O)c(O)c7 |
| 2248 | O[C@H]1Cc2c(O)cc([O-])c([C@@H]3[C@@H](OC(=O)c4cc(O)c([O-])c(O)c4)[C@H](Oc5cc(O)cc([O-])c35)c6ccc(O)c(O)c6)c2O[C@@H]1c7ccc(O)c(O)c7 |
| 2249 | OC[C@H]1O[C@H]([C@H](O)[C@@H](O)[C@@H]1O)c2c(O)cc3O[C@@H]([C@H](O)[C@@H](c4c(O)cc(O)c5C[C@H](O)[C@H](Oc45)c6ccc(O)c(O)c6)c3c2O)c7ccc(O)c(O)c7 |
| 2250 | OC[C@H]1O[C@H]([C@H](O)[C@@H](O)[C@@H]1O)c2c(O)cc3O[C@@H]([C@H](O)[C@H](c3c2O)c4c([O-])cc(O)c5C[C@H](O)[C@H](Oc45)c6ccc(O)c(O)c6)c7ccc(O)c(O)c7 |
| 2251 | OC[C@H]1O[C@H]([C@H](O)[C@@H](O)[C@@H]1O)c2c(O)cc3O[C@@H]([C@H](O)[C@H](c3c2[O-])c4c([O-])cc(O)c5C[C@H](O)[C@H](Oc45)c6ccc(O)c(O)c6)c7ccc(O)c(O)c7 |
| 2252 | Oc1cc([O-])c2[C@@H]([C@@H](OC(=O)c3cc(O)c(O)c(O)c3)[C@H](Oc2c1)c4ccc(O)c(O)c4)c5c(O)cc(O)c6C[C@@H](OC(=O)c7cc(O)c(O)c(O)c7)[C@H](Oc56)c8ccc(O)c(O)c8 |
| 2253 | Oc1cc([O-])c2[C@@H]([C@@H](OC(=O)c3cc(O)c(O)c(O)c3)[C@H](Oc2c1)c4ccc(O)c(O)c4)c5c([O-])cc(O)c6C[C@@H](OC(=O)c7cc(O)c(O)c(O)c7)[C@H](Oc56)c8ccc(O)c(O)c8 |
| 2254 | Oc1cc([O-])c2[C@@H]([C@@H](OC(=O)c3cc(O)c(O)c(O)c3)[C@H](Oc2c1)c4ccc(O)c(O)c4)c5c([O-])cc(O)c6C[C@@H](OC(=O)c7cc(O)c([O-])c(O)c7)[C@H](Oc56)c8ccc(O)c(O)c8 |
| 2255 | Oc1cc([O-])c2[C@@H]([C@@H](OC(=O)c3cc(O)c([O-])c(O)c3)[C@H](Oc2c1)c4ccc(O)c(O)c4)c5c([O-])cc(O)c6C[C@@H](OC(=O)c7cc(O)c(O)c(O)c7)[C@H](Oc56)c8ccc(O)c(O)c8 |
| 2256 | Oc1cc([O-])c2[C@@H]([C@@H](OC(=O)c3cc(O)c([O-])c(O)c3)[C@H](Oc2c1)c4ccc(O)c(O)c4)c5c([O-])cc(O)c6C[C@@H](OC(=O)c7cc(O)c([O-])c(O)c7)[C@H](Oc56)c8ccc(O)c(O)c8 |
| 2257 | Oc1ccc(\C=C\c2cc(O)cc(O)c2)cc1 |
| 2258 | Oc1ccc(\C=C\c2cc(O)cc([O-])c2)cc1 |
| 2259 | O[C@@H]1[C@@H](COC(=O)c2cc(O)c(O)c(O)c2)O[C@@H](Oc3ccc(\C=C\c4cc(O)cc(O)c4)cc3)[C@H](O)[C@H]1O |
| 2260 | O[C@@H]1[C@@H](COC(=O)c2cc(O)c([O-])c(O)c2)O[C@@H](Oc3ccc(\C=C\c4cc(O)cc(O)c4)cc3)[C@H](O)[C@H]1O |
| 2261 | O[C@@H]1[C@@H](COC(=O)c2cc(O)c([O-])c(O)c2)O[C@@H](Oc3ccc(\C=C\c4cc(O)cc([O-])c4)cc3)[C@H](O)[C@H]1O |
| 2262 | OC[C@H]1O[C@@H](Oc2ccc(\C=C\c3cc(O)cc(O)c3)cc2)[C@H](O)[C@@H](O)[C@@H]1O |
| 2263 | OC[C@H]1O[C@@H](Oc2ccc(\C=C\c3cc(O)cc([O-])c3)cc2)[C@H](O)[C@@H](O)[C@@H]1O |
| 2264 | CCCCCCCC\C=C\CCCCCCCC(=O)O[C@@H]1c2cc(C)cc(O)c2C(=O)c3c(O)cc(OC)cc13 |
| 2265 | CCCCCCCC\C=C\CCCCCCCC(=O)O[C@@H]1c2cc(C)cc(O)c2C(=O)c3c([O-])cc(OC)cc13 |
| 2266 | CCCCCCCC\C=C\CCCCCCCC(=O)O[C@@H]1c2cc(C)cc([O-])c2C(=O)c3c([O-])cc(OC)cc13 |
| 2267 | CCCCCCCCCCCCCCCCCCCCCCCCCCCC(=O)O[C@@H]1c2cccc(O)c2C(=O)c3c(O)cc(C)cc13 |
| 2268 | CCCCCCCCCCCCCCCCCCCCCCCCCCCC(=O)O[C@@H]1c2cccc(O)c2C(=O)c3c([O-])cc(C)cc13 |
| 2269 | CCCCCCCCCCCCCCCCCCCCCCCCCCCC(=O)O[C@H]1c2cccc([O-])c2C(=O)c3c([O-])cc(C)cc13 |
| 2270 | CCCCCCCCCCCCCCCCCCCCCCOc1cc(O)c2C(=O)c3c(O)cc(C)cc3C(=O)c2c1 |
| 2271 | CCCCCCCCCCCCCCCCCCCCCCOc1cc([O-])c2C(=O)c3c(O)cc(C)cc3C(=O)c2c1 |
| 2272 | CCCCCCCCCCCCCCCCCCCCCCOc1cc([O-])c2C(=O)c3c([O-])cc(C)cc3C(=O)c2c1 |
| 2273 | CCCCCCCCCCCCCCCCCCO[C@]1(O)c2cc(O)cc(O)c2C(=O)c3c(O)cc(CO)cc13 |
| 2274 | CCCCCCCCCCCCCCCCCCO[C@]1(O)c2cc(O)cc([O-])c2C(=O)c3c(O)cc(CO)cc13 |
| 2275 | COc1ccc(\C=C\c2cc(O)cc(O[C@@H]3O[C@H](CO)[C@@H](O)[C@H](O)[C@H]3O)c2)cc1O |
| 2276 | COc1ccc(\C=C\c2cc([O-])cc(O[C@@H]3O[C@H](CO)[C@@H](O)[C@H](O)[C@H]3O)c2)cc1O |
| 2277 | OC[C@H]1O[C@@H](O)[C@H](OC(=O)\C=C\c2ccc(O)cc2)[C@@H](O)[C@@H]1O |
| 2278 | OC[C@H]1O[C@@H](O)[C@H](OC(=O)c2cc(O)c(O)c(O)c2)[C@@H](O)[C@@H]1O |
| 2279 | OC[C@H]1O[C@@H](O)[C@H](OC(=O)c2cc(O)c([O-])c(O)c2)[C@@H](O)[C@@H]1O |
| 2280 | COc1ccc(\C=C\c2cc(O)cc(O[C@@H]3O[C@H](COC(=O)c4cc(O)c(O)c(O)c4)[C@@H](O)[C@H](O)[C@H]3O)c2)cc1O |
| 2281 | COc1ccc(\C=C\c2cc(O)cc(O[C@@H]3O[C@H](COC(=O)c4cc(O)c([O-])c(O)c4)[C@@H](O)[C@H](O)[C@H]3O)c2)cc1O |
| 2282 | COc1ccc(\C=C\c2cc([O-])cc(O[C@@H]3O[C@H](COC(=O)c4cc(O)c([O-])c(O)c4)[C@@H](O)[C@H](O)[C@H]3O)c2)cc1O |
| 2283 | COc1ccc(\C=C\c2cc(O)cc(O)c2)cc1O |
| 2284 | COc1ccc(\C=C\c2cc(O)cc([O-])c2)cc1O |
| 2285 | COc1ccc(\C=C\c2cc(O)cc(O)c2)cc1O[C@@H]3O[C@H](CO)[C@@H](O)[C@H](O)[C@H]3O |
| 2286 | COc1ccc(\C=C\c2cc(O)cc([O-])c2)cc1O[C@@H]3O[C@H](CO)[C@@H](O)[C@H](O)[C@H]3O |
| 2287 | Cc1cc([O-])c2C(=O)c3c([O-])cc(O)cc3[C@H]([C@@H]4c5cccc([O-])c5C(=O)c6c([O-])cc(cc46)C(=O)[O-])c2c1 |
| 2288 | Cc1cc([O-])c2C(=O)c3c([O-])cccc3[C@H]([C@@H]4c5cccc([O-])c5C(=O)c6c([O-])cc(cc46)C(=O)[O-])c2c1 |
| 2289 | COc1cc([O-])c2C(=O)c3c([O-])cc(C)cc3[C@H]([C@@H]4c5cccc([O-])c5C(=O)c6c([O-])cc(cc46)C(=O)[O-])c2c1 |
| 2290 | Oc1cccc2C(=O)c3cc(cc(O)c3C(=O)c12)C(=O)[O-] |
| 2291 | Oc1cccc2C(=O)c3cc(cc([O-])c3C(=O)c12)C(=O)[O-] |
| 2292 | [O-]C(=O)c1cc([O-])c2C(=O)c3c([O-])cccc3C(=O)c2c1 |
| 2293 | OC[C@H]1O[C@@H](Oc2cccc3C(=O)c4cc(cc(O[C@@H]5O[C@H](CO)[C@@H](O)[C@H](O)[C@H]5O)c4C(=O)c23)C(=O)[O-])[C@H](O)[C@@H](O)[C@@H]1O |
| 2294 | OC[C@H]1O[C@@H](Oc2cccc3C(=O)c4cc(cc(O)c4C(=O)c23)C(=O)[O-])[C@H](O)[C@@H](O)[C@@H]1O |
| 2295 | OC[C@H]1O[C@@H](Oc2cccc3C(=O)c4cc(cc([O-])c4C(=O)c23)C(=O)[O-])[C@H](O)[C@@H](O)[C@@H]1O |
| 2296 | OC[C@H]1O[C@@H](Oc2cccc3c2C(=O)c4c([O-])cc(cc4[C@@]3(O)[C@@H]5O[C@H](CO)[C@@H](O)[C@H](O)[C@H]5O)C(=O)[O-])[C@H](O)[C@@H](O)[C@@H]1O |
| 2297 | OC[C@H]1O[C@@H](Oc2cccc3c2C(=O)c4c([O-])cc(cc4[C@]3(O)[C@H]5O[C@H](CO)[C@@H](O)[C@H](O)[C@H]5O)C(=O)[O-])[C@H](O)[C@@H](O)[C@@H]1O |
| 2298 | OC[C@H]1O[C@@H](Oc2cccc3[C@H]([C@@H]4O[C@H](CO)[C@@H](O)[C@H](O)[C@H]4O)c5cc(cc([O-])c5C(=O)c23)C(=O)[O-])[C@H](O)[C@@H](O)[C@@H]1O |
| 2299 | OC[C@H]1O[C@@H](Oc2cccc3[C@@H]([C@@H]4O[C@H](CO)[C@@H](O)[C@H](O)[C@H]4O)c5cc(cc([O-])c5C(=O)c23)C(=O)[O-])[C@H](O)[C@@H](O)[C@@H]1O |
| 2300 | O[C@H]1[C@H](O)[C@@H](COC(=O)C(=O)[O-])O[C@@H](Oc2cccc3C(=O)c4cc(cc(O)c4C(=O)c23)C(=O)[O-])[C@@H]1O |
| 2301 | O[C@H]1[C@H](O)[C@@H](COC(=O)C(=O)[O-])O[C@@H](Oc2cccc3C(=O)c4cc(cc([O-])c4C(=O)c23)C(=O)[O-])[C@@H]1O |
| 2302 | OC1=COC(=O)[C@@H](C1)OC(=O)\C=C\c2cc(O)cc(O)c2 |
| 2303 | OC1=COC(=O)[C@H](C1)OC(=O)\C=C\c2cc(O)cc([O-])c2 |
| 2304 | [O-]C(=O)c1cc([O-])c2C(=O)c3c([O-])cccc3[C@@H]([C@@H]4c5cccc([O-])c5C(=O)c6c([O-])cc(cc46)C(=O)[O-])c2c1 |
| 2305 | [O-]C(=O)c1cc([O-])c2C(=O)c3c([O-])cccc3[C@H]([C@@H]4c5cccc([O-])c5C(=O)c6c([O-])cc(cc46)C(=O)[O-])c2c1 |
| 2306 | OCc1cc(O)c2C(=O)c3c([O-])cccc3[C@@H]([C@@H]4c5cccc([O-])c5C(=O)c6c([O-])cc(cc46)C(=O)[O-])c2c1 |
| 2307 | OCc1cc([O-])c2C(=O)c3c([O-])cccc3[C@@H]([C@@H]4c5cccc([O-])c5C(=O)c6c([O-])cc(cc46)C(=O)[O-])c2c1 |
| 2308 | OC[C@H]1O[C@@H](Oc2cccc3[C@@H]([C@@H]4c5cccc(O[C@@H]6O[C@H](CO)[C@@H](O)[C@H](O)[C@H]6O)c5C(=O)c7c([O-])cc(cc47)C(=O)[O-])c8cc(cc([O-])c8C(=O)c23)C(=O)[O-])[C@H](O)[C@@H](O)[C@@H]1O |
| 2309 | OC[C@H]1O[C@@H](Oc2cccc3[C@@H]([C@H]4c5cccc(O[C@@H]6O[C@H](CO)[C@@H](O)[C@H](O)[C@H]6O)c5C(=O)c7c([O-])cc(cc47)C(=O)[O-])c8cc(cc([O-])c8C(=O)c23)C(=O)[O-])[C@H](O)[C@@H](O)[C@@H]1O |
| 2310 | OC[C@H]1O[C@@H](Oc2cccc3[C@@H]([C@@H]4c5cccc(O[C@@H]6O[C@H](CO)[C@@H](O)[C@H](O)[C@H]6O)c5C(=O)c7c([O-])cc(cc47)C(=O)[O-])c8cc(CO)cc(O)c8C(=O)c23)[C@H](O)[C@@H](O)[C@@H]1O |
| 2311 | OC[C@H]1O[C@@H](Oc2cccc3[C@@H]([C@@H]4c5cccc(O[C@@H]6O[C@H](CO)[C@@H](O)[C@H](O)[C@H]6O)c5C(=O)c7c([O-])cc(cc47)C(=O)[O-])c8cc(CO)cc([O-])c8C(=O)c23)[C@H](O)[C@@H](O)[C@@H]1O |
| 2312 | OC[C@H]1O[C@@H](Oc2cccc3[C@H]([C@@H]4c5cccc(O[C@@H]6O[C@H](CO)[C@@H](O)[C@H](O)[C@H]6O)c5C(=O)c7c([O-])cc(cc47)C(=O)[O-])c8cc(CO)cc(O)c8C(=O)c23)[C@H](O)[C@@H](O)[C@@H]1O |
| 2313 | OC[C@H]1O[C@@H](Oc2cccc3[C@H]([C@@H]4c5cccc(O[C@@H]6O[C@H](CO)[C@@H](O)[C@H](O)[C@H]6O)c5C(=O)c7c([O-])cc(cc47)C(=O)[O-])c8cc(CO)cc([O-])c8C(=O)c23)[C@H](O)[C@@H](O)[C@@H]1O |
| 2314 | Cc1cc(O)c2C(=O)c3c(O[C@@H]4O[C@@H](CO)[C@@H](O)[C@H](O)[C@@H]4O)cc(OS(=O)(=O)[O-])cc3C(=O)c2c1 |
| 2315 | Cc1cc([O-])c2C(=O)c3c(O[C@@H]4O[C@@H](CO)[C@@H](O)[C@H](O)[C@@H]4O)cc(OS(=O)(=O)[O-])cc3C(=O)c2c1 |
| 2316 | OC[C@H]1O[C@@H](Oc2cc(\C=C\c3cc(O)cc(O)c3)ccc2O)[C@H](O)[C@@H](O)[C@@H]1O |
| 2317 | OC[C@H]1O[C@@H](Oc2cc(\C=C\c3cc(O)cc([O-])c3)ccc2O)[C@H](O)[C@@H](O)[C@@H]1O |
| 2318 | COc1cc(O[C@@H]2O[C@H](CO)[C@@H](O)[C@H](O)[C@H]2O)c3c(O)c(C(=O)C)c(C)cc3c1 |
| 2319 | COc1cc(O[C@@H]2O[C@H](COC(=O)C(=O)[O-])[C@@H](O)[C@H](O)[C@H]2O)c3c(O)c(C(=O)C)c(C)cc3c1 |
| 2320 | COc1ccc(\C=C/c2cc(O)cc(O[C@@H]3O[C@H](CO)[C@@H](O)[C@H](O)[C@H]3OC(=O)c4cc(O)c(O)c(O)c4)c2)cc1O |
| 2321 | COc1ccc(\C=C/c2cc(O)cc(O[C@@H]3O[C@H](CO)[C@@H](O)[C@H](O)[C@H]3OC(=O)c4cc(O)c([O-])c(O)c4)c2)cc1O |
| 2322 | COc1ccc(\C=C/c2cc([O-])cc(O[C@@H]3O[C@H](CO)[C@@H](O)[C@H](O)[C@H]3OC(=O)c4cc(O)c([O-])c(O)c4)c2)cc1O |
| 2323 | COc1ccc(\C=C/c2cc(O)cc(O[C@@H]3O[C@H](COC(=O)c4cc(O)c(O)c(O)c4)[C@@H](O)[C@H](O)[C@H]3O)c2)cc1O |
| 2324 | COc1ccc(\C=C/c2cc(O)cc(O[C@@H]3O[C@H](COC(=O)c4cc(O)c([O-])c(O)c4)[C@@H](O)[C@H](O)[C@H]3O)c2)cc1O |
| 2325 | COc1ccc(\C=C/c2cc([O-])cc(O[C@@H]3O[C@H](COC(=O)c4cc(O)c([O-])c(O)c4)[C@@H](O)[C@H](O)[C@H]3O)c2)cc1O |
| 2326 | COc1ccc(\C=C/c2cc(O)cc(O[C@@H]3O[C@H](CO)[C@@H](O)[C@H](O)[C@H]3O)c2)cc1O |
| 2327 | COc1ccc(\C=C/c2cc([O-])cc(O[C@@H]3O[C@H](CO)[C@@H](O)[C@H](O)[C@H]3O)c2)cc1O |
| 2328 | COc1c(O)cc2CC[NH2+][C@@H]3Cc4ccccc4c1c23 |
| 2329 | COc1ccc2cc3c4cc5OCOc5cc4CC[n+]3cc2c1OC |
| 2330 | CC(=C)[C@@H]1CC[C@]2(CO)CC[C@]3(C)[C@H](CC[C@@H]4[C@@]5(C)CC[C@H](O)C(C)(C)[C@@H]5CC[C@@]34C)[C@@H]12 |
| 2331 | CC(=C)[C@@H]1CC[C@@]2(CC[C@]3(C)[C@H](CC[C@@H]4[C@@]5(C)CCC(=O)C(C)(C)[C@@H]5CC[C@@]34C)[C@@H]12)C(=O)O |
| 2332 | CC(=C)[C@@H]1CC[C@@]2(CC[C@]3(C)[C@H](CC[C@@H]4[C@@]5(C)CCC(=O)C(C)(C)[C@@H]5CC[C@@]34C)[C@@H]12)C(=O)[O-] |
| 2333 | CC(=C)[C@@H]1CC[C@@]2(CC[C@]3(C)[C@@H](CC[C@H]4[C@@]5(C)[C@H](C=O)[C@@H](O)C(C)(C)[C@H]5CC[C@@]34C)[C@@H]12)C(=O)O |
| 2334 | CC(=C)[C@@H]1CC[C@@]2(CC[C@]3(C)[C@@H](CC[C@H]4[C@@]5(C)[C@H](C=O)[C@@H](O)C(C)(C)[C@H]5CC[C@@]34C)[C@@H]12)C(=O)[O-] |
| 2335 | CC(=C)[C@@H]1CC[C@@]2(CC[C@]3(C)[C@H](CC[C@@H]4[C@@]5(C)C[C@@H](O)[C@H](OC(=O)\C=C/c6ccc(O)cc6)C(C)(C)[C@@H]5CC[C@@]34C)[C@H]12)C(=O)O |
| 2336 | CC(=C)[C@@H]1CC[C@@]2(CC[C@]3(C)[C@H](CC[C@@H]4[C@@]5(C)C[C@@H](O)[C@H](OC(=O)\C=C/c6ccc(O)cc6)C(C)(C)[C@@H]5CC[C@@]34C)[C@H]12)C(=O)[O-] |
| 2337 | CC(=C)[C@@H]1CC[C@@]2(CC[C@]3(C)[C@H](CC[C@@H]4[C@@]5(C)C[C@@H](O)[C@H](OC(=O)\C=C\c6ccc(O)cc6)C(C)(C)[C@@H]5CC[C@@]34C)[C@H]12)C(=O)O |
| 2338 | CC(=C)[C@@H]1CC[C@@]2(CC[C@]3(C)[C@H](CC[C@@H]4[C@@]5(C)C[C@@H](O)[C@H](OC(=O)\C=C\c6ccc(O)cc6)C(C)(C)[C@@H]5CC[C@@]34C)[C@H]12)C(=O)[O-] |
| 2339 | CC1(C)CC[C@@]2(CC[C@]3(C)C(=CC[C@@H]4[C@@]5(C)C[C@@H](O)[C@H](OC(=O)\C=C/c6ccc(O)cc6)C(C)(C)[C@@H]5CC[C@@]34C)[C@@H]2C1)C(=O)O |
| 2340 | CC1(C)CC[C@@]2(CC[C@]3(C)C(=CC[C@@H]4[C@@]5(C)C[C@@H](O)[C@H](OC(=O)\C=C/c6ccc(O)cc6)C(C)(C)[C@@H]5CC[C@@]34C)[C@@H]2C1)C(=O)[O-] |
| 2341 | CC1(C)CC[C@@]2(CC[C@]3(C)C(=CC[C@@H]4[C@@]5(C)C[C@@H](O)[C@H](OC(=O)\C=C\c6ccc(O)cc6)C(C)(C)[C@@H]5CC[C@@]34C)[C@@H]2C1)C(=O)O |
| 2342 | CC1(C)CC[C@@]2(CC[C@]3(C)C(=CC[C@@H]4[C@@]5(C)C[C@@H](O)[C@H](OC(=O)\C=C\c6ccc(O)cc6)C(C)(C)[C@@H]5CC[C@@]34C)[C@@H]2C1)C(=O)[O-] |
| 2343 | COc1cc2OC(=CC(=O)c2c(O)c1[C@@H]3O[C@H](CO)[C@@H](O)[C@H](O)[C@H]3O[C@@H]4O[C@H](COC(=O)\C=C\c5ccc(O)cc5)[C@@H](O)[C@H](O)[C@H]4O)c6ccc(O)cc6 |
| 2344 | COc1cc2OC(=CC(=O)c2c(O)c1[C@@H]3O[C@H](CO)[C@@H](O)[C@H](O)[C@H]3O[C@@H]4O[C@H](COC(=O)\C=C\c5ccc(O)cc5)[C@@H](O)[C@H](O)[C@H]4O)c6ccc([O-])cc6 |
| 2345 | CC1(C)CC[C@@]2(CC[C@]3(C)C(=CC[C@@H]4[C@@]5(C)C[C@@H](O)[C@H](O)C(C)(C)[C@@H]5CC[C@@]34C)[C@@H]2C1)C(=O)O |
| 2346 | CC1(C)CC[C@@]2(CC[C@]3(C)C(=CC[C@@H]4[C@@]5(C)C[C@@H](O)[C@H](O)C(C)(C)[C@@H]5CC[C@@]34C)[C@@H]2C1)C(=O)[O-] |
| 2347 | COc1cc(\C=C\C(=O)OC[C@H]2O[C@@H](O[C@@H]3[C@@H](O)[C@H](O)[C@@H](CO)O[C@H]3c4c(O)c5C(=O)C=C(Oc5cc4OC)c6ccc(O)cc6)[C@H](O)[C@@H](O)[C@@H]2O)ccc1O |
| 2348 | COc1cc(\C=C\C(=O)OC[C@H]2O[C@@H](O[C@@H]3[C@@H](O)[C@H](O)[C@@H](CO)O[C@H]3c4c(O)c5C(=O)C=C(Oc5cc4OC)c6ccc([O-])cc6)[C@H](O)[C@@H](O)[C@@H]2O)ccc1O |
| 2349 | C[C@@H]1O[C@@H](O[C@H]2[C@H](O[C@H]3CC[C@@]4(C)[C@@H](CC[C@]5(C)[C@@H]4CC[C@@H]6[C@H]7[C@@](C)(O)[C@@H](CC=C(C)C)O[C@@]78C[C@@]56CO8)C3(C)C)O[C@H](CO)[C@H](O)[C@@H]2O[C@@H]9O[C@H](CO)[C@@H](O)[C@H](O)[C@H]9O)[C@H](O)[C@H](O)[C@H]1O |
| 2350 | C[C@@H]1O[C@@H](O[C@H]2[C@H](O[C@H]3CC[C@@]4(C)[C@@H](CC[C@]5(C)[C@@H]4CC[C@@H]6[C@H]7[C@@](C)(O)[C@@H](CC=C(C)C)O[C@@]78C[C@@]56CO8)C3(C)C)O[C@H](CO)[C@@H](O)[C@@H]2O[C@@H]9O[C@H](CO)[C@@H](O)[C@H](O)[C@H]9O)[C@H](O)[C@H](O)[C@H]1O |
| 2351 | C[C@@H]1O[C@@H](O[C@@H]2[C@@H](O)[C@@H](O)[C@@H](CO)O[C@H]2O[C@H]3CC[C@@]4(C)[C@@H](CC[C@]5(C)[C@@H]4CC[C@@H]6[C@H]7[C@@]8(C)O[C@H](C[C@H]8O[C@@]79C[C@@]56CO9)C(C)(C)O)C3(C)C)[C@H](O)[C@H](O)[C@H]1O |
| 2352 | C[C@@H]1O[C@@H](O[C@H]2[C@H](O[C@H]3CC[C@@]4(C)[C@@H](CC[C@]5(C)[C@@H]4CC[C@@H]6[C@H]7[C@@](C)(O)C[C@@H](O[C@@]78C[C@@]56CO8)C=C(C)C)C3(C)C)OC[C@H](O)[C@@H]2O[C@@H]9O[C@H](CO[C@@H]%10O[C@H](CO)[C@@H](O)[C@H](O)[C@H]%10O)[C@@H](O)[C@H](O)[C@H]9O[C@@H]%11OC[C@@H](O)[C@H](O)[C@H]%11O)[C@H](O)[C@H](O)[C@H]1O |
| 2353 | C[C@@H]1O[C@@H](O[C@H]2[C@H](O[C@H]3CC[C@@]4(C)[C@@H](CC[C@]5(C)[C@@H]4CC[C@@H]6[C@H]7[C@@](C)(O)C[C@@H](O[C@@]78C[C@@]56CO8)C=C(C)C)C3(C)C)OC[C@H](O)[C@@H]2O[C@@H]9O[C@H](CO)[C@@H](O)[C@H](O)[C@H]9O[C@@H]%10OC[C@@H](O)[C@H](O)[C@H]%10O)[C@H](O)[C@H](O)[C@H]1O |
| 2354 | C[C@H]1O[C@@H](O[C@H]2[C@H](O[C@H]3CC[C@@]4(C)[C@@H](CC[C@]5(C)[C@@H]4CC[C@@H]6[C@H]7[C@@](C)(O)C[C@H](O[C@@]78C[C@@]56CO8)C=C(C)C)C3(C)C)OC[C@H](O)[C@@H]2O[C@@H]9O[C@H](CO)[C@@H](O)[C@H](O)[C@H]9O[C@@H]%10OC[C@@H](O)[C@H](O)[C@H]%10O)[C@H](O)[C@@H](O)[C@H]1O |
| 2355 | O[C@H](CC(=O)[O-])C(=O)[O-] |
| 2356 | [O-]C(=O)c1cccnc1 |
| 2357 | CC1(C)CC[C@@]2(CC[C@]3(C)C(=CC[C@@H]4[C@@]5(C)CCC(=O)C(C)(C)[C@@H]5CC[C@@]34C)[C@@H]2C1)C(=O)O |
| 2358 | CC1(C)CC[C@@]2(CC[C@]3(C)C(=CC[C@@H]4[C@@]5(C)CCC(=O)C(C)(C)[C@@H]5CC[C@@]34C)[C@@H]2C1)C(=O)[O-] |
| 2359 | CN1CCc2cc3OCOc3cc2C(=O)Cc4ccc5OCOc5c4C1 |
| 2360 | Cc1cc2N=C3[C@@H](NC(=O)[N-]C3=O)[N@@H+](C[C@H](O)[C@H](O)[C@H](O)CO)c2cc1C |
| 2361 | Cc1cc2N=C3[C@H](NC(=O)[N-]C3=O)N(C[C@H](O)[C@H](O)[C@H](O)CO)c2cc1C |
| 2362 | COC1=CC2=C([C@@H](O)[C@@H]1[C@@H]3O[C@H](CO)[C@@H](O)[C@H](O)[C@H]3O[C@@H]4O[C@H](COC(=O)\C=C\c5cc(OC)c(O)c(OC)c5)[C@@H](O)[C@H](O)[C@H]4O)C(=O)C=C(O2)c6ccc(O)cc6 |
| 2363 | COC1=CC2=C([C@H](O)[C@H]1[C@@H]3O[C@H](CO)[C@@H](O)[C@H](O)[C@H]3O[C@@H]4O[C@H](COC(=O)\C=C\c5cc(OC)c(O)c(OC)c5)[C@@H](O)[C@H](O)[C@H]4O)C(=O)C=C(O2)c6ccc([O-])cc6 |
| 2364 | COc1cc2OC(=CC(=O)c2c(O)c1[C@@H]3O[C@H](CO)[C@@H](O)[C@H](O)[C@H]3O[C@@H]4O[C@H](CO)[C@@H](O)[C@H](O)[C@H]4O)c5ccc(O)cc5 |
| 2365 | COc1cc2OC(=CC(=O)c2c(O)c1[C@@H]3O[C@H](CO)[C@@H](O)[C@H](O)[C@H]3O[C@@H]4O[C@H](CO)[C@@H](O)[C@H](O)[C@H]4O)c5ccc([O-])cc5 |
| 2366 | COc1cc2CC[NH2+][C@@H]3CC4(C=CC(=O)C=C4)c(c1OC)c23 |
| 2367 | COc1cc2OC(=CC(=O)c2c(O)c1[C@@H]3O[C@H](CO)[C@@H](O)[C@H](O)[C@H]3O)c4ccc(O)cc4 |
| 2368 | COc1cc2OC(=CC(=O)c2c(O)c1[C@@H]3O[C@H](CO)[C@@H](O)[C@H](O)[C@H]3O)c4ccc([O-])cc4 |
| 2369 | O[C@H]([C@@H](O)C(=O)[O-])C(=O)[O-] |
| 2370 | Cc1ncc(C[n+]2csc(CCO)c2C)c(N)n1 |
| 2371 | C[C@@H](O)\C=C\[C@@]1(O)C(=CC(=O)CC1(C)C)C |
| 2372 | C[C@@H]1O[C@@H](O[C@H]2CO[C@@H](O[C@H]3CC[C@@]4(C)[C@@H](CC[C@]5(C)[C@@H]4CC[C@@H]6[C@H]7[C@](C)(C[C@@H](O[C@@]78C[C@@]56CO8)C=C(C)C)O[C@@H]9O[C@@H](C)[C@H](O)[C@@H](OC(=O)C)[C@H]9OC(=O)C)C3(C)C)[C@H](O)[C@H]2O)[C@H](O)[C@H](O)[C@H]1O |
| 2373 | CC(=C)[C@@H]1CC[C@@]2(CC[C@]3(C)[C@H](CC[C@@H]4[C@@]3(C)CC[C@H]5C(C)(C)C=C(C=O)[C@]45C)[C@@H]12)C(=O)O |
| 2374 | CC(=C)[C@@H]1CC[C@@]2(CC[C@]3(C)[C@H](CC[C@@H]4[C@@]3(C)CC[C@H]5C(C)(C)C=C(C=O)[C@]45C)[C@@H]12)C(=O)[O-] |
| 2375 | COc1ccc2C[C@@H]3c4c(CC[N+]3(C)C)cc(O)c(O)c4c2c1OC |
| 2376 | C[C@@H]1O[C@@H](O[C@H]2[C@H](O[C@H]3CC[C@@]4(C)[C@@H](CC[C@]5(C)[C@H]4CC[C@@H]6[C@H]7[C@@](C)(O)C[C@@H](O[C@@]78C[C@@]56CO8)C=C(C)C)C3(C)C)OC[C@H](O)[C@@H]2O[C@@H]9O[C@H](CO)[C@@H](O)[C@H](O)[C@H]9O)[C@H](O)[C@H](O)[C@@H]1O |
| 2377 | C[C@@H]1O[C@@H](O[C@H]2[C@H](O[C@H]3CC[C@@]4(C)[C@@H](CC[C@]5(C)[C@@H]4CC[C@@H]6[C@H]7[C@@](C)(O)C[C@@H](O[C@@]78C[C@@]56CO8)C=C(C)C)C3(C)C)OC[C@H](O)[C@@H]2O[C@@H]9O[C@H](CO)[C@@H](O)[C@H](O)[C@H]9O)[C@H](O)[C@H](O)[C@H]1O |
| 2378 | C[C@@H]1O[C@@H](O[C@H]2[C@H](O[C@H]3CC[C@@]4(C)[C@@H](CC[C@]5(C)[C@@H]4CC[C@@H]6[C@H]7[C@@](C)(O)C[C@@H](O[C@@]78C[C@@]56CO8)C=C(C)C)C3(C)C)OC[C@H](O)[C@@H]2O[C@@H]9O[C@H](CO)[C@@H](O)[C@H](O)[C@H]9O[C@@H]%10OC[C@@H](O)[C@H](O)[C@H]%10O)[C@H](O)[C@H](O)[C@@H]1O |
| 2379 | O[C@@H]1Cc2c(O)cc(O)cc2O[C@H]1c3ccc(O)c(O)c3 |
| 2380 | COc1cc(CC=C)cc2OCOc12 |
| 2381 | NC(=O)C[C@H]([NH3+])C(=O)[O-] |
| 2382 | COc1cc2CCN3Cc4c(C[C@@H]3c2cc1O)ccc(O)c4OC |
| 2383 | OC[C@@H](O)[C@@H](O)[C@H](O)[C@@H](O)C=O |
| 2384 | CC1=C(C=C)c2cc3nc(cc4[nH]c(cc5nc(cc1[nH]2)c(C)c5CCC(=O)[O-])c(CCC(=O)[O-])c4C)c(C=C)c3C |
| 2385 | O[C@@H](CC(=O)[O-])C(=O)[O-] |
| 2386 | COc1cc2CC[NH2+][C@@H](Cc3ccc(O)cc3)c2cc1O |
| 2387 | COc1cc2CCN[C@@H](Cc3ccc(O)cc3)c2cc1O |
| 2388 | [O-]C(=O)Cc1c[nH]c2ccccc12 |
| 2389 | O[C@@H]([C@H](O)C(=O)[O-])C(=O)[O-] |
| 2390 | COc1cc(\C=C\CO)cc(OC)c1O |
| 2391 | CCCCCCCCCCCCCCCC(=O)OCC1=C[C@H]2[C@H]3[C@@H](C[C@@H](C)[C@@]4(C=C(C)[C@H](O)[C@@]4(O)[C@@H]1O)C2=O)C3(C)C |
| 2392 | Cc1cc2N=C3C(=O)NC(=O)N=C3[N@H+](C[C@H](O)[C@H](O)[C@H](O)CO)c2cc1C |
| 2393 | Cc1cc2N=C3C(=O)NC(=O)N=C3N(C[C@H](O)[C@H](O)[C@H](O)CO)c2cc1C |
| 2394 | C[C@@H](O[C@@H]1O[C@H](CO)[C@@H](O)[C@H](O)[C@H]1O)\C=C\[C@@]2(O)C(=CC(=O)CC2(C)C)C |
| 2395 | C\C(=C/C=C/C=C(\C)/C=C/C=C(\C)/C=C/C1=C(C)CCCC1(C)C)\C=C\C=C(/C)\C=C\C2=C(C)CCCC2(C)C |
| 2396 | CC(=C[C@H]1C[C@](C)(O)[C@@H]2[C@H]3CC[C@@H]4[C@@]5(C)CC[C@H](O)C(C)(C)[C@@H]5CC[C@@]4(C)[C@@]36CO[C@@]2(C6)O1)C |
| 2397 | CO[C@@H]1[C@@H](O)[C@H](C)O[C@@H](O[C@@H]2CC[C@@]3(CO)[C@H](CC[C@@H]4[C@@H]3CC[C@]5(C)[C@@H](CC[C@]45O)C6=CC(=O)OC6)C2)[C@H]1O |
| 2398 | C[C@]12CC[C@H]3[C@@H](CC[C@@H]4C[C@H](O)CC[C@]34CO)[C@@]1(O)CC[C@@H]2C5=CC(=O)OC5 |
| 2399 | CC(=O)[C@H]1CC[C@]2(O)[C@]3(O)CC=C4C[C@@H](O)CC[C@]4(C)[C@@H]3C[C@@H](OC(=O)c5cccnc5)[C@]12C |
| 2400 | C[C@@H]1CC[C@@]2(CC[C@]3(C)C(=CC[C@@H]4[C@@]5(C)CC[C@H](O)C(C)(C)[C@@H]5CC[C@@]34C)[C@@H]2[C@]1(C)O)C(=O)O |
| 2401 | C[C@@H]1CC[C@@]2(CC[C@]3(C)C(=CC[C@@H]4[C@@]5(C)CC[C@H](O)C(C)(C)[C@@H]5CC[C@@]34C)[C@@H]2[C@]1(C)O)C(=O)[O-] |
| 2402 | COc1cc2ccnc3C(=O)c4ccccc4c(c1OC)c23 |
| 2403 | C[C@H]1CC[C@@H](O)[C@]2(CO)[C@@H](OC(=O)C)[C@H](OC(=O)c3ccccc3)[C@H]4C[C@@]12OC4(C)C |
| 2404 | C[C@@H]1[C@@H]2CC[C@]3(C)[C@@H]([C@H]2OC1=O)[C@](C)(O)C=CC3=O |
| 2405 | CC(C)(CO)[C@H](O)C(=O)NCCC(=O)[O-] |
| 2406 | C[C@@H]1CC[C@@]2(CC[C@]3(C)C(=CC[C@@H]4[C@@]5(C)C[C@@H](O)[C@H](O)C(C)(C)[C@@H]5CC[C@@]34C)[C@@H]2[C@H]1C)C(=O)O |
| 2407 | C[C@@H]1CC[C@@]2(CC[C@]3(C)C(=CC[C@@H]4[C@@]5(C)C[C@@H](O)[C@H](O)C(C)(C)[C@@H]5CC[C@@]34C)[C@@H]2[C@H]1C)C(=O)[O-] |
| 2408 | COc1cc2OC(=CC(=O)c2c(O)c1[C@@H]3O[C@@H](CO)[C@@H](O)[C@H](O)[C@@H]3O)c4ccc(O)cc4 |
| 2409 | COc1cc2OC(=CC(=O)c2c(O)c1[C@@H]3O[C@@H](CO)[C@@H](O)[C@H](O)[C@@H]3O)c4ccc([O-])cc4 |
| 2410 | COc1cc2CC[NH2+][C@H]3Cc4ccccc4c(c1OC)c23 |
| 2411 | COc1cc2CC[N@H+](C)[C@@H]3Cc4ccccc4c(c1OC)c23 |
| 2412 | COc1cc2CCN(C)[C@@H]3Cc4ccccc4c(c1OC)c23 |
| 2413 | CC(=C)[C@@H]1CC[C@@]2(CC[C@]3(C)C(=CC[C@@H]4[C@@]5(C)C[C@@H](O)[C@H](O)C(C)(C)[C@@H]5CC[C@@]34C)[C@@H]12)C(=O)O |
| 2414 | CC(=C)[C@@H]1CC[C@@]2(CC[C@]3(C)C(=CC[C@@H]4[C@@]5(C)C[C@@H](O)[C@H](O)C(C)(C)[C@@H]5CC[C@@]34C)[C@@H]12)C(=O)[O-] |
| 2415 | CCCCCCCCCCCCCCCCCCCCCC[C@@H](O)C(=O)N[C@@H](CO)[C@H](O)[C@H](O)CCC\C=C\CCCCCCCCC |
| 2416 | C[C@@H]1CC[C@@]2(CC[C@]3(C)C(=CC[C@@H]4[C@@]5(C)CCC(=O)C(C)(C)[C@@H]5CC[C@@]34C)[C@@H]2[C@H]1C)C(=O)O |
| 2417 | C[C@@H]1CC[C@@]2(CC[C@]3(C)C(=CC[C@@H]4[C@@]5(C)CCC(=O)C(C)(C)[C@@H]5CC[C@@]34C)[C@@H]2[C@H]1C)C(=O)[O-] |
| 2418 | CC(=C)[C@@H]1CC[C@@]2(CC[C@]3(C)[C@H](CC[C@@H]4[C@]5(C)[C@@H](CC[C@@]34C)C(C)(C)[C@@H](O)[C@@H]5C(=O)O)[C@@H]12)C(=O)O |
| 2419 | CC(=C)[C@@H]1CC[C@@]2(CC[C@]3(C)[C@H](CC[C@@H]4[C@]5(C)[C@@H](CC[C@@]34C)C(C)(C)[C@@H](O)[C@@H]5C(=O)O)[C@@H]12)C(=O)[O-] |
| 2420 | COc1cc(\C=C\C(=O)NCCc2ccc(O)cc2)ccc1O |
| 2421 | CCCCCC\C=C\CCCCCCCCCC(=O)[O-] |
| 2422 | OC[C@H]1O[C@@H](O[C@@H]2[C@@H](O)[C@H](O)[C@@H](CO)O[C@H]2OCc3ccccc3)[C@H](O)[C@@H](O)[C@@H]1O |
| 2423 | COc1cc2CC[NH2+][C@H]3CC4(C=CC(=O)C=C4)c(c1OC)c23 |
| 2424 | CC[C@@H](C)[C@H]([NH+](C)C)C(=O)N[C@H]1[C@@H](Oc2ccc(\C=C/NC(=O)[C@@H](Cc3ccccc3)NC1=O)cc2)C(C)C |
| 2425 | CC[C@@H](C)[C@H](N(C)C)C(=O)N[C@H]1[C@@H](Oc2ccc(\C=C/NC(=O)[C@@H](Cc3ccccc3)NC1=O)cc2)C(C)C |
| 2426 | CC(=C)[C@H]1CCCC(=C1)C |
| 2427 | O[C@H]1[C@H](COC(=O)c2cc(O)c(O)c(O)c2)O[C@@H](OC(=O)c3cc(O)c(O)c(O)c3)[C@H](O)[C@H]1OC(=O)c4cc(O)c(O)c(O)c4 |
| 2428 | O[C@H]1[C@H](COC(=O)c2cc(O)c(O)c(O)c2)O[C@@H](OC(=O)c3cc(O)c([O-])c(O)c3)[C@H](O)[C@H]1OC(=O)c4cc(O)c(O)c(O)c4 |
| 2429 | O[C@H]1[C@H](COC(=O)c2cc(O)c(O)c(O)c2)O[C@@H](OC(=O)c3cc(O)c(O)c(O)c3)[C@H](O)[C@H]1OC(=O)c4cc(O)c([O-])c(O)c4 |
| 2430 | O[C@H]1[C@H](COC(=O)c2cc(O)c(O)c(O)c2)O[C@@H](OC(=O)c3cc(O)c([O-])c(O)c3)[C@H](O)[C@H]1OC(=O)c4cc(O)c([O-])c(O)c4 |
| 2431 | O[C@H]1[C@H](COC(=O)c2cc(O)c([O-])c(O)c2)O[C@@H](OC(=O)c3cc(O)c([O-])c(O)c3)[C@H](O)[C@H]1OC(=O)c4cc(O)c([O-])c(O)c4 |
| 2432 | C[C@H]1O[C@@H](O[C@H]2CO[C@@H](O[C@H]3CC[C@@]4(C)[C@@H](CC[C@]5(C)[C@@H]4CC[C@@H]6[C@H]7[C@](C)(C[C@H](O[C@@]78C[C@@]56CO8)C=C(C)C)O[C@@H]9O[C@H](C)[C@H](O)[C@@H](OC(=O)C)[C@H]9OC(=O)C)C3(C)C)[C@H](O)[C@@H]2O)[C@H](O)[C@H](O)[C@@H]1O |
| 2433 | CC[C@H](C)[C@H](NC(=O)[C@H]([C@@H](C)CC)[NH+](C)C)C(=O)N1CC[C@@H]2Oc3ccc(\C=C/NC(=O)[C@@H]4CCCN4C(=O)[C@@H]12)c(OC)c3 |
| 2434 | CC[C@H](C)[C@H](NC(=O)[C@H]([C@@H](C)CC)N(C)C)C(=O)N1CC[C@@H]2Oc3ccc(\C=C/NC(=O)[C@@H]4CCCN4C(=O)[C@@H]12)c(OC)c3 |
| 2435 | C[C@@H]1O[C@@H](O[C@H]2[C@H](O[C@H]3CC[C@@]4(C)[C@H](CC[C@@]5(C)[C@H]4CC[C@@H]6[C@@H]7[C@@](C)(O)C[C@H](O[C@@]78C[C@@]56CO8)C=C(C)C)C3(C)C)OC[C@H](O)[C@@H]2O[C@@H]9O[C@H](CO)[C@@H](O)[C@H](O)[C@H]9O[C@@H]%10OC[C@@H](O)[C@H](O)[C@H]%10O)[C@H](O)[C@H](O)[C@@H]1O |
| 2436 | CC(=C[C@@H]1C[C@](C)(O)[C@H]2[C@H]3CC[C@H]4[C@@]5(C)CC[C@H](O)C(C)(C)[C@H]5CC[C@]4(C)[C@@]36CO[C@@]2(C6)O1)C |
| 2437 | C[C@@H]1O[C@@H](O[C@H]2[C@H](O[C@H]3CC[C@@]4(C)[C@H](CC[C@@]5(C)[C@H]4CC[C@@H]6[C@@H]7[C@@](C)(O)C[C@H](O[C@@]78C[C@@]56CO8)C=C(C)C)C3(C)C)OC[C@H](O)[C@@H]2O[C@@H]9O[C@H](CO)[C@@H](O)[C@H](O)[C@H]9O)[C@H](O)[C@H](O)C1(O)O |
| 2438 | C[C@@H](O[C@@H]1O[C@H](CO)[C@@H](O)[C@H](O)[C@H]1O[C@@H]2O[C@@H](C)[C@H](O)[C@@H](O)[C@H]2O)\C=C\[C@@]3(O)C(=CC(=O)CC3(C)C)C |
| 2439 | C[C@H](O)\C=C\[C@@]1(O)C(=CC(=O)CC1(C)C)C |
| 2440 | Nc1ncnc2c1ncn2[C@@H]3O[C@@H]4COP(=O)([O-])O[C@H]4[C@H]3O |
| 2441 | NC1=NC(=O)c2ncn([C@@H]3O[C@@H]4COP(=O)([O-])O[C@H]4[C@H]3O)c2N1 |
| 2442 | O=C1CCC2=C1[NH2+]COC2 |
| 2443 | O=C1CCC2=C1NCOC2 |
| 2444 | COc1ccc2C[C@H]3CCN[C@@H]3C(=O)N[C@@H](CC(C)C)C(=O)N\C=C/c1c2 |
| 2445 | CC[C@H](C)[C@H](CC(=O)[C@@H]([C@@H](C)CC)[NH+](C)C)C(=O)N1CC[C@@H]2Oc3ccc(OC)c(\C=C/NC(=O)[C@@H](NC(=O)[C@@H]12)[C@@H](C)CC)c3 |
| 2446 | CC[C@H](C)[C@H](CC(=O)[C@@H]([C@@H](C)CC)N(C)C)C(=O)N1CC[C@@H]2Oc3ccc(OC)c(\C=C/NC(=O)[C@@H](NC(=O)[C@@H]12)[C@@H](C)CC)c3 |
| 2447 | C[C@H](CCC=C(C)C)[C@H]1CC[C@H]2[C@@H]3CC=C4C[C@@H](O)CC[C@]4(C)[C@H]3CC[C@]12C |
| 2448 | CC[C@@H](C)[C@H]([NH+](C)C)C(=O)N[C@H]1[C@@H](Oc2ccc(\C=C/NC(=O)[C@H](CC(C)C)NC1=O)cc2)C(C)C |
| 2449 | CC[C@@H](C)[C@H](N(C)C)C(=O)N[C@H]1[C@@H](Oc2ccc(\C=C/NC(=O)[C@H](CC(C)C)NC1=O)cc2)C(C)C |
| 2450 | O[C@H]1O[C@@H]([C@H](O)[C@H](O)[C@H]1O)C(=O)[O-] |
| 2451 | C[C@H]1O[C@@H](O[C@H]2[C@H](O[C@@H]3CC[C@@]4(C)[C@H](CC[C@@]5(C)[C@H]4CC[C@H]6[C@@H]7[C@@](C)(O)[C@@H](CC=C(C)C)O[C@]78C[C@]56CO8)C3(C)C)O[C@H](CO)[C@H](O)[C@H]2O[C@@H]9O[C@H](CO)[C@@H](O)[C@@H](O)[C@H]9O)[C@H](O)[C@H](O)[C@@H]1O |
| 2452 | CC(=CC[C@H]1O[C@@]23C[C@@]4(CO2)[C@@H](CC[C@H]5[C@@]6(C)CC[C@@H](O)C(C)(C)[C@H]6CC[C@]45C)[C@@H]3[C@@]1(C)O)C |
| 2453 | C[C@@H]1O[C@@H](O[C@H]2[C@@H](O[C@H]3CC[C@@]4(C)[C@@H](CC[C@]5(C)[C@@H]4CC[C@@H]6[C@H]7[C@@](C)(O)C[C@@H](O[C@]78C[C@]56CO8)C=C(C)C)C3(C)C)OC[C@H](O)[C@@H]2O[C@@H]9O[C@H](CO[C@H]%10O[C@H](CO)[C@@H](O)[C@H](O)[C@H]%10O)[C@@H](O)[C@H](O)[C@H]9O[C@@H]%11OC[C@@H](O)[C@H](O)[C@H]%11O)[C@H](O)[C@H](O)[C@H]1O |
| 2454 | CC(=C[C@H]1C[C@](C)(O)[C@@H]2[C@H]3CC[C@@H]4[C@@]5(C)CC[C@H](O)C(C)(C)[C@@H]5CC[C@@]4(C)[C@]36CO[C@]2(C6)O1)C |
| 2455 | COc1cc2OC(=CC(=O)c2c(O)c1[C@H]3O[C@H](CO)[C@@H](O)[C@@H](O)[C@@H]3O)c4ccc(O)cc4 |
| 2456 | COc1cc2OC(=CC(=O)c2c(O)c1[C@H]3O[C@H](CO)[C@@H](O)[C@@H](O)[C@@H]3O)c4ccc([O-])cc4 |
| 2457 | OC[C@H]1O[C@@H](O[C@H]2[C@H](O)[C@@H](CO)O[C@@H](OCc3ccccc3)[C@@H]2O[C@@H]4O[C@@H](CO)[C@H](O)[C@@H](O)[C@@H]4O)[C@H](O)[C@@H](O)[C@@H]1O |
| 2458 | C[C@@H]1O[C@@H](O[C@H]2[C@H](O[C@H]3CC[C@@]4(C)[C@H](CC[C@@]5(C)[C@H]4CC[C@@H]6[C@@H]7[C@@](C)(O)C[C@H](O[C@@]78C[C@@]56CO8)C=C(C)C)C3(C)C)OC[C@H](O)[C@@H]2O[C@@H]9O[C@H](CO)[C@@H](O)[C@H](O)[C@H]9O)[C@H](O)[C@H](O)[C@@H]1O |
| 2459 | C[C@H](O[C@H]1O[C@@H](CO)[C@H](O)[C@@H](O[C@H]2O[C@@H](CO)[C@H](O)[C@@H](O)[C@@H]2O)[C@@H]1O[C@H]3O[C@@H](CO)[C@H](O)[C@@H](O)[C@@H]3O)\C=C\[C@]4(O)C(=CC(=O)CC4(C)C)C |
| 2460 | C[C@H](O)\C=C\[C@]1(O)C(=CC(=O)CC1(C)C)C |
| 2461 | C[C@H](O[C@H]1O[C@@H](CO)[C@H](O)[C@@H](O)[C@@H]1O[C@H]2O[C@@H](C)[C@H](O)[C@@H](O)[C@@H]2O)\C=C\[C@]3(O)C(=CC(=O)CC3(C)C)C |
| 2462 | C[C@@H]1O[C@@H](OC(=O)\C=C\c2ccc(O)cc2)[C@H](O)[C@H](O)[C@H]1O |
| 2463 | CC1(C)CC[C@]2(CC[C@@]3(C)C(=CC[C@H]4[C@@]5(C)C[C@@H](O)[C@@H](O)C(C)(C)[C@H]5CC[C@@]34C)[C@H]2C1)C(=O)O |
| 2464 | CC1(C)CC[C@]2(CC[C@@]3(C)C(=CC[C@H]4[C@@]5(C)C[C@@H](O)[C@@H](O)C(C)(C)[C@H]5CC[C@@]34C)[C@H]2C1)C(=O)[O-] |
| 2465 | C[C@@H]1CC[C@@]2(CC[C@]3(C)C(=CC[C@H]4[C@@]5(C)C[C@H](O)[C@@H](O)C(C)(C)[C@@H]5CC[C@@]34C)[C@@H]2[C@H]1C)C(=O)O |
| 2466 | C[C@@H]1CC[C@@]2(CC[C@]3(C)C(=CC[C@H]4[C@@]5(C)C[C@H](O)[C@@H](O)C(C)(C)[C@@H]5CC[C@@]34C)[C@@H]2[C@H]1C)C(=O)[O-] |
| 2467 | CC(=C)[C@H]1CC[C@]2(CC[C@]3(C)[C@@]4(C)CC[C@@H]5C(C)(C)[C@@H](OC(=O)\C=C/c6ccc(O)cc6)[C@H](O)C[C@]5(C)[C@@H]4CC[C@@]3(C)[C@@H]12)C(=O)O |
| 2468 | CC(=C)[C@H]1CC[C@]2(CC[C@]3(C)[C@@]4(C)CC[C@@H]5C(C)(C)[C@@H](OC(=O)\C=C/c6ccc(O)cc6)[C@H](O)C[C@]5(C)[C@@H]4CC[C@@]3(C)[C@@H]12)C(=O)[O-] |
| 2469 | COC(=O)C1=CO[C@@H](O[C@@H]2O[C@H](CO[C@@H]3O[C@H](COC(=O)\C=C\c4ccc(O)cc4)[C@@H](O)[C@H](O)[C@H]3O)[C@@H](O)[C@H](O)[C@H]2O)[C@H]5[C@@H]1CC=C5CO |
| 2470 | CC[C@H](C)[C@@H]1NC(=O)[C@@H](NC(=O)[C@H](CC(C)C)[NH+](C)C)[C@@H](Oc2ccc(\C=C/NC1=O)cc2)C(C)C |
| 2471 | CC[C@H](C)[C@@H]1NC(=O)[C@@H](NC(=O)[C@H](CC(C)C)N(C)C)[C@@H](Oc2ccc(\C=C/NC1=O)cc2)C(C)C |
| 2472 | CC[C@]12C[C@H]3CN(CCc4c([nH]c5ccccc45)[C@@]([C@H]3O1)(C(=O)OC)c6cc7c(cc6OC)N(C)[C@H]8[C@](O)([C@H](OC(=O)C)[C@]9(CC)C=CCN%10CC[C@]78[C@H]9%10)C(=O)OC)C2 |
| 2473 | Oc1ccc2c(OC(=O)c3c2oc4cc(O)ccc34)c1 |
| 2474 | Oc1ccc2c3C(=O)Oc4cc([O-])ccc4c3oc2c1 |
| 2475 | CC[C@@H](C)[C@H]1NC(=O)[C@H]2[C@@H](CCN2C(=O)[C@@H](Cc3ccc(O)cc3)[NH+](C)C)Oc4ccc(OC)c(\C=C\NC1=O)c4 |
| 2476 | CC[C@@H](C)[C@@H]1NC(=O)[C@@H]2[C@H](CCN2C(=O)[C@@H](Cc3ccccc3)[NH+](C)C)Oc4ccc(O)c(\C=C/NC1=O)c4 |
| 2477 | CC[C@@H](C)[C@@H]1NC(=O)[C@@H]2[C@H](CCN2C(=O)[C@@H](Cc3ccccc3)N(C)C)Oc4ccc(O)c(\C=C/NC1=O)c4 |
| 2478 | CC(C)C[C@H]1NC(=O)[C@H](NC(=O)[C@H](C(C)C)[NH+](C)C)[C@@H](Oc2ccc(CCNC1=O)cc2)C(C)C |
| 2479 | CC(C)C[C@H]1NC(=O)[C@H](NC(=O)[C@H](C(C)C)N(C)C)[C@@H](Oc2ccc(CCNC1=O)cc2)C(C)C |
| 2480 | CC[C@@H](C)[C@@H]1NC(=O)[C@@H]2[C@H](CCN2C(=O)[C@@H](Cc3ccccc3)[NH+](C)C)Oc4ccc(OC)c(\C=C/NC1=O)c4 |
| 2481 | CC[C@@H](C)[C@@H]1NC(=O)[C@@H]2[C@H](CCN2C(=O)[C@@H](Cc3ccccc3)N(C)C)Oc4ccc(OC)c(\C=C/NC1=O)c4 |
| 2482 | COc1ccc2O[C@H]3CCN([C@@H]3C(=O)N[C@H](CC(C)C)C(=O)N\C=C/c1c2)C(=O)[C@H](CC(C)C)[NH+](C)C |
| 2483 | COc1ccc2O[C@H]3CCN([C@@H]3C(=O)N[C@H](CC(C)C)C(=O)N\C=C/c1c2)C(=O)[C@H](CC(C)C)N(C)C |
| 2484 | COc1ccc2O[C@H]3CCN([C@@H]3C(=O)N[C@H](CC(C)C)C(=O)N\C=C/c1c2)C(=O)[C@H](CC(C)C)CC(=O)[C@@H](CC(C)C)[NH+](C)C |
| 2485 | COc1ccc2O[C@H]3CCN([C@@H]3C(=O)N[C@H](CC(C)C)C(=O)N\C=C/c1c2)C(=O)[C@H](CC(C)C)CC(=O)[C@@H](CC(C)C)N(C)C |
| 2486 | C[C@@H]1O[C@H](O[C@@H]2[C@@H](O)[C@@H](O)[C@@H](CO)O[C@@H]2O[C@@H]3CC[C@]4(C)[C@H](CC[C@]5(C)[C@@H]4CC[C@H]6[C@@H]7[C@@]8(C)O[C@H](C[C@H]8O[C@@]79C[C@@]56CO9)C(C)(C)O)C3(C)C)[C@H](O)[C@H](O)[C@H]1O |
| 2487 | CC(C)(O)[C@H]1C[C@H]2O[C@]34C[C@]5(CO3)[C@@H](CC[C@@H]6[C@]7(C)CC[C@@H](O)C(C)(C)[C@H]7CC[C@@]56C)[C@@H]4[C@@]2(C)O1 |
| 2488 | C[C@@H]1O[C@@H](O[C@H]2[C@H](O[C@H]3CC[C@@]4(C)[C@H](CC[C@]5(C)[C@H]4CC[C@H]6[C@@H]7[C@@](C)(O)[C@H](CC=C(C)C)O[C@@]78C[C@@]56CO8)C3(C)C)O[C@H](CO)[C@@H](O)[C@@H]2O[C@@H]9O[C@H](CO)[C@@H](O)[C@H](O)[C@H]9O)[C@H](O)[C@H](O)[C@H]1O |
| 2489 | CC(=CC[C@@H]1O[C@]23C[C@]4(CO2)[C@@H](CC[C@H]5[C@@]6(C)CC[C@H](O)C(C)(C)[C@H]6CC[C@@]45C)[C@@H]3[C@@]1(C)O)C |
| 2490 | CC(=C[C@@H]1C[C@](C)(O)[C@@H]2[C@@H]3CC[C@@H]4[C@@]5(C)CC[C@H](O)C(C)(C)[C@@H]5CC[C@@]4(C)[C@@]36CO[C@@]2(C6)O1)C |
| 2491 | C[C@H]1O[C@H](O[C@H]2[C@H](O[C@@H]3CC[C@]4(C)[C@@H](CC[C@]5(C)[C@@H]4CC[C@@H]6[C@H]7[C@](C)(O)C[C@H](O[C@]78C[C@]56CO8)C=C(C)C)C3(C)C)OC[C@@H](O)[C@@H]2O[C@@H]9O[C@H](CO[C@@H]%10O[C@H](CO)[C@H](O)[C@H](O)[C@H]%10O)[C@H](O)[C@H](O)[C@H]9O[C@H]%11OC[C@H](O)[C@@H](O)[C@H]%11O)[C@H](O)[C@@H](O)[C@H]1O |
| 2492 | CC(=C[C@@H]1C[C@@](C)(O)[C@@H]2[C@H]3CC[C@@H]4[C@]5(C)CC[C@@H](O)C(C)(C)[C@@H]5CC[C@@]4(C)[C@]36CO[C@]2(C6)O1)C |
| 2493 | C[C@H]1O[C@H](O[C@H]2[C@H](O[C@@H]3CC[C@]4(C)[C@@H](CC[C@]5(C)[C@@H]4CC[C@@H]6[C@H]7[C@](C)(O)C[C@H](O[C@]78C[C@]56CO8)C=C(C)C)C3(C)C)OC[C@@H](O)[C@@H]2O[C@@H]9O[C@H](CO[C@@H]%10O[C@H](CO)[C@H](O)[C@H](O)[C@H]%10O)[C@@H](O)[C@H](O)[C@H]9O[C@@H]%11O[C@H](CO)[C@H](O)[C@@H](O)[C@@H]%11O)[C@H](O)[C@@H](O)[C@H]1O |
| 2494 | C[C@@H]1O[C@@H](O[C@H]2[C@H](O[C@H]3CC[C@@]4(C)[C@@H](CC[C@]5(C)[C@@H]4CC[C@@H]6[C@H]7[C@@](C)(O)C[C@@H](O[C@@]78C[C@@]56CO8)C=C(C)C)C3(C)C)OC[C@H](O)[C@@H]2O[C@@H]9O[C@H](CO)[C@@H](O)[C@H](O)[C@H]9O[C@@H]%10OC[C@@H](O)[C@H](O)[C@H]%10O)[C@H](O)[C@H](O)[C@H]1O |
| 2495 | CC(C)C[C@@H]1NC(=O)[C@H]2NCC[C@@H]2Oc3ccc(\C=C/NC1=O)cc3 |
| 2496 | CC(C)[C@@H](NC(=O)[C@@H](C)[NH+](C)C)C(=O)N1CC[C@@H]2Oc3ccc(\C=C/NC(=O)[C@@H](Cc4ccccc4)NC(=O)[C@H]12)cc3 |
| 2497 | CC(C)[C@@H](NC(=O)[C@@H](C)N(C)C)C(=O)N1CC[C@@H]2Oc3ccc(\C=C/NC(=O)[C@@H](Cc4ccccc4)NC(=O)[C@H]12)cc3 |
| 2498 | CC(=C)[C@@H]1CC[C@@]2(CC[C@]3(C)[C@H](CC[C@@H]4[C@@]5(C)[C@H](C=O)[C@@H](O)C(C)(C)[C@@H]5CC[C@@]34C)[C@@H]12)C(=O)O |
| 2499 | CC(=C)[C@@H]1CC[C@@]2(CC[C@]3(C)[C@H](CC[C@@H]4[C@@]5(C)[C@H](C=O)[C@@H](O)C(C)(C)[C@@H]5CC[C@@]34C)[C@@H]12)C(=O)[O-] |
| 2500 | CC[C@H](CC[C@@H](C)[C@H]1CC[C@H]2[C@@H]3C[C@@H](O)C4=C[C@@H](O)CC[C@]4(C)[C@H]3CC[C@]12C)C(C)C |
| 2501 | COc1cc(ccc1O)C2=CC(=O)c3c(O)c(OC)c(OC)cc3O2 |
| 2502 | COc1cc(ccc1[O-])C2=CC(=O)c3c(O)c(OC)c(OC)cc3O2 |
| 2503 | COc1cc2OC(=CC(=O)c2c(O)c1OC)c3ccc(O)c(O)c3 |
| 2504 | COc1cc2OC(=CC(=O)c2c(O)c1OC)c3ccc(O)cc3 |
| 2505 | COc1cc2OC(=CC(=O)c2c(O)c1OC)c3ccc([O-])cc3 |
| 2506 | C[C@H]1COC2=C1C(=O)C(=O)c3c4CCCC(C)(C)c4ccc23 |
| 2507 | C[C@H]1COC2=C1C(=O)[C@](O)(CC(=O)C)c3c2ccc4c(C)cccc34 |
| 2508 | C[C@H]1COC2=C1C(=O)[C@](O)(CC(=O)C)c3c4CCCC(C)(C)c4ccc23 |
| 2509 | C[C@H]1CO[C@@]2(C1)OC(=O)c3c2ccc4c(C)cccc34 |
| 2510 | O[C@H](Cc1ccc(O)c(O)c1)C(=O)[O-] |
| 2511 | Oc1ccc(C[C@@H](OC(=O)\C=C\c2ccc(O)c3O[C@H]([C@H](C(=O)O[C@H](Cc4ccc(O)c(O)c4)C(=O)[O-])c23)c5ccc(O)c(O)c5)C(=O)[O-])cc1O |
| 2512 | Oc1ccc(C[C@@H](OC(=O)\C=C\c2ccc(O)c3O[C@H]([C@H](C(=O)O[C@H](Cc4ccc(O)c(O)c4)C(=O)[O-])c23)c5ccc(O)c([O-])c5)C(=O)[O-])cc1O |
| 2513 | C[C@@H](CO)C1=C([O-])C(=O)c2c(ccc3c(C)cccc23)C1=O |
| 2514 | CC(C)C1=C([O-])C(=O)c2c(ccc3c(C)cccc23)C1=O |
| 2515 | CC1=C([O-])C(=O)c2c(ccc3c(C)cccc23)C1=O |
| 2516 | C[C@@H]1COC2=C(C(=O)c3ccc4c(C)cccc4c3C2=O)[C@@]1(C)CO |
| 2517 | CC(C)C1=Cc2ccc3c(C=CCC3(C)C)c2C(=O)C1=O |
| 2518 | Cc1coc2c3ccc4c(C=CCC4(C)C)c3C(=O)C(=O)c12 |
| 2519 | C[C@H]1COC2=C1C(=O)c3ccc4c(C)cccc4c3C2=O |
| 2520 | C[C@H]1COC2=C1C(=O)C(=O)c3c2ccc4c(C)cccc34 |
| 2521 | CC1=CCCc2c1ccc3c4occ(C)c4C(=O)C(=O)c23 |
| 2522 | C[C@@H]1CO[C@]2(C1)OC(=O)c3c2ccc4c(C)cccc34 |
| 2523 | CC(C)c1cc2CC[C@H]3C(C)(C)CCC[C@]3(C)c2cc1O |
| 2524 | CC(C)c1cc2C(=O)[C@H]3OC(=O)[C@@]4(CCCC(C)(C)[C@@H]34)c2c(O)c1O |
| 2525 | COc1ccc(\C=C\C(=O)[O-])cc1O |
| 2526 | CC(C)[C@]12[C@H](C(=CC1=O)C)C3=C(C2=O)[C@@]45CCCC(C)(C)[C@@H]4[C@H](OC5=O)[C@@H]3O |
| 2527 | CC(C)[C@@]12[C@@H](C(=CC1=O)C)C3=C(C2=O)[C@@]45CCCC(C)(C)[C@@H]4[C@H](OC5=O)[C@H]3O |
| 2528 | CC(C)[C@]12[C@H](C(=CC1=O)C)C3=C(C2=O)[C@@]45CCCC(C)(C)[C@@H]4[C@H](OC5=O)[C@H]3O |
| 2529 | Cc1coc2c3ccc4C(=C)[C@@H](O)CCc4c3C(=O)C(=O)c12 |
| 2530 | Cc1coc2c3ccc4c(CC[C@@H](O)C4(C)C)c3C(=O)C(=O)c12 |
| 2531 | Cc1coc2c3ccc4c(CC[C@H](O)C4(C)C)c3C(=O)C(=O)c12 |
| 2532 | C[C@H]1COC2=C1C(=O)c3ccc4c(CCCC4(C)C)c3C2=O |
| 2533 | CC(C)C1=Cc2ccc3c(C)cccc3c2C(=O)C1=O |
| 2534 | Cc1coc2C(=O)c3c(ccc4c(C)cccc34)C(=O)c12 |
| 2535 | Cc1coc2C(=O)c3c4CCCC(C)(C)c4ccc3C(=O)c12 |
| 2536 | C[C@@H]1COC2=C1C(=O)c3ccc4c(C(=O)CCC4(C)C)c3C2=O |
| 2537 | Oc1ccc(C[C@H]2OC(=O)CCc3ccc(O)c4O[C@@H]([C@@H](C(=O)O[C@@H](Cc5ccc(O)c(O)c5)C(=O)O[Mg]OC2=O)c34)c6ccc(O)c(O)c6)cc1O |
| 2538 | Oc1ccc(C[C@H]2OC(=O)CCc3ccc(O)c4O[C@@H]([C@@H](C(=O)O[C@@H](Cc5ccc(O)c(O)c5)C(=O)O[Mg]OC2=O)c34)c6ccc(O)c([O-])c6)cc1O |
| 2539 | Oc1ccc(C[C@@H](OC(=O)\C=C\c2ccc(O)c3O[C@@H]([C@@H](C(=O)O[C@H](Cc4ccc(O)c(O)c4)C(=O)[O-])c23)c5ccc(O)c(O)c5)C(=O)[O-])cc1O |
| 2540 | Oc1ccc(C[C@@H](OC(=O)\C=C\c2ccc(O)c3O[C@@H]([C@@H](C(=O)O[C@H](Cc4ccc(O)c(O)c4)C(=O)[O-])c23)c5ccc(O)c([O-])c5)C(=O)[O-])cc1O |
| 2541 | Oc1ccc(C[C@H](OC(=O)\C=C\c2ccc(O\C(=C/c3ccc(O)c(O)c3)\C(=O)[O-])c(O)c2)C(=O)[O-])cc1O |
| 2542 | CO[C@@H]1[C@H]2OC(=O)[C@@]3(CCCC(C)(C)[C@@H]23)c4c(O)c(O)c(cc14)C(C)C |
| 2543 | Cc1coc2c3ccc4C(=C)CCCc4c3C(=O)C(=O)c12 |
| 2544 | COC(=O)\C(=C\c1ccc(O)c(O)c1)\Oc2ccc(\C=C\C(=O)O[C@@H](Cc3ccc(O)c(O)c3)C(=O)[O-])cc2O |
| 2545 | COC(=O)[C@]1(C)CCCc2c3C(=O)C(=O)c4c(C)coc4c3ccc12 |
| 2546 | CC(C)C1=C([O-])C(=O)c2c(ccc3c2C(=O)CCC3(C)C)C1=O |
| 2547 | C[C@H]1COC2=C1C(=O)C(=C3C4=C(C(=O)C=C23)C(C)(C)CCC4)[O-] |
| 2548 | CC1=C(O)C(=O)C=C2C(=C1)[C@@H]3C[C@H]4C(C)(C)CCC[C@]24CO3 |
| 2549 | CC(C)C1=Cc2ccc3c(CCCC3(C)C)c2C(=O)C1=O |
| 2550 | COC(=O)[C@H]1[C@@H](Oc2c(O)ccc(\C=C\C(=O)O[C@H](Cc3ccc(O)c(O)c3)C(=O)[O-])c12)c4ccc(O)c(O)c4 |
| 2551 | COC(=O)[C@H]1[C@@H](Oc2c(O)ccc(\C=C\C(=O)O[C@H](Cc3ccc(O)c(O)c3)C(=O)[O-])c12)c4ccc(O)c([O-])c4 |
| 2552 | C[C@@H](CO)C1=C([O-])C(=O)c2c3CCCC(C)(C)c3ccc2C1=O |
| 2553 | CC1=C([O-])C(=O)c2c3CCCC(C)(C)c3ccc2C1=O |
| 2554 | Cc1coc2c3ccc4C(=O)CCCc4c3C(=O)C(=O)c12 |
| 2555 | CCCCCCCC\C=C/CCCCCCCC(=O)OC[C@@H](C)C1=C([O-])C(=O)c2c(ccc3c(C)cccc23)C1=O |
| 2556 | CCCCCCCC\C=C/CCCCCCCC(=O)OC[C@@H](C)C1=C([O-])C(=O)c2c3CCCC(C)(C)c3ccc2C1=O |
| 2557 | CC1(C)CCCc2c3C(=O)C(=O)c4c(CO)coc4c3ccc12 |
| 2558 | Cc1cccc2c3C(=O)C(=O)c4c(CO)coc4c3ccc12 |
| 2559 | Cc1coc2c3ccc4[C@H](CO)[C@@H](O)CCc4c3C(=O)C(=O)c12 |
| 2560 | COC(=O)[C@@H](Cc1ccc(O)c(O)c1)OC(=O)\C=C\c2ccc(O)c(O)c2 |
| 2561 | Oc1ccc(C[C@H](OC(=O)\C=C\c2ccc(OC3=Cc4cc(O)c([O-])cc4OC3=O)c(O)c2)C(=O)[O-])cc1O |
| 2562 | Oc1ccc(C[C@@H](OC(=O)\C=C\c2ccc(O)c(O)c2\C=C\c3ccc(O)c(O)c3)C(=O)[O-])cc1O |
| 2563 | Oc1ccc(C[C@H](OC(=O)\C=C\c2ccc(O)c3O[C@@H]([C@H](C(=O)O[C@@H](Cc4ccc(O)c(O)c4)C(=O)[O-])c23)c5ccc(O)c(O)c5)C(=O)[O-])cc1O |
| 2564 | Oc1ccc(C[C@H](OC(=O)\C=C\c2ccc(O)c3O[C@H]([C@H](C(=O)O[C@@H](Cc4ccc(O)c(O)c4)C(=O)[O-])c23)c5ccc(O)c([O-])c5)C(=O)[O-])cc1O |
| 2565 | Oc1ccc(C[C@H](OC(=O)\C=C\c2ccc(O)c3oc(cc23)c4ccc(O)c(O)c4)C(=O)[O-])cc1O |
| 2566 | Oc1ccc(C[C@H](OC(=O)\C=C\c2ccc(O)c(O)c2CC(=O)[O-])C(=O)[O-])cc1O |
| 2567 | Oc1ccc(C[C@H](OC(=O)\C=C\c2ccc(O)c(O)c2\C(=C/c3ccc(O)c(O)c3)\C(=O)O[C@@H](Cc4ccc(O)c(O)c4)C(=O)[O-])C(=O)[O-])cc1O |
| 2568 | Oc1ccc2C=C3C(=O)[C@H](Cc4ccc(O)c(Oc2c1O)c34)C(=O)[O-] |
| 2569 | CC(C)C1=C2OC(C)(C)c3ccc4c(C)ccc(C1=O)c4c23 |
| 2570 | CC(=O)c1cc2ccc3c(CCCC3(C)C)c2cc1O |
| 2571 | CC(C)c1cc2CC[C@@H]3C(C)(C)C[C@H](O)C[C@]3(C)c2cc1O |
| 2572 | Cc1coc2c3ccc4c(CCC[C@@]4(C)C=O)c3C(=O)C(=O)c12 |
| 2573 | Cc1coc2c3ccc4c(CCC[C@@]4(O)CO)c3C(=O)C(=O)c12 |
| 2574 | Cc1coc2c3ccc4c(CC[C@@H](O)[C@]4(C)O)c3C(=O)C(=O)c12 |
| 2575 | Cc1coc2c3ccc4c(CC[C@H](O)[C@]4(C)O)c3C(=O)C(=O)c12 |
| 2576 | Cc1coc2c1OC(=O)c3c2ccc4c(C)cccc34 |
| 2577 | CC1=CO[C@@H]2[C@H]1C(=O)C(=O)c3c2ccc4c(CO)cccc34 |
| 2578 | CC1=CO[C@@H]2[C@@H]1C(=O)C(=O)c3c4CCC[C@@](C)(O)c4ccc23 |
| 2579 | Cc1coc2c3ccc4c(C)cccc4c3C(=O)C(=O)c12 |
| 2580 | Cc1coc2c3ccc4c(CCCC4(C)C)c3C(=O)C(=O)c12 |
| 2581 | Cc1coc2c3ccc4c(CCC[C@]4(C)CO)c3C(=O)C(=O)c12 |
| 2582 | C[C@@H](CO)C1=C([O-])c2ccc3c(C)cccc3c2C(=O)C1=O |
| 2583 | C[C@@H]1COC2=C1C(=O)C(=O)c3c4CCC=C(C)c4ccc23 |
| 2584 | COc1ccc(cc1)C2=CC(=O)c3c(OC)c(OC)c(OC)cc3O2 |
| 2585 | C[C@@H]1CC[C@@]2(OC1)O[C@H]3C[C@H]4[C@@H]5CC[C@H]6C[C@@H](O)CC[C@]6(C)[C@H]5CC[C@]4(C)[C@H]3[C@@H]2C |
| 2586 | CC1=CC[C@@]2(CC1)C(=C)CCCC2(C)C |
| 2587 | Oc1cc(cc([O-])c1O)C(=O)Oc2cc(cc(O)c2O)C(=O)[O-] |
| 2588 | Oc1cc(cc(OC(=O)c2cc([O-])c(O)c([O-])c2)c1O)C(=O)[O-] |
| 2589 | CC(=C1CC\C(=C/CC\C(=C/C1)\C)\C)C |
| 2590 | CC(=CCC[C@@](C)(OC(=O)C)C=C)C |
| 2591 | C[C@@H]1COC2=C1C(=O)C(=O)c3c4CCC=C(C)c4ccc23 |
| 2592 | CC[C@H](\C=C\[C@@H](C)[C@H]1CC[C@H]2[C@@H]3CC=C4C[C@@H](O)CC[C@]4(C)[C@@H]3CC[C@]12C)C(C)C |
| 2593 | CC[C@@H](CC[C@@H](C)[C@H]1CC[C@H]2[C@@H]3CC=C4C[C@@H](O)CC[C@]4(C)[C@H]3CC[C@]12C)C(C)C |
| 2594 | O[C@@H]1C[C@](O)(C[C@@H](OC(=O)\C=C\c2ccc(O)c(O)c2)[C@@H]1O)C(=O)[O-] |
| 2595 | CC(=O)O[C@H]1CC[C@@]2(C)[C@@H](CC[C@]3(C)[C@@H]2CC=C4[C@@H]5CC(C)(C)CC[C@@]5(CC[C@@]34C)C(=O)O)C1(C)C |
| 2596 | CC(=O)O[C@H]1CC[C@@]2(C)[C@@H](CC[C@]3(C)[C@@H]2CC=C4[C@@H]5CC(C)(C)CC[C@@]5(CC[C@@]34C)C(=O)[O-])C1(C)C |
| 2597 | CC(C)c1cc2C(=O)C[C@H]3C(C)(C)CCC[C@@]3(C)c2cc1O |
| 2598 | CC(C)CCC[C@@H](C)CCC[C@@H](C)CCC[C@]1(C)CCc2c(C)c(O)c(C)c(C)c2O1 |
| 2599 | CC(C)[C@H]1CCC(=C2CCC(=C[C@H]12)C)C |
| 2600 | C[C@@H]1CC[C@@]2(CC[C@]3(C)C(=CC[C@@H]4[C@@]5(C)C[C@@H](O)[C@@H](O)C(C)(C)[C@@H]5CC[C@@]34C)[C@@H]2[C@]1(C)O)C(=O)O |
| 2601 | C[C@@H]1CC[C@@]2(CC[C@]3(C)C(=CC[C@@H]4[C@@]5(C)C[C@@H](O)[C@@H](O)C(C)(C)[C@@H]5CC[C@@]34C)[C@@H]2[C@]1(C)O)C(=O)[O-] |
| 2602 | C[C@@H]1CC[C@@]2(OC1)O[C@H]3C[C@@H]4[C@@H]5CC[C@H]6C[C@@H](O)CC[C@]6(C)[C@H]5CC[C@]4(C)[C@H]3[C@@H]2C |
| 2603 | COC(=O)[C@H]1[C@H](Oc2c(O)ccc(\C=C\C(=O)O[C@H](Cc3ccc(O)c(O)c3)C(=O)[O-])c12)c4ccc(O)c(O)c4 |
| 2604 | COC(=O)[C@H]1[C@H](Oc2c(O)ccc(\C=C\C(=O)O[C@H](Cc3ccc(O)c(O)c3)C(=O)[O-])c12)c4ccc([O-])c(O)c4 |
| 2605 | C[C@@H]1CC[C@H]2C(C)(C)[C@H]3C[C@@]12CC[C@]3(C)O |
| 2606 | CSCC[C@H]([NH3+])C(=O)[O-] |
| 2607 | Cc1cc(O)c2C(=O)c3cccc(O)c3C(=O)c2c1 |
| 2608 | Cc1cc(O)c2C(=O)c3cccc([O-])c3C(=O)c2c1 |
| 2609 | Cc1cc([O-])c2C(=O)c3cccc([O-])c3C(=O)c2c1 |
| 2610 | C[C@@H]1CC[C@]2(C)CC[C@]3(C)C(=CC[C@@H]4[C@@]5(C)CC[C@@H](O)C(C)(C)[C@@H]5CC[C@@]34C)[C@@H]2[C@H]1C |
| 2611 | COc1cc(\C=C\C(=O)[O-])cc(O)c1OC |
| 2612 | CC(C)c1cc2ccc3c(C(=O)CCC3(C)C)c2c(O)c1O |
| 2613 | C[C@@H]1COC2=C1C(=O)c3ccc4c(C(=O)CCC4(C)C)c3C2=O |
| 2614 | C[C@@H]1CC[C@@]2(CC[C@]3(C)C(=CC[C@@H]4[C@@]5(C)C[C@@H](O)[C@@H](O)C(C)(C)[C@@H]5CC[C@@]34C)[C@@H]2[C@H]1C)C(=O)O |
| 2615 | C[C@@H]1CC[C@@]2(CC[C@]3(C)C(=CC[C@@H]4[C@@]5(C)C[C@@H](O)[C@@H](O)C(C)(C)[C@@H]5CC[C@@]34C)[C@@H]2[C@H]1C)C(=O)[O-] |
| 2616 | OCC(=O)c1ccc(O)c(O)c1 |
| 2617 | OCC(=O)c1ccc([O-])c(O)c1 |
| 2618 | CC(=CCC\C(=C\[C@H](O)C=O)\C)C |
| 2619 | Cc1coc2c3ccc4c([C@H](O)CCC4(C)C)c3C(=O)C(=O)c12 |
| 2620 | CC(C)[C@]12CC[C@@](C)(O)[C@H]1C2 |
| 2621 | Oc1ccc(cc1O)c2oc3c(O)ccc(\C=C\C(=O)[O-])c3c2 |
| 2622 | Oc1cc(ccc1[O-])c2oc3c(O)ccc(\C=C\C(=O)[O-])c3c2 |
| 2623 | CC(C)C1=Cc2ccc3C(=C)CCCc3c2C(=O)C1=O |
| 2624 | COc1cc(ccc1O)c2oc3c(OC)cc(CCCO)cc3c2C=O |
| 2625 | COc1cc(ccc1[O-])c2oc3c(OC)cc(CCCO)cc3c2C=O |
| 2626 | COC(=O)C1=CO[C@H](O[C@@H]2O[C@H](CO)[C@@H](O)[C@H](O)[C@H]2O)[C@@H]3[C@H]1[C@H](C[C@]3(C)OC(=O)C)OC(=O)c4cc(OC)c(O)c(OC)c4 |
| 2627 | COC(=O)C1=CO[C@H](O[C@@H]2O[C@H](CO)[C@@H](O)[C@H](O)[C@H]2O)[C@@H]3[C@H]1[C@H](C[C@]3(C)OC(=O)C)OC(=O)c4cc(OC)c([O-])c(OC)c4 |
| 2628 | COC(=O)C1=CO[C@H](O)[C@@H]2[C@H]1[C@H](C[C@]2(C)OC(=O)C)OC(=O)c3cc(OC)c(O)c(OC)c3 |
| 2629 | COC(=O)C1=CO[C@H](O)[C@@H]2[C@H]1[C@H](C[C@]2(C)OC(=O)C)OC(=O)c3cc(OC)c([O-])c(OC)c3 |
| 2630 | CC(C)C1=C([O-])C(=O)C2=C(C(=O)C[C@H]3C(C)(C)CCC[C@]23C)C1=O |
| 2631 | CC(C)C1=CC2=C(C(=O)C1=O)[C@@]3(C)CCCC(C)(C)[C@@H]3C[C@@H]2O |
| 2632 | COC(=O)[C@H](Cc1ccc(O)c(O)c1)OC(=O)\C=C\c2ccc(O)c3O[C@H]([C@@H](C(=O)O[C@@H](Cc4ccc(O)c(O)c4)C(=O)[O-])c23)c5ccc(O)c(O)c5 |
| 2633 | COC(=O)[C@H](Cc1ccc(O)c(O)c1)OC(=O)\C=C\c2ccc(O)c3O[C@H]([C@@H](C(=O)O[C@@H](Cc4ccc(O)c(O)c4)C(=O)[O-])c23)c5ccc(O)c([O-])c5 |
| 2634 | CC1(C)C[C@@H]2[C@H]1CC[C@@]3(C)O[C@H]3CCC2=C |
| 2635 | Cc1coc2c3ccc4c(C=O)cccc4c3C(=O)C(=O)c12 |
| 2636 | C\C\1=C/CCc2c(ccc3c4occ(C)c4C(=O)C(=O)c23)C(=C)CCCc5c1ccc6c7occ(C)c7C(=O)C(=O)c56 |
| 2637 | CC(C)C1=CC2=C(C(=O)C(=C1O)C(=O)C)[C@@]34CCCC(C)(C)[C@@H]3C(=O)[C@@H]2OC4=O |
| 2638 | CC(C)C1=CC2=C(C(=O)C(=C1[O-])C(=O)C)[C@@]34CCCC(C)(C)[C@@H]3C(=O)[C@@H]2OC4=O |
| 2639 | CC(C)C1=C[C@]23CC[C@H]4C(=CCCC4(C)C)[C@@H]2OC(=O)[C@]3(O)C1=O |
| 2640 | CO[C@@H]1C[C@@H]2C(=CCCC2(C)C)Cc3c(O)c(O)c(cc13)C(C)C |
| 2641 | CC(C)c1cc2CC[C@H]3C(=Cc2c(O)c1O)C=CCC3(C)C |
| 2642 | Cc1coc2c3ccc4c(CCC[C@@]4(C)O)c3C(=O)C(=O)c12 |
| 2643 | Cc1coc2c3ccc4c(CC[C@@H](O)[C@@]4(C)O)c3C(=O)C(=O)c12 |
| 2644 | Cc1coc2c3ccc4[C@H](CO)[C@H](O)CCc4c3C(=O)C(=O)c12 |
| 2645 | O[C@@H](Cc1ccc(O)c(O)c1)C(=O)[O-] |
| 2646 | CC(C)[C@@H]1[C@H]2OCC(C)(C)c3ccc4c(C)ccc(C1=O)c4c23 |
| 2647 | Oc1ccc(C[C@@H](OC(=O)\C=C\c2ccc(O)c3O[C@@H]([C@H](C(=O)O[C@H](Cc4ccc(O)c(O)c4)C(=O)[O-])c23)c5ccc(O)c(O)c5)C(=O)[O-])cc1O |
| 2648 | Oc1ccc(C[C@@H](OC(=O)\C=C\c2ccc(O)c3O[C@@H]([C@H](C(=O)O[C@H](Cc4ccc(O)c(O)c4)C(=O)[O-])c23)c5ccc(O)c([O-])c5)C(=O)[O-])cc1O |
| 2649 | OC1=CC(=C[C@H]2C=Cc3cc(O)c([O-])cc3O[C@H]12)\C=C\C(=O)O[C@H](Cc4ccc(O)c(O)c4)C(=O)[O-] |
| 2650 | CC(C)C1=Cc2ccc(C)c(CCC=C(C)C)c2C(=O)C1=O |
| 2651 | C[C@@](O)(CC[C@@H]1[C@](C)(O)CC[C@H]2C(C)(C)CCC[C@]12C)C=C |
| 2652 | Cc1coc2c3ccc4c(CCC[C@]4(C)C=O)c3C(=O)C(=O)c12 |
| 2653 | Cc1coc2c3ccc4c(CO)cccc4c3C(=O)C(=O)c12 |
| 2654 | CCCCCCC\C=C/CCCCCCCOC(=O)C |
| 2655 | CC(C)C1=Cc2ccc(C)c(CCCC(=C)C)c2C(=O)C1=O |
| 2656 | CC(C)c1cc2[C@@H]3C[C@@H]4C(C)(C)CCC[C@]4(C(=O)O3)c2c(O)c1O |
| 2657 | C[C@H](CO)C1=C([O-])C(=O)c2c(ccc3c(C)cccc23)C1=O |
| 2658 | C[C@H]1COC2=C(C(=O)c3ccc4c(C)cccc4c3C2=O)[C@]1(C)CO |
| 2659 | C[C@H]1CO[C@]2(C1)OCC(=O)c3c2ccc4c(C)cccc34 |
| 2660 | C[C@@H]1CO[C@@]2(C1)OC(=O)c3c4CCCC(C)(C)c4ccc23 |
| 2661 | CC[C@@H](CC[C@@H](C)[C@H]1CC[C@H]2[C@@H]3CC=C4C[C@H](CC[C@]4(C)[C@H]3CC[C@]12C)O[C@@H]5O[C@H](CO)[C@@H](O)[C@H](O)[C@H]5O)C(C)C |
| 2662 | C[C@@H]1[C@H]2C3=CC[C@@H]4[C@@]5(C)CC[C@@H](O)C(C)(C)[C@H]5CC[C@@]4(C)[C@]3(C)CC[C@@]2(CO)CCC1=C |
| 2663 | CC(C)C1=C([O-])C(=O)c2c3CCCC(C)(C)c3ccc2C1=O |
| 2664 | C[C@@H]1COC2=C1C(=O)c3ccc4c(C)cccc4c3C2=O |
| 2665 | C[C@H]1COC2=C1OC(=O)c3c2ccc4c(C)cccc34 |
| 2666 | C[C@@H]1COC2=C1C(=O)C(=O)c3c2ccc4c(C)cccc34 |
| 2667 | CC(=C)C1(CCCC[C@@]1(C)C=C)C(=C)C |
| 2668 | COC(=O)[C@H](Cc1ccc(O)c(O)c1)OC(=O)\C=C\c2ccc(O)c3O[C@H]([C@@H](C(=O)O[C@@H](Cc4ccc(O)c(O)c4)C(=O)OC)c23)c5ccc(O)c(O)c5 |
| 2669 | COC(=O)[C@H](Cc1ccc(O)c(O)c1)OC(=O)\C=C\c2ccc(O)c3O[C@H]([C@@H](C(=O)O[C@@H](Cc4ccc(O)c(O)c4)C(=O)OC)c23)c5ccc(O)c([O-])c5 |
| 2670 | COC(=O)[C@@H](Cc1ccc(O)c(O)c1)OC(=O)\C=C\c2ccc(O)c3O[C@@H]([C@H](C(=O)OC)c23)c4ccc(O)c(O)c4 |
| 2671 | COC(=O)[C@@H](Cc1ccc(O)c(O)c1)OC(=O)\C=C\c2ccc(O)c3O[C@@H]([C@H](C(=O)OC)c23)c4ccc([O-])c(O)c4 |
| 2672 | C[C@H]1CO[C@]2(C1)OCC(=O)[C@H]3[C@H]2C=Cc4c(C)cccc34 |
| 2673 | CCOC(=O)[C@H]1[C@H](Oc2cccc(\C=C\C(=O)O[C@H](Cc3ccccc3)C(=O)[O-])c12)c4ccc(O)cc4 |
| 2674 | CCOC(=O)[C@H]1[C@H](Oc2cccc(\C=C\C(=O)O[C@H](Cc3ccccc3)C(=O)[O-])c12)c4ccc([O-])cc4 |
| 2675 | C[C@@H]1COC2=C1C(=O)c3ccc4c(CCCC4(C)C)c3C2=O |
| 2676 | Oc1ccc(C[C@H](OC(=O)\C=C/c2ccc(O)c3Oc4cc(O)c(O)cc4C=Cc23)C(=O)[O-])cc1O |
| 2677 | Cc1coc2C(=O)c3c4CCC[C@](C)(CO)c4ccc3C(=O)c12 |
| 2678 | Oc1ccc(C[C@@H](OC(=O)\C=C\c2ccc(O)c3O[C@@H]([C@@H](C(=O)[O-])c23)c4ccc(O)c(O)c4)C(=O)[O-])cc1O |
| 2679 | Oc1ccc(C[C@@H](OC(=O)\C=C\c2ccc(O)c3O[C@@H]([C@@H](C(=O)[O-])c23)c4ccc([O-])c(O)c4)C(=O)[O-])cc1O |
| 2680 | C[C@](O)(CC[C@@]1(C)C(=C)CC[C@H]2C(C)(C)CCC[C@]12C)C=C |
| 2681 | COC(=O)[C@@H](Cc1ccc(O)c(O)c1)OC(=O)\C=C/c2ccc(O)c(O)c2 |
| 2682 | COC(=O)[C@@]1(C)CCCc2c3C(=O)C(=O)c4c(C)coc4c3ccc12 |
| 2683 | CC(C)C1=Cc2ccc(C)c3CCCC(C)(C)[C@@](O)(C1=O)c23 |
| 2684 | CC1(C)CCC(=O)[C@H]2[C@H]3C(=O)C(=CC(=O)C3=CC=C12)[O-] |
| 2685 | C[C@H](CO)C1=C([O-])c2ccc3c(CCCC3(C)C)c2C(=O)C1=O |
| 2686 | Cc1cc2[C@@H]3C[C@H]4C(C)(C)CCC[C@]4(CO3)c2cc1O |
| 2687 | CC1=C[C@]2(OC1=O)C=C(C(=O)[O-])[C@@]34CCCC(C)(C)[C@@H]3C[C@@H]2OC4 |
| 2688 | Oc1ccc(C[C@@H](OC(=O)\C=C/c2ccc(O)c(O)c2CC(=O)[O-])C(=O)[O-])cc1O |
| 2689 | Oc1ccc(cc1O)[C@@H]2Cc3c(\C=C\C(=O)[O-])ccc(O)c3O2 |
| 2690 | Oc1cc(ccc1[O-])[C@@H]2Cc3c(\C=C\C(=O)[O-])ccc(O)c3O2 |
| 2691 | Oc1ccc(cc1O)[C@H]2Oc3c(O)ccc(\C=C\C(=O)[O-])c3[C@@H]2C(=O)[O-] |
| 2692 | Oc1ccc(cc1[O-])[C@H]2Oc3c(O)ccc(\C=C\C(=O)[O-])c3[C@@H]2C(=O)[O-] |
| 2693 | Oc1ccc(C[C@@H](OC(=O)\C=C/c2ccc(O)c(O)c2)C(=O)[O-])cc1O |
| 2694 | C[C@]12C[C@@H](OC(=O)C1=CC[C@@]34COC(=O)[C@@H]3C=CC[C@]24O)c5cocc5 |
| 2695 | O[C@H](Cc1ccc(O)c(O)c1)C(=O)O[C@H](Cc2ccc(O)c(O)c2)C(=O)[O-] |
| 2696 | Oc1ccc(C[C@H](OC(=O)\C=C\c2ccc(O)c3oc(cc23)c4ccc(O)c(O)c4)C(=O)[O-])cc1O |
| 2697 | Oc1ccc(C[C@H](OC(=O)\C=C\c2ccc(O)c3oc(cc23)c4ccc([O-])c(O)c4)C(=O)[O-])cc1O |
| 2698 | Oc1ccc(C[C@@H](OC(=O)\C=C\c2ccc(O)c(O)c2CC(=O)[O-])C(=O)[O-])cc1O |
| 2699 | Oc1ccc2C[C@@H](C(=O)[O-])C(=O)C3=Cc4ccc([O-])c(O)c4Oc1c23 |
| 2700 | Oc1ccc(C[C@@H](OC(=O)\C=C\c2ccc3O[C@@H]([C@H](Oc3c2)C(=O)[O-])c4ccc(O)c(O)c4)C(=O)[O-])cc1O |
| 2701 | CC(=O)C1=C[C@@H]2C=CC3=C(CCCC3(C)C)[C@H]2C=C1O |
| 2702 | CC(C)c1cc2CC[C@@H]3C(C)(C)C[C@@H](O)C[C@@]3(C)c2cc1O |
| 2703 | CC1=Cc2ccc3c(CCCC3(C)C)c2C=C([O-])C1=O |
| 2704 | CC(C)C1=C([O-])C(=O)c2c(CCCC(=C)C)c(C)ccc2C1=O |
| 2705 | COC(=O)C1=CO[C@@H](O[C@@H]2O[C@H](CO)[C@@H](O)[C@H](O)[C@H]2O)[C@H]3[C@@H]1[C@H](O)C[C@]3(C)O |
| 2706 | COC(=O)C1=CO[C@@H](O)[C@H]2[C@@H]1[C@H](O)C[C@]2(C)O |
| 2707 | CC(C)c1cc2C(=O)C[C@H]3C(C)(C)CCC[C@]3(C)c2cc1O |
| 2708 | C[C@H](CO)C1=C([O-])c2ccc3c(C)cccc3c2C(=O)C1=O |
| 2709 | C[C@@H]1CC[C@]2(CO)CC[C@]3(C)C(=CC[C@@H]4[C@@]5(C)CC[C@H](O)C(C)(C)[C@H]5CC[C@@]34C)[C@@H]2[C@H]1C |
| 2710 | CC(C)[C@H]1CC[C@](C)(O)[C@H]2CCC(=C)C[C@@H]12 |
| 2711 | Oc1cc(cc(O)c1O)C(=O)Oc2cc(cc(O)c2O)C(=O)OC[C@H]3O[C@@H](OC(=O)c4cc(O)c(O)c(OC(=O)c5cc(O)c(O)c(O)c5)c4)[C@H](OC(=O)c6cc(O)c(O)c(OC(=O)c7cc(O)c(O)c(O)c7)c6)[C@@H](OC(=O)c8cc(O)c(O)c(OC(=O)c9cc(O)c(O)c(O)c9)c8)[C@@H]3OC(=O)c%10cc(O)c(O)c(OC(=O)c%11cc(O)c(O)c(O)c%11)c%10 |
| 2712 | Oc1cc(cc(O)c1O)C(=O)Oc2cc(cc(O)c2O)C(=O)OC[C@H]3O[C@@H](OC(=O)c4cc(O)c([O-])c(OC(=O)c5cc(O)c(O)c(O)c5)c4)[C@H](OC(=O)c6cc(O)c(O)c(OC(=O)c7cc(O)c(O)c(O)c7)c6)[C@@H](OC(=O)c8cc(O)c(O)c(OC(=O)c9cc(O)c(O)c(O)c9)c8)[C@@H]3OC(=O)c%10cc(O)c(O)c(OC(=O)c%11cc(O)c(O)c(O)c%11)c%10 |
| 2713 | Oc1cc(cc(O)c1O)C(=O)Oc2cc(cc(O)c2O)C(=O)OC[C@H]3O[C@@H](OC(=O)c4cc(O)c(O)c(OC(=O)c5cc(O)c(O)c(O)c5)c4)[C@H](OC(=O)c6cc(O)c([O-])c(OC(=O)c7cc(O)c(O)c(O)c7)c6)[C@@H](OC(=O)c8cc(O)c(O)c(OC(=O)c9cc(O)c(O)c(O)c9)c8)[C@@H]3OC(=O)c%10cc(O)c(O)c(OC(=O)c%11cc(O)c(O)c(O)c%11)c%10 |
| 2714 | Oc1cc(cc(O)c1O)C(=O)Oc2cc(cc(O)c2O)C(=O)OC[C@H]3O[C@@H](OC(=O)c4cc(O)c([O-])c(OC(=O)c5cc(O)c(O)c(O)c5)c4)[C@H](OC(=O)c6cc(O)c([O-])c(OC(=O)c7cc(O)c(O)c(O)c7)c6)[C@@H](OC(=O)c8cc(O)c(O)c(OC(=O)c9cc(O)c(O)c(O)c9)c8)[C@@H]3OC(=O)c%10cc(O)c(O)c(OC(=O)c%11cc(O)c(O)c(O)c%11)c%10 |
| 2715 | Oc1cc(cc(O)c1O)C(=O)Oc2cc(cc(O)c2O)C(=O)OC[C@H]3O[C@@H](OC(=O)c4cc(O)c(O)c(OC(=O)c5cc(O)c(O)c(O)c5)c4)[C@H](OC(=O)c6cc(O)c(O)c(OC(=O)c7cc(O)c(O)c(O)c7)c6)[C@@H](OC(=O)c8cc(O)c(O)c(OC(=O)c9cc(O)c(O)c(O)c9)c8)[C@@H]3OC(=O)c%10cc(O)c([O-])c(OC(=O)c%11cc(O)c(O)c(O)c%11)c%10 |
| 2716 | Oc1cc(cc(O)c1O)C(=O)Oc2cc(cc(O)c2O)C(=O)OC[C@H]3O[C@@H](OC(=O)c4cc(O)c([O-])c(OC(=O)c5cc(O)c(O)c(O)c5)c4)[C@H](OC(=O)c6cc(O)c(O)c(OC(=O)c7cc(O)c(O)c(O)c7)c6)[C@@H](OC(=O)c8cc(O)c(O)c(OC(=O)c9cc(O)c(O)c(O)c9)c8)[C@@H]3OC(=O)c%10cc(O)c([O-])c(OC(=O)c%11cc(O)c(O)c(O)c%11)c%10 |
| 2717 | Oc1cc(cc(O)c1O)C(=O)Oc2cc(cc(O)c2O)C(=O)OC[C@H]3O[C@@H](OC(=O)c4cc(O)c(O)c(OC(=O)c5cc(O)c(O)c(O)c5)c4)[C@H](OC(=O)c6cc(O)c([O-])c(OC(=O)c7cc(O)c(O)c(O)c7)c6)[C@@H](OC(=O)c8cc(O)c(O)c(OC(=O)c9cc(O)c(O)c(O)c9)c8)[C@@H]3OC(=O)c%10cc(O)c([O-])c(OC(=O)c%11cc(O)c(O)c(O)c%11)c%10 |
| 2718 | Oc1cc(cc(O)c1O)C(=O)Oc2cc(cc(O)c2O)C(=O)OC[C@H]3O[C@@H](OC(=O)c4cc(O)c([O-])c(OC(=O)c5cc(O)c(O)c(O)c5)c4)[C@H](OC(=O)c6cc(O)c([O-])c(OC(=O)c7cc(O)c(O)c(O)c7)c6)[C@@H](OC(=O)c8cc(O)c(O)c(OC(=O)c9cc(O)c(O)c(O)c9)c8)[C@@H]3OC(=O)c%10cc(O)c([O-])c(OC(=O)c%11cc(O)c(O)c(O)c%11)c%10 |
| 2719 | Oc1cc(cc(O)c1O)C(=O)Oc2cc(cc(O)c2O)C(=O)OC[C@H]3O[C@@H](OC(=O)c4cc(O)c(O)c(OC(=O)c5cc(O)c(O)c(O)c5)c4)[C@H](OC(=O)c6cc(O)c(O)c(OC(=O)c7cc(O)c(O)c(O)c7)c6)[C@@H](OC(=O)c8cc(O)c([O-])c(OC(=O)c9cc(O)c(O)c(O)c9)c8)[C@@H]3OC(=O)c%10cc(O)c(O)c(OC(=O)c%11cc(O)c(O)c(O)c%11)c%10 |
| 2720 | Oc1cc(cc(O)c1O)C(=O)Oc2cc(cc(O)c2O)C(=O)OC[C@H]3O[C@@H](OC(=O)c4cc(O)c([O-])c(OC(=O)c5cc(O)c(O)c(O)c5)c4)[C@H](OC(=O)c6cc(O)c(O)c(OC(=O)c7cc(O)c(O)c(O)c7)c6)[C@@H](OC(=O)c8cc(O)c([O-])c(OC(=O)c9cc(O)c(O)c(O)c9)c8)[C@@H]3OC(=O)c%10cc(O)c(O)c(OC(=O)c%11cc(O)c(O)c(O)c%11)c%10 |
| 2721 | Oc1cc(cc(O)c1O)C(=O)Oc2cc(cc(O)c2O)C(=O)OC[C@H]3O[C@@H](OC(=O)c4cc(O)c(O)c(OC(=O)c5cc(O)c(O)c(O)c5)c4)[C@H](OC(=O)c6cc(O)c([O-])c(OC(=O)c7cc(O)c(O)c(O)c7)c6)[C@@H](OC(=O)c8cc(O)c([O-])c(OC(=O)c9cc(O)c(O)c(O)c9)c8)[C@@H]3OC(=O)c%10cc(O)c(O)c(OC(=O)c%11cc(O)c(O)c(O)c%11)c%10 |
| 2722 | Oc1cc(cc(O)c1O)C(=O)Oc2cc(cc(O)c2O)C(=O)OC[C@H]3O[C@@H](OC(=O)c4cc(O)c([O-])c(OC(=O)c5cc(O)c(O)c(O)c5)c4)[C@H](OC(=O)c6cc(O)c([O-])c(OC(=O)c7cc(O)c(O)c(O)c7)c6)[C@@H](OC(=O)c8cc(O)c([O-])c(OC(=O)c9cc(O)c(O)c(O)c9)c8)[C@@H]3OC(=O)c%10cc(O)c(O)c(OC(=O)c%11cc(O)c(O)c(O)c%11)c%10 |
| 2723 | Oc1cc(cc(O)c1O)C(=O)Oc2cc(cc(O)c2O)C(=O)OC[C@H]3O[C@@H](OC(=O)c4cc(O)c(O)c(OC(=O)c5cc(O)c(O)c(O)c5)c4)[C@H](OC(=O)c6cc(O)c(O)c(OC(=O)c7cc(O)c(O)c(O)c7)c6)[C@@H](OC(=O)c8cc(O)c([O-])c(OC(=O)c9cc(O)c(O)c(O)c9)c8)[C@@H]3OC(=O)c%10cc(O)c([O-])c(OC(=O)c%11cc(O)c(O)c(O)c%11)c%10 |
| 2724 | Oc1cc(cc(O)c1O)C(=O)Oc2cc(cc(O)c2O)C(=O)OC[C@H]3O[C@@H](OC(=O)c4cc(O)c([O-])c(OC(=O)c5cc(O)c(O)c(O)c5)c4)[C@H](OC(=O)c6cc(O)c(O)c(OC(=O)c7cc(O)c(O)c(O)c7)c6)[C@@H](OC(=O)c8cc(O)c([O-])c(OC(=O)c9cc(O)c(O)c(O)c9)c8)[C@@H]3OC(=O)c%10cc(O)c([O-])c(OC(=O)c%11cc(O)c(O)c(O)c%11)c%10 |
| 2725 | Oc1cc(cc(O)c1O)C(=O)Oc2cc(cc(O)c2O)C(=O)OC[C@H]3O[C@@H](OC(=O)c4cc(O)c(O)c(OC(=O)c5cc(O)c(O)c(O)c5)c4)[C@H](OC(=O)c6cc(O)c([O-])c(OC(=O)c7cc(O)c(O)c(O)c7)c6)[C@@H](OC(=O)c8cc(O)c([O-])c(OC(=O)c9cc(O)c(O)c(O)c9)c8)[C@@H]3OC(=O)c%10cc(O)c([O-])c(OC(=O)c%11cc(O)c(O)c(O)c%11)c%10 |
| 2726 | Oc1cc(cc(O)c1O)C(=O)Oc2cc(cc(O)c2O)C(=O)OC[C@H]3O[C@@H](OC(=O)c4cc(O)c([O-])c(OC(=O)c5cc(O)c(O)c(O)c5)c4)[C@H](OC(=O)c6cc(O)c([O-])c(OC(=O)c7cc(O)c(O)c(O)c7)c6)[C@@H](OC(=O)c8cc(O)c([O-])c(OC(=O)c9cc(O)c(O)c(O)c9)c8)[C@@H]3OC(=O)c%10cc(O)c([O-])c(OC(=O)c%11cc(O)c(O)c(O)c%11)c%10 |
| 2727 | Oc1cc(cc(O)c1O)C(=O)Oc2cc(cc(O)c2O)C(=O)OC[C@H]3O[C@@H](OC(=O)c4cc(O)c([O-])c(OC(=O)c5cc(O)c(O)c(O)c5)c4)[C@H](OC(=O)c6cc(O)c([O-])c(OC(=O)c7cc(O)c(O)c(O)c7)c6)[C@@H](OC(=O)c8cc(O)c([O-])c(OC(=O)c9cc(O)c([O-])c(O)c9)c8)[C@@H]3OC(=O)c%10cc(O)c([O-])c(OC(=O)c%11cc(O)c(O)c(O)c%11)c%10 |
| 2728 | Oc1cc(cc(O)c1O)C(=O)Oc2cc(cc(O)c2O)C(=O)OC[C@H]3O[C@@H](OC(=O)c4cc(O)c([O-])c(OC(=O)c5cc(O)c(O)c(O)c5)c4)[C@H](OC(=O)c6cc(O)c([O-])c(OC(=O)c7cc(O)c(O)c(O)c7)c6)[C@@H](OC(=O)c8cc(O)c([O-])c(OC(=O)c9cc(O)c(O)c(O)c9)c8)[C@@H]3OC(=O)c%10cc(O)c([O-])c(OC(=O)c%11cc(O)c([O-])c(O)c%11)c%10 |
| 2729 | Oc1cc(cc(O)c1O)C(=O)Oc2cc(cc(O)c2O)C(=O)OC[C@H]3O[C@@H](OC(=O)c4cc(O)c([O-])c(OC(=O)c5cc(O)c(O)c(O)c5)c4)[C@H](OC(=O)c6cc(O)c([O-])c(OC(=O)c7cc(O)c(O)c(O)c7)c6)[C@@H](OC(=O)c8cc(O)c([O-])c(OC(=O)c9cc(O)c([O-])c(O)c9)c8)[C@@H]3OC(=O)c%10cc(O)c([O-])c(OC(=O)c%11cc(O)c([O-])c(O)c%11)c%10 |
| 2730 | Oc1cc(cc(O)c1O)C(=O)Oc2cc(cc(O)c2O)C(=O)OC[C@H]3O[C@@H](OC(=O)c4cc(O)c([O-])c(OC(=O)c5cc(O)c(O)c(O)c5)c4)[C@H](OC(=O)c6cc(O)c([O-])c(OC(=O)c7cc(O)c([O-])c(O)c7)c6)[C@@H](OC(=O)c8cc(O)c([O-])c(OC(=O)c9cc(O)c(O)c(O)c9)c8)[C@@H]3OC(=O)c%10cc(O)c([O-])c(OC(=O)c%11cc(O)c(O)c(O)c%11)c%10 |
| 2731 | Oc1cc(cc(O)c1O)C(=O)Oc2cc(cc(O)c2O)C(=O)OC[C@H]3O[C@@H](OC(=O)c4cc(O)c([O-])c(OC(=O)c5cc(O)c(O)c(O)c5)c4)[C@H](OC(=O)c6cc(O)c([O-])c(OC(=O)c7cc(O)c([O-])c(O)c7)c6)[C@@H](OC(=O)c8cc(O)c([O-])c(OC(=O)c9cc(O)c([O-])c(O)c9)c8)[C@@H]3OC(=O)c%10cc(O)c([O-])c(OC(=O)c%11cc(O)c(O)c(O)c%11)c%10 |
| 2732 | Oc1cc(cc(O)c1O)C(=O)Oc2cc(cc(O)c2O)C(=O)OC[C@H]3O[C@@H](OC(=O)c4cc(O)c([O-])c(OC(=O)c5cc(O)c(O)c(O)c5)c4)[C@H](OC(=O)c6cc(O)c([O-])c(OC(=O)c7cc(O)c([O-])c(O)c7)c6)[C@@H](OC(=O)c8cc(O)c([O-])c(OC(=O)c9cc(O)c(O)c(O)c9)c8)[C@@H]3OC(=O)c%10cc(O)c([O-])c(OC(=O)c%11cc(O)c([O-])c(O)c%11)c%10 |
| 2733 | Oc1cc(cc(O)c1O)C(=O)Oc2cc(cc(O)c2O)C(=O)OC[C@H]3O[C@@H](OC(=O)c4cc(O)c([O-])c(OC(=O)c5cc(O)c(O)c(O)c5)c4)[C@H](OC(=O)c6cc(O)c([O-])c(OC(=O)c7cc(O)c([O-])c(O)c7)c6)[C@@H](OC(=O)c8cc(O)c([O-])c(OC(=O)c9cc(O)c([O-])c(O)c9)c8)[C@@H]3OC(=O)c%10cc(O)c([O-])c(OC(=O)c%11cc(O)c([O-])c(O)c%11)c%10 |
| 2734 | Oc1cc(cc(O)c1O)C(=O)Oc2cc(cc(O)c2O)C(=O)OC[C@H]3O[C@@H](OC(=O)c4cc(O)c([O-])c(OC(=O)c5cc(O)c([O-])c(O)c5)c4)[C@H](OC(=O)c6cc(O)c([O-])c(OC(=O)c7cc(O)c(O)c(O)c7)c6)[C@@H](OC(=O)c8cc(O)c([O-])c(OC(=O)c9cc(O)c(O)c(O)c9)c8)[C@@H]3OC(=O)c%10cc(O)c([O-])c(OC(=O)c%11cc(O)c(O)c(O)c%11)c%10 |
| 2735 | Oc1cc(cc(O)c1O)C(=O)Oc2cc(cc(O)c2O)C(=O)OC[C@H]3O[C@@H](OC(=O)c4cc(O)c([O-])c(OC(=O)c5cc(O)c([O-])c(O)c5)c4)[C@H](OC(=O)c6cc(O)c([O-])c(OC(=O)c7cc(O)c(O)c(O)c7)c6)[C@@H](OC(=O)c8cc(O)c([O-])c(OC(=O)c9cc(O)c([O-])c(O)c9)c8)[C@@H]3OC(=O)c%10cc(O)c([O-])c(OC(=O)c%11cc(O)c(O)c(O)c%11)c%10 |
| 2736 | Oc1cc(cc(O)c1O)C(=O)Oc2cc(cc(O)c2O)C(=O)OC[C@H]3O[C@@H](OC(=O)c4cc(O)c([O-])c(OC(=O)c5cc(O)c([O-])c(O)c5)c4)[C@H](OC(=O)c6cc(O)c([O-])c(OC(=O)c7cc(O)c(O)c(O)c7)c6)[C@@H](OC(=O)c8cc(O)c([O-])c(OC(=O)c9cc(O)c(O)c(O)c9)c8)[C@@H]3OC(=O)c%10cc(O)c([O-])c(OC(=O)c%11cc(O)c([O-])c(O)c%11)c%10 |
| 2737 | Oc1cc(cc(O)c1O)C(=O)Oc2cc(cc(O)c2O)C(=O)OC[C@H]3O[C@@H](OC(=O)c4cc(O)c([O-])c(OC(=O)c5cc(O)c([O-])c(O)c5)c4)[C@H](OC(=O)c6cc(O)c([O-])c(OC(=O)c7cc(O)c(O)c(O)c7)c6)[C@@H](OC(=O)c8cc(O)c([O-])c(OC(=O)c9cc(O)c([O-])c(O)c9)c8)[C@@H]3OC(=O)c%10cc(O)c([O-])c(OC(=O)c%11cc(O)c([O-])c(O)c%11)c%10 |
| 2738 | Oc1cc(cc(O)c1O)C(=O)Oc2cc(cc(O)c2O)C(=O)OC[C@H]3O[C@@H](OC(=O)c4cc(O)c([O-])c(OC(=O)c5cc(O)c([O-])c(O)c5)c4)[C@H](OC(=O)c6cc(O)c([O-])c(OC(=O)c7cc(O)c([O-])c(O)c7)c6)[C@@H](OC(=O)c8cc(O)c([O-])c(OC(=O)c9cc(O)c(O)c(O)c9)c8)[C@@H]3OC(=O)c%10cc(O)c([O-])c(OC(=O)c%11cc(O)c(O)c(O)c%11)c%10 |
| 2739 | Oc1cc(cc(O)c1O)C(=O)Oc2cc(cc(O)c2O)C(=O)OC[C@H]3O[C@@H](OC(=O)c4cc(O)c([O-])c(OC(=O)c5cc(O)c([O-])c(O)c5)c4)[C@H](OC(=O)c6cc(O)c([O-])c(OC(=O)c7cc(O)c([O-])c(O)c7)c6)[C@@H](OC(=O)c8cc(O)c([O-])c(OC(=O)c9cc(O)c([O-])c(O)c9)c8)[C@@H]3OC(=O)c%10cc(O)c([O-])c(OC(=O)c%11cc(O)c(O)c(O)c%11)c%10 |
| 2740 | Oc1cc(cc(O)c1O)C(=O)Oc2cc(cc(O)c2O)C(=O)OC[C@H]3O[C@@H](OC(=O)c4cc(O)c([O-])c(OC(=O)c5cc(O)c([O-])c(O)c5)c4)[C@H](OC(=O)c6cc(O)c([O-])c(OC(=O)c7cc(O)c([O-])c(O)c7)c6)[C@@H](OC(=O)c8cc(O)c([O-])c(OC(=O)c9cc(O)c(O)c(O)c9)c8)[C@@H]3OC(=O)c%10cc(O)c([O-])c(OC(=O)c%11cc(O)c([O-])c(O)c%11)c%10 |
| 2741 | Oc1cc(cc(O)c1O)C(=O)Oc2cc(cc(O)c2O)C(=O)OC[C@H]3O[C@@H](OC(=O)c4cc(O)c([O-])c(OC(=O)c5cc(O)c([O-])c(O)c5)c4)[C@H](OC(=O)c6cc(O)c([O-])c(OC(=O)c7cc(O)c([O-])c(O)c7)c6)[C@@H](OC(=O)c8cc(O)c([O-])c(OC(=O)c9cc(O)c([O-])c(O)c9)c8)[C@@H]3OC(=O)c%10cc(O)c([O-])c(OC(=O)c%11cc(O)c([O-])c(O)c%11)c%10 |
| 2742 | Oc1cc(cc(O)c1O)C(=O)Oc2cc(cc(O)c2[O-])C(=O)O[C@@H]3O[C@H](COC(=O)c4cc(O)c(O)c(OC(=O)c5cc(O)c([O-])c(O)c5)c4)[C@@H](OC(=O)c6cc(O)c([O-])c(OC(=O)c7cc(O)c(O)c(O)c7)c6)[C@H](OC(=O)c8cc(O)c([O-])c(OC(=O)c9cc(O)c(O)c(O)c9)c8)[C@H]3OC(=O)c%10cc(O)c([O-])c(OC(=O)c%11cc(O)c(O)c(O)c%11)c%10 |
| 2743 | Oc1cc(cc(O)c1O)C(=O)Oc2cc(cc(O)c2[O-])C(=O)O[C@@H]3O[C@H](COC(=O)c4cc(O)c(O)c(OC(=O)c5cc(O)c([O-])c(O)c5)c4)[C@@H](OC(=O)c6cc(O)c([O-])c(OC(=O)c7cc(O)c(O)c(O)c7)c6)[C@H](OC(=O)c8cc(O)c([O-])c(OC(=O)c9cc(O)c([O-])c(O)c9)c8)[C@H]3OC(=O)c%10cc(O)c([O-])c(OC(=O)c%11cc(O)c(O)c(O)c%11)c%10 |
| 2744 | Oc1cc(cc(O)c1O)C(=O)Oc2cc(cc(O)c2[O-])C(=O)O[C@@H]3O[C@H](COC(=O)c4cc(O)c(O)c(OC(=O)c5cc(O)c([O-])c(O)c5)c4)[C@@H](OC(=O)c6cc(O)c([O-])c(OC(=O)c7cc(O)c([O-])c(O)c7)c6)[C@H](OC(=O)c8cc(O)c([O-])c(OC(=O)c9cc(O)c(O)c(O)c9)c8)[C@H]3OC(=O)c%10cc(O)c([O-])c(OC(=O)c%11cc(O)c(O)c(O)c%11)c%10 |
| 2745 | Oc1cc(cc(O)c1O)C(=O)Oc2cc(cc(O)c2[O-])C(=O)O[C@@H]3O[C@H](COC(=O)c4cc(O)c(O)c(OC(=O)c5cc(O)c([O-])c(O)c5)c4)[C@@H](OC(=O)c6cc(O)c([O-])c(OC(=O)c7cc(O)c([O-])c(O)c7)c6)[C@H](OC(=O)c8cc(O)c([O-])c(OC(=O)c9cc(O)c([O-])c(O)c9)c8)[C@H]3OC(=O)c%10cc(O)c([O-])c(OC(=O)c%11cc(O)c(O)c(O)c%11)c%10 |
| 2746 | Oc1cc(cc(O)c1O)C(=O)Oc2cc(cc(O)c2[O-])C(=O)O[C@@H]3O[C@H](COC(=O)c4cc(O)c(O)c(OC(=O)c5cc(O)c([O-])c(O)c5)c4)[C@@H](OC(=O)c6cc(O)c([O-])c(OC(=O)c7cc(O)c(O)c(O)c7)c6)[C@H](OC(=O)c8cc(O)c([O-])c(OC(=O)c9cc(O)c(O)c(O)c9)c8)[C@H]3OC(=O)c%10cc(O)c([O-])c(OC(=O)c%11cc(O)c([O-])c(O)c%11)c%10 |
| 2747 | Oc1cc(cc(O)c1O)C(=O)Oc2cc(cc(O)c2[O-])C(=O)O[C@@H]3O[C@H](COC(=O)c4cc(O)c(O)c(OC(=O)c5cc(O)c([O-])c(O)c5)c4)[C@@H](OC(=O)c6cc(O)c([O-])c(OC(=O)c7cc(O)c(O)c(O)c7)c6)[C@H](OC(=O)c8cc(O)c([O-])c(OC(=O)c9cc(O)c([O-])c(O)c9)c8)[C@H]3OC(=O)c%10cc(O)c([O-])c(OC(=O)c%11cc(O)c([O-])c(O)c%11)c%10 |
| 2748 | Oc1cc(cc(O)c1O)C(=O)Oc2cc(cc(O)c2[O-])C(=O)O[C@@H]3O[C@H](COC(=O)c4cc(O)c(O)c(OC(=O)c5cc(O)c([O-])c(O)c5)c4)[C@@H](OC(=O)c6cc(O)c([O-])c(OC(=O)c7cc(O)c([O-])c(O)c7)c6)[C@H](OC(=O)c8cc(O)c([O-])c(OC(=O)c9cc(O)c(O)c(O)c9)c8)[C@H]3OC(=O)c%10cc(O)c([O-])c(OC(=O)c%11cc(O)c([O-])c(O)c%11)c%10 |
| 2749 | Oc1cc(cc(O)c1O)C(=O)Oc2cc(cc(O)c2[O-])C(=O)O[C@@H]3O[C@H](COC(=O)c4cc(O)c(O)c(OC(=O)c5cc(O)c([O-])c(O)c5)c4)[C@@H](OC(=O)c6cc(O)c([O-])c(OC(=O)c7cc(O)c([O-])c(O)c7)c6)[C@H](OC(=O)c8cc(O)c([O-])c(OC(=O)c9cc(O)c([O-])c(O)c9)c8)[C@H]3OC(=O)c%10cc(O)c([O-])c(OC(=O)c%11cc(O)c([O-])c(O)c%11)c%10 |
| 2750 | Oc1cc(cc(O)c1O)C(=O)Oc2cc(cc(O)c2[O-])C(=O)O[C@@H]3[C@@H](COC(=O)c4cc(O)c(O)c(OC(=O)c5cc(O)c([O-])c(O)c5)c4)O[C@@H](OC(=O)c6cc(O)c([O-])c(OC(=O)c7cc(O)c([O-])c(O)c7)c6)[C@H](OC(=O)c8cc(O)c([O-])c(OC(=O)c9cc(O)c(O)c(O)c9)c8)[C@H]3OC(=O)c%10cc(O)c([O-])c(OC(=O)c%11cc(O)c(O)c(O)c%11)c%10 |
| 2751 | Oc1cc(cc(O)c1O)C(=O)Oc2cc(cc(O)c2[O-])C(=O)O[C@@H]3[C@@H](COC(=O)c4cc(O)c(O)c(OC(=O)c5cc(O)c([O-])c(O)c5)c4)O[C@@H](OC(=O)c6cc(O)c([O-])c(OC(=O)c7cc(O)c([O-])c(O)c7)c6)[C@H](OC(=O)c8cc(O)c([O-])c(OC(=O)c9cc(O)c(O)c(O)c9)c8)[C@H]3OC(=O)c%10cc(O)c([O-])c(OC(=O)c%11cc(O)c([O-])c(O)c%11)c%10 |
| 2752 | Oc1cc(cc(O)c1O)C(=O)Oc2cc(cc(O)c2[O-])C(=O)O[C@H]3[C@H](OC(=O)c4cc(O)c([O-])c(OC(=O)c5cc(O)c([O-])c(O)c5)c4)O[C@H](COC(=O)c6cc(O)c(O)c(OC(=O)c7cc(O)c([O-])c(O)c7)c6)[C@@H](OC(=O)c8cc(O)c([O-])c(OC(=O)c9cc(O)c([O-])c(O)c9)c8)[C@@H]3OC(=O)c%10cc(O)c([O-])c(OC(=O)c%11cc(O)c(O)c(O)c%11)c%10 |
| 2753 | Oc1cc(cc(O)c1O)C(=O)Oc2cc(cc(O)c2[O-])C(=O)O[C@H]3[C@H](OC(=O)c4cc(O)c([O-])c(OC(=O)c5cc(O)c([O-])c(O)c5)c4)O[C@H](COC(=O)c6cc(O)c(O)c(OC(=O)c7cc(O)c([O-])c(O)c7)c6)[C@@H](OC(=O)c8cc(O)c([O-])c(OC(=O)c9cc(O)c([O-])c(O)c9)c8)[C@@H]3OC(=O)c%10cc(O)c([O-])c(OC(=O)c%11cc(O)c([O-])c(O)c%11)c%10 |
| 2754 | Oc1cc(cc(O)c1O)C(=O)Oc2cc(cc(O)c2[O-])C(=O)O[C@@H]3[C@@H](COC(=O)c4cc(O)c(O)c(OC(=O)c5cc(O)c([O-])c(O)c5)c4)O[C@@H](OC(=O)c6cc(O)c([O-])c(OC(=O)c7cc(O)c([O-])c(O)c7)c6)[C@H](OC(=O)c8cc(O)c([O-])c(OC(=O)c9cc(O)c([O-])c(O)c9)c8)[C@H]3OC(=O)c%10cc(O)c([O-])c(OC(=O)c%11cc(O)c(O)c(O)c%11)c%10 |
| 2755 | Oc1cc(cc(O)c1O)C(=O)Oc2cc(cc(O)c2[O-])C(=O)O[C@@H]3[C@@H](COC(=O)c4cc(O)c(O)c(OC(=O)c5cc(O)c([O-])c(O)c5)c4)O[C@@H](OC(=O)c6cc(O)c([O-])c(OC(=O)c7cc(O)c([O-])c(O)c7)c6)[C@H](OC(=O)c8cc(O)c([O-])c(OC(=O)c9cc(O)c([O-])c(O)c9)c8)[C@H]3OC(=O)c%10cc(O)c([O-])c(OC(=O)c%11cc(O)c([O-])c(O)c%11)c%10 |
| 2756 | Oc1cc(cc(O)c1O)C(=O)Oc2cc(cc(O)c2[O-])C(=O)O[C@H]3[C@H](OC(=O)c4cc(O)c([O-])c(OC(=O)c5cc(O)c([O-])c(O)c5)c4)[C@@H](COC(=O)c6cc(O)c(O)c(OC(=O)c7cc(O)c([O-])c(O)c7)c6)O[C@@H](OC(=O)c8cc(O)c([O-])c(OC(=O)c9cc(O)c([O-])c(O)c9)c8)[C@@H]3OC(=O)c%10cc(O)c([O-])c(OC(=O)c%11cc(O)c([O-])c(O)c%11)c%10 |
| 2757 | Oc1cc(cc(OC(=O)c2cc(O)c([O-])c(O)c2)c1O)C(=O)OC[C@H]3O[C@@H](OC(=O)c4cc(O)c([O-])c(OC(=O)c5cc(O)c([O-])c(O)c5)c4)[C@H](OC(=O)c6cc(O)c([O-])c(OC(=O)c7cc(O)c([O-])c(O)c7)c6)[C@@H](OC(=O)c8cc(O)c([O-])c(OC(=O)c9cc(O)c([O-])c(O)c9)c8)[C@@H]3OC(=O)c%10cc(O)c([O-])c(OC(=O)c%11cc(O)c([O-])c(O)c%11)c%10 |
| 2758 | Oc1cc(cc(O)c1[O-])C(=O)Oc2cc(cc(O)c2[O-])C(=O)OC[C@H]3O[C@@H](OC(=O)c4cc(O)c([O-])c(OC(=O)c5cc(O)c([O-])c(O)c5)c4)[C@H](OC(=O)c6cc(O)c([O-])c(OC(=O)c7cc(O)c([O-])c(O)c7)c6)[C@@H](OC(=O)c8cc(O)c([O-])c(OC(=O)c9cc(O)c([O-])c(O)c9)c8)[C@@H]3OC(=O)c%10cc(O)c([O-])c(OC(=O)c%11cc(O)c([O-])c(O)c%11)c%10 |
| 2759 | Oc1cc(cc(O)c1[O-])C(=O)Oc2cc(cc(O)c2[O-])C(=O)O[C@@H]3O[C@H](COC(=O)c4cc(O)c([O-])c(OC(=O)c5cc(O)c([O-])c([O-])c5)c4)[C@@H](OC(=O)c6cc(O)c([O-])c(OC(=O)c7cc(O)c([O-])c(O)c7)c6)[C@H](OC(=O)c8cc(O)c([O-])c(OC(=O)c9cc(O)c([O-])c(O)c9)c8)[C@H]3OC(=O)c%10cc(O)c([O-])c(OC(=O)c%11cc(O)c([O-])c(O)c%11)c%10 |
| 2760 | Oc1cc(cc(O)c1[O-])C(=O)Oc2cc(cc(O)c2[O-])C(=O)OC[C@H]3O[C@@H](OC(=O)c4cc(O)c([O-])c(OC(=O)c5cc(O)c([O-])c([O-])c5)c4)[C@H](OC(=O)c6cc(O)c([O-])c(OC(=O)c7cc(O)c([O-])c(O)c7)c6)[C@@H](OC(=O)c8cc(O)c([O-])c(OC(=O)c9cc(O)c([O-])c(O)c9)c8)[C@@H]3OC(=O)c%10cc(O)c([O-])c(OC(=O)c%11cc(O)c([O-])c(O)c%11)c%10 |
| 2761 | Oc1cc(cc(O)c1[O-])C(=O)Oc2cc(cc(O)c2[O-])C(=O)O[C@@H]3[C@@H](COC(=O)c4cc(O)c([O-])c(OC(=O)c5cc(O)c([O-])c([O-])c5)c4)O[C@@H](OC(=O)c6cc(O)c([O-])c(OC(=O)c7cc(O)c([O-])c([O-])c7)c6)[C@H](OC(=O)c8cc(O)c([O-])c(OC(=O)c9cc(O)c([O-])c(O)c9)c8)[C@H]3OC(=O)c%10cc(O)c([O-])c(OC(=O)c%11cc(O)c([O-])c(O)c%11)c%10 |
| 2762 | Oc1cc(cc(O)c1[O-])C(=O)Oc2cc(cc(O)c2[O-])C(=O)OC[C@H]3O[C@@H](OC(=O)c4cc(O)c([O-])c(OC(=O)c5cc(O)c([O-])c(O)c5)c4)[C@H](OC(=O)c6cc(O)c([O-])c(OC(=O)c7cc(O)c([O-])c([O-])c7)c6)[C@@H](OC(=O)c8cc(O)c([O-])c(OC(=O)c9cc(O)c([O-])c(O)c9)c8)[C@@H]3OC(=O)c%10cc(O)c([O-])c(OC(=O)c%11cc(O)c([O-])c(O)c%11)c%10 |
| 2763 | Oc1cc(cc(O)c1[O-])C(=O)Oc2cc(cc(O)c2[O-])C(=O)O[C@@H]3O[C@H](COC(=O)c4cc(O)c([O-])c(OC(=O)c5cc(O)c([O-])c([O-])c5)c4)[C@@H](OC(=O)c6cc(O)c([O-])c(OC(=O)c7cc(O)c([O-])c(O)c7)c6)[C@H](OC(=O)c8cc(O)c([O-])c(OC(=O)c9cc(O)c([O-])c(O)c9)c8)[C@H]3OC(=O)c%10cc(O)c([O-])c(OC(=O)c%11cc(O)c([O-])c([O-])c%11)c%10 |
| 2764 | Oc1cc(cc(O)c1[O-])C(=O)Oc2cc(cc(O)c2[O-])C(=O)OC[C@H]3O[C@@H](OC(=O)c4cc(O)c([O-])c(OC(=O)c5cc(O)c([O-])c([O-])c5)c4)[C@H](OC(=O)c6cc(O)c([O-])c(OC(=O)c7cc(O)c([O-])c([O-])c7)c6)[C@@H](OC(=O)c8cc(O)c([O-])c(OC(=O)c9cc(O)c([O-])c(O)c9)c8)[C@@H]3OC(=O)c%10cc(O)c([O-])c(OC(=O)c%11cc(O)c([O-])c(O)c%11)c%10 |
| 2765 | Oc1cc(cc(O)c1[O-])C(=O)Oc2cc(cc(O)c2[O-])C(=O)O[C@@H]3[C@@H](COC(=O)c4cc(O)c([O-])c(OC(=O)c5cc(O)c([O-])c([O-])c5)c4)O[C@@H](OC(=O)c6cc(O)c([O-])c(OC(=O)c7cc(O)c([O-])c([O-])c7)c6)[C@H](OC(=O)c8cc(O)c([O-])c(OC(=O)c9cc(O)c([O-])c([O-])c9)c8)[C@H]3OC(=O)c%10cc(O)c([O-])c(OC(=O)c%11cc(O)c([O-])c(O)c%11)c%10 |
| 2766 | Oc1cc(cc(O)c1[O-])C(=O)Oc2cc(cc(O)c2[O-])C(=O)OC[C@H]3O[C@@H](OC(=O)c4cc(O)c([O-])c(OC(=O)c5cc(O)c([O-])c(O)c5)c4)[C@H](OC(=O)c6cc(O)c([O-])c(OC(=O)c7cc(O)c([O-])c(O)c7)c6)[C@@H](OC(=O)c8cc(O)c([O-])c(OC(=O)c9cc(O)c([O-])c(O)c9)c8)[C@@H]3OC(=O)c%10cc(O)c([O-])c(OC(=O)c%11cc(O)c([O-])c([O-])c%11)c%10 |
| 2767 | Oc1cc(cc(O)c1[O-])C(=O)Oc2cc(cc(O)c2[O-])C(=O)O[C@@H]3O[C@H](COC(=O)c4cc(O)c([O-])c(OC(=O)c5cc(O)c([O-])c([O-])c5)c4)[C@@H](OC(=O)c6cc(O)c([O-])c(OC(=O)c7cc(O)c([O-])c([O-])c7)c6)[C@H](OC(=O)c8cc(O)c([O-])c(OC(=O)c9cc(O)c([O-])c(O)c9)c8)[C@H]3OC(=O)c%10cc(O)c([O-])c(OC(=O)c%11cc(O)c([O-])c(O)c%11)c%10 |
| 2768 | Oc1cc(cc(O)c1[O-])C(=O)Oc2cc(cc(O)c2[O-])C(=O)OC[C@H]3O[C@@H](OC(=O)c4cc(O)c([O-])c(OC(=O)c5cc(O)c([O-])c([O-])c5)c4)[C@H](OC(=O)c6cc(O)c([O-])c(OC(=O)c7cc(O)c([O-])c(O)c7)c6)[C@@H](OC(=O)c8cc(O)c([O-])c(OC(=O)c9cc(O)c([O-])c(O)c9)c8)[C@@H]3OC(=O)c%10cc(O)c([O-])c(OC(=O)c%11cc(O)c([O-])c([O-])c%11)c%10 |
| 2769 | Oc1cc(cc(O)c1[O-])C(=O)Oc2cc(cc(O)c2[O-])C(=O)O[C@H]3[C@H](OC(=O)c4cc(O)c([O-])c(OC(=O)c5cc(O)c([O-])c([O-])c5)c4)O[C@H](COC(=O)c6cc(O)c([O-])c(OC(=O)c7cc(O)c([O-])c([O-])c7)c6)[C@@H](OC(=O)c8cc(O)c([O-])c(OC(=O)c9cc(O)c([O-])c([O-])c9)c8)[C@@H]3OC(=O)c%10cc(O)c([O-])c(OC(=O)c%11cc(O)c([O-])c(O)c%11)c%10 |
| 2770 | Oc1cc(cc(O)c1[O-])C(=O)Oc2cc(cc(O)c2[O-])C(=O)OC[C@H]3O[C@@H](OC(=O)c4cc(O)c([O-])c(OC(=O)c5cc(O)c([O-])c(O)c5)c4)[C@H](OC(=O)c6cc(O)c([O-])c(OC(=O)c7cc(O)c([O-])c([O-])c7)c6)[C@@H](OC(=O)c8cc(O)c([O-])c(OC(=O)c9cc(O)c([O-])c(O)c9)c8)[C@@H]3OC(=O)c%10cc(O)c([O-])c(OC(=O)c%11cc(O)c([O-])c([O-])c%11)c%10 |
| 2771 | Oc1cc(cc(O)c1[O-])C(=O)Oc2cc(cc(O)c2[O-])C(=O)O[C@@H]3O[C@H](COC(=O)c4cc(O)c([O-])c(OC(=O)c5cc(O)c([O-])c([O-])c5)c4)[C@@H](OC(=O)c6cc(O)c([O-])c(OC(=O)c7cc(O)c([O-])c([O-])c7)c6)[C@H](OC(=O)c8cc(O)c([O-])c(OC(=O)c9cc(O)c([O-])c(O)c9)c8)[C@H]3OC(=O)c%10cc(O)c([O-])c(OC(=O)c%11cc(O)c([O-])c([O-])c%11)c%10 |
| 2772 | Oc1cc(cc(O)c1[O-])C(=O)Oc2cc(cc(O)c2[O-])C(=O)OC[C@H]3O[C@@H](OC(=O)c4cc(O)c([O-])c(OC(=O)c5cc(O)c([O-])c([O-])c5)c4)[C@H](OC(=O)c6cc(O)c([O-])c(OC(=O)c7cc(O)c([O-])c([O-])c7)c6)[C@@H](OC(=O)c8cc(O)c([O-])c(OC(=O)c9cc(O)c([O-])c(O)c9)c8)[C@@H]3OC(=O)c%10cc(O)c([O-])c(OC(=O)c%11cc(O)c([O-])c([O-])c%11)c%10 |
| 2773 | Oc1cc(cc(O)c1[O-])C(=O)Oc2cc(cc(O)c2[O-])C(=O)O[C@H]3[C@H](OC(=O)c4cc(O)c([O-])c(OC(=O)c5cc(O)c([O-])c([O-])c5)c4)[C@@H](COC(=O)c6cc(O)c([O-])c(OC(=O)c7cc(O)c([O-])c([O-])c7)c6)O[C@@H](OC(=O)c8cc(O)c([O-])c(OC(=O)c9cc(O)c([O-])c([O-])c9)c8)[C@@H]3OC(=O)c%10cc(O)c([O-])c(OC(=O)c%11cc(O)c([O-])c([O-])c%11)c%10 |
| 2774 | Oc1cc(cc(O)c1[O-])C(=O)Oc2cc(cc(O)c2[O-])C(=O)OC[C@H]3O[C@@H](OC(=O)c4cc(O)c([O-])c(OC(=O)c5cc(O)c([O-])c(O)c5)c4)[C@H](OC(=O)c6cc(O)c([O-])c(OC(=O)c7cc(O)c([O-])c(O)c7)c6)[C@@H](OC(=O)c8cc(O)c([O-])c(OC(=O)c9cc(O)c([O-])c([O-])c9)c8)[C@@H]3OC(=O)c%10cc(O)c([O-])c(OC(=O)c%11cc(O)c([O-])c(O)c%11)c%10 |
| 2775 | Oc1cc(cc(O)c1[O-])C(=O)Oc2cc(cc(O)c2[O-])C(=O)O[C@@H]3O[C@H](COC(=O)c4cc(O)c([O-])c(OC(=O)c5cc(O)c([O-])c([O-])c5)c4)[C@@H](OC(=O)c6cc(O)c([O-])c(OC(=O)c7cc(O)c([O-])c(O)c7)c6)[C@H](OC(=O)c8cc(O)c([O-])c(OC(=O)c9cc(O)c([O-])c([O-])c9)c8)[C@H]3OC(=O)c%10cc(O)c([O-])c(OC(=O)c%11cc(O)c([O-])c(O)c%11)c%10 |
| 2776 | Oc1cc(cc(O)c1[O-])C(=O)Oc2cc(cc(O)c2[O-])C(=O)OC[C@H]3O[C@@H](OC(=O)c4cc(O)c([O-])c(OC(=O)c5cc(O)c([O-])c([O-])c5)c4)[C@H](OC(=O)c6cc(O)c([O-])c(OC(=O)c7cc(O)c([O-])c(O)c7)c6)[C@@H](OC(=O)c8cc(O)c([O-])c(OC(=O)c9cc(O)c([O-])c([O-])c9)c8)[C@@H]3OC(=O)c%10cc(O)c([O-])c(OC(=O)c%11cc(O)c([O-])c(O)c%11)c%10 |
| 2777 | Oc1cc(cc(O)c1[O-])C(=O)Oc2cc(cc(O)c2[O-])C(=O)O[C@@H]3[C@@H](COC(=O)c4cc(O)c([O-])c(OC(=O)c5cc(O)c([O-])c([O-])c5)c4)O[C@@H](OC(=O)c6cc(O)c([O-])c(OC(=O)c7cc(O)c([O-])c([O-])c7)c6)[C@H](OC(=O)c8cc(O)c([O-])c(OC(=O)c9cc(O)c([O-])c(O)c9)c8)[C@H]3OC(=O)c%10cc(O)c([O-])c(OC(=O)c%11cc(O)c([O-])c([O-])c%11)c%10 |
| 2778 | Oc1cc(cc(O)c1[O-])C(=O)Oc2cc(cc(O)c2[O-])C(=O)OC[C@H]3O[C@@H](OC(=O)c4cc(O)c([O-])c(OC(=O)c5cc(O)c([O-])c(O)c5)c4)[C@H](OC(=O)c6cc(O)c([O-])c(OC(=O)c7cc(O)c([O-])c([O-])c7)c6)[C@@H](OC(=O)c8cc(O)c([O-])c(OC(=O)c9cc(O)c([O-])c([O-])c9)c8)[C@@H]3OC(=O)c%10cc(O)c([O-])c(OC(=O)c%11cc(O)c([O-])c(O)c%11)c%10 |
| 2779 | Oc1cc(cc(O)c1[O-])C(=O)Oc2cc(cc(O)c2[O-])C(=O)O[C@@H]3O[C@H](COC(=O)c4cc(O)c([O-])c(OC(=O)c5cc(O)c([O-])c([O-])c5)c4)[C@@H](OC(=O)c6cc(O)c([O-])c(OC(=O)c7cc(O)c([O-])c(O)c7)c6)[C@H](OC(=O)c8cc(O)c([O-])c(OC(=O)c9cc(O)c([O-])c([O-])c9)c8)[C@H]3OC(=O)c%10cc(O)c([O-])c(OC(=O)c%11cc(O)c([O-])c([O-])c%11)c%10 |
| 2780 | Oc1cc(cc(O)c1[O-])C(=O)Oc2cc(cc(O)c2[O-])C(=O)OC[C@H]3O[C@@H](OC(=O)c4cc(O)c([O-])c(OC(=O)c5cc(O)c([O-])c([O-])c5)c4)[C@H](OC(=O)c6cc(O)c([O-])c(OC(=O)c7cc(O)c([O-])c([O-])c7)c6)[C@@H](OC(=O)c8cc(O)c([O-])c(OC(=O)c9cc(O)c([O-])c([O-])c9)c8)[C@@H]3OC(=O)c%10cc(O)c([O-])c(OC(=O)c%11cc(O)c([O-])c(O)c%11)c%10 |
| 2781 | Oc1cc(cc(O)c1[O-])C(=O)Oc2cc(cc(O)c2[O-])C(=O)O[C@@H]3[C@@H](COC(=O)c4cc(O)c([O-])c(OC(=O)c5cc(O)c([O-])c([O-])c5)c4)O[C@@H](OC(=O)c6cc(O)c([O-])c(OC(=O)c7cc(O)c([O-])c([O-])c7)c6)[C@H](OC(=O)c8cc(O)c([O-])c(OC(=O)c9cc(O)c([O-])c([O-])c9)c8)[C@H]3OC(=O)c%10cc(O)c([O-])c(OC(=O)c%11cc(O)c([O-])c([O-])c%11)c%10 |
| 2782 | Oc1cc(cc(O)c1[O-])C(=O)Oc2cc(cc(O)c2[O-])C(=O)OC[C@H]3O[C@@H](OC(=O)c4cc(O)c([O-])c(OC(=O)c5cc(O)c([O-])c(O)c5)c4)[C@H](OC(=O)c6cc(O)c([O-])c(OC(=O)c7cc(O)c([O-])c(O)c7)c6)[C@@H](OC(=O)c8cc(O)c([O-])c(OC(=O)c9cc(O)c([O-])c([O-])c9)c8)[C@@H]3OC(=O)c%10cc(O)c([O-])c(OC(=O)c%11cc(O)c([O-])c([O-])c%11)c%10 |
| 2783 | Oc1cc(cc(O)c1[O-])C(=O)Oc2cc(cc(O)c2[O-])C(=O)O[C@@H]3O[C@H](COC(=O)c4cc(O)c([O-])c(OC(=O)c5cc(O)c([O-])c([O-])c5)c4)[C@@H](OC(=O)c6cc(O)c([O-])c(OC(=O)c7cc(O)c([O-])c([O-])c7)c6)[C@H](OC(=O)c8cc(O)c([O-])c(OC(=O)c9cc(O)c([O-])c([O-])c9)c8)[C@H]3OC(=O)c%10cc(O)c([O-])c(OC(=O)c%11cc(O)c([O-])c(O)c%11)c%10 |
| 2784 | Oc1cc(cc(O)c1[O-])C(=O)Oc2cc(cc(O)c2[O-])C(=O)OC[C@H]3O[C@@H](OC(=O)c4cc(O)c([O-])c(OC(=O)c5cc(O)c([O-])c([O-])c5)c4)[C@H](OC(=O)c6cc(O)c([O-])c(OC(=O)c7cc(O)c([O-])c(O)c7)c6)[C@@H](OC(=O)c8cc(O)c([O-])c(OC(=O)c9cc(O)c([O-])c([O-])c9)c8)[C@@H]3OC(=O)c%10cc(O)c([O-])c(OC(=O)c%11cc(O)c([O-])c([O-])c%11)c%10 |
| 2785 | Oc1cc(cc(O)c1[O-])C(=O)Oc2cc(cc(O)c2[O-])C(=O)O[C@H]3[C@H](OC(=O)c4cc(O)c([O-])c(OC(=O)c5cc(O)c([O-])c([O-])c5)c4)O[C@H](COC(=O)c6cc(O)c([O-])c(OC(=O)c7cc(O)c([O-])c([O-])c7)c6)[C@@H](OC(=O)c8cc(O)c([O-])c(OC(=O)c9cc(O)c([O-])c([O-])c9)c8)[C@@H]3OC(=O)c%10cc(O)c([O-])c(OC(=O)c%11cc(O)c([O-])c([O-])c%11)c%10 |
| 2786 | Oc1cc(cc(O)c1[O-])C(=O)Oc2cc(cc(O)c2[O-])C(=O)OC[C@H]3O[C@@H](OC(=O)c4cc(O)c([O-])c(OC(=O)c5cc(O)c([O-])c(O)c5)c4)[C@H](OC(=O)c6cc(O)c([O-])c(OC(=O)c7cc(O)c([O-])c([O-])c7)c6)[C@@H](OC(=O)c8cc(O)c([O-])c(OC(=O)c9cc(O)c([O-])c([O-])c9)c8)[C@@H]3OC(=O)c%10cc(O)c([O-])c(OC(=O)c%11cc(O)c([O-])c([O-])c%11)c%10 |
| 2787 | Oc1cc(cc(O)c1[O-])C(=O)Oc2cc(cc(O)c2[O-])C(=O)O[C@@H]3O[C@H](COC(=O)c4cc(O)c([O-])c(OC(=O)c5cc(O)c([O-])c([O-])c5)c4)[C@@H](OC(=O)c6cc(O)c([O-])c(OC(=O)c7cc(O)c([O-])c([O-])c7)c6)[C@H](OC(=O)c8cc(O)c([O-])c(OC(=O)c9cc(O)c([O-])c([O-])c9)c8)[C@H]3OC(=O)c%10cc(O)c([O-])c(OC(=O)c%11cc(O)c([O-])c([O-])c%11)c%10 |
| 2788 | Oc1cc(cc(O)c1[O-])C(=O)Oc2cc(cc(O)c2[O-])C(=O)OC[C@H]3O[C@@H](OC(=O)c4cc(O)c([O-])c(OC(=O)c5cc(O)c([O-])c([O-])c5)c4)[C@H](OC(=O)c6cc(O)c([O-])c(OC(=O)c7cc(O)c([O-])c([O-])c7)c6)[C@@H](OC(=O)c8cc(O)c([O-])c(OC(=O)c9cc(O)c([O-])c([O-])c9)c8)[C@@H]3OC(=O)c%10cc(O)c([O-])c(OC(=O)c%11cc(O)c([O-])c([O-])c%11)c%10 |
| 2789 | Oc1cc(cc([O-])c1[O-])C(=O)Oc2cc(cc(O)c2[O-])C(=O)OC[C@H]3O[C@@H](OC(=O)c4cc(O)c([O-])c(OC(=O)c5cc(O)c([O-])c([O-])c5)c4)[C@H](OC(=O)c6cc(O)c([O-])c(OC(=O)c7cc(O)c([O-])c([O-])c7)c6)[C@@H](OC(=O)c8cc(O)c([O-])c(OC(=O)c9cc(O)c([O-])c([O-])c9)c8)[C@@H]3OC(=O)c%10cc(O)c([O-])c(OC(=O)c%11cc(O)c([O-])c([O-])c%11)c%10 |
| 2790 | Oc1cc(O)c2cc(O)c([o+]c2c1)c3ccc(O)c(O)c3 |
| 2791 | COc1ccc(C(=O)C)c(O[C@@H]2O[C@H](CO[C@@H]3OC[C@](O)(CO)[C@H]3O)[C@@H](O)[C@H](O)[C@H]2O)c1 |
| 2792 | COc1ccc(C(=O)C)c(O[C@@H]2O[C@H](CO)[C@@H](O)[C@H](O)[C@H]2O)c1 |
| 2793 | COc1ccc(C(=O)C)c(O[C@@H]2O[C@H](CO[C@@H]3OC[C@](O)(COC(=O)c4cc(O)c(O)c(O)c4)[C@H]3O)[C@@H](O)[C@H](O)[C@H]2O)c1 |
| 2794 | COc1ccc(C(=O)C)c(O[C@@H]2O[C@H](CO[C@@H]3OC[C@](O)(COC(=O)c4cc(O)c([O-])c(O)c4)[C@H]3O)[C@@H](O)[C@H](O)[C@H]2O)c1 |
| 2795 | COc1ccc(C(=O)C)c(O[C@@H]2O[C@H](CO[C@@H]3OC[C@](O)(CO)[C@H]3O)[C@@H](OC(=O)c4cc(O)c(O)c(O)c4)[C@H](O)[C@H]2O)c1 |
| 2796 | COc1ccc(C(=O)C)c(O[C@@H]2O[C@H](CO[C@@H]3OC[C@](O)(CO)[C@H]3O)[C@@H](OC(=O)c4cc(O)c([O-])c(O)c4)[C@H](O)[C@H]2O)c1 |
| 2797 | COc1ccc(C(=O)C)c(O[C@@H]2O[C@H](CO[C@@H]3OC[C@H](OC(=O)c4cc(O)c(O)c(O)c4)[C@H](O)[C@H]3O)[C@@H](O)[C@H](O)[C@H]2O)c1 |
| 2798 | COc1ccc(C(=O)C)c(O[C@@H]2O[C@H](CO[C@@H]3OC[C@H](OC(=O)c4cc(O)c([O-])c(O)c4)[C@H](O)[C@H]3O)[C@@H](O)[C@H](O)[C@H]2O)c1 |
| 2799 | COc1ccc(C(=O)C)c(O[C@@H]2O[C@H](CO[C@@H]3OC[C@H](O)[C@H](O)[C@H]3O)[C@@H](OC(=O)c4cc(O)c(O)c(O)c4)[C@H](O)[C@H]2O)c1 |
| 2800 | COc1ccc(C(=O)C)c(O[C@@H]2O[C@H](CO[C@@H]3OC[C@H](O)[C@H](O)[C@H]3O)[C@@H](OC(=O)c4cc(O)c([O-])c(O)c4)[C@H](O)[C@H]2O)c1 |
| 2801 | Oc1ccc(cc1)[C@@H]2[C@@H]3[C@H](c4ccc(O)cc4)c5cc(O)ccc5[C@H]6[C@H](Oc7cc([O-])c([C@H]2c8cc(O)cc([O-])c8)c3c67)c9cc(O)cc(O)c9 |
| 2802 | Oc1ccc(cc1)[C@@H]2[C@@H]3[C@H](c4ccc(O)cc4)c5cc(O)ccc5[C@H]6[C@H](Oc7cc([O-])c([C@H]2c8cc(O)cc([O-])c8)c3c67)c9cc(O)cc([O-])c9 |
| 2803 | Oc1ccc(cc1)[C@@H]2[C@@H]3[C@H](c4ccc(O)cc4)c5cc([O-])ccc5[C@H]6[C@H](Oc7cc([O-])c([C@H]2c8cc(O)cc([O-])c8)c3c67)c9cc(O)cc([O-])c9 |
| 2804 | Oc1ccc(cc1)[C@@H]2[C@@H]3[C@H](c4ccc(O)cc4)c5cc([O-])ccc5[C@H]6[C@H](Oc7cc([O-])c([C@H]2c8cc([O-])cc([O-])c8)c3c67)c9cc(O)cc([O-])c9 |
| 2805 | C[C@@H]1CCC(=C(C)C)[C@]12CCC(=CC2)C |
| 2806 | C[C@@H]1CCC(=C(C)C)[C@@]12CCC(=CC2)C |
| 2807 | C\C=C(\C)/C=C\C=C(C)C |
| 2808 | CCC\C=C\1/OC(=O)C2=C1CC[C@@H]3[C@@H](CCC)[C@]4(OC(=O)C5=C4CCC=C5)[C@H]23 |
| 2809 | COc1cc(OC)c(C(=O)C=C(C)C)c2OC(=O)C=Cc12 |
| 2810 | C\C=C(\C)/C(=O)O[C@@H]1[C@H](O)c2c(OC1(C)C)ccc3C=CC(=O)Oc23 |
| 2811 | C\C=C(\C)/C(=O)O[C@@H]1C=C(C)CC[C@]2(O)[C@H](CC[C@]12C)C(C)C |
| 2812 | OC[C@@]1(O)CO[C@@H](OC[C@H]2O[C@@H](Oc3ccc4C=CC(=O)Oc4c3)[C@H](O)[C@@H](O)[C@@H]2O)[C@@H]1O |
| 2813 | CC(C)(O[C@@H]1O[C@H](CO)[C@@H](O)[C@H](O)[C@H]1O)[C@H]2Oc3ccc4C=CC(=O)Oc4c3[C@H]2O |
| 2814 | C\C=C(/C)\C(=O)O[C@H]1[C@H](Oc2ccc3C=CC(=O)Oc3c12)C(C)(C)OC(=O)\C(=C/C)\C |
| 2815 | CC(C)(O)[C@H](O)COc1c2C=CC(=O)Oc2cc3occc13 |
| 2816 | [O-]C(=O)CCCCCCCC(=O)[O-] |
| 2817 | CC(=C)[C@H]1[C@H]2[C@@H](CC[C@]1(C)C=C)C2(C)C |
| 2818 | C[C@@H]1CCC\C=C\[C@H]2C[C@@H](O)C[C@@H]2[C@@H](O)\C=C\C(=O)O1 |
| 2819 | CC1(C)[C@H](CC[C@]1(C)C(=O)[O-])C(=O)[O-] |
| 2820 | OC[C@H]1O[C@@H](Oc2cc3occc3cc2CCC(=O)[O-])[C@H](O)[C@@H](O)[C@@H]1O |
| 2821 | Cc1ccccc1O |
| 2822 | CC(=CC(=O)O[C@H]1Cc2cc3C=CC(=O)Oc3cc2OC1(C)C)C |
| 2823 | CC1(C)Oc2cc3OC(=O)C=Cc3cc2C[C@@H]1O |
| 2824 | CC(=CCc1cc2C=CC(=O)Oc2cc1O)C |
| 2825 | CC(=CCc1cc2C=CC(=O)Oc2cc1[O-])C |
| 2826 | Cc1cccc(O)c1C |
| 2827 | O=C1OC(=O)C2=CC=CC[C@H]12 |
| 2828 | CC(=O)c1ccc(O)cc1O |
| 2829 | CC(=O)c1ccc([O-])cc1O |
| 2830 | C[C@@]12CCC[C@](C)([C@H]1CC[C@@]34C[C@@H](CC[C@@H]23)[C@@](O)(CO)C4)C(=O)O |
| 2831 | C[C@@]12CCC[C@](C)([C@H]1CC[C@@]34C[C@@H](CC[C@@H]23)[C@@](O)(CO)C4)C(=O)[O-] |
| 2832 | COc1c(CC=C)cc2OCOc2c1OC |
| 2833 | COC(=O)CCCCCCCC(=O)OC |
| 2834 | COC(=O)[C@H]1CC[C@@](C)(C(=O)OC)C1(C)C |
| 2835 | C[C@H]1CC[C@H](C(=C)C)[C@]12CCC(=CC2)C |
| 2836 | COC(=O)CCCCCCCCC(=O)OC |
| 2837 | CC(=CCC[C@](C)(O)[C@@H]1CCC(=CC1)C)C |
| 2838 | C[C@H]1O[C@@]1(C)C(=O)O[C@H]2Cc3cc4C=CC(=O)Oc4cc3OC2(C)C |
| 2839 | C[C@@H]1O[C@]1(C)C(=O)O[C@H]2Cc3cc4C=CC(=O)Oc4cc3OC2(C)C |
| 2840 | C[C@@H]1O[C@]1(C)C(=O)O[C@@H]2[C@H](OC(=O)C)c3c(OC2(C)C)ccc4C=CC(=O)Oc34 |
| 2841 | CCOc1ccc(cc1OC)C(=O)[O-] |
| 2842 | CCc1cccc(O)c1 |
| 2843 | CCc1ccc(O)cc1 |
| 2844 | CCc1ccc(O)cc1O |
| 2845 | NC1=NC2=C(N(C=O)[C@@H](CNc3ccc(cc3)C(=O)N[C@@H](CCC(=O)[O-])CC(=O)[O-])CN2)C(=O)N1 |
| 2846 | CCCC\C=C\1/OC(=O)C2=C1CC[C@H](O)[C@@H]2O |
| 2847 | CCCC\C=C\1/OC(=O)C2=C1CC[C@@H](O)[C@@H]2O |
| 2848 | Oc1ccc(CC#N)cc1 |
| 2849 | C\C(=C/C(=O)O[C@H]1Cc2cc3C=CC(=O)Oc3cc2OC1(C)C)\CO |
| 2850 | COc1ccc(C(=O)\C=C\c2ccc(O)cc2)c(O)c1CC=C(C)C |
| 2851 | CC(=C)[C@H](O)Cc1cc2C=CC(=O)Oc2cc1[O-] |
| 2852 | C[C@@]1(CC[C@@H]2C(=C1)[C@H](O)C[C@H]3[C@@]2(C)CCC[C@@]3(C)C(=O)O)C=C |
| 2853 | C[C@@]1(CC[C@@H]2C(=C1)[C@H](O)C[C@H]3[C@@]2(C)CCC[C@@]3(C)C(=O)[O-])C=C |
| 2854 | C[C@@]1(CC[C@@H]2C(=C1)[C@@H](O)C[C@H]3[C@@]2(C)CCC[C@@]3(C)C(=O)O)C=C |
| 2855 | C[C@@]1(CC[C@@H]2C(=C1)[C@@H](O)C[C@H]3[C@@]2(C)CCC[C@@]3(C)C(=O)[O-])C=C |
| 2856 | C\C(=C/CO)\C(=O)O[C@H]1Cc2cc3C=CC(=O)Oc3cc2OC1(C)C |
| 2857 | CC[C@@H](C)C(=O)O[C@H]1[C@@H](OC(=O)\C(=C\C)\C)C(C)(C)Oc2ccc3C=CC(=O)Oc3c12 |
| 2858 | CC(C)(O)[C@@H]1Cc2c(O1)ccc(CCC(=O)[O-])c2O[C@@H]3O[C@H](CO)[C@@H](O)[C@H](O)[C@H]3O |
| 2859 | COc1cc(ccc1O)[C@@H](O)[C@H](CO)Oc2ccc(\C=C\CO[C@@H]3O[C@H](CO)[C@@H](O)[C@H](O)[C@H]3O)cc2OC |
| 2860 | CC(C)(O)[C@@H]1Oc2ccc(CCC(=O)[O-])c(O[C@@H]3O[C@H](CO)[C@@H](O)[C@H](O)[C@H]3O)c2[C@@H]1O |
| 2861 | CC(C)(O)[C@@H]1Cc2cc(CCC(=O)[O-])c(O[C@@H]3O[C@H](CO)[C@@H](O)[C@H](O)[C@H]3O)cc2O1 |
| 2862 | C[C@@H]1O[C@]1(C)C(=O)O[C@@H]2[C@H](OC(=O)C)c3c(OC2(C)C)ccc4C=CC(=O)Oc34 |
| 2863 | COc1cc(\C=C\C)ccc1O |
| 2864 | C\C=C(\C)/C(=O)O[C@@H]1[C@H](OC(=O)C)c2c(OC1(C)C)ccc3C=CC(=O)Oc23 |
| 2865 | C\C=C(\C)/C(=O)O[C@@H]1Cc2c(OC1(C)C)ccc3C=CC(=O)Oc23 |
| 2866 | C[C@@]12CCC[C@](C)([C@H]1CC[C@@]34C[C@@H](CC[C@@H]23)C(=C)C4)C(=O)O |
| 2867 | C[C@@]12CCC[C@](C)([C@H]1CC[C@@]34C[C@@H](CC[C@@H]23)C(=C)C4)C(=O)[O-] |
| 2868 | C[C@@]1(CC[C@@H]2C(=C1)C(=O)C[C@H]3[C@@]2(C)CCC[C@@]3(C)C(=O)O)C=C |
| 2869 | C[C@@]1(CC[C@@H]2C(=C1)C(=O)C[C@H]3[C@@]2(C)CCC[C@@]3(C)C(=O)[O-])C=C |
| 2870 | CC(=C)c1oc2ccc3C=CC(=O)Oc3c2c1 |
| 2871 | COc1cc(OCC=C(C)C)c2C=CC(=O)Oc2c1 |
| 2872 | COc1c2C=CC(=O)Oc2c(OC[C@@H](O)C(C)(C)OC)c3occc13 |
| 2873 | CCCCCCCC(=O)CCC(C)C |
| 2874 | CC(C)(O[C@@H]1O[C@H](CO)[C@@H](O)[C@H](O)[C@H]1O)[C@H]2Cc3cc4C=CC(=O)Oc4cc3O2 |
| 2875 | C\C=C(\C)/C(=O)O[C@@H](COc1c2C=CC(=O)Oc2cc3occc13)C(C)(C)O |
| 2876 | CC1(C)O[C@@H]1COc2c3C=CC(=O)Oc3cc4occc24 |
| 2877 | CC1(C)O[C@H]1COc2c3C=CC(=O)Oc3cc4occc24 |
| 2878 | CC(C)(O)[C@H](O)Cc1cc2C=CC(=O)Oc2cc1O[C@@H]3O[C@H](CO)[C@@H](O)[C@H](O)[C@H]3O |
| 2879 | CC(C)(O)C(=O)Cc1cc2C=CC(=O)Oc2cc1[O-] |
| 2880 | CC(C)[C@@H]1CCC(=C)C=C1 |
| 2881 | O=C1OC(=O)c2ccccc12 |
| 2882 | C[C@@]1(CC[C@@H]2C(=C1)CC[C@H]3[C@@]2(C)CCC[C@@]3(C)C(=O)O)C=C |
| 2883 | C[C@@]1(CC[C@@H]2C(=C1)CC[C@H]3[C@@]2(C)CCC[C@@]3(C)C(=O)[O-])C=C |
| 2884 | C[C@@]1(CO)CCC[C@@]2(C)[C@@H]3CC[C@](C)(C=C)C=C3CC[C@H]12 |
| 2885 | COc1c(OC)c2occc2c3OC(=O)C=Cc13 |
| 2886 | CC1(C)Oc2ccc3C=CC(=O)Oc3c2[C@@H](O)[C@H]1O[C@@H]4O[C@H](CO)[C@@H](O)[C@H](O)[C@H]4O |
| 2887 | C\C=C(\C)/C(=O)O[C@H]1[C@@H](OC(=O)C)C(C)(C)Oc2ccc3C=CC(=O)Oc3c12 |
| 2888 | C\C=C(/C)\C(=O)O[C@H]1[C@@H](O)C(C)(C)Oc2ccc3C=CC(=O)Oc3c12 |
| 2889 | [O-]C(=O)CCCCCCCCC(=O)[O-] |
| 2890 | CC[C@H](\C=C\[C@@H](C)[C@H]1CC[C@H]2[C@@H]3CC=C4C[C@H](CC[C@]4(C)[C@H]3CC[C@]12C)O[C@@H]5O[C@H](CO)[C@@H](O)[C@H](O)[C@H]5O)C(C)C |
| 2891 | CC1(C)CC[C@@]2(CC[C@]3(C)C(=CC[C@H]4[C@@]5(C)CC[C@H](O[C@@H]6O[C@@H]([C@@H](O[C@@H]7OC[C@@H](O)[C@H](O)[C@H]7O)[C@H](O)[C@H]6O)C(=O)[O-])C(C)(C)[C@H]5CC[C@@]34C)[C@@H]2C1)C(=O)O[C@@H]8O[C@H](CO)[C@@H](O)[C@H](O)[C@H]8O |
| 2892 | CCC\C=C\1/OC(=O)C2=C1CC[C@H]3[C@H]2[C@@]45C=CCC[C@@H]4[C@@]3(OC5=O)C(=O)CCC |
| 2893 | Cc1cc(C)c(C=O)c(C)c1 |
| 2894 | CC1=CC(C)(C)C(=CC1=O)C=O |
| 2895 | CCCCC(=O)c1ccccc1C(=O)[O-] |
| 2896 | CC1=CC(=O)c2c(O)c3C[C@H](Oc3cc2O1)C(C)(C)O |
| 2897 | CC1(C)Oc2cc3OC(=O)C=Cc3cc2C=C1 |
| 2898 | CCCCOC(=O)NC(=O)N |
| 2899 | CCCCOC(=O)[N-]C(=O)N |
| 2900 | COc1ccc(cc1OC)[C@@H]2[C@@H](O)[C@H](O)[C@@H](C)[N@H+]2C |
| 2901 | [O-]C(=O)C1=NC=COC1 |
| 2902 | CC(C)C[C@@]12C[C@]1(CC(C)C)C(=O)C(C)(C)C2=O |
| 2903 | CCO[C@]1(CO)OC[C@@H](O)[C@@H](O)[C@@H]1O |
| 2904 | OC[C@@]1(O)OC[C@@H](O)[C@@H](O)[C@@H]1O |
| 2905 | [O-]C(=O)c1occc1 |
| 2906 | CCC\C=C\CO[C@@H]1OC[C@H](O)[C@H](O)[C@H]1O[C@@H]2O[C@H](CO)[C@@H](O)[C@H](O)[C@H]2O |
| 2907 | CCC\C=C\CO[C@@H]1O[C@H](CO)[C@@H](O)[C@H](O)[C@H]1O |
| 2908 | CC\C=C/CCO[C@@H]1O[C@H](CO)[C@@H](O)[C@H](O)[C@H]1O |
| 2909 | CCC\C=C\CO[C@@H]1O[C@H](CO)[C@@H](O)[C@H](O)[C@H]1O[C@@H]2O[C@H](CO)[C@@H](O)[C@H](O)[C@H]2O |
| 2910 | CCCCCCO[C@@H]1O[C@H](CO)[C@@H](O)[C@H](O)[C@H]1O |
| 2911 | CCCCCCO[C@@H]1O[C@H](CO)[C@@H](O)[C@H](O)[C@H]1O[C@@H]2O[C@H](CO)[C@@H](O)[C@H](O)[C@H]2O |
| 2912 | CCCCCCO[C@@H]1O[C@H](CO[C@@H]2O[C@H](CO)[C@@H](O)[C@H](O)[C@H]2O)[C@@H](O)[C@H](O)[C@H]1O |
| 2913 | OCc1ccc(O)cn1 |
| 2914 | OCc1ccc([O-])cn1 |
| 2915 | CCCCCC\C=C\C=C\CCCCCCCC(=O)[O-] |
| 2916 | CCCCCCC\C=C\CCCCCCCCC(=O)[O-] |
| 2917 | OC[C@H]1O[C@H](O)[C@@H](O)[C@@H](O)[C@@H]1O |
| 2918 | COc1oc(C=O)cc1 |
| 2919 | COCc1oc(C=O)cc1 |
| 2920 | COc1ccccc1Oc2ccccc2O |
| 2921 | CCC(=O)OC\C=C(\C)/CCC=C(C)C |
| 2922 | C[N@@H+]1CCC[C@H]1c2cccnc2 |
| 2923 | CN1CCC[C@H]1c2cccnc2 |
| 2924 | CCCC\C=C\C=C\C(=O)[O-] |
| 2925 | OC[C@H]1O[C@@](O)(CN2[C@@H](CCC2=O)C(=O)[O-])[C@@H](O)[C@@H]1O |
| 2926 | C[C@@H]1O[C@@H](O)[C@H](O)[C@H](O)[C@H]1O |
| 2927 | OC[C@H]1O[C@@H](O)[C@H](O)[C@@H]1O |
| 2928 | CC[C@H](\C=C\[C@@H](C)[C@H]1CC[C@H]2[C@@H]3CC=C4CC(=O)CC[C@]4(C)[C@@H]3CC[C@]12C)C(C)C |
| 2929 | CC[C@H](\C=C\[C@@H](C)[C@H]1CC[C@@H]2C3=CC[C@H]4CC(=O)CC[C@]4(C)[C@H]3CC[C@]12C)C(C)C |
| 2930 | CC[C@H](CC[C@@H](C)[C@H]1CC[C@H]2C3=CC[C@H]4C[C@H](CC[C@]4(C)[C@H]3CC[C@]12C)O[C@@H]5O[C@H](CO)[C@@H](O)[C@H](O)[C@H]5O)C(C)C |
| 2931 | CC[C@H](CC[C@@H](C)[C@H]1CC[C@@H]2C3=CC[C@H]4CC(=O)CC[C@]4(C)[C@H]3CC[C@]12C)C(C)C |
| 2932 | COc1cc(C=O)cc(OC)c1O |
| 2933 | COc1cc(C=O)cc(OC)c1[O-] |
| 2934 | COc1cc(\C=C\CO)cc(OC)c1O[C@@H]2O[C@H](CO)[C@@H](O)[C@H](O)[C@H]2O |
| 2935 | COc1cc(\C=C\COC(=O)C[C@@](C)(CC(=O)[O-])O[C@@H]2O[C@H](CO)[C@@H](O)[C@H](O)[C@H]2O)cc(OC)c1O[C@@H]3O[C@H](CO)[C@@H](O)[C@H](O)[C@H]3O |
| 2936 | COc1cc(cc(OC)c1O[C@@H]2O[C@H](CO)[C@@H](O)[C@H](O)[C@H]2O)[C@@H](O)C=C |
| 2937 | COc1cc(\C=C\Cc2c(OC)c(O[C@@H]3O[C@H](CO)[C@@H](O)[C@H](O)[C@H]3O)c(OC)cc2\C=C\CO)cc(OC)c1O[C@@H]4O[C@H](CO)[C@@H](O)[C@H](O)[C@H]4O |
| 2938 | COc1cc(\C=C\Cc2c(OC)c(O[C@@H]3O[C@H](CO)[C@@H](O)[C@H](O)[C@H]3O)c(OC)cc2\C=C\COC(=O)C[C@](C)(CC(=O)[O-])O[C@@H]4O[C@H](CO)[C@@H](O)[C@H](O)[C@H]4O)cc(OC)c1O[C@@H]5O[C@H](CO)[C@@H](O)[C@H](O)[C@H]5O |
| 2939 | CC1(C)CC[C@]2(C)CC=C3[C@]4(C)CC[C@H]5C(C)(C)[C@@H](O)CC[C@]5(C)[C@H]4CC[C@@]3(C)[C@@H]2C1 |
| 2940 | CC(=O)O[C@H]1CC[C@@]2(C)[C@@H](CC[C@]3(C)[C@@H]2CC[C@@]4(C)[C@@H]5CC(C)(C)CC[C@]5(C)CC=C34)C1(C)C |
| 2941 | O[C@@H]1CO[C@H](O)[C@H](O)[C@H]1O |
| 2942 | COc1cc(cc(OC)c1O)[C@H]2OC[C@H]3[C@@H]2CO[C@H]3c4cc(OC)c(O)c(OC)c4 |
| 2943 | CC[C@H](C)CCCCCCCCCCCCC(=O)[O-] |
| 2944 | CCO[C@@]1(CO)O[C@H](CO)[C@@H](O)[C@@H]1O |
| 2945 | C[C@@H](CCC=C(C)C)c1ccc(C)cc1 |
| 2946 | C\C\1=C/Cc2c(C)coc2C\C(=C\CC1)\C |
| 2947 | O[C@H]1CO[C@H](OC2=C(Oc3cc(O)cc(O)c3C2=O)c4ccc(O)c(O)c4)[C@H](O)[C@H]1O |
| 2948 | CO[C@H]1C\C(=C\Cc2c(C)coc2C\C(=C\1)\C)\C |
| 2949 | CCCCCCCCCCCCCC=C |
| 2950 | O[C@@H]([C@@H](O)C(=O)[O-])C(=O)[O-] |
| 2951 | C[C@H](CCCC(=C)C)C1=CCC(=CC1)C |
| 2952 | CCCCO |
| 2953 | CC[C@H](\C=C\[C@@H](C)[C@H]1CC[C@H]2[C@H]3CC=C4C[C@H](CC[C@]4(C)[C@@H]3CC[C@]12C)O[C@H]5O[C@H](CO)[C@@H](O)[C@H](O)[C@H]5O)C(C)C |
| 2954 | CC[C@H](\C=C\[C@@H](C)[C@H]1CC[C@H]2[C@H]3CC=C4C[C@@H](O)CC[C@]4(C)[C@@H]3CC[C@]12C)C(C)C |
| 2955 | CC1(C)CC[C@]2(C)CC=C3[C@]4(C)CC[C@H]5C(C)(C)C(=O)CC[C@]5(C)[C@H]4CC[C@@]3(C)[C@@H]2C1 |
| 2956 | COc1c(O)c2C(=O)C(=COc2cc1O[C@@H]3O[C@H](CO)[C@@H](O)[C@H](O)[C@H]3O)c4ccc(O)cc4 |
| 2957 | COc1c(O)c2C(=O)C(=COc2cc1O[C@@H]3O[C@H](CO)[C@@H](O)[C@H](O)[C@H]3O)c4ccc([O-])cc4 |
| 2958 | COc1c(O)cc2OC=C(C(=O)c2c1O)c3ccc(O)cc3 |
| 2959 | COc1c(O)cc2OC=C(C(=O)c2c1O)c3ccc([O-])cc3 |
| 2960 | COc1ccc2C(=O)C(=C(C)Oc2c1)c3ccccc3 |
| 2961 | C\C(=C\C=C\C=C(\C)/C=C\C=C(/C)\[C@@H]1O[C@]2(C)C[C@H](O)CC(C)(C)C2=C1)\C=C\C=C(/C)\C=C\[C@@H]3C(=C[C@@H](O)CC3(C)C)C |
| 2962 | COc1c(O)c2C(=O)C=C(Oc2cc1O[C@H]3O[C@@H](C)[C@H](O)[C@@H](O)[C@H]3O)c4ccc(O)c(O)c4 |
| 2963 | O=C1Oc2ccccc2C3=C1C(=O)c4ccccc4O3 |
| 2964 | CC[C@H](CC[C@@H](C)[C@H]1CC[C@H]2C3=CC[C@@H]4C[C@@H](O)CC[C@]4(C)[C@H]3CC[C@]12C)C(C)C |
| 2965 | OCCCCCCCCCCCCCCCCCCCCCCO |
| 2966 | COC1=C(C#N)C(=O)N(C)C=C1 |
| 2967 | CCCC(=O)[O-] |
| 2968 | [O-]C(=O)[C@@H]1[NH2+]CCc2c1[nH]c3ccccc23 |
| 2969 | [O-]C(=O)[C@@H]1NCCc2c1[nH]c3ccccc23 |
| 2970 | CC[C@H](C)CCCCCCCCCCCC(=O)[O-] |
| 2971 | OC[C@H]1O[C@@H](Oc2cc([O-])cc3OC(=CC(=O)c23)c4ccc(O)c(O)c4)[C@H](O)[C@@H](O)[C@@H]1O |
| 2972 | COc1cc(\C=C/COC(=O)C[C@](C)(O)CC(=O)[O-])cc(OC)c1O |
| 2973 | COc1cc(\C=C/Cc2c(OC)c(O[C@@H]3O[C@@H](CO)[C@@H](O)[C@@H](O)[C@H]3O)c(OC)cc2\C=C\CO)cc(OC)c1O[C@H]4O[C@H](CO)[C@@H](O)[C@@H](O)[C@@H]4O |
| 2974 | COc1cc(\C=C/Cc2c(OC)c(O)c(OC)cc2\C=C/CO)cc(OC)c1O |
| 2975 | COc1cc(\C=C\Cc2c(OC)c(O[C@@H]3O[C@H](CO)[C@@H](O)[C@H](O)[C@H]3O)c(OC)cc2\C=C\COC(=O)C[C@](C)(CC(=O)[O-])O[C@H]4O[C@@H](CO)[C@H](O)[C@@H](O)[C@@H]4O)cc(OC)c1O[C@H]5O[C@@H](CO)[C@H](O)[C@@H](O)[C@@H]5O |
| 2976 | COc1cc(\C=C\Cc2c(OC)c(O)c(OC)cc2\C=C\COC(=O)C[C@@](C)(O)CC(=O)[O-])cc(OC)c1O |
| 2977 | CC(C)(C)c1ccccc1 |
| 2978 | CC\C=C\CC\C=C\CO |
| 2979 | Cc1ccc2[nH]c3ccccc3c2c1 |
| 2980 | CS(=O)CCCC[NH+]=C=S |
| 2981 | OC12CCC(CC1)(CC2)c3ccccc3 |
| 2982 | COc1cc(O)c(Cc2ccc(O)cc2)c(\C=C\c3ccccc3)c1Cc4ccc(O)cc4 |
| 2983 | CC[C@@H](CC[C@@H](C)[C@H]1CC[C@@H]2[C@@H]3CC(=O)[C@H]4CC(=O)CC[C@]4(C)[C@H]3CC[C@]12C)C(C)C |
| 2984 | COc1c(O)c(OC)c2c(O)c(c(C)cc2c1C(C)C)c3c(C)cc4c(C(C)C)c(OC)c(O)c(OC)c4c3O |
| 2985 | CCCCC1CCCCC1 |
| 2986 | COc1c(O)c2COc3cc(O)cc4CCc(c1OC)c2c34 |
| 2987 | COc1c(O)c2COc3cc([O-])cc4CCc(c1OC)c2c34 |
| 2988 | C[C@]12CC[C@H]3[C@@H](CC=C4CC=CC(=O)[C@]34C)[C@H]1CC[C@@H]2[C@H]5CO[C@@]6(C)C[C@@H]5OC(=O)C6=C |
| 2989 | CCOC(=O)c1cc(O)c(OC(=O)c2cc(O)c(O)c([O-])c2)c([O-])c1 |
| 2990 | COc1cc2C(=O)C(=COc2cc1O)c3ccc(O)cc3 |
| 2991 | COc1cc2C(=O)C(=COc2cc1[O-])c3ccc(O)cc3 |
| 2992 | COc1cc2C(=O)C(=COc2cc1[O-])c3ccc([O-])cc3 |
| 2993 | CS(=O)CC[C@H]([NH3+])C(=O)[O-] |
| 2994 | CC(=O)O[C@@H]1CC(=C)[C@@H](CC[C@@]2(C)O[C@@H]2[C@H]3OC(=O)C(=C)[C@H]13)OO |
| 2995 | CC(=O)O[C@@H]1[C@@H](O)[C@H](CO[C@H]1O[C@H]2C[C@@]3(C)[C@@H]4CC=C5[C@@H](C=C([O-])C(=O)C5(C)C)[C@]4(C)CC[C@]3(C)[C@H]2[C@@](C)(O)C(=O)\C=C\C(C)(C)O)OC(=C)O |
| 2996 | CC[C@H](\C=C\[C@@H](C)[C@H]1CC[C@H]2[C@@H]3CC=C4CC(=O)CC[C@]4(C)[C@H]3CC[C@]12C)C(C)C |
| 2997 | CC[C@@H](\C=C/[C@H](C)[C@H]1CC[C@@H]2[C@@H]3CC=C4C[C@H](CC[C@]4(C)[C@H]3CC[C@]12C)OC(=O)\C=C\c5ccc(O)c(OC)c5)C(C)C |
| 2998 | COc1cc(cc(OC)c1O)[C@H](O)C=C |
| 2999 | CON1C(=O)[C@@]2(C[C@H]3[NH2+]C\C(=C\C)\[C@@H]4C[C@H]2OC[C@H]34)c5ccc(O)cc15 |
| 3000 | CC[C@@H](\C=C\[C@H](C)[C@H]1CC[C@H]2C3=CC[C@H]4CC(=O)CC[C@]4(C)[C@H]3CC[C@]12C)C(C)C |
| 3001 | CC[C@@H](\C=C/[C@H](C)[C@H]1CC[C@@H]2[C@@H]3CC[C@H]4C[C@@H](O)CC[C@]4(C)[C@H]3CC[C@]12C)C(C)C |
| 3002 | CC[C@@H](CC[C@H](C)[C@H]1CC[C@H]2C3=CC[C@H]4CC(=O)CC[C@]4(C)[C@H]3CC[C@]12C)C(C)C |
| 3003 | CC[C@H](CC[C@@H](C)[C@H]1CC[C@@H]2[C@@H]3CC[C@H]4C[C@@H](CC[C@]4(C)[C@H]3CC[C@]12C)OC[C@@H]5O[C@H](O)[C@@H](O)[C@H](O)[C@H]5O)C(C)C |
| 3004 | CCOC[C@@H]1O[C@](COCC)(OCC)[C@@H](OCC)[C@H]1OCC |
| 3005 | CC[C@H](CC[C@@H](C)[C@H]1CC[C@@H]2[C@H]3CC=C4C[C@@H](CC[C@]4(C)[C@@H]3CC[C@]12C)O[C@@H]5O[C@H](CO)[C@@H](O)[C@H](O)[C@H]5O)C(C)C |
| 3006 | CC[C@H](CC[C@@H](C)[C@H]1CC[C@@H]2[C@H]3CC=C4C[C@H](O)CC[C@]4(C)[C@@H]3CC[C@]12C)C(C)C |
| 3007 | C[N+](C)(C)CC(=O)[O-] |
| 3008 | [O-]C(=O)\C=C/c1ccccc1 |
| 3009 | C[NH+]1[C@@H]2CC[C@H]1CC(C2)OC(=O)[C@H](CO)c3ccccc3 |
| 3010 | CCCCC\C=C/C\C=C\CCCCCCCC(=O)[O-] |
| 3011 | CC\C=C/C\C=C/C=C/[C@@H](O)CCCCCCCC(=O)[O-] |
| 3012 | O[C@@H]1CC[C@@]2(O)[C@@H](O)[C@H](O)[C@@H]([C@@H]12)[N+](=O)[O-] |
| 3013 | COc1cc2C=CC(=O)Oc2cc1O[C@H]3O[C@H](CO)[C@@H](O)[C@@H](O)[C@@H]3O |
| 3014 | C[N@H+]1[C@@H]2CC[C@@]1(CO)C[C@H](C2)c3cccc(CC(=O)[O-])c3 |
| 3015 | CCCCC\C=C/C=C/[C@H](O)CCCCCCCC(=O)[O-] |
| 3016 | Oc1ccc(CCC(=O)NCCC[NH2+]CCCC[NH2+]CCCNC(=O)CCc2ccc(O)c(O)c2)cc1O |
| 3017 | [NH3+]CCS(=O)(=O)[O-] |
| 3018 | NCCS(=O)(=O)[O-] |
| 3019 | O=C(CNC(=O)[C@H](Cc1ccccc1)NC(=O)c2ccccc2)OCCCc3ccccc3 |
| 3020 | OC\C=C\c1ccccc1 |
| 3021 | CC(C)[C@H]1NC(=O)[C@H](NC(=O)[C@@H](Cc2ccc(O)cc2)NC(=O)[C@@H]3CCCN3C(=O)[C@@H]4CCC(=O)N4)n5cc(C[C@@H](NC(=O)[C@H](CO)NC(=O)CNC1=O)C(=O)[O-])c6ccccc56 |
| 3022 | CC(C)[C@H]1NC(=O)[C@H](NC(=O)[C@H](Cc2c[nH]c3ccccc23)NC(=O)[C@@H]4CCCN4C(=O)[C@@H]5CCC(=O)N5)n6cc(C[C@@H](NC(=O)[C@H](CO)NC(=O)CNC1=O)C(=O)[O-])c7ccccc67 |
| 3023 | CC(=CCC[C@@H]1[C@@H]2[C@H](C[C@]3(C)C4=CC[C@@H]5C(C)(C)C(=O)CC[C@]5(C)[C@@H]4CC[C@@]23C)OC1=O)C |
| 3024 | CCCCCCCCCCCC(=O)OCC |
| 3025 | CC(C)c1cccc(C)c1 |
| 3026 | CCCCCCCCCC |
| 3027 | CC1=CC[C@H]2[C@@H](C1)C2(C)C |
| 3028 | CCCCCCC[C@H](C)O |
| 3029 | CC(=CCCC(=C)[C@@H]1CCC(=CC1)C)C |
| 3030 | Cc1cccc(O)c1 |
| 3031 | Cc1ccccc1C |
| 3032 | CC1=CC[C@H](CC=O)C1(C)C |
| 3033 | C[C@@H]1CC[C@]23C[C@H]1C(C)(C)[C@@H]2CC[C@@]3(C)OC(=O)C |
| 3034 | CC(C)(O[C@@H]1O[C@H](CO)[C@@H](O)[C@H](O)[C@H]1O)[C@@H]2Cc3cc4C=CC(=O)Oc4cc3O2 |
| 3035 | C\C=C(\C)/C(=O)OC(C)(C)[C@@H]1Cc2c(O1)ccc3C=CC(=O)Oc23 |
| 3036 | CC(=O)OC(C)(C)[C@@H]1Cc2c(O1)ccc3C=CC(=O)Oc23 |
| 3037 | CC(C)(O)[C@@H]1Cc2c(O1)ccc3C=CC(=O)Oc23 |
| 3038 | COc1cc(C)cc(OC)c1 |
| 3039 | CCCCCCCCC=C |
| 3040 | CC1=CCC=CC1 |
| 3041 | CCCCCCCCCCCCCCCCCCCl |
| 3042 | Cc1cc(C)c(C)c(O)c1C |
| 3043 | CCC\C=C\C |
| 3044 | Cc1ccccc1C(C)(C)C |
| 3045 | CCCCCCCC[C@@H](C)C1CCCCC1 |
| 3046 | CCCCCCC(C)C |
| 3047 | CCc1cccc(C)c1O |
| 3048 | CCCCCC[C@@H](C)CC |
| 3049 | O=C1C(=C1c2ccccc2)c3ccccc3 |
| 3050 | COc1cc2occc2c3OC(=O)C=Cc13 |
| 3051 | CC(=CCC[C@@]1(C)[C@H]2CC=C(C)[C@@H]1C2)C |
| 3052 | COc1cc2OC(=O)C=Cc2cc1[C@@H](OC(=O)\C(=C\C)\C)[C@@H](O)C(C)(C)O |
| 3053 | COc1cc2OC(=O)C=Cc2cc1[C@@H](OC(=O)\C(=C/C)\C)[C@@H](O)C(C)(C)O |
| 3054 | C\C=C(\C)/C(=O)[O-] |
| 3055 | COc1c(O)cc2C(=O)O[C@@H]3[C@@H](O)[C@H](O)[C@@H](CO)C[C@H]3c2c1O |
| 3056 | COc1cc2OC(=O)C=Cc2cc1[C@@H](OC(=O)CC(C)C)[C@@H](O)C(C)(C)O |
| 3057 | CCCCCCCCC |
| 3058 | CC(C)[C@@H]1CCC(=C[C@H]1c2cc(CC=C)cc(c2O)c3cc(CC=C)cc([C@@H]4C=C(C)CC[C@H]4C(C)C)c3O)C |
| 3059 | CC(C)[C@]1(C)CCC(=CC1)C |
| 3060 | CCCCCCCC=C |
| 3061 | CC(C)CC(=O)O[C@@H]1C[C@H]2CC[C@]1(C)C2(C)C |
| 3062 | CCCCCCCCCC=C |
| 3063 | C1Cc2cccc3cccc1c23 |
| 3064 | COc1cc(cc(OC)c1O)[C@H]2OC[C@H]3[C@@H]2CO[C@@H]3c4cc(OC)c(O[C@@H]5O[C@H](CO)[C@@H](O)[C@H](O)[C@H]5O)c(OC)c4 |
| 3065 | CC(=O)O[C@@]1(C)C[C@@H](O)[C@@H]2C=CO[C@@H](O[C@@H]3O[C@H](CO)[C@@H](O)[C@H](O)[C@H]3O)[C@]12C |
| 3066 | COc1cc(ccc1O[C@@H]2O[C@H](CO)[C@@H](O)[C@H](O)[C@H]2O)[C@@H](O)[C@H](CO)Oc3c(OC)cc(\C=C\CO)cc3OC |
| 3067 | COc1cc(\C=C\CO)ccc1O[C@@H]2O[C@H](CO)[C@@H](O)[C@H](O)[C@H]2O |
| 3068 | COc1cc2C[C@@](O)(CO)[C@H](CO)[C@@H](c3ccc(O)c(OC)c3)c2cc1O |
| 3069 | COc1cc(ccc1O)[C@@H]2[C@@H](CO)[C@](O)(CO)Cc3cc(OC)c([O-])cc23 |
| 3070 | COc1ccc(cc1O[C@@H]2O[C@H](CO)[C@@H](O)[C@H](O)[C@H]2O)[C@H]3Oc4c(OC)cc(\C=C\CO[C@@H]5O[C@H](CO)[C@@H](O)[C@H](O)[C@H]5O)cc4[C@@H]3CO |
| 3071 | OCC[C@H]1CCC(=C1CO)CO |
| 3072 | COc1cc(ccc1O)[C@@H]2Oc3c(OC)cc(cc3[C@@H]2CO)[C@@H](O)[C@H](O)CO |
| 3073 | COc1cc(ccc1[O-])[C@@H]2Oc3c(OC)cc(cc3[C@@H]2CO)[C@@H](O)[C@H](O)CO |
| 3074 | COc1cc(ccc1O)[C@H]2Oc3c(OC)cc(cc3[C@@H]2CO)[C@@H](O)[C@H](O)CO |
| 3075 | COc1cc(ccc1[O-])[C@H]2Oc3c(OC)cc(cc3[C@@H]2CO)[C@@H](O)[C@H](O)CO |
| 3076 | OC[C@@H](O)[C@H](O)[C@H](O)[C@@H](O)CO |
| 3077 | COc1cc(ccc1O)[C@H]2OC[C@H]3[C@@H]2CO[C@H]3c4ccc(O)c(OC)c4 |
| 3078 | COc1cc(ccc1O)[C@H]2OC[C@H]3[C@@H]2CO[C@@H]3c4cc(OC)c(O[C@@H]5O[C@H](CO)[C@@H](O)[C@H](O)[C@H]5O)c(OC)c4 |
| 3079 | OCC[C@H]1[C@H](O)CC(=C1CO)CO |
| 3080 | OC[C@H]1O[C@@H](OCC[C@H]2[C@H](O)CC(=C2CO)CO)[C@H](O)[C@@H](O)[C@@H]1O |
| 3081 | OCC[C@H]1[C@@H](CC(=C1CO)CO)O[C@@H]2O[C@H](CO)[C@@H](O)[C@H](O)[C@H]2O |
| 3082 | COC(=O)C1=CO[C@@H](O)[C@H]2[C@@H]1CC=C2CO |
| 3083 | COc1cc(ccc1O)[C@@H](O)[C@@H](O)CO |
| 3084 | COc1cc(ccc1O)[C@H](O)[C@@H](O)CO |
| 3085 | COc1cc(ccc1O[C@@H]2O[C@H](CO)[C@@H](O)[C@H](O)[C@H]2O)[C@H](O)[C@@H](CO)Oc3c(OC)cc(cc3OC)[C@H]4OC[C@H]5[C@@H]4CO[C@@H]5c6ccc(O[C@@H]7O[C@H](CO)[C@@H](O)[C@H](O)[C@H]7O)c(OC)c6 |
| 3086 | [NH3+]CCc1cnc[nH]1 |
| 3087 | OC1CCC(CC1)C(=O)[O-] |
| 3088 | COc1cc(ccc1O[C@@H]2O[C@H](CO)[C@@H](O)[C@H](O)[C@H]2O)[C@H]3OC[C@@]4(O)[C@@H]3CO[C@@H]4c5ccc(O[C@@H]6O[C@H](CO)[C@@H](O)[C@H](O)[C@H]6O)c(OC)c5 |
| 3089 | COc1cc(ccc1O)[C@H]2OC[C@@]3(O)[C@@H]2CO[C@@H]3c4ccc(O[C@@H]5O[C@H](CO)[C@@H](O)[C@H](O)[C@H]5O)c(OC)c4 |
| 3090 | COc1cc(ccc1O)[C@H]2OC[C@@H]3[C@H](OC[C@]23O)c4ccc(O[C@@H]5O[C@H](CO)[C@@H](O)[C@H](O)[C@H]5O)c(OC)c4 |
| 3091 | OC[C@H]1O[C@H](OC[C@H]2O[C@@H](O)[C@H](O)[C@@H](O)[C@@H]2O)[C@H](O)[C@@H](O)[C@@H]1O |
| 3092 | COc1cc(O[C@@H]2O[C@H](CO)[C@@H](O)[C@H](O)[C@H]2O)cc(OC)c1O |
| 3093 | COc1cc(cc(OC)c1O[C@@H]2O[C@H](CO)[C@@H](O)[C@H](O)[C@H]2O)[C@H]3OC[C@H]4[C@@H]3CO[C@@H]4c5cc(OC)c(O[C@@H]6O[C@H](CO)[C@@H](O)[C@H](O)[C@H]6O)c(OC)c5 |
| 3094 | COc1cc(ccc1O)[C@H]2OC[C@H]3[C@@H]2CO[C@@H]3c4cc(OC)c(O)c(OC)c4 |
| 3095 | COc1cc(ccc1O[C@@H]2O[C@H](CO)[C@@H](O)[C@H](O)[C@H]2O)[C@H]3OC[C@H]4[C@@H]3CO[C@@H]4c5cc(OC)c(O[C@@H]6O[C@H](CO)[C@@H](O)[C@H](O)[C@H]6O)c(OC)c5 |
| 3096 | COC(=O)[C@]1(O)C[C@@H](O)[C@@H](O)[C@@H](C1)OC(=O)\C=C\c2ccc(O)c(O)c2 |
| 3097 | CCCCCCCCCCCCCCCCCCCCCCCCCCCCC |
| 3098 | COc1cc(C[C@@]2(O)CO[C@@H]([C@H]2CO)c3ccc(O)c(OC)c3)ccc1O |
| 3099 | COc1cc(C[C@@]2(O)CO[C@@H]([C@H]2CO)c3ccc(O[C@@H]4O[C@H](CO)[C@@H](O)[C@H](O)[C@H]4O)c(OC)c3)ccc1O[C@@H]5O[C@H](CO)[C@@H](O)[C@H](O)[C@H]5O |
| 3100 | COc1cc(ccc1O)[C@H]2OC[C@](O)(Cc3ccc(O[C@@H]4O[C@H](CO)[C@@H](O)[C@H](O)[C@H]4O)c(OC)c3)[C@@H]2CO |
| 3101 | COc1cc(C[C@@]2(O)CO[C@@H]([C@H]2CO)c3ccc(O[C@@H]4O[C@H](CO)[C@@H](O)[C@H](O)[C@H]4O)c(OC)c3)ccc1O |
| 3102 | COc1cc(ccc1O)[C@H]2OC[C@H]3[C@@H]2CO[C@@H]3c4ccc(O)c(OC)c4 |
| 3103 | COc1cc(ccc1O[C@@H]2O[C@H](CO)[C@@H](O)[C@H](O)[C@H]2O)[C@H]3OC[C@H]4[C@@H]3CO[C@@H]4c5ccc(O[C@@H]6O[C@H](CO)[C@@H](O)[C@H](O)[C@H]6O)c(OC)c5 |
| 3104 | COc1cc(ccc1O)[C@H]2OC[C@H]3[C@@H]2CO[C@@H]3c4ccc(O[C@@H]5O[C@H](CO)[C@@H](O)[C@H](O)[C@H]5O)c(OC)c4 |
| 3105 | CC(=O)O[C@@]1(C)CC[C@]2(O)C=CO[C@@H](O[C@@H]3O[C@H](CO)[C@@H](O)[C@H](O)[C@H]3O)[C@H]12 |
| 3106 | COc1cc(cc(OC)c1O[C@@H]2O[C@H](CO)[C@@H](O)[C@H](O)[C@H]2O)[C@@H](O)[C@H](CO)Oc3c(OC)cc(cc3OC)[C@H]4OC[C@H]5[C@@H]4CO[C@@H]5c6cc(OC)c(O[C@@H]7O[C@H](CO)[C@@H](O)[C@H](O)[C@H]7O)c(OC)c6 |
| 3107 | CCCCCCCCCCCCCCCCCCCCCCCCCCCCCCO |
| 3108 | CC(=CCC\C(=C\CC\C(=C/CC\C(=C/CC\C(=C\C[C@@H](CO)C(=C)C)\C)\C)\C)\C)C |
| 3109 | OC[C@H]1O[C@@H](OC[C@H]2O[C@@H](O[C@@H]3OC=C[C@H]4[C@H](O)C=C(CO)[C@@H]34)[C@H](O)[C@@H](O)[C@@H]2O)[C@H](O)[C@@H](O)[C@@H]1O |
| 3110 | O[C@H]1Cc2c(O)cc(O)cc2O[C@H]1c3ccc(O)c(O)c3 |
| 3111 | O[C@H]1Cc2c(O)cc([O-])cc2O[C@H]1c3ccc(O)c(O)c3 |
| 3112 | COc1cc(cc(OC)c1O)[C@H]2OC[C@H]3[C@@H]2CO[C@@H]3c4cc(OC)c(O)c(OC)c4 |
| 3113 | CO[C@@H]1C[C@]23[N@H+](CCc4cc5OCOc5cc24)CC=C3C=C1 |
| 3114 | Oc1ccc(cc1)C2=COc3cc([O-])cc(O)c3C2=O |
| 3115 | Oc1cc([O-])cc2OC=C(C(=O)c12)c3ccc([O-])cc3 |
| 3116 | CCCCCCCCCCCCCCCCCCCCCCCCCCCC(=O)[O-] |
| 3117 | COc1cc(O)cc(OC)c1O |
| 3118 | COc1cc(ccc1O)[C@@H]2Oc3c(OC)cc(CCCO)cc3[C@H]2CO |
| 3119 | COc1cc(ccc1[O-])[C@@H]2Oc3c(OC)cc(CCCO)cc3[C@H]2CO |
| 3120 | CC1(C)C[C@H](O)C[C@@]2(C)OC(=O)C=C12 |
| 3121 | CC(C)CCC[C@H](C)CCC[C@@H](C)CCC\C(=C\CO)\C |
| 3122 | [O-]C(=O)\C=C/C(=O)[O-] |
| 3123 | COc1cc(\C=C\C=O)ccc1O |
| 3124 | COc1cc(\C=C\CO)ccc1O |
| 3125 | Oc1ccc(CCC(=O)[O-])cc1O |
| 3126 | COC(=O)[C@@H]1CO[C@@H]2OC[C@]3(O)C=C[C@H]1[C@H]23 |
| 3127 | COc1cc(C[C@]2(O)CO[C@H]([C@@H]2CO)c3ccc(O)c(OC)c3)ccc1O |
| 3128 | CCC\C=C\C\C=C\CCCCCCCc1cccc(O)c1O |
| 3129 | C\C=C/C(=O)C1=C(C)C=CCC1(C)C |
| 3130 | CC1(C)[C@H]2CCC(=C[C@@H]12)C=O |
| 3131 | COc1ccc(\C=C/C(=O)[O-])cc1OC |
| 3132 | COc1cc(C[C@]2(O)CO[C@@H]([C@H]2CO)c3ccc(O)c(OC)c3)ccc1O |
| 3133 | OC[C@H]1O[C@H]([C@H](O)[C@@H](O)[C@@H]1O)c2c(O)cc(O)c3C(=O)c4cc(O)c(O)cc4Oc23 |
| 3134 | OC[C@H]1O[C@H]([C@H](O)[C@@H](O)[C@@H]1O)c2c(O)cc(O)c3C(=O)c4cc([O-])c(O)cc4Oc23 |
| 3135 | Oc1cccc(\C=C\C(=O)[O-])c1 |
| 3136 | CC1(C)[C@@H](O)CC[C@@]2(C)[C@H]1CC[C@]3(C)[C@@H]2CC=C4[C@@H]5C[C@](C)(CC[C@]5(C)CC[C@@]34C)C(=O)[O-] |
| 3137 | CCCCC[C@H](O)C[C@H](O)CCC |
| 3138 | CC(C)C(=O)C |
| 3139 | COc1cc(O)c2C(=O)C(=C(Oc2c1)c3ccc(OC)c(O)c3)[O-] |
| 3140 | OC[C@@H](O)[C@@H](O)[C@H](O)[C@@H](O)CO |
| 3141 | O[C@@H]1C[C@@](O)(C[C@@H](OC(=O)\C=C\c2ccc(O)c(O)c2)[C@H]1O)C(=O)[O-] |
| 3142 | COc1cc(ccc1O)[C@H]2OC[C@@]3(O)[C@@H]2CO[C@@H]3c4ccc(O)c(OC)c4 |
| 3143 | COc1cc(cc(OC)c1OC)[C@H]2OC[C@H]3[C@@H]2CO[C@@H]3c4cc(OC)c(OC)c(OC)c4 |
| 3144 | CC(=O)O[C@@]1(C)C[C@@H](O)[C@]2(O)C=CO[C@@H](O[C@@H]3O[C@H](CO)[C@@H](O)[C@H](O)[C@H]3O)[C@H]12 |
| 3145 | CC(=O)O[C@@]1(C)C[C@@H](O)[C@]2(O)C=CO[C@@H](O)[C@H]12 |
| 3146 | CC(=O)O[C@@]1(C)CC[C@]2(O)C=CO[C@@H](O[C@@H]3O[C@H](CO)[C@@H](O)[C@H](O)[C@H]3O)[C@H]12 |
| 3147 | CC(=O)O[C@@]1(C)CC[C@]2(O)C=CO[C@@H](O)[C@H]12 |
| 3148 | COc1cc(ccc1O)[C@@H](O)[C@@H](CO)Oc2ccc(\C=C\C=O)cc2OC |
| 3149 | CCCCCCCCCCCCCCC[C@H](O)CCCCCCCCCCCCCC |
| 3150 | COc1cc(ccc1O[C@@H]2O[C@H](CO)[C@@H](O)[C@H](O)[C@H]2O)[C@H]3OC[C@@]4(O)[C@@H]3CO[C@@H]4c5ccc(O[C@@H]6O[C@H](CO)[C@@H](O)[C@H](O)[C@H]6O)c(OC)c5 |
| 3151 | COc1cc(ccc1O[C@@H]2O[C@H](CO)[C@@H](O)[C@H](O)[C@H]2O)[C@@H]3OC[C@@H]4[C@H]3CO[C@H]4c5cc(OC)c(O[C@@H]6O[C@H](CO)[C@@H](O)[C@H](O)[C@H]6O)c(OC)c5 |
| 3152 | COc1cc(ccc1O)[C@@H]2OC[C@@H]3[C@H]2CO[C@H]3c4cc(OC)c(O)c(OC)c4 |
| 3153 | COc1cc(ccc1O[C@H]2O[C@H](CO)[C@H](O)[C@@H](O)[C@H]2O)[C@H]3OC[C@H]4[C@@H]3CO[C@@H]4c5ccc(O[C@H]6O[C@H](CO)[C@H](O)[C@@H](O)[C@H]6O)c(OC)c5 |
| 3154 | CC[C@@H]1C[N@H+](C)[C@H]2Cc3c([nH]c4ccccc34)C(=O)C[C@@H]1[C@H]2C(=O)OC |
| 3155 | CC[C@@H]1CN(C)[C@H]2Cc3c([nH]c4ccccc34)C(=O)C[C@@H]1[C@H]2C(=O)OC |
| 3156 | CC\C(=C/c1occc1)\C=O |
| 3157 | Oc1cccc(CCC(=O)[O-])c1 |
| 3158 | O[C@@H]1CC[NH2+][C@H]1C(=O)[O-] |
| 3159 | CC(=O)O[C@@]1(C)C[C@H](O)[C@@H]2C=CO[C@@H](O[C@@H]3O[C@H](CO)[C@@H](O)[C@H](O)[C@H]3O)[C@H]12 |
| 3160 | CC(=O)O[C@@]1(C)C[C@H](O)[C@@H]2C=CO[C@@H](O)[C@H]12 |
| 3161 | OCC1=C[C@H](O)[C@H]2C=CO[C@H](O)[C@@H]12 |
| 3162 | C[C@@H]1CC[C@@]2(OC1)O[C@H]3C[C@@H]4[C@@H]5C[C@H](O)[C@H]6C[C@@H](O)CC[C@]6(C)[C@H]5CC[C@]4(C)[C@H]3[C@@H]2C |
| 3163 | N1\C(=C\2/C=Nc3ccccc23)\SC=C1c4c[nH]c5ccccc45 |
| 3164 | O=C1CCCCCCC\C=C/CCCCCCC1 |
| 3165 | C[C@@H]1C[NH2+][C@@H]2[C@@H](C1)O[C@]3(CC[C@H]4[C@@H]5CC=C6C[C@@H](O)CC[C@]6(C)[C@H]5CC4=C3C)[C@H]2C |
| 3166 | COc1ccc(cc1O)[C@H]2Oc3c(OC)cc(\C=C\CO)cc3[C@@H]2CO |
| 3167 | COc1cc(CC=C)cc(c1O)c2cc(CC=C)cc(OC)c2O |
| 3168 | COc1ccc2nccc([C@@H](O)[C@H]3C[C@@H]4CC[N@H+]3C[C@@H]4C=C)c2c1 |
| 3169 | COc1ccc2nccc([C@@H](O)[C@H]3C[C@@H]4CCN3C[C@@H]4C=C)c2c1 |
| 3170 | COc1cc(ccc1O)[C@H]2Oc3c(OC)cc(cc3[C@@H]2CO)[C@H](O)[C@@H](O)CO |
| 3171 | COc1cc(ccc1[O-])[C@H]2Oc3c(OC)cc(cc3[C@@H]2CO)[C@H](O)[C@@H](O)CO |
| 3172 | [NH3+][C@H]([C@H](O)C(=O)[O-])C(=O)[O-] |
| 3173 | OC[C@H]1O[C@H](O[C@@H]2OC=C([C@H]3CC=C(CO)[C@@H]23)C(=O)[O-])[C@H](O)[C@@H](O)[C@H]1O |
| 3174 | COC(=O)C1=CO[C@@H](O[C@@H]2O[C@H](CO[C@@H]3O[C@H](CO)[C@@H](O)[C@H](O)[C@H]3O)[C@@H](O)[C@H](O)[C@H]2O)[C@H]4[C@@H]1CC=C4CO |
| 3175 | COc1ccccc1OC[C@@H](O)CO |
| 3176 | C[C@@]1(C[C@@H](O)[C@]2(O)C=CO[C@@H](O[C@@H]3O[C@H](CO)[C@@H](O)[C@H](O)[C@H]3O)[C@H]12)OC(=O)\C=C\c4ccccc4 |
| 3177 | C[C@@]1(C[C@@H](O)[C@]2(O)C=CO[C@@H](O)[C@H]12)OC(=O)\C=C\c3ccccc3 |
| 3178 | C[C@@H]1C[C@H]2OC(=O)C(=C)[C@H]2[C@H](O)[C@@]3(C)[C@H]1C=CC3=O |
| 3179 | CC1(C)C[C@H]2[C@H]1CC[C@@]3(C)O[C@@H]3CCC2=O |
| 3180 | CC[C@@H](C)C(=O)Oc1c(O)c(OC)c(O)c(C(=O)\C=C\c2ccccc2)c1OC |
| 3181 | CC[C@@H](C)C(=O)Oc1c([O-])c(OC)c(O)c(C(=O)\C=C\c2ccccc2)c1OC |
| 3182 | COc1ccc(cc1OC)[C@H]2OC[C@H]3[C@@H]2CO[C@@H]3c4ccc(OC)c(OC)c4 |
| 3183 | Oc1cc(O)cc(\C=C/c2ccccc2)c1 |
| 3184 | Oc1cc([O-])cc(\C=C/c2ccccc2)c1 |
| 3185 | CCCCC[C@@H]1OC(=O)C[C@@H]1O |
| 3186 | OC[C@H]1C[C@H](O)[C@H]2C=CO[C@@H](O[C@@H]3O[C@H](CO[C@H]4O[C@H](CO)[C@@H](O)[C@H](O)[C@H]4O)[C@@H](O)[C@H](O)[C@H]3O)[C@H]12 |
| 3187 | OC[C@H]1C[C@H](O)[C@H]2C=CO[C@@H](O)[C@H]12 |
| 3188 | NC(=O)NCCCC[C@H]([NH3+])C(=O)[O-] |
| 3189 | COc1cc(ccc1O)[C@H]2Oc3c(OC)cc(\C=C\CO)cc3[C@@H]2CO |
| 3190 | COc1cc(ccc1[O-])[C@H]2Oc3c(OC)cc(\C=C\CO)cc3[C@@H]2CO |
| 3191 | COc1cc(O[C@@H]2O[C@H](CO)[C@@H](O)[C@H](O)[C@@H]2O)c3cc(O[C@@H]4O[C@H](CO)[C@@H](O)[C@H](O)[C@@H]4O)c([o+]c3c1)c5cc(OC)c(O)c(OC)c5 |
| 3192 | COc1cc(O[C@@H]2O[C@H](CO)[C@@H](O)[C@H](O)[C@@H]2O)c3cc(O[C@@H]4O[C@H](CO)[C@@H](O)[C@H](O)[C@@H]4O)c([o+]c3c1)c5cc(OC)c([O-])c(OC)c5 |
| 3193 | COc1cc(O)c2cc(O)c([o+]c2c1)c3cc(OC)c(O)c(OC)c3 |
| 3194 | COc1cc(O)c2cc(O)c([o+]c2c1)c3cc(OC)c([O-])c(OC)c3 |
| 3195 | COc1cc(O[C@@H]2O[C@H](CO)[C@@H](O)[C@H](O)[C@H]2O)c([C@@H]3OC[C@@H]4[C@H]3CO[C@@H]4c5c(O[C@@H]6O[C@H](CO)[C@@H](O)[C@H](O)[C@H]6O)cc(OC)c(O[C@@H]7O[C@H](CO)[C@@H](O)[C@H](O)[C@H]7O)c5OC)c(OC)c1O[C@@H]8O[C@H](CO)[C@@H](O)[C@H](O)[C@H]8O |
| 3196 | COc1cc(O)c([C@H]2OC[C@@H]3[C@H]2CO[C@H]3c4c(O)cc(OC)c(O)c4OC)c(OC)c1O |
| 3197 | OC[C@H]1O[C@@H](O[C@H]2[C@H](O)[C@H](O)[C@@H](CO)O[C@H]2OC3=C(Oc4cc(O)cc(O)c4C3=O)c5ccc(O)c(O)c5)[C@H](O)[C@H](O)[C@@H]1O |
| 3198 | OC1CCC(CC1)C(=O)[O-] |
| 3199 | COc1cc(cc(OC)c1O)C2=C([O-])C(=O)c3c(O)cc(O)cc3O2 |
| 3200 | COc1cc(cc(OC)c1[O-])C2=C([O-])C(=O)c3c(O)cc(O)cc3O2 |
| 3201 | CC(=O)O[C@H]1Cc2c(O)c3C(=O)C=C(C)Oc3cc2OC1(C)C |
| 3202 | C[C@@](O)(CO)[C@@H]1Cc2c(O1)ccc3C=CC(=O)Oc23 |
| 3203 | C[C@@H]1C[C@]2(O[C@H]3C[C@@]4(C)C5=CC[C@H]6C(C)(C)[C@H](CC[C@@]67C[C@@]57C[C@@H](OC(=O)C)[C@]4(C)[C@@H]13)O[C@@H]8OC[C@@H](O)[C@H](O)[C@H]8O)O[C@@H](O)[C@]9(C)O[C@H]29 |
| 3204 | C\C=C(\C)/C(=O)OC(C)(C)[C@@H]1Cc2cc3C=CC(=O)Oc3cc2O1 |
| 3205 | COc1cc2C[C@]3(COc4cc(O)cc(O)c4C3=O)c2cc1OC |
| 3206 | COc1cc(O)c2C(=O)c3c(C)cc(O)cc3Oc2c1 |
| 3207 | COc1cc([O-])c2C(=O)c3c(C)cc(O)cc3Oc2c1 |
| 3208 | CC(=O)O[C@H]1Cc2c(O)c3C(=O)C=C(CO)Oc3cc2OC1(C)C |
| 3209 | COc1cc(CCO[C@@H]2O[C@H](COC(=O)\C=C\c3ccc(O)c(OC)c3)[C@@H](O)[C@H](O[C@@H]4O[C@@H](C)[C@H](O)[C@@H](O)[C@H]4O)[C@H]2O)ccc1O |
| 3210 | COc1c2C[C@H](Oc2cc3OC(=CC(=O)c13)C)C(C)(C)O[C@@H]4O[C@H](CO)[C@@H](O)[C@H](O)[C@H]4O |
| 3211 | CC1=CC(=O)c2c(O)c3C[C@H](O)C(C)(C)Oc3cc2O1 |
| 3212 | CCCCCCC[C@@H](O)\C=C\C#CC#C[C@@H](O)C=C |
| 3213 | CCCCCCCC[C@H](O)CC#CC#C[C@@H](O)C=C |
| 3214 | C\C=C(\C)/C(=O)OC(C)(C)[C@H]1Oc2cc3OC(=O)C=Cc3cc2[C@@H]1O |
| 3215 | COc1cc2OC[C@H](Cc3ccc(O)cc3)C(=O)c2c(O)c1OC |
| 3216 | COc1cc(O)c2C(=O)[C@@H](Cc3ccc(O)cc3)COc2c1 |
| 3217 | COc1cc(O)c2C(=O)[C@]3(COc2c1)Cc4cc(OC)c(OC)cc34 |
| 3218 | C\C=C(\C)/C(=O)O[C@H]1Cc2c(O)c3C(=O)C=C(CO)Oc3cc2OC1(C)C |
| 3219 | CC(C)(O)C=C |
| 3220 | COc1c2C[C@H](Oc2cc3OC(=CC(=O)c13)C)C(C)(C)O |
| 3221 | CCC[C@H](O)CCC=C |
| 3222 | C[C@@H]1O[C@@H](O[C@H]2[C@H](OC(=O)\C=C\c3ccc(O)c(O)c3)[C@@H](CO)O[C@@H]4OC[C@@H](O[C@@H]24)c5ccc(O)c(O)c5)[C@H](O)[C@H](O)[C@H]1O |
| 3223 | C\C\1=C/CCC2=C[C@@H](C\C(=C\[C@@H]3OC(=O)C(=C)[C@H]3CC1)\C)OC2=O |
| 3224 | C\C\1=C/[C@@H]2OC(=O)C(=C)[C@H]2CC[C@]3(C)CC[C@@H](O3)\C(=C\CC1)\C(=O)[O-] |
| 3225 | COc1c2C[C@H](Oc2cc3OC(=CC(=O)c13)CO[C@@H]4O[C@H](CO)[C@@H](O)[C@H](O)[C@H]4O)C(C)(C)O |
| 3226 | COc1ccc(C[C@]2(O)COc3cc(O)cc(O)c3C2=O)cc1 |
| 3227 | COc1ccc(C[C@]2(O)COc3cc(O)cc([O-])c3C2=O)cc1 |
| 3228 | Cc1cc(O)cc2Oc3cc(O)cc([O-])c3C(=O)c12 |
| 3229 | COc1cc(OC)c2C(=O)[C@@H](Cc3ccc(O)cc3)COc2c1OC |
| 3230 | CCCCC\C=C(\CCCC)/C=O |
| 3231 | C\C=C(/C)\C(=O)OC(C)(C)[C@@H]1Cc2cc3C=CC(=O)Oc3cc2O1 |
| 3232 | COc1cc(ccc1O)[C@H]2Oc3c(OC)cc4C=CC(=O)Oc4c3O[C@@H]2CO |
| 3233 | COc1cc(ccc1[O-])[C@H]2Oc3c(OC)cc4C=CC(=O)Oc4c3O[C@@H]2CO |
| 3234 | CCCCCCCCOC(=O)C |
| 3235 | [O-]C(=O)\C=C\C(=O)[O-] |
| 3236 | CCCCCCCCCCCCCCCCCCCCCCCCC(=O)[O-] |
| 3237 | CC1=CC(=O)[C@H]2C[C@@H]1C2(C)C |
| 3238 | CC1(C)[C@H]2CC=C(C=O)[C@@H]1C2 |
| 3239 | COC(=O)CCCCCCCCCCCCC(C)C |
| 3240 | C[C@]12CC[C@H](C=C1)C2(C)C |
| 3241 | CC(=O)O[C@H]1C(C)(C)[C@@H]2CC[C@@]1(C)C2 |
| 3242 | CCCCCC[C@H](O)C=C |
| 3243 | CC(C)CC(=O)OCc1ccccc1 |
| 3244 | CC(=CCCc1cocc1)C |
| 3245 | CCC(C)(C)C |
| 3246 | CCCCCCC\C=C/CC#CC#C[C@@H](O)C=C |
| 3247 | C\C(=C/CCC(=C)[C@H]1CCC(=CC1)C)\CO |
| 3248 | CCCCCCCCCCCC#C |
| 3249 | COc1cc(O)c2C(=O)C=C(Oc2c1)c3ccccc3 |
| 3250 | O=C1C[C@H](Oc2ccccc12)c3ccccc3 |
| 3251 | CCCCCCC\C=C\CO |
| 3252 | CCCCCCC[C@@H](O)\C=C\C#CC#C[C@@H](O)C=C |
| 3253 | CCCCCCC\C=C\CCCCCCCCC(=O)OC |
| 3254 | CC1(C)Oc2cc3OC(=CC(=O)c3c(O)c2C[C@H]1OOC[C@H](O)[C@@H](O)[C@H](O)[C@H](O)CO)CO |
| 3255 | CC1(C)Oc2cc3OC(=CC(=O)c3c(O)c2C[C@H]1O)CO |
| 3256 | C\C=C(/C)\C(=O)O[C@H]1[C@H](OC(=O)\C(=C\C)\C)C(C)(C)Oc2ccc3C=CC(=O)Oc3c12 |
| 3257 | COc1c2C[C@H](Oc2cc3OC(=CC(=O)c13)CO)C(C)(C)O |
| 3258 | C\C=C(\C)/C(=O)O[C@H]1Cc2c(O)c3C(=O)C=C(C)Oc3cc2OC1(C)C |
| 3259 | COc1c2C[C@H](Oc2cc3OC(=CC(=O)c13)C(=O)[O-])C(C)(C)O |
| 3260 | CCOC(=O)[C@H]1CCC(=O)[C@@H]1CC(=O)c2occc2 |
| 3261 | CCOC(=O)C1=CC(=C)c2c(O1)cc3O[C@@H](Cc3c2OC)C(C)(C)O |
| 3262 | CC(=O)O[C@@H]1Cc2c(O)c3C(=O)C=C(CO)Oc3cc2OC1(C)C |
| 3263 | OC[C@@H](O)[C@@H](O)C[C@H]1[C@@H]2[C@@H]3CCCN(C(=O)C3)C2=NC1=O |
| 3264 | COc1cc2C=CC(=O)Cc2c(O)c1OC |
| 3265 | CC1=CC(=O)c2c(O)c3C[C@@H](O)C(C)(C)Oc3cc2O1 |
| 3266 | COc1cc2C=CC(=O)Cc2c(OC)c1O |
| 3267 | COc1cc2C=CC(=O)Cc2c(OC)c1[O-] |
| 3268 | C\C=C(\C)/C(=O)O[C@@H]1Cc2c(O)c3C(=O)C=C(CO)Oc3cc2OC1(C)C |
| 3269 | CC(C)[C@H]1Cc2cc3C=CC(=O)Oc3cc2O1 |
| 3270 | COc1c2C[C@H](Oc2cc3OC(=CC(=O)c13)COOC[C@H](O)[C@@H](O)[C@H](O)[C@H](O)CO)C(C)(C)O |
| 3271 | CC1=CC(=O)c2c(O)c3C[C@@H](OOC[C@H](O)[C@@H](O)[C@H](O)[C@H](O)CO)C(C)(C)Oc3cc2O1 |
| 3272 | COc1c2OC(=O)C=Cc2c(O)c3ccoc13 |
| 3273 | CCC[C@@H](O)CCC=C |
| 3274 | CC(C)[C@H](CO)CC[C@H]([C@H]1[C@@H](C[C@@]2(C)C3=C(CC[C@]12C)[C@@]4(C)CC[C@H](OC(=O)C)C(C)(C)[C@@H]4CC3)OC(=O)C)C(=O)O |
| 3275 | CC(C)[C@H](CO)CC[C@H]([C@H]1[C@@H](C[C@@]2(C)C3=C(CC[C@]12C)[C@@]4(C)CC[C@H](OC(=O)C)C(C)(C)[C@@H]4CC3)OC(=O)C)C(=O)[O-] |
| 3276 | CC(=O)O[C@H]1CC[C@@]2(C)[C@@H](CC[C@]3(C)[C@@H]2CC=C4[C@@H]5CC(C)(C)CC[C@]5(C)CC[C@@]34C)C1(C)C |
| 3277 | C[C@@H]1O[C@@H](O[C@@H]2[C@H](Oc3cc(O)cc(O)c3C2=O)c4ccc(O)c(O)c4)[C@H](O)[C@H](O)[C@H]1O |
| 3278 | O[C@@H]1CC(=C[C@@H](OC(=O)\C=C\c2ccc(O)c(O)c2)[C@H]1O)C(=O)[O-] |
| 3279 | COC(=O)[C@]1(C)CCC[C@@]2(C)[C@H]1CCc3cc(ccc23)C(C)C |
| 3280 | CC(C)C(=C)CC[C@H]([C@H]1CC[C@@]2(C)C3=CC[C@H]4C(C)(C)[C@@H](O)CC[C@]4(C)C3=CC[C@]12C)C(=O)O |
| 3281 | CC(C)C(=C)CC[C@H]([C@H]1CC[C@@]2(C)C3=CC[C@H]4C(C)(C)[C@@H](O)CC[C@]4(C)C3=CC[C@]12C)C(=O)[O-] |
| 3282 | CC(C)C(=C)CC[C@H]([C@H]1[C@H](O)C[C@@]2(C)C3=CC[C@H]4C(C)(C)[C@@H](O)CC[C@]4(C)C3=CC[C@]12C)C(=O)O |
| 3283 | CC(C)C(=C)CC[C@H]([C@H]1[C@H](O)C[C@@]2(C)C3=CC[C@H]4C(C)(C)[C@@H](O)CC[C@]4(C)C3=CC[C@]12C)C(=O)[O-] |
| 3284 | C[C@@H]1O[C@@H](O[C@@H]2[C@H](Oc3cc(O)cc(O)c3C2=O)c4ccc(O)cc4)[C@H](O)[C@H](O)[C@H]1O |
| 3285 | O[C@@H]1[C@H](Oc2cc(O)cc(O)c2C1=O)c3ccc(O)c(O)c3 |
| 3286 | COc1ccc(O)c(c1)C2=COc3cc(O)ccc3C2=O |
| 3287 | COc1ccc(O)c(c1)C2=COc3cc([O-])ccc3C2=O |
| 3288 | [O-]C(=O)CCCCCCCCCC=C |
| 3289 | CC(C)C(=C)CC[C@H]([C@H]1CC[C@@]2(C)C3=C(CC[C@]12C)[C@@]4(C)CC[C@H](O)C(C)(C)[C@@H]4CC3)C(=O)O |
| 3290 | CC(C)C(=C)CC[C@H]([C@H]1CC[C@@]2(C)C3=C(CC[C@]12C)[C@@]4(C)CC[C@H](O)C(C)(C)[C@@H]4CC3)C(=O)[O-] |
| 3291 | CC(C)C(=C)CC[C@H]([C@H]1[C@H](O)C[C@@]2(C)C3=CC[C@H]4C(C)(C)[C@H](O)CC[C@]4(C)C3=CC[C@]12C)C(=O)O |
| 3292 | CC(C)C(=C)CC[C@H]([C@H]1[C@H](O)C[C@@]2(C)C3=CC[C@H]4C(C)(C)[C@H](O)CC[C@]4(C)C3=CC[C@]12C)C(=O)[O-] |
| 3293 | CC(C)[C@@H](C)\C=C\[C@@H](C)[C@H]1CC[C@H]2C3=CC=C4C[C@@H](O)CC[C@]4(C)[C@H]3CC[C@]12C |
| 3294 | CC(=CCC[C@H]([C@H]1[C@@H](C[C@@]2(C)C3=CC[C@H]4C(C)(C)[C@@H](O)CC[C@]4(C)C3=CC[C@]12C)OC(=O)C)C(=O)[O-])C |
| 3295 | CC(C)C(=C)CC[C@H]([C@H]1[C@H](O)C[C@@]2(C)C3=CC[C@H]4C(C)(C)[C@H](CC[C@]4(C)C3=CC[C@]12C)OC(=O)c5ccc(O)cc5)C(=O)O |
| 3296 | CC(C)C(=C)CC[C@H]([C@H]1[C@H](O)C[C@@]2(C)C3=CC[C@H]4C(C)(C)[C@H](CC[C@]4(C)C3=CC[C@]12C)OC(=O)c5ccc(O)cc5)C(=O)[O-] |
| 3297 | CC(C)C(=C)CC[C@H]([C@H]1[C@H](O)C[C@@]2(C)C3=CC[C@H]4C(C)(C)[C@H](CC[C@]4(C)C3=CC[C@]12C)OC(=O)c5ccc([O-])cc5)C(=O)[O-] |
| 3298 | CC(C)(O)C(=C)CC[C@H]([C@H]1[C@H](O)C[C@@]2(C)C3=CC[C@H]4C(C)(C)[C@H](O)CC[C@]4(C)C3=CC[C@]12C)C(=O)O |
| 3299 | CC(C)(O)C(=C)CC[C@H]([C@H]1[C@H](O)C[C@@]2(C)C3=CC[C@H]4C(C)(C)[C@H](O)CC[C@]4(C)C3=CC[C@]12C)C(=O)[O-] |
| 3300 | CC(=CCC[C@H]([C@H]1CC[C@@]2(C)C3=CC[C@H]4C(C)(C)[C@@H](O)CC[C@]4(C)C3=CC[C@]12C)C(=O)[O-])C |
| 3301 | C[C@@H]1O[C@@H](O[C@@H]2[C@H](Oc3cc(O)cc(O)c3C2=O)c4cc(O)cc(O)c4)[C@H](O)[C@H](O)[C@H]1O |
| 3302 | C[C@@H]1O[C@@H](O[C@@H]2[C@H](Oc3cc(O)cc(O)c3C2=O)c4cc(O)cc([O-])c4)[C@H](O)[C@H](O)[C@H]1O |
| 3303 | C[C@@H]1O[C@@H](O[C@H]2[C@H](Oc3cc(O)cc(O)c3C2=O)c4ccc(O)cc4)[C@H](O)[C@H](O)[C@H]1O |
| 3304 | CC(C)C(=C)CC[C@H]([C@H]1[C@H](O)C[C@@]2(C)C3=C(CC[C@]12C)[C@@]4(C)CC[C@H](OC(=O)C)C(C)(C)[C@@H]4CC3)C(=O)O |
| 3305 | CC(C)C(=C)CC[C@H]([C@H]1[C@H](O)C[C@@]2(C)C3=C(CC[C@]12C)[C@@]4(C)CC[C@H](OC(=O)C)C(C)(C)[C@@H]4CC3)C(=O)[O-] |
| 3306 | COC(=O)[C@H](CCC(=C)C(C)C)[C@H]1[C@H](O)C[C@@]2(C)C3=C(CC[C@]12C)[C@@]4(C)CC[C@H](OC(=O)C)C(C)(C)[C@@H]4CC3 |
| 3307 | CC(=CCC[C@@H]([C@H]1CC[C@@]2(C)C3=C(CC[C@]12C)[C@@]4(C)CCC(=O)C(C)(C)[C@@H]4CC3)C(=O)[O-])C |
| 3308 | CC(C)C(=C)CC[C@H]([C@H]1[C@H](O)C[C@@]2(C)C3=CC[C@@H]4C(C)(C)C(=O)CC[C@]4(C)C3=CC[C@]12C)C(=O)O |
| 3309 | CC(C)C(=C)CC[C@H]([C@H]1[C@H](O)C[C@@]2(C)C3=CC[C@@H]4C(C)(C)C(=O)CC[C@]4(C)C3=CC[C@]12C)C(=O)[O-] |
| 3310 | CC(C)C(=C)CC[C@@H]([C@H]1[C@H](O)C[C@@]2(C)C3=CC[C@@H](C(=C)C)[C@](C)(CCC(=O)[O-])C3=CC[C@]12C)C(=O)O |
| 3311 | CC(C)C(=C)CC[C@@H]([C@H]1[C@H](O)C[C@@]2(C)C3=CC[C@@H](C(=C)C)[C@](C)(CCC(=O)[O-])C3=CC[C@]12C)C(=O)[O-] |
| 3312 | CC(=CCC[C@H]([C@H]1[C@H](O)C[C@@]2(C)C3=CC[C@@H](C(=C)C)[C@](C)(CCC(=O)[O-])C3=CC[C@]12C)C(=O)[O-])C |
| 3313 | CC(C)C(=C)CC[C@H]([C@H]1CC[C@@]2(C)C3=CC[C@H](C(=C)C)[C@](C)(CCC(=O)[O-])C3=CC[C@]12C)C(=O)O |
| 3314 | CC(C)C(=C)CC[C@H]([C@H]1CC[C@@]2(C)C3=CC[C@H](C(=C)C)[C@](C)(CCC(=O)[O-])C3=CC[C@]12C)C(=O)[O-] |
| 3315 | CC(=C)[C@@H]1CC=C2C(=CC[C@]3(C)[C@@H]([C@@H](CCC(=C)C(C)(C)O)C(=O)O)[C@H](O)C[C@@]23C)[C@@]1(C)CCC(=O)[O-] |
| 3316 | CC(=C)[C@@H]1CC=C2C(=CC[C@]3(C)[C@@H]([C@@H](CCC(=C)C(C)(C)O)C(=O)[O-])[C@H](O)C[C@@]23C)[C@@]1(C)CCC(=O)[O-] |
| 3317 | COC(=O)CC[C@@]1(C)[C@@H](CC=C2C1=CC[C@]3(C)[C@@H]([C@H](CCC(=C)C(C)(C)O)C(=O)O)[C@H](O)C[C@@]23C)C(=C)C |
| 3318 | COC(=O)CC[C@@]1(C)[C@@H](CC=C2C1=CC[C@]3(C)[C@@H]([C@H](CCC(=C)C(C)(C)O)C(=O)[O-])[C@H](O)C[C@@]23C)C(=C)C |
| 3319 | CC(=CCC[C@H]([C@H]1[C@H](O)C[C@@]2(C)C3=C(CC[C@]12C)[C@@](C)(CCC(=O)[O-])[C@@H](CC3)C(=C)C)C(=O)[O-])C |
| 3320 | CC(C)C(=C)CC[C@H]([C@H]1[C@H](O)C[C@@]2(C)C3=C(CC[C@]12C)[C@@](C)(CCC(=O)[O-])[C@@H](CC3)C(=C)C)C(=O)O |
| 3321 | CC(C)C(=C)CC[C@H]([C@H]1[C@H](O)C[C@@]2(C)C3=C(CC[C@]12C)[C@@](C)(CCC(=O)[O-])[C@@H](CC3)C(=C)C)C(=O)[O-] |
| 3322 | COc1cc(\C=C\C(=O)OC[C@H]2O[C@@](COC(=O)\C=C\c3ccc(O)c(OC)c3)(O[C@H]4O[C@H](COC(=O)C)[C@@H](OC(=O)C)[C@H](O)[C@H]4OC(=O)C)[C@@H](OC(=O)\C=C\c5ccc(O)c(OC)c5)[C@@H]2O)ccc1O |
| 3323 | COc1cc(\C=C\C(=O)OC[C@H]2O[C@@](COC(=O)\C=C\c3ccc(O)c(OC)c3)(O[C@H]4O[C@H](COC(=O)C)[C@@H](O)[C@H](O)[C@H]4OC(=O)C)[C@@H](OC(=O)\C=C\c5ccc(O)c(OC)c5)[C@@H]2O)ccc1O |
| 3324 | COc1cc(\C=C\C(=O)OC[C@H]2O[C@@](CO)(O[C@H]3O[C@H](COC(=O)C)[C@@H](OC(=O)C)[C@H](O)[C@H]3OC(=O)C)[C@@H](OC(=O)\C=C\c4ccc(O)c(OC)c4)[C@@H]2O)ccc1O |
| 3325 | COc1cc(\C=C\C(=O)OC[C@H]2O[C@@](COC(=O)\C=C\c3ccc(O)cc3)(O[C@H]4O[C@H](COC(=O)C)[C@@H](OC(=O)C)[C@H](O)[C@@H]4OC(=O)C)[C@@H](OC(=O)\C=C\c5ccc(O)c(OC)c5)[C@@H]2O)ccc1O |
| 3326 | COc1cc(\C=C\C(=O)OC[C@H]2O[C@@](COC(=O)\C=C\c3ccc(O)cc3)(O[C@H]4O[C@H](COC(=O)C)[C@@H](O)[C@H](O)[C@H]4OC(=O)C)[C@@H](OC(=O)\C=C\c5ccc(O)c(OC)c5)[C@@H]2O)ccc1O |
| 3327 | C[C@@H]1O[C@@H](O[C@H]2[C@H](Oc3cc(O)cc(O)c3C2=O)c4cc(O)cc(O)c4)[C@H](O)[C@H](O)[C@H]1O |
| 3328 | C[C@@H]1O[C@@H](O[C@H]2[C@H](Oc3cc(O)cc(O)c3C2=O)c4cc(O)cc([O-])c4)[C@H](O)[C@H](O)[C@H]1O |
| 3329 | COc1cc(cc(OC)c1O)C(=O)[O-] |
| 3330 | COc1cc(cc(OC)c1[O-])C(=O)[O-] |
| 3331 | CC(=CCC[C@H]([C@H]1CC[C@@]2(C)C3=C(CC[C@]12C)[C@@]4(C)CC[C@H](O)C(C)(C)[C@@H]4CC3)C(=O)[O-])C |
| 3332 | CC(=O)c1c(O)cc(O[C@@H]2O[C@H](CO)[C@@H](O)[C@H](O)[C@H]2O)cc1O[C@@H]3O[C@H](CO)[C@@H](O)[C@H](O)[C@H]3O |
| 3333 | CC(=O)c1c([O-])cc(O[C@@H]2O[C@H](CO)[C@@H](O)[C@H](O)[C@H]2O)cc1O[C@@H]3O[C@H](CO)[C@@H](O)[C@H](O)[C@H]3O |
| 3334 | C[C@@H]1O[C@@H](OC2=COc3cc(O)cc(O)c3C2=O)[C@H](O)[C@H](O)[C@H]1O |
| 3335 | CC(C)C(=C)CC[C@H]([C@H]1[C@H](O)C[C@@]2(C)C3=C(CC[C@]12C)[C@@]4(C)CC[C@H](O)C(C)(C)[C@@H]4CC3)C(=O)O |
| 3336 | CC(C)C(=C)CC[C@H]([C@H]1[C@H](O)C[C@@]2(C)C3=C(CC[C@]12C)[C@@]4(C)CC[C@H](O)C(C)(C)[C@@H]4CC3)C(=O)[O-] |
| 3337 | COC(=O)[C@H](CCC(=C)C(C)C)[C@H]1[C@H](O)C[C@@]2(C)C3=C(CC[C@]12C)[C@@]4(C)CC[C@H](O)C(C)(C)[C@@H]4CC3 |
| 3338 | Cc1cccc(C)c1 |
| 3339 | Cc1ccc(C)cc1 |
| 3340 | CC1=CC[C@@H](CC1)C(C)(C)O |
| 3341 | Oc1ccc2[C@@H]3Oc4cc5OCOc5cc4[C@@H]3COc2c1 |
| 3342 | OC[C@H]1O[C@@H]([C@H](O)[C@@H](O)[C@@H]1O)c2c(O)c([C@H]3O[C@@H](CO)[C@@H](O)[C@H](O)[C@@H]3O)c4OC(=CC(=O)c4c2O)c5ccc(O)cc5 |
| 3343 | OC[C@@H]1O[C@@H]([C@@H](O)[C@H](O)[C@H]1O)c2c(O)c([C@H]3O[C@@H](CO)[C@H](O)[C@@H](O)[C@@H]3O)c4OC(=CC(=O)c4c2O)c5ccc([O-])cc5 |
| 3344 | CC1=C[C@@H]2[C@H](CC1)[C@](C)(O)CCC2(C)C |
| 3345 | CC(=CCC1=C(Oc2c3C=CC(C)(C)Oc3cc([O-])c2C1=O)c4ccc(O)cc4O)C |
| 3346 | [O-]C(=O)c1c[nH]c2ccccc12 |
| 3347 | Oc1ccc(cc1)[C@@H]2CC(=O)c3ccc(O)cc3O2 |
| 3348 | CC(=C)[C@@H]1CC[C@]2(CC[C@@]3(C)[C@@H](CC[C@H]4[C@@]5(C)CC[C@H](O)C(C)(C)[C@H]5CC[C@@]34C)[C@@H]12)C(=O)O |
| 3349 | CC(=C)[C@@H]1CC[C@]2(CC[C@@]3(C)[C@@H](CC[C@H]4[C@@]5(C)CC[C@H](O)C(C)(C)[C@H]5CC[C@@]34C)[C@@H]12)C(=O)[O-] |
| 3350 | COc1c(CC=C(C)C)c(O)cc2OC(=O)c3c(oc4cc(O)ccc34)c12 |
| 3351 | COC1=C(Oc2cc(OC)cc(O)c2C1=O)c3ccc(O)cc3 |
| 3352 | COC1=C(Oc2cc(OC)cc(O)c2C1=O)c3ccc([O-])cc3 |
| 3353 | C[C@@H]1C[C@H]2[C@@H](C=C1)C2(C)C |
| 3354 | COc1ccc2[C@H]3COc4cc(O)ccc4[C@H]3Oc2c1 |
| 3355 | CC1(C)CC[C@@]2(CC[C@@]3(C)C(=CC[C@@H]4[C@]5(C)CC[C@@H](O)C(C)(C)[C@H]5CC[C@]34C)[C@H]2C1)C(=O)O |
| 3356 | CC1(C)CC[C@@]2(CC[C@@]3(C)C(=CC[C@@H]4[C@]5(C)CC[C@@H](O)C(C)(C)[C@H]5CC[C@]34C)[C@H]2C1)C(=O)[O-] |
| 3357 | CCc1ccccc1 |
| 3358 | C[C@@H]1O[C@H](OC[C@@H]2O[C@H](OC3=C(Oc4cc(O)cc(O)c4C3=O)c5ccc(O)cc5)[C@@H](O)[C@H](O)[C@H]2O)[C@@H](O)[C@H](O)[C@H]1O |
| 3359 | C[C@@H]1O[C@H](OC[C@@H]2O[C@H](OC3=C(Oc4cc(O)cc(O)c4C3=O)c5ccc([O-])cc5)[C@@H](O)[C@H](O)[C@H]2O)[C@@H](O)[C@H](O)[C@H]1O |
| 3360 | Oc1cc(O)c2C(=O)C[C@H](Oc2c1)c3ccccc3 |
| 3361 | CC(=CCc1c(O)c(O)ccc1C2=C([O-])C(=O)c3cc(O)c(O)cc3O2)C |
| 3362 | CC[C@H](CC[C@@H](C)[C@@H]1CC[C@@H]2[C@H]3CC=C4C[C@H](O)CC[C@@]4(C)[C@@H]3CC[C@]12C)C(C)C |
| 3363 | CC(=CCc1c(O)cc(O)c2C(=O)C(=COc12)c3ccc(O)cc3)C |
| 3364 | CC(=CCc1c(O)cc(O)c2C(=O)C(=COc12)c3ccc([O-])cc3)C |
| 3365 | Oc1ccc(cc1)C2=CC(=O)c3ccc(O)cc3O2 |
| 3366 | Oc1ccc2C(=O)C=C(Oc2c1)c3ccc([O-])cc3 |
| 3367 | [O-]c1ccc(cc1)C2=CC(=O)c3ccc([O-])cc3O2 |
| 3368 | COc1cc(ccc1O)C2=C(O[C@H]3O[C@H](CO[C@H]4O[C@@H](C)[C@@H](O)[C@@H](O)[C@H]4O)[C@@H](O)[C@H](O)[C@H]3O)C(=O)c5c(O)cc(O)cc5O2 |
| 3369 | COc1cc(ccc1[O-])C2=C(O[C@H]3O[C@@H](CO[C@H]4O[C@@H](C)[C@H](O)[C@@H](O)[C@@H]4O)[C@H](O)[C@@H](O)[C@@H]3O)C(=O)c5c(O)cc(O)cc5O2 |
| 3370 | COc1ccc(cc1)C2=COc3cc(O)ccc3C2=O |
| 3371 | COc1ccc(cc1)C2=COc3cc([O-])ccc3C2=O |
| 3372 | CC1(C)[C@@H]2CCC(=C[C@H]12)C=O |
| 3373 | C[C@@H]1O[C@H](OC[C@@H]2O[C@H](OC3=C(Oc4cc(O)cc(O)c4C3=O)c5ccc(O)c(O)c5)[C@@H](O)[C@H](O)[C@H]2O)[C@@H](O)[C@H](O)[C@H]1O |
| 3374 | COc1ccc(cc1O)C2=COc3cc(O)ccc3C2=O |
| 3375 | COc1ccc(cc1O)C2=COc3cc([O-])ccc3C2=O |
| 3376 | Oc1ccc(cc1)[C@H]2CC(=O)c3c(O)cc(O)cc3O2 |
| 3377 | OC[C@@H]1O[C@H](OC2=C(Oc3cc(O)cc(O)c3C2=O)c4ccc(O)c(O)c4)[C@@H](O)[C@H](O)[C@H]1O |
| 3378 | CC(=CCc1c(O)c(CC=C(C)C)c2OC=C(C(=O)c2c1O)c3ccc(O)cc3)C |
| 3379 | CC(=CCc1c(O)c(CC=C(C)C)c2OC=C(C(=O)c2c1O)c3ccc([O-])cc3)C |
| 3380 | COc1ccc(cc1)C2=COc3cc(O)c(OC)cc3C2=O |
| 3381 | COc1ccc(cc1)C2=COc3cc([O-])c(OC)cc3C2=O |
| 3382 | CO[C@@H]1[C@@H]([C@@H](O)Oc2cc(O[C@H]3O[C@@H](CO)[C@H](O)[C@@H](O)[C@@H]3O)ccc12)c4ccccc4 |
| 3383 | CCCCOC\C=C\CCC |
| 3384 | CC(=CCc1cc(ccc1O)[C@@H]2Oc3c(CC=C(C)C)c(O)ccc3C(=O)[C@@H]2O)C |
| 3385 | CC1(C)[C@@H](O)CC[C@]2(C)[C@H]1CC[C@]3(C)[C@H]2C(=O)C=C4[C@H]5C[C@@](C)(CC[C@@]5(C)CC[C@@]34C)C(=O)[O-] |
| 3386 | CC(=CCc1cc(ccc1O)[C@@H]2CC(=O)c3ccc4OC(C)(C)C=Cc4c3O2)C |
| 3387 | C\C=C/c1cc(ccc1OC(C)C)[C@@H]2CC(=O)c3ccc(O)c(CC=C(C)C)c3O2 |
| 3388 | O[C@@H]1O[C@H]([C@@H](O)[C@H](O)[C@@H]1O)C(=O)[O-] |
| 3389 | COc1cc2OC[C@@H](C(=O)c2c(O)c1CC=C(C)C)c3ccc(O)cc3O |
| 3390 | COc1cc2OC(=C(C(=O)c2cc1CC=C(C)C)c3ccccc3)Oc4ccc(O)cc4O |
| 3391 | COc1cc2OC(=C(C(=O)c2cc1CC=C(C)C)c3ccccc3)Oc4ccc(O)cc4[O-] |
| 3392 | CC1(C)Oc2c(C=C1)c(O)ccc2[C@H]3COc4cc(O)cc(O)c4C3=O |
| 3393 | CC1(C)Oc2c(C=C1)c(O)ccc2[C@H]3COc4cc(O)cc([O-])c4C3=O |
| 3394 | COc1c(CC=C(C)C)c(O)cc2OC[C@H](Cc12)c3ccc(O)cc3O |
| 3395 | COc1cc2OC[C@@H](Cc2c(OC)c1CC=C(C)C)c3ccc(O)cc3O |
| 3396 | COc1ccc([C@H]2COc3cc(O)ccc3C2)c(O)c1CC=C(C)C |
| 3397 | COc1cc(O)cc2OC(=O)c3c(oc4cc(O)ccc34)c12 |
| 3398 | COc1cc([O-])cc2OC(=O)c3c(oc4cc(O)ccc34)c12 |
| 3399 | CC1(C)Oc2ccc(\C=C\C(=O)c3ccc(O)cc3O)cc2C=C1 |
| 3400 | CC(=CCc1cc(C[C@@H](O)C(=O)c2cc(CC=C(C)C)c(O)cc2O)ccc1O)C |
| 3401 | COc1cc2OC=C(C(=O)c2c(O)c1CC=C(C)C)c3ccc(O)c(CC=C(C)C)c3O |
| 3402 | CC(C)Oc1c(C=C)c(O)ccc1C2=COc3c(CC=C(C)C)c(O)c(CC=C(C)C)c(O)c3C2=O |
| 3403 | CC(C)Oc1c(C=C)c([O-])ccc1C2=COc3c(CC=C(C)C)c(O)c(CC=C(C)C)c(O)c3C2=O |
| 3404 | CC(=CCc1c(O)ccc(C2=COc3cc(O)c(CCC(C)(C)O)c(O)c3C2=O)c1O)C |
| 3405 | CC1(C)Oc2ccc3C=C(C(=O)Oc3c2C=C1)c4ccc(O)cc4O |
| 3406 | CC(=CCc1c(O)ccc([C@H]2COc3c(C2)ccc(O)c3CC=C(C)C)c1O)C |
| 3407 | CC(=CCc1cc(\C=C\C(=O)c2ccc(O)cc2O)ccc1O)C |
| 3408 | CC(=CCc1c(C)ccc2[C@H]3Oc4cc(C)ccc4[C@H]3COc12)C |
| 3409 | COc1c2C[C@H](Oc2cc3OC(=O)C(=Cc13)c4ccc(O)cc4O)C(C)(C)O |
| 3410 | CC(=CCc1cc(ccc1O)\C(=C\C(=O)c2cc(CC=C(C)C)c(O)cc2O)\O)C |
| 3411 | CC(=CCc1cc(ccc1O)\C(=C\C(=O)c2cc(CC=C(C)C)c(O)cc2O)\[O-])C |
| 3412 | C[C@@H]1O[C@H](O[C@H]2[C@H](O)[C@@H](O)[C@H](CO)O[C@@H]2Oc3cc(O)c4C(=O)C[C@H](Oc4c3)c5ccc(O)cc5)[C@@H](O)[C@H](O)[C@H]1O |
| 3413 | CC1(C)Oc2c(O)cc(cc2C=C1)C3=COc4cc(O)cc(O)c4C3=O |
| 3414 | CC(=CCc1c(O)cc(O)c2C(=O)C(=C(Oc12)c3ccccc3)[O-])C |
| 3415 | CC(=CCc1c(O)cc(O)c2C(=O)[C@H](O)[C@@H](Oc12)c3ccccc3)C |
| 3416 | CCCC\C=C\C=C |
| 3417 | CC(=CCc1cc(\C=C\C(=O)c2ccc(O)c(CC=C(C)C)c2O)ccc1O)C |
| 3418 | CCCCOC(=O)c1ccccc1 |
| 3419 | CC1(C)Oc2ccc([C@@H]3COc4cc(O)ccc4C3)c(O)c2C=C1 |
| 3420 | COc1ccc(C2=Cc3ccc([O-])cc3OC2)c(O)c1 |
| 3421 | COc1ccc(cc1)C(=O)\C=C\c2ccc(O)cc2OC |
| 3422 | COc1cc(O)ccc1\C=C/C(=O)c2ccc(O)cc2 |
| 3423 | COc1cc(O)ccc1\C=C/C(=O)c2ccc([O-])cc2 |
| 3424 | O=C(CC(=O)c1ccccc1)c2ccccc2 |
| 3425 | CC1(C)Oc2c(C=C1)c(O)ccc2c3oc4cc(O)ccc4c3 |
| 3426 | C[C@@H]1CCC[C@H]1C |
| 3427 | COc1cc2oc(c(O)c2cc1OC)c3ccc(O)c(CC=C(C)C)c3 |
| 3428 | COc1c(O)c(O)ccc1\C=C\C(=O)c2ccc(O)cc2 |
| 3429 | COc1c(O)c(O)ccc1\C=C\C(=O)c2ccc([O-])cc2 |
| 3430 | COc1c(\C=C/C(=O)c2ccc(O)cc2)ccc(O)c1CC=C(C)C |
| 3431 | COc1c(\C=C/C(=O)c2ccc([O-])cc2)ccc(O)c1CC=C(C)C |
| 3432 | COc1c(O)c(O)ccc1\C=C/C(=O)c2ccc(O)c(CC=C(C)C)c2 |
| 3433 | CC(=CCc1cc(ccc1O)[C@H]2CC(=O)c3ccc(O)c(CC=C(C)C)c3O2)C |
| 3434 | CC1(C)[C@@H](CC[C@]2(C)[C@H]1CC[C@@]3(C)[C@H]2C(=O)C=C4[C@@H]5C[C@](C)(CC[C@]5(C)CC[C@]34C)C(=O)[O-])O[C@H]6O[C@H]([C@H](O)[C@@H](O)[C@@H]6O[C@H]7OC[C@@](O)(CO)[C@@H]7O)C(=O)[O-] |
| 3435 | CCCC(C)(C)C |
| 3436 | COc1cc(O)c(cc1\C=C\C(=O)c2ccc(O)cc2O)C(C)(C)C=C |
| 3437 | COc1cc(O)c(c2OC(=O)C(=Cc12)c3ccc(O)cc3O)C(C)(C)C=C |
| 3438 | CC1(C)[C@H](O)CC[C@]2(C)[C@H]1CC[C@]3(C)[C@H]2C(=O)C=C4[C@H]5C[C@@](C)([C@@H]6C[C@@]5(C)[C@H](C[C@]34C)O6)C(=O)[O-] |
| 3439 | CC(=CCc1cc2C(=O)C=C(Oc2cc1O)c3ccc(O)cc3)C |
| 3440 | CC(=CCc1cc2C(=O)C=C(Oc2cc1O)c3ccc([O-])cc3)C |
| 3441 | CC(=CCc1cc(ccc1O)C2=CC(=O)c3cc(CC=C(C)C)c(O)cc3O2)C |
| 3442 | CC(=CCc1c(O)cc2OC(=C([O-])C(=O)c2c1O)c3ccc(O)cc3)C |
| 3443 | CC(=CCc1c(O)cc2OC(=C([O-])C(=O)c2c1O)c3ccc([O-])cc3)C |
| 3444 | CC(=CCc1cc(ccc1O)C2=CC(=O)c3c(O)cc(O)cc3O2)C |
| 3445 | COc1cc(O)c(C2=COc3cc(O)ccc3C2=O)c(OC)c1CC=C(C)C |
| 3446 | COc1cc(O)c(C2=COc3cc([O-])ccc3C2=O)c(OC)c1CC=C(C)C |
| 3447 | COc1ccc(cc1)C2=COc3cc(O)c(CC=C(C)C)c(O)c3C2=O |
| 3448 | COc1ccc(cc1O)C2=COc3cc(O)c(CC=C(C)C)c(O)c3C2=O |
| 3449 | C\C(=C/Cc1c(O)cc(O)c2C(=O)C(=COc12)c3ccc(O)cc3)\CO |
| 3450 | C\C(=C/Cc1c(O)cc(O)c2C(=O)C(=COc12)c3ccc([O-])cc3)\CO |
| 3451 | CCC[C@H](C)C(C)C |
| 3452 | COc1cc(O)c2C(=O)C(=COc2c1)c3ccc(O)cc3 |
| 3453 | COc1cc(O)c2C(=O)C(=COc2c1)c3ccc([O-])cc3 |
| 3454 | OC[C@@H]1O[C@H](Oc2ccc(cc2)[C@@H]3CC(=O)c4ccc(O)cc4O3)[C@@H](O[C@H]5OC[C@@](O)(COC(=O)c6c[nH]c7ccccc67)[C@@H]5O)[C@H](O)[C@H]1O |
| 3455 | COc1ccc(cc1O)C2=COc3c(C\C=C(/C)\CO)c(O)cc(O)c3C2=O |
| 3456 | CC(=CCc1cc(cc(O)c1O)[C@@H]2CC(=O)c3c(O)cc(O)c(CC=C(C)C)c3O2)C |
| 3457 | CC(=CCc1c(O)cc(O)c2C(=O)C(=COc12)c3ccc(O)c(O)c3)C |
| 3458 | COc1ccc(cc1)C2=COc3c(CC=C(C)C)c(O)cc(O)c3C2=O |
| 3459 | COc1ccc(C2=COc3cc(O)c(CC=C(C)C)c(O)c3C2=O)c(O)c1 |
| 3460 | CC(=CCc1c(O)cc2OC(=CC(=O)c2c1O)c3ccc(O)c(O)c3)C |
| 3461 | CC(=CCc1c(O)cc2OC(=C([O-])C(=O)c2c1O)c3ccc(O)c(O)c3)C |
| 3462 | CC(=CCc1cc(ccc1O)C2=CC(=O)c3c(O)c(CC=C(C)C)c(O)cc3O2)C |
| 3463 | CC(=CCc1c(O)cc(O)c(CC=C(C)C)c1CCc2ccc(O)c(O)c2)C |
| 3464 | CC(=CCc1c(O)cc(CCc2ccc(O)c(O)c2)c(CC=C(C)C)c1O)C |
| 3465 | CC[C@H](C)C(C)C |
| 3466 | CC(=CCc1c(O)cc2OC(C)(C)[C@H](O)Cc2c1CCc3ccc(O)c(O)c3)C |
| 3467 | CC(=CCc1c(O)c(CC=C(C)C)c2CCc3cc(O)c(O)cc3c2c1O)C |
| 3468 | CC(=CCc1c(O)c(CC=C(C)C)c2CCc3cc([O-])c(O)cc3c2c1O)C |
| 3469 | CC(=CCc1c(O)cc(O)c2c1CCc3cc(O)c(O)cc23)C |
| 3470 | CC(=CCc1c(O)cc(O)c2c1CCc3cc([O-])c(O)cc23)C |
| 3471 | COc1c(CC=C(C)C)c(O)cc(O)c1C2=COc3cc(O)ccc3C2=O |
| 3472 | COc1c(CC=C(C)C)c(O)cc(O)c1C2=COc3cc([O-])ccc3C2=O |
| 3473 | CC1(C)[C@@H](CC[C@]2(C)[C@H]1CC[C@@]3(C)[C@H]2C(=O)C=C4[C@@H]5C[C@](C)(CC[C@]5(C)CC[C@]34C)C(=O)[O-])O[C@@H]6O[C@@H]([C@@H](O)[C@H](O)[C@H]6O[C@@H]7O[C@@H]([C@@H](O)[C@H](O)[C@H]7O)C(=O)[O-])C(=O)[O-] |
| 3474 | COc1c(CC=C(C)C)c(O)cc2OC[C@@H](Cc12)c3ccc(O)c(CC=C(C)C)c3O |
| 3475 | COc1c(CC=C(C)C)c(O)cc2OC(=O)C(=Cc12)c3ccc(O)cc3O |
| 3476 | COc1c(CC=C(C)C)c([O-])cc2OC(=O)C(=Cc12)c3ccc(O)cc3O |
| 3477 | COc1cc2OC(=O)C(=Cc2c(OC)c1CC=C(C)C)c3ccc(O)cc3O |
| 3478 | Cc1cc(C)c2CCCCc2[nH+]1 |
| 3479 | Cc1cc(C)c2CCCCc2n1 |
| 3480 | CC(=CCc1c(O)ccc(C(=O)\C=C\c2ccc(O)cc2O)c1O)C |
| 3481 | COc1c(CC=C(C)C)c(O)cc2oc(cc12)c3ccc(O)cc3O |
| 3482 | CC(=CCc1c(O)ccc(C2=COc3cc(O)cc(O)c3C2=O)c1O)C |
| 3483 | CC1(C)Oc2ccc(C3=COc4cc(O)cc(O)c4C3=O)c(O)c2C=C1 |
| 3484 | CC1(C)Oc2ccc([C@@H]3COc4cc(O)cc(O)c4C3=O)c(O)c2C=C1 |
| 3485 | CC1(C)Oc2ccc([C@@H]3COc4cc(O)cc([O-])c4C3=O)c(O)c2C=C1 |
| 3486 | CC1(C)[C@@H](CC[C@@]2(C)[C@@H]1CC[C@]3(C)[C@@H]2C=CC4=C5C[C@](C)(CC[C@@]5(C)CC[C@@]34C)C(=O)[O-])O[C@H]6O[C@H]([C@@H](O)[C@@H](O)[C@H]6O[C@@H]7O[C@H]([C@@H](O)[C@@H](O)[C@@H]7O)C(=O)[O-])C(=O)[O-] |
| 3487 | CC1(C)[C@@H](O)CC[C@]2(C)[C@H]1CC[C@@]3(C)[C@@H]2C=CC4=C5C[C@](C)(CC[C@]5(C)CC[C@]34C)C(=O)[O-] |
| 3488 | CC1(C)[C@H](CC[C@]2(C)[C@H]1CC[C@@]3(C)[C@@H]2CC=C4[C@H]5C[C@@]6(C)C[C@H](OC6=O)[C@@]5(C)CC[C@@]34C)O[C@H]7O[C@H]([C@H](O)[C@@H](O)[C@@H]7O[C@H]8O[C@H]([C@H](O)[C@@H](O)[C@@H]8O[C@H]9O[C@H]([C@H](O)[C@@H](O)[C@@H]9O)C(=O)[O-])C(=O)[O-])C(=O)[O-] |
| 3489 | CC1(C)[C@H](O)CC[C@]2(C)[C@@H]1CC[C@@]3(C)[C@H]2CC=C4[C@@H]5C[C@]6(C)C[C@@H](OC6=O)[C@]5(C)CC[C@]34C |
| 3490 | CC[C@H](C)CC(C)C |
| 3491 | CC1(C)Oc2ccc3[C@H]4Oc5cc(O)ccc5[C@H]4COc3c2C=C1 |
| 3492 | CC1(C)Oc2ccc3[C@@H]4Oc5cc([O-])ccc5[C@@H]4COc3c2C=C1 |
| 3493 | C[C@@]12CC[C@](C)(C[C@H]1C3=CC(=O)[C@H]4[C@]5(C)CC[C@H](O[C@@H]6O[C@H]([C@H](O)[C@@H](O)[C@H]6O[C@@H]7O[C@H]([C@H](O)[C@@H](O)[C@H]7O)C(=O)[O-])C(=O)[O-])[C@@](C)(CO)[C@@H]5CC[C@]4(C)[C@@]3(C)CC2)C(=O)[O-] |
| 3494 | C[C@@]12CC[C@@](C)(C[C@H]1C3=CC(=O)[C@H]4[C@]5(C)CC[C@H](O)[C@](C)(CO)[C@@H]5CC[C@]4(C)[C@@]3(C)CC2)C(=O)[O-] |
| 3495 | C[C@]12CC[C@@](C)(C[C@@H]1C3=CC[C@H]4[C@]5(C)CC[C@@H](O[C@H]6O[C@@H]([C@@H](O)[C@H](O)[C@H]6O[C@@H]7O[C@@H]([C@@H](O)[C@H](O)[C@H]7O)C(=O)[O-])C(=O)[O-])[C@](C)(CO)[C@@H]5CC[C@]4(C)[C@@]3(C)CC2)C(=O)[O-] |
| 3496 | C[C@]12CC[C@@](C)(C[C@H]1C3=CC[C@H]4[C@]5(C)CC[C@@H](O)[C@@](C)(CO)[C@@H]5CC[C@]4(C)[C@@]3(C)CC2)C(=O)[O-] |
| 3497 | CC(=CCc1cc(\C=C\C(=O)c2ccc(O)cc2O)cc(O)c1O)C |
| 3498 | CC1(C)[C@@H](CC[C@]2(C)[C@@H]1CC[C@@]3(C)[C@H]2CC=C4[C@@H]5C[C@@](C)(CC[C@]5(C)CC[C@]34C)C(=O)[O-])O[C@H]6O[C@H]([C@H](O)[C@@H](O)[C@@H]6O[C@H]7O[C@H]([C@H](O)[C@@H](O)[C@@H]7O)C(=O)[O-])C(=O)[O-] |
| 3499 | C[C@@]1(CO)[C@H](CC[C@@]2(C)[C@H]1CC[C@@]3(C)[C@@H]2C=CC4=C5C[C@@](C)(CC[C@]5(C)CC[C@@]34C)C(=O)[O-])O[C@H]6O[C@H]([C@H](O)[C@@H](O)[C@@H]6O[C@H]7O[C@H]([C@H](O)[C@@H](O)[C@@H]7O)C(=O)[O-])C(=O)[O-] |
| 3500 | C[C@@]1(CC[C@]2(C)CC[C@@]3(C)C(=C2C1)C=C[C@@H]4[C@]5(C)CC[C@H](O)[C@@](C)(CO)[C@@H]5CC[C@@]34C)C(=O)[O-] |
| 3501 | C[C@@]1(CO)CC[C@@]2(C)CC[C@]3(C)C(=CC(=O)[C@H]4[C@]5(C)CC[C@H](O)C(C)(C)[C@@H]5CC[C@@]34C)[C@H]2C1 |
| 3502 | OC[C@@H]1O[C@H](Oc2ccc(cc2)[C@@H]3CC(=O)c4ccc(O)cc4O3)[C@@H](O)[C@H](O)[C@H]1O |
| 3503 | COc1c2CC[C@](C)(CO)Oc2cc3OC(=O)C(=Cc13)c4ccc(O)cc4O |
| 3504 | COC(=O)[C@]12CC[C@]3(C)[C@@H]4C[C@](C)(C[C@@H]3C1=CC(=O)[C@H]5[C@@]6(C)CC[C@H](O)C(C)(C)[C@H]6CC[C@@]25C)C(=O)O4 |
| 3505 | CC1(C)Oc2ccc([C@@H]3COc4c(C3)ccc5OC(C)(C)C=Cc45)c(O)c2C=C1 |
| 3506 | Oc1ccc2C(=O)C(=COc2c1)c3ccc4OCOc4c3O |
| 3507 | Oc1c2OCOc2ccc1C3=COc4cc([O-])ccc4C3=O |
| 3508 | CC1(C)Oc2ccc3C[C@H](COc3c2C=C1)c4ccc(O)cc4O |
| 3509 | CC1(C)[C@@H](O)CC[C@@]2(C)[C@@H]1CC[C@]3(C)[C@H]2C(=O)C=C4[C@H]5C[C@@]6(C)C[C@H](OC6=O)[C@@]5(C)CC[C@]34C |
| 3510 | CC(=CCc1c(O)cc(O)c2C(=O)C[C@H](Oc12)c3ccccc3)C |
| 3511 | CC1(C)Oc2c(C=C1)c(O)ccc2C3=Cc4ccc([O-])cc4OC3 |
| 3512 | CC1(C)Oc2c(C=C1)c([O-])ccc2C3=Cc4ccc([O-])cc4OC3 |
| 3513 | CC1(C)Oc2ccc(C3=COc4cc(O)ccc4C3=O)c(O)c2C=C1 |
| 3514 | CC1(C)Oc2ccc(C3=COc4cc([O-])ccc4C3=O)c(O)c2C=C1 |
| 3515 | COc1ccc2c3C(=O)Oc4cc(O)cc(O)c4c3oc2c1 |
| 3516 | COc1ccc2c3C(=O)Oc4cc(O)cc([O-])c4c3oc2c1 |
| 3517 | COc1cc2oc3c(C(=O)Oc4cc(O)cc(O)c34)c2cc1OC |
| 3518 | COc1cc2oc3c(C(=O)Oc4cc(O)cc([O-])c34)c2cc1OC |
| 3519 | CC(=CCc1c(O)ccc(C2=COc3cc(O)ccc3C2=O)c1O)C |
| 3520 | CC(=CCc1c(O)ccc(C2=COc3cc([O-])ccc3C2=O)c1O)C |
| 3521 | CCCC[C@H](CCC)CCC(C)C |
| 3522 | COc1ccc(cc1)C2=COc3cc(O[C@H]4O[C@@H](CO)[C@H](O)[C@H](O)[C@@H]4O[C@H]5OC[C@@](O)(CO)[C@@H]5O)ccc3C2=O |
| 3523 | CCCCCC |
| 3524 | CC1CCCCC1 |
| 3525 | CC1CCCC1 |
| 3526 | CCCCCCCCCCCCCCCCCCCCCCOC(=O)\C=C/c1ccc(O)c(O)c1 |
| 3527 | CCCC[C@H](CC)CCCC(C)C |
| 3528 | COc1ccc(cc1)C2=COc3cc(O[C@H]4O[C@@H](CO)[C@H](O)[C@@H](O)[C@@H]4O)ccc3C2=O |
| 3529 | COc1ccc2[C@H]3COc4cc(O[C@H]5O[C@@H](CO)[C@H](O)[C@@H](O)[C@@H]5O)ccc4[C@H]3Oc2c1 |
| 3530 | OC[C@H]1O[C@@H]([C@H](O)[C@@H](O)[C@@H]1O)c2c(O)cc(O)c3C(=O)C=C(Oc23)c4ccc(O)cc4 |
| 3531 | OC[C@H]1O[C@@H]([C@@H](O)[C@@H](O)[C@@H]1O)c2c(O)cc(O)c3C(=O)C=C(Oc23)c4ccc([O-])cc4 |
| 3532 | OC[C@H]1O[C@@H]([C@@H](O)[C@H](O)[C@@H]1O)c2c([O-])cc(O)c3C(=O)C=C(Oc23)c4ccc([O-])cc4 |
| 3533 | OC[C@@H]1O[C@H](Oc2ccc(cc2)[C@@H]3CC(=O)c4c(O)cc(O)cc4O3)[C@@H](O)[C@H](O)[C@H]1O |
| 3534 | CC(=CCc1c(O)ccc([C@H]2COc3c(C2)ccc4OC(C)(C)C=Cc34)c1O)C |
| 3535 | C[C@H]1O[C@@H]([C@@H](O)[C@@H](O)[C@H]1O)c2c(O)c([C@H]3O[C@H](CO)[C@@H](O)[C@@H](O)[C@H]3O)c(O)c4C(=O)C=C(Oc24)c5ccc(O)cc5 |
| 3536 | C[C@@H]1O[C@@H]([C@@H](O)[C@H](O)[C@H]1O)c2c(O)c([C@H]3O[C@@H](CO)[C@H](O)[C@@H](O)[C@@H]3O)c(O)c4C(=O)C=C(Oc24)c5ccc([O-])cc5 |
| 3537 | CC(=CCc1cc(cc(O)c1O)C2=C([O-])C(=O)c3c(O)cc(O)cc3O2)C |
| 3538 | COC1=C(Oc2cc(O)cc(O)c2C1=O)c3cc(O)c(O)c(CC=C(C)C)c3 |
| 3539 | CC1(C)[C@@H](CC[C@]2(C)[C@H]1CC[C@@]3(C)[C@H]2C(=O)C=C4[C@@H]5C[C@](C)(CC[C@]5(C)CC[C@]34C)C(=O)[O-])O[C@H]6O[C@H]([C@H](O)[C@@H](O[C@H]7O[C@H]([C@H](O)[C@@H](O)[C@@H]7O)C(=O)[O-])[C@@H]6O)C(=O)[O-] |
| 3540 | CCCC(C)C |
| 3541 | CC(=CCc1cc(cc(O)c1O)[C@@H]2CC(=O)c3c(O)cc(O)cc3O2)C |
| 3542 | COC1=C(Oc2ccc(O)c(O)c2C1=O)c3cc(O)c(O)cc3CC=C(C)C |
| 3543 | O[C@H]1CO[C@@H](OC(=O)c2ccc(O)c(O)c2)[C@H](O)[C@@H]1O |
| 3544 | O[C@H]1CO[C@H](OC(=O)c2ccc([O-])c(O)c2)[C@@H](O)[C@@H]1O |
| 3545 | OC[C@@H]1O[C@H](Oc2c(O)c(O[C@H]3OC[C@H](O)[C@@H](O)[C@@H]3O)c4OC(=CC(=O)c4c2O)c5ccc(O)cc5)[C@@H](O)[C@H](O)[C@H]1O |
| 3546 | OC[C@@H]1O[C@H](Oc2c(O)c(O[C@H]3OC[C@H](O)[C@@H](O)[C@@H]3O)c4OC(=CC(=O)c4c2O)c5ccc([O-])cc5)[C@@H](O)[C@H](O)[C@H]1O |
| 3547 | Oc1ccc(cc1)C2=CC(=O)c3c(O)c(O)c(O)c(O)c3O2 |
| 3548 | Oc1c(O)c(O)c2C(=O)C=C(Oc2c1O)c3ccc([O-])cc3 |
| 3549 | OC[C@@H]1O[C@H](Oc2ccc3C(=O)C[C@H](Oc3c2)c4ccc(O)cc4)[C@@H](O)[C@H](O)[C@H]1O |
| 3550 | CCCCCCCCC\C=C\C |
| 3551 | OC[C@H]1O[C@@H](Oc2ccc(C(=O)\C=C/c3ccc(O)cc3)c(O)c2)[C@@H](O)[C@@H](O)[C@@H]1O |
| 3552 | CCC1(O)CCC1 |
| 3553 | CC(=CCc1c(O)ccc2C(=O)C[C@H](Oc12)c3ccc(O)cc3)C |
| 3554 | CCCCCCCCCCCCC(=O)C |
| 3555 | C[C@@H]1O[C@@H]([C@@H](O)[C@H](O)[C@H]1O)c2c(O)c([C@H]3O[C@@H](CO)[C@H](O)[C@@H](O)[C@@H]3O)c4OC(=CC(=O)c4c2O)c5ccc(O)cc5 |
| 3556 | C[C@@H]1O[C@@H]([C@H](O)[C@@H](O)[C@H]1O)c2c(O)c([C@H]3O[C@@H](CO)[C@H](O)[C@H](O)[C@H]3O)c4OC(=CC(=O)c4c2O)c5ccc([O-])cc5 |
| 3557 | COc1c2CCC(C)(C)Oc2cc3OC(=O)c4c(oc5cc(O)ccc45)c13 |
| 3558 | CC(=CCc1cc(ccc1O)C2=C([O-])C(=O)c3c(O)cc(O)cc3O2)C |
| 3559 | COc1c2CCC(C)(C)Oc2cc3OC(=O)C(=Cc13)c4ccc(O)cc4O |
| 3560 | OC[C@@H]1O[C@H](Oc2ccc(\C=C/C(=O)c3ccc(O)cc3O)cc2)[C@@H](O)[C@@H](O)[C@H]1O |
| 3561 | OC[C@@H]1O[C@@H](Oc2ccc(C(=O)\C=C\c3ccc(O)cc3)c(O)c2)[C@H](O[C@H]4OC[C@@](O)(CO)[C@@H]4O)[C@@H](O)[C@@H]1O |
| 3562 | OC[C@@H]1O[C@H](Oc2ccc(cc2)[C@@H]3CC(=O)c4ccc(O)cc4O3)[C@@H](O[C@H]5OC[C@@](O)(CO)[C@@H]5O)[C@H](O)[C@H]1O |
| 3563 | CC(=CCc1cc2C(=O)C[C@H](Oc2c(CC=C(C)C)c1O)c3ccc(O)cc3)C |
| 3564 | CC1(C)[C@H](O)CC[C@@]2(C)[C@@H]1CC[C@]3(C)[C@@H]2C(=O)C=C4[C@@]3(C)CC[C@]5(C)CC[C@@]6(C)C[C@@]45OC6=O |
| 3565 | COc1ccc2C(=O)C(=COc2c1)c3ccc(O[C@H]4O[C@@H](CO)[C@H](O)[C@@H](O)[C@@H]4O)cc3 |
| 3566 | COc1ccc2C(=O)C(=COc2c1)c3ccc(O)cc3 |
| 3567 | COc1ccc2C(=O)C(=COc2c1)c3ccc([O-])cc3 |
| 3568 | OC[C@@H]1O[C@@H]([C@H](O)[C@H](O)[C@H]1O)c2c(O)c([C@@H]3OC[C@H](O)[C@@H](O)[C@H]3O)c(O)c4C(=O)C=C(Oc24)c5ccc(O)cc5 |
| 3569 | OC[C@@H]1O[C@@H]([C@H](O)[C@@H](O)[C@H]1O)c2c(O)c([C@H]3OC[C@@H](O)[C@H](O)[C@@H]3O)c(O)c4C(=O)C=C(Oc24)c5ccc([O-])cc5 |
| 3570 | COc1cc(O)cc2OC[C@H]3[C@@H](Oc4c(CC=C(C)C)c(O)ccc34)c12 |
| 3571 | CC(=O)O[C@H]1C[C@](C)(C[C@H]2C3=CC(=O)[C@@H]4[C@]5(C)CC[C@H](O)C(C)(C)[C@@H]5CC[C@@]4(C)[C@]3(C)CC[C@]12C)C(=O)[O-] |
| 3572 | COC1=C(Oc2cc(O)cc(O)c2C1=O)c3ccc(O)c(OC)c3 |
| 3573 | COC1=C(Oc2cc(O)cc(O)c2C1=O)c3ccc([O-])c(OC)c3 |
| 3574 | C[C@]12CC[C@](C)(C[C@@H]1C3=CC[C@@H]4[C@]5(C)CC[C@H](O)[C@@](C)(O)[C@@H]5CC[C@]4(C)[C@]3(C)CC2)C(=O)[O-] |
| 3575 | Oc1ccc(C(=O)\C=C/c2ccccc2)c(O)c1 |
| 3576 | Oc1cc([O-])ccc1C(=O)\C=C/c2ccccc2 |
| 3577 | CC(=CCc1c(O)ccc([C@H]2COc3c(CC=C(C)C)c(O)cc(O)c3C2=O)c1O)C |
| 3578 | COc1ccc([C@H]2COc3c(C2)ccc4OC(C)(C)C=Cc34)c(O)c1O |
| 3579 | COc1ccc([C@H]2COc3c(C2)ccc4OC(C)(C)C=Cc34)c(O)c1[O-] |
| 3580 | CCC(C)(C)CC |
| 3581 | COc1c(O)c(O)ccc1C\C=C/c2ccc(O)c(O)c2 |
| 3582 | CCc1cc(C)ccc1C |
| 3583 | COc1cc(O)c(cc1\C=C\C(=O)c2ccc(O)cc2)C(C)(C)C=C |
| 3584 | COc1cc(O)c(cc1\C=C\C(=O)c2ccc([O-])cc2)C(C)(C)C=C |
| 3585 | CCCC[C@H](C)CC |
| 3586 | CCC[C@H](C)CC |
| 3587 | CCC(C)CC |
| 3588 | CCC(CC)CC |
| 3589 | COc1c(O)ccc([C@H]2COc3c(C2)ccc4OC(C)(C)C=Cc34)c1O |
| 3590 | CC1(C)[C@H](CC[C@@]2(C)[C@@H]1CC[C@]3(C)[C@H]2C(=O)C=C4[C@H]5C[C@@]6(C)C[C@H](OC6=O)[C@@]5(C)CC[C@]34C)OC=O |
| 3591 | COc1ccc(cc1)C2=COc3cc(OC)ccc3C2=O |
| 3592 | COc1c(CC=C(C)C)c(O)cc2OC[C@H]3[C@H](Oc4cc(O)c(CC=C(C)C)cc34)c12 |
| 3593 | COc1ccc([C@H]2COc3c(C2)ccc4OC(C)(C)C=Cc34)c(O)c1 |
| 3594 | O[C@@H](Cc1ccc(O)cc1)C(=O)c2ccc(O)cc2O |
| 3595 | O[C@H](Cc1ccc(O)cc1)C(=O)c2ccc(O)cc2[O-] |
| 3596 | CC(=CCc1cc2C(=CC(=O)Oc2cc1O)c3ccc(O)cc3)C |
| 3597 | CC1(C)Oc2ccc(C(=O)\C=C\c3ccc(O)cc3)c(O)c2C=C1 |
| 3598 | CC1=C(O)C(=O)[C@@H](O)CO1 |
| 3599 | CC1=C([O-])C(=O)[C@@H](O)CO1 |
| 3600 | Cc1cc[nH+]c2CCCCc12 |
| 3601 | Cc1ccnc2CCCCc12 |
| 3602 | COc1cc2OC[C@@H](Cc2c(OC)c1CC=C(C)C)c3ccc(O)c(CC=C(C)C)c3O |
| 3603 | CCCCCCCCCCCCCC\C=C\CCCC(=O)[O-] |
| 3604 | C[C@H]1[C@H](OC(=O)C)O[C@H](Oc2ccc(cc2)[C@@H]3CC(=O)c4ccc(O)cc4O3)[C@@H](O)[C@@H]1O |
| 3605 | CC1(C)[C@H](O)CC[C@@]2(C)[C@@H]1CC[C@]3(C)[C@H]2CC=C4[C@@H]5C[C@@](C)(CC[C@@]5(C)CC[C@@]34C)C(=O)[O-] |
| 3606 | COc1c(CC=C(C)C)c(O)cc2OC[C@H]3[C@@H](Oc4cc5OC(C)(C)C=Cc5cc34)c12 |
| 3607 | CC(=CCc1c(O)cc2O[C@@H](CC(=O)c2c1O)c3ccc(O)c(O)c3)C |
| 3608 | COc1cc([O-])cc2OC(=O)C(=Cc12)c3ccc(O)cc3O |
| 3609 | CC(=O)Oc1ccc2C(=O)C(=C(C)Oc2c1)c3ccccc3 |
| 3610 | CC1=C(C(=O)c2ccc(O)cc2O1)c3ccccc3 |
| 3611 | CC1=C(C(=O)c2ccc([O-])cc2O1)c3ccccc3 |
| 3612 | CC(=CCc1c(O)cc(O)c2C(=O)C[C@H](Oc12)c3ccc(O)c(O)c3)C |
| 3613 | CC[C@H](C)CCCCCCCCCCC(=O)OC |
| 3614 | COc1c2CCC(C)(C)Oc2cc3OC[C@@H](Cc13)c4ccc(O)c(CC=C(C)C)c4O |
| 3615 | CCCCCCCCCC\C=C\CCCCCCCC(=O)[O-] |
| 3616 | CC1(C)[C@@H](CC[C@]2(C)[C@H]1CC[C@@]3(C)[C@H]2C(=O)C=C4[C@@H]5C[C@](C)(CC[C@]5(C)CC[C@]34C)C(=O)[O-])O[C@H]6O[C@H]([C@H](O)[C@@H](O)[C@@H]6O[C@H]7OC[C@H](O)[C@@H](O)[C@@H]7O)C(=O)[O-] |
| 3617 | CC(=CCC1=C(Oc2c3C=CC(C)(C)Oc3cc([O-])c2C1=O)c4cc(O)c(O)cc4O)C |
| 3618 | COc1ccc([C@@H]2COc3cc(O)ccc3C2)c(O)c1 |
| 3619 | COc1cc2OC=C(C(=O)c2c(O)c1CC=C(C)C)c3ccc(O)cc3 |
| 3620 | COc1cc2OC=C(C(=O)c2c(O)c1CC=C(C)C)c3ccc([O-])cc3 |
| 3621 | CC(=CCc1c(O)cc2OC=C(C(=O)c2c1O)c3cc(O)c4OC(C)(C)C=Cc4c3)C |
| 3622 | COc1ccc2[C@H]3COc4c(CC=C(C)C)c(O)ccc4[C@@H]3Oc2c1 |
| 3623 | COc1cc2oc(cc2c(OC)c1CC=C(C)C)c3ccc(O)cc3O |
| 3624 | CC(=CCc1cc(ccc1O)C2=C([O-])C(=O)c3c(O)c(CC=C(C)C)c(O)cc3O2)C |
| 3625 | COc1ccc([C@H]2COc3cc(OC)c(CC=C(C)C)cc3C2=O)c(O)c1 |
| 3626 | COc1cc(O)cc2OC[C@H](C(=O)c12)c3ccc4OC(C)(C)C=Cc4c3O |
| 3627 | CC1(C)Oc2ccc(cc2C[C@@H]1O)C3=C([O-])C(=O)c4c(O)cc(O)cc4O3 |
| 3628 | CC(=CCc1cc2C(=O)C=C(Oc2cc1O)c3ccc4OC(C)(C)C=Cc4c3)C |
| 3629 | CC(=CCc1cc(ccc1O)[C@H]2Oc3c4C=CC(C)(C)Oc4ccc3C(=O)[C@H]2O)C |
| 3630 | CC(=C)[C@@H]1Cc2c(O)c3C(=O)C(=COc3cc2O1)c4ccc(O)cc4 |
| 3631 | CC(=C)[C@@H]1Cc2c(O)c3C(=O)C(=COc3cc2O1)c4ccc([O-])cc4 |
| 3632 | CC1(C)[C@@H](O)CC[C@]2(C)[C@@H]1CC[C@@]3(C)[C@@H]2C(=O)C=C4[C@@]3(C)CC[C@@]5(C)CC[C@](C)(C[C@@]45O)C(=O)[O-] |
| 3633 | COc1cc(\C=C\C(=O)OC[C@@]2(O)CO[C@H](O[C@@H]3[C@@H](Oc4ccc(\C=C/C(=O)c5ccc(O)cc5O)cc4)O[C@@H](CO)[C@H](O)[C@H]3O)[C@H]2O)ccc1O |
| 3634 | COc1ccc(cc1O)C2=COc3cc(O)c(OC)cc3C2=O |
| 3635 | COc1ccc(cc1O)C2=COc3cc([O-])c(OC)cc3C2=O |
| 3636 | CC(=CCc1c(O)ccc2c1OC(=O)c3c2oc4cc(O)ccc34)C |
| 3637 | CC1(C)Oc2ccc(cc2C=C1)[C@@H]3CC(=O)c4ccc5OC(C)(C)C=Cc5c4O3 |
| 3638 | CC(=CCc1cc(ccc1O)[C@H]2CC(=O)c3ccc(O)cc3O2)C |
| 3639 | CC(=CCc1c(O)cc2OCC(=Cc2c1O)c3ccc(O)cc3O)C |
| 3640 | C[C@H]1CC[C@@]2(CC[C@]3(C)C(=CC[C@@H]4[C@@]5(C)CC[C@H](O)C(C)(C)[C@H]5CC[C@]34C)[C@H]2[C@@H]1C)C(=O)O |
| 3641 | C[C@H]1CC[C@@]2(CC[C@]3(C)C(=CC[C@@H]4[C@@]5(C)CC[C@H](O)C(C)(C)[C@H]5CC[C@]34C)[C@H]2[C@@H]1C)C(=O)[O-] |
| 3642 | OC[C@@H]1O[C@H](OC2=C(Oc3cc(O)cc(O)c3C2=O)c4ccc(O)cc4)[C@@H](O)[C@H](O)[C@H]1O |
| 3643 | OC[C@H]1O[C@@H](OC2=C(Oc3cc(O)cc(O)c3C2=O)c4ccc([O-])cc4)[C@H](O)[C@H](O)[C@H]1O |
| 3644 | CC(C)[C@@H]1CC[C@@H](C)C[C@@H]1O |
| 3645 | CC[N@H+]1C[C@]2(COC)[C@H](O)C[C@H](OC)[C@@]34[C@@H]5C[C@]6(O)[C@@H](OC)[C@H](O)[C@](OC(=O)C)([C@@H]([C@H](OC)[C@H]23)[C@@H]14)[C@H]5[C@@H]6OC(=O)c7ccccc7 |
| 3646 | CCN1C[C@]2(COC)[C@H](O)C[C@H](OC)[C@@]34[C@@H]5C[C@]6(O)[C@@H](OC)[C@H](O)[C@](OC(=O)C)([C@@H]([C@H](OC)[C@H]23)[C@@H]14)[C@H]5[C@@H]6OC(=O)c7ccccc7 |
| 3647 | Nc1cccc(O)c1 |
| 3648 | Nc1ccccc1O |
| 3649 | Nc1ccc(O)cc1 |
| 3650 | CC[N@H+]1C[C@]2(COC)CC[C@H](O)[C@@]34[C@@H]5C[C@@H]6[C@H](C[C@@](O)([C@H]5[C@H]6OC(=O)c7ccc(OC)cc7)[C@@H]([C@H](OC)[C@H]23)[C@@H]14)OC |
| 3651 | CC[N@H+]1C[C@]2(COC)CC[C@H](O)[C@@]34[C@@H]5C[C@H]6[C@H](O)[C@@H]5[C@](O)(C[C@@H]6OC)[C@@H]([C@H](OC)[C@H]23)[C@@H]14 |
| 3652 | CC[N@H+]1C[C@]2(COC)CC[C@H](O)[C@@]34[C@@H]5C[C@@H]6[C@H](C[C@@](O)([C@H]5[C@H]6OC(=O)C)[C@@H]([C@H](OC)[C@H]23)[C@@H]14)OC |
| 3653 | CC[N@H+]1C[C@]2(C)CC[C@H](O)[C@@]34[C@@H]5C[C@H]6[C@H](O)[C@@H]5[C@](O)(C[C@@H]6OC)[C@@H](C[C@H]23)[C@@H]14 |
| 3654 | CC[N@H+]1C[C@]2(COC)CC[C@H](O)[C@@]34[C@@H]5C[C@@H]6[C@H](C[C@@](O)([C@H]5[C@H]6OC(=O)\C=C\c7ccccc7)[C@@H]([C@H](OC)[C@H]23)[C@@H]14)OC |
| 3655 | CC[N@H+]1C[C@]2(COC)CC[C@H](OC)[C@@]34[C@@H]5C[C@]6(O)[C@H](C[C@](OC(=O)C)([C@@H]([C@H](OC)[C@H]23)[C@@H]14)[C@H]5[C@H]6OC(=O)c7ccc(OC)cc7)OC |
| 3656 | COC(=O)c1ccccc1NC(=O)[C@H](C)CC(=O)N |
| 3657 | CC[N@H+]1C[C@]2(COC)CC[C@H](OC)[C@@]34[C@@H]5C[C@@H]6[C@H](C[C@@]7(OCO[C@@]7([C@@H](OC)[C@H]23)[C@@H]14)[C@H]5[C@H]6O)OC |
| 3658 | CC[N@H+]1C[C@]2(COC)CC[C@H](O)[C@@]34[C@@H]5C[C@@H]6[C@H](C[C@@]7(OCO[C@@]7([C@@H](OC)[C@H]23)[C@@H]14)[C@H]5[C@H]6O)OC |
| 3659 | CC[N@H+]1C[C@]2(COC)CC[C@H](OC)[C@@]34[C@@H]5C[C@@H]6[C@H](C[C@@]7(OCO[C@@]7([C@@H](OC)[C@H]23)[C@@H]14)[C@H]5[C@H]6OC)OC |
| 3660 | CC[N@H+]1C[C@]2(COC)CC[C@H](OC)[C@@]34[C@@H]5C[C@@H]6[C@H](C[C@@](O)([C@H]5[C@H]6OC)[C@](O)([C@@H](OC)[C@H]23)[C@@H]14)OC |
| 3661 | CC[N@@H+]1C[C@]2(COC(=O)c3ccccc3NC(=O)[C@H](C)CC(=O)N)CC[C@H](OC)[C@@]45[C@@H]6C[C@@H]7[C@H](C[C@@](O)([C@H]6[C@H]7OC)[C@](O)([C@@H](OC)[C@H]24)[C@@H]15)OC |
| 3662 | CC[N@H+]1C[C@]2(COC(=O)c3ccccc3NC(=O)C[C@@H](C)C(=O)N)CC[C@H](OC)[C@@]45[C@@H]6C[C@@H]7[C@H](C[C@@](O)([C@H]6[C@H]7OC)[C@](O)([C@@H](OC)[C@H]24)[C@@H]15)OC |
| 3663 | CC[N@@H+]1C[C@]2(C)CC[C@H](OC)[C@]34[C@@H]2[C@H](OC(=O)C)[C@]5(OCO[C@@]56C[C@H](OC)[C@H]7C[C@]3(O)[C@@H]6[C@H]7OC)[C@@H]14 |
| 3664 | CC[N@H+]1C[C@]2(C)CC[C@H](OC)[C@]34[C@@H]2[C@H](O)[C@@]5(OCO[C@@]56C[C@H](OC)[C@H]7C[C@]3(O)[C@@H]6[C@H]7OC)[C@@H]14 |
| 3665 | Oc1ccc(C[C@H]2[NH2+]CCc3cc(O)c(O)cc23)cc1 |
| 3666 | Oc1ccc(C[C@H]2NCCc3cc(O)c(O)cc23)cc1 |
| 3667 | Oc1ccc(C[C@H]2NCCc3cc(O)c([O-])cc23)cc1 |
| 3668 | CC[N@H+]1C[C@]2(COC(=O)c3ccccc3NC(=O)[C@H](C)CC(=O)[O-])CC[C@H](OC)[C@@]45[C@@H]6C[C@@H]7[C@H](C[C@@](O)([C@H]6[C@H]7OC)[C@](O)([C@@H](OC)[C@H]24)[C@@H]15)OC |
| 3669 | CC[N@H+]1C[C@]2(COC(=O)c3ccccc3NC(=O)C[C@@H](C)C(=O)[O-])CC[C@H](OC)[C@@]45[C@@H]6C[C@@H]7[C@H](C[C@@](O)([C@H]6[C@H]7OC)[C@](O)([C@@H](OC)[C@H]24)[C@@H]15)OC |
| 3670 | CC[N@H+]1C[C@]2(C)CCC[C@@]34[C@@H]2C[C@@H]([C@@H]13)[C@@]56CC[C@@H]([C@H](O)[C@@H]45)C(=C)[C@H]6O |
| 3671 | CC[N@H+]1C[C@]2(COC)CC[C@H](OC)[C@@]34[C@@H]5C[C@]6(O)[C@@H](OC)[C@H](O)[C@@](OC(=O)C)([C@H]5[C@H]6OC(=O)c7ccccc7)[C@@H]([C@H](OC)[C@H]23)[C@@H]14 |
| 3672 | CC[N@H+]1C[C@@H]2CC[C@H](OC)[C@]34[C@@H]2C[C@H]([C@H]5C[C@H](OC)[C@H]6C[C@@H]3[C@@H]5C6=O)[C@@H]14 |
| 3673 | C[N@H+]1C[C@]2(C)CCC[C@@]34[C@@H]2C[C@@H]([C@@H]13)[C@@]56CC[C@@H](C[C@H]45)[C@@](O)(CO)[C@H]6O |
| 3674 | OC[C@H](O)[C@@H]1O[C@H](O[C@@H]2[C@H](O)[C@@H](OC(CO)CO)O[C@H]2[C@H](O)CO)[C@@H](O)[C@@H]1O |
| 3675 | COC[C@@]12CC[C@H](OC)[C@@]34[C@@H]5C[C@]6(O)[C@@H](OC)[C@H](O)[C@@](OC(=O)C)([C@H]5[C@H]6OC(=O)c7ccccc7)[C@@H]([C@H](OC)[C@H]13)[C@H]4[N@@H+](C)C2 |
| 3676 | COC[C@@]12CC[C@H](OC)[C@@]34[C@@H]5C[C@@H]6[C@@H](OC)[C@H](O)[C@@](OC(=O)C)([C@H]5[C@H]6OC(=O)c7ccccc7)[C@@H]([C@H](OC)[C@H]13)[C@H]4[N@@H+](C)C2 |
| 3677 | CC[N@H+]1C[C@]2(COC)CC[C@H](O)[C@@]34[C@@H]5C[C@H]6[C@H](O)[C@@H]5[C@](O)(C[C@@H]6OC)[C@@H](C[C@H]23)[C@@H]14 |
| 3678 | CCCCCCCCCCCCCCCC(=O)O[C@]12C[C@H](OC)[C@@]3(O)C[C@H]([C@@H]1[C@H]3OC(=O)c4ccc(OC)cc4)[C@@]56[C@H](CC[C@@]7(COC)C[N@H+](CC)[C@@H]5[C@@H]2[C@H](OC)[C@@H]67)OC |
| 3679 | CCCCCCCCCCCCCCCC(=O)O[C@]12[C@@H](O)[C@H](OC)[C@@]3(O)C[C@H]([C@@H]1[C@H]3OC(=O)c4ccccc4)[C@@]56[C@H](CC[C@@]7(COC)C[N@H+](C)[C@@H]5[C@@H]2[C@H](OC)[C@@H]67)OC |
| 3680 | COC[C@]12C[N@H+](C)[C@@H]3[C@@H]4[C@H](OC)[C@H]1[C@@]3([C@H](C[C@H]2O)OC)[C@@H]5C[C@]6(O)[C@@H](OC)[C@H](O)[C@@]4(OC(=O)C)[C@H]5[C@H]6OC(=O)c7ccccc7 |
| 3681 | CC[N@H+]1C[C@]2(COC)CC[C@H](OC)[C@@]34[C@@H]5C[C@]6(O)[C@@H](OC)[C@H](O)[C@@](OC)([C@H]5[C@H]6OC(=O)c7ccccc7)[C@@H]([C@H](OC)[C@H]23)[C@@H]14 |
| 3682 | CC[N@H+]1C[C@]2(COC)CC[C@@H](O)[C@@]34[C@@H]5C[C@H]6[C@H](O)[C@@H]5[C@](O)([C@@H](O)[C@@H]6OC)[C@@H]([C@H](OC)[C@H]23)[C@@H]14 |
| 3683 | CC[N@H+]1C[C@]2(COC)CC[C@H](OC)[C@@]34[C@@H]5C[C@H]6[C@H](O)[C@@H]5[C@](O)(C[C@@H]6OC)[C@@H](C[C@H]23)[C@@H]14 |
| 3684 | CC[N@H+]1C[C@]2(COC)CC[C@H](O)[C@@]34[C@@H]5C[C@@H]6[C@H](C[C@@](O)([C@H]5[C@H]6OC(=O)c7ccc(OC)c(OC)c7)[C@@H]([C@H](OC)[C@H]23)[C@@H]14)OC |
| 3685 | CC[N@H+]1C[C@]2(COC)CC[C@H](OC)[C@@]34[C@@H]5C[C@@H]6[C@H](C[C@@](OC(=O)C)([C@H]5[C@H]6OC(=O)c7ccc(OC)cc7)[C@@H]([C@H](OC)[C@H]23)[C@@H]14)OC |
| 3686 | C[C@@]1(CO)[C@H](O)CC[C@]2(C)[C@@H]1CCC(=C)[C@H]2\C=C/[C@@H]3C=COC3=O |
| 3687 | CC[N@H+]1C[C@]2(COC)CC[C@H](OC)[C@@]34[C@@H]5C[C@@H]6[C@H](C[C@@]7(OCO[C@]7([C@@H](O)[C@H]23)[C@@H]14)[C@H]5[C@@H]6OC)OC |
| 3688 | CC[N@@H+]1C[C@]2(COC)CC[C@@H](O)[C@@]34[C@H]5C[C@@H]6[C@@H](O)[C@H]5[C@](O)(C[C@@H]6OC)[C@@](O)([C@@H](OC)[C@H]23)[C@@H]14 |
| 3689 | OC[C@H]1O[C@@H](Oc2cc3cc(O[C@@H]4O[C@H](CO)[C@@H](O)[C@H](O)[C@H]4O)c([o+]c3cc2O)c5cc(O)c(O)c(O)c5)[C@H](O)[C@@H](O)[C@@H]1O |
| 3690 | OC[C@H]1O[C@@H](Oc2cc3cc(O[C@@H]4O[C@H](CO)[C@@H](O)[C@H](O)[C@H]4O)c([o+]c3cc2O)c5cc(O)c(O)c([O-])c5)[C@H](O)[C@@H](O)[C@@H]1O |
| 3691 | Oc1cc2cc(O)c([o+]c2cc1O)c3cc(O)c(O)c(O)c3 |
| 3692 | Oc1cc(cc(O)c1O)c2[o+]c3cc(O)c([O-])cc3cc2O |
| 3693 | Oc1cc(cc([O-])c1O)c2[o+]c3cc(O)c([O-])cc3cc2O |
| 3694 | CC[N@H+]1C[C@]2(COC(=O)c3ccccc3NC(=O)C[C@H](C)C(=O)N)CC[C@H](OC)[C@@]45[C@@H]6C[C@@H]7[C@@H](C[C@@](O)([C@H]6[C@H]7OC)[C@@](O)([C@@H](OC)[C@H]24)[C@@H]15)OC |
| 3695 | CC[N@H+]1C[C@]2(COC)CC[C@H](O)[C@@]34[C@@H]5C[C@@H]6[C@@H](C[C@@](O)([C@H]5[C@H]6OC)[C@@](O)([C@@H](OC)[C@H]23)[C@@H]14)OC |
| 3696 | CC[N@H+]1C[C@]2(C)CC[C@H](OC)[C@]34[C@@H]2[C@H](O)[C@@]5(OCO[C@@]56C[C@@H](OC)[C@H]7C[C@]3(O)[C@@H]6[C@H]7OC)[C@@H]14 |
| 3697 | CC(=O)\C(=C/OC(C)(C)[C@@H]1Cc2cc3C=CC(=O)Oc3cc2O1)\C |
| 3698 | CC[N@@H+]1C[C@]2(COC(=O)c3ccccc3NC(=O)[C@@H](C)CC(=O)[O-])CC[C@H](OC)[C@@]45[C@@H]6C[C@@H]7[C@@H](C[C@@](O)([C@H]6[C@H]7OC)[C@@](O)([C@@H](OC)[C@H]24)[C@@H]15)OC |
| 3699 | CC[N@@H+]1C[C@]2(COC(=O)c3ccccc3NC(=O)C[C@H](C)C(=O)[O-])CC[C@H](OC)[C@@]45[C@@H]6C[C@@H]7[C@@H](C[C@@](O)([C@H]6[C@H]7OC)[C@@](O)([C@@H](OC)[C@H]24)[C@@H]15)OC |
| 3700 | C[C@@]1(CO)[C@H](O)CC[C@@]2(C)[C@H](CCC3=CCOC3=O)C(=C)CC[C@@H]12 |
| 3701 | CC[N@@H+]1C[C@@]2(COC)CC[C@@H](OC)[C@]34[C@H]5C[C@H]6[C@@H](C[C@@](O)([C@H](C[C@@H]23)[C@H]14)[C@@H]5[C@@H]6OC(=O)C)OC |
| 3702 | CC[N@H+]1C[C@]2(C)CC[C@H](O)[C@@]34[C@@H]5C[C@H]6[C@H](O)[C@@H]5[C@](O)(C[C@@H]6OC)[C@@H](C[C@H]23)[C@@H]14 |
| 3703 | COC1=C(Oc2c(ccc3occc23)C1=O)c4ccccc4 |
| 3704 | COc1cc(CC[NH3+])cc(OC)c1OC |
| 3705 | CC[N@H+]1C[C@]2(COC)CC[C@@H](OC)[C@@]34[C@@H]5C[C@@]6(O)[C@@H](OC)[C@H](O)[C@@](OC)([C@@H]5[C@H]6OC(=O)c7ccccc7)[C@@H]([C@H](OC)[C@H]23)[C@@H]14 |
| 3706 | C[C@H](CC\C=C(\C)/C(=O)[O-])[C@@H]1CC[C@]2(C)[C@@H](CC3=C2CC[C@@H]4C(C)(C)C(=O)CC[C@]34C)C1=C |
| 3707 | CC[N@@H+]1C[C@@]2(COC)CC[C@@H](O)[C@]34[C@H]5C[C@H]6[C@@H](O)[C@H]5[C@@](O)(C[C@H]6OC)[C@H]([C@H](O)[C@@H]23)[C@H]14 |
| 3708 | CC[N@@H+]1C[C@@]2(COC)CC[C@@H](O)[C@]34[C@H]5C[C@@H]6[C@@H](O)[C@H]5[C@@](O)([C@H](O)[C@H]6OC)[C@H](C[C@@H]23)[C@H]14 |
| 3709 | CC[N@@H+]1C[C@]2(COC)CC[C@@H](O)[C@]34[C@@H]5C[C@H]6[C@H](O)[C@@H]5[C@](O)([C@@H](O)[C@@H]6OC)[C@H]([C@@H](OC)[C@@H]23)[C@H]14 |
| 3710 | CCCCCCCCCCCCCCCCCCCCCCCc1cc(O)cc(OC)c1 |
| 3711 | CC(=O)O[C@H]1C[C@]2([C@@H](OC(=O)c3ccccc3)[C@H]4[C@@]5(CO[C@@H]5C[C@H](OC(=O)c6ccccc6)[C@@]4(C)[C@@H](OC(=O)C)[C@H](OC(=O)C)C2=C1C)OC(=O)C)C(C)(C)O |
| 3712 | COC[C@]12C[N@@H+](C)[C@@H]3[C@@H]4[C@H](OC)[C@H]1[C@@]3([C@H](C[C@H]2O)OC)[C@]5(O)C[C@]6(O)[C@H](OC)[C@H](O)[C@@]4(OC(=O)C)[C@H]5[C@H]6OC(=O)c7ccccc7 |
| 3713 | CC[N@H+]1C[C@]2(COC)[C@@H](O)C[C@@H](OC)[C@@]34[C@@H]5C[C@]6(O)[C@H](OC)[C@H](O)[C@@](O)([C@H]5[C@H]6OC(=O)c7ccccc7)[C@@H]([C@H](OC)[C@H]23)[C@@H]14 |
| 3714 | COC[C@]12CC[C@@H](OC)[C@]34[C@H]5C[C@]6(O)[C@H](OC)[C@@H](O)[C@@](O)([C@H]5[C@H]6OC(=O)c7ccccc7)[C@H]([C@H](OC)[C@@H]13)[C@@H]4[N@H+](C)C2 |
| 3715 | CC[N@H+]1C[C@]2(COC)CC[C@H](OC)[C@@]34[C@@H]5C[C@H]6[C@H](O)[C@@H]5[C@](O)(C[C@@H]6OC)[C@@H]([C@H](OC)[C@H]23)[C@@H]14 |
| 3716 | C[N+](C)(C)CCc1ccc(O)c(O)c1 |
| 3717 | CC[N@H+]1C[C@]2(COC)CC[C@H](O)[C@@]34[C@@H]5C[C@@H]6[C@@H](C[C@@](O)([C@H]5[C@H]6OC)[C@@](O)([C@@H](O)[C@H]23)[C@@H]14)OC |
| 3718 | COc1cc2CC[N+](C)(C)[C@@H]3Cc4ccc(O)c(O)c4c(c1OC)c23 |
| 3719 | C[C@@]12C[N@@H+]3[C@H]4C[C@@]56[C@@H]7C[C@@H](C[C@@]5(O)[C@@](C[C@@H](OC(=O)c8ccccc8)[C@H]1O)([C@H]37)[C@@H]24)C(=C)[C@H]6O |
| 3720 | CC[N@H+]1C[C@]2(COC)[C@H](O)C[C@H](OC)[C@@]34[C@@H]5C[C@]6(O)[C@@H](OC)[C@@H](O)[C@@](OC(=O)C)([C@H]5[C@@H]6OC(=O)c7ccc(OC)cc7)[C@@H]([C@@H](OC)[C@H]23)[C@@H]14 |
| 3721 | CCN1C[C@]2(COC)[C@H](O)C[C@H](OC)[C@@]34[C@@H]5C[C@]6(O)[C@@H](OC)[C@@H](O)[C@@](OC(=O)C)([C@H]5[C@@H]6OC(=O)c7ccc(OC)cc7)[C@@H]([C@@H](OC)[C@H]23)[C@@H]14 |
| 3722 | CC[N@@H+]1C[C@@]2(COC)CC[C@@H](O)[C@]34[C@H]5C[C@H]6[C@@H](O)[C@H]5[C@@](O)(C[C@H]6OC)[C@H]([C@H](OC)[C@@H]23)[C@H]14 |
| 3723 | OCc1ccc(C=O)n1CCc2ccc(O)cc2 |
| 3724 | C[C@@H]1[NH2+]CCc2cc(O)c(O)cc12 |
| 3725 | C[C@@H]1[NH2+]CCc2cc(O)c([O-])cc12 |
| 3726 | CC[N@@H+]1C[C@@]2(COC)CC[C@H](OC)[C@@]34[C@@H]5C[C@H]6[C@H](O)[C@@H]5[C@](O)(C[C@@H]6OC)[C@@H](C[C@H]23)[C@@H]14 |
| 3727 | C[C@@H](CC\C=C(/C)\CO[C@@H]1O[C@H](CO[C@@H]2O[C@H](CO)[C@@H](O)[C@H](O)[C@H]2O)[C@@H](O)[C@H](O)[C@H]1O)[C@H]3CC[C@@]4(C)[C@@H]5CC=C6[C@@H](CC[C@H](O)C6(C)C)[C@]5(C)C(=O)C[C@]34C |
| 3728 | C[C@H](CC\C=C(/C)\CO)[C@H]1CC[C@@]2(C)[C@@H]3CC=C4[C@@H](CC[C@@H](O)C4(C)C)[C@]3(C)C(=O)C[C@]12C |
| 3729 | CC[N@H+]1C[C@]2(COC)[C@H](O)C[C@H](OC)[C@@]34[C@@H]5C[C@@]6(O)[C@H](O)[C@@H]5[C@](O)([C@@H](O)[C@@H]6OC)[C@@H]([C@H](OC)[C@H]23)[C@@H]14 |
| 3730 | CC[N@H+]1C[C@@H]2CC[C@H](OC)[C@@]34[C@@H]2C[C@@H]([C@@H]13)[C@@]5(O)C[C@H](OC)[C@@]6(O)C[C@H]4[C@@H]5[C@H]6OC(=O)c7ccccc7 |
| 3731 | CC[N@H+]1C[C@]2(COC)CC[C@H](OC)[C@@]34[C@@H]5C[C@@H]6[C@@H](C[C@@]7(OCO[C@]7([C@@H](OC)[C@H]23)[C@@H]14)[C@H]5[C@H]6O)OC |
| 3732 | CC[N@H+]1C[C@]2(COC)CC[C@H](OC)[C@@]34[C@@H]5C[C@@H]6[C@@H](C[C@@]7(OCO[C@]7([C@@H](OC)[C@H]23)[C@@H]14)[C@H]5[C@H]6OC)OC |
| 3733 | COC1=CC(=O)[C@]2([C@@H](Cl)C[C@]34[NH2+]CC[C@]23CC(=O)C(=C4OC)OC)[C@@H]1O |
| 3734 | COC1=CC(=O)[C@]2([C@@H](Cl)C[C@]34[N@@H+](C)CC[C@]23CC(=O)C(=C4OC)OC)[C@@H]1O |
| 3735 | COC1=CC(=O)[C@]2([C@@H](Cl)C[C@]34N(C)CC[C@]23CC(=O)C(=C4OC)OC)[C@@H]1O |
| 3736 | COC1=CC(=O)[C@@]2(C1)[C@@H](Cl)C[C@]34[N@H+](C)CC[C@]23CC(=O)C(=C4OC)OC |
| 3737 | NC(=O)N[C@@H]1NC(=O)NC1=O |
| 3738 | COc1cc2CC[N@@H+]3Cc4c(C[C@H]3c2cc1O)ccc5OCOc45 |
| 3739 | COc1cc2CCN3Cc4c(C[C@H]3c2cc1O)ccc5OCOc45 |
| 3740 | COc1cc2CCNC(=O)c2cc1OC |
| 3741 | COc1cc2CC[NH+]=C(O)c2cc1OC |
| 3742 | COc1cc2CCN=C(O)c2cc1OC |
| 3743 | Oc1ccc(cc1)C2=COc3cc(O)ccc3C2=O |
| 3744 | Oc1ccc(cc1)C2=COc3cc([O-])ccc3C2=O |
| 3745 | [O-]c1ccc(cc1)C2=COc3cc([O-])ccc3C2=O |
| 3746 | OC[C@H]1O[C@@H](Oc2ccc(cc2)C3=COc4cc(O[C@@H]5O[C@H](CO)[C@@H](O)[C@H](O)[C@H]5O)ccc4C3=O)[C@H](O)[C@@H](O)[C@@H]1O |
| 3747 | OC[C@H]1O[C@@H](Oc2ccc3C(=O)C(=COc3c2)c4ccc(O)cc4)[C@H](O)[C@@H](O)[C@@H]1O |
| 3748 | OC[C@H]1O[C@@H](Oc2ccc3C(=O)C(=COc3c2)c4ccc([O-])cc4)[C@H](O)[C@@H](O)[C@@H]1O |
| 3749 | COc1cc2CCN(C)[C@H](Cc3ccc(Oc4cc(C[C@H]5N(C)CCc6cc(OC)c(OC)cc56)ccc4O)cc3)c2cc1OC |
| 3750 | COc1cc2[C@@H](Cc3ccc(O)c(Oc4ccc(C[C@H]5N(C)CCc6cc(OC)c(OC)cc56)cc4)c3)N(C)CCc2cc1O |
| 3751 | COc1cc2[C@@H](Cc3ccc(Oc4cc(C[C@H]5N(C)CCc6cc(O)c(OC)cc56)ccc4O)cc3)N(C)CCc2cc1O |
| 3752 | COc1cc2[C@@H](Cc3ccc(Oc4cc(C[C@H]5N(C)CCc6cc(OC)c(OC)cc56)ccc4O)cc3)N(C)CCc2cc1O |
| 3753 | COc1ccc2c(c1)C(=O)c3c(NCCc4ccc(O)cc4)c(OC)cc5cc[nH+]c2c35 |
| 3754 | COc1ccc2c(c1)C(=O)c3c(NCCc4ccc(O)cc4)c(OC)cc5ccnc2c35 |
| 3755 | COc1ccc2c(c1)C(=O)c3c(N)c(OC)c(OC)c4cc[nH+]c2c34 |
| 3756 | CNc1c(OC)cc2cc[nH+]c3c4ccc(OC)cc4C(=O)c1c23 |
| 3757 | CNc1c(OC)cc2ccnc3c4ccc(OC)cc4C(=O)c1c23 |
| 3758 | COc1cc2cc[nH+]c3c4ccc([O-])cc4C(=O)c(c1OC)c23 |
| 3759 | COc1cc2ccnc3c4ccc([O-])cc4C(=O)c(c1OC)c23 |
| 3760 | COc1cc2CCN(C)[C@H](Cc3ccc(O)c(Oc4ccc(C[C@H]5N(C)CCc6cc(OC)c(OC)cc56)cc4)c3)c2cc1O |
| 3761 | COC1=CC(=O)[C@]2(CC[C@]34[N@@H+](C)CC[C@]23CC(=O)C(=C4OC)OC)[C@H]1O |
| 3762 | COC1=CC(=O)[C@]2(CC[C@]34N(C)CC[C@]23CC(=O)C(=C4OC)OC)[C@H]1O |
| 3763 | COc1cc2C=CNC(=O)c2cc1O |
| 3764 | COc1cc2C=CNC(=O)c2cc1[O-] |
| 3765 | CC(=O)OC[C@H]1O[C@H]([C@H](O)[C@@H](O)[C@@H]1O)c2c(O)ccc3C(=O)C[C@H](Oc23)c4ccc(OC(=O)C)cc4 |
| 3766 | COc1ccc2C3=[NH+]CCc4c(OC)c(OC)c(OC)c(C(=O)c2c1)c34 |
| 3767 | COc1cc2C(=O)N(C)C(=O)C(=O)c2cc1OC |
| 3768 | COc1cc2C(=O)N(C)C(=O)c2cc1OC |
| 3769 | COc1cc2C=CN(C)C(=O)c2cc1O |
| 3770 | COc1cc2C=CN(C)C(=O)c2cc1[O-] |
| 3771 | COc1ccc(cc1)C2=COc3cc(O[C@@H]4O[C@H](CO)[C@@H](O)[C@H](O)[C@H]4O)ccc3C2=O |
| 3772 | OC[C@H]1O[C@@H](Oc2cc(O)c3C(=O)C(=COc3c2)c4ccc(O)cc4)[C@H](O)[C@@H](O)[C@@H]1O |
| 3773 | OC[C@H]1O[C@@H](Oc2cc(O)c3C(=O)C(=COc3c2)c4ccc([O-])cc4)[C@H](O)[C@@H](O)[C@@H]1O |
| 3774 | COc1cc2ccnc(C(=O)c3ccc(O)cc3)c2cc1O |
| 3775 | COc1cc2C(=O)N(C)C(=O)c2cc1O |
| 3776 | COc1cc2C(=O)N(C)C(=O)c2cc1[O-] |
| 3777 | C[C@@H]1O[C@@H](O[C@@H]2[C@@H](O)[C@@H](O)[C@@H](CO)O[C@H]2O[C@@H]3[C@@H](O)[C@H](O)[C@H](O[C@H]3O[C@H]4CC[C@@]5(C)[C@@H](CC[C@]6(C)[C@@H]5CC=C7[C@@H]8CC(C)(C)C[C@@H](O)[C@]8(C)CC[C@@]67C)C4(C)C)C(=O)[O-])[C@H](O)[C@H](O)[C@H]1O |
| 3778 | COC(=O)[C@@]1(C)C[C@H]2C3=CC[C@@H]4[C@@]5(C)CC[C@H](O)[C@](C)(CO)[C@H]5CC[C@@]4(C)[C@]3(C)CC[C@@]2(C)[C@H](O)[C@@H]1O |
| 3779 | CC1(C)C[C@H]2C3=CC[C@@H]4[C@@]5(C)CC[C@H](O)[C@](C)(CO)[C@@H]5CC[C@@]4(C)[C@]3(C)CC[C@@]2(C)C[C@@H]1O |
| 3780 | O[C@H]1[C@H](O)[C@@H](COC(=O)CC(=O)[O-])O[C@@H](Oc2ccc3C(=O)[C@H](COc3c2)c4ccc(O)cc4)[C@@H]1O |
| 3781 | O[C@H]1[C@H](O)[C@@H](COC(=O)CC(=O)[O-])O[C@@H](Oc2ccc3C(=O)[C@@H](COc3c2)c4ccc([O-])cc4)[C@@H]1O |
| 3782 | COc1ccc2C[C@H]3c4c(CC[N+]3(C)C)cc(OC)c(OC)c4c2c1O |
| 3783 | COc1ccc2c(c1)C(=O)c3c(OC)c(OC)cc4cc[nH+]c2c34 |
| 3784 | COc1cc(ccc1O)C2=COc3c(ccc(O)c3[C@@H]4O[C@H](CO)[C@@H](O)[C@H](O)[C@H]4O)C2=O |
| 3785 | COc1cc(ccc1[O-])C2=COc3c(ccc(O)c3[C@@H]4O[C@H](CO)[C@@H](O)[C@H](O)[C@H]4O)C2=O |
| 3786 | COc1cc(ccc1[O-])C2=COc3c(ccc([O-])c3[C@@H]4O[C@H](CO)[C@@H](O)[C@H](O)[C@H]4O)C2=O |
| 3787 | COc1cc2CCN(C)C(=O)c2cc1OC |
| 3788 | C[C@@H]1NC(=O)NC1=O |
| 3789 | C[C@@H]1NC(=O)[N-]C1=O |
| 3790 | COc1ccc(cc1)C2=COc3c(ccc(O)c3[C@@H]4O[C@H](CO)[C@@H](O)[C@H](O)[C@H]4O)C2=O |
| 3791 | COc1ccc(cc1)C2=COc3c(ccc([O-])c3[C@@H]4O[C@H](CO)[C@@H](O)[C@H](O)[C@H]4O)C2=O |
| 3792 | COc1cc2CCNC(=O)c2cc1O |
| 3793 | COc1cc2CC[NH+]=C(O)c2cc1O |
| 3794 | COc1cc2CCN=C(O)c2cc1O |
| 3795 | OC[C@H]1O[C@@H](Oc2ccc(cc2)C3=COc4c([C@@H]5O[C@H](CO)[C@@H](O)[C@H](O)[C@H]5O)c(O)ccc4C3=O)[C@H](O)[C@@H](O)[C@@H]1O |
| 3796 | OC[C@H]1O[C@@H](Oc2ccc(cc2)C3=COc4c([C@@H]5O[C@H](CO)[C@@H](O)[C@H](O)[C@H]5O)c([O-])ccc4C3=O)[C@H](O)[C@@H](O)[C@@H]1O |
| 3797 | OC[C@H]1O[C@H]([C@H](O)[C@@H](O)[C@@H]1O)c2c(O[C@@H]3OC[C@@H](O)[C@H](O)[C@H]3O)ccc4C(=O)C(=COc24)c5ccc(O)cc5 |
| 3798 | OC[C@H]1O[C@H]([C@H](O)[C@@H](O)[C@@H]1O)c2c(O[C@@H]3OC[C@@H](O)[C@H](O)[C@H]3O)ccc4C(=O)C(=COc24)c5ccc([O-])cc5 |
| 3799 | O[C@@H]1CO[C@@H](OC[C@H]2O[C@H]([C@H](O)[C@@H](O)[C@@H]2O)c3c(O)ccc4C(=O)C(=COc34)c5ccc(O)cc5)[C@H](O)[C@H]1O |
| 3800 | O[C@@H]1CO[C@@H](OC[C@H]2O[C@H]([C@H](O)[C@@H](O)[C@@H]2O)c3c(O)ccc4C(=O)C(=COc34)c5ccc([O-])cc5)[C@H](O)[C@H]1O |
| 3801 | O[C@@H]1CO[C@@H](OC[C@H]2O[C@H]([C@H](O)[C@@H](O)[C@@H]2O)c3c([O-])ccc4C(=O)C(=COc34)c5ccc([O-])cc5)[C@H](O)[C@H]1O |
| 3802 | CC(=CCC\C(=C\Cc1cc2c(OC(=O)c3c2oc4cc(O)ccc34)cc1O)\C)C |
| 3803 | C[C@@H]1O[C@@H](OC[C@H]2O[C@@H](Oc3cc([O-])ccc3C4=CC(=O)O[C@H]4Cc5ccc(O)cc5)[C@H](O)[C@@H](O)[C@@H]2O)[C@H](O)[C@H](O)[C@H]1O |
| 3804 | COc1ccc(C2=CC(=O)O[C@H]2Cc3ccc(O[C@@H]4O[C@H](CO)[C@@H](O)[C@H](O)[C@H]4O)cc3)c(O[C@@H]5O[C@H](CO)[C@@H](O)[C@H](O)[C@H]5O)c1 |
| 3805 | C[C@@H]1O[C@@H](OC[C@H]2O[C@@H](OC3=C(Oc4cc(O[C@@H]5O[C@@H](C)[C@H](O)[C@@H](O)[C@H]5O)cc(O)c4C3=O)c6ccc(O)cc6)[C@H](O)[C@@H](O)[C@H]2O)[C@H](O)[C@H](O)[C@H]1O |
| 3806 | C[C@@H]1O[C@@H](OC[C@H]2O[C@@H](OC3=C(Oc4cc(O[C@@H]5O[C@@H](C)[C@H](O)[C@@H](O)[C@H]5O)cc(O)c4C3=O)c6ccc([O-])cc6)[C@H](O)[C@@H](O)[C@H]2O)[C@H](O)[C@H](O)[C@H]1O |
| 3807 | COC1=C[C@@H]2[C@@H]3Cc4ccc(OC)c(O)c4[C@]2(CC[N@@H+]3C)CC1=O |
| 3808 | COC1=C[C@@H]2[C@@H]3Cc4ccc(OC)c(O)c4[C@]2(CCN3C)CC1=O |
| 3809 | COC1=C[C@@H]2[C@@H]3Cc4ccc(OC)c([O-])c4[C@]2(CCN3C)CC1=O |
| 3810 | CC1(C)C[C@@H](O)[C@]2(C)CC[C@]3(C)C(=CC[C@H]4[C@@]5(C)CC[C@H](O)C(C)(C)[C@H]5CC[C@@]34C)[C@@H]2C1 |
| 3811 | COc1cc2CCN3Cc4c(C[C@H]3c2cc1O)ccc(O)c4OC |
| 3812 | COc1ccc2C[C@@H]3[N@@H+](C)CCc4cc(OC)c(OC)c(Oc5cc6[C@H](Cc7ccc(Oc1c2)cc7)N(C)CCc6cc5OC)c34 |
| 3813 | COc1ccc2C[C@@H]3N(C)CCc4cc(OC)c(OC)c(Oc5cc6[C@H](Cc7ccc(Oc1c2)cc7)N(C)CCc6cc5OC)c34 |
| 3814 | COc1cc2CCN(C)C(=O)c2cc1O |
| 3815 | COc1cc2C(=O)C(=COc2cc1O[C@@H]3O[C@H](CO[C@@H]4OC[C@@H](O)[C@H](O)[C@H]4O)[C@@H](O)[C@H](O)[C@H]3O)c5ccc(O)cc5 |
| 3816 | COc1cc2C(=O)C(=COc2cc1O[C@@H]3O[C@H](CO[C@@H]4OC[C@@H](O)[C@H](O)[C@H]4O)[C@@H](O)[C@H](O)[C@H]3O)c5ccc([O-])cc5 |
| 3817 | COc1c(O)c2C(=O)C(=COc2cc1O[C@@H]3O[C@H](CO[C@@H]4OC[C@@H](O)[C@H](O)[C@H]4O)[C@@H](O)[C@H](O)[C@H]3O)c5ccc(O)cc5 |
| 3818 | COc1c(O)c2C(=O)C(=COc2cc1O[C@@H]3O[C@H](CO[C@@H]4OC[C@@H](O)[C@H](O)[C@H]4O)[C@@H](O)[C@H](O)[C@H]3O)c5ccc([O-])cc5 |
| 3819 | CC1(C)Oc2cc3O[C@@H]4c5ccc(O)cc5OC[C@]4(O)c3cc2C=C1 |
| 3820 | COc1ccc2c(c1)C(=O)c3c(NCCc4ccc(O)cc4)c(OC)c(OC)c5cc[nH+]c2c35 |
| 3821 | COc1ccc2c(c1)C(=O)c3c(NCCc4ccc(O)cc4)c(OC)c(OC)c5ccnc2c35 |
| 3822 | CC12CCC(CC1)C(C)(C)O2 |
| 3823 | CC(=CCC\C(=C\C=O)\C)C |
| 3824 | CC(=CCC\C(=C\C=O)\C)C |
| 3825 | COc1cc(CC[C@H](C[C@H](CCc2cc(OC)c(O)c(OC)c2)OC(=O)C)OC(=O)C)ccc1O |
| 3826 | CC1(C)CCC[C@]2(C)[C@@H](C\C=C(/CC=O)\C=O)[C@@]3(CC[C@H]12)CO3 |
| 3827 | CCCCC\C(=C\C(=O)CCc1ccc(O)c(OC)c1)\O |
| 3828 | CCCCC\C(=C\C(=O)CCc1ccc(O)c(OC)c1)\[O-] |
| 3829 | CCCCCC(=O)CC(=O)CCc1ccc(O)c(OC)c1 |
| 3830 | CCCCCCCCC\C(=C\C(=O)CCc1ccc(O)c(OC)c1)\O |
| 3831 | CCCCCCCCC\C(=C\C(=O)CCc1ccc(O)c(OC)c1)\[O-] |
| 3832 | COc1cc(CC\C=C\C(=O)CCc2ccc(O)c(OC)c2)ccc1O |
| 3833 | COc1cc(CCC(=O)\C=C\CCc2cc(OC)c(O)c(OC)c2)ccc1O |
| 3834 | COc1cc(CCC(=O)\C=C\CCc2ccc(O)cc2)ccc1O |
| 3835 | CCC[C@H](O)CC(=O)CCc1ccc(O)c(OC)c1 |
| 3836 | CCCCC[C@H](O)CC(=O)CCc1ccc(O)c(OC)c1 |
| 3837 | CCCCCCC[C@H](O)CC(=O)CCc1ccc(O)c(OC)c1 |
| 3838 | CCCCCCCCC[C@H](O)CC(=O)CCc1ccc(O)c(OC)c1 |
| 3839 | CCCCCCCCCCC[C@H](O)CC(=O)CCc1ccc(O)c(OC)c1 |
| 3840 | CCCCC[C@H](C)O |
| 3841 | COc1cc(CC[C@H](O)CC(=O)CCc2ccc(O)c(OC)c2)ccc1O |
| 3842 | COc1cc(CC[C@H](O)CC(=O)CCc2cc(OC)c(O)c(OC)c2)ccc1O |
| 3843 | COc1cc(CCC(=O)C[C@H](O)CCc2cc(OC)c(O)c(OC)c2)ccc1O |
| 3844 | COc1cc(CCC(=O)C[C@H](O)CCc2ccc(O)cc2)ccc1O |
| 3845 | COc1cc(CC\C=C\C(=O)CCc2cc(OC)c(O)c(OC)c2)ccc1O |
| 3846 | COc1cc(CC[C@@H](C[C@@H](CCc2ccc(O)c(OC)c2)OC(=O)C)OC(=O)C)ccc1O |
| 3847 | CCCCCCC[C@H](C)O |
| 3848 | CC(C)[C@@H]1CC=C(C)C=C1 |
| 3849 | COc1cc(CCC(=O)C)ccc1O |
| 3850 | C[C@H]1CCC(=C(C)C)CC2=C1CC[C@H]2C |
| 3851 | C[C@@H](CCO)CCC=C(C)C |
| 3852 | CC(C)[C@@H]1CCC(=C)[C@@H]2CCC(=C[C@@H]12)C |
| 3853 | C\C(=C/C=C/C(=C/C=C/C=C(\C)/C=C/C=C(\C)/C=C/C1=C(C)C[C@H](O)CC1(C)C)/C)\C=O |
| 3854 | CC1(C)C2C[C@H]3[C@@H](C2)C13C |
| 3855 | CC(C)(O)[C@@H]1CC[C@@](C)(O1)C=C |
| 3856 | C[C@@H]1CC[C@]23O[C@]12C[C@H](CCC3=O)C(=C)C |
| 3857 | C\C\1=C/CC(C)(C)\C=C\CC(=C)CC1 |
| 3858 | C[C@@H](CCC=C(C)C)[C@@H]1CCC(=C)C=C1 |
| 3859 | C[C@H](CCOC(=O)C)CCC=C(C)C |
| 3860 | O=C(C1CC1)C2CC2 |
| 3861 | C[C@@]12CC[C@@H](C1)C(C)(C)[C@H]2O |
| 3862 | CC(=O)OC[C@](O)(OC(=O)C)[C@@H]1CC[C@@]2(C)[C@H](O)CC[C@@](C)(O)[C@]2(C)[C@H]1O |
| 3863 | CCCO[C@@H]1O[C@H](CO[C@H]2O[C@H](CO)[C@H](O)[C@H](O)[C@@H]2O)[C@H](O)[C@@](O)(OC(=O)CCCCCCC\C=C/C\C=C/C\C=C/CC)C1(O)O |
| 3864 | CCCO[C@@H]1O[C@H](CO)[C@H](O)[C@@](O)(OC(=O)CCCCCCC\C=C/C\C=C/C\C=C/CC)C1(O)O |
| 3865 | CCCCC\C=C/C\C=C/CCCCCCCC(=O)OC[C@@H](O)CO[C@@H]1O[C@H](CO[C@H]2O[C@H](CO)[C@H](O)[C@H](O)[C@H]2O)[C@H](O)[C@H](O)[C@H]1O |
| 3866 | CCCCC\C=C/C\C=C/CCCCCCCC(=O)OC[C@@H](O)CO |
| 3867 | CCCCCCCC\C=C/CCCCCCCC(=O)O[C@]1(O)[C@@H](O)[C@@H](CO[C@H]2O[C@H](CO)[C@H](O)[C@H](O)[C@@H]2O)O[C@@H](OCCC)C1(O)O |
| 3868 | CCCCCCCC\C=C/CCCCCCCC(=O)O[C@]1(O)[C@@H](O)[C@@H](CO)O[C@@H](OCCC)C1(O)O |
| 3869 | CCCCCCCCCCC[C@@H](O)CC(=O)CCc1ccc(O)c(OC)c1 |
| 3870 | C[C@]1(O)CC[C@H]2[C@@H]3CC[C@H]4CC(=O)C=C[C@]4(C)[C@H]3CC[C@]12C |
| 3871 | CC1=CC[C@H](C=C1)C(C)(C)O |
| 3872 | CC1=C[C@H]2C(=C(C)CCCC2(C)C)CC1 |
| 3873 | C1CCCCc2cccc(CCCC1)n2 |
| 3874 | C[C@H](CCC=C(C)C)C=O |
| 3875 | CCCCC[C@@H](C)O |
| 3876 | CCC[C@H](C)C=O |
| 3877 | CCCCC=C(C)C |
| 3878 | C[C@@H](CCC=C(C)C)C=C |
| 3879 | CCCCCC(=O)CCCCc1ccc(O)c(OC)c1 |
| 3880 | C[C@H](CCC=C(C)C)[C@H]1CCC(=O)C=C1 |
| 3881 | COc1cc(CCC(=O)C[C@H](O)CCc2ccc(O)cc2)ccc1O |
| 3882 | C[C@@H](CCCC(=C)C)CCOC(=O)C |
| 3883 | CCCCC[C@H](C[C@H](CCc1ccc(O)c(OC)c1)OC(=O)C)OC(=O)C |
| 3884 | CCCCC[C@@H](O)C[C@H](CCc1ccc(O)c(OC)c1)OC(=O)C |
| 3885 | CCCCC[C@H](C[C@@H](O)CCc1ccc(O)c(OC)c1)OC(=O)C |
| 3886 | CCCCC[C@@H](O)C[C@@H](O)CCc1ccc(O)c(OC)c1 |
| 3887 | CCCCC[C@@H](CC(=O)CCc1ccc(O)c(OC)c1)S(=O)(=O)[O-] |
| 3888 | C[C@@H](O)CO |
| 3889 | CC(C)[C@]12C[C@H]1[C@@H](C)C=C2 |
| 3890 | CC(=CCC[C@@]1(C)[C@@H]2CC[C@@]1(C)[C@H](O)C2)C |
| 3891 | CO[C@@H]1C[C@H](O[C@H]2CC[C@]3(C)[C@H]4C[C@@H](OC(=O)\C=C(/C)\C(C)C)[C@]5(CC(=O)C)[C@@H](O)CC[C@]5(O)[C@]4(O)CC=C3C2)O[C@H](C)[C@@H]1O[C@H]6C[C@@H](OC)[C@@H](O[C@H]7C[C@@H](OC)[C@@H](O[C@@H]8O[C@H](CO)[C@@H](O)[C@H](O)[C@H]8O)[C@@H](C)O7)[C@@H](C)O6 |
| 3892 | Oc1cc(cc(O)c1O)C(=O)O[C@H]2O[C@@H]3COC(=O)c4cc(O)c(O)c(O)c4c5c(O)c(O)c(O)cc5C(=O)O[C@H]6[C@@H]3OC(=O)C7=CC(=O)[C@]8(O)Oc9c([O-])c(O)cc(C(=O)O[C@@H]26)c9[C@H]7C8(O)O |
| 3893 | Oc1cc(cc(O)c1O)C(=O)O[C@H]2O[C@@H]3COC(=O)c4cc(O)c([O-])c(O)c4c5c(O)c(O)c(O)cc5C(=O)O[C@H]6[C@@H]3OC(=O)C7=CC(=O)[C@]8(O)Oc9c([O-])c(O)cc(C(=O)O[C@@H]26)c9[C@H]7C8(O)O |
| 3894 | Oc1cc2C(=O)O[C@H]3[C@@H]4OC(=O)C5=CC(=O)[C@]6(O)Oc7c([O-])c(O)cc(C(=O)O[C@H]3[C@@H](OC(=O)c8cc(O)c([O-])c(O)c8)O[C@@H]4COC(=O)c9cc(O)c([O-])c(O)c9c2c(O)c1O)c7[C@H]5C6(O)O |
| 3895 | COc1cc(O)c2C(=O)C=C(Oc2c1)c3ccc(OC)c(c3)c4c(O)cc(O)c5C(=O)C=C(Oc45)c6ccc(O)cc6 |
| 3896 | COc1cc(O)c2C(=O)C=C(Oc2c1)c3ccc(OC)c(c3)c4c(O)cc(O)c5C(=O)C=C(Oc45)c6ccc([O-])cc6 |
| 3897 | COc1cc(O)c2C(=O)C=C(Oc2c1)c3ccc(OC)c(c3)c4c([O-])cc(O)c5C(=O)C=C(Oc45)c6ccc([O-])cc6 |
| 3898 | CC(=CCc1c(O)c(C=O)cc2c3ccccc3[nH]c12)C |
| 3899 | COc1ccc(cc1)C2=CC(=O)c3c(O)cc(O)c(c3O2)c4cc(ccc4OC)C5=CC(=O)c6c(O)cc([O-])cc6O5 |
| 3900 | COc1ccc(cc1)C2=CC(=O)c3c(O)cc([O-])c(c3O2)c4cc(ccc4OC)C5=CC(=O)c6c(O)cc([O-])cc6O5 |
| 3901 | O[C@H]1CNC[C@@H](O)C1O |
| 3902 | C[C@H](CCC=C(C)C)c1ccc(C)c(O)c1 |
| 3903 | COc1c([O-])cc(O)c2C(=O)C(=C(Oc12)c3ccc(O)cc3)[O-] |
| 3904 | COc1c([O-])cc(O)c2C(=O)C(=C(Oc12)c3ccc([O-])cc3)[O-] |
| 3905 | CC(C)[C@@H]1CC[C@@](C)(C=C)[C@H](C(=C)C)C1=O |
| 3906 | C[C@H]1CC[C@@]2(OC1)O[C@H]3C[C@H]4[C@@H]5CC=C6C[C@H](CC[C@]6(C)[C@H]5CC[C@]4(C)[C@@H]3[C@@H]2C)O[C@@H]7O[C@H](CO)[C@@H](O[C@@H]8O[C@H](CO)[C@@H](O)[C@H](O)[C@H]8O)[C@H](O)[C@H]7O[C@@H]9O[C@@H](C)[C@H](O)[C@@H](O)[C@H]9O |
| 3907 | C[C@H]1CC[C@@]2(OC1)O[C@H]3C[C@H]4[C@@H]5CC=C6C[C@@H](O)CC[C@]6(C)[C@H]5CC[C@]4(C)[C@@H]3[C@@H]2C |
| 3908 | OCCCCCCCCCCO |
| 3909 | C[C@H](CCO)CCC=C(C)C |
| 3910 | OC[C@@H](O)[C@H]1OC(=O)C(=C1O)[O-] |
| 3911 | C[NH+]1[C@@H]2CC[C@H]1CC(C2)OC(=O)[C@H](CO)c3ccccc3 |
| 3912 | CC(C)[C@@H](C)CC[C@@H](C)[C@H]1CC[C@H]2[C@@H]3CC=C4C[C@@H](O)CC[C@]4(C)[C@H]3CC[C@]12C |
| 3913 | [O-]C(=O)\C=C\c1ccccc1 |
| 3914 | C\C=C(\CC[C@@H](C)[C@H]1CC[C@H]2C3=CC[C@H]4[C@H](C)[C@@H](O)CC[C@]4(C)[C@H]3CC[C@]12C)/C(C)C |
| 3915 | C\C(=C/C=C/C=C(\C)/C=C/C=C(\C)/C=C/C1=C(C)C[C@@H](O)CC1(C)C)\C=C\C=C(/C)\C=C\C2=C(C)CCCC2(C)C |
| 3916 | CC[C@@]12CC[C@]3(C)[C@H](CC[C@@]3(C)[C@@H]1CC[C@@H]4[C@H]2CC[C@H](O)C4(C)C)[C@H](C)CCCC(C)C |
| 3917 | CC[C@@]12CC[C@]3(C)[C@H](CC[C@@]3(C)[C@@H]1CC[C@H]4[C@H](C)[C@@H](O)CC[C@H]24)[C@H](C)CCC(=C)C(C)C |
| 3918 | CC(=O)\C=C\C1=C(C)[C@@H](O)CCC1(C)C |
| 3919 | C[NH+]1[C@@H]2CC[C@H]1CC(C2)OC(=O)[C@H](CO)c3ccccc3 |
| 3920 | CC(=O)\C=C\C1=C(C)CCCC1(C)C |
| 3921 | C[C@H](CCC=C(C)C)[C@H]1CC[C@@]2(C)C3=C(CC[C@]12C)[C@@]4(C)CC[C@H](O)C(C)(C)[C@@H]4CC3 |
| 3922 | C[C@H](CCC(=C(C)C)C)[C@H]1CC[C@H]2[C@@H]3CC=C4C[C@@H](O)CC[C@]4(C)[C@H]3CC[C@]12C |
| 3923 | CC(C)CCC[C@@H](C)[C@H]1CC[C@H]2C3=C(CC[C@]12C)[C@@]4(C)CC[C@H](O)[C@@H](C)[C@@H]4CC3 |
| 3924 | CC(C)C(=C)CC[C@@H](C)[C@H]1CC[C@H]2[C@@H]3CC=C4C[C@@H](O)CC[C@]4(C)[C@H]3CC[C@]12C |
| 3925 | CC(C)C(=C)CC[C@@H](C)[C@H]1CC[C@@]2(C)[C@@H]3CC[C@H]4C(C)(C)[C@@H](O)CC[C@@]45C[C@@]35CC[C@]12C |
| 3926 | CC(C)C(=C)CC[C@@H](C)[C@H]1CC[C@@]2(C)C3=C(CC[C@]12C)[C@@]4(C)CC[C@H](O)C(C)(C)[C@@H]4CC3 |
| 3927 | CCC(=C(C)C)CC[C@@H](C)[C@H]1CC[C@H]2C3=CC[C@H]4[C@H](C)[C@@H](O)CC[C@]4(C)[C@H]3CC[C@]12C |
| 3928 | CC(C)[C@@H](C)CC[C@@H](C)[C@H]1CC[C@H]2C3=CC[C@H]4[C@H](C)[C@@H](O)CC[C@]4(C)[C@H]3CC[C@]12C |
| 3929 | CC(C)[C@H](C)CC[C@@H](C)[C@H]1CC[C@@]2(C)[C@@H]3CC[C@H]4[C@H](C)[C@@H](O)CC[C@]4(C)C3=CC[C@]12C |
| 3930 | CC[C@@]12CC[C@]3(C)[C@H](CC[C@@]3(C)[C@@H]1CC[C@H]4[C@H](C)[C@@H](O)CC[C@H]24)[C@H](C)CCCC(C)C |
| 3931 | CC(C)CCC[C@@H](C)[C@H]1CC[C@@]2(C)C3=C(CC[C@]12C)[C@@]4(C)CC[C@H](O)[C@@H](C)[C@@H]4CC3 |
| 3932 | CC(C)CCC[C@@H](C)[C@H]1CC[C@@]2(C)[C@@H]3CC[C@H]4[C@H](C)[C@@H](O)CC[C@]4(C)C3=CC[C@]12C |
| 3933 | CC(C)C(=C)CC[C@@H](C)[C@H]1CC[C@@]2(C)C3=C(CC[C@]12C)[C@@]4(C)CC[C@H](O)[C@@H](C)[C@@H]4CC3 |
| 3934 | CCCCCCCCCCCCCCCC(=O)O[C@@H]1CC(=C(\C=C\C(=C\C=C\C(=C\C=C\C=C(/C)\C=C\C=C(/C)\C=C\C2=C(C)C[C@H](CC2(C)C)OC(=O)CCCCCCCCCCCCCCC)\C)\C)C(C)(C)C1)C |
| 3935 | CC1=C(C=O)C(C)(C)CC=C1 |
| 3936 | C[C@H](CCC(=C(C)C)C)[C@H]1CC[C@@]2(C)C3=C(CC[C@]12C)[C@@]4(C)CC[C@H](O)[C@@H](C)[C@@H]4CC3 |
| 3937 | [NH3+][C@@H](Cc1c[nH]c2ccccc12)C(=O)[O-] |
| 3938 | C\C(=C/C=C/C=C(\C)/C=C/C=C(\C)/C=C/C1=C(C)C[C@@H](O)CC1(C)C)\C=C\C=C(/C)\C=C\C2=C(C)C[C@@H](O)CC2(C)C |
| 3939 | C=CCc1ccc2OCOc2c1 |
| 3940 | COc1cc2C=CC(=O)Oc2cc1O[C@H]3O[C@H](CO)[C@H](O)[C@@H](O)[C@H]3O |
| 3941 | CC(=C)[C@@H]1CC[C@]2(C)CC[C@]3(C)[C@H](CC[C@@H]4[C@@]5(C)CC[C@H](OC(=O)C)C(C)(C)[C@@H]5CC[C@@]34C)[C@@H]12 |
| 3942 | CCCCCCCCCCCCCCCCCCCCCCCCCCCCCCCCO |
| 3943 | C\C=C(\CC[C@@H](C)[C@H]1CC[C@H]2C3=CC[C@H]4[C@H](C)[C@@H](O)CC[C@]4(C)[C@H]3CC[C@]12C)/C(C)C |
| 3944 | CCOC(=O)CCCCCCC\C=C\C\C=C\C\C=C\CC |
| 3945 | CCCCCCCCCCCCCC(=O)OCC |
| 3946 | C=CCCCCCC\C=C/CCCCCCCC=O |
| 3947 | OC[C@H](O)[C@H]1OC(=O)C(=C1O)[O-] |
| 3948 | CCCCCCCCCCCCCCCCCCCCCC=C |
| 3949 | CC(C)C(=C)CC[C@@H](C)[C@H]1CC[C@@]2(C)[C@@H]3CC[C@H]4C(C)(C)[C@@H](O)CC[C@@]45C[C@@]35CC[C@]12C |
| 3950 | COC(=O)[C@H]1O[C@@H](O[C@H]2CC[C@@]3(C)[C@H](CC[C@]4(C)[C@@H]3CC=C5[C@@H]6CC(C)(C)[C@@H](OC(=O)\C(=C/C)\C)[C@H](OC(=O)\C(=C/C)\C)[C@]6(CO)[C@H](O)[C@H](O)[C@@]45C)[C@]2(C)C(=O)OC)[C@H](O)[C@@H](O[C@@H]7OC[C@H](O)[C@H](O)[C@H]7O)[C@@H]1O |
| 3951 | COC(=O)[C@]1(C)[C@@H](O)CC[C@@]2(C)[C@@H]1CC[C@]3(C)[C@@H]2CC=C4[C@H]5CC(C)(C)[C@@H](OC(=O)\C(=C/C)\C)[C@H](OC(=O)\C(=C/C)\C)[C@]5(CO)[C@H](O)[C@H](O)[C@@]34C |
| 3952 | COc1cc(\C=C/C(=O)O[C@H]2CC[C@]3(C)[C@H]4CC[C@]5(C)[C@H](CC[C@@H]5[C@@H]4CC=C3C2)[C@H](C)CCCC(C)C)ccc1O |
| 3953 | COc1cc(\C=C\C(=O)O[C@H]2CC[C@]3(C)[C@H]4CC[C@]5(C)[C@H](CC[C@@H]5[C@@H]4CC=C3C2)[C@@H](C)CC[C@@H](C)C(C)C)ccc1O |
| 3954 | C\C(=C/C=C/C=C(\C)/C=C/C=C(\C)/C=C/C1=C(C)C[C@H](O)CC1(C)C)\C=C\C=C(/C)\C=C\[C@H]2C(=C[C@H](O)CC2(C)C)C |
| 3955 | [O-]C(=O)C1=COC(=O)C=C1 |
| 3956 | C[C@H]1CC[C@@H]2C[C@H](C)CC[C@H]2C1 |
| 3957 | Cc1ccc2c(C=CCC2(C)C)c1 |
| 3958 | NC(=O)NCCC[C@H]([NH3+])C(=O)[O-] |
| 3959 | CCCCCCCCCCCCCCCCCCCCCCCCCCCCCCCCCCCCCCCCCCCC |
| 3960 | CC(C)[C@@H](C)CC[C@H](C)[C@@H]1CC[C@H]2[C@H]3CC=C4C[C@@H](O)CC[C@]4(C)[C@@H]3CC[C@@]12C |
| 3961 | CCCCCCCCCCCCCCCCC\C=C\CCCCCCCCCCCCCCCC |
| 3962 | CC(C)CCC[C@H](C)CCC[C@H](C)CCCC(C)C |
| 3963 | CCCCCCCCC#CCCCCCCCC |
| 3964 | CCCC\C=C\C\C=C\CCCCCCCCC(=O)OC |
| 3965 | CC(C)CCN1C=C\C(=C/c2cc[n+](CCC(C)C)c3ccccc23)\c4ccccc14 |
| 3966 | CC(C)C(=C)CC[C@@H](C)[C@H]1CC[C@H]2C3=CC[C@H]4[C@H](C)[C@@H](O)CC[C@]4(C)[C@H]3CC[C@]12C |
| 3967 | CC(C)[C@@H]1CC[C@@](C)(O)[C@H]2CCC(=C[C@@H]12)C |
| 3968 | CC[C@@H](CC[C@@H](C)[C@@H]1CC[C@H]2[C@H]3CC=C4C[C@@H](CC[C@@]4(C)[C@H]3CC[C@@]12C)O[C@@H]5O[C@H](CO)[C@@H](O)[C@H](O)[C@H]5O)C(C)C |
| 3969 | CC[C@@H](CC[C@@H](C)[C@@H]1CC[C@H]2[C@H]3CC=C4C[C@H](O)CC[C@@]4(C)[C@H]3CC[C@@]12C)C(C)C |
| 3970 | C[C@H]1CC(=O)C=C(C)[C@]12CC[C@H](C2)C(=C)C |
| 3971 | CCCCCCCCCCCC(CCCCCCCCCC)CCCCCCCCCC |
| 3972 | CCCCCCCCCCC[C@@H](CCCCCCCCC)CCCCCCCCCC |
| 3973 | CCCCCCCCCCCC[C@H](C)CCCC(C)CCC[C@H](C)CCCCCCCCCCCC |
| 3974 | CC[C@H]1CC[C@@H]2[C@@H]3CC[C@@H]4CCCC[C@]4(C)[C@H]3CC[C@]12C |
| 3975 | CC1C(=C(C)C(=C1C)C)C |
| 3976 | CC(=C)[C@H]1CC[C@@]2(C)C=CC(=O)C(=C2C1)C |
| 3977 | C[C@H]1CCC[C@H]2[C@H](C)CCC[C@H]12 |
| 3978 | BrCCCCCCBr |
| 3979 | CC(C)[C@]1(C)CCC[C@@H]2C=C(C)CC=C12 |
| 3980 | C[C@H]1CC[C@@H]2[C@@H](C)CCC[C@H]2C1 |
| 3981 | CCCCCCCCCCCCCCCCCCI |
| 3982 | CC[C@H](CC[C@@H](C)[C@H]1CC[C@@]2(C)C3=CC[C@@H]4[C@H](C)[C@H](CC[C@]4(C)[C@H]3CC[C@]12C)OC(=O)C)C(=C)C |
| 3983 | CC(C)[C@@H](C)CC[C@@H](C)[C@@H]1CC[C@H]2C3=CC[C@@H]4C[C@H](O)CC[C@@]4(C)[C@H]3CC[C@@]12C |
| 3984 | CC(C)[C@@H](C)\C=C\[C@@H](C)[C@@H]1CC[C@H]2C3=CC[C@@H]4C[C@H](O)CC[C@@]4(C)[C@H]3CC[C@@]12C |
| 3985 | CC(C)C(=C)CC[C@@H](CO)[C@H]1CC[C@@]2(C)[C@@H]3CC[C@H]4C(C)(C)[C@@H](O)CC[C@@]45C[C@@]35CC[C@]12C |
| 3986 | COc1cc(\C=C\C(=O)O[C@H]2CC[C@]34C[C@]35CC[C@]6(C)[C@H](CC[C@@]6(C)[C@@H]5CC[C@H]4C2(C)C)[C@H](C)CCC(=C)C(C)C)ccc1O |
| 3987 | CC[C@@H](\C=C\[C@@H](C)[C@H]1CC[C@H]2[C@@H]3CC[C@@H]4C[C@H](O)CC[C@]4(C)[C@H]3CC[C@]12C)C(C)C |
| 3988 | CC[C@@H](\C=C\[C@@H](C)[C@H]1CC[C@H]2[C@@H]3CC=C4C[C@H](O)CC[C@]4(C)[C@H]3CC[C@]12C)C(C)C |
| 3989 | CC[C@H](CC[C@@H](C)[C@H]1CC[C@H]2[C@@H]3CC[C@H]4C[C@@H](O)CC[C@]4(C)[C@H]3CC[C@]12C)C(C)C |
| 3990 | CC(C)[C@H](C)CC[C@H](C)[C@H]1CC[C@@]2(C)[C@H]3CC[C@@H]4[C@@H](C)[C@H](O)CC[C@]4(C)C3=CC[C@]12C |
| 3991 | CC(C)C(=C)CC[C@@H](C)[C@H]1CC[C@@]2(C)C3=C(CC[C@]12C)[C@@]4(C)CC[C@H](O)C(C)(C)[C@H]4CC3 |
| 3992 | C\C=C(/CC[C@@H](C)[C@H]1CC[C@H]2[C@@H]3CC=C4C[C@@H](O)CC[C@]4(C)[C@H]3CC[C@]12C)\C(C)C |
| 3993 | CC(C)CCCCCCCCCCCCCCCCCCC(C)C |
| 3994 | C[C@H]1C[C@H]2CCCC[C@@H]2C[C@@H]1C |
| 3995 | CC[C@@H](C)CCCC[C@@H](C)CCC[C@@H](C)CCCC(C)C |
| 3996 | CCCCCC[C@@H](C)CCC[C@@H](C)CCCC(C)C |
| 3997 | CCCCCC[C@@H](C)CC[C@H](C)C(C)C |
| 3998 | CO[C@]1(F)C[C@H]1c2ccccc2 |
| 3999 | CCC[C@@H](CC)CCC(C)C |
| 4000 | OC[C@H](O)[C@H]1OC(=O)C(=C1O)O[C@H]2O[C@H](CO)[C@@H](O)[C@H](O)[C@H]2O |
| 4001 | C[C@@H](CCC[C@@H](C)C(=C)C)[C@H]1CC[C@@]2(C)[C@@H]3CC[C@@H]4[C@H](C)[C@@H](O)CC[C@@]45C[C@@]35CC[C@]12C |
| 4002 | CC(C)CCC[C@H](C)[C@H]1CC[C@]2(C)[C@@H]3CC[C@H]4[C@@H](C)[C@@H](O)CC[C@@]45C[C@@]35CC[C@]12C |
| 4003 | CC(C)CCC[C@H](C)[C@H]1CC[C@@]2(C)[C@H]3CC[C@@H]4[C@@H](C)[C@H](O)CC[C@]4(C)C3=CC[C@]12C |
| 4004 | C[C@H](CCC=C(C)C)[C@@H]1CC[C@@]2(C)C3=C(CC[C@@]12C)[C@]4(C)CC[C@@H](O)[C@@H](C)[C@H]4CC3 |
| 4005 | CC(C)[C@H](C)CC[C@H](C)[C@H]1CC[C@H]2C3=CC[C@H]4[C@H](C)[C@@H](O)CC[C@]4(C)[C@H]3CC[C@]12C |
| 4006 | C\C=C(/C)\C\C=C(\C)/[C@H]1CCC=CC1 |
| 4007 | COc1ccc(\C=C/[C@@H](C=C)c2ccc(O)cc2)cc1 |
| 4008 | CC(=O)c1cnc(NN)nc1C |
| 4009 | CC(C)CCC[C@@H](C)[C@H]1CC[C@H]2C3=CC[C@H]4[C@H](C)[C@@H](O)CC[C@]4(C)[C@H]3CC[C@]12C |
| 4010 | C[C@H](CCC(=C(C)C)C)[C@H]1CC[C@@]2(C)C3=C(CC[C@]12C)[C@@]4(C)CC[C@@H](O)[C@@H](C)[C@H]4CC3 |
| 4011 | C[C@H](CCC(=C(C)C)C)[C@H]1CC[C@H]2C3=CC[C@H]4[C@H](C)[C@@H](O)CC[C@]4(C)[C@H]3CC[C@]12C |
| 4012 | CCCCC(CCCC)CCCC |
| 4013 | CC(C)CCC[C@@H](C)[C@H]1CC[C@H]2C3=CC(=C4C[C@@H](O)CC[C@]4(C)[C@H]3CC[C@]12C)F |
| 4014 | COc1cc2O[C@H](CC(=O)c2c(O)c1O[C@H]3O[C@H](CO)[C@@H](O)[C@H](O)[C@H]3O)c4ccc(O)c(O)c4 |
| 4015 | COc1cc2O[C@H](CC(=O)c2c(O)c1O)c3ccc(O)c(O)c3 |
| 4016 | COc1cc2O[C@H](CC(=O)c2c(O)c1[O-])c3ccc(O)c(O)c3 |
| 4017 | CCCCCCCC\C=C(/CBr)\CCCCCC |
| 4018 | CCCCCCCCC(CCCCCCCC)CCCCCCCC |
| 4019 | COC(C)(C)C\C=C\C(=C\C=C\C(=C\C=C\C(=C\C=C\C=C(/C)\C=C\C=C(/C)\C=C\C=C(/C)\CCC=C(C)C)\C)\C)\C |
| 4020 | C[NH+]1[C@@H]2CC[C@H]1CC(C2)OC(=O)[C@@H](CO)c3ccccc3 |
| 4021 | C\C(=C/C=C/C=C(\C)/C=C\C=C(\C)/C=C/[C@@]12O[C@]1(C)C[C@@H](O)CC2(C)C)\C=C\C=C(/C)\C=C\C3=C(C)CCCC3(C)C |
| 4022 | CC(C)CCC[C@@H](C)[C@H]1CC[C@@]2(C)[C@@H]3CC[C@@H]4C(C)(C)[C@H](CC[C@@]45C[C@@]35CC[C@]12C)OC(=O)C |
| 4023 | CC(C)C(=C)CC[C@@H](C)[C@H]1CC[C@@]2(C)[C@@H]3CC[C@H]4[C@H](C)[C@@H](O)CC[C@@]45C[C@@]35CC[C@]12C |
| 4024 | C[C@H](CC[C@H](O)C(C)(C)O)[C@H]1[C@H](C[C@@]2(C)[C@@H]3CC[C@H]4[C@@](C)(CO)[C@H](CC[C@@]45C[C@@]35CC[C@]12C)O[C@@H]6OC[C@H](O)[C@H](O)[C@H]6O)O[C@@H]7O[C@H](CO[C@@H]8O[C@@H](C)[C@H](O)[C@@H](O)[C@H]8O)[C@@H](O)[C@H](O)[C@H]7O |
| 4025 | C[C@H](CC[C@H](O)C(C)(C)O)[C@H]1[C@@H](O)C[C@@]2(C)[C@H]3CC[C@@H]4[C@@](C)(CO)[C@@H](O)CC[C@@]45C[C@@]35CC[C@]12C |
| 4026 | CCCC\C=C\CCCCCCCC\C=C\CC(=O)OCC |
| 4027 | CCOC(=O)c1ccc(C)cc1 |
| 4028 | [NH3+][C@H](CCC(=O)[O-])C(=O)[O-] |
| 4029 | COC(=O)C1=CO[C@H](O[C@@H]2O[C@H](CO)[C@@H](O)[C@H](O)[C@H]2O)[C@H]3[C@](C)(O)CC[C@@]13O |
| 4030 | COC(=O)C1=CO[C@H](O)[C@H]2[C@](C)(O)CC[C@@]12O |
| 4031 | C\C=C(/C)\C(=O)O[C@@H]1CC(C)(C)C[C@H]2C3=CC[C@@H]4[C@@]5(C)CCC(=O)C(C)(C)[C@H]5CC[C@@]4(C)[C@]3(C)CC[C@@]12C(=O)O |
| 4032 | C\C=C(/C)\C(=O)O[C@@H]1CC(C)(C)C[C@H]2C3=CC[C@@H]4[C@@]5(C)CCC(=O)C(C)(C)[C@H]5CC[C@@]4(C)[C@]3(C)CC[C@@]12C(=O)[O-] |
| 4033 | C[C@]1(O)CCC(=O)[C@@]2(C)CC[C@@H]3[C@H]([C@@H]12)C3(C)C |
| 4034 | C[C@@]12C[C@H](OC(=O)C1=C)[C@]3(C)OC(=O)[C@]4(O)CC[C@H]5[C@@H]([C@H](O)C=C6CC=CC(=O)[C@]56C)[C@]7(O)O[C@@]34[C@H]2C7=O |
| 4035 | C[C@@H](CCC(=O)NCCS(=O)(=O)[O-])[C@H]1CC[C@@H]2[C@@H]3[C@H](O)C[C@@H]4C[C@H](O)CC[C@]4(C)[C@H]3CC[C@]12C |
| 4036 | CCCC\C=C/CCCCCCCCCC(=O)[O-] |
| 4037 | C\C\1=C/CC[C@@]2(C)O[C@@H]2C(=O)c3c(C)coc3C1 |
| 4038 | Nc1cccc[nH+]1 |
| 4039 | Nc1ccccn1 |
| 4040 | CC(C)CCC[C@@H](C)[C@H]1CC[C@H]2[C@@H]3CC[C@H]4C[C@@H](O)CC[C@]4(C)[C@H]3CC[C@]12C |
| 4041 | C[C@@H]1CCCC[C@@]12CCC[C@@H]2C |
| 4042 | C\C(=C/C=C/C=C(\C)/C=C/C=C(\C)/C=C/C1=C(C)C[C@H](O)CC1(C)C)\C=C\C=C(/C)\C=C\C2=C(C)CCCC2(C)C |
| 4043 | CCOC(=O)c1ccc(OC)cc1 |
| 4044 | NC(=O)CC[C@@H]([NH3+])C(=O)[O-] |
| 4045 | CC(C)CCC[C@@H](C)[C@H]1CC[C@@]2(C)C3=C(CC[C@]12C)[C@@]4(C)CC[C@H](O)C(C)(C)[C@@H]4CC3 |
| 4046 | CC(C)CCC[C@@H](C)[C@H]1CC[C@@]2(C)C3=C(CC[C@]12C)[C@@]4(C)CC[C@@H](O)C(C)(C)[C@H]4CC3 |
| 4047 | CC(C)CCC[C@@H](C)[C@H]1CC[C@H]2C3=CC[C@H]4C[C@@H](O)CC[C@]4(C)[C@H]3CC[C@]12C |
| 4048 | C\C(=C/C=C/C=C(\C)/C=C/C=C(\C)/[C@H]1O[C@H]2C[C@H](O)CC(C)(C)C2=C1)\C=C\C=C(/C)\C=C/C3=C(C)C[C@@H](O)CC3(C)C |
| 4049 | CC1(C)CC[C@H]2O[C@]23C(C)(C)[C@H]4CC[C@@]13C4 |
| 4050 | CCCCCCCCCCCCCCCCCCOC(=O)c1ccc(cc1)[N+](=O)[O-] |
| 4051 | C[C@H]1CCCC[C@@]12CCC[C@@H]2C |
| 4052 | CC(=CCC\C(=C\C=C\C(=C\C=C\C(=C\C=C\C=C(/C)\C=C\C=C(/C)\C=C\[C@H]1C(=CCCC1(C)C)C)\C)\C)\C)C |
| 4053 | CC(C)[C@H]1CC[C@@H]2[C@]1(C)CC[C@]3(C)[C@H]4CC[C@H]5C(C)(C)CCC[C@]5(C)C4=CC[C@@]23C |
| 4054 | CC(C)[C@H]1CC[C@@H]2[C@]1(C)CC[C@]3(C)C4=CC[C@H]5C(C)(C)CCC[C@]5(C)C4=CC[C@@]23C |
| 4055 | Oc1ccc(cc1)C(=O)c2c(O)cc(O)cc2O |
| 4056 | Oc1cc(O)c(C(=O)c2ccc([O-])cc2)c(O)c1 |
| 4057 | Oc1cc(O)c(C(=O)c2ccc([O-])cc2)c([O-])c1 |
| 4058 | COc1cc(O)c(C[C@@H](CC=C(C)C)C(=C)C)c2O[C@@H](CC(=O)c12)c3ccc(O)cc3O |
| 4059 | COC(=O)C[C@H]1C[C@@H](O[C@@H]2O[C@H](CO)[C@@H](O)[C@H](O)[C@H]2O)[C@]3(C)CCC(=CC[C@H]13)C(=O)OC |
| 4060 | COC(=O)C[C@@H]1C[C@@H](O)[C@]2(C)CCC(=CC[C@H]12)C(=O)OC |
| 4061 | C[C@@H]1O[C@@H](O[C@@H]2[C@@H](O)[C@H](O)[C@@H](CO)O[C@H]2Oc3cc(O)c4C(=O)C[C@H](Oc4c3)c5ccc(O)c(O)c5)[C@H](O)[C@H](O)[C@H]1O |
| 4062 | O[C@H]1[C@@H](O)[C@@](O[C@@H]2Cc3c(O)cc(O)cc3O[C@H]2c4ccc(O)c(O)c4)(Oc5cc(O)cc(O)c15)c6ccc(O)c(O)c6 |
| 4063 | CC(=CC[C@H](Cc1c(O)cc(O)c2C(=O)C[C@H](Oc12)c3ccc(O)cc3O)C(=C)C)C |
| 4064 | CC(=CC[C@H](Cc1c([O-])cc(O)c2C(=O)C[C@H](Oc12)c3ccc(O)cc3O)C(=C)C)C |
| 4065 | COc1cc(O)c(C(=O)\C=C\c2ccc(O)cc2)c(O)c1CC=C(C)C |
| 4066 | C[C@@H]1O[C@@H](OC[C@H]2O[C@@H](Oc3cc(O)c4C(=O)C[C@H](Oc4c3)c5ccc(O)cc5)[C@H](O)[C@@H](O)[C@@H]2O)[C@H](O)[C@H](O)[C@H]1O |
| 4067 | Oc1ccc(cc1)[C@H]2CC(=O)c3c(O)cc(O)cc3O2 |
| 4068 | COc1cc(C[C@@H](CO)[C@H](CO)Cc2ccc(O)c(OC)c2)ccc1O |
| 4069 | Oc1cc(O)c2C(=O)\C(=C\c3ccc(O)c(O)c3)\Oc2c1 |
| 4070 | Oc1cc([O-])c2C(=O)\C(=C\c3ccc(O)c(O)c3)\Oc2c1 |
| 4071 | CC(=C)[C@H]1CC[C@@]2(C)[C@H]1CC[C@]3(C)[C@@H]2CC[C@@H]4[C@@]5(C)CCCC(C)(C)[C@@H]5CC[C@@]34C |
| 4072 | CC(C)(O)[C@H]1CC[C@@]2(C)[C@H]1CC[C@]3(C)[C@@H]2CC[C@@H]4[C@@]5(C)CCCC(C)(C)[C@@H]5CC[C@@]34C |
| 4073 | Oc1cc(O)c2C(=O)C[C@@H](Oc2c1)c3ccc(O)c(O)c3 |
| 4074 | CC1=C(O[C@@H]2O[C@H](CO)[C@@H](O)[C@H](O)[C@H]2O)C(=O)C=CO1 |
| 4075 | CC1=C([O-])C(=O)C=CO1 |
| 4076 | OC[C@H]1O[C@H]([C@H](O)[C@@H](O)[C@@H]1O)c2c(O)cc(O)c3C(=O)C=C(Oc23)c4ccc(O)c(O)c4 |
| 4077 | OC[C@H]1O[C@H]([C@H](O)[C@@H](O)[C@@H]1O)c2c([O-])cc(O)c3C(=O)C=C(Oc23)c4ccc(O)c(O)c4 |
| 4078 | C[C@@H]1O[C@@H](OC2=C(Oc3cc(O)cc(O)c3C2=O)c4ccc(O)cc4)[C@H](O)[C@H](O)[C@H]1O |
| 4079 | C[C@@H]1O[C@@H](OC2=C(Oc3cc(O)cc(O)c3C2=O)c4ccc([O-])cc4)[C@H](O)[C@H](O)[C@H]1O |
| 4080 | OC[C@H]1O[C@H]([C@H](O)[C@@H](O)[C@@H]1O)c2c(O)cc3Oc4cc(O)c(O)cc4C(=O)c3c2O |
| 4081 | O[C@@H]1Cc2c(O)cc(O)cc2O[C@@H]1c3ccc(O)c(O)c3 |
| 4082 | CC(=CC[C@H](Cc1c([O-])cc(O)c2C(=O)C(=C(Oc12)c3ccc(O)cc3O)[O-])C(=C)C)C |
| 4083 | COc1cc(O)ccc1[C@@H]2CC(=O)c3c(O)cc(O)c(C[C@@H](CC=C(C)C)C(=C)C)c3O2 |
| 4084 | COc1cc(O)ccc1[C@@H]2CC(=O)c3c(O)cc([O-])c(C[C@@H](CC=C(C)C)C(=C)C)c3O2 |
| 4085 | O[C@@H]1Cc2c(O)cc(O)cc2O[C@@H]1c3ccc(O)cc3 |
| 4086 | O[C@@H]1Cc2c(O)cc(O)c([C@@H]3[C@@H](O)[C@H](Oc4cc(O)cc(O)c34)c5ccc(O)c(O)c5)c2O[C@@H]1c6ccc(O)c(O)c6 |
| 4087 | O[C@@H]1Cc2c(O)cc([O-])c([C@@H]3[C@@H](O)[C@H](Oc4cc(O)cc(O)c34)c5ccc(O)c(O)c5)c2O[C@@H]1c6ccc(O)c(O)c6 |
| 4088 | OC[C@H]1O[C@@H](Oc2cc(O)cc3O[C@@H]([C@@H](O)Cc23)c4ccc(O)c(O)c4)[C@@H](O)[C@@H](O)[C@@H]1O |
| 4089 | OC[C@H]1O[C@@H](Oc2cc([O-])cc3O[C@@H]([C@@H](O)Cc23)c4ccc(O)c(O)c4)[C@@H](O)[C@@H](O)[C@@H]1O |
| 4090 | CC[C@@H](\C=C\[C@@H](C)[C@@H]1CC[C@H]2[C@H]3CC[C@@H]4CC(=O)CC[C@]4(C)[C@@H]3CC[C@@]12C)C(C)C |
| 4091 | OC[C@H]1O[C@@H](Oc2cc(O)c3C(=O)\C(=C\c4ccc(O)c(O)c4)\Oc3c2)[C@H](O)[C@@H](O)[C@@H]1O |
| 4092 | OC[C@H]1O[C@@H](Oc2cc([O-])c3C(=O)\C(=C\c4ccc(O)c(O)c4)\Oc3c2)[C@H](O)[C@@H](O)[C@@H]1O |
| 4093 | C[C@@H](CC[C@H](C)C(=C)C)[C@H]1CC[C@@]2(C)[C@H]3CC[C@H]4C(C)(C)[C@@H](CC[C@]45C[C@]35CC[C@]12C)OC(=O)C |
| 4094 | OC[C@H]1O[C@@H](Oc2cc(O)cc3O[C@@H]([C@H](O)Cc23)c4ccc(O)cc4)[C@@H](O)[C@@H](O)[C@@H]1O |
| 4095 | OC[C@H]1O[C@@H](Oc2cc([O-])cc3O[C@@H]([C@H](O)Cc23)c4ccc(O)cc4)[C@@H](O)[C@@H](O)[C@@H]1O |
| 4096 | OC[C@H]1O[C@@H](O[C@@H]2Cc3c(O)cc(O)cc3O[C@@H]2c4ccc(O)c(O)c4)[C@H](O)[C@H](O)[C@@H]1O |
| 4097 | O[C@@H]1[C@@H](O)[C@H](Oc2cc(O)c3C(=O)C[C@@H](Oc3c2)c4ccc(O)c(O)c4)O[C@@H]([C@H]1O)C(=O)[O-] |
| 4098 | CC1(C)CCC[C@@]2(C)[C@H]1CC[C@]3(C)[C@@H]2CC[C@@H]4[C@@]5(C)CC(=O)C[C@H]5CC[C@@]34C |
| 4099 | O[C@H]1CO[C@@H](Oc2cc(O)c3C(=O)C(=C(Oc3c2)c4ccc(O)cc4)[O-])[C@@H](O)[C@H]1O |
| 4100 | O[C@H]1CO[C@@H](Oc2cc(O)c3C(=O)C(=C(Oc3c2)c4ccc([O-])cc4)[O-])[C@@H](O)[C@H]1O |
| 4101 | OC[C@H]1O[C@H]([C@H](O)[C@@H](O)[C@@H]1O)c2c(O)c([C@@H]3O[C@H](CO)[C@@H](O)[C@H](O)[C@H]3O)c4OC(=CC(=O)c4c2O)c5ccc(O)c(O)c5 |
| 4102 | O[C@@H]1[C@@H](O)[C@H](Oc2cc(O)c3C(=O)C=C(Oc3c2)c4ccc(O)c(O)c4)O[C@@H]([C@H]1O)C(=O)[O-] |
| 4103 | C[C@@H]1O[C@@H](O[C@H]2[C@@H](O)[C@H](O)[C@H](CO)O[C@H]2Oc3ccc(cc3O)C4=CC(=O)c5c(O)cc(O[C@@H]6O[C@@H](CO)[C@@H](O)[C@@H](O)[C@H]6O)cc5O4)[C@@H](O)[C@@H](O)[C@H]1O |
| 4104 | O[C@H]1Cc2c(O)cc(O)c([C@@H]3[C@@H](O)[C@@H](Oc4cc(O)cc([O-])c34)c5ccc(O)c(O)c5)c2O[C@H]1c6ccc(O)c(O)c6 |
| 4105 | O[C@H]1Cc2c(O)cc([O-])c([C@@H]3[C@@H](O)[C@@H](Oc4cc(O)cc([O-])c34)c5ccc(O)c(O)c5)c2O[C@H]1c6ccc(O)c(O)c6 |
| 4106 | CC1=CC(=O)c2c(O)c(ccc2C1=O)C3=C(C)C(=O)c4cccc(O)c4C3=O |
| 4107 | C[C@@H](CCC=C(C)C)[C@H]1CC[C@]2(C)[C@H]3CC[C@H]4C(C)(C)C(=O)CC[C@]45C[C@@]35CC[C@]12C |
| 4108 | C[C@@H](CC[C@H](C)C(=C)C)[C@H]1CC[C@@]2(C)[C@H]3CC[C@H]4C(C)(C)[C@@H](O)CC[C@]45C[C@]35CC[C@]12C |
| 4109 | OC[C@H]1O[C@@H](O[C@@H]2Cc3c(O)cc(O)c([C@H]4CCC(=O)N4)c3O[C@@H]2c5ccc(O)c(O)c5)[C@H](O)[C@H](O)[C@@H]1O |
| 4110 | O[C@@H]1Cc2c(O)cc(O)c([C@H]3CCC(=O)N3)c2O[C@@H]1c4ccc(O)c(O)c4 |
| 4111 | OC[C@H]1O[C@@H](O[C@@H]2Cc3c(O)cc(O)c([C@@H]4CCC(=O)N4)c3O[C@H]2c5ccc(O)c(O)c5)[C@H](O)[C@H](O)[C@@H]1O |
| 4112 | O[C@@H]1Cc2c(O)cc(O)c([C@@H]3CCC(=O)N3)c2O[C@H]1c4ccc(O)c(O)c4 |
| 4113 | OC[C@H]1O[C@H](Oc2cc(O)c3C(=O)C[C@H](Oc3c2)c4ccc(O)c(O)c4)[C@H](O)[C@@H](O)[C@@H]1O |
| 4114 | Oc1ccc(cc1)C(=O)[O-] |
| 4115 | [O-]C(=O)c1ccc([O-])cc1 |
| 4116 | COC(=O)\C=C\c1ccccc1 |
| 4117 | O=C\C=C/c1ccccc1 |
| 4118 | O=C\C=C\c1ccccc1 |
| 4119 | CC(=O)O[C@H]1C[C@H]2CC[C@]1(C)C2(C)C |
| 4120 | CC(=O)c1ccccc1C |
| 4121 | CC(C)[C@H]1CC[C@H](C)C2=CCC(=C[C@H]12)C |
| 4122 | O[C@H]1[C@@H](Oc2cc(O)cc(O)c2C1=O)c3ccc(O)c(O)c3 |
| 4123 | O[C@H]1[C@@H](Oc2cc([O-])cc(O)c2C1=O)c3ccc(O)c(O)c3 |
| 4124 | CC(C)c1ccc(C)c2ccc(C)cc12 |
| 4125 | C[C@]12CC[C@H](C1)C(C)(C)C2=O |
| 4126 | CCOC(=O)\C=C\c1ccc(OC)cc1 |
| 4127 | O=C(\C=C\c1ccccc1)c2ccccc2 |
| 4128 | CCc1ccc(O)c(OC)c1 |
| 4129 | O=CCCc1ccccc1 |
| 4130 | CC[C@@H](C)C(=O)[O-] |
| 4131 | CCOC(=O)\C=C\c1ccccc1 |
| 4132 | COc1ccc(C=O)cc1 |
| 4133 | Oc1ccc(CC=C)cc1 |
| 4134 | COc1ccccc1C=O |
| 4135 | C[C@H]1CCC(=C(C)C)CC2=C1CC[C@@H]2C |
| 4136 | Cc1ccc(C=O)cc1 |
| 4137 | OCCCc1ccccc1 |
| 4138 | CC(=O)C(=O)c1ccccc1 |
| 4139 | CC(=O)OC\C=C\c1ccccc1 |
| 4140 | COc1ccccc1\C=C\C=O |
| 4141 | CC(=O)OCc1ccccc1 |
| 4142 | Oc1ccccc1\C=C\C(=O)[O-] |
| 4143 | CCCCCCCCCCCCCCCCCC=O |
| 4144 | c1ccc2cc3ccccc3cc2c1 |
| 4145 | C=CC=O |
| 4146 | CC(C)C1=C2C=C(C)CC[C@H]2[C@H](C)CC1 |
| 4147 | C[C@H]1CC[C@@H](O)C[C@H]1C |
| 4148 | CCO[C@H](C)CO |
| 4149 | CC(C)CCOC(=O)c1ccccc1 |
| 4150 | CC(C)CCOC(=O)CC(C)C |
| 4151 | C[C@@H]1CC[C@H]2C(C)(C)[C@@H]3C[C@]12CC=C3C |
| 4152 | CCC(=O)CCCCCCC[C@H](C)CCCC(C)C |
| 4153 | COc1ccccc1\C=C\C(=O)[O-] |
| 4154 | [O-]C(=O)C1=Cc2cc3CCCN4CCCc(c2OC1=O)c34 |
| 4155 | C=C(C=O)c1ccccc1 |
| 4156 | CO\C=C\c1ccccc1 |
| 4157 | Oc1cccc(C=O)c1 |
| 4158 | C[C@@H](CCC=C(C)C)[C@@H]1C=C(C)CC[C@@H]1O |
| 4159 | COCCOC(=O)c1ccccc1C(=O)OCCOC |
| 4160 | CC1=CC(=O)N(C1=O)c2ccccc2 |
| 4161 | CC(=CCCC(=C)c1ccccc1)C |
| 4162 | C1Cc2ccccc12 |
| 4163 | O=C(CCC(=O)c1ccccc1)c2ccccc2 |
| 4164 | O=C(OCCc1ccccc1)c2ccccc2 |
| 4165 | CCOC(=O)c1ccccc1 |
| 4166 | COc1ccccc1\C=C/C(=O)[O-] |
| 4167 | Oc1ccccc1\C=C/C(=O)[O-] |
| 4168 | O=COCCc1ccccc1 |
| 4169 | COc1ccccc1CC(=O)C |
| 4170 | [O-]C(=O)\C=C\[C@H]1CC=CC=C1 |
| 4171 | COC(=O)\C=C/c1ccccc1 |
| 4172 | CCOC(=O)\C=C/c1ccccc1 |
| 4173 | CC(C)[C@@H](C)\C=C\[C@@H](C)[C@H]1CC[C@@H]2[C@]1(C)CC[C@@H]3[C@@]4(C)CC[C@H](O)C[C@@]45OO[C@@]23C=C5 |
| 4174 | CC(=O)OCCc1ccccc1 |
| 4175 | CC1(C)[C@H]2C[C@@H](O)C(=C)[C@@H]1C2 |
| 4176 | COc1ccccc1C(=O)[O-] |
| 4177 | CC1=C[C@H]2[C@@H](CC1)C(=C)CCCC2(C)C |
| 4178 | CC(C)c1ccc(O)cc1C |
| 4179 | COc1ccc(\C=C\C=O)cc1 |
| 4180 | N(c1ccccc1)c2ccc3ccccc3c2 |
| 4181 | C\C=C(\CC[C@@H](C)[C@H]1CC[C@H]2[C@@H]3CC=C4C[C@@H](O)CC[C@]4(C)[C@H]3CC[C@]12C)/C(C)C |
| 4182 | O=C1[NH2+]c2ccccc2S1 |
| 4183 | O=C1Nc2ccccc2S1 |
| 4184 | O=C1CCNc2ccccc12 |
| 4185 | Oc1cccc2C(=O)OCc12 |
| 4186 | C[C@@H]1CC[C@@H]2C(C)(C)[C@H]3C[C@@]12CC[C@@]3(C)O |
| 4187 | OC[C@H](Cc1ccccc1)NC(=O)[C@H](Cc2ccccc2)NC(=O)c3ccccc3 |
| 4188 | CO[C@@H]1O[C@@H]([C@H](O[C@@H]2O[C@H]([C@H](OC)[C@@H](O)[C@H]2O)C(=O)[O-])[C@@H](O)[C@H]1O)C(=O)[O-] |
| 4189 | O=C(OCCOCCOC(=O)c1ccccc1)c2ccccc2 |
| 4190 | [NH3+]CCOP(=O)([O-])OC[C@@H](COC=O)OC=O |
| 4191 | OC(c1ccccc1)(c2ccccc2)C(O)(c3ccccc3)c4ccccc4 |
| 4192 | CCCCC\C=C/C\C=C/CCCCCC=C |
| 4193 | CCCC#CC#CCCCOC(=O)CC(C)C |
| 4194 | O[C@H]1C[C@@](O)(C[C@@H](OC(=O)\C=C\c2ccc(O)c(O)c2)[C@@H]1O)C(=O)[O-] |
| 4195 | CC(=O)O[C@H]1CC[C@@]2(C)[C@H](CC[C@]3(C)[C@@H]2CC=C4[C@H]5CC(C)(C)CC[C@]5(C)CC[C@@]34C)C1(C)C |
| 4196 | COc1cc(\C=C/CO)cc(OC)c1O |
| 4197 | OC[C@H]1O[C@@H](OCc2ccccc2)[C@H](O)[C@@H](O)[C@@H]1O |
| 4198 | CC1(C)CCC[C@]2(C)OC(=O)C=C12 |
| 4199 | CC\C=C/C\C=C/C\C=C/CCCCCCCC(=O)OC |
| 4200 | CCCCCCCCCCCCCCCC=C |
| 4201 | OC[C@H]1O[C@@H](Oc2cc(O)c3C(=O)C(=C(Oc3c2)c4ccc(O)c(O)c4)[O-])[C@H](O)[C@@H](O)[C@@H]1O |
| 4202 | CCCCCCCCCCCCCCCC(=O)OCC(O)COC(=O)CCCCCCCCCCCCCCC |
| 4203 | Oc1cc(O)c2C(=O)C(=C(Oc2c1)c3cc(O)c(O)c(O)c3)[O-] |
| 4204 | Oc1cc(O)c2C(=O)C(=C(Oc2c1)c3cc(O)c([O-])c(O)c3)[O-] |
| 4205 | C\C\1=C\CCC(=C)[C@@H]2CC(C)(C)[C@H]2CC1 |
| 4206 | CC#CC#CC#CC#C\C=C\C=C |
| 4207 | CC\C=C/C\C=C/C\C=C/CCCCCC=C |
| 4208 | CCCCCCCCCCCCCCCC(=O)OC[C@H](CO)OC(=O)CCCCCCCCCCCCCCC |
| 4209 | CCOC(OCC)OCC |
| 4210 | C\C(=C/C=C/C=C(\C)/C=C/C=C(\C)/[C@@H]1O[C@]2(C)C[C@@H](O)CC(C)(C)C2=C1)\C=C\C=C(/C)\C=C\[C@H]3C(=C[C@H](O)CC3(C)C)C |
| 4211 | c1ccc2c(c1)c3cccc4cccc2c34 |
| 4212 | OC[C@@H](O)[C@H](O)[C@H](O)[C@@H](O)C=O |
| 4213 | CCCCC\C=C/C\C=C/C\C=C/CCCCC(=O)[O-] |
| 4214 | CC(=CCC\C(=C\CC\C(=C\CC[C@]1(C)CCc2cc(O)c(C)c(C)c2O1)\C)\C)C |
| 4215 | CCCCCCCC(=O)OC[C@@H](O)CO |
| 4216 | [O-]C(=O)C=O |
| 4217 | NC1=Nc2c(ncn2[C@H]3O[C@H](CO)[C@@H](O)[C@H]3O)C(=O)N1 |
| 4218 | CCCCCCCCCCCCCCCCCCCCC[C@@H](O)C[C@@H](O)CCCCCCC |
| 4219 | COc1cc(C)cc(OC)c1OC |
| 4220 | OC[C@H]1O[C@@H]([C@H](O)[C@@H](O)[C@@H]1O)C2=C([O-])\C(=C(/O)\C=C\c3ccc(O)cc3)\C(=O)[C@@](O)([C@@H]4O[C@H](CO)[C@@H](O)[C@H](O)[C@H]4O)C2=O |
| 4221 | OC[C@H]1O[C@@H]([C@H](O)[C@@H](O)[C@@H]1O)C2=C([O-])\C(=C(/[O-])\C=C\c3ccc(O)cc3)\C(=O)[C@@](O)([C@@H]4O[C@H](CO)[C@@H](O)[C@H](O)[C@H]4O)C2=O |
| 4222 | OC[C@H]1O[C@@H](OC2=C(Oc3cc(O)cc(O)c3C2=O)c4ccc(O)cc4)[C@@H](O)[C@@H](O)[C@@H]1O |
| 4223 | OC[C@H]1O[C@@H](OC2=C(Oc3cc(O)cc(O)c3C2=O)c4ccc([O-])cc4)[C@@H](O)[C@@H](O)[C@@H]1O |
| 4224 | COC1=CC(=C\C=C\C=C2C=C(OC)C(=O)C(=C2)OC)C=C(OC)C1=O |
| 4225 | CCOC(=O)[C@H]1[C@H](C)C(=O)c2cc(OC)c(OC)cc2[C@@H]1c3cc(OC)c(OC)c(OC)c3 |
| 4226 | COc1cc(cc(O)c1O)[C@@H]2OC[C@@H]3[C@H]2CO[C@@H]3c4cc(OC)c(O)c(OC)c4 |
| 4227 | CCCCCCCCCCCCCCCC(=O)O[C@@H]1CC[C@@]2(C)[C@@H](CC[C@]3(C)[C@@H]2CC[C@@H]4[C@H]5[C@@H](CC[C@]5(C)CC[C@@]34C)C(=C)C)C1(C)C |
| 4228 | OC[C@@H]1O[C@H](Oc2cc(O)c3C(=O)C=C(Oc3c2)c4ccc(O)c(O)c4)[C@@H](O[C@@H]5OC[C@@](O)(CO)[C@H]5O)[C@@H](O)[C@@H]1O |
| 4229 | OC[C@H]1O[C@@H](Oc2c(O)c(O)cc3O[C@@H](CC(=O)c23)c4ccc(O)cc4)[C@H](O)[C@@H](O)[C@@H]1O |
| 4230 | OC[C@H]1O[C@@H](Oc2c([O-])c(O)cc3O[C@@H](CC(=O)c23)c4ccc(O)cc4)[C@H](O)[C@@H](O)[C@@H]1O |
| 4231 | CCCCCCCCCCCCCCCCC=C |
| 4232 | [NH3+][C@H](Cc1ccccc1)C(=O)[O-] |
| 4233 | CCCCCCCCCCCCCCCCOC[C@H](COP(=O)([O-])OC[C@H]1O[C@H](C[C@@H]1N=[N+]=[N-])N2C=C(C)C(=O)NC2=O)OCC |
| 4234 | CC(=CCC\C(=C\CC\C(=C\CC\C(=C\C=C\C=C(/C)\CC\C=C(/C)\CC\C=C(/C)\CCC=C(C)C)\C)\C)\C)C |
| 4235 | CC(=CCC\C(=C\CC\C(=C\CC\C(=C\C=C\C=C(/C)\C=C\C=C(/C)\CC\C=C(/C)\CCC=C(C)C)\C)\C)\C)C |
| 4236 | C[C@H](\C=C/c1ccc(O)cc1)c2c(O)cc(O)c(CCO[C@@H]3O[C@H](O)[C@@H](O)[C@@H](O)[C@H]3O)c2O |
| 4237 | OC[C@@H](O)CO[C@@H]1O[C@H](O)[C@H]([C@H](O)[C@H]1O)[C@@H]2O[C@@H](O)[C@@H](O)[C@H]2O |
| 4238 | CC[C@@H](O)C=C |
| 4239 | Oc1ccc(cc1)C2=C([O-])C(=O)c3c(O)c(O)c([O-])cc3O2 |
| 4240 | Oc1c([O-])cc2OC(=C([O-])C(=O)c2c1O)c3ccc([O-])cc3 |
| 4241 | COc1cc(O)c2C(=O)C(=C(Oc2c1)c3ccc(O)cc3)OC[C@@H]4O[C@H](OC[C@@H](O)[C@@H](O)[C@H](O)[C@@H](O)C=O)[C@H](O)[C@H](O)[C@H]4O |
| 4242 | COc1cc(O)c2C(=O)C(=C(Oc2c1)c3ccc([O-])cc3)OC[C@@H]4O[C@H](OC[C@@H](O)[C@@H](O)[C@H](O)[C@@H](O)C=O)[C@H](O)[C@H](O)[C@H]4O |
| 4243 | C[C@]1(O)C(=C(C(C(=O)[O-])C2=C([O-])[C@](C)(O)C(=C(C(=O)\C=C/c3ccc(O)cc3)C2=O)[O-])C(=O)C(=C1[O-])C(=O)\C=C/c4ccc(O)cc4)[O-] |
| 4244 | Oc1ccc(\C=C\C(=O)C2=C([O-])C(=O)C(=O)C=C2[O-])cc1 |
| 4245 | Oc1ccc(cc1)[C@@H]2CC(=O)c3c(O)c(O)c(O)cc3O2 |
| 4246 | C[C@H](\C=C/c1ccc(O)cc1)c2c(O)cc(O)c(CCO)c2O |
| 4247 | Oc1ccc(cc1O)C2=C([O-])C(=O)c3c(O)c(O)c(O)cc3O2 |
| 4248 | O[C@H]1C(=O)C(=C(O)\C(=C(/O)\C=C\c2ccc(O)cc2)\C1=O)[C@@H](CC(=O)[O-])c3ccc(O)cc3 |
| 4249 | O[C@H]1C(=O)C(=C(O)\C(=C(/[O-])\C=C\c2ccc(O)cc2)\C1=O)[C@@H](CC(=O)[O-])c3ccc(O)cc3 |
| 4250 | OC[C@H]1O[C@H]2[C@H](OC3=C2C(=O)C(=C(O)C3(O)O)C(=O)\C=C/c4ccc(O)cc4)[C@@H](O)[C@H]1O |
| 4251 | OC[C@H]1O[C@H]2[C@H](OC3=C2C(=O)C(=C([O-])C3(O)O)C(=O)\C=C/c4ccc(O)cc4)[C@@H](O)[C@H]1O |
| 4252 | C[C@]1(O)C(=C(C([C@H](O)[C@@H](O)[C@H](O)[C@H](O)CO)C2=C([O-])[C@](C)(O)C(=C(C(=O)\C=C/c3ccc(O)cc3)C2=O)[O-])C(=O)C(=C1[O-])C(=O)\C=C/c4ccc(O)cc4)[O-] |
| 4253 | CC#CC#CC#CC#CC#CC=C |
| 4254 | OC[C@H]1O[C@H](OC2=C(Oc3cc(O[C@H]4O[C@H](CO)[C@@H](O)[C@H](O)[C@H]4O)cc(O)c3C2=O)c5ccc(O)c(O)c5)[C@H](O)[C@@H](O)[C@@H]1O |
| 4255 | OC[C@H]1O[C@@H](Oc2c(O)cc3OC(=C([O-])C(=O)c3c2O)c4ccc(O)c(O)c4)[C@H](O)[C@@H](O)[C@@H]1O |
| 4256 | CC#CC#CC#C\C=C\C=C\C=C |
| 4257 | OC[C@H]1O[C@H]([C@H](O)[C@@H](O)[C@@H]1O)C2=C([O-])\C(=C(/O)\C=C\c3ccc(O)cc3)\C(=O)[C@](O)([C@@H]4O[C@H](CO)[C@@H](O)[C@H](O)[C@H]4O)C2=O |
| 4258 | OC[C@H]1O[C@H]([C@H](O)[C@@H](O)[C@@H]1O)C2=C([O-])\C(=C(/[O-])\C=C\c3ccc(O)cc3)\C(=O)[C@](O)([C@@H]4O[C@H](CO)[C@@H](O)[C@H](O)[C@H]4O)C2=O |
| 4259 | OC[C@@H]1O[C@H]([C@H](O)[C@@H](O)[C@H]1O)[C@]2(O)C(=O)C(=C([O-])\C(=C(/O)\C=C\c3ccc(O)cc3)\C2=O)[C@H](CC(=O)[O-])c4ccc(O)cc4 |
| 4260 | OC[C@@H]1O[C@H]([C@H](O)[C@@H](O)[C@H]1O)[C@]2(O)C(=O)C(=C([O-])\C(=C(/[O-])\C=C\c3ccc(O)cc3)\C2=O)[C@H](CC(=O)[O-])c4ccc(O)cc4 |
| 4261 | OC[C@@H]1O[C@@H](O[C@@]2(O)C(=C(C(=O)\C=C/c3ccc(O)cc3)C(=O)C4=C2O[C@@H]5[C@@H](O)[C@@H](O)[C@@H](CO)O[C@H]45)O)[C@@H](O)[C@H](O)[C@@H]1O |
| 4262 | OC[C@@H]1O[C@@H](O[C@@]2(O)C(=C(C(=O)\C=C/c3ccc(O)cc3)C(=O)C4=C2O[C@@H]5[C@@H](O)[C@@H](O)[C@@H](CO)O[C@H]45)[O-])[C@@H](O)[C@H](O)[C@@H]1O |
| 4263 | OC[C@H](O)[C@H](O)[C@H](O)[C@H](O)[C@H](C1=C([O-])[C@@](O)(C[C@@H]2O[C@@H](CO)[C@H](O)[C@H](O)[C@H]2O)C(=C(C(=O)\C=C/c3ccc(O)cc3)C1=O)[O-])C4=C([O-])[C@@](O)(C[C@@H]5O[C@H](CO)[C@@H](O)[C@H](O)[C@H]5O)C(=C(C(=O)\C=C\c6ccc(O)cc6)C4=O)[O-] |
| 4264 | Oc1ccc(cc1)C2=CC(=O)c3c(O)c(O)c(O)cc3O2 |
| 4265 | Oc1cc2OC(=CC(=O)c2c(O)c1O)c3ccc([O-])cc3 |
| 4266 | CCCCCc1ccc(O)c(CCCCC)c1 |
| 4267 | OC[C@H]1O[C@@H](OC2=C(Oc3cc(O)c(O)c(O)c3C2=O)c4ccc(O)c(O)c4)[C@@H](O)[C@@H](O)[C@@H]1O |
| 4268 | CC(C)[C@H](NC(=O)[C@@H](CC(=O)[O-])NC(=O)[C@@H](CCCC[NH3+])NC(=O)[C@H]([NH3+])CCC[NH+]=C(N)N)C(=O)N[C@@H](Cc1ccc(O)cc1)C(=O)[O-] |
| 4269 | CC(C)[C@H](NC(=O)[C@@H](CC(=O)[O-])NC(=O)[C@@H](CCCC[NH3+])NC(=O)[C@H]([NH3+])CCCN=C(N)N)C(=O)N[C@@H](Cc1ccc(O)cc1)C(=O)[O-] |
| 4270 | CC(C)[C@H](NC(=O)[C@@H](CC(=O)[O-])NC(=O)[C@@H](CCCC[NH3+])NC(=O)[C@H](N)CCCN=C(N)N)C(=O)N[C@@H](Cc1ccc(O)cc1)C(=O)[O-] |
| 4271 | OC[C@@H]1[C@H](O)[C@@H](O)O[C@@H]([C@H]1O)[C@]2(O)C(=C(C(=O)\C=C/c3ccc(O)cc3)C(=O)C(=C2\C(=C/4\[NH2+][C@H](O)[C@@H](O)[C@H]4O)\O)[O-])[O-] |
| 4272 | OC[C@@H]1[C@H](O)[C@@H](O)O[C@@H]([C@H]1O)[C@]2(O)C(=C(C(=O)\C=C/c3ccc(O)cc3)C(=O)C(=C2\C(=C/4\N[C@H](O)[C@@H](O)[C@H]4O)\O)[O-])[O-] |
| 4273 | CCC[C@H](O)CC |
| 4274 | [NH3+][C@@H](Cc1c[nH]c2ccc(O)cc12)C(=O)[O-] |
| 4275 | CC(C)CC[C@@H](C)O |
| 4276 | CC(C)CCC[C@@H](C)CCC[C@@H](C)CCC[C@@H](C)O |
| 4277 | OC[C@@H]1O[C@H](OC2=C(Oc3cc(O[C@@H]4O[C@@H](CO)[C@H](O)[C@H](O)[C@H]4O)c(O[C@@H]5O[C@@H](CO)[C@H](O)[C@H](O)[C@H]5O)c(O)c3C2=O)c6ccc(O)cc6)[C@@H](O)[C@H](O)[C@H]1O |
| 4278 | OC[C@@H]1O[C@H](OC2=C(Oc3cc(O[C@@H]4O[C@@H](CO)[C@H](O)[C@H](O)[C@H]4O)c(O[C@@H]5O[C@@H](CO)[C@H](O)[C@H](O)[C@H]5O)c(O)c3C2=O)c6ccc([O-])cc6)[C@@H](O)[C@H](O)[C@H]1O |
| 4279 | C\C=C\C#CC#C\C=C\C=C\C=C |
| 4280 | OC[C@@H]1O[C@H](OC2=C(Oc3cc([O-])c(O)c(O)c3C2=O)c4ccc(O)cc4)[C@@H](O)[C@H](O)[C@H]1O |
| 4281 | OC[C@@H]1O[C@H](OC2=C(Oc3cc([O-])c(O)c(O)c3C2=O)c4ccc([O-])cc4)[C@@H](O)[C@H](O)[C@H]1O |
| 4282 | OC[C@@H]1O[C@@H](Oc2c(O)c3C(=O)C(=C(Oc3cc2[O-])c4ccc(O)cc4)OC[C@@H]5O[C@H](OC[C@@H](O)[C@@H](O)[C@H](O)[C@@H](O)C=O)[C@H](O)[C@H](O)[C@H]5O)[C@@H](O)[C@H](O)[C@H]1O |
| 4283 | OC[C@@H]1O[C@@H](Oc2c(O)c3C(=O)C(=C(Oc3cc2[O-])c4ccc([O-])cc4)OC[C@@H]5O[C@H](OC[C@@H](O)[C@@H](O)[C@H](O)[C@@H](O)C=O)[C@H](O)[C@H](O)[C@H]5O)[C@@H](O)[C@H](O)[C@H]1O |
| 4284 | OC[C@H]1O[C@H](OC2=C(Oc3cc(O)c(O)c(O)c3C2=O)c4ccc(O)cc4)[C@H](O)[C@@H](O)[C@@H]1O |
| 4285 | OC[C@H]1O[C@H](OC2=C(Oc3cc(O)c(O)c(O)c3C2=O)c4ccc([O-])cc4)[C@H](O)[C@@H](O)[C@@H]1O |
| 4286 | OC[C@@H]1O[C@H](OC2=C(Oc3cc([O-])c(O[C@@H]4O[C@@H](CO)[C@H](O)[C@@H](O)[C@@H]4O)c(O)c3C2=O)c5ccc(O)cc5)[C@@H](O)[C@H](O)[C@H]1O |
| 4287 | OC[C@@H]1O[C@H](OC2=C(Oc3cc([O-])c(O[C@@H]4O[C@@H](CO)[C@H](O)[C@@H](O)[C@@H]4O)c(O)c3C2=O)c5ccc([O-])cc5)[C@@H](O)[C@H](O)[C@H]1O |
| 4288 | OC[C@@H]1O[C@@H](Oc2cc3OC(=C([O-])C(=O)c3c(O)c2O[C@H]4O[C@@H](CO)[C@H](O)[C@H](O)[C@H]4O)c5ccc(O)cc5)[C@H](O)[C@@H](O)[C@H]1O |
| 4289 | OC[C@@H]1O[C@@H](Oc2cc3OC(=C([O-])C(=O)c3c(O)c2O[C@H]4O[C@@H](CO)[C@H](O)[C@H](O)[C@H]4O)c5ccc([O-])cc5)[C@H](O)[C@@H](O)[C@H]1O |
| 4290 | OC[C@H]1O[C@@H](Oc2cc3OC(=C([O-])C(=O)c3c(O)c2O)c4ccc(O)cc4)[C@H](O)[C@@H](O)[C@@H]1O |
| 4291 | OC[C@H]1O[C@@H](Oc2cc3OC(=C([O-])C(=O)c3c(O)c2O)c4ccc([O-])cc4)[C@H](O)[C@@H](O)[C@@H]1O |
| 4292 | Cc1nc2cc3NC(=O)NC(=O)c3cc2nc1C |
| 4293 | C\C=C/C#CC#C\C=C\COC(=O)CC(C)C |
| 4294 | CCCCCCCCCCCCCCCC(=O)OCC(COC(=O)CCCCCCCCCCCCCCC)OC(=O)\C=C\C=C\C |
| 4295 | Oc1ccc(\C=C/C(=O)OC(=O)C2=C(C(=O)c3cccc(C2=O)c3O)c4ccc(O)cc4)cc1 |
| 4296 | Oc1ccc(\C=C/C(=O)OC(=O)C2=C(C(=O)c3cccc(C2=O)c3O)c4ccc([O-])cc4)cc1 |
| 4297 | OC[C@H]1O[C@@H](O[C@@H]2[C@@H](O)[C@H](O)[C@@H](CO)O[C@H]2OC3=C(Oc4cc(O)cc(O)c4C3=O)c5ccc(O)cc5)[C@H](O)[C@@H](O)[C@@H]1O |
| 4298 | OC[C@H]1O[C@@H](O[C@@H]2[C@@H](O)[C@H](O)[C@@H](CO)O[C@H]2OC3=C(Oc4cc(O)cc(O)c4C3=O)c5ccc([O-])cc5)[C@H](O)[C@@H](O)[C@@H]1O |
| 4299 | O[C@H](CO[C@@H]1O[C@@H](COC2=C(Oc3cc([O-])cc(O)c3C2=O)c4ccc(O)cc4)[C@H](O)[C@@H](O)[C@H]1O)[C@@H](O)[C@H](O)[C@@H](O)C=O |
| 4300 | O[C@H](CO[C@@H]1O[C@@H](COC2=C(Oc3cc([O-])cc(O)c3C2=O)c4ccc([O-])cc4)[C@H](O)[C@@H](O)[C@H]1O)[C@@H](O)[C@H](O)[C@@H](O)C=O |
| 4301 | O[C@H](CO[C@H]1O[C@@H](COC2=C(Oc3cc(OC[C@H]4O[C@H](O)[C@H](O)[C@@H](O)[C@H]4O)cc(O)c3C2=O)c5ccc(O)cc5)[C@H](O)[C@@H](O)[C@H]1O)[C@@H](O)[C@H](O)[C@@H](O)C=O |
| 4302 | O[C@H](CO[C@H]1O[C@@H](COC2=C(Oc3cc(OC[C@H]4O[C@H](O)[C@H](O)[C@@H](O)[C@H]4O)cc(O)c3C2=O)c5ccc([O-])cc5)[C@H](O)[C@@H](O)[C@H]1O)[C@@H](O)[C@H](O)[C@@H](O)C=O |
| 4303 | Oc1ccc(\C=C\C(=O)NCCCCN(CCCNC(=O)\C=C\c2ccc(O)cc2)C(=O)\C=C\c3ccc(O)cc3)cc1 |
| 4304 | Oc1ccc(\C=C/C(=O)NCCCCN(CCCNC(=O)\C=C/c2ccc(O)cc2)C(=O)\C=C/c3ccc(O)cc3)cc1 |
| 4305 | OC[C@@H]1O[C@@H](OC2=C(Oc3cc(O[C@@H]4O[C@@H](CO)[C@H](O)[C@H](O)[C@H]4O)cc(O)c3C2=O)c5ccc(O)c(O)c5)[C@H](O)[C@@H](O)[C@H]1O |
| 4306 | OC[C@@H]1O[C@@H](OC2=C(Oc3cc(O[C@@H]4O[C@@H](CO)[C@H](O)[C@H](O)[C@H]4O)cc(O)c3C2=O)c5ccc([O-])c(O)c5)[C@H](O)[C@@H](O)[C@H]1O |
| 4307 | C\C=C/C#CC#CC#CC#CC=C |
| 4308 | CC(C)[C@H]1CC[C@@]2(C)[C@@H]3CC=C(C)[C@H]2[C@H]13 |
| 4309 | C\C(=C\CC[C@@]1(C)[C@@H]2CC[C@@H](C2)C1=C)\CO |
| 4310 | c1csc(c1)c2ccc(s2)c3cccs3 |
| 4311 | C\C=C/C#CC#CCCCOC(=O)CC(C)C |
| 4312 | CCCCCCCC\C=C/CCCCCCCC(=O)OC[C@H]1O[C@@H](O[C@H]2CC[C@]3(C)[C@H]4CC[C@]5(C)[C@H](CC[C@H]5[C@@H]4CC=C3C2)[C@H](C)CC[C@H](CC)C(C)C)[C@H](O)[C@@H](O)[C@@H]1O |
| 4313 | CC(C)CCC[C@@H](C)CCC[C@@H](C)CCC[C@]1(C)CCc2c(C)c(O)cc(C)c2O1 |
| 4314 | C\C=C\C#CC#CC#C\C=C/C=C |
| 4315 | c1ccc2scnc2c1 |
| 4316 | OC[C@H]1O[C@H](C[C@]2(O)C(=C([C@@H](C(=O)[O-])C3=C([O-])[C@@](O)(C[C@@H]4O[C@@H](CO)[C@H](O)[C@H](O)[C@H]4O)C(=C(C(=O)\C=C/c5ccc(O)cc5)C3=O)[O-])C(=O)C(=C2[O-])C(=O)\C=C\c6ccc(O)cc6)[O-])[C@H](O)[C@@H](O)[C@@H]1O |
| 4317 | OC[C@@H]1O[C@H](OC2=C(C(=O)\C=C\c3ccc(O)cc3)C(=CC(=O)C2=O)[O-])[C@H](O)[C@H](O)[C@H]1O |
| 4318 | Cc1cc2Oc3cc(O)cc(O[C@@H]4O[C@H](CO)[C@@H](O)[C@H](O)[C@H]4O)c3C(=O)c2cc1C |
| 4319 | Cc1cc2Oc3cc([O-])cc(O[C@@H]4O[C@H](CO)[C@@H](O)[C@H](O)[C@H]4O)c3C(=O)c2cc1C |
| 4320 | COc1cc(O)cc2C(=O)c3cc(C)cc(O)c3C(=O)c12 |
| 4321 | COc1cc(O)cc2C(=O)c3cc(C)cc([O-])c3C(=O)c12 |
| 4322 | CO[C@H]1C[C@H](O)[C@@H]2[C@H](C1)C(=O)c3cc(C)cc(O)c3C2=O |
| 4323 | CO[C@H]1C[C@H](O)[C@@H]2[C@H](C1)C(=O)c3cc(C)cc([O-])c3C2=O |
| 4324 | OC[C@H]1O[C@@H](Oc2c(O)cc(O)cc2\C=C\c3ccc(O)cc3)[C@H](O)[C@@H](O)[C@@H]1O |
| 4325 | OC[C@H]1O[C@@H](Oc2c([O-])cc(O)cc2\C=C\c3ccc(O)cc3)[C@H](O)[C@@H](O)[C@@H]1O |
| 4326 | O[C@@H]1Cc2c(O)cc(O)c([C@@H]3[C@H](OC(=O)c4cc(O)c(O)c(O)c4)[C@@H](Oc5cc(O)cc([O-])c35)c6ccc(O)c(O)c6)c2O[C@H]1c7ccc(O)c(O)c7 |
| 4327 | Cc1cc(O)c2c(O)c3c(O)cccc3cc2c1 |
| 4328 | CC(=O)c1c(O)cc(O)c(O[C@H]2O[C@@H](CO)[C@H](O)[C@@H](O)[C@@H]2O)c1O |
| 4329 | CC(=O)c1c(O)cc(O)c(O[C@H]2O[C@@H](CO)[C@H](O)[C@@H](O)[C@@H]2O)c1[O-] |
| 4330 | C[N@H+]1CCc2cc3OCOc3cc2[C@H]1[C@H]4OC(=O)c5c6OCOc6ccc45 |
| 4331 | CN1CCc2cc3OCOc3cc2[C@H]1[C@H]4OC(=O)c5c6OCOc6ccc45 |
| 4332 | COc1ccc2CC(=O)c3cc4OCOc4cc3CC[N@@H+](C)Cc2c1OC |
| 4333 | COc1ccc2CC(=O)c3cc4OCOc4cc3CCN(C)Cc2c1OC |
| 4334 | COc1cc(ccc1O[C@@H]2O[C@H](CO)[C@@H](O)[C@H](O)[C@H]2O)C(=O)C |
| 4335 | CC(=O)OC(C)(C)CCC(=O)[C@](C)(O)[C@H]1[C@H](O)C[C@@]2(C)[C@@H]3CC=C4[C@@H](C[C@H](O[C@@H]5O[C@H](CO)[C@@H](O)[C@H](O)[C@H]5O)C(=O)C4(C)C)[C@]3(C)C(=O)C[C@]12C |
| 4336 | CC(C)(O)\C=C\C(=O)[C@](C)(O)[C@H]1[C@H](O)C[C@@]2(C)[C@@H]3CC=C4[C@@H](C[C@H](O[C@@H]5O[C@H](CO)[C@@H](O)[C@H](O)[C@H]5O)C(=O)C4(C)C)[C@]3(C)C(=O)C[C@]12C |
| 4337 | COc1cc2CCN(C)[C@H]3Cc4ccc(Oc5cc(C[C@H]6[N@@H+](C)CCc7cc(OC)c(OC)c(Oc1cc23)c67)ccc5O)cc4 |
| 4338 | COc1cc2CCN(C)[C@H]3Cc4ccc(Oc5cc(C[C@H]6N(C)CCc7cc(OC)c(OC)c(Oc1cc23)c67)ccc5O)cc4 |
| 4339 | COc1ccc2cc3c4cc5OCOc5cc4[C@@H](O)C[n+]3cc2c1OC |
| 4340 | COc1ccc2C[C@@H]3[N@H+](C)CCc4cc5OCOc5c(c34)c2c1O |
| 4341 | COc1ccc2C[C@@H]3N(CCc4cc(OC)c(OC)c(O)c34)Cc2c1OC |
| 4342 | COc1cc2CCN3Cc4c(C[C@H]3c2c(O)c1OC)ccc(O)c4OC |
| 4343 | OC[C@H]1O[C@@H](Oc2ccc(O)c(c2)C(=O)[O-])[C@H](O)[C@@H](O)[C@@H]1O |
| 4344 | COc1cc2CC[n+]3cc4c(OC)c(OC)ccc4cc3c2cc1O |
| 4345 | COc1cc2CC[n+]3cc4c(OC)c(OC)ccc4cc3c2cc1[O-] |
| 4346 | C1Oc2cc3CC[n+]4cc5c6OCOc6ccc5cc4c3cc2O1 |
| 4347 | COc1cc2CCN3Cc4c(C[C@H]3c2cc1OC)ccc(O)c4OC |
| 4348 | COc1cc2CC[N@@H+](C)Cc2cc1O |
| 4349 | COc1cc2CCN(C)Cc2cc1O |
| 4350 | COc1cc2[C@@H]3Cc4ccc(OC)c(OC)c4CN3CCc2cc1O |
| 4351 | COc1cc2CCN(C)Cc3c(CC(=O)c2cc1OC)ccc4OCOc34 |
| 4352 | CN1Cc2c3OCOc3ccc2c4ccc5cc6OCOc6cc5c14 |
| 4353 | OC[C@H]1O[C@@H](OCCc2ccc(O)c(O)c2)[C@H](O)[C@@H](O)[C@@H]1O |
| 4354 | COc1cc2CC[n+]3cc4c5OCOc5ccc4cc3c2cc1OC |
| 4355 | C\C=C\1/CCNC1=O |
| 4356 | COc1cc(\C=C\C(=O)OC[C@H]2O[C@@H](O)[C@H](O)[C@@H](O)[C@@H]2O)ccc1O |
| 4357 | COc1cc2c(CC[n+]3cc4c5OCOc5ccc4cc23)cc1O |
| 4358 | COc1ccc(CCO[C@@H]2O[C@H](CO)[C@@H](OC(=O)\C=C\c3ccc(O)c(OC)c3)[C@H](O[C@@H]4O[C@H](CO)[C@@H](O)[C@H](O)[C@H]4O)[C@H]2O)cc1O |
| 4359 | COc1ccc2C[C@@H]3[N@@H+](C)CCc4c(OC)c(OC)c(OC)c(Oc5cc6[C@H](Cc7ccc(Oc1c2)cc7)N(C)CCc6cc5OC)c34 |
| 4360 | COc1ccc2C[C@@H]3N(C)CCc4c(OC)c(OC)c(OC)c(Oc5cc6[C@H](Cc7ccc(Oc1c2)cc7)N(C)CCc6cc5OC)c34 |
| 4361 | CC(=O)OC[C@H]1O[C@@H](O[C@@H]2OC=C[C@H]3[C@H](OC(=O)C)[C@@H]4O[C@]4(COC(=O)C)[C@@H]23)[C@H](OC(=O)C)[C@@H](OC(=O)C)[C@@H]1OC(=O)C |
| 4362 | COc1cc(ccc1OC(=O)C)C(=O)O[C@H]2[C@@H]3C=CO[C@@H](O[C@@H]4O[C@H](COC(=O)C)[C@@H](OC(=O)C)[C@H](OC(=O)C)[C@H]4OC(=O)C)[C@@H]3[C@@]5(COC(=O)C)O[C@@H]25 |
| 4363 | Compound4426   SciTegic10181810153D     66 68 0 0 0 0 999 V2000   -1.8406 1.4104 -0.1359 C 0 0 1 0 0 0   -2.5823 3.7151 0.0671 C 0 0 2 0 0 0   -1.5177 4.4890 0.8905 C 0 0 1 0 0 0   -0.3676 1.0520 -0.3351 C 0 0 2 0 0 0   -0.7684 5.5781 0.0962 C 0 0 2 0 0 0   -2.1149 2.6766 -0.7926 O 0 0   -3.4183 4.6119 -0.6771 O 0 0   -2.7500 0.3291 -0.7641 C 0 0 1 0 0 0   -0.9661 -1.2972 -0.6287 O 0 0   -0.1132 -0.4067 0.0848 C 0 0 1 0 0 0   -1.7283 6.4288 -0.7488 C 0 0 1 0 0 0   -2.7361 5.5588 -1.5237 C 0 0 1 0 0 0   -2.3501 -1.0873 -0.3103 C 0 0 2 0 0 0   5.0220 -4.8774 1.8753 C 0 0   4.3873 -6.0789 1.5469 C 0 0   4.5954 -3.6571 1.3558 C 0 0   0.4404 1.9287 0.4656 O 0 0   -2.1948 5.1702 1.9791 O 0 0   0.2669 5.0366 -0.7229 O 0 0   3.2960 -6.0455 0.6818 C 0 0   1.2567 -0.7027 -0.2101 O 0 0   -4.1050 0.5923 -0.3960 O 0 0   -2.4242 7.3567 0.0988 O 0 0   3.5135 -3.6234 0.4726 C 0 0   6.1044 -4.8682 2.7146 O 0 0   2.8687 -4.8261 0.1420 C 0 0   -3.1946 -2.1859 -1.0016 C 0 0   -2.1155 4.8831 -2.7585 C 0 0   4.9223 -7.1945 2.1338 O 0 0   1.6507 -1.9330 0.3991 C 0 0   -2.9259 -3.4885 -0.4453 O 0 0   -3.1036 4.0990 -3.4293 O 0 0   3.0368 -2.3166 -0.1097 C 0 0   4.4442 -8.4581 1.6857 C 0 0   -2.0596 1.5006 0.9365 H 0 0   -3.2838 3.2592 0.7767 H 0 0   -0.7985 3.8022 1.3403 H 0 0   -0.0820 1.2144 -1.3820 H 0 0   -0.2761 6.2360 0.8252 H 0 0   -2.6880 0.3994 -1.8578 H 0 0   -0.2661 -0.5142 1.1678 H 0 0   -1.1598 7.0378 -1.4611 H 0 0   -3.5412 6.2109 -1.8853 H 0 0   -2.4838 -1.2007 0.7739 H 0 0   5.1285 -2.7537 1.6396 H 0 0   2.7538 -6.9417 0.4031 H 0 0   2.0182 -4.8214 -0.5398 H 0 0   -2.9562 -2.2351 -2.0691 H 0 0   -4.2641 -1.9927 -0.8761 H 0 0   -1.2829 4.2199 -2.5166 H 0 0   -1.7483 5.6364 -3.4626 H 0 0   1.6678 -1.8053 1.4884 H 0 0   0.9272 -2.7198 0.1561 H 0 0   3.0281 -2.3901 -1.2048 H 0 0   3.7448 -1.5093 0.1170 H 0 0   5.0293 -9.2348 2.1871 H 0 0   3.3942 -8.5976 1.9624 H 0 0   4.5860 -8.5748 0.6061 H 0 0   1.3530 1.8237 0.1352 H 0 0   -2.4133 4.4892 2.6417 H 0 0   0.9008 5.7603 -0.8932 H 0 0   -4.3357 1.4644 -0.7721 H 0 0   -2.8249 6.8351 0.8256 H 0 0   6.3652 -5.7995 2.8856 H 0 0   -3.5114 -4.1270 -0.8967 H 0 0   -3.4092 3.4633 -2.7540 H 0 0   1 6 1 0   1 35 1 0   2 6 1 0   2 36 1 0   3 2 1 0   3 18 1 0   3 37 1 0   4 1 1 0   4 17 1 0   4 38 1 0   5 3 1 0   5 11 1 0   5 19 1 0   5 39 1 0   7 2 1 0   8 1 1 0   8 22 1 0   8 40 1 0   9 10 1 0   10 4 1 0   10 21 1 0   10 41 1 0   11 12 1 0   11 23 1 0   11 42 1 0   12 7 1 0   12 28 1 0   12 43 1 0   13 8 1 0   13 9 1 0   13 27 1 0   13 44 1 0   14 16 2 0   15 14 1 0   15 20 2 0   16 24 1 0   16 45 1 0   17 59 1 0   18 60 1 0   19 61 1 0   20 26 1 0   20 46 1 0   22 62 1 0   23 63 1 0   24 33 1 0   25 14 1 0   25 64 1 0   26 24 2 0   26 47 1 0   27 48 1 0   27 49 1 0   28 50 1 0   28 51 1 0   29 15 1 0   30 21 1 0   30 52 1 0   30 53 1 0   31 27 1 0   31 65 1 0   32 28 1 0   32 66 1 0   33 30 1 0   33 54 1 0   33 55 1 0   34 29 1 0   34 56 1 0   34 57 1 0   34 58 1 0  M END  > <Name>  Compound4426    > <Alias>  2-(3-Hydroxy-4-methoxyphenyl)-ethyl-O-beta-D-glucopyranosyl (1->3)-beta�CD-glucopyranoside    > <ID>  10442    > <Ionization pH>  7.5    > <IonizationIndex>  1    > <NumberOfIonizations>  1    > <source>  huanglian    > <Forcefield>  MMFF    > <ForcefieldBase>  MMFF    > <ForcefieldFFML>  <?xml version="1.0" encoding="UTF-8"?>  <!DOCTYPE ffml SYSTEM "Ffml.dtd">  <ffml version="1.0">  <forcefield name="MMFF" derivedFrom="MMFF" base="MMFF">  <atomTypes>  <atomType  name="C100"  element="C"  mass="12.011"  annotation=""/>  <atomType  name="O101"  element="O"  mass="16"  annotation=""/>  <atomType  name="H102"  element="H"  mass="1.008"  annotation=""/>  </atomTypes>  <parameters>  </parameters>  </forcefield>  </ffml>    > <Initial Potential Energy>  265.9633    > <Initial RMS Gradient>  11.12533    > <Minimization Criteria>  CONJUG> Minimization exiting with gradient tolerance ( 0.0100000) satisfied.    > <CHARMm Energy>  219.75918    > <Potential Energy>  219.75918    > <RMS Gradient>  0.00975    > <Bond Energy>  7.03052    > <Angle Energy>  20.3179    > <STRBend>  1.77098    > <Dihedral Energy>  34.49936    > <OOPLane>  0.00281    > <Van der Waals Energy>  49.8607    > <Electrostatic Energy>  106.27692    > <Hydrogen Bond Energy>  0    > <Solvation Free Energy>  0    > <HasGasteigerCharges>  true    > <REMARK99>  REMARK 99 MacroMolecule created from Mol2 file  REMARK 99 Compound4426  REMARK 99 PROTEIN  REMARK 99 GASTEIGER  REMARK 99  REMARK 99    > <DS no>  4363    $$$$ |
| 4364 | COc1ccc2C[C@H]3[N@H+](C)CCc4cc(OC)c(OC)c(Oc5cc6[C@H](Cc7ccc(Oc1c2)cc7)N(C)CCc6cc5OC)c34 |
| 4365 | COc1ccc2C[C@H]3N(C)CCc4cc(OC)c(OC)c(Oc5cc6[C@H](Cc7ccc(Oc1c2)cc7)N(C)CCc6cc5OC)c34 |
| 4366 | COc1cc2c(CC[n+]3cc4c(OC)c(OC)ccc4cc23)cc1O |
| 4367 | COc1cc(ccc1O)C(=O)OC[C@]23O[C@H]2[C@@H](O)[C@@H]4C=CO[C@@H](O[C@@H]5O[C@H](CO)[C@@H](O)[C@H](O)[C@H]5O)[C@H]34 |
| 4368 | COc1cc(ccc1[O-])C(=O)OC[C@]23O[C@H]2[C@@H](O)[C@@H]4C=CO[C@@H](O[C@@H]5O[C@H](CO)[C@@H](O)[C@H](O)[C@H]5O)[C@H]34 |
| 4369 | COc1cc(ccc1O)[C@H]2OC[C@H](Cc3ccc(O[C@@H]4O[C@H](CO)[C@@H](O)[C@H](O)[C@H]4O)c(OC)c3)[C@@H]2CO |
| 4370 | CC1(C)O[C@H]2CC(=O)OC[C@]23[C@H]4CC[C@@]5(C)[C@@H](OC(=O)[C@H]6O[C@@]56[C@]4(C)C(=O)C[C@@H]13)c7cocc7 |
| 4371 | COc1ccc2C[C@H]3c4c(CC[N+]3(C)C)cc(OC)c(O)c4c2c1O |
| 4372 | OC[C@@H](O)[C@@H](O)[C@@H](O)[C@@H](O)CO |
| 4373 | COc1ccc(\C=C\C(=O)O[C@H]2[C@@H]3C=CO[C@@H](O[C@@H]4O[C@H](CO)[C@@H](O)[C@H](O)[C@H]4O)[C@@H]3[C@@]5(CO)O[C@@H]25)cc1O |
| 4374 | CCCCCCCCCC(O)CCCCCCCCC |
| 4375 | CC1(C)O[C@H]2CC(=O)OC[C@]23[C@H]4CC[C@@]5(C)[C@@H](OC(=O)[C@H]6O[C@@]56[C@]4(C)C(=O)C[C@H]13)c7cocc7 |
| 4376 | COc1cc2CCN(C)[C@@H]3Cc4ccc(O)c(Oc5ccc(C[C@@H]6N(C)CCc7cc(OC)c(OC)c(Oc1cc23)c67)cc5)c4 |
| 4377 | CN1C(=O)c2c3OCOc3ccc2c4ccc5cc6OCOc6cc5c14 |
| 4378 | COc1cc2CCN(C)[C@@H](Cc3ccc(OC4=C[C@]5(C[C@H]6[N@H+](C)CCc7cc(OC)c(OC)c5c67)C=CC4=O)cc3)c2cc1OC |
| 4379 | COc1cc2CCN(C)[C@@H](Cc3ccc(OC4=C[C@]5(C[C@H]6N(C)CCc7cc(OC)c(OC)c5c67)C=CC4=O)cc3)c2cc1OC |
| 4380 | COC1=C[C@@]23CCN(C)[C@@H](Cc4cc(O)c(OC)cc24)C3=CC1=O |
| 4381 | COc1ccc2cc3c4cc(OC)c(OC)cc4CC[n+]3cc2c1OC |
| 4382 | CC(=O)OC[C@]12O[C@H]1[C@@H](OC(=O)C)[C@@H]3C=CO[C@H](O[C@@H]4O[C@H](COC(=O)\C=C\c5ccccc5)[C@@H](OC(=O)C)[C@H](OC(=O)C)[C@H]4OC(=O)C)[C@H]23 |
| 4383 | CC(C)(O)CCC(=O)[C@](C)(O)[C@H]1[C@H](O)C[C@@]2(C)[C@@H]3CC=C4[C@@H](C[C@H](O)[C@H](O)C4(C)C)[C@]3(C)CC[C@]12C |
| 4384 | CC(=O)c1ccc(O[C@@H]2O[C@H](CO)[C@@H](O)[C@H](O)[C@H]2O)cc1 |
| 4385 | OC[C@H]1O[C@@H](O[C@@H]2OC=C[C@H]3[C@H](O)[C@@H]4O[C@]4(COC(=O)\C=C/c5ccccc5)[C@@H]23)[C@H](O)[C@@H](O)[C@@H]1O |
| 4386 | COc1cc(ccc1O)C(=O)O[C@H]2[C@@H]3C=CO[C@@H](O[C@@H]4O[C@H](CO)[C@@H](O)[C@H](O)[C@H]4O)[C@@H]3[C@@]5(CO)O[C@@H]25 |
| 4387 | COc1cc(ccc1[O-])C(=O)O[C@H]2[C@@H]3C=CO[C@@H](O[C@@H]4O[C@H](CO)[C@@H](O)[C@H](O)[C@H]4O)[C@@H]3[C@@]5(CO)O[C@@H]25 |
| 4388 | COc1cc(\C=C\C(=O)O[C@H]2[C@@H]3C=CO[C@@H](O[C@@H]4O[C@H](CO)[C@@H](O)[C@H](O)[C@H]4O)[C@@H]3[C@@]5(CO)O[C@@H]25)ccc1O |
| 4389 | OCC[C@@H]1[C@@H](O)[C@@H](O)c2cocc12 |
| 4390 | O[C@H]1[C@@H](O)c2cocc2[C@@H]1CC(=O)[O-] |
| 4391 | COC(=O)C[C@@H]1[C@@H](O)[C@@H](O)c2cocc12 |
| 4392 | OC[C@H]1O[C@@H](O[C@H]2[C@H](O)[C@@H](COC(=O)\C=C\c3ccc(O)c(O)c3)O[C@@H](OCCc4ccc(O)c(O)c4)[C@@H]2O)[C@H](O)[C@@H](O)[C@@H]1O |
| 4393 | COc1ccc(CCO[C@@H]2O[C@H](COC(=O)\C=C\c3ccc(O)c(OC)c3)[C@@H](O)[C@H](O[C@@H]4O[C@H](CO)[C@@H](O)[C@H](O)[C@H]4O)[C@H]2O)cc1O |
| 4394 | COC1=C[C@@]23CCN(C)[C@@H](Cc4ccc(OC)c(O)c24)C3=CC1=O |
| 4395 | C[n+]1cc2c3OCOc3ccc2c4ccc5cc6OCOc6cc5c14 |
| 4396 | COc1cc2CCN3Cc4c(C[C@H]3c2cc1O)ccc(OC)c4O |
| 4397 | COc1cc(\C=C\C(=O)OC[C@H]2O[C@@H](OCCc3ccc(O)c(O)c3)[C@H](O)[C@@H](O[C@@H]4O[C@H](CO)[C@@H](O)[C@H](O)[C@H]4O[C@@H]5O[C@H](CO)[C@@H](O)[C@H](O)[C@H]5O)[C@@H]2O)ccc1O |
| 4398 | OC[C@H]1O[C@@H](O[C@H]2[C@H](O)[C@@H](CO)O[C@@H](OCCc3ccc(O)c(O)c3)[C@@H]2O)[C@H](O)[C@@H](O)[C@@H]1O |
| 4399 | C1Oc2cc3CC[N@H+]4Cc5c(C[C@@H]4c3cc2O1)ccc6OCOc56 |
| 4400 | C1Oc2cc3CCN4Cc5c(C[C@@H]4c3cc2O1)ccc6OCOc56 |
| 4401 | C[C@@H]1[C@H]2[N@@H+](CCc3cc4OCOc4cc23)Cc5c6OCOc6ccc15 |
| 4402 | C[C@@H]1[C@H]2N(CCc3cc4OCOc4cc23)Cc5c6OCOc6ccc15 |
| 4403 | COc1cc2CCN3Cc4c(C[C@H]3c2cc1OC)ccc(OC)c4OC |
| 4404 | COc1ccc2C[C@@H]3[N@@H+](C)CCc4c(OC)c(OC)c(OC)c(Oc5cc6C(=[NH+]CCc6cc5OC)Cc7ccc(Oc1c2)cc7)c34 |
| 4405 | COc1ccc2C[C@@H]3N(C)CCc4c(OC)c(OC)c(OC)c(Oc5cc6C(=[NH+]CCc6cc5OC)Cc7ccc(Oc1c2)cc7)c34 |
| 4406 | COc1cc2C[C@@H]3[N@H+](C)CCc4c(OC)c5OCOc5c(c2cc1OC)c34 |
| 4407 | COc1cc2C(=O)c3nccc4c(OC)c5OCOc5c(c2cc1OC)c34 |
| 4408 | COc1cc2C[C@@H]3N(C)CCc4c(OC)c(OC)c(OC)c(c34)c2cc1OC |
| 4409 | COc1c(O)ccc2CC(=O)c3cc4OCOc4cc3CC[N@@H+](C)Cc12 |
| 4410 | COc1c(O)ccc2CC(=O)c3cc4OCOc4cc3CCN(C)Cc12 |
| 4411 | COc1cc2CCN3Cc4c5OCOc5ccc4[C@@H](C)[C@@H]3c2cc1OC |
| 4412 | COc1ccc2C[C@@H]3[N@@H+](C)CCc4c(O)c(OC)c(OC)c(Oc5cc6[C@H](Cc7ccc(Oc1c2)cc7)N(C)CCc6cc5OC)c34 |
| 4413 | COc1ccc2C[C@@H]3N(C)CCc4c(O)c(OC)c(OC)c(Oc5cc6[C@H](Cc7ccc(Oc1c2)cc7)N(C)CCc6cc5OC)c34 |
| 4414 | COc1ccc2C[C@@H]3[N@@H+](C)CCc4c(OC)c(OC)c(OC)c(Oc5cc6[C@@H](Cc7ccc(Oc1c2)cc7)NCCc6cc5OC)c34 |
| 4415 | COc1ccc2C[C@H]3N(C)CCc4c(OC)c(OC)c(OC)c(Oc5cc6[C@H](Cc7ccc(Oc1c2)cc7)NCCc6cc5OC)c34 |
| 4416 | Oc1ccc(CCC(=O)[O-])c(O)c1O |
| 4417 | COc1cc(ccc1O)C(=O)OC[C@H]2O[C@@H](O[C@@H]3OC=C[C@H]4[C@H](O)[C@@H]5O[C@]5(CO)[C@@H]34)[C@H](O)[C@@H](O)[C@@H]2O |
| 4418 | COc1cc(ccc1[O-])C(=O)OC[C@H]2O[C@@H](O[C@@H]3OC=C[C@H]4[C@H](O)[C@@H]5O[C@]5(CO)[C@@H]34)[C@H](O)[C@@H](O)[C@@H]2O |
| 4419 | OC[C@H]1O[C@@H](O[C@@H]2OC=C[C@@H]3[C@H](OC(=O)c4ccccc4)[C@@H]5O[C@]5(CO)[C@@H]23)[C@H](O)[C@@H](O)[C@@H]1O |
| 4420 | COc1cc(cc(OC)c1OC)[C@@H]2Oc3c(OC)cc(\C=C\CO)cc3[C@H]2CO |
| 4421 | COc1cc(cc(OC)c1OC)[C@@H]2Oc3c(O)cc(\C=C\CO[C@@H]4O[C@H](CO)[C@@H](O)[C@H](O)[C@H]4O)cc3[C@H]2CO |
| 4422 | COc1cc(cc(OC)c1OC)[C@@H]2Oc3c(OC(=O)C)cc(\C=C\CO[C@@H]4O[C@H](CO)[C@@H](O)[C@H](O)[C@H]4O)cc3[C@H]2CO |
| 4423 | COc1cc(cc(OC)c1OC)[C@@H]2Oc3c(OC)cc(\C=C\CO[C@@H]4O[C@H](COC(=O)C(=C)CCO)[C@@H](O)[C@H](O)[C@H]4O)cc3[C@H]2CO |
| 4424 | COc1cc(cc(OC)c1OC)[C@@H]2Oc3c(OC)cc(\C=C\CO[C@@H]4O[C@H](COC(=O)C(=C)CCO)[C@@H](O)[C@H](O)[C@H]4O)cc3[C@H]2COC(=O)C |
| 4425 | COc1cc(ccc1O)[C@@H]2Oc3c(OC)cc(\C=C\CO[C@@H]4O[C@H](COC(=O)C(=C)CCO)[C@@H](O)[C@H](O)[C@H]4O)cc3[C@H]2CO |
| 4426 | COc1cc(ccc1[O-])[C@@H]2Oc3c(OC)cc(\C=C\CO[C@@H]4O[C@H](COC(=O)C(=C)CCO)[C@@H](O)[C@H](O)[C@H]4O)cc3[C@H]2CO |
| 4427 | Cc1c2c3cc4OCOc4cc3CC[n+]2cc5cc6OCOc6cc15 |
| 4428 | O[C@@H](C=O)[C@@H](O)[C@@H](O)[C@H](O)C(=O)[O-] |
| 4429 | C[C@@]1(O)CC[C@@H]2[C@H](OC(=O)C2=C)[C@@H]3[C@H]1CC[C@@]3(C)O |
| 4430 | O[C@H]1[C@@H](Oc2cc(O)cc(O)c2C1=O)c3ccc(O)cc3 |
| 4431 | O[C@H]1[C@@H](Oc2cc([O-])cc(O)c2C1=O)c3ccc(O)cc3 |
| 4432 | Cc1cc([O-])c2C(=O)c3c([O-])cc(O)cc3[C@@H]([C@H]4c5cccc([O-])c5C(=O)c6c(O)cc(CO)cc46)c2c1 |
| 4433 | Cc1cc([O-])c2C(=O)c3c([O-])cc(O)cc3[C@@H]([C@H]4c5cccc([O-])c5C(=O)c6c([O-])cc(CO)cc46)c2c1 |
| 4434 | COc1ccc2C[C@@H]3c4c(CC[N+]3(C)C)cc(OC)c(O)c4c2c1O |
| 4435 | COc1cc2CCN3Cc4c(C[C@@H]3c2cc1OC)ccc(OC)c4OC |
| 4436 | COc1cc2CCN3Cc4c(C[C@H]3c2cc1O)ccc(OC)c4OC |
| 4437 | CC(=CCc1c(O)cc(O)c2C(=O)[C@H](O)[C@H](Oc12)c3ccc(O)cc3)C |
| 4438 | CC1=CC(=O)CC(C)(C)C1=O |
| 4439 | COc1ccc2C[C@@H]3[N@@H+](CCc4cc5OCOc5cc34)Cc2c1OC |
| 4440 | COc1ccc2C[C@@H]3N(CCc4cc5OCOc5cc34)Cc2c1OC |
| 4441 | COc1ccc(C=O)cc1O |
| 4442 | COC(=O)[C@@]12OC[C@@]34[C@@H](C[C@@H](C(=O)C)[C@]5(C)CC(=O)O[C@@H]([C@@H]1OC(=O)C)[C@@H]35)OC(=O)[C@H](OC(=O)C)[C@@H]24 |
| 4443 | CN1C(=O)C2=C(OC(C)(C)C=C2)c3ccccc13 |
| 4444 | CCc1ccccc1O |
| 4445 | COc1cc(cc(OC)c1O)[C@@H]2[C@@H](CO)[C@@H](CO)Cc3cc(OC)c(O)c(OC)c23 |
| 4446 | COc1cc(cc(OC)c1O)[C@@H]2[C@@H](CO)[C@@H](CO)Cc3cc(OC)c([O-])c(OC)c23 |
| 4447 | CC(=O)O[C@H]1C[C@@H]2C(C)(C)OC(=O)C[C@@H](OC(=O)C)[C@]2(C)[C@@H]3CC[C@]4(C)[C@H](CC=C4[C@]13C)c5cocc5 |
| 4448 | CC(C)(O)[C@@H]1CC(=O)[C@]2(C)[C@H](CC[C@@]3(C)[C@@H](OC(=O)[C@H]4O[C@@]234)c5cocc5)[C@@]1(C)\C=C\C(=O)[O-] |
| 4449 | COc1cc2CC[N@@H+](C)[C@@H]3Cc4ccc(O)c(Oc5ccc(C[C@@H]6N(C)CCc7cc(OC)c(Oc(c1O)c23)cc67)cc5)c4 |
| 4450 | COc1cc2CCN(C)[C@@H]3Cc4ccc(O)c(Oc5ccc(C[C@@H]6N(C)CCc7cc(OC)c(Oc(c1O)c23)cc67)cc5)c4 |
| 4451 | COc1cc2CCN(C)[C@@H]3Cc4ccc(O)c(Oc5ccc(C[C@@H]6N(C)CCc7cc(OC)c(Oc(c1[O-])c23)cc67)cc5)c4 |
| 4452 | CC(C)(O)CCc1c(O)c2C(=O)[C@@H](O)[C@@H](Oc2cc1O[C@@H]3O[C@H](CO)[C@@H](O)[C@H](O)[C@H]3O)c4ccc(O)cc4 |
| 4453 | CC(C)(O)CCc1c(O)cc2O[C@@H]([C@@H](O)C(=O)c2c1O)c3ccc(O)cc3 |
| 4454 | CC(C)(O)CCc1c(O)c2C(=O)[C@H](O)[C@H](Oc2cc1[O-])c3ccc(O)cc3 |
| 4455 | COc1cc2C[N@+]3(C)CCc4cc(OC)c(O)cc4[C@@H]3Cc2cc1O |
| 4456 | COc1cc2CC[N@@+]3(C)Cc4cc(OC)c([O-])cc4C[C@H]3c2cc1O |
| 4457 | COc1cc2C[N@+]3(C)CCc4cc(OC)c([O-])cc4[C@@H]3Cc2cc1[O-] |
| 4458 | CC[C@@H](CC[C@@H](C)[C@H]1CC[C@H]2C3=CC[C@@H]4C[C@@H](O)CC[C@]4(C)[C@H]3CC[C@]12C)C(C)C |
| 4459 | [NH3+]CCc1ccccc1 |
| 4460 | COc1cc(CO)ccc1O[C@@H]2O[C@@H](CO)[C@@H](O)[C@@H](O)[C@@H]2O |
| 4461 | COc1cc(CO)ccc1O |
| 4462 | O=C1C=CC=C2N1C[C@H]3CCC[N@@H+]4CCC=C2[C@@H]34 |
| 4463 | O=C1C=CC=C2N1C[C@H]3CCCN4CCC=C2[C@@H]34 |
| 4464 | COc1c2ccoc2nc3ccccc13 |
| 4465 | CC(C)(O)CCc1c(O[C@@H]2O[C@H](CO)[C@@H](O)[C@H](O)[C@H]2O)cc(O)c3C(=O)C(=C(Oc13)c4ccc(O)cc4)[O-] |
| 4466 | CC(C)(O)CCc1c(O[C@@H]2O[C@H](CO)[C@@H](O)[C@H](O)[C@H]2O)cc(O)c3C(=O)C(=C(Oc13)c4ccc([O-])cc4)[O-] |
| 4467 | CC(C)(O)CCc1c([O-])cc(O)c2C(=O)C(=C(Oc12)c3ccc(O)cc3)[O-] |
| 4468 | CC(C)(O)CCc1c([O-])cc(O)c2C(=O)C(=C(Oc12)c3ccc([O-])cc3)[O-] |
| 4469 | C[C@@H](C[C@H](O)[C@H]1OC1(C)C)[C@@H]2CC[C@]3(C)C4=CC[C@@H]5C(C)(C)[C@H](O)CC[C@]5(C)[C@H]4CC[C@@]23C |
| 4470 | C[C@@H](C[C@@H](O)[C@H](O)C(C)(C)O)[C@@H]1CC[C@]2(C)C3=CC[C@@H]4C(C)(C)[C@@H](O)CC[C@]4(C)[C@H]3CC[C@@]12C |
| 4471 | CC(=O)O[C@H]1C[C@@H]2C(C)(C)OC(=O)C[C@@H](OC(=O)C)[C@]2(C)[C@@H]3CC[C@]4(C)[C@@H](CC=C4[C@]13C)[C@H]5COC(=O)C5 |
| 4472 | CC1(C)OC(=O)C=C[C@@]2(C)[C@H]1CC(=O)[C@]3(C)[C@@H]2CC[C@@]4(C)[C@@H](OC(=O)[C@H]5O[C@@]345)C6=CC(=O)O[C@H]6O |
| 4473 | C[C@H](C[C@@H](O)[C@@H]1OC1(C)C)[C@@H]2CC[C@]3(C)C4=CC[C@H]5C(C)(C)C(=O)CC[C@]5(C)[C@H]4CC[C@@]23C |
| 4474 | CC(=O)O[C@@H]1CC(=O)OC(C)(C)[C@@H]2CC(=O)[C@]3(C)[C@H](CC[C@@]4(C)[C@H](OC(=O)[C@H]5O[C@@]345)c6cocc6)[C@@]12C |
| 4475 | O=C1N2CCc3c([nH]c4ccccc34)C2=Nc5ccccc15 |
| 4476 | COc1ccc2c(OC)c3ccoc3[nH+]c2c1OC |
| 4477 | COc1ccc2c(OC)c3ccoc3nc2c1OC |
| 4478 | COc1cccc2c(OC)c3ccoc3nc12 |
| 4479 | COc1cc(\C=C\C(=C)O)ccc1O |
| 4480 | COc1ccc2c(ccc3c2ccc4cc5OCOc5cc34)c1OC |
| 4481 | CC[C@@H](C)C(=O)OC[C@]1(C)[C@H]2[C@@H]3C=C(COC(=O)C)C[C@@]4(O)[C@@H](C=C(C)C4=O)[C@@]3(O)[C@H](C)C[C@@]12OC(=O)Cc5ccccc5 |
| 4482 | CCCCC\C=C/C\C=C/CCCCCCCC(=O)Oc1cc(OC)c2CC[C@@](C)(CC(=O)C=C(C)C)Oc2c1C=O |
| 4483 | CC1(C)OC[C@@H](C[C@@H](O)[C@@H]1O)[C@@H]2CC[C@]3(C)C4=CC[C@@H]5C(C)(C)C(=O)CC[C@]5(C)[C@H]4CC[C@@]23C |
| 4484 | COc1ccc2cc3c4cc5OCOc5cc4CC[n+]3cc2c1O |
| 4485 | COc1ccc2cc3c4cc5OCOc5cc4CC[n+]3cc2c1[O-] |
| 4486 | O=C1NCCc2cc3OCOc3cc12 |
| 4487 | COc1cc2C[N@@+]3(C)CCc4cc(OC)c(O)cc4[C@H]3Cc2cc1O |
| 4488 | COc1cc2CC[N@+]3(C)Cc4cc(OC)c([O-])cc4C[C@@H]3c2cc1O |
| 4489 | COc1cc2C[N@@+]3(C)CCc4cc(OC)c([O-])cc4[C@H]3Cc2cc1[O-] |
| 4490 | CCOC(=O)\C=C\c1ccc(O)c(O)c1 |
| 4491 | CC1(C)O[C@H]2CC(=O)OC[C@]23[C@H]4CC[C@@]5(C)[C@@H](OC(=O)[C@H]6O[C@@]56[C@]4(C)C(=O)C[C@@H]13)c7cocc7 |
| 4492 | COc1cc(C)ccc1O |
| 4493 | O=C1CCC=C1 |
| 4494 | COC(=O)\C=C\c1ccc(O)c(O)c1 |
| 4495 | COc1ccc2nc3occc3c(OC)c2c1 |
| 4496 | C[C@@H](C[C@@H](O)[C@H](O)C(C)(C)O)[C@@H]1CC[C@]2(C)C3=CC[C@H]4C(C)(C)C(=O)CC[C@]4(C)[C@H]3CC[C@@]12C |
| 4497 | O=C1C=Cc2[nH+]ccc3c4ccccc4n1c23 |
| 4498 | O=C1C=Cc2nccc3c4ccccc4n1c23 |
| 4499 | CC(C)[C@H]1CCC(=C)[C@H](O)CCC(=C)\C=C\1 |
| 4500 | COc1cc(cc(OC)c1O)[C@@H]2OC[C@@H]3[C@H]2CO[C@H]3c4cc(OC)c(O)c(OC)c4 |
| 4501 | C[C@@H](C[C@@H](O)[C@H]1OC1(C)C)[C@@H]2CC[C@]3(C)C4=CC[C@@H]5C(C)(C)[C@H](O)CC[C@]5(C)[C@H]4CC[C@@]23C |
| 4502 | NC(=[NH2+])N |
| 4503 | NC(=N)N |
| 4504 | CC1(C)O[C@H]1[C@H]2C[C@H]([C@H](O)O2)[C@@H]3CC[C@]4(C)C5=CC[C@H]6C(C)(C)C(=O)CC[C@]6(C)[C@H]5CC[C@@]34C |
| 4505 | COC(C)(C)[C@@H](O)[C@H](O)C[C@H](C)[C@H]1CC[C@]2(C)C3=CC[C@@H]4C(C)(C)C(=O)CC[C@]4(C)[C@H]3CC[C@@]12C |
| 4506 | OCCc1cc2C=CC(=O)Oc2cc1[O-] |
| 4507 | COc1c(O)ccc2cc3c4cc5OCOc5cc4CC[n+]3cc12 |
| 4508 | COc1c([O-])ccc2cc3c4cc5OCOc5cc4CC[n+]3cc12 |
| 4509 | COc1cc(CO)ccc1O[C@@H]2O[C@H](CO)[C@@H](O)[C@H](O)[C@H]2O |
| 4510 | CCCCCC\C=C/CCCCCCCC=O |
| 4511 | CC[C@@H](C)C[C@@H](C)CC(C)C |
| 4512 | COC(=O)c1c(C)cc(O)c(C)c1O |
| 4513 | COC(=O)c1c(C)cc([O-])c(C)c1O |
| 4514 | C[N+](C)(C)CCc1ccc(O)cc1 |
| 4515 | CCCCCCCC\C=C\C(=O)[O-] |
| 4516 | COc1ccc(C)cc1OC |
| 4517 | CC1(C)OC(=O)C=C[C@@]2(C)[C@H]1CC(=O)[C@]3(C)[C@@H]2CC[C@@]4(C)[C@@H](OC(=O)[C@H]5O[C@@]345)c6cocc6 |
| 4518 | CC(=CCC\C(=C\COc1ccc2C=CC(=O)Oc2c1)\C)C |
| 4519 | CC(=O)O[C@@H]1CO[C@@H](O[C@@H]2CC[C@]34C[C@]35CC[C@]6(C)[C@H]([C@@H](O)C[C@@]6(C)[C@H]5C[C@@H](O[C@@H]7O[C@H](CO)[C@@H](O)[C@H](O)[C@H]7O)[C@H]4C2(C)C)[C@@]8(C)CC[C@H](O8)C(C)(C)O)[C@H](OC(=O)C)[C@H]1OC(=O)C |
| 4520 | C[C@@H]1O[C@@H](O[C@@H]2[C@@H](O)[C@H](O)CO[C@H]2O[C@H]3CC[C@]45C[C@]46CC[C@]7(C)[C@H]([C@@H](O)C[C@@]7(C)[C@H]6C[C@H](O)[C@@H]5C3(C)C)[C@@]8(C)CC[C@H](O8)C(C)(C)O[C@@H]9O[C@H](CO)[C@@H](O)[C@H](O)[C@H]9O)[C@H](O)[C@H](O)[C@H]1O |
| 4521 | C[C@@H]1O[C@@H](O[C@H]2[C@H](O[C@H]3CC[C@]45C[C@]46CC[C@]7(C)[C@H]([C@@H](O)C[C@@]7(C)[C@H]6C[C@H](O)[C@@H]5C3(C)C)[C@@]8(C)CC[C@H](O8)C(C)(C)O[C@@H]9O[C@H](CO)[C@@H](O)[C@H](O)[C@H]9O)OC[C@@H](O)[C@@H]2OC(=O)C)[C@H](O)[C@H](O)[C@H]1O |
| 4522 | C[C@@H]1O[C@@H](O[C@@H]2[C@@H](O)[C@H](O)CO[C@H]2O[C@H]3CC[C@]45C[C@]46CC[C@]7(C)[C@H]([C@@H](O)C[C@@]7(C)[C@@H]6C[C@H](O)[C@@H]5C3(C)C)[C@@]8(C)CC[C@H](O8)C(C)(C)O)[C@H](O)[C@H](O)[C@H]1O |
[truncated: 427,075 more chars]
